# Asymmetric [2+1] cycloaddition of difluoroalkyl-substituted carbenes with alkenes under rhodium catalysis: Synthesis of chiral difluoroalkyl-substituted cyclopropanes

**DOI:** 10.1016/j.isci.2022.105896

**Published:** 2023-02-15

**Authors:** Xinyu Zhang, Yongquan Ning, Chunqi Tian, Giuseppe Zanoni, Xihe Bi

**Affiliations:** 1 Department of Chemistry, Northeast Normal University, Changchun 130024, China; 2 Department of ChemistryUniversity of Pavia, Viale Taramelli 12, 27100 Pavia, Italy; 3 State Key Laboratory of Elemento-Organic Chemistry, Nankai University, Tianjin 300071, China

**Keywords:** Catalysis, Organic synthesis, Organic compound

## Abstract

Herein, we report a novel strategy for the synthesis of chiral difluoroalkyl-substituted cyclopropanes through enantioselective [2 + 1] cyclopropanation of alkenes and difluoroalkyl-substituted carbenes under rhodium catalysis, wherein the newly designed α, α-difluoro-β-carbonyl ketone *N*-triftosylhydrazones are used as the difluoroalkyl-substituted carbenes precursors. This approach represents the first asymmetric cyclopropanation of alkenes with difluoroalkyl carbenes, featuring high yield, high enantioselectivity, and broad substrate scope. Gram-scale synthesis and further interconversion of diverse functional groups demonstrate the usefulness of this protocol in the preparation of diverse functionalized chiral difluoroalkyl-substituted cyclopropanes.

## Highlights

• Chiral difluoromethylene cyclopropanes
• Difluoroalkyl-substituted carbenes
• Broad substrate scope

## Graphical abstract

**Figure:**
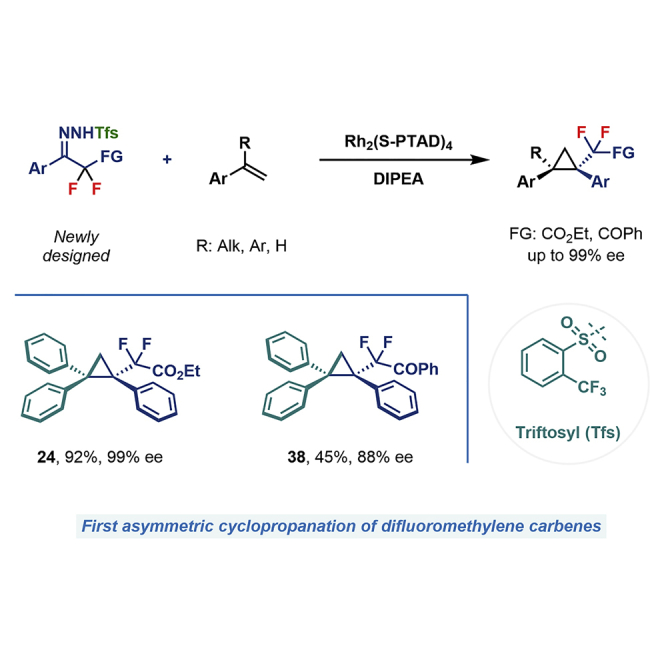

## Introduction

**Scheme 1:**
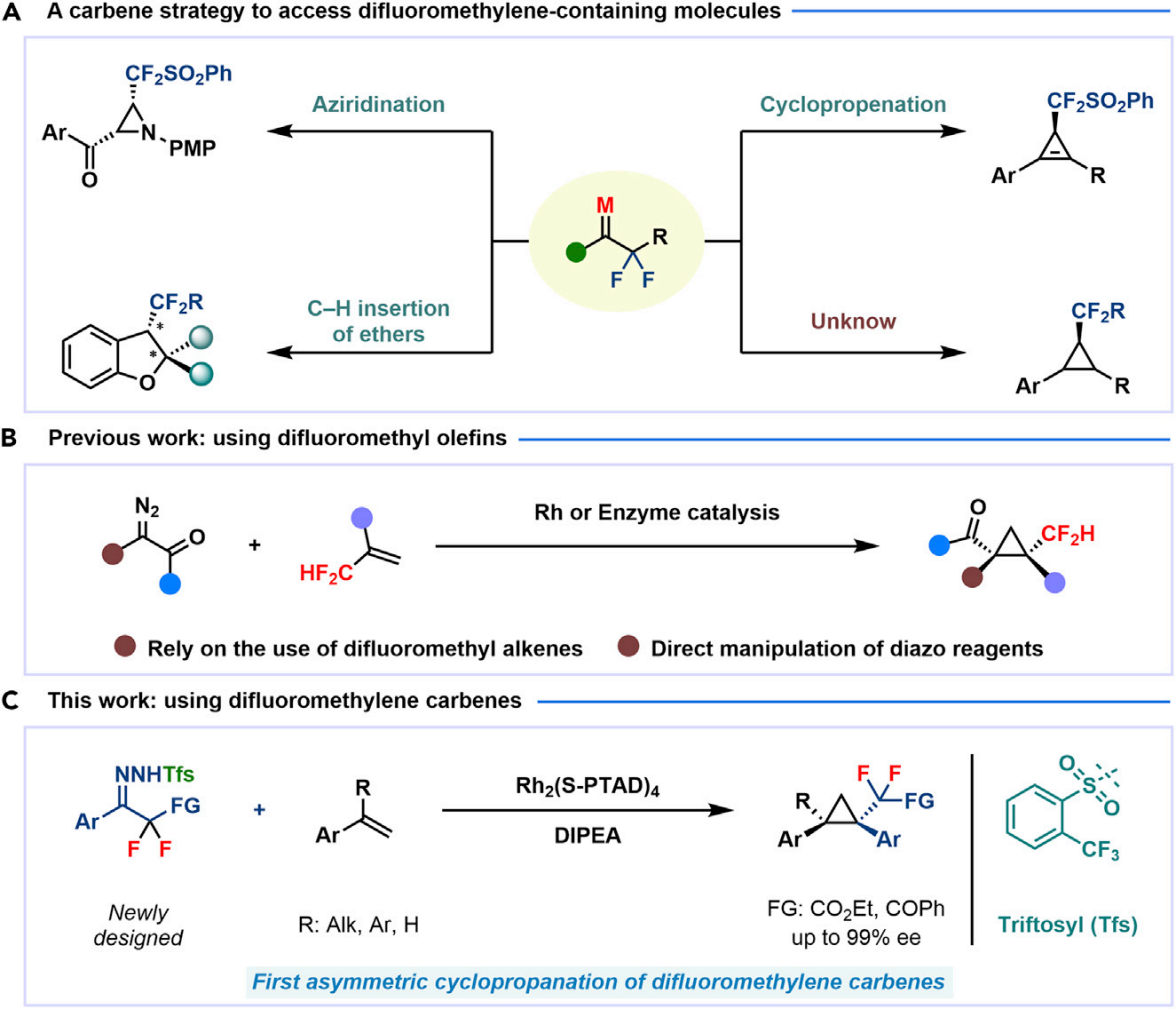
Synthetic strategies for chiral difluoromethylene- or difluoromethyl-substituted molecules

The synthesis of partially fluorinated chiral organic molecules has drawn increasing attention, owing to the applications of such substrates in the design and development of bioactive molecules and drugs.^1^^,^^2^^,^^3^^,^^4^ Difluoromethylene carbenes, which can considered as a suitable source of partially fluorinated moieties, have been widely used for the introduction of difluoroalkyl units into molecular skeletons.^5^^,^^6^^,^^7^^,^^8^^,^^9^ However, in sharp contrast to the significant advances in the asymmetric reaction of perfluoroalkyl carbenes,^5^^,^^6^^,^^7^^,^^8^^,^^9^^,^^10^^,^^11^^,^^12^^,^^13^^,^^14^^,^^15^^,^^16^^,^^17^^,^^18^ the study on the enantioselective reactions involving difluoromethylene carbenes is still in its very early stage. To date, only three types of reactions are known in the prior art, namely the enantioselective cyclopropenation and aziridination reactions developed by Ma and coworkers^19^^,^^20^ and our report of the asymmetric intramolecular C–H insertion of ethers (Scheme 1A).^21^ The [2 + 1] cyclopropanation represents one of the most typical reactions of carbenes; however, the analogous asymmetric reaction involving difluoroalkyl-substituted carbenes, which would allow for the synthesis of chiral difluoroalkyl-substituted cyclopropanes that have been found as the key structural unit of HIV inhibitors, remains undeveloped so far.^22^^,^^23^ This may be due to the low reactivity of the available difluoroalkyl-substituted carbenes and to the lack of appropriate carbene precursors. Regarding the synthesis of chiral difluoroalkyl cyclopropanes, only one strategy has been disclosed, which involves the cycloaddition of difluoromethyl olefins with diazo compounds under, respectively, rhodium and enzyme catalysis.^24^^,^^25^ On the other hand, the direct handling of hazardous diazo reagents remains until now the main disadvantage of this strategy. Consequently, an alternative strategy for asymmetric cyclopropanation of alkenes using difluoroalkyl-substituted carbenes is highly desirable.

As our continued interest in the chemistry of fluoroalkyl *N*-triftosylhydrazones,^26^^,^^27^^,^^28^^,^^29^^,^^30^ herein, we report the first enantioselective cyclopropanation of alkenes with difluoroalkyl-substituted carbenes *in situ* generated from α, α-difluoro-β-carbonyl ketone, which enables the successful application of difluoromethylene carbene in the asymmetric cyclopropanation. The use of *N*-triftosylhydrazones as difluoroalkyl-substituted carbenes surrogates avoids the need for direct manipulation of hazardous diazo reagents and, thus, reduces safety issues. It is worth mentioning that diverse alkenes, including aryl-alkyl, diaryl, and aryl terminal alkenes, are suitable in this protocol and have been proved to provide the chiral cyclopropanes in high yield and excellent enantioselectivity.^31^^,^^32^^,^^33^ More importantly, *N*-triftosylhydrazones, with the advantage of easy decomposition at relatively low temperature, plays an essential role in the success of this kind of novel asymmetric [2 + 1] cycloaddition reaction.

## Results and discussion

We began the study with the screening of chiral dirhodium catalyst for the cyclopropanation of suitable precursors in *α*-methyl-phenylethylene (**2**) with *N*-triftosylhydrazone (**1**). All the catalysts Rh_2_(*S*-DOSP)_4_, Rh_2_(*S*-PTTL)_4_, and Rh_2_(*S*-PTAD)_4_ afforded the desired difluoroalkyl-substituted cyclopropane in moderate yield at room temperature except with Rh_2_(*S*-PTAD)_4_ showing enantiocontrol up to 91% *ee* (Table 1, entry 1–3). Notably, the *D2* symmetric catalyst Rh_2_(*S*-BTPCP)_4_ was found to be ineffective in this reaction (entry 4), whereas the *C4* symmetric catalysts (Rh_2_(*S*-DOSP)_4_, Rh_2_(*S*-PTTL)_4_, and Rh_2_(*S*-PTAD)_4_) were more suitable for this [2 + 1] cycloaddition reaction.^34^^,^^35^ Commonly used bases, such as K_2_CO_3_, Cs_2_CO_3_, and *t*BuOK, were not suitable for the cyclopropenation reaction (entries 5–7). Performing the reaction in other solvents, such as toluene, DCM, and DMF, led to lower yield or no reaction (entries 8–10). Subsequently, a decrease in enantioselectivity was found when the reaction temperature was increased, which indicated the advantage of *N*-triftosylhydrazone decomposable at low temperatures (room temperature or below) in such asymmetric reactions (room temperature or lower) (entry 11 and 12).^19^^,^^20^^,^^28^ However, at low catalyst loadings (0.5 mol % of Rh_2_(*S*-PTAD)_4_), no significant changes in both yield and enantioselectivity were found (entry 13). Notably, low yield and enantioselectivity were observed when the reaction was run with 0.25 mol % of catalyst (entry 14). Surprisingly, when *α*-methyl-4-NO_2_-phenylethylene was used instead of *α*-methyl-phenylethylene, the desired product was obtained in 93% yield with 95% *ee* (entry 15). Based on these results, we speculated that the electronic setup of the substrate might have a significant effect on the yield of this reaction. Finally, the reaction condition as stated in entry 9 [DIPEA (2.0 equiv), Rh_2_(*S*-PTAD)_4_ (0.5 mol %), benzotrifluoride (TFT, 3.0 mL) at room temperature] was identified as the optimal condition for investigating the scope of this reaction.

**Table 1:** Optimization of the reaction conditions


Entry	Rh catalyst^a^	Base	Solvent	Temp.	Yield	*ee*
1	Rh_2_(*S*-DOSP)_4_ (1 mol %)	DIPEA	TFT	25°C	58%	59%
2	Rh_2_(*S*-PTTL)_4_ (1 mol %)	DIPEA	TFT	25°C	66%	87%
3	Rh_2_(*S*-PTAD)_4_ (1 mol %)	DIPEA	TFT	25°C	61%	91%
4	Rh_2_(*S*-BTPCP)_4_ (1 mol %)	DIPEA	TFT	25°C	*N.D.*	–
5	Rh_2_(*S*-PTAD)_4_ (1 mol %)	K_2_CO_3_	TFT	25°C	*N*.*R*.	–
6	Rh_2_(*S*-PTAD)_4_ (1 mol %)	CsCO_3_	TFT	25°C	*N*.*R*.	–
7	Rh_2_(*S*-PTAD)_4_ (1 mol %)	*t*BuOK	TFT	25°C	*N*.*R*.	–
8	Rh_2_(S-PTAD)_4_ (1 mol %)	DIPEA	Toluene	25°C	53%	82%
9	Rh_2_(S-PTAD)_4_ (1 mol %)	DIPEA	DCM	25°C	47%	20%
10	Rh_2_(S-PTAD)_4_ (1 mol %)	DIPEA	DMF	25°C	Trace	–
11	Rh_2_(*S*-PTAD)_4_ (1 mol %)	DIPEA	TFT	40°C	73%	87%
12	Rh_2_(*S*-PTAD)_4_ (1 mol %)	DIPEA	TFT	0°C	21%	–
13	Rh_2_(*S*-PTAD)_4_ (0.5 mol %)	DIPEA	TFT	25°C	61%	92%
14	Rh_2_(*S*-PTAD)_4_ (0.25 mol %)	DIPEA	TFT	25°C	50%	74%
15^b^	Rh_2_(*S*-PTAD)_4_ (0.5 mol %)	DIPEA	TFT	25°C	93%	95%


*N.D.*, Not detect.

a Reaction conditions: *N*-Triftosylhydrazone **1** (0.2 mmol), 1,1-diphenylethylene **2** (0.1 mmol), [Rh] catalyst (1 mol %), DIPEA (*N*, *N*-diisopropylethylamine) (0.2 mmol), TFT (benzotrifluoride, 3.0 mL), 12 h, under N_2_.

b *α*-Methyl-4-NO_2_-phenylethylene was used.

**Scheme 2:**
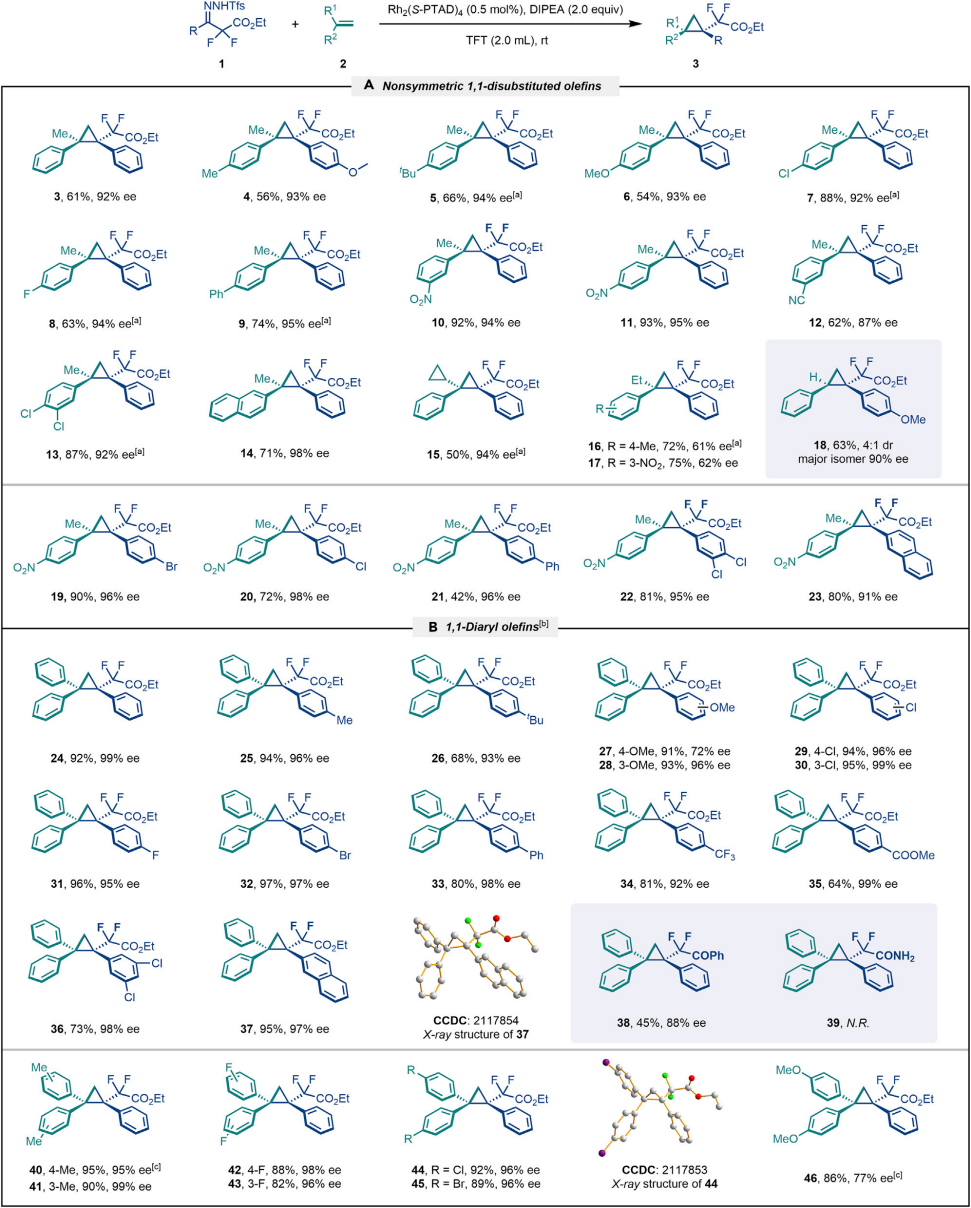
Reaction conditions: **1** (0.4 mmol), Rh_2_(*S*-PTAD)_4_ (0.5 mol %), **2** (0.2 mmol) and DIPEA (0.4 mmol) in TFT (3 mL) at rt (A) The enantiomer ratio was determined by converting the compound to an alcohol. (B) Rh_2_(*S*-PTAD)_4_ (0.25 mol %) was used. (C) DCM (3 mL) used as solvent. Diastereoselective ratio >20:1.

With the optimized condition in hand, initially, we directed our studies toward exploring the scope of *α*-methyl-phenylethylenes in this asymmetric [2 + 1] cycloaddition reaction. Remarkably, under the reaction condition as shown in Scheme 2A, *α*-methyl-phenylethylenes bearing a wide range of functional groups, such as methyl (**4**), *tert*-butyl (**5**), methoxy (**6**), halogen (**7**; **8**), phenyl (**9**), nitro (**10**), and cyano (**11**), were tolerated, leading to the desired chiral difluoroalkyl-substituted cyclopropanes (**3**–**12**) in moderate to high yield with excellent enantioselectivity. The substrates having electron-withdrawing and electron-donating substituents have no significant effect on enantioselectivity, albeit moderate yields were observed from the latter (**3**–**6**). The 3,4-dichloro phenyl and naphthyl-substituted *α*-methyl terminal alkenes were also found to be suitable coupling partners to afford the corresponding products in good yield and excellent enantioselectivity (**13** and **14**). By substituting methyl by cyclopropyl, the chiral difluoromethylene bicyclopropane (**15**) product was obtained in 94% *ee* with a moderate yield (50%; Scheme 2). Notably, such a bicyclopropane structural skeleton is present in a variety of protein inhibitors, antibiotic, and antifungal agents.^36^^,^^37^^,^^38^^,^^39^ Both methyl- and nitro-containing α-ethylstyrene substrates (**16** and **17**) responded well under these conditions to afford good yields (72%, 75%; Scheme 2), but with a significant decrease in enantioselectivity (61%, 62%). Styrene was also found to be suitable for this transformation and provided the chiral difluoroalkyl-substituted cyclopropane in 63% yield with 4:1 diastereoisomer ratio (**18**), wherein the major configuration retained high enantioselectivity (90% *ee*). We subsequently screened several difluoromethylene *N*-triftosylhydrazones with various substituents such as Br, Cl, phenyl, naphthyl etc., and were pleased to find that all of them reacted with excellent enantioselectivity (**19**–**23**).

Encouraged by these promising results, we envisioned that this strategy could be extended to the more sterically hindered 1,1-diarylethenes (Scheme 2B). After fine optimization of the reaction condition (for detail see Supplemental information), when the reaction was run under the catalysis of 0.25 mol % Rh_2_(*S*-PTAD)_4_ between 1,1-diphenylethylene and difluoromethylene hydrazone **1,** chiral triaryl difluoroalkyl-substituted cyclopropane **24** was obtained with remarkable enantiocontrol and high yield (92% yield, 99% *ee*). Subsequently, various structurally diverse difluoromethylene hydrazones were proved to be effective reaction components without having affected by any electronic and substituent effects. Substituents such as methyl (**25**), *tert*-butyl (**26**), methoxy (**27**, **28**), halogen (**29**–**32**), phenyl (**33**), trifluoromethyl (**34**), and ester (**35**) were well tolerated and the corresponding products were obtained in excellent yield and enantioselectivity. Multi-substituted aryl and naphthyl difluoromethylene hydrazones also underwent asymmetric cyclopropanation smoothly to provide the desired products (**36**, **37**) in 98% and 97% *ee*, respectively. The absolute configuration of **37** was assigned by *X*-ray crystallographic analysis. In addition, chiral difluorocarbonyl cyclopropane **38** could also be prepared with high enantioselectivity (88% *ee*) starting from difluorocarbonyl phenyl *N*-triftosylhydrazone. However, difluoramide phenyl *N*-triftosylhydrazone is not compatible with this reaction, mainly due to its low solubility which renders it incapable releasing the diazo compound in the reaction medium (**39**). This transformation also proved its usefulness, affording excellent yield and high enantioselectivity using various 1,1-diphenylethylene derivatives, which illustrates the potential of this method for the synthesis of diverse optically pure triaryl difluoroalkyl substituted (**40**–**46**).

**Scheme 3:**
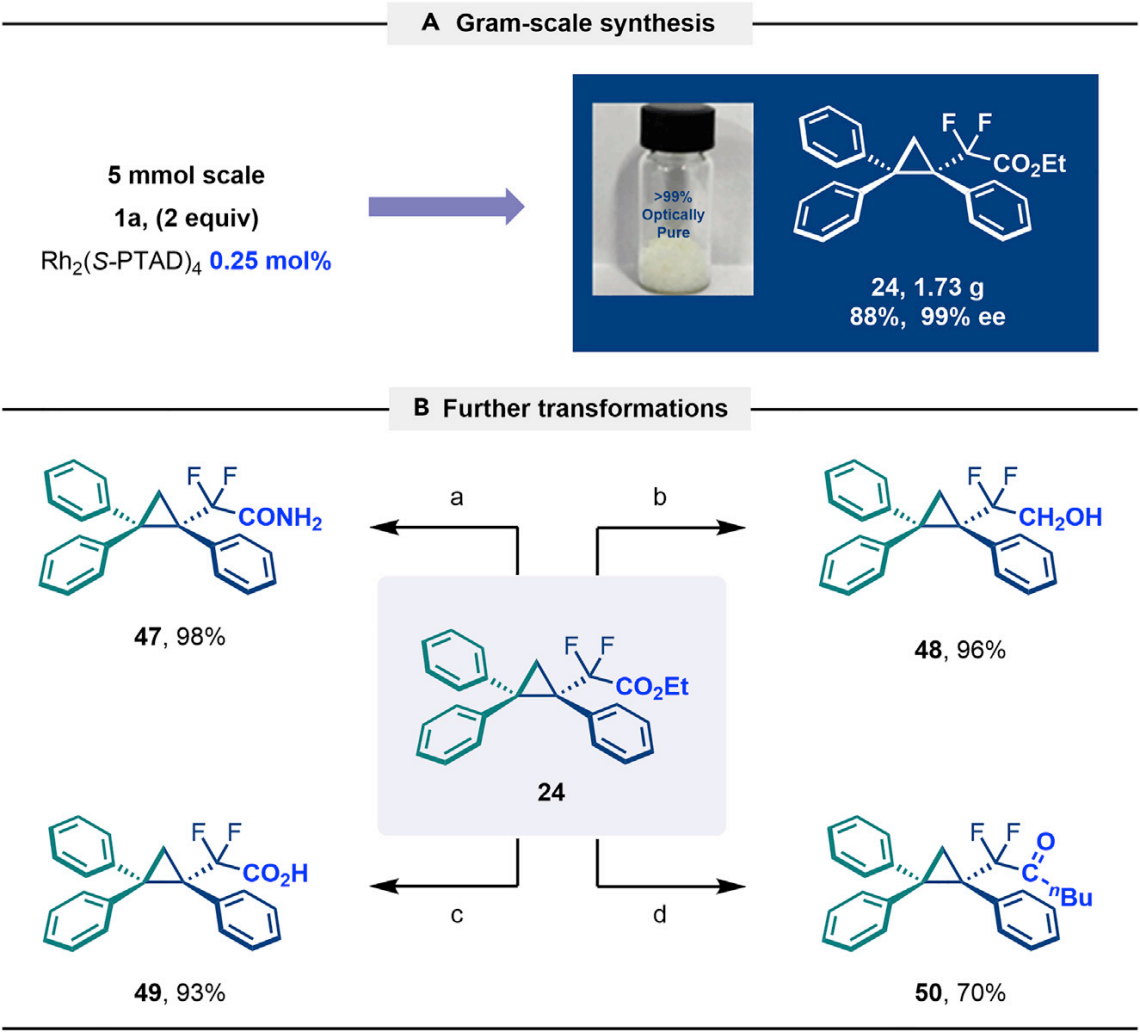
Gram-scale synthesis and functional-group transformations Reaction conditions (A) **24** (0.2 mmol), Ammonia (7 M in methanol) (0.5 mL), 25°C, 16 h. (B) **24** (0.5 mmol), LiAlH_4_ (0.75 mmol), THF (3 mL), N_2_, 0–25°C, 4 h. (C) **24** (0.2 mmol), NaOH (0.4 mmol), EtOH (2 mL), 60°C, 2 h. (D) **24** (0.2 mmol), ^n^BuLi (1.6 M in hexane, 0.25 mmol), THF (1 mL), N_2_, -78°C, 2.5 h.

The same synthetic protocol was demonstrated to be robust and applicable also on gram-scale synthesis. As shown in Scheme 3A, the cyclopropanation of 1,1-diphenylethylene can be scaled up to 5 mmol, affording **24** in high yield (1.73 g, 88%) with excellent enantioselectivity (99% *ee*) even with low catalyst loading Rh_2_(*S*-PTAD)_4_ (0.25 mol %). It is also noteworthy that the use of sulfonyl hydrazones instead of diazo compounds can significantly reduce the safety risk during large-scale synthesis and simplify operational procedures. Taking into account the ester group interconversion, we subsequently carried out the functionality derivatization of the optically pure product **24**. As demonstrated in Scheme 3B, diversely functionalized chiral difluoroalkyl-substituted cyclopropane derivatives were readily obtained from **24**. The latter was converted into amide **47** (98% yield), alcohol **48** (96% yield), acid **49** (93% yield), and ketone (**50**, 70% yield) using appropriate reagents.^40^

**Scheme 4:**
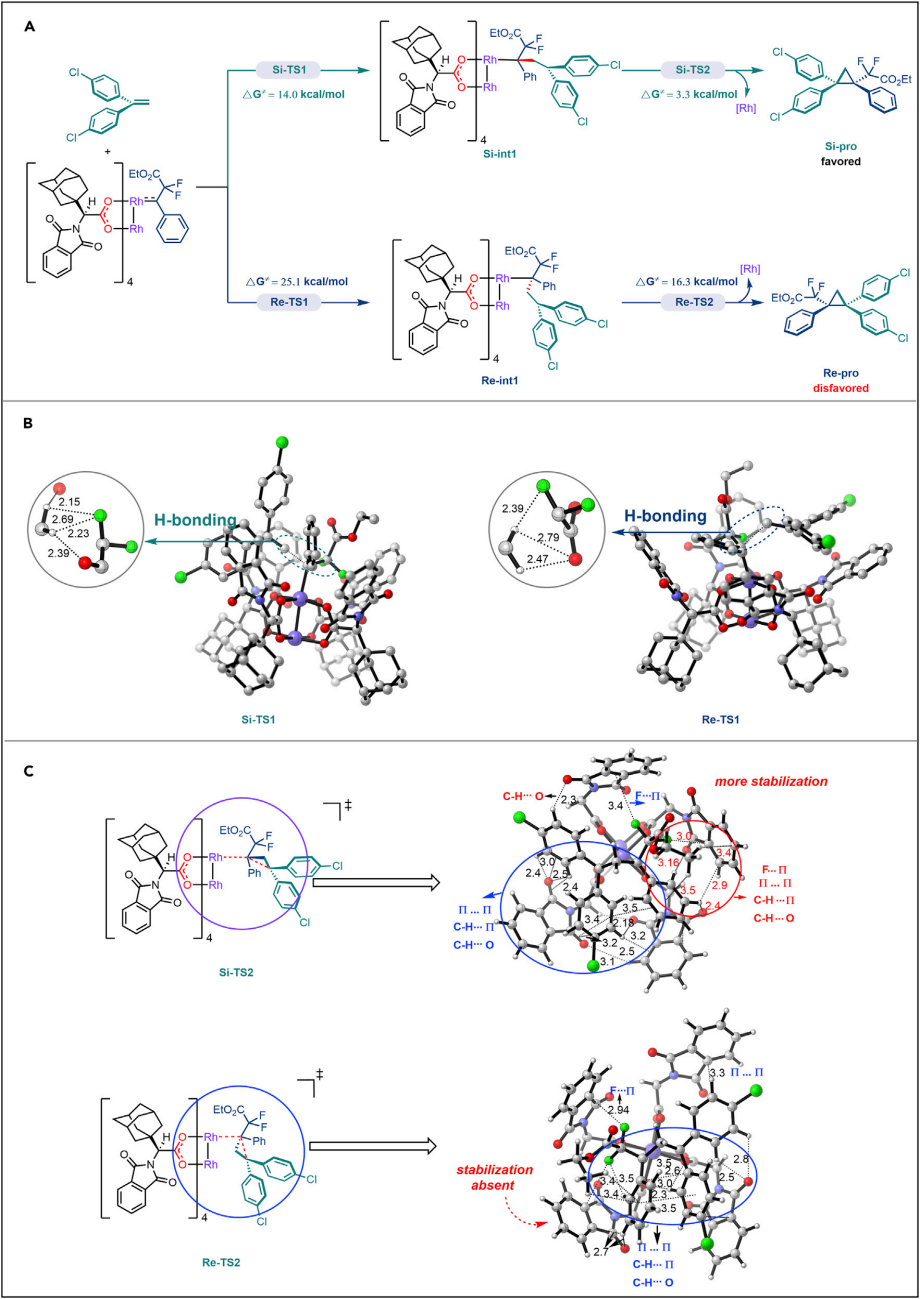
Theoretical research on proposed asymmetric [2 + 1] cycloaddition process at M06L/6-31G (d)-SDD(Rh) level of theory The computed relative free energies are in kcal/mol and the values in (B) and (C) are in Å.

Experimental outcome has shown that the Rh_2_(*S*-PTAD)_4_ catalyst has a highly stereo-selectivity for this asymmetric [2 + 1] cycloaddition process. To better understand the origin for the stereo-selectivity, a plausible mechanism was proposed using the cycloaddition of *N*-triftosylhydrazone **1,** which could be decomposed *in situ* to form the diazo compound under alkaline conditions,^41^ with 1,1-diphenylethylene as model and rationalized by density functional theory calculations at M06L/6-31G(*d*)-SDD(Rh) level of theory (for detailed computational methods, see Supplemental information). As shown in Scheme 4, the cycloaddition process is stepwisely owing to the geometric constraints derived from the body cavity of rhodium carbene. Firstly, the nucleophilic attack of 1,1-diphenylethylene to the carbene carbon can occur separately from the *Si-* or *Re-*plane of the carbene carbon, which determines the stereoselectivity of the product (Δ**G**^**≠**^(**Si-TS1**) = 14.0 kcal/mol; Δ**G**^**≠**^(**Re-TS1**) = 25.1 kcal/mol) (Scheme 4A). The non-covalent interactions analysis (Scheme 4B) shows that **Si-TS1** displayed stronger hydrogen bond interactions (C-H···F, C-H···O) than **Re-TS1**, resulting in a more stable transition state and thus a lower kinetic energy barrier. Subsequently, the cyclization process from **Si-int1** and **Re-int1** to form the final enantioselective product via **Si-TS2** (Δ**G**^**≠**^ **=** 3.3 **kcal/mol**) and **Re-TS2** (Δ**G**^**≠**^ **=** 16.3 **kcal/mol**) occurred. The lower energy barrier for **Si-TS2** may be derived from the more and stronger interactions exist in the configuration (See the part circled in red; Scheme 4C, upside), which renders the transition state more stable. Therefore, the formation of **Si-pro** is both kinetically and thermodynamically more favorable than **Re-pro**; this is in consistence with the experimental outcomes.

In summary, we have reported the first asymmetric [2 + 1] cycloaddition reaction of alkenes with difluoroalkyl-substituted carbenes, which were generated *in situ* from the easily decomposable α,α-difluoro-β-carbonyl ketone *N*-triftosylhydrazones. This protocol enables the enantioselective cyclopropanation of difluoroalkyl-substituted carbenes with alkenes, thereby providing a facile route to access enantioenriched difluoroalkyl-substituted cyclopropanes in high yield and excellent enantioselectivity. No doubt that the α, α-difluoro-β-carbonyl ketone *N*-triftosylhydrazones as the difluoroalkyl-substituted carbenes surrogates would find wide applications in asymmetric difluoroalkyl carbene transfer reactions in the near future.

### Limitations of the study

This study is limited to the terminal alkenes, and it is still necessary to further develop an asymmetric catalytic system suitable for internally substituted alkenes.

## STAR★Methods

### Key resources table

**Table:** 

REAGENT or RESOURCE	SOURCE	IDENTIFIER
**Chemicals, peptides, and recombinant proteins**		

2-(Trifluoromethyl)benzenesulfonyl chloride	776-04-5	Energy Chemical
DIPEA	7087-68-5	Energy Chemical
Rh_2_(*S*-PTAD)_4_	909389-99-7	J&K Scientific
TFT(Benzotrifluoride)	98-08-8	Energy Chemical
Hydrazinium hydroxide solution	10217-52-4	Energy Chemical

### Resource availability

#### Lead contact

Further information and requests for resources and reagents should be directed to and will be fulfilled by the lead contact, Xihe Bi (bixh507@nenu.edu.cn).

#### Materials availability

This study did not generate new unique reagents.

All reagents were purchased from commercial sources (Energy Chemical, Adamas-beta®, J&K Scientific, Sigma-Aldrich) and used without purification unless otherwise mentioned. The products were purified by column chromatography over silica gel (200–400 size). ^1^H, ^13^C and ^19^F nuclear magnetic resonance (NMR) spectra were recorded at 25°C on a Bruker 600 MHz, 150 MHz and 565 MHz or Bruker 500 MHz, 125 MHz and 470 MHz (TMS was used as internal standard). High resolution mass spectra (HRMS) were recorded on Waters Premier GC-TOF MS by using EI method or Bruck microTof by using ESI method. High-pressure liquid chromatography (HPLC) was performed on Agilent 1220 Series chromatographs using a chiral column (25 cm) as noted for each compound. Enantiomer excess was determined by HPLC analysis employing Darcel Chiracel AD-H, OD-H, OJ-H, AS-H or IF column.

### Method details

#### General procedure for the asymmetric cyclopropanation

In a nitrogen-filled glovebox, a flame-dried screw-cap reaction tube equipped with a Teflon-coated magnetic stir bar was charged with sulfonyl hydrazone 1 (0.8 mmol) and anhydrous TFT solvent (1.0 mL). The reaction mixture was stirred until complete dissolve, DIPEA (0.8 mmol) was added to the reaction system. The alkene 2 (0.4 mmol) was dissolved in 1.0 mL of anhydrous TFT and added to the reaction system. Rh_2_(*S*-PTAD)_4_ (0.5 mol%) was dissolved in anhydrous TFT (1 mL) and then added to the reaction system. The mixture was stirred at 25°C for 12 h until the reaction was complete as indicated by ^1^H NMR. The reaction crude was filtered through a short silica gel eluting with DCM. The filtrate was evaporated under reduced pressure to leave a crude mixture, which was separated by flash column chromatography to afford the pure product.

Note: The yield of the reaction decreases as the dosage of DIPEA decreases, but the enantioselectivity remains unchanged.

Synthesis of racemate: The same procedure uses Rh_2_(esp)_2_ instead of Rh_2_(*S*-PTAD)_4_ and performs at room temperature.

#### General procedure for the synthesis of olefins

A sealed flask was charged with benzaldehyde (10 mmol), then THF (30 mL) was added and stirred at 0°C. Grignard reagent (15 mmol) was slowly added and the mixture was placed at room temperature for 4 h. When the reaction was complete (monitored by TLC), saturated NH_4_Cl was added. The mixture was extracted with EA (30 mL × 3), then the organic phase was dried with anhydrous Na_2_SO_4_, concentrated under reduced pressure to give the pure compound.

#### Synthesis of benzophenones

A round-bottomed flask was charged with alcohol and dissolved in DCM (40 mL). Then Dess-Martin oxidant (1.5 equiv) was slowly added and stirred at room temperature overnight. After completion of the reaction (monitored by TLC), saturated NaHCO_3_ and Na_2_S_2_O_3_ solution were slowly added. Extracted with DCM (30 mL × 3) and washed the organic phases with brine, then dried with anhydrous Na_2_SO_4_. The residue was purified by flash column chromatography to give the corresponding benzophenone compound.

#### Synthesis of olefins

A sealed flask was charged with methyltriphenylphosphine bromide (1.5 equiv), THF (30 mL) was added and stirred at 0°C. Potassium tert-butoxide (1.5 equiv) and ketone (1.0 equiv) in THF (20 mL) was slowly added, the mixture was reacted at room temperature for 4 h. After completion of the reaction (monitored by TLC), NH_4_Cl solution was slowly added. The mixture was extracted with EA (30 mL × 3) and dried with anhydrous Na_2_SO_4_. Then concentrated under reduced pressure to give the pure compound.perform flash column chromatography.

#### General procedure for the synthesis of compound 47

A sealed reaction tube was charged with 24 (0.2 mmol) and ammonia (7 M in methanol) (0.5 mL). The mixture was stirred at 25°C for 16 h. Then the mixture was concentrated under reduced pressure to give the pure compound.

#### General procedure for the synthesis of compound 48

A sealed reaction tube was charged with LiAlH_4_ (0.3 mmol) under nitrogen atmosphere. 24 (0.2 mmol) in THF (3.0 mL) was added to the reaction tube under 0°C. Then the mixture was stirred at 25°C for 4 h. After the reaction was complete (monitored by TLC), aqueous NH_4_Cl was added, and quenched with EA. The organic phase was dried over anhydrous Na_2_SO_4_ and concentrated under reduced pressure to give the pure compound.

#### General procedure for the synthesis of compound 49

A bottom flask was charged with 24 (0.2 mmol), NaOH (0.4 mmol) and EtOH (2.0 mL). The mixture was stirred at 60°C for 2 h. After the reaction was complete (monitored by TLC), aqueous NH_4_Cl was added, and quenched with EA. The organic phase was dried over anhydrous Na_2_SO_4_ and concentrated under reduced pressure to give the pure compound.

#### General procedure for the synthesis of compound 50

A sealed reaction tube was replaced nitrogen three times, then 24 (0.2 mmol) in dry THF (3.0 mL) was added. The mixture was placed at −78°C, ^*n*^BuLi (1.6 M in hexane, 0.25 mmol) was added dropwise and stirred for 2.5 h. After the reaction was complete, aqueous NH_4_Cl was added, and quenched with CH_3_CO_2_Et. The organic phase was dried over anhydrous Na_2_SO_4_ and concentrated under reduced pressure and purified by column chromatography to give the pure compound.

#### Characterization data for the products

**Figure:**
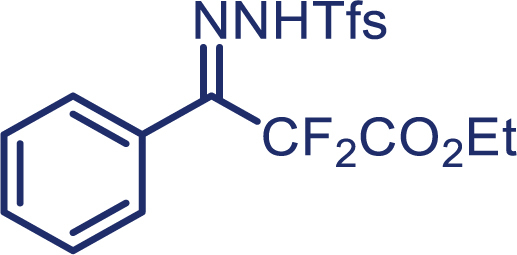

**1a**, White solid; m.p: 80–81°C; ^**1**^**H NMR** (500 MHz, CDCl_3_) δ 8.30–8.27 (m, 1H), 8.10 (s, 1H), 7.92–7.88 (m, 1H), 7.81–7.77 (m, 2H), 7.58–7.52 (m, 3H), 7.28–7.26 (m, 2H), 4.33 (q, *J* = 7.2 Hz, 2H), 1.35 (t, *J* = 7.2 Hz, 3H). ^**13**^**C NMR** (125 MHz, CDCl_3_) δ 162.0 (t, *J* = 31.0 Hz), 146.8 (t, *J* = 27.5 Hz), 136.0, 133.9, 133.5, 132.5, 131.5, 129.9, 128.4 (q, *J* = 6.3 Hz), 128.2, 127.7 (q, *J* = 32.3 Hz), 124.9, 122.6 (q, *J* = 281.3 Hz), 111.9 (t, *J* = 250.9 Hz), 63.2, 13.9. ^**19**^**F NMR** (470 MHz, CDCl_3_) δ −58.4, −104.0. **HRMS** (ESI) *m/z* calculated C_18_H_15_F_5_N_2_O_4_S [M]^+^ 450.0567, found 450.0565.

**Figure:**
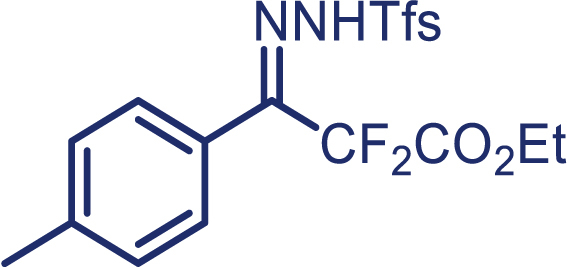

**1e**, White solid; m.p: 83–84°C; ^**1**^**H NMR** (500 MHz, CDCl_3_) δ 8.30–8.26 (m, 1H), 8.13 (s, 1H), 7.91–7.87 (m, 1H), 7.80–7.76 (m, 2H), 7.34 (d, *J* = 8.0 Hz, 2H), 7.16 (d, *J* = 8.0 Hz, 2H), 4.32 (q, *J* = 7.2 Hz, 2H), 2.42 (s, 3H), 1.34 (t, *J* = 7.2 Hz, 3H). ^**13**^**C NMR** (125 MHz, CDCl_3_) δ 162.1 (t, *J* = 30.9 Hz), 147.0 (t, *J* = 32.3 Hz), 142.0, 136.0, 133.9, 133.5, 132.5, 130.5, 128.4 (q, *J* = 6.3 Hz), 128.1, 127.7 (q, *J* = 34.3 Hz), 122.6 (q, *J* = 271.8 Hz), 121.9, 111.9 (t, *J* = 248.8 Hz), 63.2, 21.5, 13.9. ^**19**^**F NMR** (470 MHz, CDCl_3_) δ −58.3, −104.0.

**Figure:**
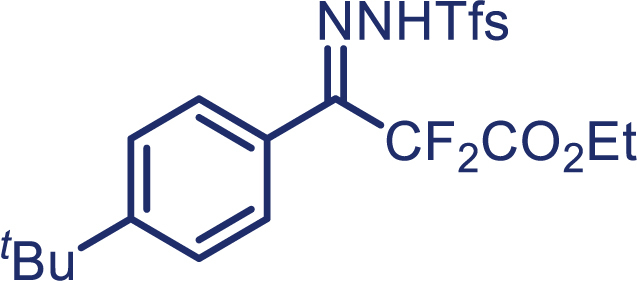

**1f**, White solid; m.p: 82–83°C; ^**1**^**H NMR** (600 MHz, CDCl_3_) δ 8.30–8.24 (m, 1H), 8.17 (s, 1H), 7.91–7.89 (m, 1H), 7.80–7.77 (m, 2H), 7.55–7.54 (m, 2H), 7.20 (d, *J* = 8.3 Hz, 2H), 4.32 (q, *J* = 7.2 Hz, 2H), 1.36 (s, 9H), 1.34 (t, *J* = 7.2 Hz, 3H). ^**13**^**C NMR** (150 MHz, CDCl_3_) δ 162.1 (t, *J* = 30.9 Hz), 155.0, 147.0 (t, *J* = 32.3 Hz), 136.0, 133.9, 133.5, 132.5, 128.4 (q, *J* = 6.1 Hz), 127.9, 127.7 (q, *J* = 32.3 Hz), 126.8, 122.4 (q, *J* = 274.0 Hz), 121.9, 112.0 (t, *J* = 250.5 Hz), 63.2, 35.0, 31.0, 13.9. ^**19**^**F NMR** (564 MHz, CDCl_3_) δ −58.3, −104.0. **HRMS** (ESI) *m/z* calculated C_22_H_23_F_5_N_2_O_4_S [M]^+^ 506.1199, found 506.1191.

**Figure:**
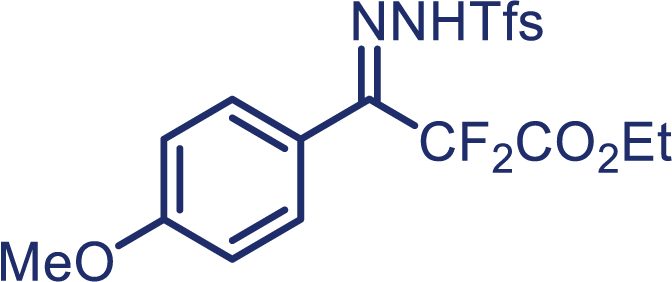

**1g**, White solid; m.p: 92–93°C; ^**1**^**H NMR** (500 MHz, CDCl_3_) δ 8.30–8.26 (m, 1H), 8.15 (s, 1H), 7.90–7.87 (m, 1H), 7.80–7.76 (m, 2H), 7.23 (d, *J* = 8.6 Hz, 2H), 7.03 (d, *J* = 8.6 Hz, 2H), 4.32 (q, *J* = 7.2 Hz, 2H), 3.87 (s, 3H), 1.34 (t, *J* = 7.2 Hz, 3H). ^**13**^**C NMR** (125 MHz, CDCl_3_) δ 162.1 (t, *J* = 31.1 Hz), 161.8, 146.9 (t, *J* = 32.1 Hz), 136.0, 133.9, 133.5, 132.5, 129.9, 128.4 (q, *J* = 6.3 Hz), 127.7 (q, *J* = 33.4 Hz), 122.5 (q, *J* = 270.0 Hz), 116.6, 115.3, 112.1 (t, *J* = 250.4 Hz), 63.2, 55.4, 13.9. ^**19**^**F NMR** (470 MHz, CDCl_3_) δ −58.3, −103.9. **HRMS** (ESI) *m/z* calculated C_19_H_17_F_5_N_2_O_5_S [M]^+^ 480.0670, found 480.0671.

**Figure:**
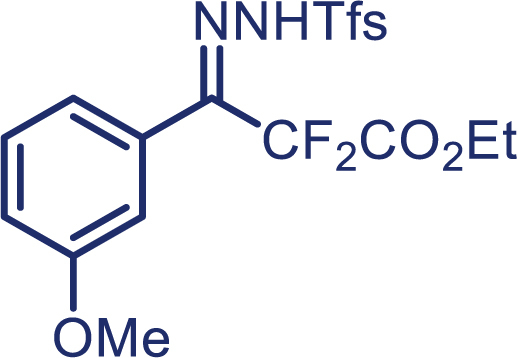

**1h**, White solid; m.p: 96–97°C; ^**1**^**H NMR** (600 MHz, CDCl_3_) δ 8.30–8.27 (m, 1H), 8.17 (s, 1H), 7.92–7.89 (m, 1H), 7.81–7.77 (m, 2H), 7.45 (t, *J* = 7.8 Hz, 1H), 7.09–7.07 (m, 1H), 6.82 (d, *J* = 7.8 Hz, 1H), 6.77–6.75 (m, 1H), 4.33 (q, *J* = 7.2 Hz, 2H), 3.83 (s, 3H), 1.35 (t, *J* = 7.2 Hz, 3H). ^**13**^**C NMR** (150 MHz, CDCl_3_) δ 162.0 (t, *J* = 31.3 Hz), 160.5, 146.5 (t, *J* = 32.4 Hz), 136.0, 133.9, 133.5, 132.5, 131.1, 128.5 (q, *J* = 6.3 Hz), 127.7 (q, *J* = 33.3 Hz), 126.0, 122.5 (q, *J* = 274.6 Hz), 120.1, 117.5, 113.2, 111.9 (t, *J* = 250.6 Hz), 63.2, 55.4, 13.9. ^**19**^**F NMR** (564 MHz, CDCl_3_) δ −58.3, −104.0. **HRMS** (ESI) *m/z* calculated C_19_H_17_F_5_N_2_O_5_S [M]^+^ 480.0670, found 480.0671.

**Figure:**
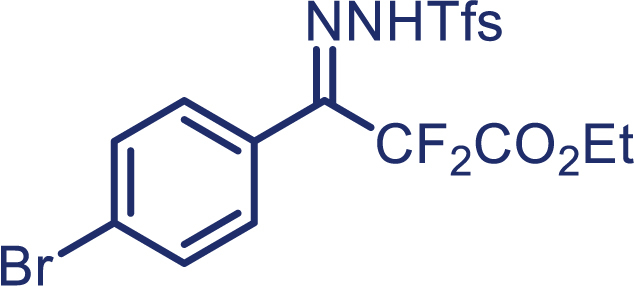

**1i**, White solid; m.p: 116–117°C; ^**1**^**H NMR** (500 MHz, CDCl_3_) δ 8.30–8.26 (m, 1H), 8.08 (s, 1H), 7.91–7.88 (m, 1H), 7.82–7.77 (m, 2H), 7.70 (d, *J* = 8.5 Hz, 2H), 7.17 (d, *J* = 8.5 Hz, 2H), 4.32 (q, *J* = 7.0 Hz, 2H), 1.35 (t, *J* = 7.0 Hz, 3H). ^**13**^**C NMR** (150 MHz, CDCl_3_) δ 161.8 (t, *J* = 30.3 Hz), 145.4 (t, *J* = 32.7 Hz), 135.9, 134.0, 133.5, 133.3, 132.6, 129.9, 128.5 (q, *J* = 6.1 Hz), 127.7 (q, *J* = 30.3 Hz), 126.5, 123.7, 122.5 (q, *J* = 274.2 Hz), 111.7 (t, *J* = 250.8 Hz), 63.3, 13.9. ^**19**^**F NMR** (564 MHz, CDCl_3_) δ −58.3, −103.8. **HRMS** (ESI) *m/z* calculated C_18_H_14_BrF_5_N_2_O_4_S [M]^+^ 527.9684, found 527.9670.

**Figure:**
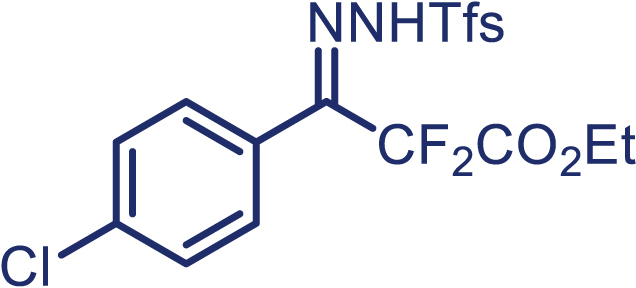

**1j**, White solid; m.p: 96–97°C; ^**1**^**H NMR** (500 MHz, CDCl_3_) δ 8.30–8.26 (m, 1H), 8.07 (s, 1H), 7.91–7.88 (m, 1H), 7.81–7.78 (m, 2H), 7.55–7.53 (m, 2H), 7.24 (d, *J* = 8.5 Hz, 2H), 4.32 (q, *J* = 7.1 Hz, 2H), 1.35 (t, *J* = 7.1 Hz, 3H). ^**13**^**C NMR** (125 MHz, CDCl_3_) δ 161.8 (t, *J* = 31.0 Hz), 145.4 (t, *J* = 32.7 Hz), 138.1, 135.9, 134.0, 133.5, 132.5, 130.3, 129.8, 128.5 (q, *J* = 6.3 Hz), 127.7 (q, *J* = 33.6 Hz), 123.2, 122.4 (d, *J* = 273.5 Hz), 111.8 (t, *J* = 250.8 Hz), 63.3, 13.9. ^**19**^**F NMR** (470 MHz, CDCl_3_) δ −58.3, −103.8. **HRMS** (ESI) *m/z* calculated C_18_H_14_ClF_5_N_2_O_4_S [M]^+^ 484.0180, found 484.0175.

**Figure:**
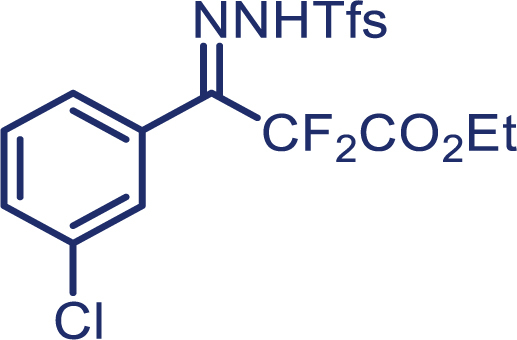

**1k**, White solid; m.p: 127–128°C; ^**1**^**H NMR** (600 MHz, CDCl_3_) δ 8.30–8.26 (m, 1H), 8.11 (s, 1H), 7.92–7.90 (m, 1H), 7.82–7.78 (m, 2H), 7.56–7.54 (m, 1H), 7.50 (t, *J* = 7.8 Hz, 1H), 7.27 (t, *J* = 1.8 Hz, 1H), 7.18–7.16 (m, 1H), 4.33 (q, *J* = 7.2 Hz, 2H), 1.35 (t, *J* = 7.2 Hz, 3H). ^**13**^**C NMR** (150 MHz, CDCl_3_) δ 161.7 (t, *J* = 31.1 Hz), 144.9 (t, *J* = 32.8 Hz), 136.1, 135.8, 134.1, 133.5, 132.6, 131.8, 131.2, 128.5 (q, *J* = 6.6 Hz), 128.4, 127.7 (q, *J* = 33.3 Hz), 126.6, 126.4, 122.5 (q, *J* = 274.0 Hz), 111.7 (t, *J* = 251.0 Hz), 63.4, 13.9. ^**19**^**F NMR** (564 MHz, CDCl_3_) δ −58.3, −103.7. **HRMS** (ESI) *m/z* calculated C_18_H_14_ClF_5_N_2_O_4_S [M]^+^ 484.0171, found 484.0175.

**Figure:**
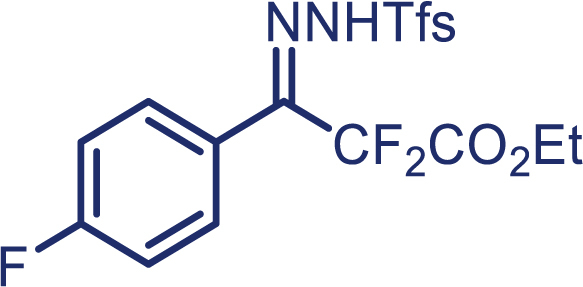

**1l**, White solid; m.p: 87–88°C; ^**1**^**H NMR** (500 MHz, CDCl_3_) δ 8.30–8.27 (m, 1H), 8.09 (s, 1H), 7.92–7.89 (m, 1H), 7.81–7.78 (m, 2H), 7.32–7.23 (m, 4H), 4.32 (q, *J* = 7.1 Hz, 2H), 1.35 (t, *J* = 7.1 Hz, 3H). ^**13**^**C NMR** (125 MHz, CDCl_3_) δ 164.3 (d, *J* = 253.5 Hz), 161.8 (t, *J* = 30.9 Hz), 145.6 (t, *J* = 32.7 Hz), 135.9, 134.0, 133.5, 132.5, 130.7 (d, *J* = 8.6 Hz), 128.5 (q, *J* = 6.2 Hz), 127.7 (q, *J* = 33.0 Hz), 122.4 (q, *J* = 274.2 Hz), 120.8 (d, *J* = 3.5 Hz), 117.3 (d, *J* = 22.0 Hz), 111.8 (t, *J* = 250.5 Hz), 63.3, 13.9. ^**19**^**F NMR** (470 MHz, CDCl_3_) δ −58.3, −103.8, (−106.6)-(-106.7) (m). **HRMS** (ESI) *m/z* calculated C_18_H_14_F_6_N_2_O_4_S [M]^+^ 468.0471, found 468.0471.

**Figure:**
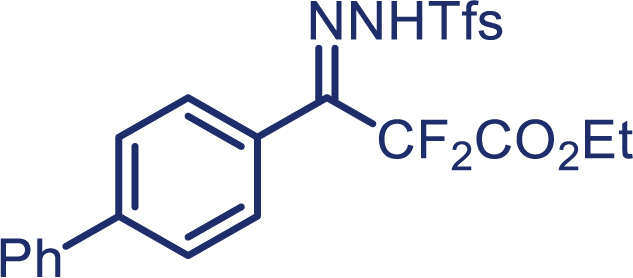

**1m**, White solid; m.p: 97–98°C; ^**1**^**H NMR** (600 MHz, CDCl_3_) δ 8.31–8.29 (m, 1H), 8.21 (s, 1H), 7.92–7.89 (m, 1H), 7.81–7.78 (m, 2H), 7.76–7.73 (m, 2H), 7.62–7.60 (m, 2H), 7.50–7.48 (m, 2H), 7.44–7.41 (m, 1H), 7.36 (d, *J* = 8.2 Hz, 2H), 4.34 (q, *J* = 7.2 Hz, 2H), 1.36 (t, *J* = 7.2 Hz, 3H). ^**13**^**C NMR** (150 MHz, CDCl_3_) δ 162.0 (t, *J* = 31.1 Hz), 146.5 (t, *J* = 32.3 Hz), 144.5, 139.5, 136.0, 133.9, 133.5, 132.5, 129.0, 128.7, 128.5, 128.4 (q, *J* = 6.1 Hz), 128.3, 127.7 (q, *J* = 33.2 Hz), 127.2, 123.5, 122.6 (q, *J* = 272.3 Hz), 112.0 (t, *J* = 250.7 Hz), 63.3, 13.9. ^**19**^**F NMR** (564 MHz, CDCl_3_) δ −58.3, −103.8. **HRMS** (ESI) *m/z* calculated C_24_H_19_F_5_N_2_O_4_S [M]^+^ 526.0886, found 526.0878.

**Figure:**
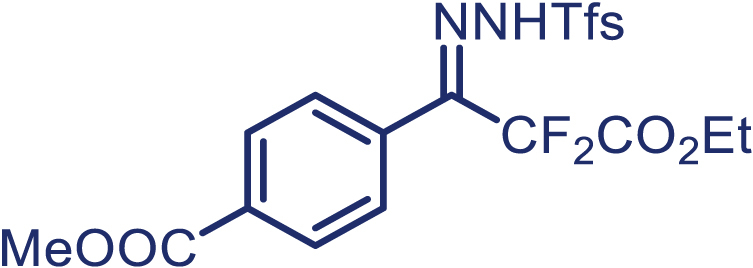

**1n**, White solid; m.p: 111–112°C; ^**1**^**H NMR** (600 MHz, CDCl_3_) δ 8.29–8.24 (m, 2H), 8.19–8.16 (m, 2H), 7.92–7.89 (m, 1H), 7.82–7.78 (m, 2H), 7.39 (d, *J* = 8.1 Hz, 2H), 4.32 (q, *J* = 7.2 Hz, 2H), 3.94 (s, 3H), 1.34 (t, *J* = 7.2 Hz, 3H). ^**13**^**C NMR** (150 MHz, CDCl_3_) δ 165.6, 161.7 (t, *J* = 30.7 Hz), 145.3 (t, *J* = 32.4 Hz), 135.8, 134.0, 133.4, 132.8, 132.5, 130.8, 129.3, 128.5, 128.4 (q, *J* = 6.2 Hz), 127.6 (q, *J* = 33.4 Hz), 122.5 (q, *J* = 274.0 Hz), 111.7 (t, *J* = 251.0 Hz), 63.3, 52.5, 13.8. ^**19**^**F NMR** (564 MHz, CDCl_3_) δ −58.3, −103.6.

**Figure:**
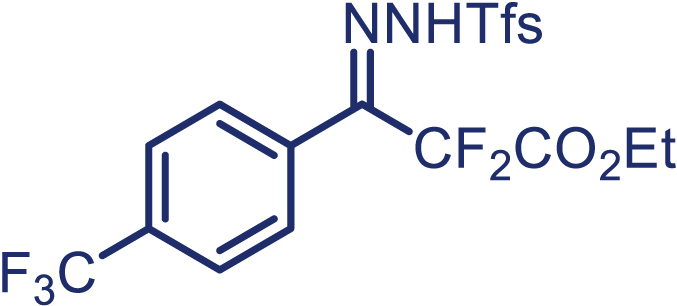

**1o**, White solid; m.p: 95–96°C; ^**1**^**H NMR** (500 MHz, CDCl_3_) δ 8.30–8.28 (m, 1H), 8.06 (s, 1H), 7.93–7.91 (m, 1H), 7.83–7.80 (m, 4H), 7.44 (d, *J* = 8.0 Hz, 2H), 4.33 (q, *J* = 7.2 Hz, 2H), 1.35 (t, *J* = 7.2 Hz, 3H). ^**13**^**C NMR** (125 MHz, CDCl_3_) δ 161.6 (t, *J* = 30.9 Hz), 144.9 (t, *J* = 32.9 Hz), 135.8, 134.1, 133.6 (q, *J* = 32.5 Hz), 133.5, 132.6, 129.1, 128.7, 128.6 (q, *J* = 6.3 Hz), 127.7 (q, *J* = 33.2 Hz), 126.9 (q, *J* = 3.8 Hz), 123.2 (q, *J* = 271.3 Hz), 122.6 (q, *J* = 272.5 Hz), 111.7 (t, *J* = 250.9 Hz), 63.4, 13.9. ^**19**^**F NMR** (470 MHz, CDCl_3_) δ −58.3, −63.3, −103.6. **HRMS** (ESI) *m/z* calculated C_19_H_14_F_8_N_2_O_4_S [M]^+^ 518.0446, found 518.0439.

**Figure:**
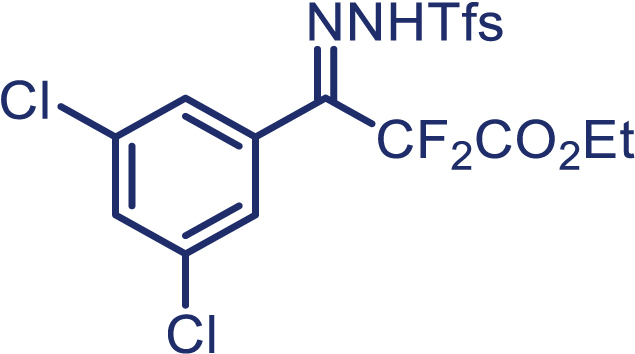

**1p**, ^**1**^**H NMR** (500 MHz, CDCl_3_) δ 8.29–8.27 (m, 1H), 8.12 (s, 1H), 7.94–7.92 (m, 1H), 7.84–7.78 (m, 2H), 7.57 (t, *J* = 1.9 Hz, 1H), 7.17 (d, *J* = 1.9 Hz, 2H), 4.33 (q, *J* = 7.2 Hz, 2H), 1.35 (t, *J* = 7.2 Hz, 3H). ^**13**^**C NMR** (150 MHz, CDCl_3_) δ 161.5 (t, *J* = 31.1 Hz), 143.3 (t, *J* = 32.8 Hz), 137.0, 135.7, 134.2, 133.5, 132.6, 131.9, 128.6 (q, *J* = 5.6 Hz), 127.8 (q, *J* = 33.2 Hz), 127.7, 126.7, 122.5 (q, *J* = 273.5 Hz), 111.5 (t, *J* = 243.0 Hz), 63.5, 13.9. ^**19**^**F NMR** (564 MHz, CDCl_3_) δ −58.3, −103.5.

**Figure:**
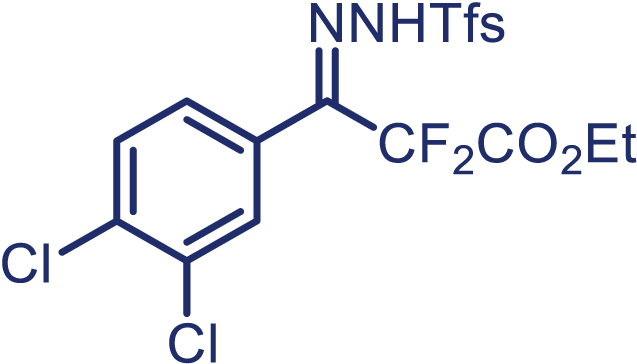

**1q**, White solid; m.p: 96–97°C; ^**1**^**H NMR** (500 MHz, CDCl_3_) δ 8.27–8.25 (m, 1H), 8.21 (s, 1H), 7.93–7.90 (m, 1H), 7.83–7.77 (m, 2H), 7.62 (d, *J* = 8.2 Hz, 1H), 7.40 (d, *J* = 2.0 Hz, 1H), 7.14 (dd, *J* = 8.3, 2.0 Hz, 1H), 4.31 (q, *J* = 7.2 Hz, 2H), 1.33 (t, *J* = 7.2 Hz, 3H). ^**13**^**C NMR** (150 MHz, CDCl_3_) δ 161.6 (t, *J* = 30.8 Hz), 143.7 (t, *J* = 32.8 Hz), 136.4, 135.7, 134.6, 134.1, 133.4, 132.6, 132.0, 130.4, 128.6 (q, *J* = 6.6 Hz), 127.7 (q, *J* = 32.2 Hz), 127.6, 124.6, 122.5 (q, *J* = 274.0 Hz), 111.6 (t, *J* = 251.2 Hz), 63.4, 13.8. ^**19**^**F NMR** (564 MHz, CDCl_3_) δ −58.3, −103.6. **HRMS** (ESI) *m/z* calculated C_18_H_13_Cl_2_F_5_N_2_O_4_S [M]^+^ 517.9799, found 517.9785.

**Figure:**
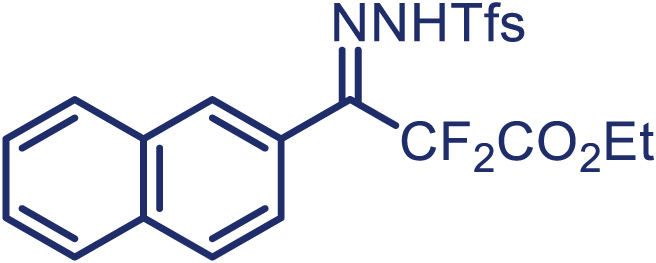

**1r**, White solid; m.p: 108–109°C; ^**1**^**H NMR** (600 MHz, CDCl_3_) δ 8.30–8.28 (m, 1H), 8.24 (s, 1H), 7.99 (d, *J* = 8.4 Hz, 1H), 7.90–7.87 (m, 3H), 7.81 (s, 1H), 7.80–7.76 (m, 2H), 7.62–7.57 (m, 2H), 7.31 (d, *J* = 8.4 Hz, 1H), 4.33 (q, *J* = 7.2 Hz, 2H), 1.35 (t, *J* = 7.2 Hz, 3H). ^**13**^**C NMR** (150 MHz, CDCl_3_) δ 162.0 (t, *J* = 30.9 Hz), 146.7 (t, *J* = 32.4 Hz), 135.9, 134.1, 133.9, 133.4, 132.9, 132.5, 130.0, 129.0, 128.5, 128.4 (q, *J* = 6.5 Hz), 128.3, 127.9, 127.6 (q, *J* = 33.5 Hz), 127.4, 123.9, 122.5 (q, *J* = 272.3 Hz), 122.1, 112.1 (t, *J* = 250.7 Hz), 63.2, 13.8. ^**19**^**F NMR** (564 MHz, CDCl_3_) δ −58.3, −103.5.

**Figure:**
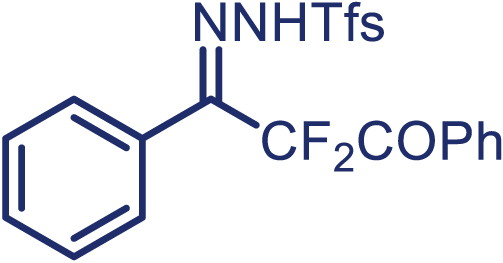

**1s**, White solid; m.p: 125–126°C; ^**1**^**H NMR** (500 MHz, CDCl_3_) δ 8.15 (s, 1H), 7.90–7.86 (m, 3H), 7.80 (d, *J* = 7.8 Hz, 1H), 7.65 (t, *J* = 7.7 Hz, 1H), 7.60–7.56 (m, 4H), 7.48 (t, *J* = 7.7 Hz, 1H), 7.41 (t, *J* = 7.8 Hz, 2H), 7.35–7.34 (m, 2H). ^**13**^**C NMR** (125 MHz, CDCl_3_) δ 187.1 (t, *J* = 27.8 Hz), 148.0 (t, *J* = 31.9 Hz), 135.5, 134.0, 133.71, 133.65, 132.6, 132.4, 131.6, 123.0, 128.5, 128.2, 128.1 (q, *J* = 6.3 Hz), 127.4 (q, *J* = 32.5 Hz), 125.4, 122.5 (q, *J* = 278.8 Hz), 113.9 (t, *J* = 252.0 Hz). ^**19**^**F NMR** (470 MHz, CDCl_3_) δ −58.4, −99.5.

**Figure:**
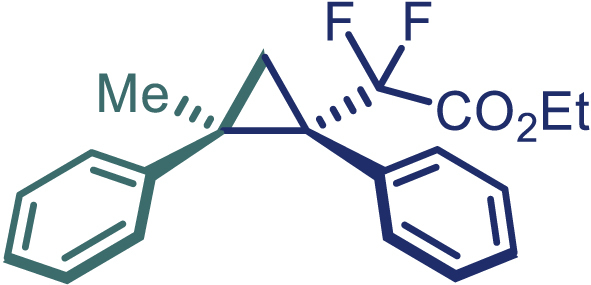

**3**, Colorless oil; ^**1**^**H NMR** (500 MHz, CDCl_3_) δ 7.09–7.02 (m, 6H), 7.01–6.94 (m, 4H), 3.97 (q, *J* = 7.1 Hz, 2H), 2.17–2.14 (m, 1H), 1.87 (d, *J* = 2.1 Hz, 3H), 1.81–1.78 (m, 1H), 1.01 (t, *J* = 7.1 Hz, 3H). ^**13**^**C NMR** (125 MHz, CDCl_3_) δ 163.8 (dd, *J* = 36.1, 34.9 Hz), 142.5, 134.6 (d, *J* = 6.8 Hz), 131.5, 128.0, 127.7, 127.5, 127.3, 126.0, 117.1 (t, *J* = 255.7 Hz), 62.3, 39.5 (dd, *J* = 26, 22.9 Hz), 32.7, 22.0 (d, *J* = 6.7 Hz), 20.7 (t, *J* = 5.0 Hz), 13.6. ^**19**^**F NMR** (470 MHz, CDCl_3_) δ −97.9 (d, *J* = 249.6 Hz), (−103.5)-(-105.0) (m). **IR** (Film): 2963, 1772, 1497, 1450, 1260, 1100 cm^–1^. [α]25 D = −10.3, (c = 1.00, CH_2_Cl_2_).

**Figure:**
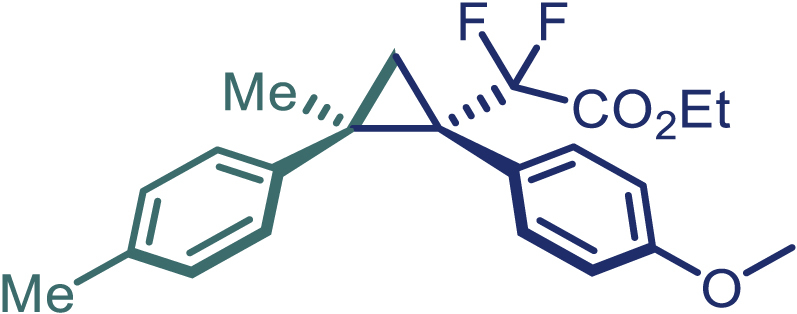

**4**, White solid; mp: 76–77°C; ^**1**^**H NMR** (500 MHz, CDCl_3_) δ 7.01 (d, *J* = 8.8 Hz, 2H), 6.96–6.94 (m, 2H), 6.86 (d, *J* = 7.9 Hz, 2H), 6.55–6.53 (m, 2H), 4.03–3.96 (m, 2H), 3.64 (s, 3H), 2.16 (s, 3H), 2.04–2.02 (m, 1H), 1.82–1.80 (m, 3H), 1.75–1.72 (m, 1H), 1.06 (t, *J* = 7.1 Hz, 3H). ^**13**^**C NMR** (125 MHz, CDCl_3_) δ 163.9 (dd, *J* = 35.4, 34.4 Hz), 158.5, 139.7, 135.3, 132.7, 128.4, 127.8, 126.8 (d, *J* = 6.1 Hz), 117.3 (t, *J* = 256.3 Hz), 112.9, 62.3, 55.0, 38.8 (dd, *J* = 25.8, 22.0 Hz), 32.3, 22.2 (d, *J* = 6.8 Hz), 21.0 (t, *J* = 4.3 Hz), 20.9, 13.7. ^**19**^**F NMR** (470 MHz, CDCl_3_) δ −97.8 (d, *J* = 248.2 Hz), −104.7 (d, *J* = 248.2 Hz). **HRMS** (ESI) *m/z* calculated C_22_H_24_F_2_O_3_ [M]^+^ 374.1587, found 374.1586. **IR** (Film): 2926, 2923, 1773, 1600, 1260, 1109, 1021 cm^–1^. [α]25 D = −12.1, (c = 1.00, CH_2_Cl_2_).

**Figure:**
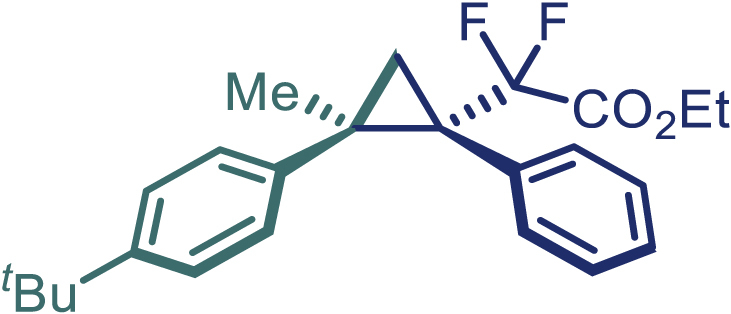

**5**, Colorless oil; ^**1**^**H NMR** (500 MHz, CDCl_3_) δ 7.07–7.02 (m, 4H), 6.98–6.94 (m, 5H), 3.96 (q, *J* = 7.2 Hz, 2H), 2.11 (t, *J* = 5.0 Hz, 1H), 1.87 (s, 3H), 1.77 (d, *J* = 5.9 Hz, 1H), 1.15 (s, 9H), 1.01 (t, *J* = 7.2 Hz, 3H). ^**13**^**C NMR** (125 MHz, CDCl_3_) δ 163.9 (dd, *J* = 35.4, 34.4 Hz), 148.7, 139.3, 134.8 (d, *J* = 6.0 Hz), 131.6, 127.4, 127.1, 124.4, 117.1 (t, *J* = 256.0 Hz), 62.3, 39.6 (dd, *J* = 26.4, 22.4 Hz), 34.1, 32.1, 31.2, 21.8 (d, *J* = 6.6 Hz), 20.9 (t, *J* = 5.0 Hz), 13.6. ^**19**^**F NMR** (470 MHz, CDCl_3_) δ −97.9 (d, *J* = 248.5 Hz), −104.4 (d, *J* = 248.5 Hz). **HRMS** (ESI) *m/z* calculated C_24_H_28_F_2_O_2_ [M]^+^ 386.1948, found 386.1950. **IR** (Film): 2960, 1772, 1500, 1260, 1100 cm^–1^. [α]25 D = −3.9, (c = 1.00, CH_2_Cl_2_).

**Figure:**
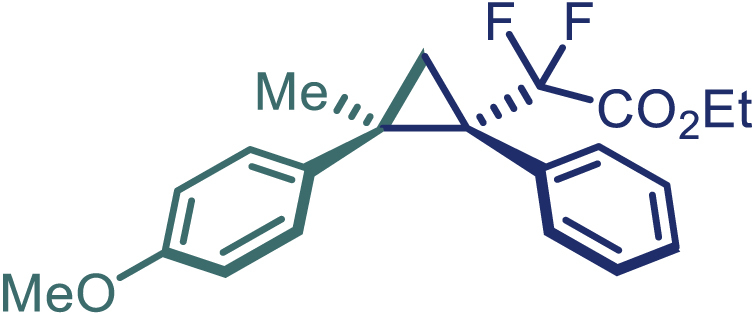

**6**, White solid; mp: 80–81°C; ^**1**^**H NMR** (500 MHz, CDCl_3_) δ 7.09 (d, *J* = 7.2 Hz, 2H), 7.02–6.96 (m, 5H), 6.60–6.56 (m, 2H), 3.95 (q, *J* = 7.1 Hz, 2H), 3.65 (s, 3H), 2.09–2.07 (m, 1H), 1.83 (d, *J* = 1.5 Hz, 3H), 1.78–1.76 (m, 1H), 1.00 (t, *J* = 7.1 Hz, 3H). ^**13**^**C NMR** (125 MHz, CDCl_3_) δ 163.8 (dd, *J* = 35.5, 33.9 Hz), 157.5, 134.8 (d, *J* = 6.0 Hz), 134.7, 131.5, 128.9, 127.6, 127.3, 117.1 (d, *J* = 255.8 Hz), 113.0, 62.3, 55.0, 39.5 (dd, *J* = 26.5, 22.8 Hz), 32.1, 22.3 (d, *J* = 6.6 Hz), 20.9 (t, *J* = 4.9 Hz), 13.5. ^**19**^**F NMR** (470 MHz, CDCl_3_) δ −97.8 (d, *J* = 248.6 Hz), −104.2 (d, *J* = 248.6 Hz). **HRMS** (ESI) *m/z* calculated C_21_H_22_F_2_O_3_ [M]^+^ 360.1419, found 360.1429. **IR** (Film): 3021, 2959,1765, 1613, 1516 cm–1 [α]25 D = −15.5, (c = 1.00, CH_2_Cl_2_).

**Figure:**
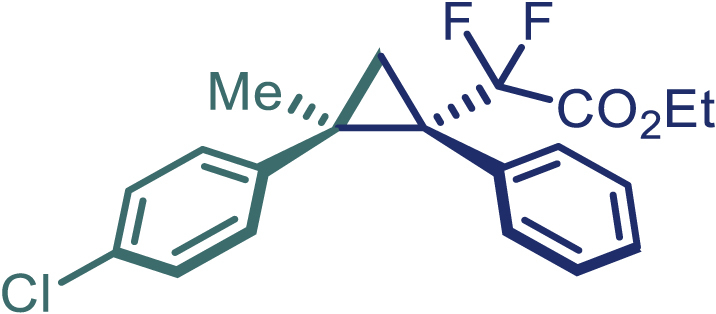

**7**, White solid; mp: 75–76°C; ^**1**^**H NMR** (500 MHz, CDCl_3_) δ 7.09–7.07 (m, 2H), 7.03–6.98 (m, 7H), 3.96 (q, *J* = 7.1 Hz, 2H), 2.10 (t, *J* = 5.4 Hz, 1H), 1.84–1.80 (m, 4H), 1.00 (t, *J* = 7.1 Hz, 3H). ^**13**^**C NMR** (150 MHz, CDCl_3_) δ 163.6 (t, *J* = 34.7 Hz), 141.2, 134.3 (d, *J* = 5.8 Hz), 131.7, 131.2, 129.3, 127.8, 127.7, 127.6, 116.9 (t, *J* = 256.3 Hz), 62.4, 39.6 (dd, *J* = 26.1, 22.1 Hz), 32.1, 22.0 (d, *J* = 6.8 Hz), 20.8 (t, *J* = 5.2 Hz), 13.5. ^**19**^**F NMR** (564 MHz, CDCl_3_) δ −97.9 (d, *J* = 250.7 Hz), −104.4 (d, *J* = 250.7 Hz). **HRMS** (ESI) *m/z* calculated C_20_H_19_ClF_2_O_2_ [M]^+^ 364.0927, found 364.0934. **IR** (Film): 3053, 2989, 2950, 1765, 1493 cm^–1^. [α]25 D = −20.4, (c = 1.00, CH_2_Cl_2_).

**Figure:**
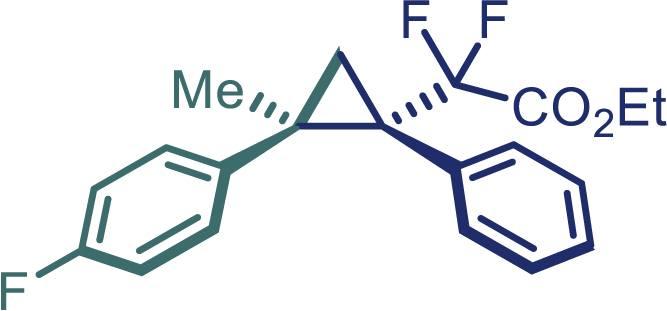

**8**, Colorless oil; ^**1**^**H NMR** (500 MHz, CDCl_3_) δ 7.08–7.01 (m, 7H), 6.73 (t, *J* = 8.7 Hz, 2H), 3.97 (q, *J* = 7.1 Hz, 2H), 2.10 (t, *J* = 5.0 Hz, 1H), 1.84 (s, 3H), 1.80 (d, *J* = 6.1 Hz, 1H), 1.00 (t, *J* = 7.1 Hz, 3H). ^**13**^**C NMR** (150 MHz, CDCl_3_) δ 163.7 (t, *J* = 34.2 Hz), 160.9 (d, *J* = 244.3 Hz), 138.4, 134.5 (d, *J* = 6.5 Hz), 131.3, 129.5 (d, *J* = 8.0 Hz), 127.7, 127.4, 117.0 (t, *J* = 255.8 Hz), 114.5 (d, *J* = 21.0 Hz), 62.4, 39.5 (dd, *J* = 25.7, 22.2 Hz), 32.1, 22.2 (d, *J* = 6.2 Hz), 20.9 (t, *J* = 5.0 Hz), 13.6. ^**19**^**F NMR** (564 MHz, CDCl_3_) δ −97.9 (d, *J* = 249.8 Hz), −104.3 (d, *J* = 251.6 Hz), (−114.5)-(-120.9) (m). **IR** (Film): 2935, 1773, 1560, 1512, 1161 cm^–1^. [α]25 D = −3.1, (c = 1.00, CH_2_Cl_2_).

**Figure:**
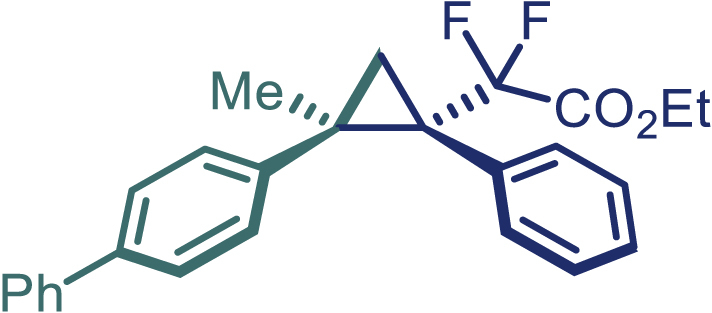

**9**, White solid; mp: 65–66°C; ^**1**^**H NMR** (500 MHz, CDCl_3_) δ 7.46–7.44 (m, 2H), 7.37–7.33 (m, 2H), 7.30–7.26 (m, 3H), 7.13–7.11 (m, 4H), 7.01–6.95 (m, 3H), 3.97 (q, *J* = 7.1 Hz, 2H), 2.18 (t, *J* = 5.5, 1H), 1.90 (d, *J* = 1.4 Hz, 3H), 1.83 (d, *J* = 6.0 Hz, 1H), 1.01 (t, *J* = 7.1 Hz, 3H). ^**13**^**C NMR** (125 MHz, CDCl_3_) δ 163.8 (dd, *J* = 35.4, 33.9 Hz), 141.7, 140.6, 138.5, 134.6 (d, *J* = 6.1 Hz), 131.5, 128.6, 128.3, 127.6, 127.4, 127.0, 126.8, 126.3, 117.1 (t, *J* = 257.1 Hz), 62.3, 39.7 (dd, *J* = 25.9, 22.5 Hz), 32.4, 22.0 (d, *J* = 5.6 Hz), 20.9 (t, *J* = 4.8 Hz), 13.6. ^**19**^**F NMR** (470 MHz, CDCl_3_) δ −97.9 (d, *J* = 249.3 Hz), −104.4 (d, *J* = 249.9 Hz). **HRMS** (ESI) *m/z* calculated C_26_H_24_F_2_O_2_ [M]^+^ 406.1646, found 406.1637. **IR** (Film): 3054, 2985, 2306, 1767, 1421, 1264, 1118, 895, 731, 702 cm^–1^. [α]25 D = −46.1, (c = 1.00, CH_2_Cl_2_).

**Figure:**
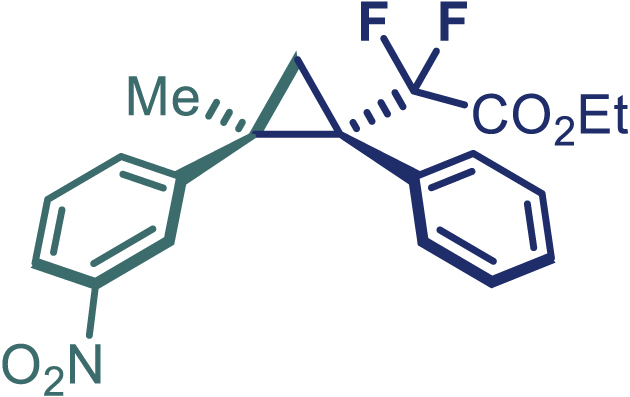

**10**, White solid; mp: 110–111°C; ^**1**^**H NMR** (600 MHz, CDCl_3_) δ 7.90 (t, *J* = 1.9 Hz, 1H), 7.84–7.82 (m, 1H), 7.43 (d, *J* = 8.1 Hz, 1H), 7.22 (t, *J* = 8.0 Hz, 1H), 7.08 (d, *J* = 7.2 Hz, 2H), 7.02–6.97 (m, 3H), 4.00–3.97 (m, 2H), 2.22 (t, *J* = 5.2 Hz, 1H), 1.93–1.89 (m, 4H), 1.01 (t, *J* = 7.1 Hz, 3H). ^**13**^**C NMR** (150 MHz, CDCl_3_) δ 163.4 (t, *J* = 34.1 Hz), 147.7, 144.9, 134.4, 133.7 (d, *J* = 6.3 Hz), 131.1, 128.6, 128.0, 127.8, 122.8, 121.2, 116.7 (t, *J* = 257.4 Hz), 62.5, 39.8 (dd, *J* = 25.8, 22.7 Hz), 32.2, 21.6 (d, *J* = 6.9 Hz), 20.9 (t, *J* = 4.7 Hz), 13.5. ^**19**^**F NMR** (564 MHz, CDCl_3_) δ −98.0 (d, *J* = 251.1 Hz), −104.4 (d, *J* = 251.1 Hz). **IR** (Film): 3055, 2936, 2306, 1770, 6530, 1348, 1265, 1112, 909, 730, 704 cm^–1^. [α]25 D = −19.0, (c = 1.00, CH_2_Cl_2_).

**Figure:**
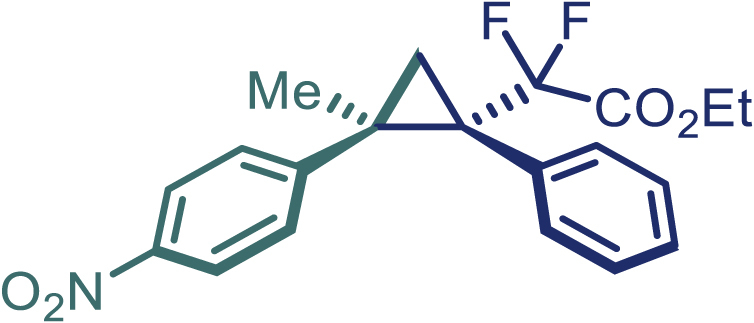

**11**, White solid; mp: 89–90°C; ^**1**^**H NMR** (600 MHz, CDCl_3_) δ 7.91 (d, *J* = 8.9 Hz, 2H), 7.23 (d, *J* = 8.9 Hz, 2H), 7.10–7.06 (m, 2H), 7.04–7.00 (m, 3H), 3.98 (q, *J* = 7.2 Hz, 2H), 2.23–2.21(m, 1H), 1.93–1.89 (m, 4H), 1.01 (t, *J* = 7.2 Hz, 3H). ^**13**^**C NMR** (150 MHz, CDCl_3_) δ 163.4 (t, *J* = 34.0 Hz), 150.3, 146.0, 133.6 (d, *J* = 5.8 Hz), 131.0, 128.8, 128.0, 127.9, 123.0, 116.7 (t, *J* = 256.8 Hz), 62.5, 40.0 (t, *J* = 22.5 Hz), 32.4, 21.5 (d, *J* = 6.7 Hz), 21.1 (t, *J* = 4.7 Hz), 13.5. ^**19**^**F NMR** (564 MHz, CDCl_3_) δ −98.1 (d, *J* = 251.0 Hz), −104.5 (d, *J* = 251.0 Hz). **HRMS** (ESI) *m/z* calculated C_20_H_19_F_2_NO_4_ [M]^+^ 375.1172, found 375.1174. **IR** (Film): 3060, 2990, 2939, 1771, 1599, 1518, 1345 cm^–1^. [α]25 D = −32.2, (c = 1.00, CH_2_Cl_2_).

**Figure:**
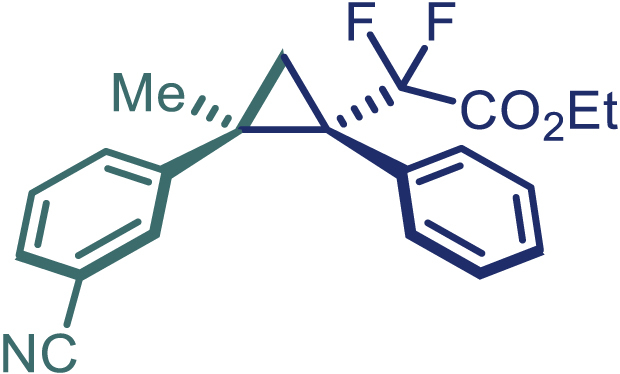

**12**, White solid; mp: 91–92°C; ^**1**^**H NMR** (600 MHz, CDCl_3_) δ 7.34–7.31 (m, 2H), 7.26–7.24 (m, 1H), 7.16 (t, *J* = 8.0 Hz, 1H), 7.09–6.99 (m, 5H), 3.98 (q, *J* = 7.1 Hz, 2H), 2.15 (t, *J* = 5.2 Hz, 1H), 1.88–1.85 (m, 4H), 1.01 (t, *J* = 7.1 Hz, 3H). ^**13**^**C NMR** (150 MHz, CDCl_3_) δ 163.4 (t, *J* = 35.2 Hz), 144.2, 133.8 (d, *J* = 6.1 Hz), 132.7, 131.5, 131.1, 129.8, 128.5, 127.9, 127.8, 118.8, 116.7 (t, *J* = 256.4 Hz), 111.8, 62.5, 39.8 (dd, *J* = 26.4, 22.4 Hz), 32.1, 21.6 (d, *J* = 5.4 Hz), 20.6 (t, *J* = 4.8 Hz), 13.6. ^**19**^**F NMR** (564 MHz, CDCl_3_) δ −98.0 (d, *J* = 251.2 Hz), −104.4 (d, *J* = 251.2 Hz). **HRMS** (ESI) *m/z* calculated C_21_H_19_F_2_NO_2_ [M]^+^ 355.1279, found 355.1276. **IR** (Film): 2940, 2255, 1559, 1502, 1160 cm^–1^. [α]25 D = −21.1, (c = 1.00, CH_2_Cl_2_).

**Figure:**
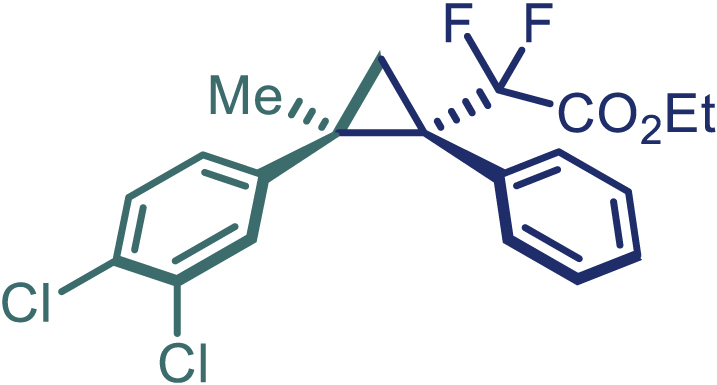

**13**, Colorless oil; ^**1**^**H NMR** (500 MHz, CDCl_3_) δ 7.15–7.05 (m, 7H), 6.89 (d, *J* = 7.1 Hz, 1H), 3.97 (q, *J* = 7.1 Hz, 2H), 2.08 (t, *J* = 5.3 Hz, 1H), 1.86–1.79 (m, 4H), 1.00 (t, *J* = 7.1 Hz, 3H). ^**13**^**C NMR** (150 MHz, CDCl_3_) δ 163.5 (t, *J* = 34.6 Hz), 143.1, 133.9 (d, *J* = 5.9 Hz), 131.7, 130.1, 129.9, 129.6, 128.4 (d, *J* = 4.1 Hz), 127.9, 127.8, 127.5, 116.8 (t, *J* = 256.4 Hz), 62.5, 39.7 (dd, *J* = 25.2, 21.9 Hz), 31.9, 21.8 (d, *J* = 6.9 Hz), 20.9 (t, *J* = 4.8 Hz), 13.6. ^**19**^**F NMR** (564 MHz, CDCl_3_) δ −98.0 (d, *J* = 250.7 Hz), −104.4 (d, *J* = 250.7 Hz). **HRMS** (ESI) *m/z* calculated C_20_H_18_Cl_2_F_2_O_2_ [M]^+^ 398.0539, found 398.0544. **IR** (Film): 3066, 2963, 2937, 1773, 1654, 1102 cm^–1^. [α]25 D = −10.7, (c = 1.00, CH_2_Cl_2_).

**Figure:**
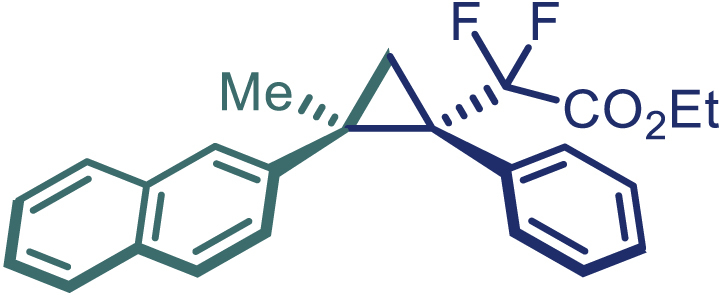

**14**, White solid; mp: 70–71°C; ^**1**^**H NMR** (500 MHz, CDCl_3_) δ 7.66–7.62 (m, 2H), 7.55 (d, *J* = 8.5 Hz, 1H), 7.44 (d, *J* = 1.9 Hz, 1H), 7.37–7.31 (m, 3H), 7.16 (d, *J* = 7.6 Hz, 2H), 6.96–6.86 (m, 3H), 3.97 (q, *J* = 7.2 Hz, 2H), 2.30–2.27 (m, 1H), 1.93 (d, *J* = 2.0 Hz, 3H), 1.89 (d, *J* = 6.0 Hz, 1H), 1.01 (t, *J* = 7.2 Hz, 3H). ^**13**^**C NMR** (150 MHz, CDCl_3_) δ 163.8 (t, *J* = 34.7 Hz), 140.3, 134.4 (d, *J* = 6.7 Hz), 133.0, 131.8, 131.3, 127.62, 127.57, 127.4, 127.34, 127.27, 127.1, 126.0, 125.7, 125.4, 117.2 (t, *J* = 256.0 Hz), 62.4, 39.7 (dd, *J* = 26.0, 21.9 Hz), 33.1, 22.2 (d, *J* = 6.7 Hz), 20.9 (t, *J* = 4.9 Hz), 13.6. ^**19**^**F NMR** (564 MHz, CDCl_3_) δ −97.9 (d, *J* = 249.4 Hz), −104.2 (d, *J* = 249.4 Hz). **HRMS** (ESI) *m/z* calculated C_24_H_22_F_2_O_4_ [M]^+^ 380.1471, found 380.1480. **IR** (Film): 3057, 3020, 2970, 2934, 1768, 1496, 1312,1107 cm^–1^. [α]25 D = +2.3, (c = 1.00, CH_2_Cl_2_).

**Figure:**
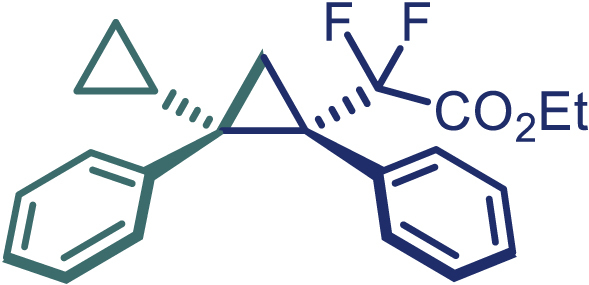

**15**, Colorless oil; ^**1**^**H NMR** (500 MHz, CDCl_3_) δ 7.15–6.90 (m, 10H), 3.98 (q, *J* = 7.2 Hz, 2H), 1.98 (t, *J* = 5.4 Hz, 1H), 1.92–1.85 (m, 1H), 1.79 (d, *J* = 6.2 Hz, 1H), 1.01 (t, *J* = 7.2 Hz, 3H), 0.91–0.81 (m, 1H), 0.77–0.71 (m, 1H), 0.62–0.55 (m, 1H), 0.50–0.46 (m, 1H). ^**13**^**C NMR** (125 MHz, CDCl_3_) δ 164.0 (dd, *J* = 36.1, 33.8 Hz), 141.5, 134.7 (d, *J* = 6.1 Hz), 131.4, 128.7, 127.5, 127.4, 127.3, 125.8, 117.0 (t, *J* = 256.3 Hz), 62.3, 40.3 (t, *J* = 22.5 Hz), 38.7, 18.2 (d, *J* = 4.2 Hz), 15.1 (d, *J* = 3.8 Hz), 13.6, 8.5 (d, *J* = 7.2 Hz), 6.6 (d, *J* = 3.3 Hz). ^**19**^**F NMR** (470 MHz, CDCl_3_) δ −98.8 (d, *J* = 251.4 Hz), −104.7 (d, *J* = 251.4 Hz). **HRMS** (ESI) *m/z* calculated C_22_H_22_F_2_O_2_ [M]^+^ 356.1489, found 356.1480. **IR** (Film): 1740, 1535, 1503, 1152 cm^–1^. [α]25 D = −3.7, (c = 1.00, CH_2_Cl_2_).

**Figure:**
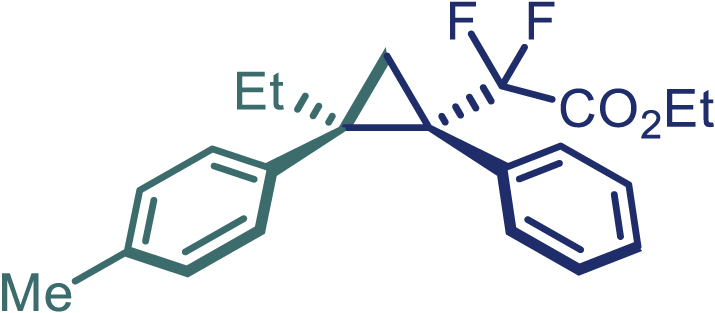

**16**, Colorless oil; ^**1**^**H NMR** (500 MHz, CDCl_3_) δ 7.19–7.06 (m, 1H), 7.01–6.93 (m, 6H), 6.83 (d, *J* = 7.9 Hz, 2H), 3.93 (q, *J* = 7.1 Hz, 2H), 2.37–2.30 (m, 1H), 2.14 (s, 3H), 2.06–1.99 (m, 2H), 1.72 (d, *J* = 5.8 Hz, 1H), 0.98 (t, *J* = 7.1 Hz, 3H), 0.79 (t, *J* = 7.3 Hz, 3H). ^**13**^**C NMR** (150 MHz, CDCl_3_) δ 163.8 (dd, *J* = 35.6, 33.8 Hz), 136.9, 135.4, 134.9 (d, *J* = 6.5 Hz), 131.2, 129.1, 128.3, 127.5, 127.1, 117.4 (t, *J* = 257.7 Hz), 62.3, 40.2 (dd, *J* = 26.4, 21.6 Hz), 39.4, 27.1 (d, *J* = 7.2 Hz), 20.9, 18.9 (t, *J* = 5.2 Hz), 13.5, 11.9. ^**19**^**F NMR** (564 MHz, CDCl_3_) δ −96.2 (d, *J* = 248.4 Hz), (−102.6)-(-103.1) (m). **HRMS** (ESI) *m/z* calculated C_22_H_24_F_2_O_2_ [M]^+^ 358.1632, found 358.1637. **IR** (Film): 1726, 1540, 1513, 1135 cm^–1^. [α]25 D = −2.0, (c = 1.00, CH_2_Cl_2_).

**Figure:**
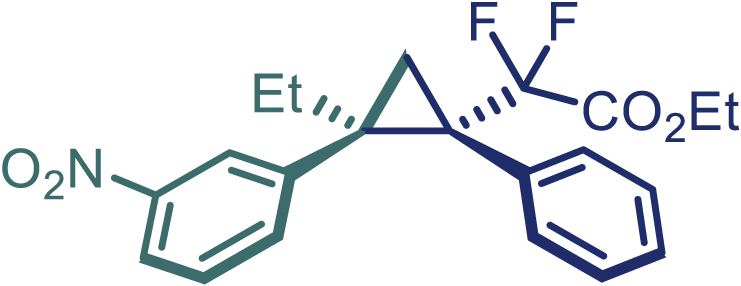

**17**, Colorless oil; ^**1**^**H NMR** (500 MHz, CDCl_3_) δ 7.90 (t, *J* = 2.0 Hz, 1H), 7.84–7.81 (m, 1H), 7.46–7.43 (m, 1H), 7.26 (s, 1H), 7.23 (t, *J* = 8.0 Hz, 1H), 7.03–6.95 (m, 4H), 3.96 (q, *J* = 7.1 Hz, 2H), 2.44–2.36 (m, 1H), 2.19–2.09 (m, 2H), 1.86 (t, *J* = 6.3 Hz, 1H), 0.99 (t, *J* = 7.1 Hz, 3H), 0.82 (t, *J* = 7.3 Hz, 3H). ^**13**^**C NMR** (125 MHz, CDCl_3_) δ 163.4 (dd, *J* = 35.5, 34.1 Hz), 147.6, 142.7, 135.6, 133.9 (d, *J* = 6.1 Hz), 132.3, 128.6, 128.0, 127.7, 124.1, 121.3, 116.9 (t, *J* = 257.2 Hz), 62.5, 40.4 (dd, *J* = 26.1, 22.3 Hz), 39.2, 26.7 (d, *J* = 7.3 Hz), 18.9 (d, *J* = 5.1 Hz), 13.5, 11.9. ^**19**^**F NMR** (470 MHz, CDCl_3_) δ −96.4 (d, *J* = 250.5 Hz), −102.9 (d, *J* = 250.5 Hz). **HRMS** (ESI) *m/z* calculated C_21_H_21_F_2_NO_4_ [M]^+^ 389.1324, found 389.1331. **IR** (Film): 1730, 1610, 1513, 1320, 1140 cm^–1^. [α]25 D = +5.0, (c = 1.00, CH_2_Cl_2_).

**Figure:**
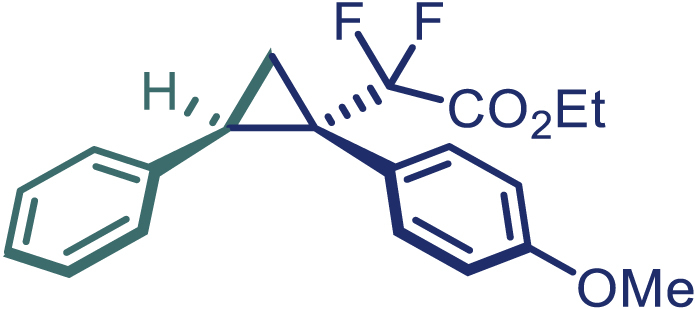

**18**, Colorless oil; ^**1**^**H NMR** (600 MHz, CDCl_3_) δ 7.11–7.05 (m, 3H), 6.94 (d, *J* = 8.4 Hz, 2H), 6.82–6.79 (m, 2H), 6.63 (d, *J* = 8.4 Hz, 2H), 4.16 (q, *J* = 7.1 Hz, 2H), 3.70 (s, 3H), 2.91 (dd, *J* = 9.6, 6.6 Hz, 1H), 1.93 (dd, *J* = 9.0, 5.7 Hz, 1H), 1.61–1.57 (m, 1H), 1.20 (t, *J* = 7.2 Hz, 3H). ^**13**^**C NMR** (150 MHz, CDCl_3_) δ 163.8 (t, *J* = 35.2 Hz), 159.2, 136.6, 133.6, 127.9, 127.7, 126.1, 124.1, 115.2 (t, *J* = 254.1 Hz), 113.4, 62.5, 55.0, 36.2 (t, *J* = 24.6 Hz), 25.1 (t, *J* = 3.4 Hz), 15.1 (t, *J* = 3.7 Hz), 13.9. ^**19**^**F NMR** (564 MHz, CDCl_3_) δ −108.8 (d, *J* = 244.6 Hz), −110.7 (d, *J* = 244.6 Hz). **HRMS** (ESI) *m/z* calculated C_20_H_20_F_2_O_3_ [M]^+^ 346.1268, found 346.1273. **IR** (Film): 3057, 2253, 1766, 1610, 1515, 1265, 906, 721, 649 cm^–1^. [α]25 D = +0.9, (c = 1.00, CH_2_Cl_2_).

**Figure:**
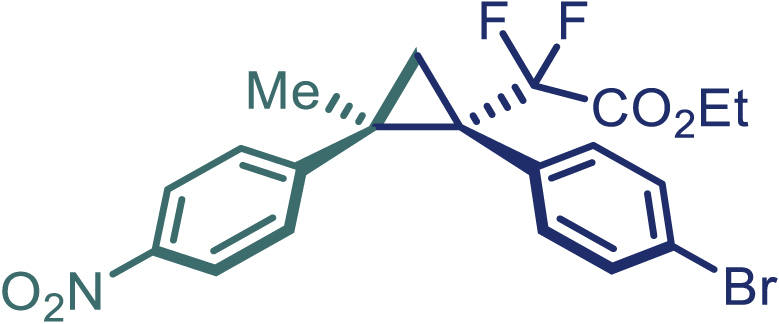

**19**, Colorless oil; ^**1**^**H NMR** (500 MHz, CDCl_3_) δ 7.95 (d, *J* = 8.5 Hz, 2H), 7.23 (d, *J* = 8.5 Hz, 2H), 7.17 (d, *J* = 8.3 Hz, 2H), 6.97 (d, *J* = 8.2 Hz, 2H), 4.07–3.99 (m, 2H), 2.16 (t, *J* = 5.5 Hz, 1H), 1.92 (d, *J* = 6.4 Hz, 1H), 1.87 (s, 3H), 1.07 (t, *J* = 7.1 Hz, 3H). ^**13**^**C NMR** (125 MHz, CDCl_3_) δ 163.2 (dd, *J* = 37.5, 34.5 Hz), 149.8, 146.2, 132.9 (d, *J* = 6.0 Hz), 132.7, 131.2, 128.8, 123.2, 122.3, 116.3 (t, *J* = 257.0 Hz), 62.8, 39.4 (dd, *J* = 25.8, 22.8 Hz), 32.6, 21.6 (d, *J* = 6.7 Hz), 21.1 (t, *J* = 4.9 Hz), 13.6. ^**19**^**F NMR** (470 MHz, CDCl_3_) δ −98.2 (d, *J* = 253.1 Hz), −104.3 (d, *J* = 253.1 Hz). **HRMS** (ESI) *m/z* calculated C_20_H_18_BrF_2_NO_4_ [M]^+^ 453.0289, found 453.0279. **IR** (Film): 3078, 2926, 1766, 1516, 1346, 1104 cm^–1^. [α]25 D = −40.8, (c = 1.00, CH_2_Cl_2_).

**Figure:**
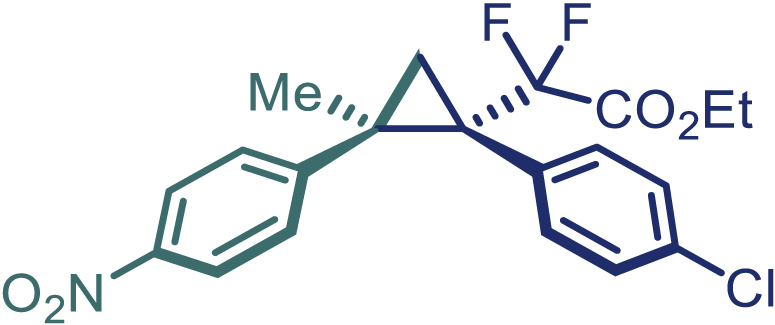

**20**, White solid; mp: 93–94°C; ^**1**^**H NMR** (500 MHz, CDCl_3_) δ 7.95 (d, *J* = 8.8 Hz, 2H), 7.23 (d, *J* = 8.8 Hz, 2H), 7.05–7.00 (m, 4H), 4.08–3.98 (m, 2H), 2.16 (t, *J* = 5.2 Hz, 1H), 1.92 (d, *J* = 6.4 Hz, 1H), 1.88 (s, 3H), 1.07 (t, *J* = 7.1 Hz, 3H). ^**13**^**C NMR** (150 MHz, CDCl_3_) δ 163.2 (dd, *J* = 35.1, 33.0 Hz), 149.9, 146.2, 134.0, 132.5, 132.3 (d, *J* = 5.9 Hz), 128.8, 128.3, 123.2, 116.3 (t, *J* = 257.0 Hz), 62.7, 39.3 (dd, *J* = 25.4, 8.3 Hz), 32.6, 21.6 (d, *J* = 6.9 Hz), 21.1 (t, *J* = 4.5 Hz), 13.6. ^**19**^**F NMR** (564 MHz, CDCl_3_) δ −98.2 (d, *J* = 252.7 Hz), −104.3 (d, *J* = 252.7 Hz). **HRMS** (ESI) *m/z* calculated C_20_H_18_ClF_2_NO_4_ [M]^+^ 409.0787, found 409.0785. **IR** (Film): 3078, 2986, 2940, 1769, 1757, 1517, 1370, 1126 cm^–1^. [α]25 D = −20.3, (c = 1.00, CH_2_Cl_2_).

**Figure:**
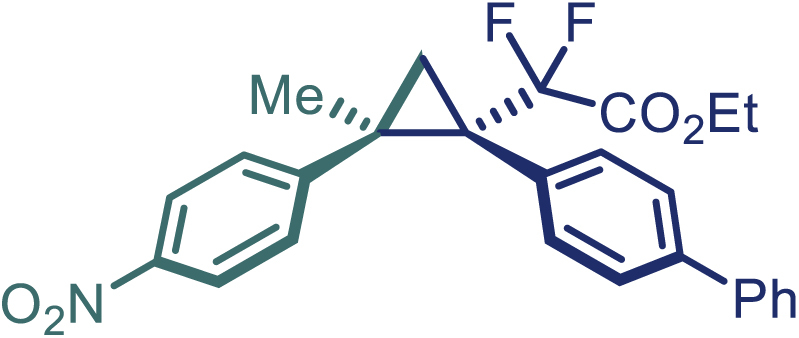

**21**, Colorless oil; ^**1**^**H NMR** (500 MHz, CDCl_3_) δ 7.93 (d, *J* = 8.9 Hz, 2H), 7.44–7.40 (m, 2H), 7.36 (t, *J* = 7.6 Hz, 2H), 7.31–7.26 (m, 5H), 7.15 (d, *J* = 8.1 Hz, 2H), 4.04–3.97 (m, 2H), 2.24 (t, *J* = 5.5 Hz, 1H), 1.96–1.90 (m, 4H), 1.01 (t, *J* = 7.1 Hz, 3H). ^**13**^**C NMR** (150 MHz, CDCl_3_) δ 163.4 (t, *J* = 34.4 Hz), 150.3, 146.1, 140.5, 139.9, 132.7 (d, *J* = 5.8 Hz), 131.6, 128.9, 128.7, 127.5, 126.9, 126.5, 123.1, 116.7 (t, *J* = 257.1 Hz), 62.6, 39.7 (dd, *J* = 25.5, 24.1 Hz), 32.6, 21.7 (d, *J* = 6.7 Hz), 21.2 (t, *J* = 5.0 Hz), 13.6 (d, *J* = 8.1 Hz). ^**19**^**F NMR** (564 MHz, CDCl_3_) δ −102.7 (d, *J* = 251.2 Hz), −109.2 (d, *J* = 251.2 Hz). **HRMS** (ESI) *m/z* calculated C_26_H_23_F_2_NO_4_ [M]^+^ 451.1485, found 451.1487. **IR** (Film): 3031, 2931, 1771, 1518, 1345, 1111 cm^–1^. [α]25 D = −10.9, (c = 1.00, CH_2_Cl_2_).

**Figure:**
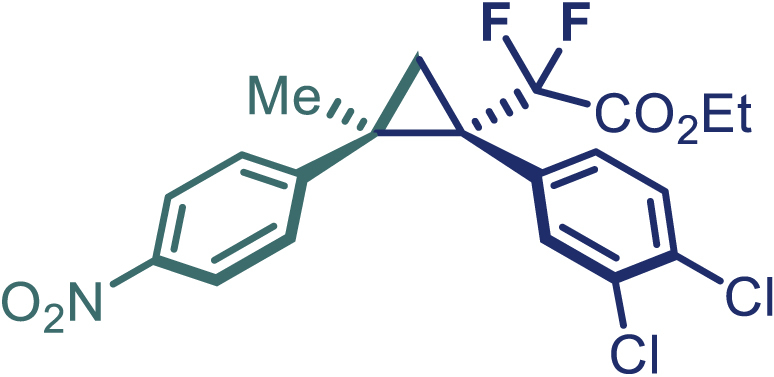

**22**, White solid; mp: 98–99°C; ^**1**^**H NMR** (500 MHz, CDCl_3_) δ 7.99 (d, *J* = 8.8 Hz, 2H), 7.26 (d, *J* = 8.6 Hz, 2H), 7.20 (s, 1H), 7.11 (d, *J* = 8.4 Hz, 1H), 6.95 (d, *J* = 8.4 Hz, 1H), 4.09 (q, *J* = 7.1 Hz, 2H), 2.15 (t, *J* = 5.5 Hz, 1H), 1.94 (d, *J* = 6.5 Hz, 1H), 1.87 (s, 3H), 1.11 (t, *J* = 7.1 Hz, 3H). ^**13**^**C NMR** (150 MHz, CDCl_3_) δ 163.0 (t, *J* = 34.1 Hz), 149.4, 146.4, 134.2 (d, *J* = 5.8 Hz), 133.0, 132.4, 132.2, 130.6, 130.0, 128.8, 123.4, 116.0 (t, *J* = 256.7 Hz), 62.9, 39.0 (dd, *J* = 26.1, 24.3 Hz), 32.8, 21.6 (d, *J* = 6.8 Hz), 21.1 (t, *J* = 4.7 Hz), 13.6. ^**19**^**F NMR** (564 MHz, CDCl_3_) δ −98.2 (d, *J* = 253.8 Hz), −103.9 (d, *J* = 253.8 Hz). **HRMS** (ESI) *m/z* calculated C_20_H_17_Cl_2_F_2_NO_4_ [M]^+^ 443.0385, found 443.0395. **IR** (Film): 3078, 2984, 2935, 1769, 1598, 1516 1370, 1110 cm^–1^. [α]25 D = −77.7, (c = 1.00, CH_2_Cl_2_).

**Figure:**
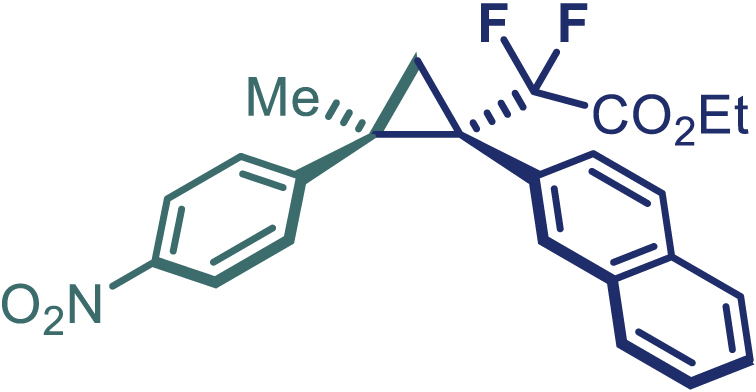

**23**, Colorless oil; ^**1**^**H NMR** (600 MHz, CDCl_3_) δ 7.85 (d, *J* = 8.9 Hz, 2H), 7.64–7.60 (m, 2H), 7.56 (s, 1H), 7.49 (d, *J* = 8.4 Hz, 1H), 7.40–7.36 (m, 2H), 7.29 (d, *J* = 8.8 Hz, 2H), 7.20 (d, *J* = 8.0 Hz, 1H), 3.89 (q, *J* = 7.1 Hz, 2H), 2.35 (t, *J* = 5.0 Hz, 1H), 2.00 (d, *J* = 6.3 Hz, 1H), 1.94 (s, 3H), 0.86 (t, *J* = 7.1 Hz, 3H). ^**13**^**C NMR** (150 MHz, CDCl_3_) δ 163.4 (dd, *J* = 35.0, 33.5 Hz), 150.1, 146.0, 132.6, 132.5, 131.3, 128.8, 128.4, 127.7, 127.6, 127.5, 126.5, 126.2, 123.0, 116.8 (t, *J* = 257.1 Hz), 62.5, 40.1 (dd, *J* = 25.5, 22.1 Hz), 32.6, 21.7 (d, *J* = 6.5 Hz), 21.4, 13.5. ^**19**^**F NMR** (564 MHz, CDCl_3_) δ −98.0 (d, *J* = 251.4 Hz), −104.2 (d, *J* = 251.4 Hz). **HRMS** (ESI) *m/z* calculated C_24_H_21_F_2_NO_4_ [M]^+^ 425.1322, found 425.1331. **IR** (Film): 3057, 2935, 1770, 1518, 1345, 1114, 749 cm^–1^. [α]25 D = −36.9, (c = 1.00, CH_2_Cl_2_).

**Figure:**
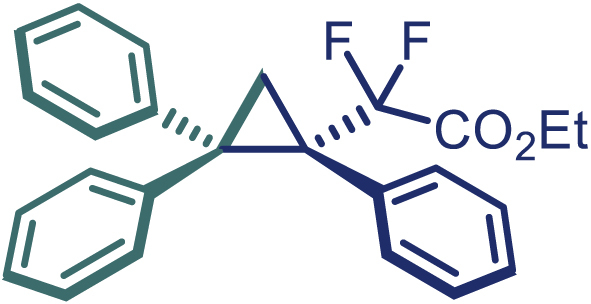

**24**, White solid; mp: 87–88°C; ^**1**^**H NMR** (500 MHz, CDCl_3_) δ 7.69 (d, *J* = 8.0 Hz, 2H), 7.36–7.29 (m, 4H), 7.24–7.19 (m, 1H), 7.15–7.06 (m, 5H), 6.98–6.94 (m, 2H), 6.90–6.86 (m, 1H), 3.96 (q, *J* = 7.0 Hz, 2H), 2.46–2.43 (m, 1H), 2.41–2.38 (m, 1H), 1.00 (t, *J* = 7.0 H z, 3H). ^**13**^**C NMR** (150 MHz, CDCl_3_) δ 163.6 (dd, *J* = 35.7, 32.3 Hz), 141.6, 140.9, 133.4 (d, *J* = 5.7 Hz), 131.7, 130.2, 129.0, 128.1, 127.8, 127.74, 127.69, 126.7, 126.0, 116.4 (t, *J* = 255.5 Hz), 62.3, 42.7, 40.7 (dd, *J* = 26.4, 21.6 Hz), 20.4 (t, *J* = 3.8 Hz), 13.6. ^**19**^**F NMR** (564 MHz, CDCl_3_) δ −102.8 (d, *J* = 249.5 Hz), −105.1 (d, *J* = 249.5 Hz). **HRMS** (ESI) *m/z* calculated C_25_H_22_F_2_O_2_ [M]^+^ 392.1485, found 392.1480. **IR** (Film): 3057, 2253, 1766, 1494, 1304, 1261, 1136, 906, 724, 649 cm^–1^. [α]25 D = −51.2, (c = 1.00, CH_2_Cl_2_).

**Figure:**
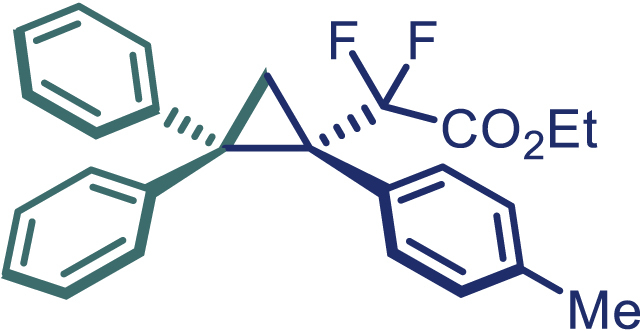

**25**, White solid; mp: 89–90°C; ^**1**^**H NMR** (500 MHz, CDCl_3_) δ 7.68 (d, *J* = 7.5 Hz, 2H), 7.31 (t, *J* = 7.5 Hz, 2H), 7.23–7.17 (m, 3H), 7.15 (d, *J* = 8.0 Hz, 2H), 6.98 (t, *J* = 7.5 Hz, 2H), 6.93–6.87 (m, 3H), 4.00–3.93 (m, 2H), 2.41–2.35 (m, 2H), 2.18 (s, 3H), 1.03 (t, *J* = 7.3 Hz, 3H). ^**13**^**C NMR** (150 MHz, CDCl_3_) δ 163.6 (dd, *J* = 32.9, 3.6 Hz), 141.8, 141.1, 137.4, 131.5, 130.20 (d, *J* = 6.2 Hz), 130.16, 129.0, 128.6, 128.1, 127.7, 126.6, 126.0, 116.5 (t, *J* = 255.2 Hz), 62.3, 42.7, 40.4 (dd, *J* = 26.5, 20.9 Hz), 21.0, 20.6 (dd, *J* = 5.2, 3.6 Hz), 13.6. ^**19**^**F NMR** (564 MHz, CDCl_3_) δ −102.7 (d, *J* = 248.3 Hz), −105.2 (d, *J* = 248.3 Hz). **HRMS** (ESI) *m/z* calculated C_26_H_24_F_2_O_2_ [M]^+^ 406.1642, found 406.1637. **IR** (Film): 3055, 2253, 1765, 1305, 1264, 1088, 906, 726, 649 cm^−1^.

**Figure:**
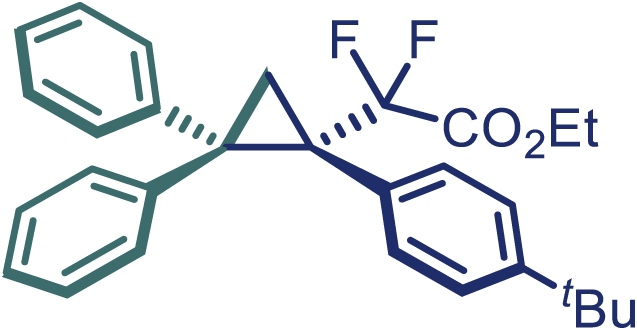

**26**, White solid; mp: 80–81°C; ^**1**^**H NMR** (600 MHz, CDCl_3_) δ 7.68 (d, *J* = 7.8 Hz, 2H), 7.32 (t, *J* = 7.5 Hz, 2H), 7.23–7.19 (m, 3H), 7.13–7.10 (m, 4H), 6.95 (t, *J* = 7.8 Hz, 2H), 6.87 (t, *J* = 7.5 Hz, 1H), 4.00–3.90 (m, 2H), 2.41–2.37 (m, 2H), 1.18 (s, 9H), 0.95 (t, *J* = 7.2 Hz, 3H). ^**13**^**C NMR** (150 MHz, CDCl_3_) δ 163.7 (dd, *J* = 36.2, 32.7 Hz), 150.6, 141.7, 141.1, 131.3, 130.22, 130.16 (d, *J* = 9.8 Hz), 129.0, 128.1, 127.6, 126.6, 125.8, 124.7, 116.5 (t, *J* = 255.4 Hz), 62.2, 42.6, 40.4 (dd, *J* = 26.4, 20.8 Hz), 34.3, 31.1, 20.6 (t, *J* = 4.2 Hz), 13.5. ^**19**^**F NMR** (564 MHz, CDCl_3_) δ −102.6 (d, *J* = 247.8 Hz), −105.6 (d, *J* = 247.8 Hz). **HRMS** (ESI) *m/z* calculated C_29_H_30_F_2_O_2_ [M]^+^ 448.2112, found 448.2106. **IR** (Film): 3057, 2957, 2923, 2859, 2358, 1761, 1459, 1378, 1265, 806, 736, 706 cm^–1^. [α]25 D = −16.9, (c = 1.00, CH_2_Cl_2_).

**Figure:**
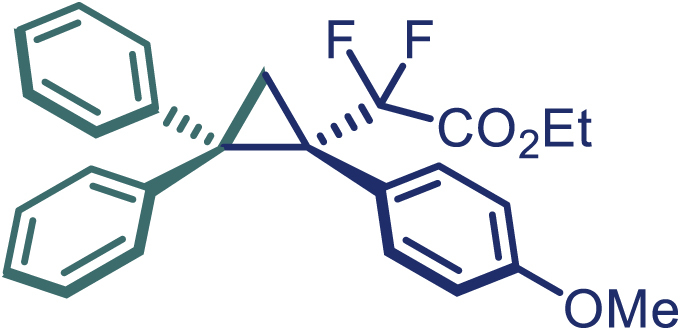

**27**, White solid; mp: 106–107°C; ^**1**^**H NMR** (500 MHz, CDCl_3_) δ 7.67 (d, *J* = 8.0 Hz, 2H), 7.31 (t, *J* = 7.5 Hz, 2H), 7.23–7.19 (m, 3H), 7.15 (d, *J* = 7.5 Hz, 2H), 6.99 (t, *J* = 7.8 Hz, 2H), 6.90 (t, *J* = 7.3 Hz, 1H), 6.65 (d, *J* = 9.0 Hz, 2H), 3.99 (q, *J* = 7.3 Hz, 2H), 3.69 (s, 3H), 2.39–2.35 (m, 2H), 1.06 (t, *J* = 7.3 Hz, 3H). ^**13**^**C NMR** (150 MHz, CDCl_3_) δ 163.6 (dd, *J* = 36.3, 33.0 Hz), 158.9, 141.7, 141.1, 132.8, 130.2, 129.0, 128.1, 127.7, 126.6, 126.0, 125.3 (d, *J* = 4.8 Hz), 116.5 (t, *J* = 256.9 Hz), 113.3, 62.3, 55.0, 42.7, 40.0 (dd, *J* = 26.4, 20.9 Hz), 20.7, 13.7. ^**19**^**F NMR** (470 MHz, CDCl_3_) δ −102.6 (d, *J* = 249.0 Hz), −105.4 (d, *J* = 249.0 Hz). **HRMS** (ESI) *m/z* calculated C_26_H_24_F_2_O_3_ [M]^+^ 422.1590, found 422.1586. **IR** (Film): 3055, 2253, 1766, 1514, 1294, 1261, 905, 725, 649 cm^–1^. [α]25 D = −32.5, (c = 1.00, CH_2_Cl_2_).

**Figure:**
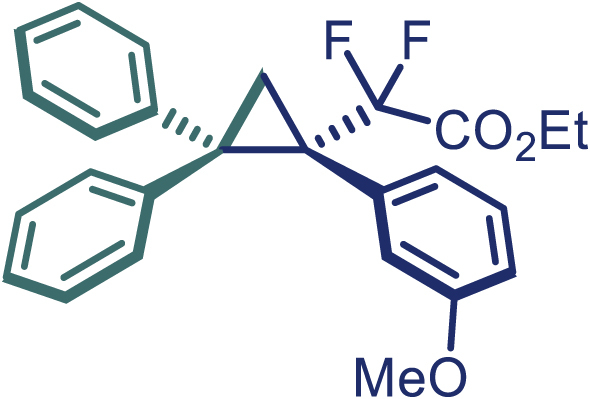

**28**, White solid; mp: 87–88°C; ^**1**^**H NMR** (500 MHz, CDCl_3_) δ 7.68 (d, *J* = 7.7 Hz, 2H), 7.32 (t, *J* = 7.7 Hz, 2H), 7.22 (t, *J* = 7.4 Hz, 1H), 7.16 (d, *J* = 7.6 Hz, 2H), 7.04 (t, *J* = 7.9 Hz, 1H), 6.98 (t, *J* = 7.7 Hz, 2H), 6.94–6.89 (m, 2H), 6.85 (s, 1H), 6.63 (dd, *J* = 8.0, 2.4 Hz, 1H), 4.02–3.97 (m, 2H), 3.68 (s, 3H), 2.44–2.41 (m, 1H), 2.39–2.36 (m, 1H), 1.04 (t, *J* = 7.1 Hz, 3H). ^**13**^**C NMR** (125 MHz, CDCl_3_) δ 163.5 (dd, *J* = 35.9, 32.2 Hz), 159.0, 141.6, 140.9, 134.9 (d, *J* = 5.5 Hz), 130.2, 128.9, 128.7, 128.1, 127.7, 126.7, 126.1, 124.1, 117.7, 116.3 (t, *J* = 257.0 Hz), 113.2, 62.3, 55.1, 42.7, 40.8 (dd, *J* = 26.0, 21.1 Hz), 20.6 (t, *J* = 4.5 Hz), 13.6. ^**19**^**F NMR** (470 MHz, CDCl_3_) δ −102.6 (d, *J* = 249.6 Hz), −104.8 (d, *J* = 249.6 Hz). **HRMS** (ESI) *m/z* calculated C_26_H_24_F_2_O_3_ [M]^+^ 422.1590, found 422.1587. **IR** (Film): 3055, 2923, 2841, 1753, 1580, 1493 cm^–1^. [α]25 D = −54.9, (c = 1.00, CH_2_Cl_2_).

**Figure:**
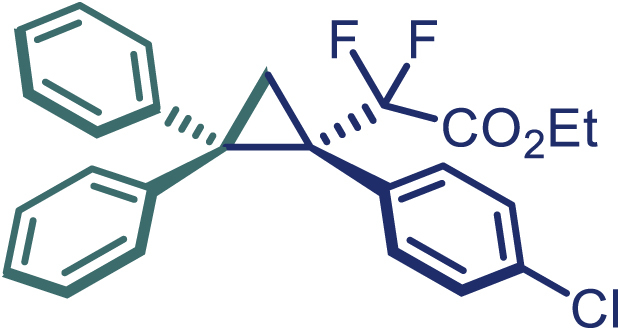

**29**, Colorless oil; ^**1**^**H NMR** (500 MHz, CDCl_3_) δ 7.66 (d, *J* = 7.5 Hz, 2H), 7.34–7.24 (m, 4H), 7.24–7.20 (m, 1H), 7.15–7.09 (m, 4H), 7.00 (t, *J* = 7.5 Hz, 2H), 6.94–6.90 (m, 1H), 4.01–3.96 (m, 2H), 2.43–2.38 (m, 2H), 1.05 (t, *J* = 7.3 Hz, 3H). ^**13**^**C NMR** (125 MHz, CDCl_3_) δ 163.4 (dd, *J* = 36.3, 32.5 Hz), 141.3, 140.5, 133.8, 133.0, 132.2 (d, *J* = 5.0 Hz), 130.1, 128.9, 128.2, 128.1, 127.9, 126.8, 126.3, 116.1 (t, *J* = 255.6 Hz), 62.5, 42.9, 40.1 (dd, *J* = 26.3, 21.9 Hz), 20.5 (t, *J* = 5.0 Hz), 13.6. ^**19**^**F NMR** (470 MHz, CDCl_3_) δ −102.7 (d, *J* = 250.5 Hz), −104.7 (d, *J* = 250.5 Hz). **HRMS** (ESI) *m/z* calculated C_25_H_21_ClF_2_O_2_ [M]^+^ 426.1086, found 426.1090. **IR** (Film): 3057, 2983, 2253, 1381, 1263, 908, 723, 649 cm^–1^. [α]25 D = −33.5, (c = 1.00, CH_2_Cl_2_).

**Figure:**
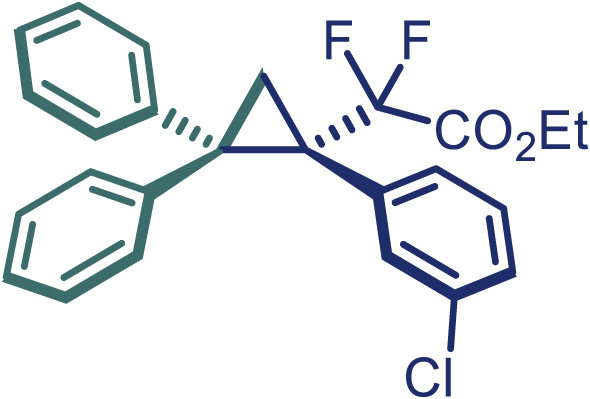

**30**, Colorless oil; ^**1**^**H NMR** (500 MHz, CDCl_3_) δ 7.67 (d, *J* = 8.0 Hz, 2H), 7.34–7.30 (m, 3H), 7.25–7.20 (m, 2H), 7.16–7.14 (m, 2H), 7.09–7.04 (m, 2H), 7.04–6.99 (m, 2H), 6.94–6.90 (m, 1H), 4.00 (q, *J* = 7.0 Hz, 2H), 2.44–2.39 (m, 2H), 1.05 (t, *J* = 7.0 Hz, 3H). ^**13**^**C NMR** (125 MHz, CDCl_3_) δ 163.3 (dd, *J* = 35.6, 32.8 Hz), 141.2, 140.4, 135.7 (d, *J* = 5.5 Hz), 133.6, 131.7, 130.1, 129.1, 128.9, 128.2, 128.0, 127.9, 126.8, 126.3, 116.0 (t, *J* = 255.3 Hz), 62.5, 42.9, 40.4 (dd, *J* = 26.2, 21.8 Hz), 20.4 (t, *J* = 4.2 Hz), 13.6. ^**19**^**F NMR** (564 MHz, CDCl_3_) δ −102.9 (d, *J* = 251.0 Hz), −104.7 (d, *J* = 251.0 Hz). **HRMS** (ESI) *m/z* calculated C_25_H_21_ClF_2_O_2_ [M]^+^ 426.1090, found 426.1090. **IR** (Film): 3055, 2253, 1767, 1449, 1304, 1265, 1137, 906, 726, 649 cm^–1^. [α]25 D = −48.3, (c = 1.00, CH_2_Cl_2_).

**Figure:**
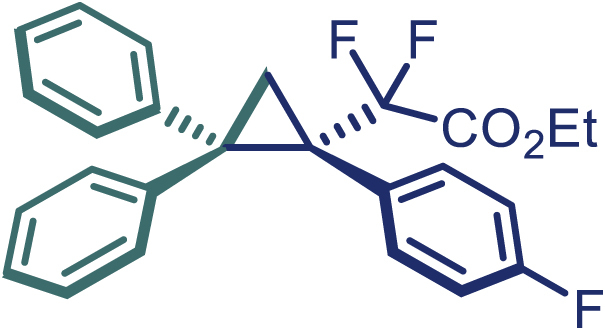

**31**, White solid; mp: 113–114°C; ^**1**^**H NMR** (500 MHz, CDCl_3_) δ 7.67 (d, *J* = 8.0 Hz, 2H), 7.34–7.29 (m, 4H), 7.22 (t, *J* = 7.5 Hz, 1H), 7.13 (d, *J* = 7.5 Hz, 2H), 6.99 (t, *J* = 7.5 Hz, 2H), 6.91 (t, *J* = 7.5 Hz, 1H), 6.81 (t, *J* = 7.5 Hz, 2H), 3.99 (q, *J* = 7.2 Hz, 2H), 2.43–2.38 (m, 2H), 1.05 (t, *J* = 7.2 Hz, 3H). ^**13**^**C NMR** (125 MHz, CDCl_3_) δ 163.5 (dd, *J* = 35.0, 32.5 Hz), 162.1 (d, *J* = 248.5 Hz), 141.4, 140.7, 133.4, 130.2, 129.4 (dd, *J* = 5.5, 3.4 Hz),128.9, 128.1, 127.9, 126.8, 126.2, 116.2 (t, *J* = 255.0 Hz), 114.8 (d, *J* = 22.5 Hz), 62.4, 42.8, 40.0 (dd, *J* = 27.5, 21.9 Hz), 20.6 (dd, *J* = 5.0, 3.8 Hz), 13.7. ^**19**^**F NMR** (470 MHz, CDCl_3_) δ −102.8 (d, *J* = 249.1 Hz), −105.1 (d, *J* = 249.1 Hz), (−113.7)-(-113.8) (m). **HRMS** (ESI) *m/z* calculated C_25_H_21_F_3_O_2_ [M]^+^ 410.1394, found 410.1386. **IR** (Film): 3055, 2358, 2253, 1766, 1512, 1303, 1261, 1135, 905, 725, 649 cm^–1^. [α]25 D = −50.2, (c = 1.00, CH_2_Cl_2_).

**Figure:**
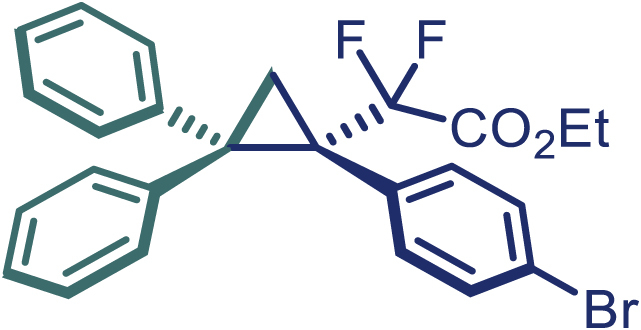

**32**, White solid; mp: 114–115°C; ^**1**^**H NMR** (500 MHz, CDCl_3_) δ 7.66 (d, *J* = 7.5 Hz, 2H), 7.32 (t, *J* = 7.5 Hz, 2H), 7.27–7.19 (m, 5H), 7.13 (d, *J* = 7.5 Hz, 2H), 7.00 (t, *J* = 7.5 Hz, 2H), 6.94–6.90 (m, 1H), 4.03–3.93 (m, 2H), 2.42–2.37 (m, 2H), 1.05 (t, *J* = 7.0 Hz, 3H). ^**13**^**C NMR** (125 MHz, CDCl_3_) δ 163.3 (dd, *J* = 35.6, 32.8 Hz), 141.3, 140.5, 133.3, 132.7 (d, *J* = 5.6 Hz), 131.0, 130.1, 128.9, 128.1, 127.9, 126.8, 126.3, 122.1, 116.0 (t, *J* = 255.4 Hz), 62.5, 42.9, 40.2 (dd, *J* = 26.4, 21.4 Hz), 20.4 (t, *J* = 4.3 Hz), 13.6. ^**19**^**F NMR** (470 MHz, CDCl_3_) δ −102.8 (d, *J* = 251.0 Hz), −104.7 (d, *J* = 251.0 Hz). **HRMS** (ESI) *m/z* calculated C_25_H_21_BrF_2_O_2_ [M]^+^ 470.0587, found 470.0585. **IR** (Film): 2986, 2253, 1766, 1491, 1264, 1136, 906, 726, 649 cm^–1^. [α]25 D = −51.1, (c = 1.00, CH_2_Cl_2_).

**Figure:**
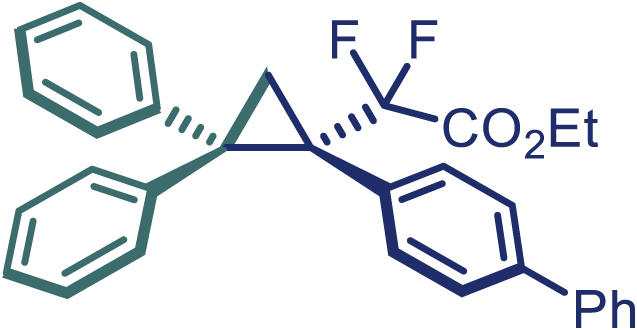

**33**, Colorless oil; ^**1**^**H NMR** (600 MHz, CDCl_3_) δ 7.70 (d, *J* = 8.4 Hz, 2H), 7.50–7.47 (m, 2H), 7.40–7.36 (m, 6H), 7.35–7.28 (m, 3H), 7.24–7.21 (m, 1H), 7.19–7.17 (m, 2H), 6.99–6.96 (m, 2H), 6.90–6.87 (m, 1H), 4.01–3.95 (m, 2H), 2.48–2.46 (m, 1H), 2.44–2.42 (m, 1H), 1.01 (t, *J* = 6.9 Hz, 3H). ^**13**^**C NMR** (150 MHz, CDCl_3_) δ 163.6 (dd, *J* = 35.9, 32.0 Hz), 141.6, 140.9, 140.3, 132.5 (d, *J* = 5.7 Hz), 132.0, 130.2, 129.0, 128.7, 128.1, 127.8, 127.4, 126.9, 126.7, 126.4, 126.1, 116.4 (t, *J* = 255.5 Hz), 62.4, 42.9, 40.5 (dd, *J* = 26.4, 20.9 Hz), 20.6, 13.6. ^**19**^**F NMR** (564 MHz, CDCl_3_) δ −102.6 (d, *J* = 250.0 Hz), −104.9 (d, *J* = 250.0 Hz). **HRMS** (ESI) *m/z* calculated C_31_H_26_F_2_O_2_ [M]^+^ 468.1787, found 468.1793. **IR** (Film): 3057, 2253, 1766, 1424, 1265, 906, 722, 649 cm^–1^. [α]25 D = −25.4, (c = 1.00, CH_2_Cl_2_).

**Figure:**
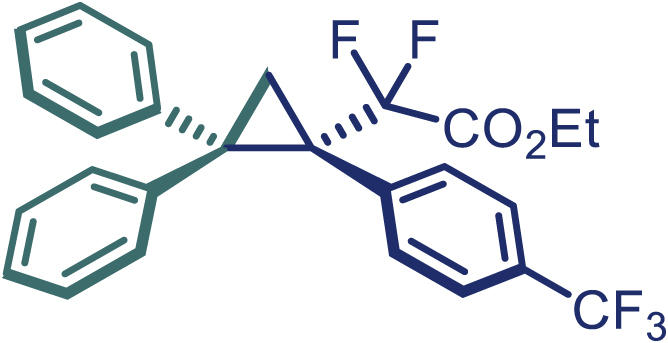

**34**, White solid; mp: 97–98°C; ^**1**^**H NMR** (500 MHz, CDCl_3_) δ 7.68 (d, *J* = 8.0 Hz, 2H), 7.46 (d, *J* = 7.0 Hz, 2H), 7.38 (d, *J* = 8.5 Hz, 2H), 7.33 (t, *J* = 7.5 Hz, 2H), 7.23 (t, *J* = 7.3 Hz, 1H), 7.12 (d, *J* = 8.0 Hz, 2H), 6.98 (t, *J* = 8.0 Hz, 2H), 6.91 (t, *J* = 8.0 Hz, 1H), 4.01–3.90 (m, 2H), 2.49–2.44 (m, 2H), 1.00 (t, *J* = 7.3 Hz, 3H). ^**13**^**C NMR** (125 MHz, CDCl_3_) δ 163.3 (dd, *J* = 35.4, 32.6 Hz), 141.1, 140.3, 137.8 (d, *J* = 5.1 Hz), 132.1, 130.0, 129.9 (q, *J* = 32.5 Hz), 128.8, 128.2, 128.0, 126.9, 126.4, 124.7 (q, *J* = 3.4 Hz), 123.7 (q, *J* = 270.6 Hz), 116.0 (t, *J* = 255.4 Hz), 62.6, 43.0, 40.5 (dd, *J* = 26.4, 21.9 Hz), 20.4 (t, *J* = 4.3 Hz), 13.5. ^**19**^**F NMR** (470 MHz, CDCl_3_) δ −62.8, −102.8 (d, *J* = 251.5 Hz), −104.3 (d, *J* = 251.5 Hz). **IR** (Film): 3055, 2253, 1766, 1449, 1326, 1264, 1129, 907, 726, 649 cm^–1^. [α]25 D = −27.8, (c = 1.00, CH_2_Cl_2_).

**Figure:**
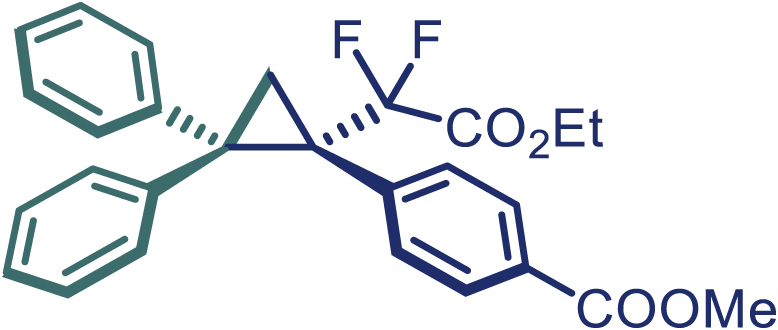

**35**, Colorless oil; ^**1**^**H NMR** (600 MHz, CDCl_3_) δ 7.81–7.78 (m, 2H), 7.68 (d, *J* = 7.8 Hz, 2H), 7.42 (d, *J* = 7.2 Hz, 2H), 7.33 (t, *J* = 7.5 Hz, 2H), 7.24–7.21 (m, 1H), 7.15–7.12 (m, 2H), 6.98–6.95 (m, 2H), 6.90–6.87 (m, 1H), 3.96 (q, *J* = 7.2 Hz, 2H), 3.84 (s, 3H), 2.51–2.48 (m, 1H), 2.45–2.42 (m, 1H), 1.02 (t, *J* = 7.2 Hz, 3H). ^**13**^**C NMR** (150 MHz, CDCl_3_) δ 166.6, 163.3 (dd, *J* = 35.7, 32.2 Hz), 141.2, 140.4, 138.9 (d, *J* = 5.0 Hz), 131.6, 130.0, 129.4, 129.1, 128.9, 128.2, 127.9, 126.8, 126.3, 116.0 (t, *J* = 255.3 Hz), 62.5, 52.1, 43.1, 40.6 (dd, *J* = 26.6, 21.8 Hz), 20.4 (t, *J* = 4.5 Hz), 13.6. ^**19**^**F NMR** (564 MHz, CDCl_3_) δ −102.8 (d, *J* = 251.4 Hz), −104.2 (d, *J* = 251.4 Hz). **HRMS** (ESI) *m/z* calculated C_27_H_24_F_2_O_4_ [M]^+^ 450.1538, found 450.1535. **IR** (Film): 3055, 2985, 1768, 1720, 1264, 1113, 731, 703, 601 cm^–1^. [α]25 D = −33.4, (c = 1.00, CH_2_Cl_2_).

**Figure:**
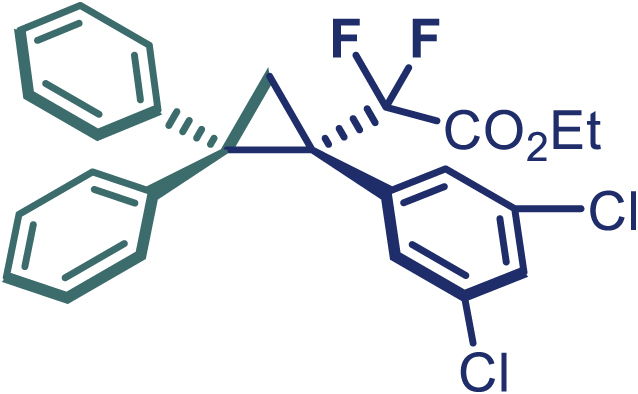

**36**, White solid; mp: 95–96°C; ^**1**^**H NMR** (600 MHz, CDCl_3_) δ 7.66 (d, *J* = 8.4 Hz, 2H), 7.32 (t, *J* = 8.1 Hz, 2H), 7.24–7.21 (m, 3H), 7.17–7.15 (m, 2H), 7.09 (t, *J* = 1.8 Hz, 1H), 7.07–7.03 (m, 2H), 6.98–6.94 (m, 1H), 4.08–4.01 (m, 2H), 2.40 (s, 2H), 1.10 (t, *J* = 7.2 Hz, 3H). ^**13**^**C NMR** (150 MHz, CDCl_3_) δ 163.1 (dd, *J* = 35.9, 32.9 Hz), 140.8, 139.9, 137.3 (d, *J* = 5.3 Hz), 134.3, 130.3, 130.0, 128.7, 128.2, 128.1, 128.0, 127.0, 126.6, 115.7 (t, *J* = 255.5 Hz), 62.7, 43.1, 40.2 (dd, *J* = 26.7, 22.4 Hz), 20.4 (t, *J* = 3.9 Hz), 13.7. ^**19**^**F NMR** (564 MHz, CDCl_3_) δ −102.9 (d, *J* = 253.0 Hz), −104.2 (d, *J* = 253.0 Hz). **HRMS** (ESI) *m/z* calculated C_25_H_20_Cl_2_F_2_O_2_ [M]^+^ 460.0712, found 460.0701. **IR** (Film): 3086, 2984, 2253, 1768, 1264, 905, 726, 649 cm^–1^. [α]25 D = −40.0, (c = 1.00, CH_2_Cl_2_).

**Figure:**
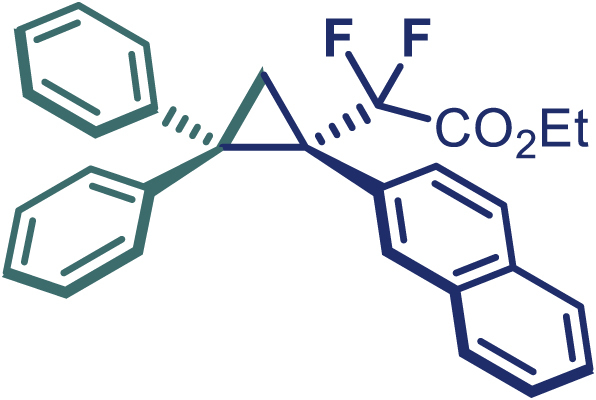

**37**, White solid; mp: 122–123°C; ^**1**^**H NMR** (500 MHz, CDCl_3_) δ 7.77–7.71 (m, 3H), 7.70–7.67 (m, 2H), 7.60 (d, *J* = 8.5 Hz, 1H), 7.54 (s, 1H), 7.40–7.32 (m, 4H), 7.25–7.19 (m, 3H), 6.90 (t, *J* = 7.8 Hz, 2H), 6.79 (t, *J* = 7.3 Hz, 1H), 3.87 (q, *J* = 7.0 Hz, 2H), 2.58 (t, *J* = 4.0 Hz, 1H), 2.49 (d, *J* = 5.5 Hz, 1H), 0.85 (t, *J* = 7.0 Hz, 3H). ^**13**^**C NMR** (125 MHz, CDCl_3_) δ 163.5 (dd, *J* = 35.8, 32.8 Hz), 141.7, 140.8, 132.8, 132.6, 131.2 (d, *J* = 4.8 Hz), 130.9, 130.2, 129.3, 129.0, 128.1, 127.8, 127.7, 127.44, 127.39, 126.7, 126.2, 126.1, 125.9, 116.5 (t, *J* = 255.7 Hz), 62.3, 42.9, 40.9 (dd, *J* = 26.4, 21.3 Hz), 20.8 (t, *J* = 4.3 Hz), 13.5. ^**19**^**F NMR** (470 MHz, CDCl_3_) δ −102.4 (d, *J* = 249.1 Hz), −104.7 (d, *J* = 249.1 Hz). **HRMS** (ESI) *m/z* calculated C_29_H_24_F_2_O_2_ [M]^+^ 442.1641, found 442.1637. **IR** (Film): 3055, 2253, 1766, 1378, 1261, 1009, 907, 725, 649 cm^–1^. [α]25 D = −2.1, (c = 1.00, CH_2_Cl_2_).

**Figure:**
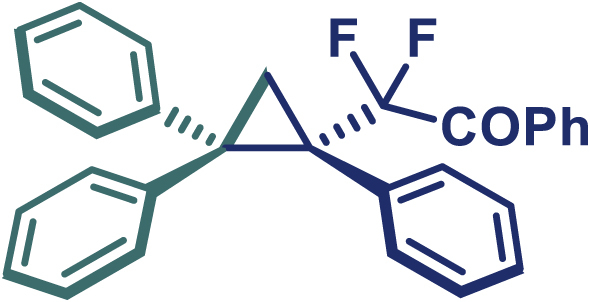

**38**, Colorless oil; ^**1**^**H NMR** (500 MHz, CDCl_3_) δ 7.75 (d, *J* = 7.7 Hz, 2H), 7.63 (d, *J* = 7.8 Hz, 2H), 7.47 (t, *J* = 7.5 Hz, 1H), 7.37–7.28 (m, 5H), 7.24–7.22 (m, 2H), 7.16–7.13 (m, 2H), 7.02–6.94 (m, 5H), 6.90–6.86 (m, 1H), 2.50–2.44 (m, 2H). ^**13**^**C NMR** (150 MHz, CDCl_3_) δ 190.5 (dd, *J* = 33.6, 29.0 Hz), 141.9, 140.9, 133.7, 133.5 (d, *J* = 5.8 Hz), 133.3, 132.2, 130.4, 129.6, 129.0, 128.11, 128.09, 127.69, 127.65, 127.5, 126.6, 126.0, 119.5 (t, *J* = 260.1 Hz), 42.7, 41.1 (dd, *J* = 26.3, 20.9 Hz), 21.1 (d, *J* = 6.1 Hz). ^**19**^**F NMR** (564 MHz, CDCl_3_) δ −96.0 (d, *J* = 264.6 Hz), −99.4 (d, *J* = 264.6 Hz). **HRMS** (ESI) *m/z* calculated C_29_H_22_F_2_O [M]^+^ 424.1535, found 424.1531. **IR** (Film): 3087, 2253, 1710, 1449, 1264, 906, 726, 649 cm^–1^. [α]25 D = −18.9, (c = 1.00, CH_2_Cl_2_).

**Figure:**
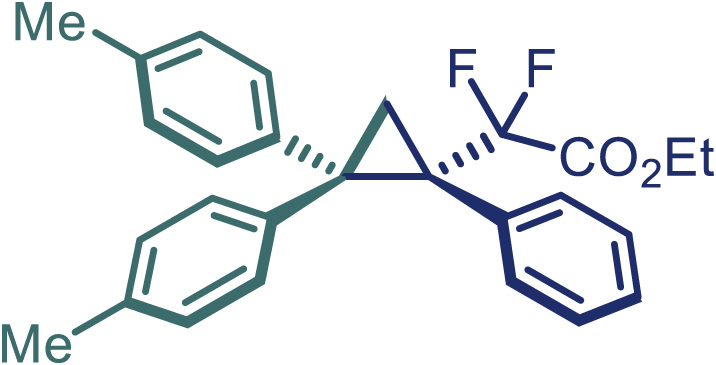

**40**, Colorless oil; ^**1**^**H NMR** (500 MHz, CDCl_3_) δ 7.54 (d, *J* = 7.5 Hz, 2H), 7.39–7.29 (m, 2H), 7.15–7.07 (m, 5H), 7.00 (d, *J* = 8.0 Hz, 2H), 6.76 (d, *J* = 8.0 Hz, 2H), 3.95 (q, *J* = 7.1 Hz, 2H), 2.39–2.34 (m, 2H), 2.30 (s, 3H), 2.07 (s, 3H), 1.00 (t, *J* = 7.1 Hz, 3H). ^**13**^**C NMR** (125 MHz, CDCl_3_) δ 163.6 (dd, *J* = 36.0, 32.8 Hz), 138.9, 138.3, 136.1, 135.4, 133.7 (d, *J* = 5.7 Hz), 131.7, 129.9, 128.81, 128.76, 128.4, 127.8, 127.7, 116.5 (t, *J* = 256.4 Hz), 62.3, 42.1, 40.7 (dd, *J* = 26.8, 20.9 Hz), 21.1, 20.7, 20.5 (t, *J* = 4.5 Hz), 13.6. ^**19**^**F NMR** (470 MHz, CDCl_3_) δ −102.7 (d, *J* = 248.1 Hz), −105.0 (d, *J* = 248.1 Hz). **HRMS** (ESI) *m/z* calculated C_27_H_26_F_2_O_2_ [M]^+^ 420.1787, found 420.1789. **IR** (Film): 3060, 2245, 1705, 1435, 1260 cm^–1^. [α]25 D = −18.9, (c = 1.00, CH_2_Cl_2_).

**Figure:**
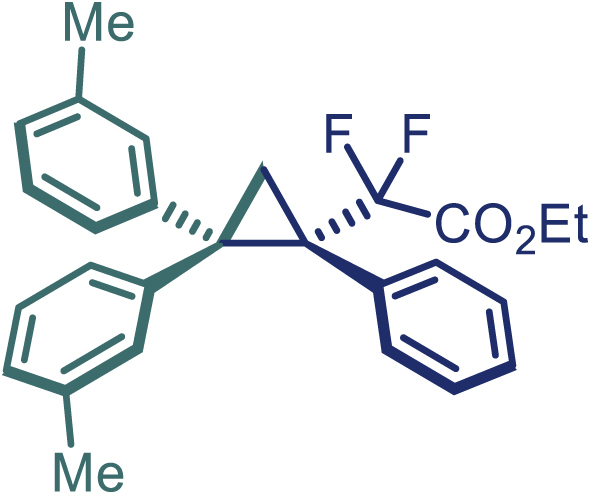

**41**, White solid; mp: 73–74°C; ^**1**^**H NMR** (600 MHz, CDCl_3_) δ 7.51 (d, *J* = 7.8 Hz, 1H), 7.45 (s, 1H), 7.38–7.28 (m, 2H), 7.21 (t, *J* = 7.8 Hz, 1H), 7.14–7.06 (m, 3H), 7.02 (d, *J* = 7.8 Hz, 1H), 6.96 (d, *J* = 7.8 Hz, 1H), 6.93 (s, 1H), 6.85 (t, *J* = 7.8 Hz, 1H), 6.68 (d, *J* = 7.8 Hz, 1H), 3.95 (q, *J* = 7.2 Hz, 2H), 2.41–2.38 (m, 1H), 2.37–2.34 (m, 4H), 2.09 (s, 3H), 1.00 (t, *J* = 7.2 Hz, 3H). ^**13**^**C NMR** (125 MHz, CDCl_3_) δ 163.6 (dd, *J* = 35.6, 32.7 Hz), 141.6, 141.0, 137.5, 137.0, 133.5 (d, *J* = 5.6 Hz), 131.6, 130.8, 129.8, 127.8, 127.72, 127.68, 127.5, 127.4, 127.3, 126.7, 126.2, 116.4 (t, *J* = 255.3 Hz), 62.3, 42.7, 40.6 (dd, *J* = 26.4, 21.1 Hz), 21.5, 21.3, 20.5 (t, *J* = 4.4 Hz), 13.6. ^**19**^**F NMR** (470 MHz, CDCl_3_) δ −102.7 (d, *J* = 248.6 Hz), −105.0 (d, *J* = 248.6 Hz). **IR** (Film): 3057, 2924, 2253, 1766, 1603, 1449, 1264, 1132, 906, 726, 649 cm^–1^. [α]25 D = −19.8, (c = 1.00, CH_2_Cl_2_).

**Figure:**
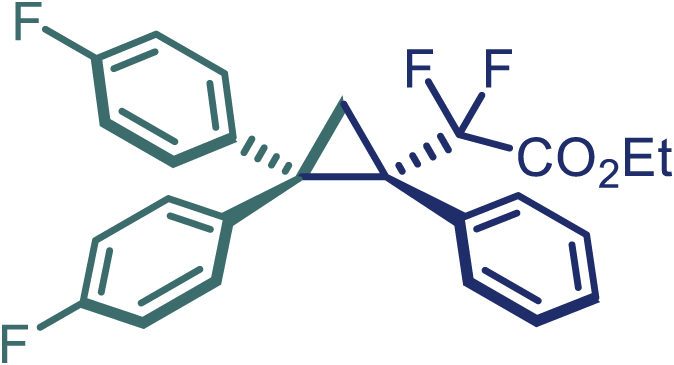

**42**, Colorless oil; ^**1**^**H NMR** (500 MHz, CDCl_3_) δ 7.65–7.60 (m, 2H), 7.34–7.26 (m, 2H), 7.16–7.09 (m, 3H), 7.08–6.99 (m, 4H), 6.69–6.63 (m, 2H), 3.97 (q, *J* = 7.1 Hz, 2H), 2.41–2.35 (m, 2H), 1.00 (t, *J* = 7.1 Hz, 3H). ^**13**^**C NMR** (150 MHz, CDCl_3_) δ 163.4 (dd, *J* = 35.7, 32.5 Hz), 161.7 (d, *J* = 245.3 Hz), 161.0 (d, *J* = 245.5 Hz), 137.3, 136.7 (d, *J* = 3.1 Hz), 133.0 (d, *J* = 5.6 Hz), 131.6 (d, *J* = 7.9 Hz), 130.4 (d, *J* = 8.1 Hz), 128.04, 128.00, 118.0, 116.3 (d, *J* = 257.3 Hz), 115.1 (d, *J* = 21.7 Hz), 114.7 (d, *J* = 21.3 Hz), 62.4, 41.2, 40.9 (dd, *J* = 26.1, 21.0 Hz), 20.8 (t, *J* = 4.5 Hz), 13.6. ^**19**^**F NMR** (564 MHz, CDCl_3_) δ −102.7 (d, *J* = 249.0 Hz), −105.2 (d, *J* = 249.0 Hz), (−115.7)-(-115.8) (m), (−116.3)-(-116.4) (m). **HRMS** (ESI) *m/z* calculated C_25_H_20_F_4_O_2_ [M]^+^ 428.1290, found 428.1292. **IR** (Film): 2926, 2360, 1751, 1511, 1230 cm^–1^. [α]25 D = −22.6 (c = 1.00, CH_2_Cl_2_).

**Figure:**
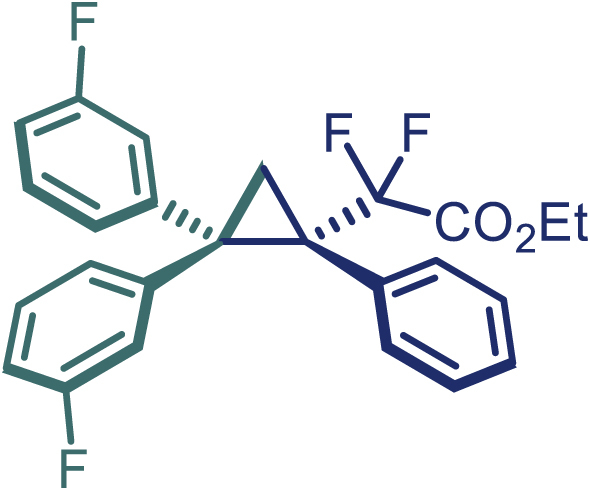

**43**, White solid; mp: 84–85°C; ^**1**^**H NMR** (600 MHz, CDCl_3_) δ 7.46 (d, *J* = 7.8 Hz, 1H), 7.36 (d, *J* = 10.2 Hz, 1H), 7.33–7.28 (m, 3H), 7.17–7.10 (m, 3H), 6.97–6.92 (m, 2H), 6.90 (d, *J* = 7.8 Hz, 1H), 6.83–6.81 (m, 1H), 6.63–6.60 (m, 1H), 3.98 (q, *J* = 7.2 Hz, 2H), 2.43–2.40 (m, 1H), 2.39–2.36 (m, 1H), 1.01 (t, *J* = 7.2 Hz, 3H). ^**13**^**C NMR** (150 MHz, CDCl_3_) δ 163.3 (dd, *J* = 35.7, 32.0 Hz), 162.5 (d, *J* = 245.2 Hz), 162.2 (d, *J* = 246.3 Hz), 143.4 (d, *J* = 7.5 Hz), 142.9 (d, *J* = 7.0 Hz), 132.6 (d, *J* = 5.1 Hz), 131.5, 129.7 (d, *J* = 8.5 Hz), 129.2 (d, *J* = 8.2 Hz), 128.09 (d, *J* = 6.9 Hz), 128.07, 125.9, 124.6 (d, *J* = 3.3 Hz), 117.2 (d, *J* = 21.6 Hz), 116.1 (t, *J* = 255.5 Hz), 116.0 (d, *J* = 21.9 Hz), 114.0 (d, *J* = 20.9 Hz), 113.3 (d, *J* = 21.3 Hz), 62.5, 41.8, 41.1 (dd, *J* = 26.3, 20.9 Hz), 20.6 (t, *J* = 4.6 Hz), 13.6. ^**19**^**F NMR** (564 MHz, CDCl_3_) δ −102.9 (d, *J* = 249.6 Hz), −105.5 (d, *J* = 249.6 Hz), (−113.1)-(-113.4) (m). **HRMS** (ESI) *m/z* calculated C_25_H_20_F_4_O_2_ [M]^+^ 428.1290, found 428.1292. **IR** (Film): 3065, 2963, 2925, 1753, 1587, 1487, 1261, 1183 cm^–1^. [α]25 D = −38.1, (c = 1.00, CH_2_Cl_2_).

**Figure:**
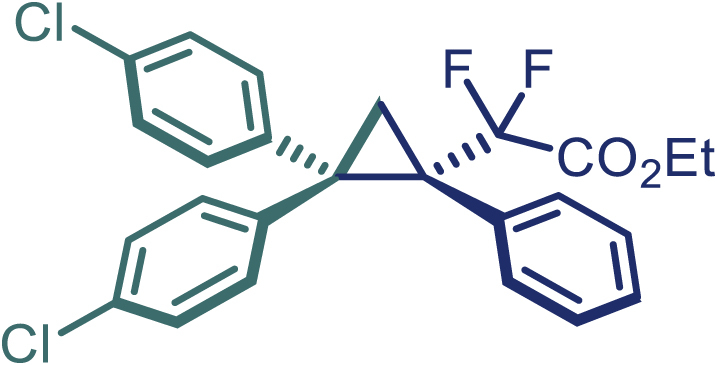

**44**, White solid; mp: 94–95°C; ^**1**^**H NMR** (500 MHz, CDCl_3_) δ 7.58 (d, *J* = 7.6 Hz, 2H), 7.33–7.27 (m, 4H), 7.17–7.13 (m, 3H), 7.02 (d, *J* = 8.7 Hz, 2H), 6.95 (d, *J* = 8.7 Hz, 2H), 3.97 (q, *J* = 7.1 Hz, 2H), 2.40–2.35 (m, 2H), 1.00 (t, *J* = 7.1 Hz, 3H). ^**13**^**C NMR** (150 MHz, CDCl_3_) δ 163.3 (dd, *J* = 35.6, 30.5 Hz), 139.7, 139.2, 132.8, 132.7 (d, *J* = 5.5 Hz), 132.2, 131.4, 130.2, 128.5, 128.2, 128.1, 128.0, 116.2 (t, *J* = 257.3 Hz), 62.5, 41.4, 41.0 (dd, *J* = 26.3, 20.9 Hz), 20.6 (t, *J* = 4.5 Hz), 13.6. ^**19**^**F NMR** (564 MHz, CDCl_3_) δ −102.6 (d, *J* = 249.5 Hz), −105.3 (d, *J* = 249.5 Hz). **HRMS** (ESI) *m/z* calculated C_25_H_20_Cl_2_F_2_O_2_ [M]^+^ 460.0707, found 460.0701. IR (Film): 3055, 2985, 2253, 1422, 1263, 902, 719, 649 cm^–1^. [α]25 D = −22.6, (c = 1.00, CH_2_Cl_2_).

**Figure:**
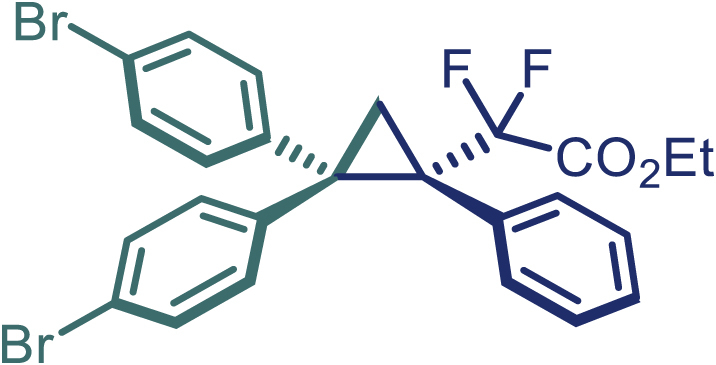

**45**, Colorless oil; ^**1**^**H NMR** (600 MHz, CDCl_3_) δ 7.52 (d, *J* = 7.8 Hz, 2H), 7.45 (d, *J* = 8.6 Hz, 2H), 7.34–7.27 (m, 2H), 7.17–7.13 (m, 3H), 7.12–7.08 (m, 2H), 6.97–6.94 (m, 2H), 3.97 (q, *J* = 7.1 Hz, 2H), 2.39–2.34 (m, 2H), 1.00 (t, *J* = 7.1 Hz, 3H). ^**13**^**C NMR** (125 MHz, CDCl_3_) δ 163.3 (dd, *J* = 35.6, 32.7 Hz), 140.2, 139.6, 132.7 (d, *J* = 5.4 Hz), 131.7, 131.4, 131.0, 130.6, 128.19, 128.17, 121.0, 120.4, 116.2 (t, *J* = 257.1 Hz), 62.5, 41.5, 41.0 (dd, *J* = 26.4, 21.0 Hz), 20.6 (t, *J* = 4.3 Hz), 13.6. ^**19**^**F NMR** (470 MHz, CDCl_3_) δ −102.6 (d, *J* = 249.5 Hz), −105.3 (d, *J* = 249.5 Hz). **IR** (Film): 3052, 2985, 2253, 1767, 1491, 1421, 1264, 907, 728, 703, 649 cm^–1^. [α]25 D = −27.6, (c = 1.00, CH_2_Cl_2_).

**Figure:**
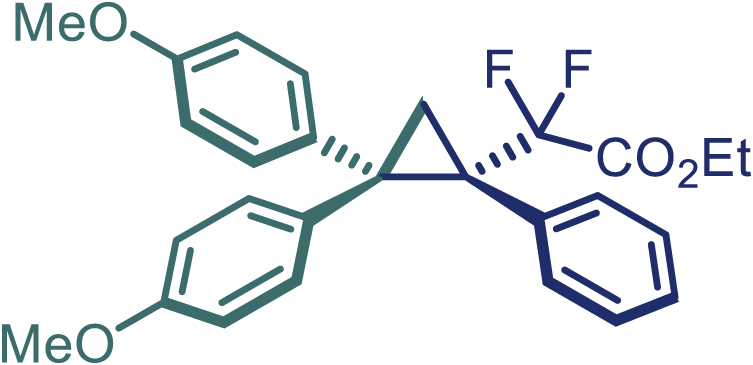

**46**, Colorless oil; ^**1**^**H NMR** (500 MHz, CDCl_3_) δ 7.56 (d, *J* = 7.9 Hz, 2H), 7.35–7.30 (m, 2H), 7.14–7.07 (m, 3H), 7.00 (d, *J* = 8.8 Hz, 2H), 6.85 (d, *J* = 8.8 Hz, 2H), 6.50 (d, *J* = 8.9 Hz, 2H), 3.95 (q, *J* = 7.1 Hz, 2H), 3.78 (s, 3H), 3.60 (s, 3H), 2.37–2.32 (m, 2H), 1.01 (t, *J* = 7.1 Hz, 3H). ^**13**^**C NMR** (125 MHz, CDCl_3_) δ 163.7 (dd, *J* = 36.0, 32.6 Hz), 158.2, 157.5, 134.1, 133.7 (d, *J* = 5.7 Hz), 133.6, 131.7, 131.0, 129.8, 127.9, 127.7, 116.5 (t, *J* = 256.5 Hz), 113.5, 113.1, 62.3, 55.2, 55.0, 41.3, 40.9 (dd, *J* = 26.6, 21.1 Hz), 20.8 (t, *J* = 4.5 Hz), 13.6. ^**19**^**F NMR** (470 MHz, CDCl_3_) δ −102.8 (d, *J* = 248.6 Hz), −104.8 (d, *J* = 248.6 Hz). **HRMS** (ESI) *m/z* calculated C_27_H_26_F_2_O_4_ [M]^+^ 452.1688, found 452.1691. **IR** (Film): 3046, 2253, 1766, 1512, 1264, 905, 726, 649 cm^–1^. [α]25 D = −9.2 (c = 1.00, CH_2_Cl_2_).

**Figure:**
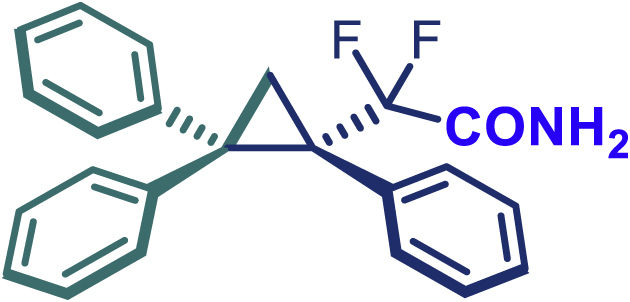

**47**, White solid; mp: 98–99°C; ^**1**^**H NMR** (500 MHz, CDCl_3_) δ 7.67 (d, *J* = 7.6 Hz, 2H), 7.33–7.28 (m, 4H), 7.20 (t, *J* = 7.4 Hz, 1H), 7.11–7.03 (m, 5H), 6.94 (t, *J* = 7.6 Hz, 2H), 6.86 (t, *J* = 7.3 Hz, 1H), 5.73 (s, 1H), 5.64 (s, 1H), 2.47–2.44 (m, 1H), 2.38 (d, *J* = 6.0 Hz, 1H). ^**13**^**C NMR** (150 MHz, CDCl_3_) δ 166.0 (dd, *J* = 33.2, 30.1 Hz), 141.7, 140.7, 133.7 (d, *J* = 4.9 Hz), 132.0, 130.2, 128.8, 128.0, 127.8, 127.6, 126.6, 125.9, 117.8 (t, *J* = 258.4 Hz), 41.8, 40.4 (dd, *J* = 26.7, 21.8 Hz), 20.6 (t, *J* = 4.2 Hz). ^**19**^**F NMR** (564 MHz, CDCl_3_) δ −102.8 (d, *J* = 248.0 Hz), −105.1 (d, *J* = 248.0 Hz). **HRMS** (ESI) *m/z* calculated C_23_H_19_F_2_NO [M]^+^ 363.1326, found 363.1327. **IR** (Film): 3035, 2250, 1740, 1501, 1151, 750, 637 cm^–1^. [α]25 D = −15.3 (c = 1.00, CH_2_Cl_2_).

**Figure:**
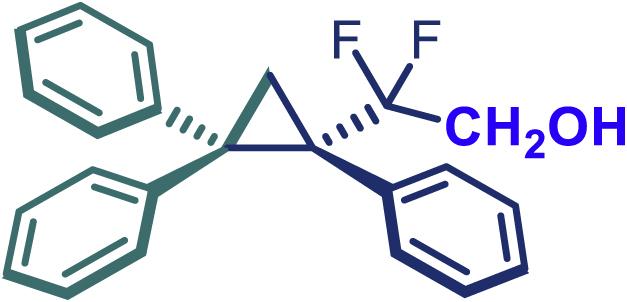

**48**, Colorless oil; ^**1**^**H NMR** (500 MHz, CDCl_3_) δ 7.69 (d, *J* = 7.6 Hz, 2H), 7.38–7.29 (m, 4H), 7.22–7.19 (m, 1H), 7.16–7.06 (m, 5H), 6.95 (t, *J* = 7.6 Hz, 2H), 6.86 (t, *J* = 7.3 Hz, 1H), 3.56–3.41 (m, 2H), 2.40 (t, *J* = 5.1 Hz, 1H), 2.14 (d, *J* = 5.7 Hz, 1H), 1.65 (s, 1H). ^**13**^**C NMR** (125 MHz, CDCl_3_) δ 142.5, 141.1, 135.2 (d, *J* = 6.0 Hz), 131.1, 130.1, 129.0, 128.0, 127.6, 127.4, 126.4, 125.9, 123.0 (dd, *J* = 249.1, 246.3 Hz), 64.8 (dd, *J* = 34.4, 27.3 Hz), 41.8, 40.0 (dd, *J* = 25.9, 21.1 Hz), 19.8 (dd, *J* = 5.8, 3.7 Hz). ^**19**^**F NMR** (470 MHz, CDCl_3_) δ −105.9 (dd, *J* = 247.7, 12.7 Hz), −109.4 (dt, *J* = 247.5, 16.8 Hz). **IR** (Film): 2237, 1421, 1209, 1150, 920 cm^–1^. [α]25 D = −30.2, (c = 1.00, CH_2_Cl_2_).

**Figure:**
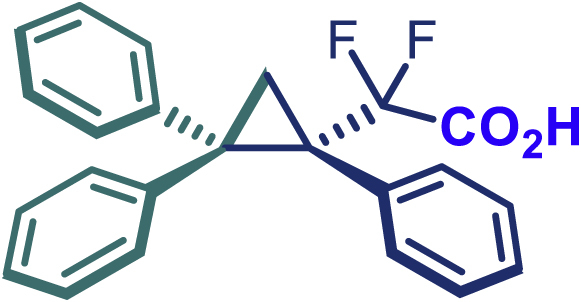

**49**, White solid; mp: 90–91°C; ^**1**^**H NMR** (500 MHz, CDCl_3_) δ 8.33–8.10 (m, 1H), 7.67 (d, *J* = 7.7 Hz, 2H), 7.36–7.27 (m, 4H), 7.22 (t, *J* = 7.4 Hz, 1H), 7.12–7.09 (m, 5H), 6.97 (t, *J* = 7.5 Hz, 2H), 6.89 (t, *J* = 7.3 Hz, 1H), 2.45 (t, *J* = 5.1 Hz, 1H), 2.36 (d, *J* = 6.0 Hz, 1H). ^**13**^**C NMR** (125 MHz, CDCl_3_) δ 167.2 (dd, *J* = 37.8, 33.5 Hz), 141.3, 140.6, 132.8 (d, *J* = 5.2 Hz), 131.7, 130.1, 128.9, 128.2, 128.0, 127.9, 127.7, 126.8, 126.1, 116.1 (t, *J* = 256.7 Hz), 42.8, 40.3 (dd, *J* = 25.9, 20.9 Hz), 20.5. ^**19**^**F NMR** (470 MHz, CDCl_3_) δ −103.4 (d, *J* = 251.4 Hz), −105.4 (d, *J* = 251.4 Hz). **IR** (Film): 3023, 2267, 1420, 922 cm^–1^. [α]25 D = −27.5, (c = 1.00, CH_2_Cl_2_).

**Figure:**
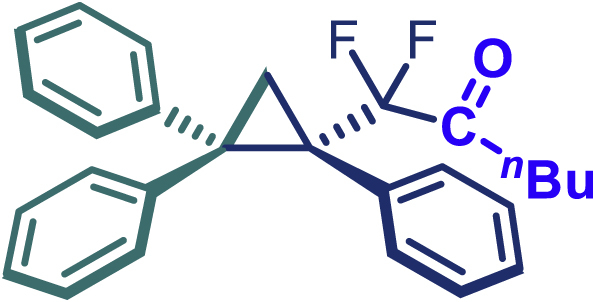

**50**, Colorless oil; ^**1**^**H NMR** (500 MHz, CDCl_3_) δ 7.69 (d, *J* = 7.8 Hz, 2H), 7.35–7.20 (m, 5H), 7.14–7.05 (m, 5H), 6.96 (t, *J* = 7.6 Hz, 2H), 6.88 (t, *J* = 7.3 Hz, 1H), 2.46–2.42 (m, 1H), 2.39–2.35 (m, 1H), 2.31–2.23 (m, 1H), 1.90–1.82 (m, 1H), 1.31–1.13 (m, 2H), 1.06–0.95 (m, 2H), 0.70 (t, *J* = 7.3 Hz, 3H). ^**13**^**C NMR** (150 MHz, CDCl_3_) δ 201.6 (dd, *J* = 35.8, 27.0 Hz), 141.8, 140.7, 133.3 (d, *J* = 5.8 Hz), 132.1, 130.3, 128.9, 128.1, 128.0, 127.71, 127.67, 126.6, 126.0, 118.2 (t, *J* = 259.5 Hz), 42.2, 40.3 (dd, *J* = 27.0, 21.4 Hz), 37.5, 24.3, 21.7, 20.5 (dd, *J* = 26.1, 22.2 Hz), 13.6. ^**19**^**F NMR** (564 MHz, CDCl_3_) δ −102.4 (d, *J* = 250.4 Hz), −108.4 (d, *J* = 250.4 Hz). **IR** (Film): 3050, 2250, 1715, 1433, 1251, 922, 630 cm^–1^. [α]25 D = −22.3, (c = 1.00, CH_2_Cl_2_).

#### Theoretical methodology

The quantum chemical calculations described in this work were carried out with Gaussian16 package.^42^ Geometry optimizations were conducted in the framework of the density functional theory (DFT) at the M06l^43^ level. The effective core potential SDD^44^ basis set was used to represent Rh atom, all the other atoms (C, H, O, F etc.) were described with 6-31G(d) basis set.^45^^,^^46^^,^^47^ The nature of the local minima was established with analytical frequencies calculations. Intrinsic reaction coordinate (IRC)^48^^,^^49^ calculations were carried out to ascertain the true nature of the transition states. 3D diagrams of the computed species were generated using CYLview visualization software.^50^ All structures are optimized under gas phase conditions.

## Data Availability

• All original crystal structures have been deposited at CCDC and are publicly available as of the date of publication. Compound 37: CCDC 2117854. Compound 44: CCDC 2117853. • This paper does not report original code. • Any additional information required to reanalyze the data reported in this paper can be obtained from the lead contact upon request.

## Footnotes

Supplemental information can be found online at https://doi.org/10.1016/j.isci.2022.105896.
