# Supplementary material for: Asymmetric [2+1] cycloaddition of difluoroalkyl-substituted carbenes with alkenes under rhodium catalysis: Synthesis of chiral difluoroalkyl-substituted cyclopropanes
Source: iScience. 2023 Feb 15;26(3):105896. doi: 10.1016/j.isci.2022.105896 (PMC10040897; doi:10.1016/j.isci.2022.105896)
Supplement: Document S1. Figures S1–S231, Data S1, and Tables S1–S3 [file mmc1.pdf]

iScience, Volume *26*

Supplemental information

Asymmetric [2+1] cycloaddition of difluoroalkyl-substituted carbenes with alkenes under rhodium catalysis: Synthesis of chiral difluoroalkyl-substituted cyclopropanes

Xinyu Zhang, Yongquan Ning, Chunqi Tian, Giuseppe Zanoni, and Xihe Bi

Table S1. Optimization of the reaction conditions, related to STAR Methods

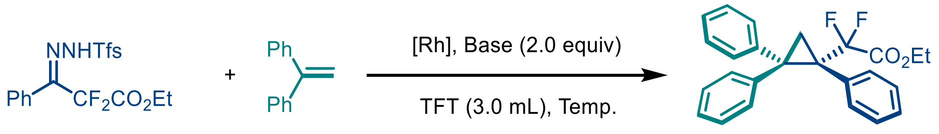

1a **2a** **24**

Entry	Catalyst	Base	Temp.	Yield	*ee*
1	Rh_2(*S*-PTAD)$_4$ (1 mol%)	Cs_2CO_3	25 °C	*N.R.*	--
2	Rh_2(*S*-PTAD)$_4$ (1 mol%)	KO^{t}Bu	25 °C	*N.R.*	--
3	Rh_2(*S*-PTAD)$_4$ (1 mol%)	KHDMS	25 °C	*N.R.*	--
4	Rh_2(*S*-DOSP)$_4$ (1 mol%)	DIPEA	25 °C	27%	--
5	Rh_2(*S*-BTPCP)$_4$ (1 mol%)	DIPEA	25 °C	*N.D.*	--
6	Rh_2(*S*-PTTL)$_4$ (1 mol%)	DIPEA	25 °C	90%	98%
7	Rh_2(*S*-PTAD)$_4$ (1 mol%)	DIPEA	25 °C	92%	99%
9	Rh_2(*S*-PTAD)$_4$ (1 mol%)	DIPEA	0 °C	46%	99%
10	Rh_2(*S*-PTAD)$_4$ (1 mol%)	DIPEA	-20 °C	*N.D.*	--
11	Rh_2(*S*-PTAD)$_4$ (0.5 mol%)	DIPEA	25 °C	*NMR quantitive*	99%
12	Rh_2(*S*-PTAD)$_4$ (0.25 mol%)	DIPEA	25 °C	*NMR quantitive*	99%
13[a]	Rh_2(*S*-PTAD)$_4$ (1 mol%)	DIPEA	25 °C	*N.D*	--
14[b]	Rh_2(*S*-PTAD)$_4$ (1 mol%)	DIPEA	25 °C	*N.D.*	--

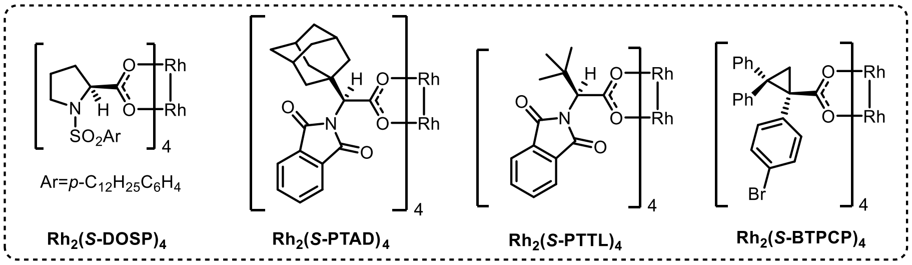

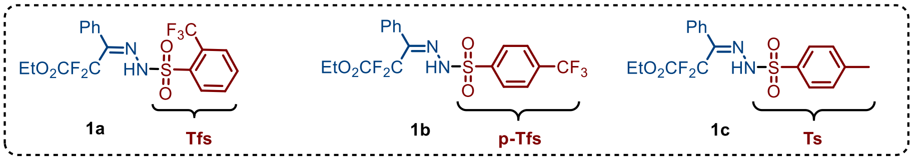

Reaction conditions: **1a** (0.2 mmol), **2a** (0.1 mmol), [Rh] catalyst (1 mol%), DIPEA (*N*,*N*-diisopropylethylamine) (0.2 mmol), benzotrifluoride (TFT, 3.0 mL), 12 h, under N_2. [a]**1b** was used. [b]**1c** was used

Table S2. Crystal Structure of 37 (CCDC No. 2117854)， related to STAR Methods

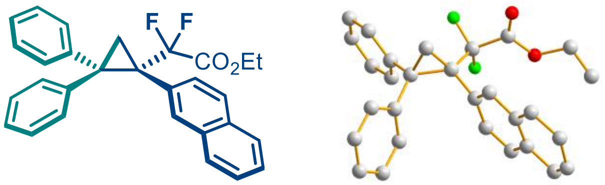

Empirical formula	$C_{29}H_{24}F_2O_2$
Temperature	293(2) K
Wavelength	0.71073 Å
Unit cell dimensions	a = 10.9993(10) Å<br>b = 11.4422(12) Å<br>c = 19.035(3) Å<br>alpha = 90 deg.<br>beta = 90 deg.<br>gamma = 90 deg.
Volume	2395.7(5) $Å^3$
Z	4
Calculated density	1.227 Mg/m^3
Absorption coefficient	0.109 mm^{-1}
F(000)	928
Crystal size	0.1 x 0.2 x 0.1 mm
Theta range for data collection	7.122 to 58.582 deg.
Reflections collected / unique	7991 / 4985 [R(int) = 0.1351]
Data / restraints / parameters	4985/0/299
Goodness-of-fit on F^2	0.912
Final R indices [I>2sigma(I)]	R_1 = 0.0939, wR_2 = 0.1992
Rindices (all data)	R_1 = 0.2444, wR_2 = 0.2827
Space group	P212121
Flack parameters	0.7

Table S3. Crystal Structure of 44 (CCDC No. 2117853), related to STAR Methods.

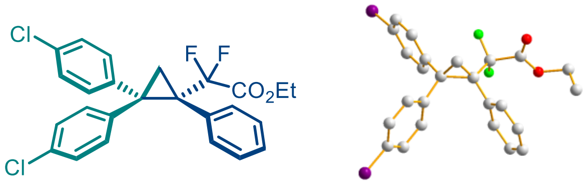

Empirical formula	$C_{25}H_{20}Cl_2F_2O_2$
Temperature	293(2) K

Wavelength	0.71073 Å
Unit cell dimensions	a = 10.3915(11) Å<br>b = 10.8708(10) Å<br>c = 11.0915(12) Å<br>alpha = 90 deg.<br>beta = 114.646(13) deg.<br>gamma = 90 deg.
Volume	1138.8(2) Å ^{3}
Z	2
Calculated density	1.345 Mg/m^3
Absorption coefficient	0.109 mm^{-1}
F(000)	476.0
Crystal size	0.1 x 0.2 x 0.1 mm
Theta range for data collection	7.036 to 58.468 deg.
Reflections collected / unique	5225 / 3847 [R(int) = 0.0868]
Data / restraints / parameters	3847/1/281
Goodness-of-fit on F^2	1.002
Final R indices [I>2sigma(I)]	R_1 = 0.0739, wR_2 = 0.1757
Rindices (all data)	R_1 = 0.0940, wR_2 = 0.2007
Space group	P21
Flack parameters	0.02

Figure S1-S43, Analytical data and copies of chiral HPLC traces related to scheme 2 and 3.

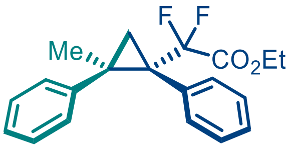

Chiralcel OD-H, 2 % *i*Pr-OH in hexane, 1 mL/min λ= 210 nm, tR= 5.983 min, major; tR= 6.822 min, minor.

Racemate:

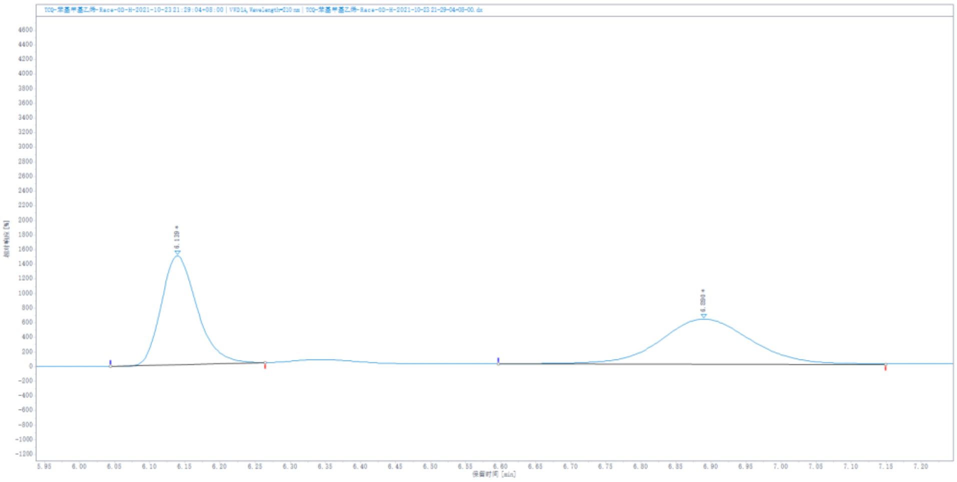

Enantiomer:

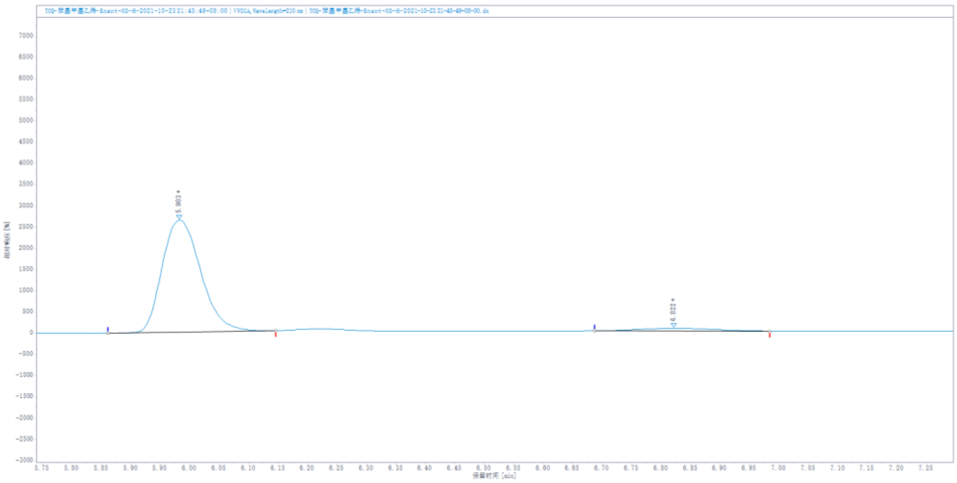

Figure S1. HPLC traces of compound 3

RetTime	Area	Area%	Hight	Hight%
5.983	14581.43	95.44	3219.59	97.57
6.822	696.97	4.56	80.06	2.43

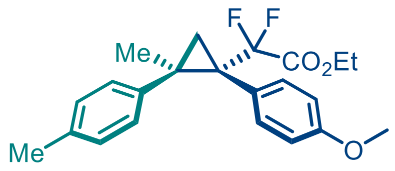

Chiralcel AD-H, 2 % *i*Pr-OH in hexane, 1 mL/min λ= 210 nm, tR= 7.262 min, minor; tR= 7.726 min, major.

Racemate:

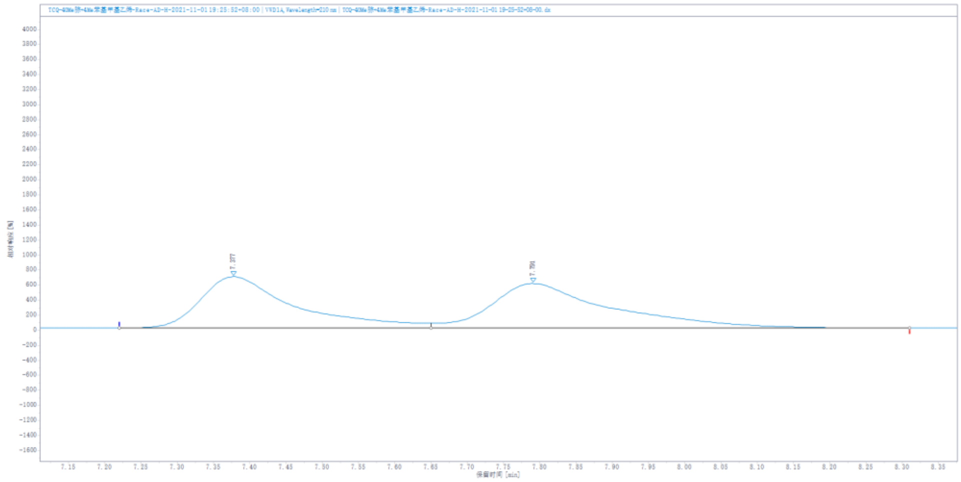

Enantiomer:

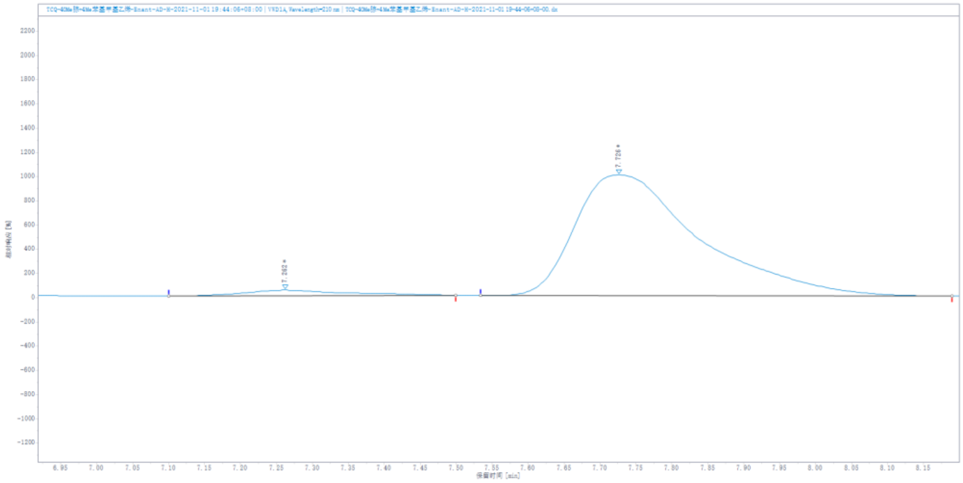

Figure S2. HPLC traces of compound 4

RetTime	Area	Area%	Hight	Hight%
7.262	1617.48	3.69	163.83	4.46
7.726	42202.04	96.31	3509.58	95.54

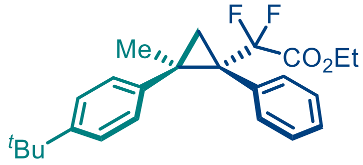

Chiralcel OD-H, 5 % *i*Pr-OH in hexane, 1 mL/min λ= 210 nm, tR= 7.348 min, minor; tR= 8.471 min, major.

Racemate:

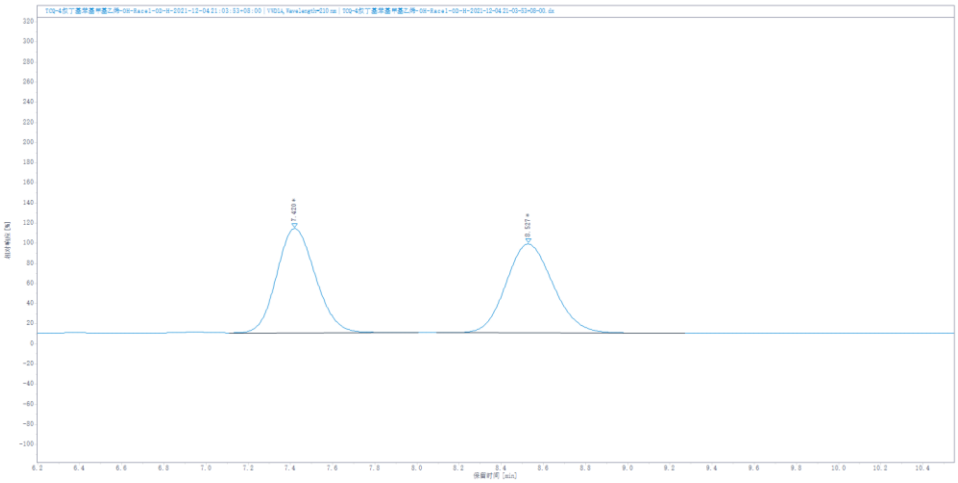

Enantiomer:

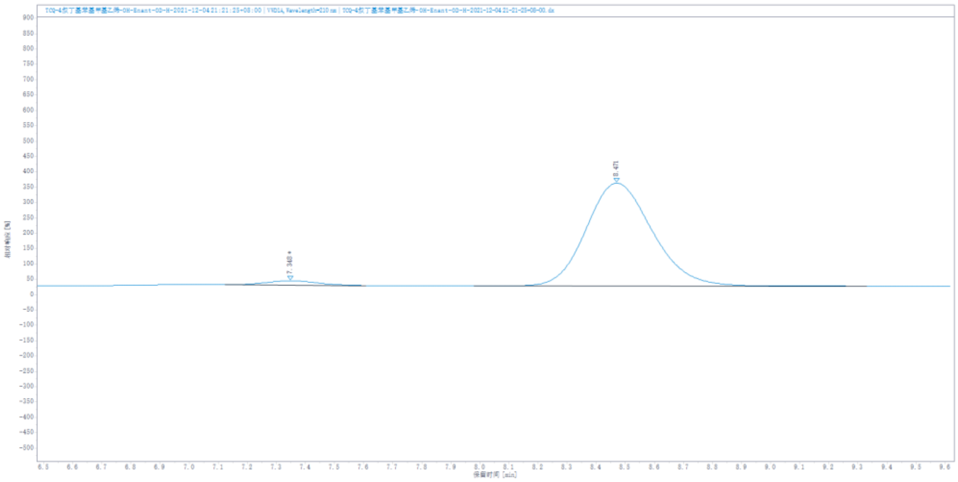

Figure S3. HPLC traces of compound 5

RetTime	Area	Area%	Hight	Hight%
7.348	357.33	3.07	29.60	3.98
8.471	11295.63	96.93	713.78	96.02

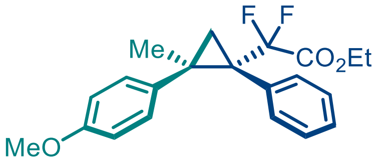

Chiralcel OD-H, 2 % *i*Pr-OH in hexane, 1 mL/min λ= 210 nm, tR= 6.402 min, major; tR= 7.467 min, minor.

Racemate:

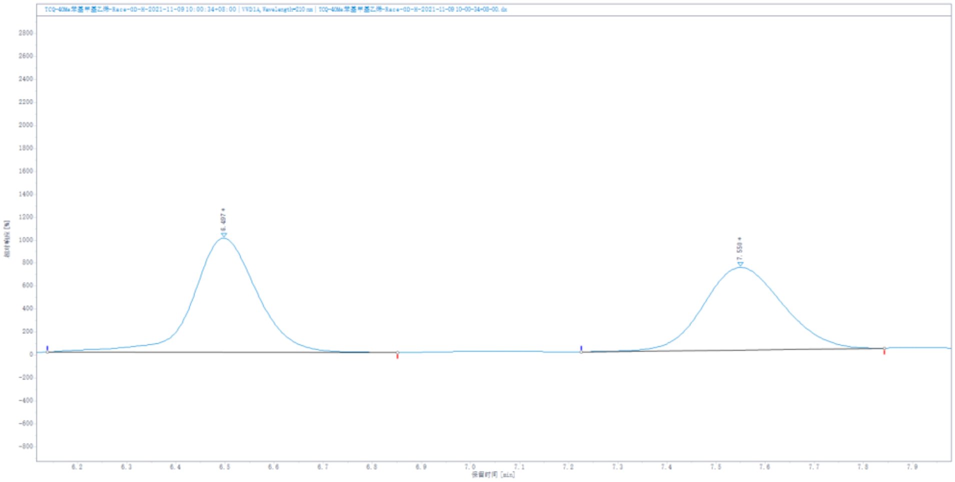

Enantiomer:

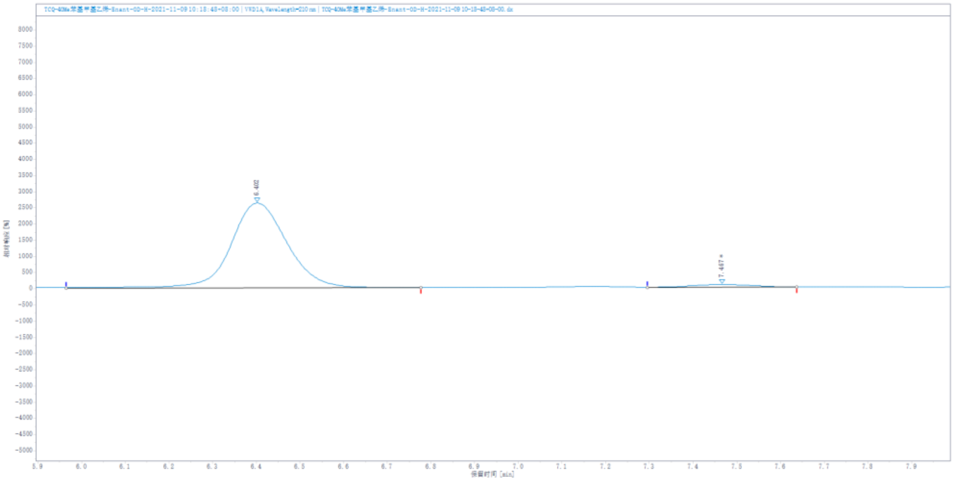

Figure S4. HPLC traces of compound 6

RetTime	Area	Area%	Hight	Hight%
6.402	26114.07	96.30	2892.46	96.72
7.467	1002.03	3.70	98.18	3.28

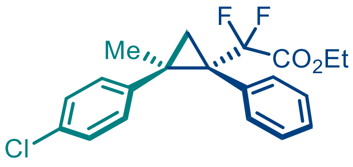

Chiralcel OD-H, 2 % *i*Pr-OH in hexane, 1 mL/min λ= 210 nm, tR= 14.738 min, minor; tR= 15.605 min, major.

Racemate:

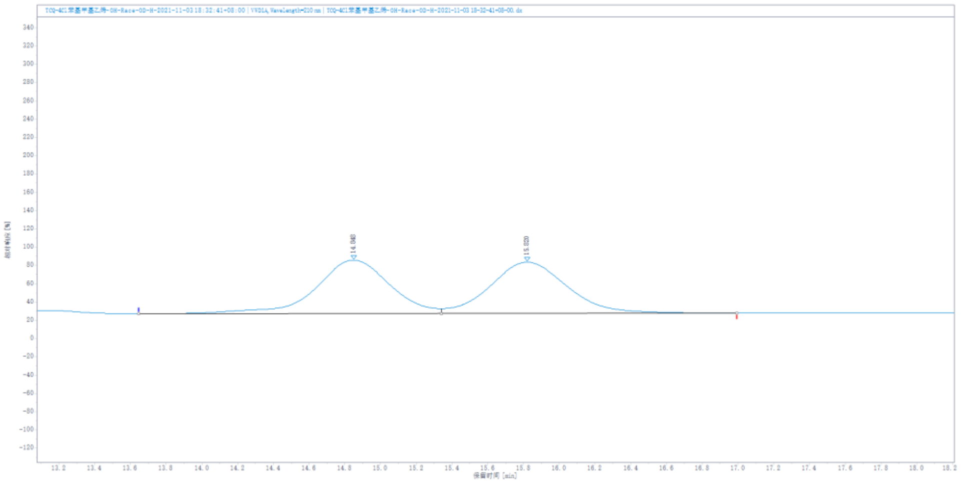

Enantiomer:

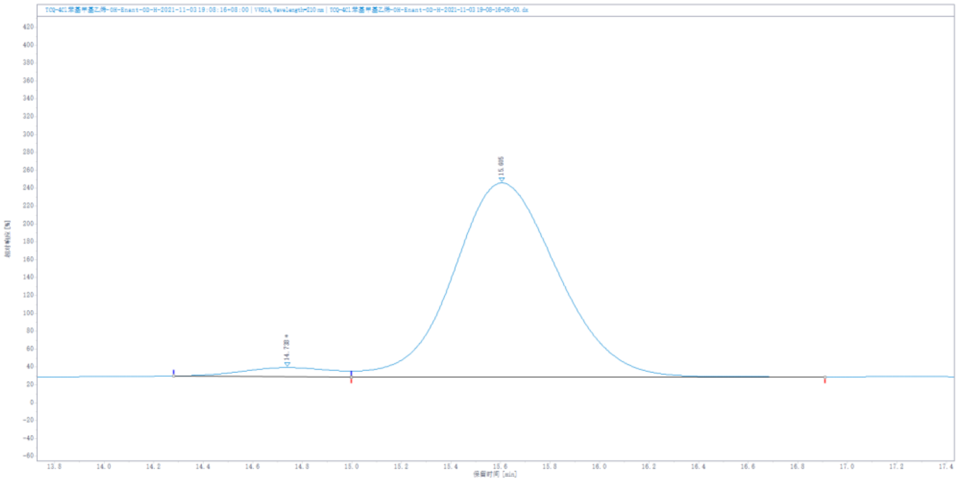

Figure S5. HPLC traces of compound 7

RetTime	Area	Area%	Hight	Hight%
14.738	536.20	3.82	21.36	4.43
15.605	13518.32	96.18	460.92	95.57

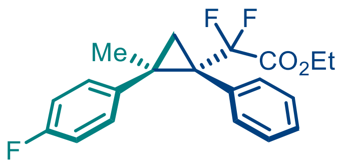

Chiralcel OD-H, 2 % *i*Pr-OH in hexane, 1 mL/min λ= 210 nm, tR= 13.827 min, minor; tR= 14.302 min, major.

Racemate:

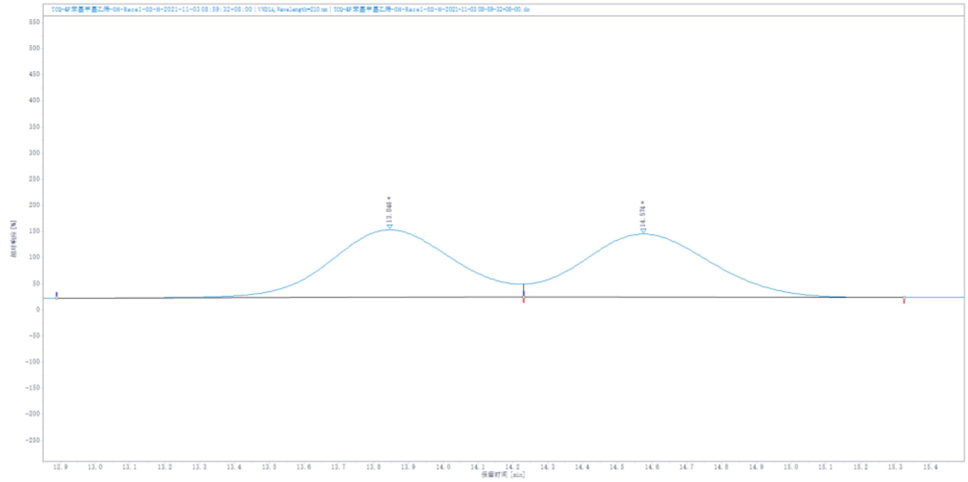

Enantiomer:

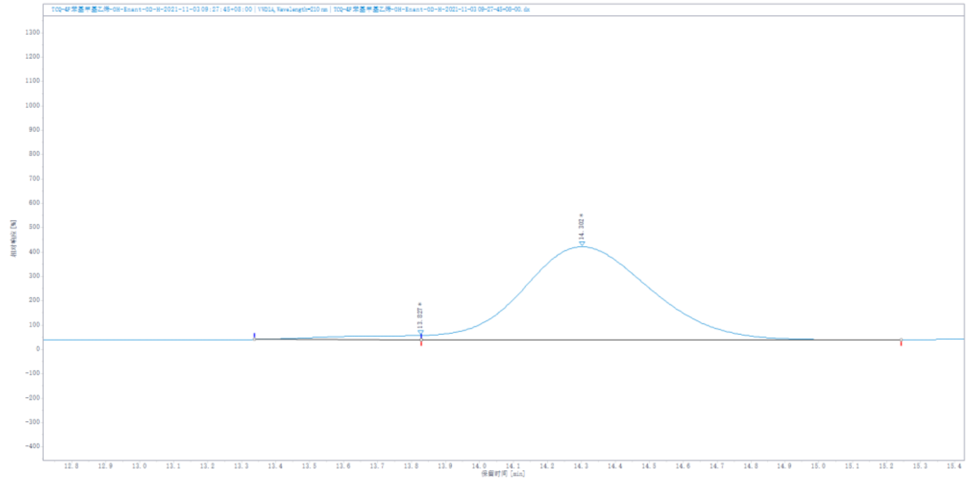

Figure S6. HPLC traces of compound 8

RetTime	Area	Area%	Hight	Hight%
13.827	449.95	3.09	25.67	4.54
14.302	14133.53	96.91	539.84	95.46

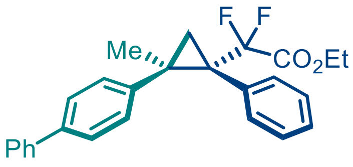

Chiralcel OD-H, 2 % *i*Pr-OH in hexane, 1 mL/min λ= 210 nm, tR= 9.687 min, minor; tR= 10.249 min, major.

Racemate:

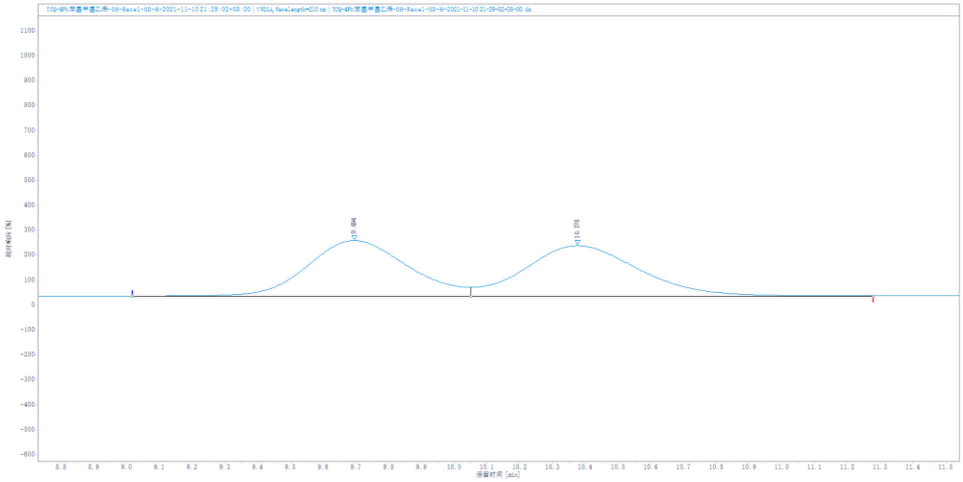

Enantiomer:

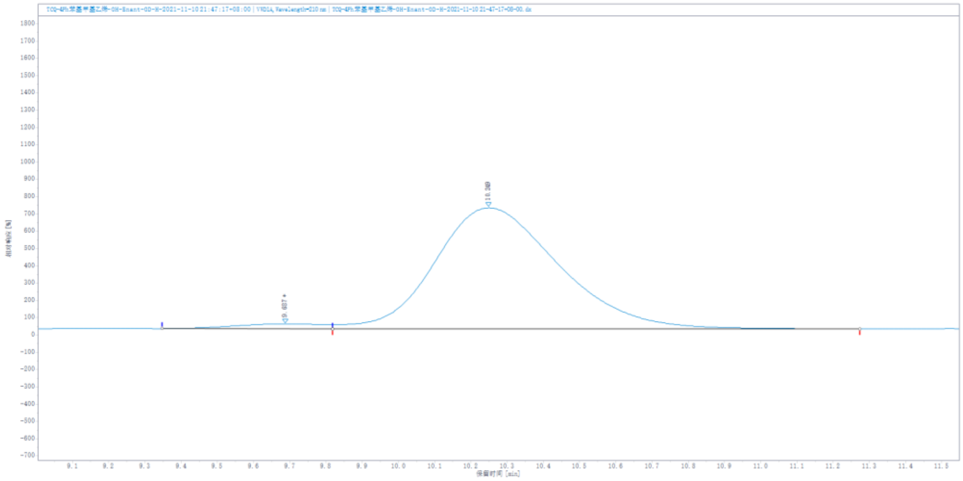

Figure S7. HPLC traces of compound 9

RetTime	Area	Area%	Hight	Hight%
9.687	727.20	2.66	43.06	3.72
10.249	26577.50	97.34	1114.08	96.28

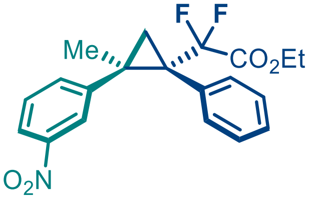

Chiralcel OD-H, 5 % *i*Pr-OH in hexane, 1 mL/min λ= 210 nm, tR= 6.344 min, major; tR= 6.876 min, minor.

Racemate:

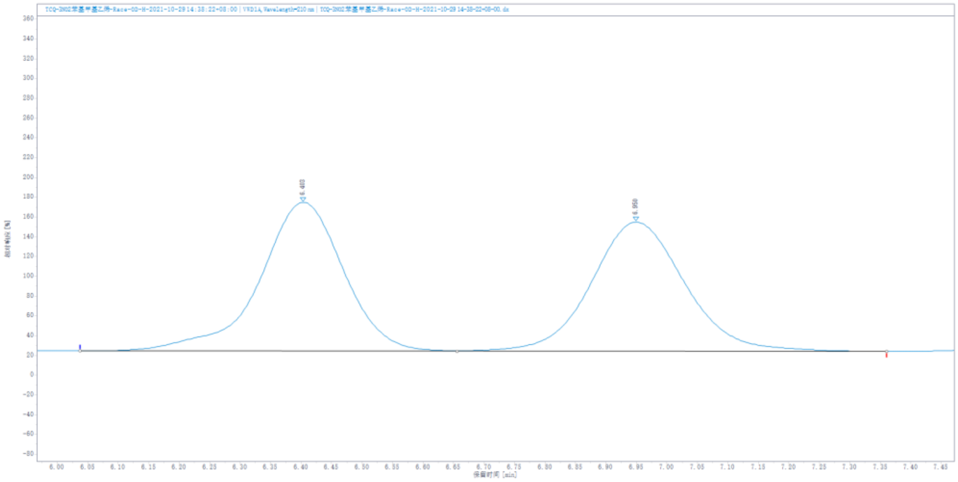

Enantiomer:

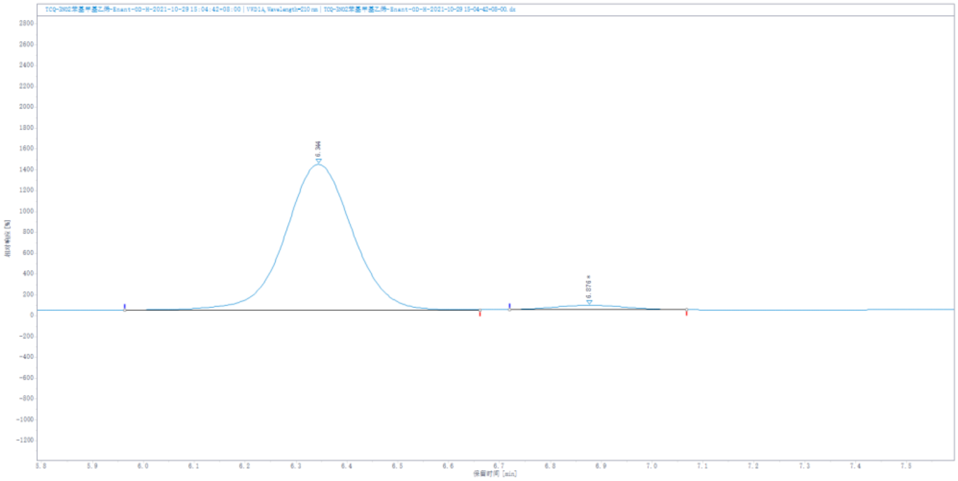

Figure S8. HPLC traces of compound 10

RetTime	Area	Area%	Hight	Hight%
6.344	18099.07	96.99	1970.15	97.12
6.876	560.78	3.01	58.32	2.88

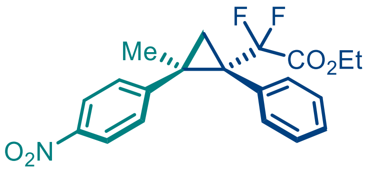

Chiralcel OD-H, 2 % *i*Pr-OH in hexane, 1 mL/min λ= 210 nm, tR= 9.257 min, major; tR= 10.764 min, minor.

Racemate:

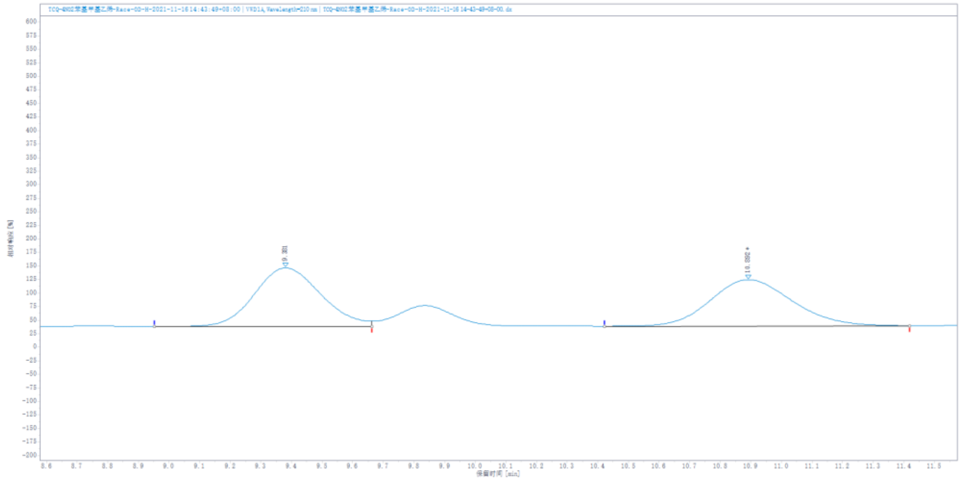

Enantiomer:

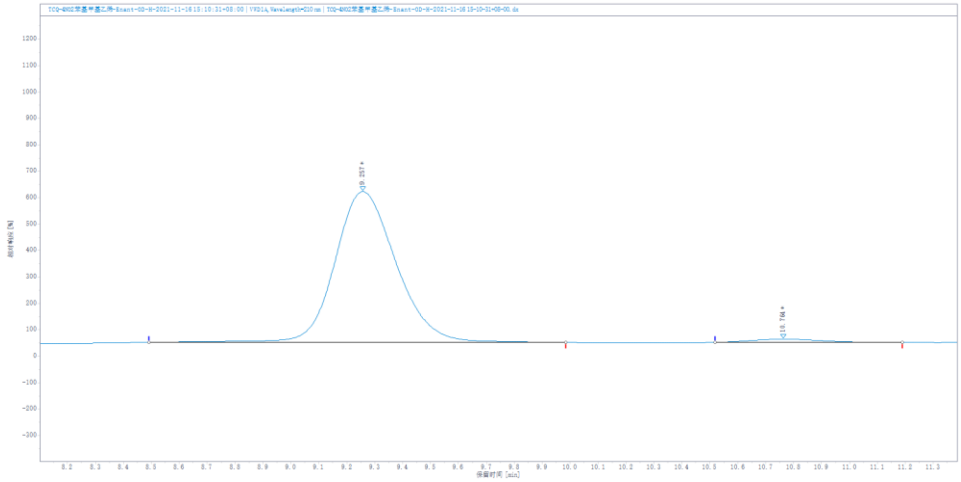

Figure S9. HPLC traces of compound 11

RetTime	Area	Area%	Hight	Hight%
9.257	8496.82	97.73	538.02	97.92
10.764	197.33	2.27	11.41	2.08

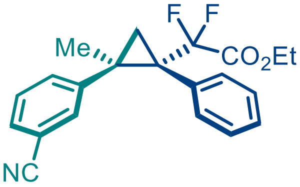

Chiralcel OD-H, 2 % *i*Pr-OH in hexane, 1 mL/min λ= 210 nm, tR= 9.680 min, major; tR= 12.280 min, minor.

Racemate:

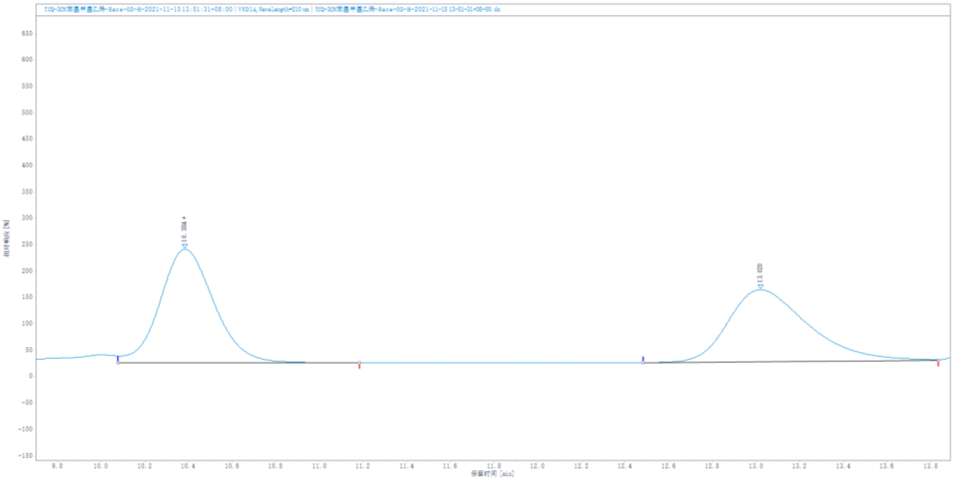

Enantiomer:

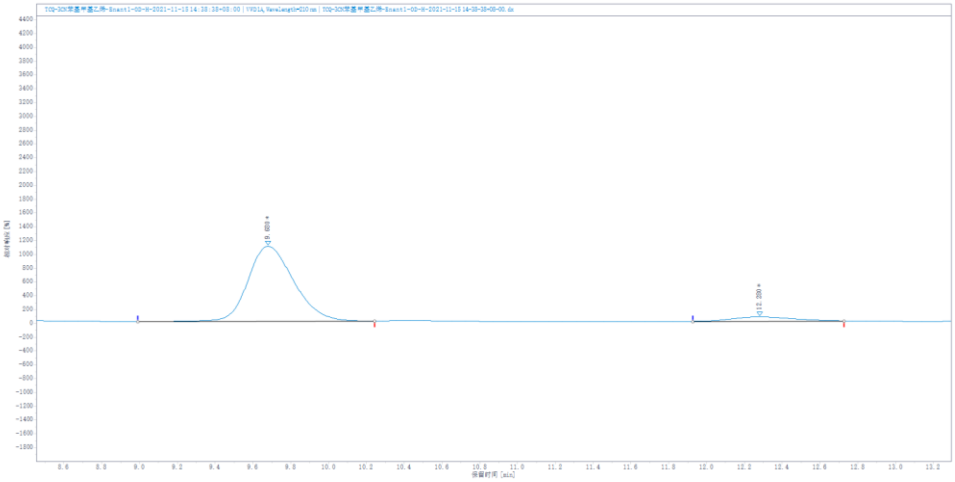

Figure S10. HPLC traces of compound 12

RetTime	Area	Area%	Hight	Hight%
9.680	31201.83	93.04	1861.24	94.61
12.280	2334.64	6.96	106.03	5.39

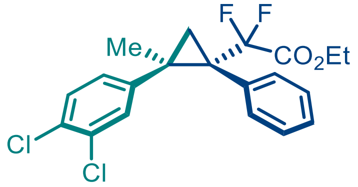

Chiralcel AD-H, 5 % *i*Pr-OH in hexane, 1 mL/min λ= 210 nm, tR= 11.901 min, minor; tR= 14.039 min, major.

Racemate:

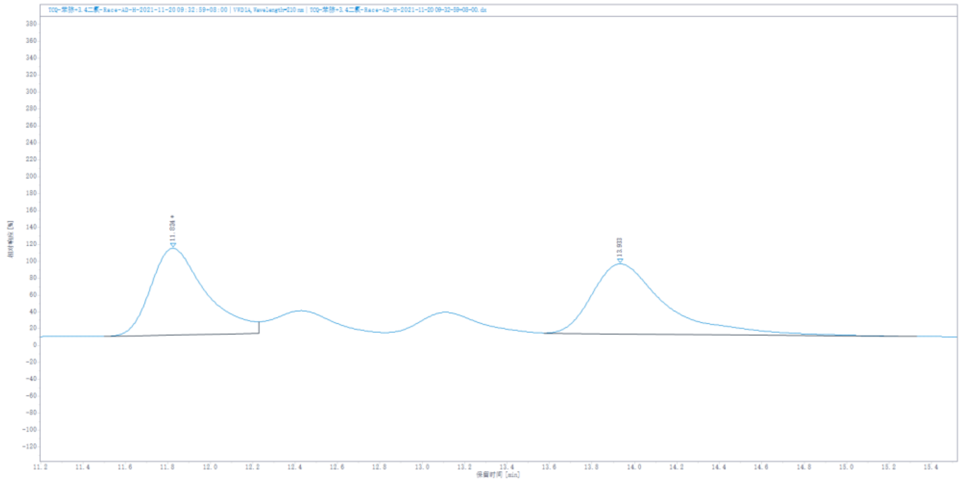

Enantiomer:

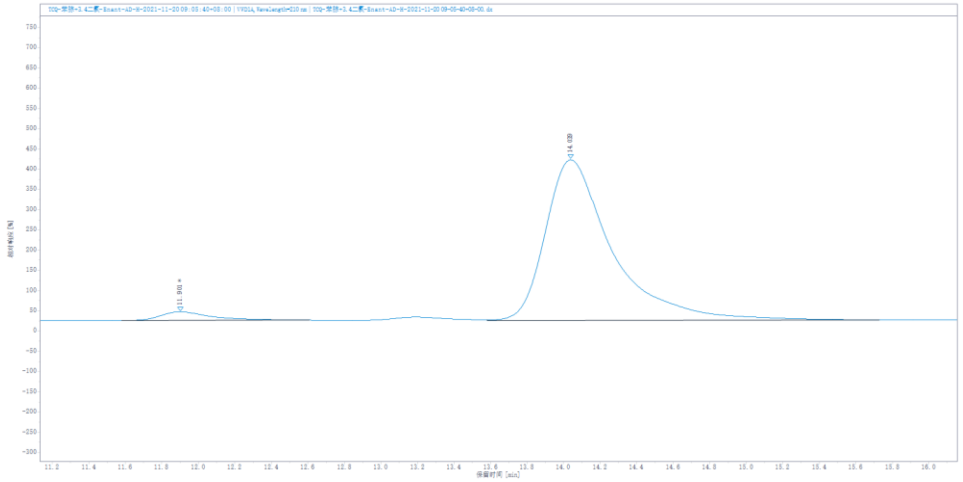

Figure S11. HPLC traces of compound 13

RetTime	Area	Area%	Hight	Hight%
11.901	727.12	3.92	37.79	5.11
14.039	17819.40	96.08	701.55	94.89

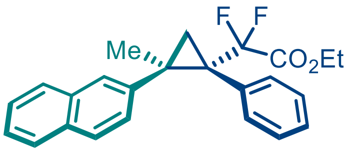

Chiralcel AD-H, 2 % *i*Pr-OH in hexane, 1 mL/min λ= 210 nm, tR= 6.157 min, major; tR= 6.730 min, minor.

Racemate:

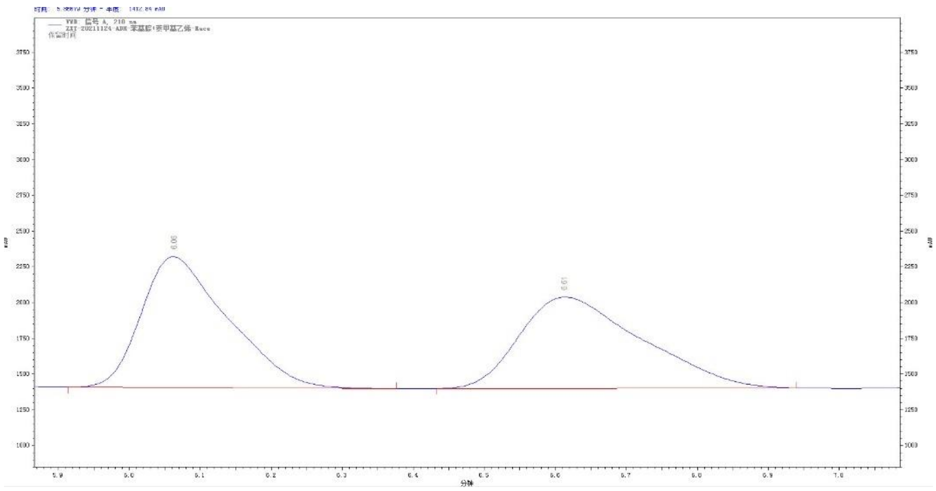

Enantiomer:

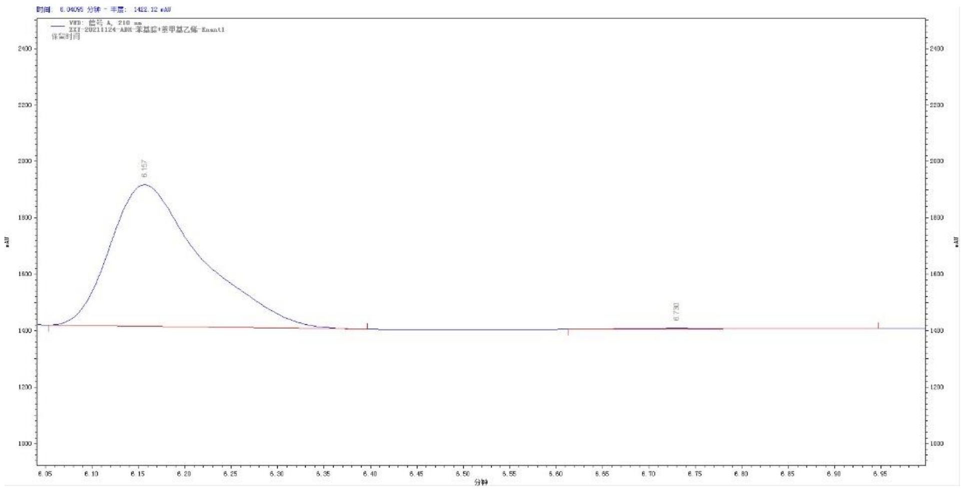

Figure S12. HPLC traces of compound 14

RetTime	Area	Area%	Hight	Hight%
6.157	57498232	99.10	8410164	99.38
6.730	523179	0.90	52849	0.62

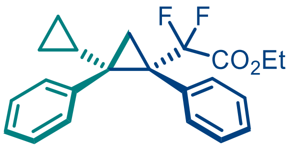

Chiralcel OD-H, 2 % *i*Pr-OH in hexane, 1 mL/min λ= 210 nm, tR= 15.608 min, minor; tR= 16.300 min, major.

Racemate:

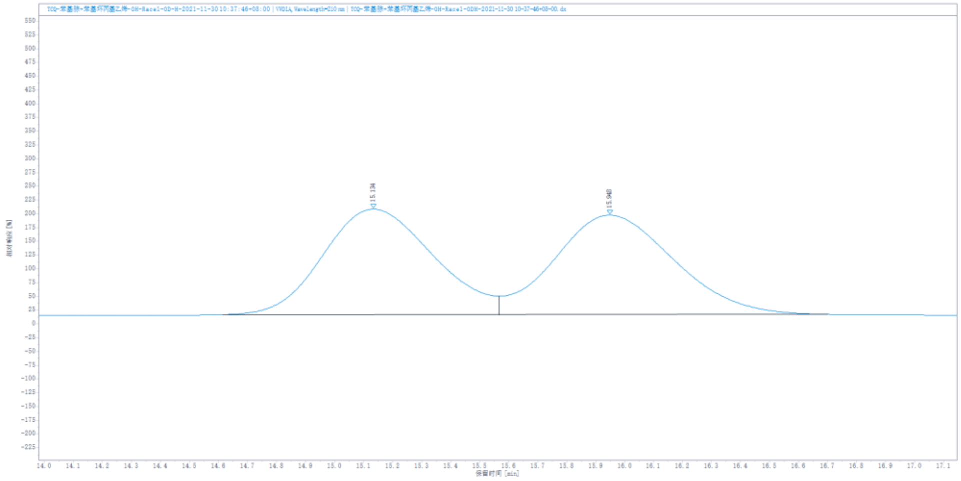

Enantiomer:

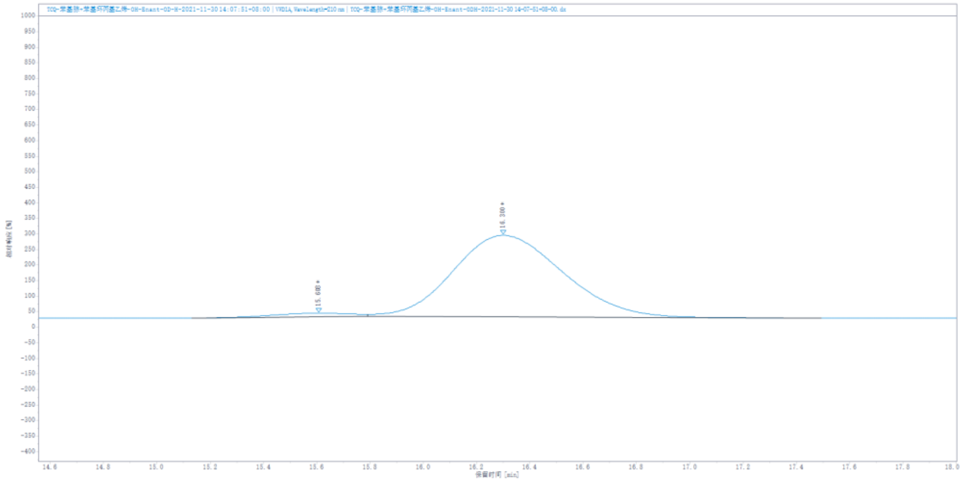

Figure S13. HPLC traces of compound 15

RetTime	Area	Area%	Hight	Hight%
15.608	340.00	2.95	16.54	4.05
16.300	11204.19	97.05	391.51	95.95

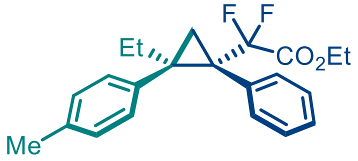

Chiralcel OD-H, 2 % *i*Pr-OH in hexane, 1 mL/min λ= 210 nm, tR= 13.074 min, minor; tR= 13.833 min, major.

Racemate:

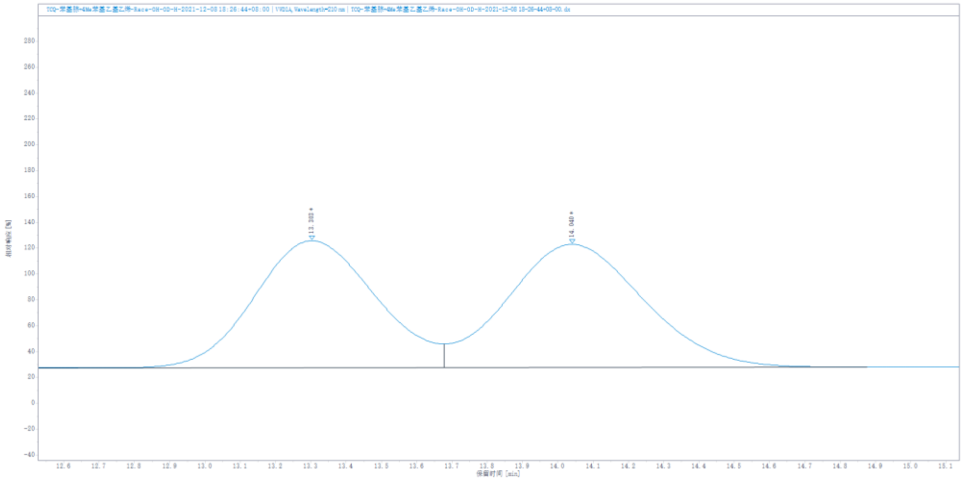

Enantiomer:

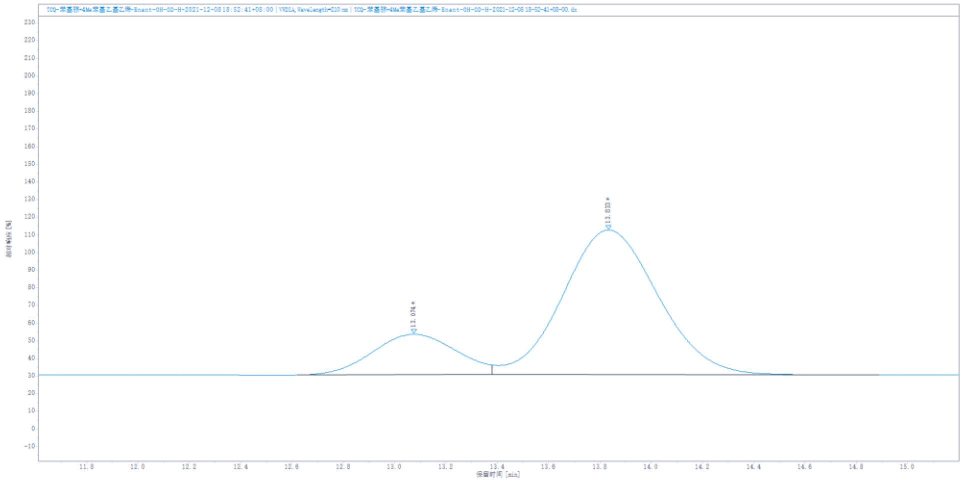

Figure S14. HPLC traces of compound 16

RetTime	Area	Area%	Hight	Hight%
13.074	670.16	19.59	29.67	21.77
13.833	2751.10	80.41	106.60	78.23

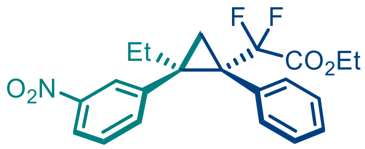

Chiralcel OD-H, 2 % *i*Pr-OH in hexane, 1 mL/min λ= 210 nm, tR= 7.328 min, major; tR= 8.180 min, minor.

Racemate:

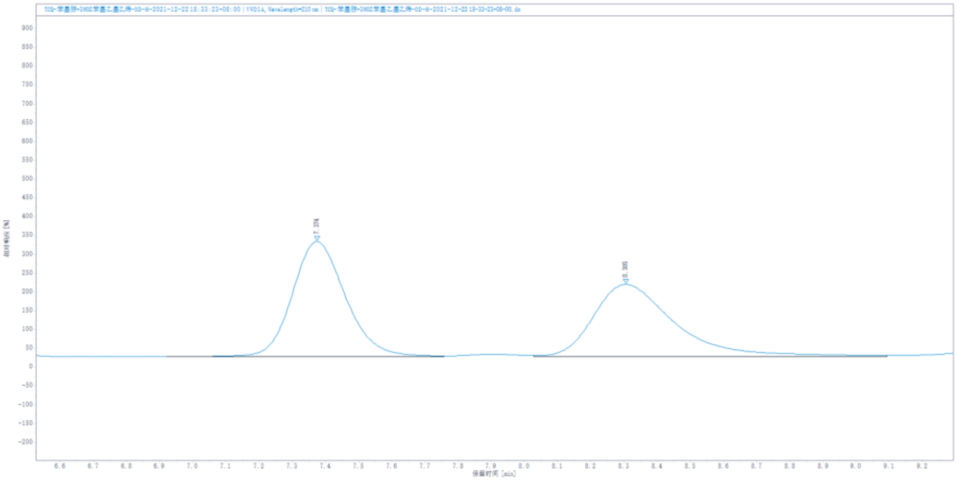

Enantiomer:

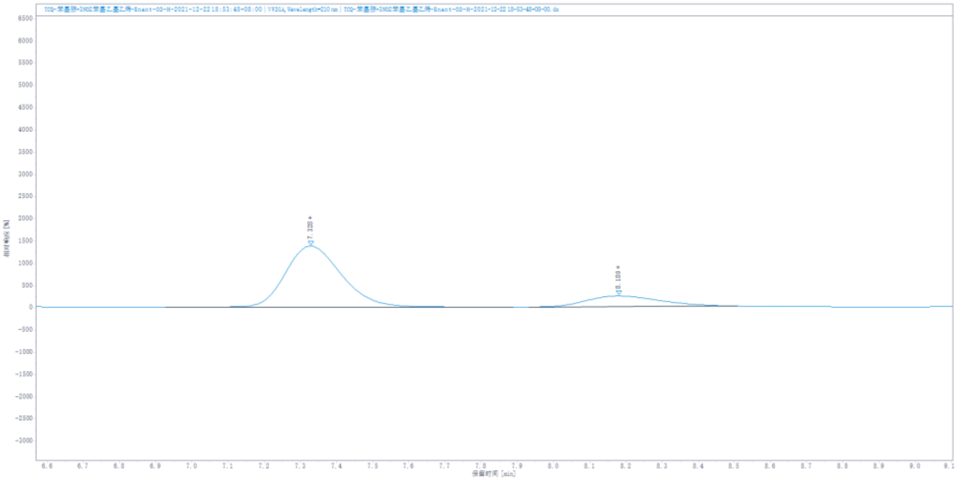

Figure S15. HPLC traces of compound 17

RetTime	Area	Area%	Hight	Hight%
7.328	27475.64	81.05	2563.19	85.05

8.180	6425.60	18.95	450.63	14.95

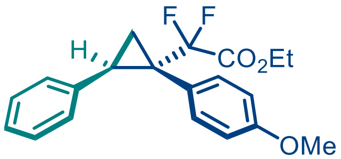

Chiralcel OD-H, 1 % *i*Pr-OH in hexane, 1 mL/min λ= 210 nm, tR= 10.627 min, minor; tR= 11.807 min, major.

Racemate:

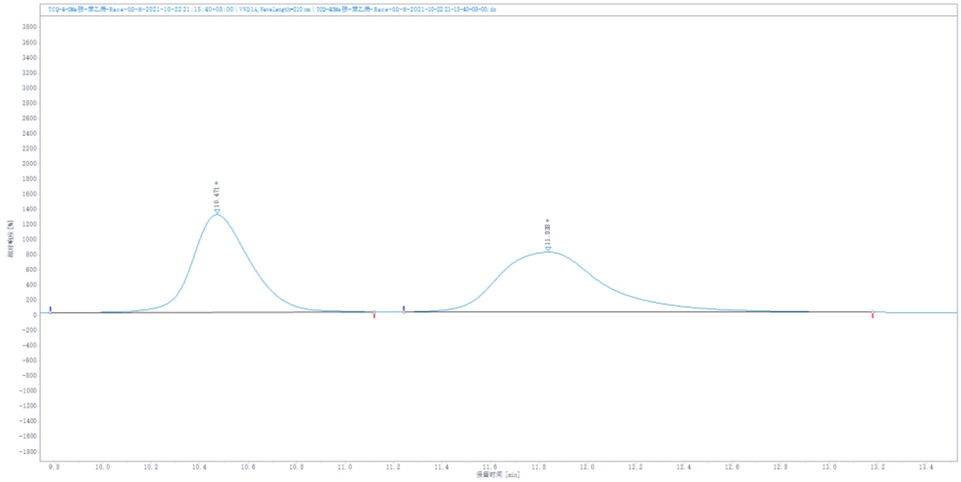

Enantiomer:

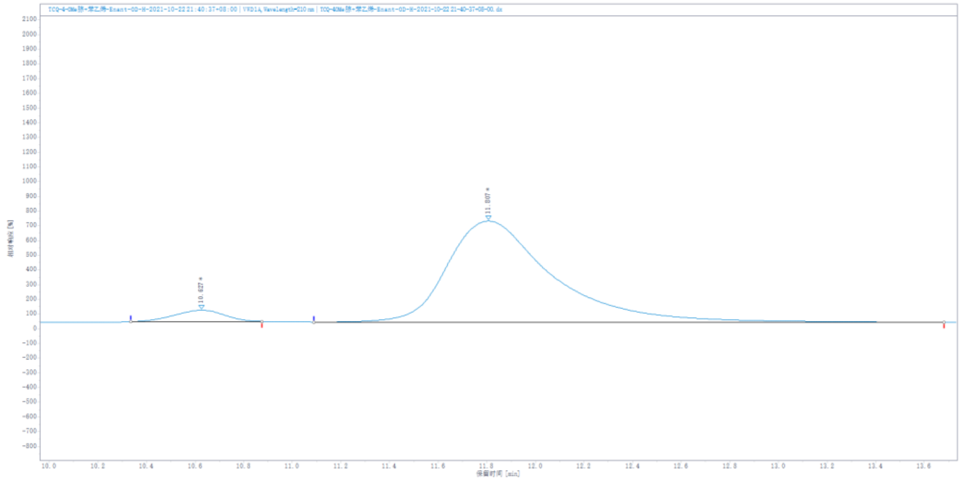

Figure S16. HPLC traces of compound 18

RetTime	Area	Area%	Hight	Hight%
10.627	846.37	5.04	59.43	10.08

11.807	15950.71	94.96	530.07	89.92

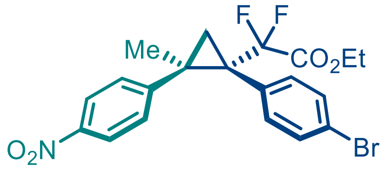

Chiralcel AD-H, 5 % *i*Pr-OH in hexane, 1 mL/min λ= 210 nm, tR= 11.169 min, minor; tR= 13.805 min, major.

Racemate:

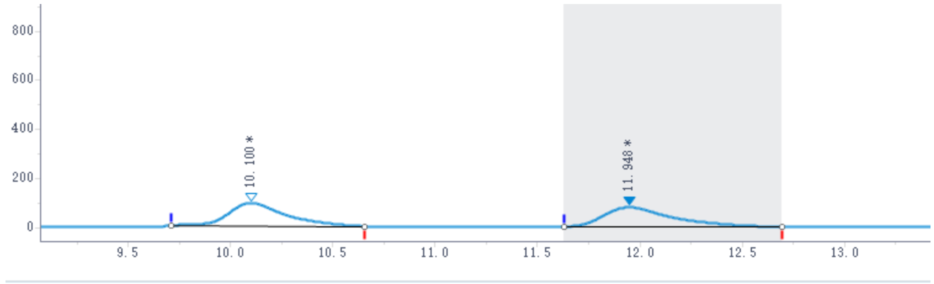

Enantiomer:

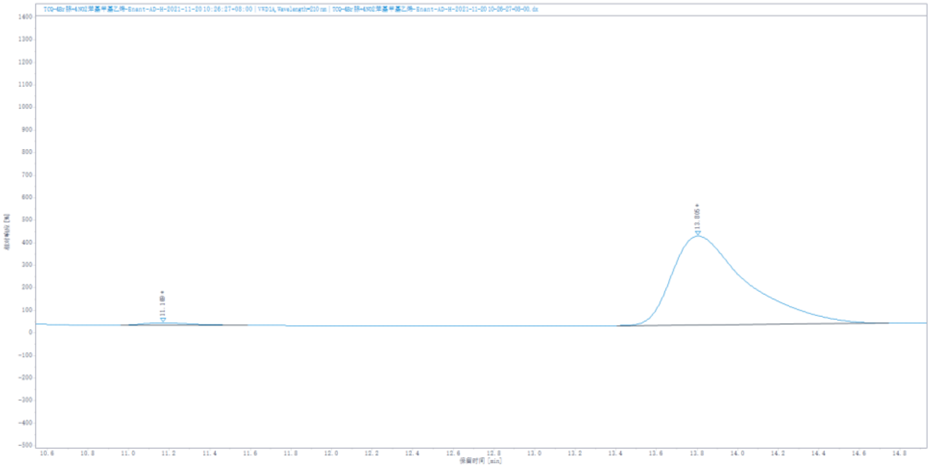

Figure S17. HPLC traces of compound 19

RetTime	Area	Area%	Hight	Hight%
11.169	316.26	1.79	20.27	2.90
13.805	17361.26	98.21	679.20	97.10

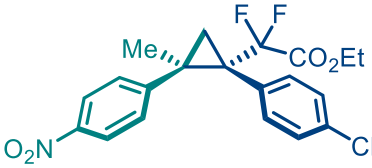

Chiralcel OD-H, 2 % *i*Pr-OH in hexane, 1 mL/min λ= 210 nm, tR= 8.237 min, minor; tR= 9.683 min, major.

Racemate:

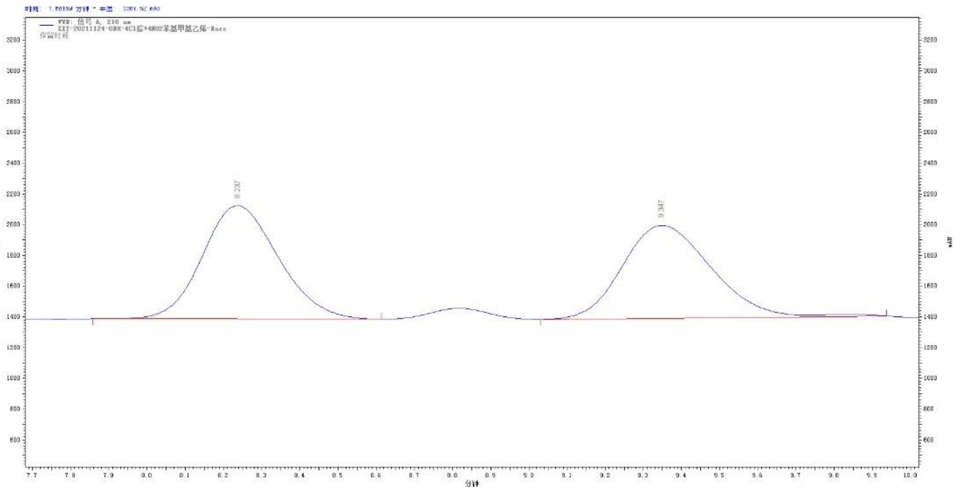

Enantiomer:

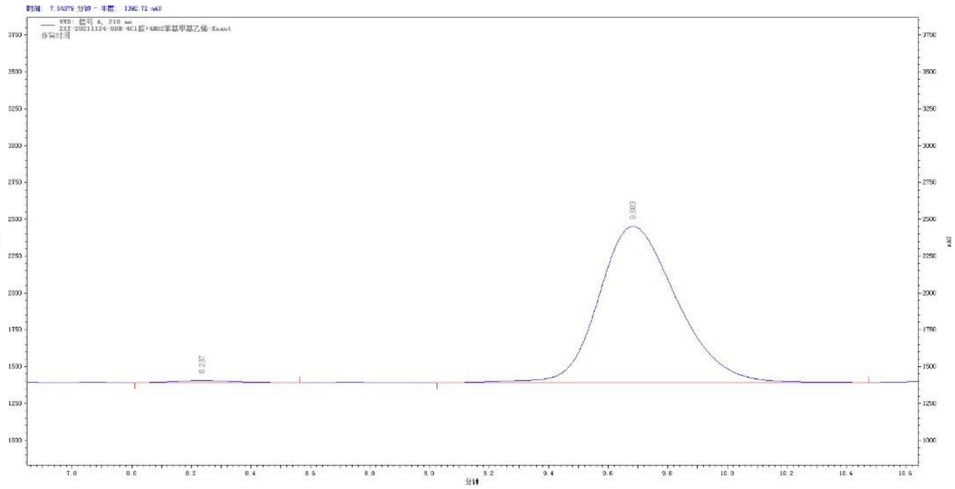

Figure S18. HPLC traces of compound 20

RetTime	Area	Area%	Hight	Hight%

8.237	3057200	0.93	231399	1.28
9.683	324630222	99.07	17796170	98.72

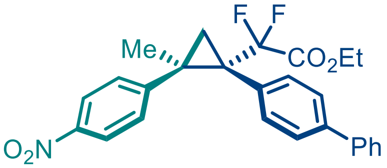

Chiralcel OD-H, 2 % *i*Pr-OH in hexane, 1 mL/min λ= 210 nm, tR= 12.550 min, major; tR= 14.142 min, minor.

Racemate:

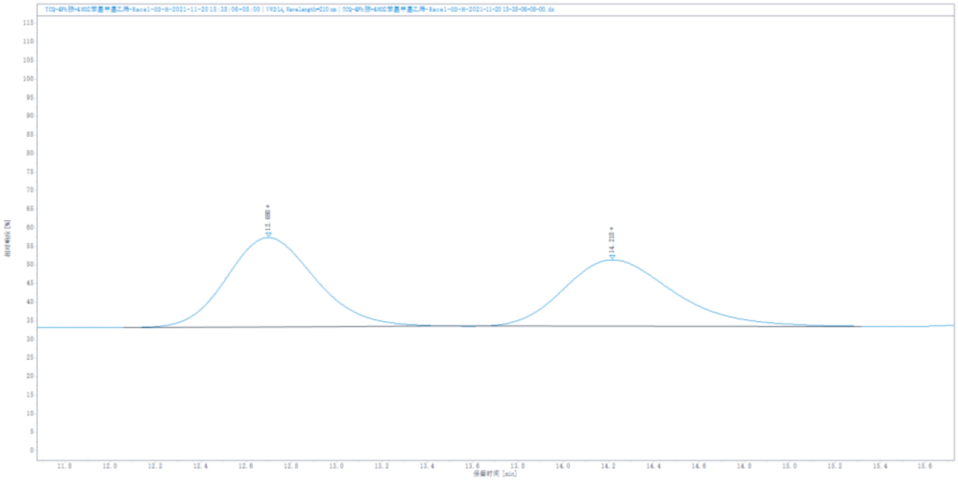

Enantiomer:

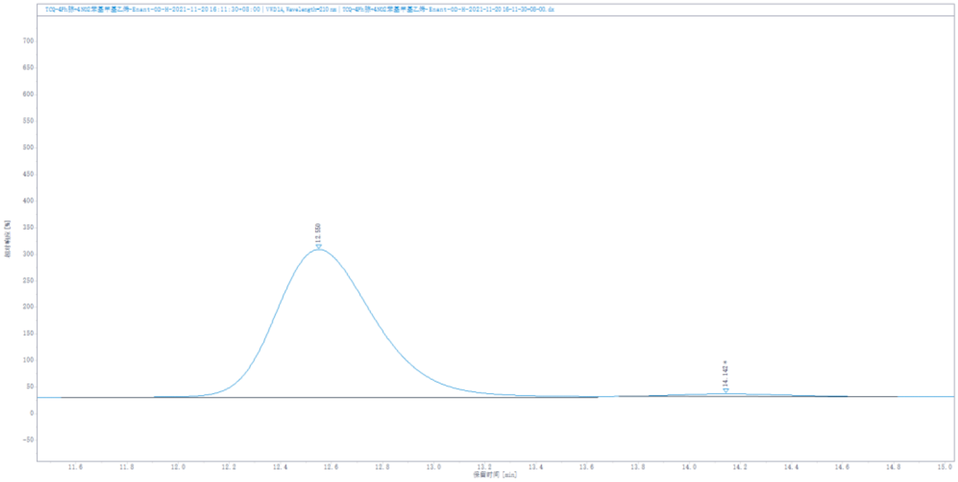

Figure S19. HPLC traces of compound 21

RetTime	Area	Area%	Hight	Hight%
12.550	13563.29	97.98	497.55	98.17
14.142	279.25	2.02	9.25	1.83

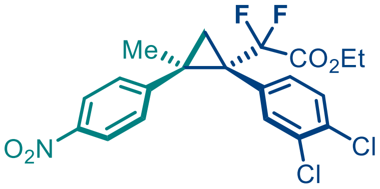

Chiralcel OD-H, 2 % *i*Pr-OH in hexane, 1 mL/min λ= 210 nm, tR= 11.720 min, major; tR= 13.242 min, minor.

Racemate:

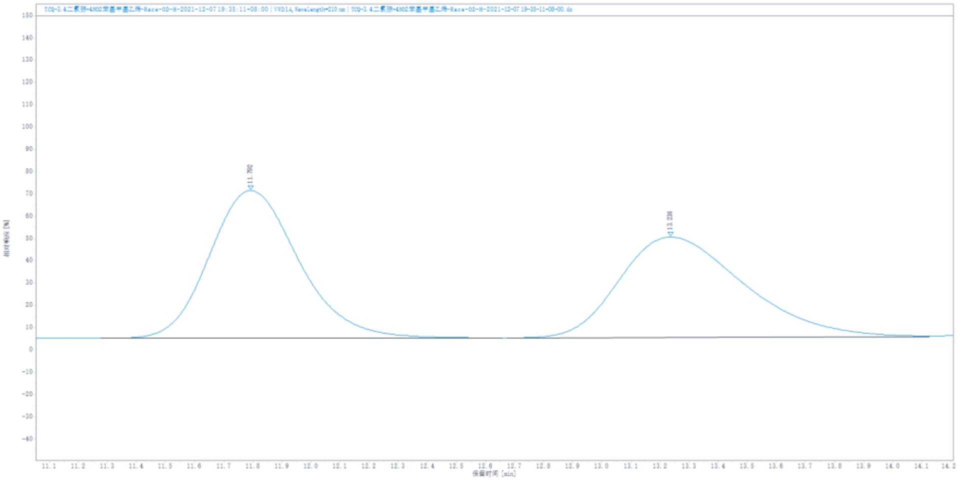

Enantiomer:

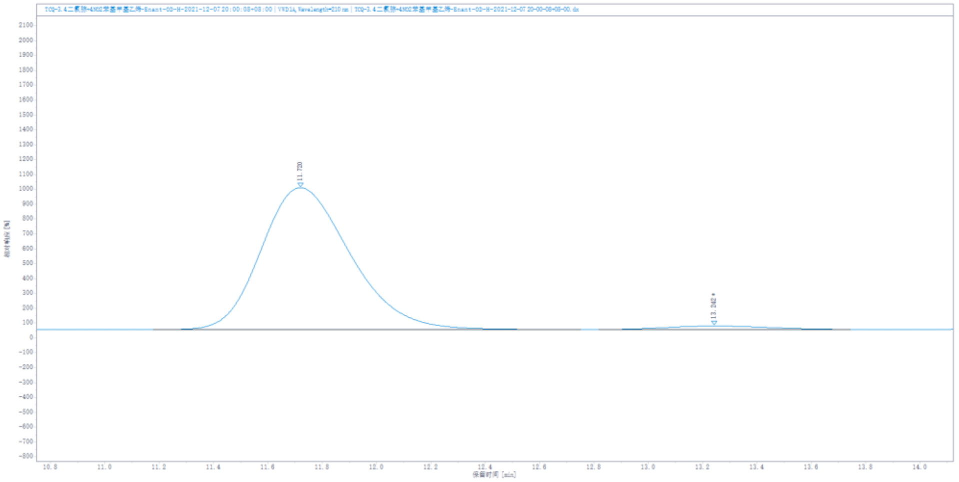

Figure S20. HPLC traces of compound 22

RetTime	Area	Area%	Hight	Hight%
11.720	15290.82	97.28	670.70	97.59
13.242	427.61	2.72	16.57	2.41

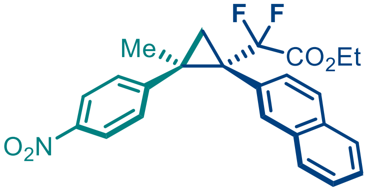

Chiralcel OD-H, 2 % *i*Pr-OH in hexane, 1 mL/min λ= 210 nm, tR= 9.543 min, major; tR= 10.333 min, minor.

Racemate:

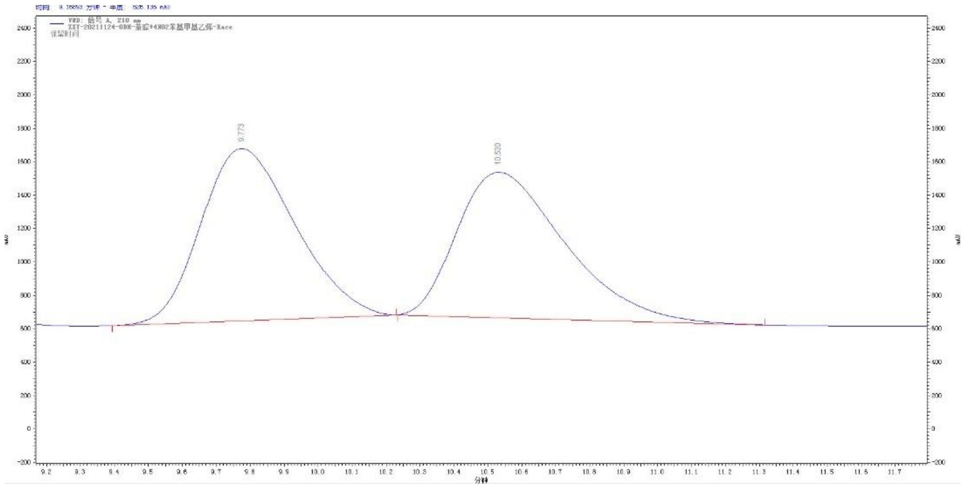

Enantiomer:

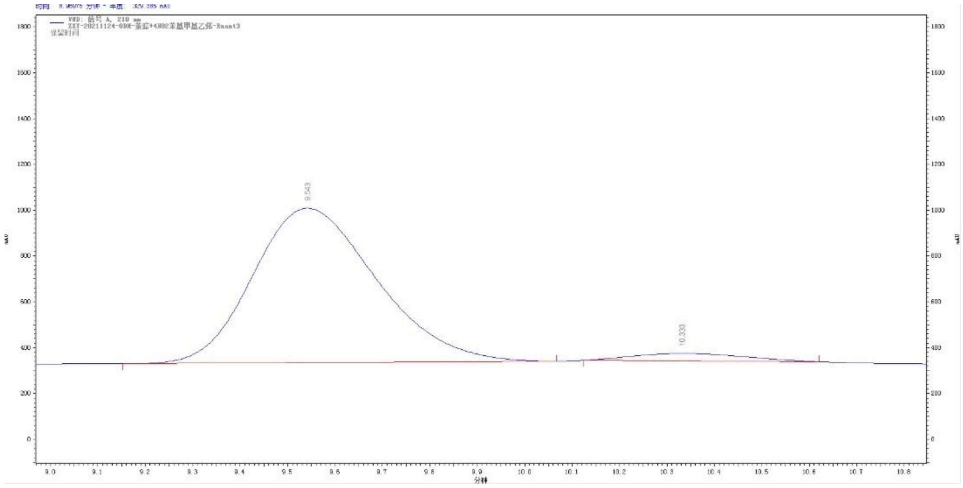

Figure S21. HPLC traces of compound 23

RetTime	Area	Area%	Hight	Hight%
9.543	204832715	95.81	11306452	95.41
10.333	8957706	4.19	543680	4.59

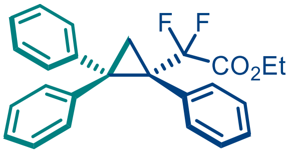

Chiralcel OD-H, 3 % *i*Pr-OH in hexane, 1 mL/min λ= 210 nm, tR= 6.200 min, major; tR= 8.540 min, minor.

Racemate:

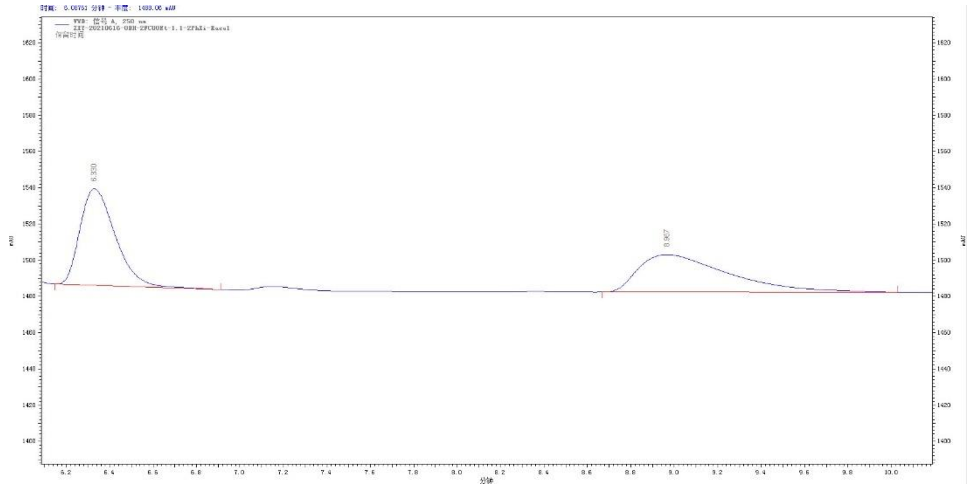

Enantiomer:

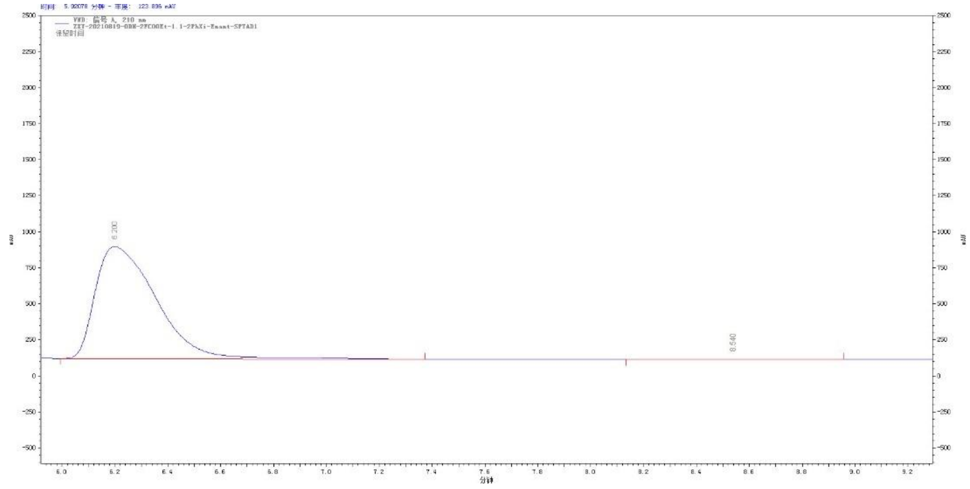

Figure S22. HPLC traces of compound 24

RetTime	Area	Area%	Hight	Hight%
6.200	207948993	99.62	13040304	99.80
8.540	784364	0.38	25829	0.20

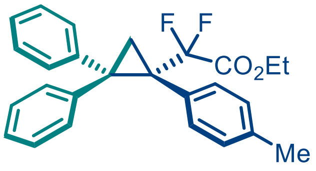

Chiralcel OZ-H, 1 % *i*Pr-OH in hexane, 1 mL/min λ= 210 nm, tR= 8.310 min, major; tR= 14.393 min, minor.

Racemate:

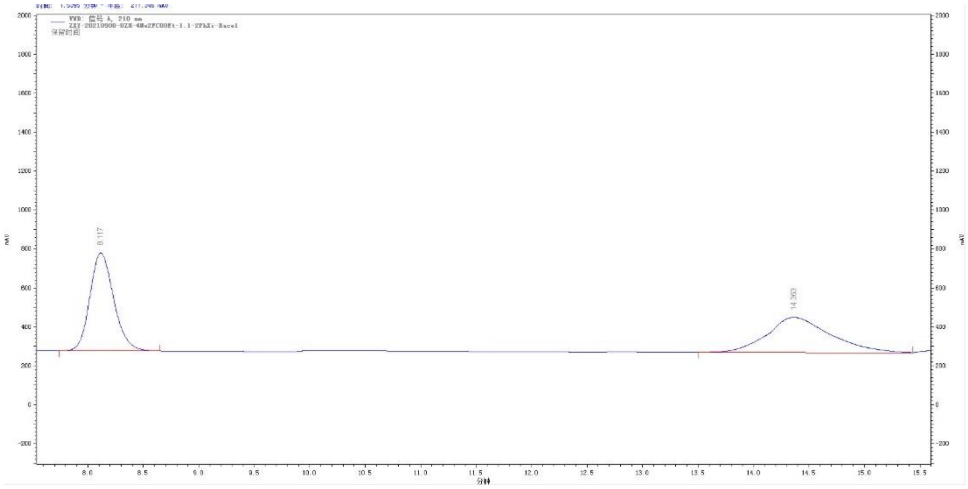

Enantiomer:

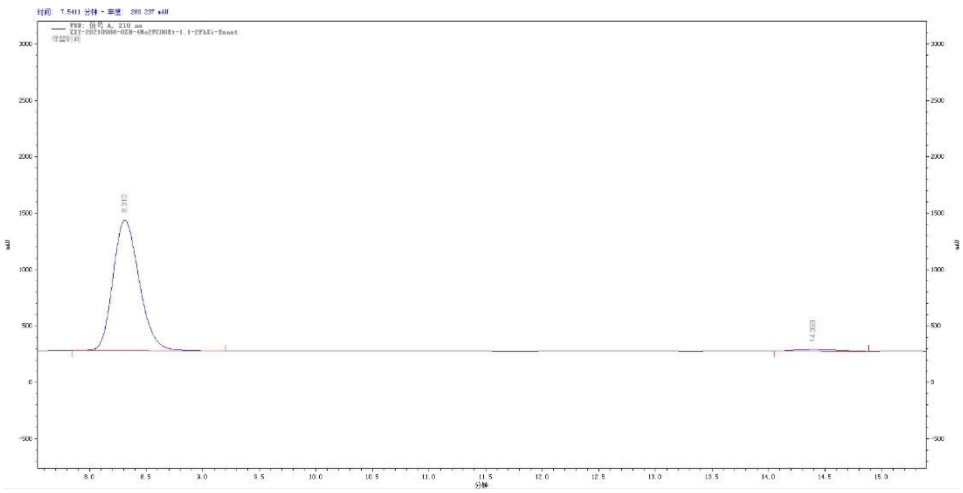

Figure S23. HPLC traces of compound 25

RetTime	Area	Area%	Hight	Hight%
8.310	315125225	97.93	19442947	98.77
14.393	6673616	2.07	242490	1.23

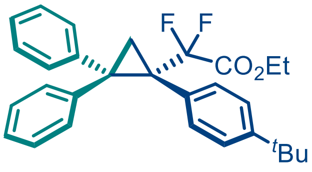

Chiralcel OZ-H, 1 % *i*Pr-OH in hexane, 1 mL/min λ= 210 nm, tR= 5.343 min, major; tR= 7.660 min, minor.

Racemate:

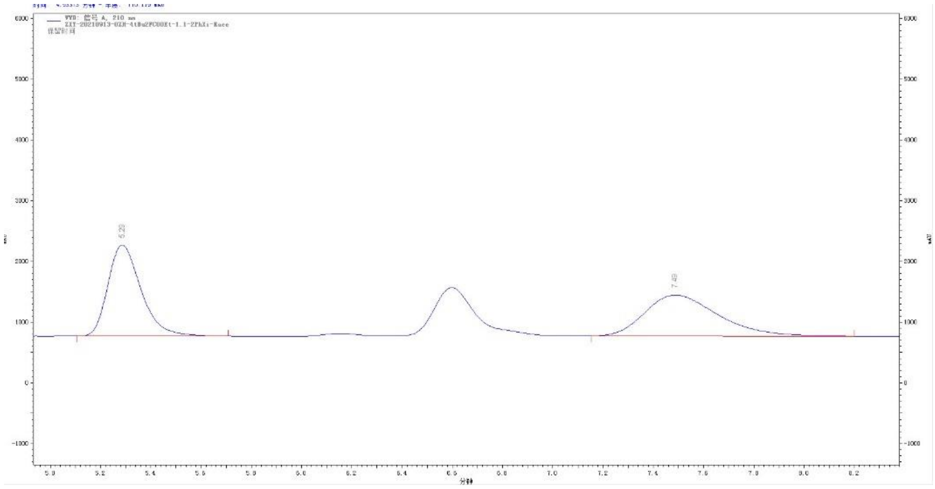

Enantiomer:

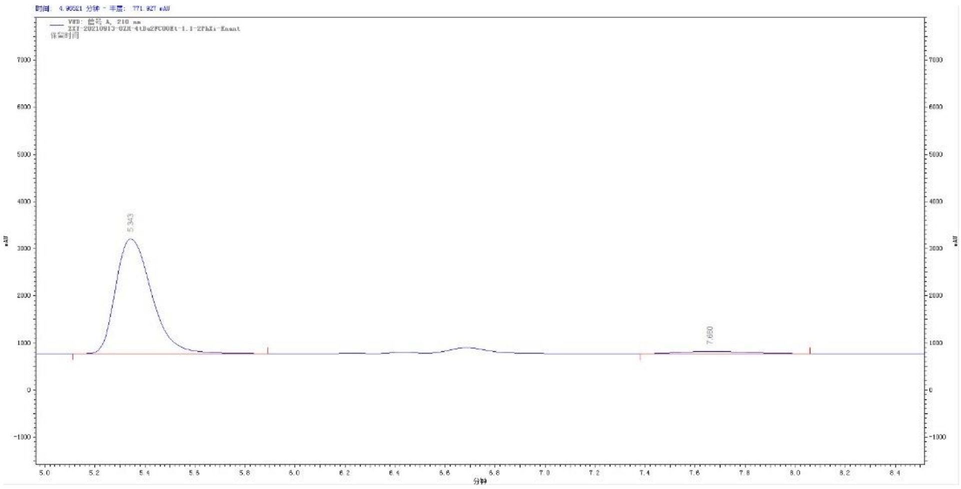

Figure S24. HPLC traces of compound 26

RetTime	Area	Area%	Hight	Hight%
5.343	406561150	96.43	40768224	98.09
7.660	15034852	3.57	793154	1.91

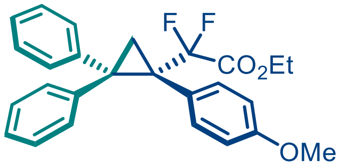

Chiralcel OD-H, 10 % *i*Pr-OH in hexane, 0.5 mL/min λ= 210 nm, tR= 11.417 min, major; tR= 13.810 min, minor.

Racemate:

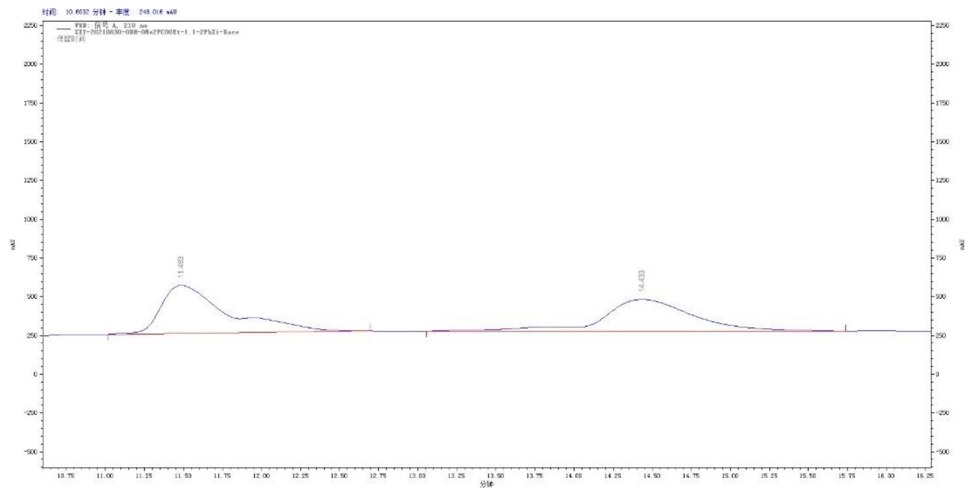

Enantiomer:

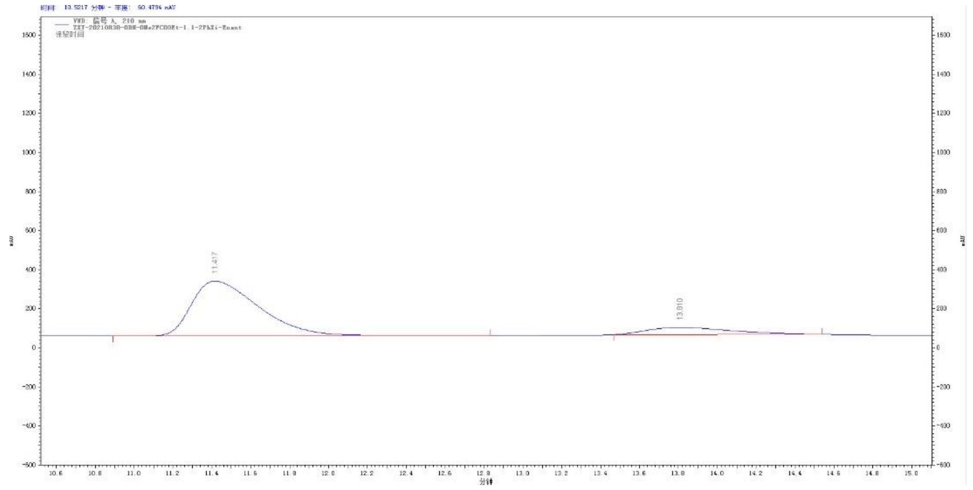

Figure S25. HPLC traces of compound 27

RetTime	Area	Area%	Hight	Hight%
11.417	112595733	85.96	4692823	88.52
13.810	18395882	14.04	608710	11.48

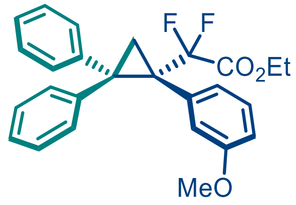

Chiralcel OZ-H, 5 % *i*Pr-OH in hexane, 1 mL/min λ= 210 nm, tR= 6.270 min, major; tR= 9.767 min, minor.

Racemate:

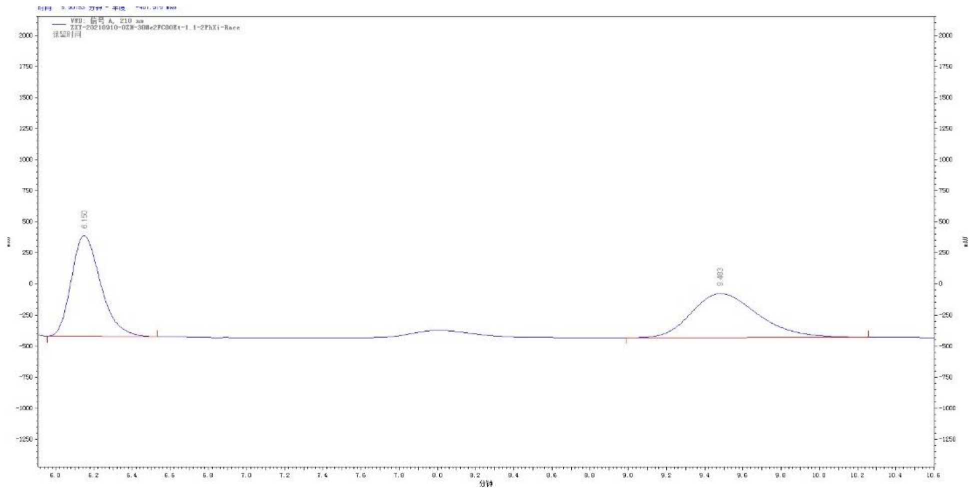

Enantiomer:

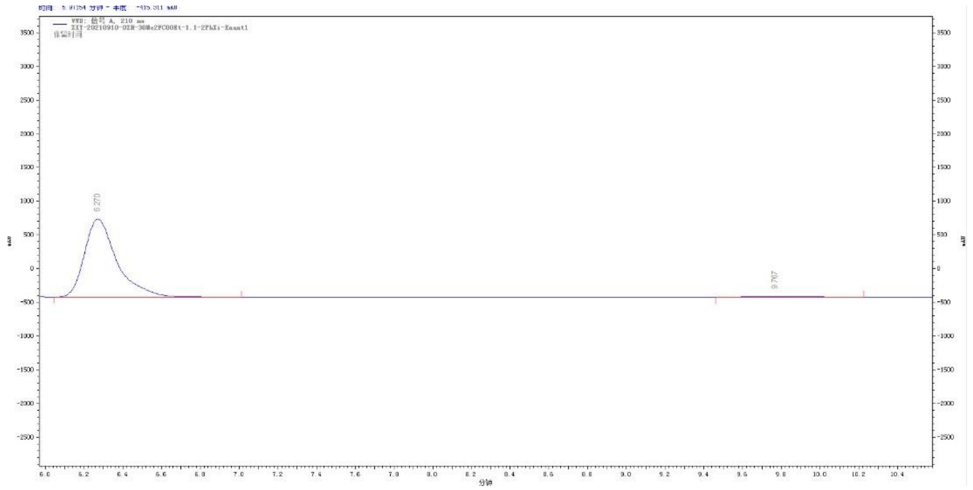

Figure S26. HPLC traces of compound 28

RetTime	Area	Area%	Hight	Hight%
6.270	226055901	98.12	19387874	99.02
9.767	4331746	1.88	190910	0.98

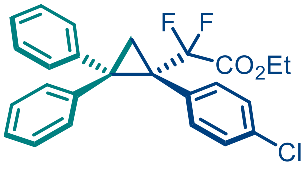

Chiralcel OZ-H, 1 % *i*Pr-OH in hexane, 1 mL/min λ= 210 nm, tR= 6.697 min, major; tR= 9.917 min, minor.

Racemate:

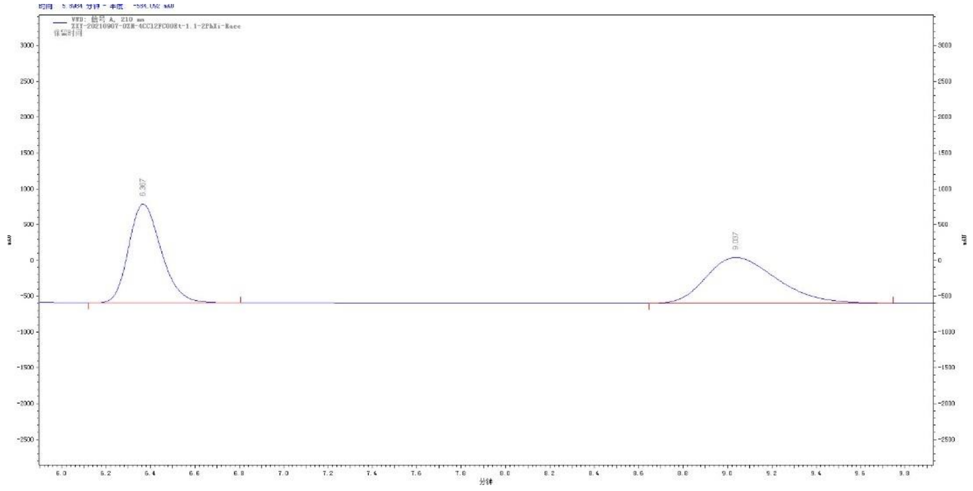

Enantiomer:

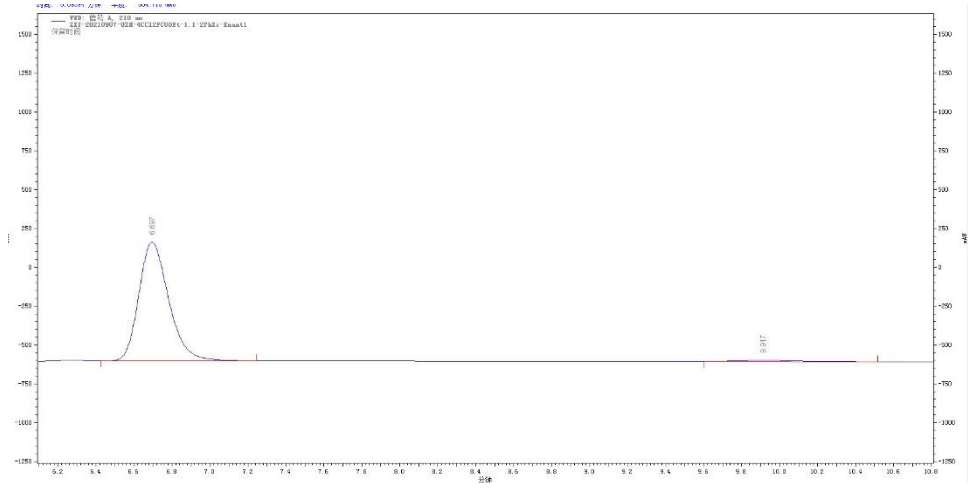

Figure S27. HPLC traces of compound 29

RetTime	Area	Area%	Hight	Hight%
6.697	139987458	97.90	12810501	98.99
9.917	2996617	2.10	130871	1.01

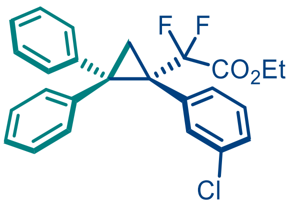

Chiralcel OZ-H, 5 % *i*Pr-OH in hexane, 1 mL/min λ= 210 nm, tR= 5.243 min, major; tR= 7.165 min, minor.

Racemate:

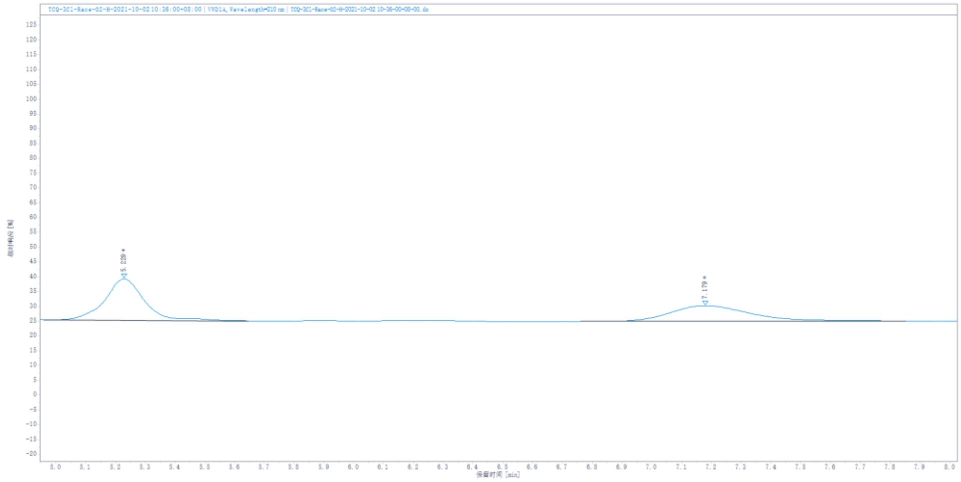

Enantiomer:

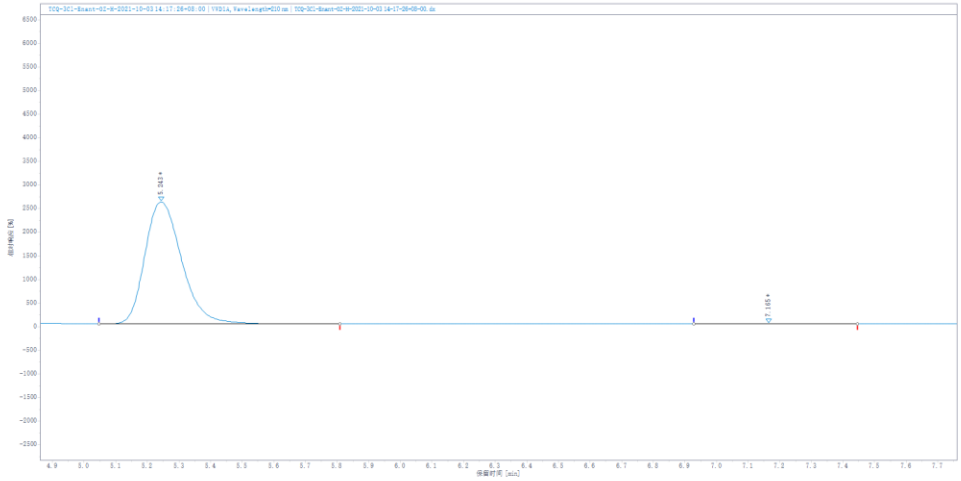

Figure S28. HPLC traces of compound 30

RetTime	Area	Area%	Hight	Hight%
5.243	24341.99	99.68	3002.34	99.82
7.165	77.93	0.32	5.40	0.18

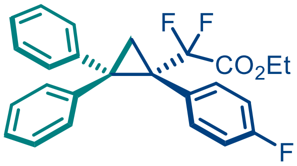

Chiralcel OZ-H, 1 % *i*Pr-OH in hexane, 1 mL/min λ= 210 nm, tR= 6.990 min, major; tR= 10.283 min, minor.

Racemate:

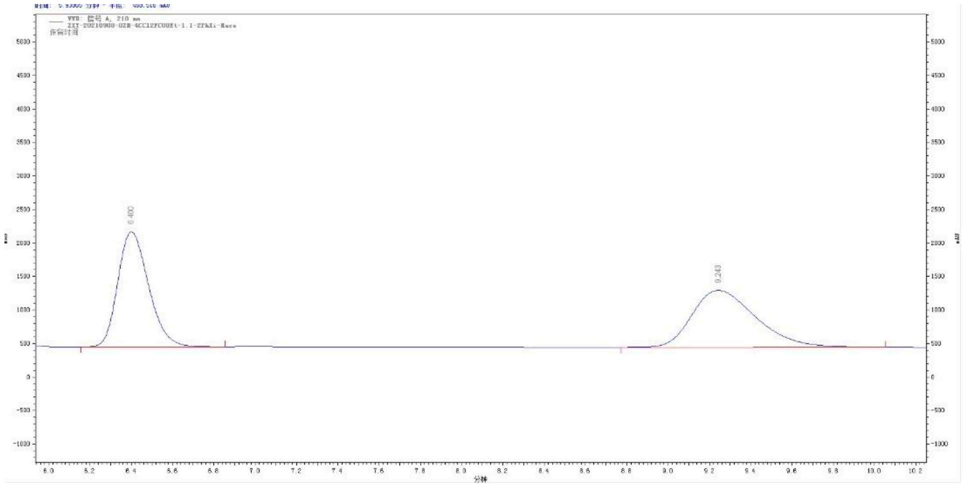

Enantiomer:

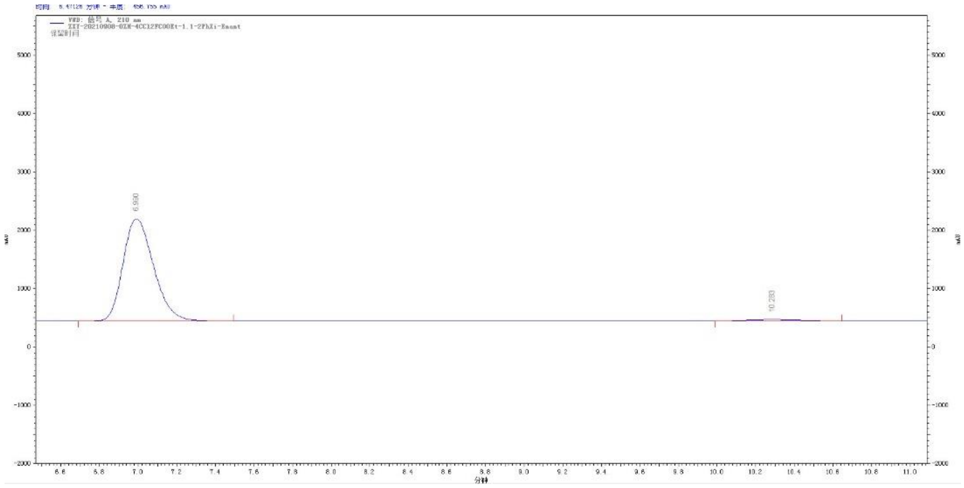

Figure S29. HPLC traces of compound 31

RetTime	Area	Area%	Hight	Hight%
6.990	323992744	97.43	29420316	98.54
10.283	8538349	2.57	436158	1.46

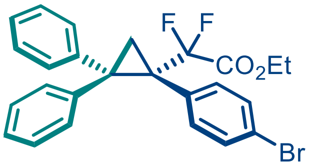

Chiralcel OZ-H, 1 % *i*Pr-OH in hexane, 1 mL/min λ= 210 nm, tR= 7.373 min, major; tR= 11.490 min, minor.

Racemate:

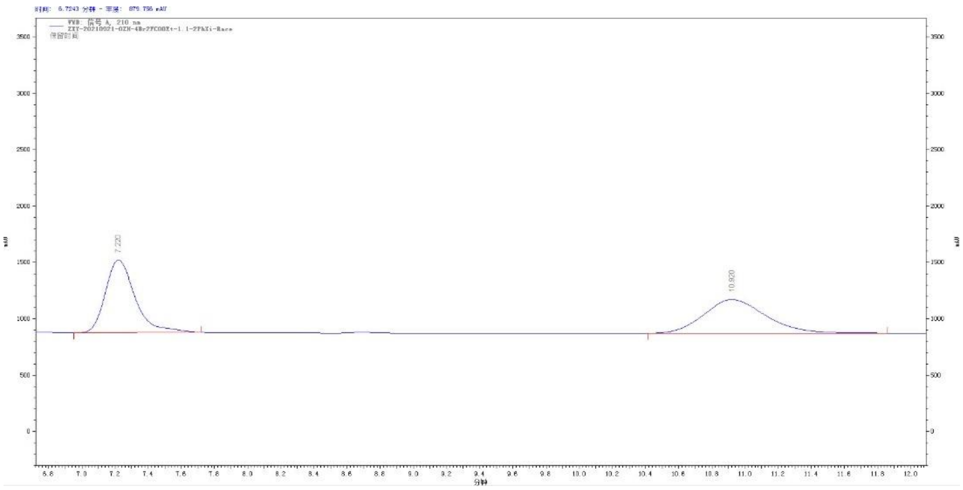

Enantiomer:

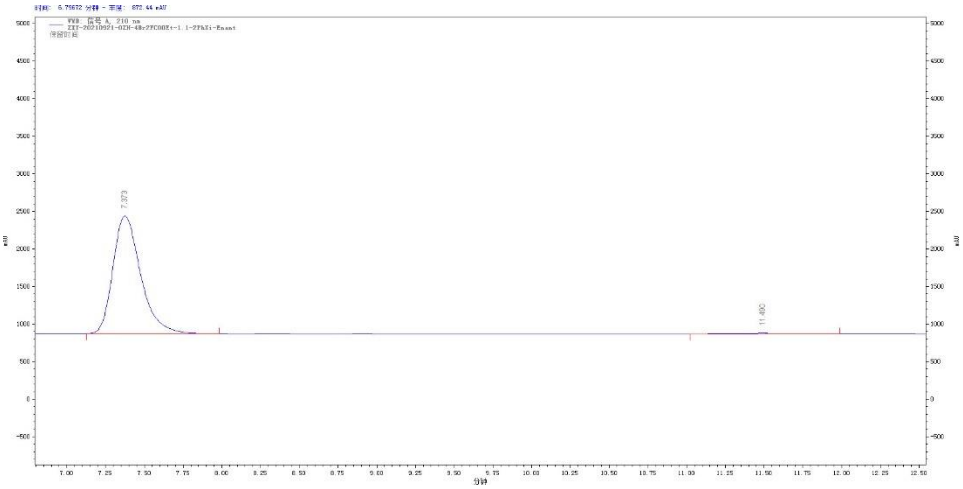

Figure S30. HPLC traces of compound 32

RetTime	Area	Area%	Hight	Hight%
7.373	326597481	98.72	26278514	99.47
11.490	4225623	1.28	139353	0.53

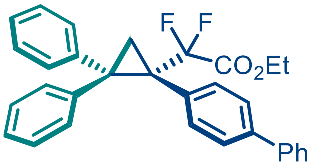

Chiralcel OZ-H, 5 % *i*Pr-OH in hexane, 1 mL/min λ= 210 nm, tR= 6.357 min, major; tR= 8.963 min, minor.

Racemate:

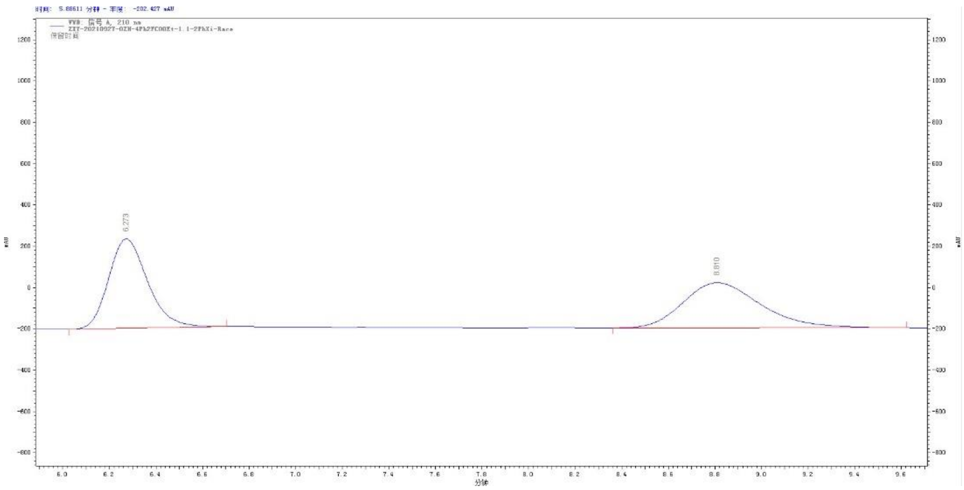

Enantiomer:

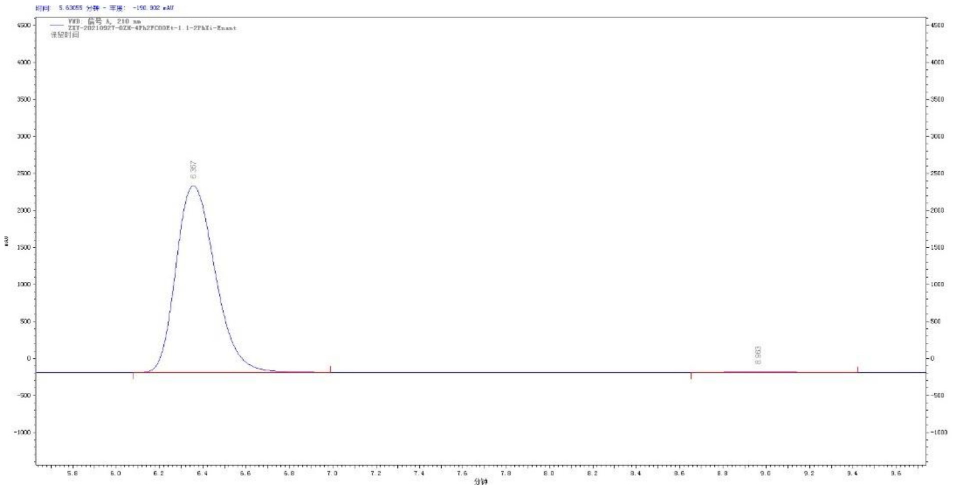

Figure S31. HPLC traces of compound 33

RetTime	Area	Area%	Hight	Hight%

6.357	532613583	99.23	42339982	99.54
8.963	4158954	0.77	194552	0.46

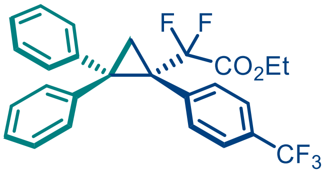

Chiralcel OZ-H, 2 % *i*Pr-OH in hexane, 1 mL/min λ= 210 nm, tR= 5.080 min, major; tR= 5.920 min, minor.

Racemate:

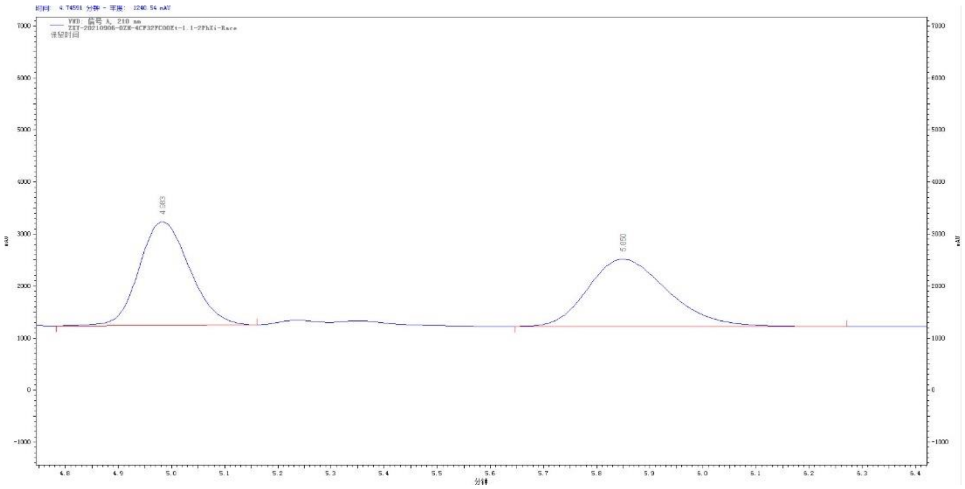

Enantiomer:

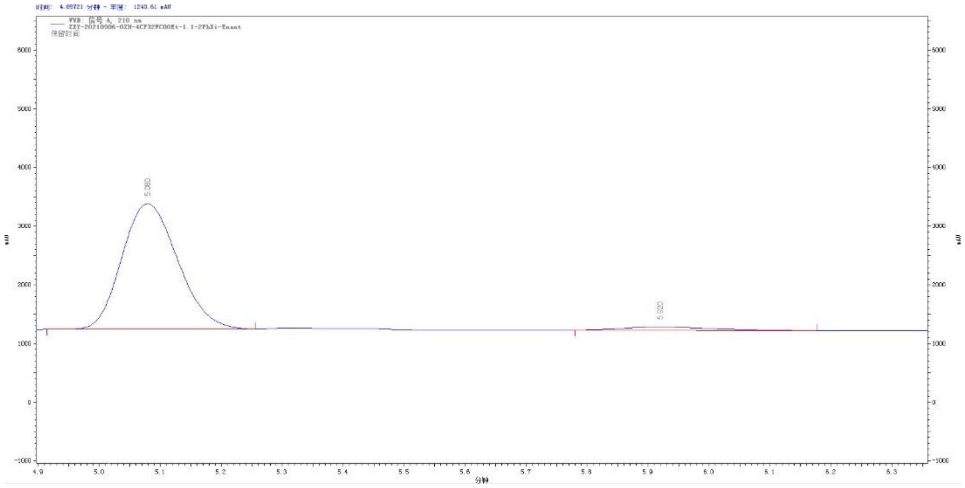

Figure S32. HPLC traces of compound 34

RetTime	Area	Area%	Hight	Hight%
5.080	228194277	95.76	35823807	97.31
5.920	10092830	4.24	991096	2.69

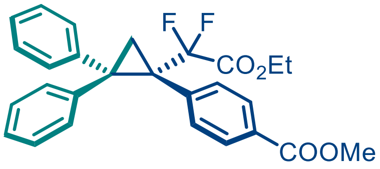

Chiralcel OZ-H, 10 % *i*Pr-OH in hexane, 1 mL/min λ= 210 nm, tR= 7.342 min, major; tR= 9.804 min, minor.

Racemate:

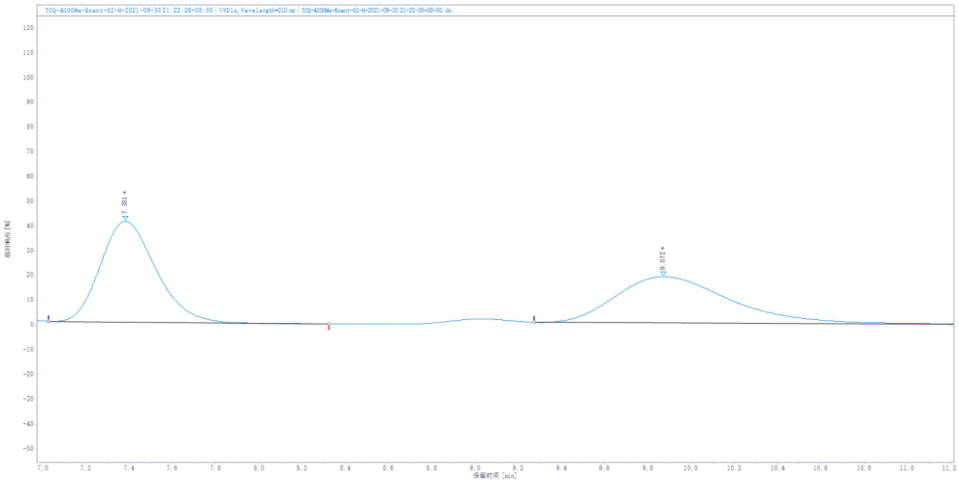

Enantiomer:

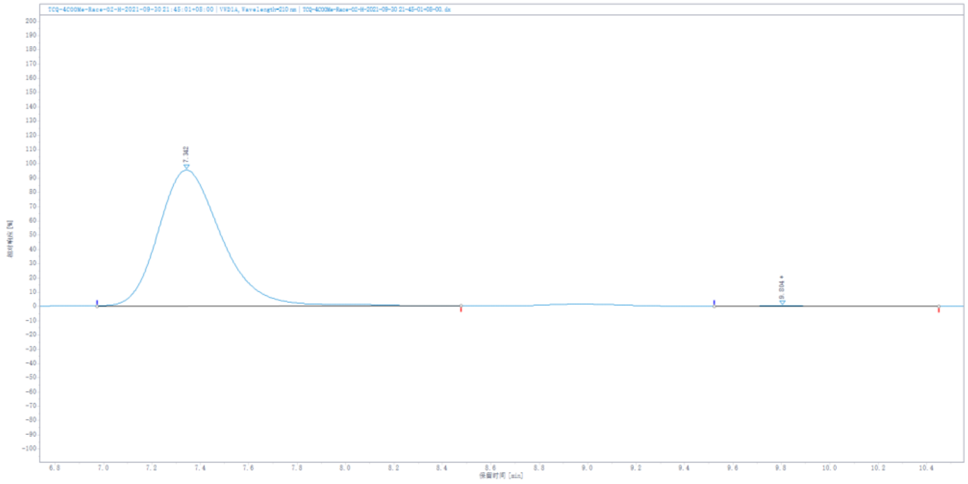

Figure S33. HPLC traces of compound 35

RetTime	Area	Area%	Hight	Hight%
7.342	4325.23	99.71	238.43	99.80
9.804	12.66	0.29	0.50	0.20

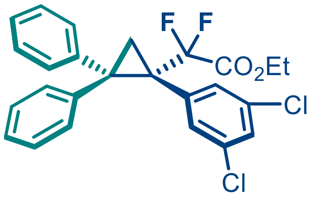

Chiralcel OZ-H, 5 % *i*Pr-OH in hexane, 1 mL/min λ= 210 nm, tR= 4.525 min, major; tR= 5.804 min, minor.

Racemate:

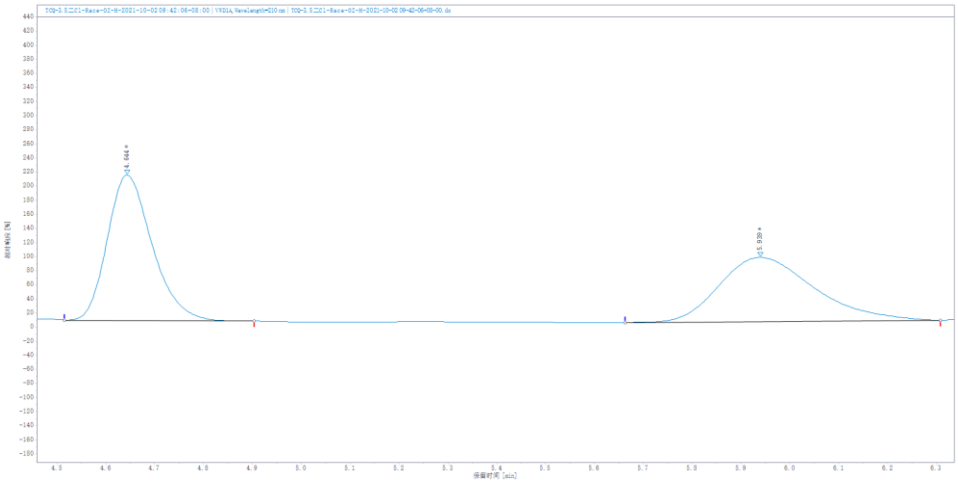

Enantiomer:

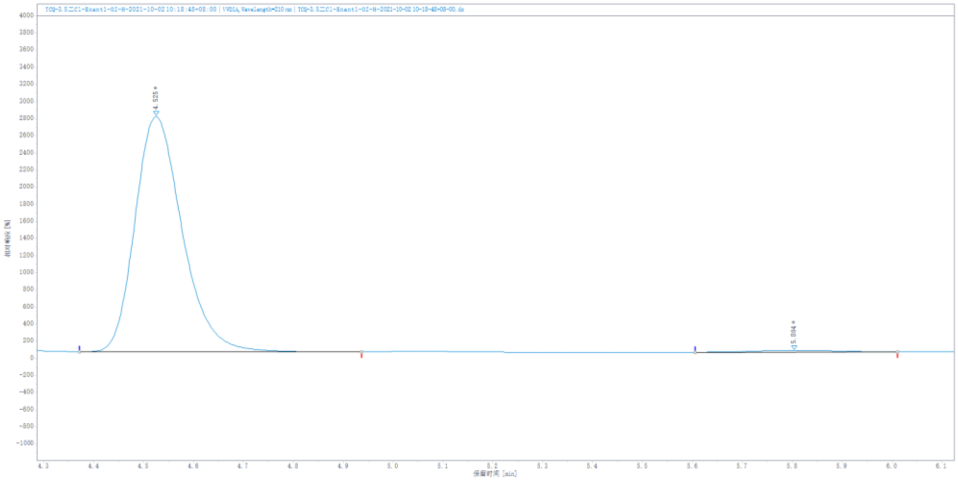

Figure S34. HPLC traces of compound 36

RetTime	Area	Area%	Hight	Hight%
4.525	18643.79	98.75	2839.51	99.29
5.804	235.08	1.25	20.40	0.71

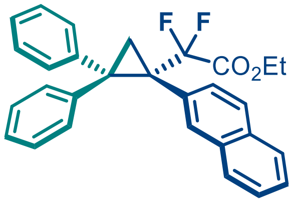

Chiralcel OZ-H, 3 % *i*Pr-OH in hexane, 1 mL/min λ= 210 nm, tR= 7.583 min, major; tR= 14.043 min, minor.

Racemate:

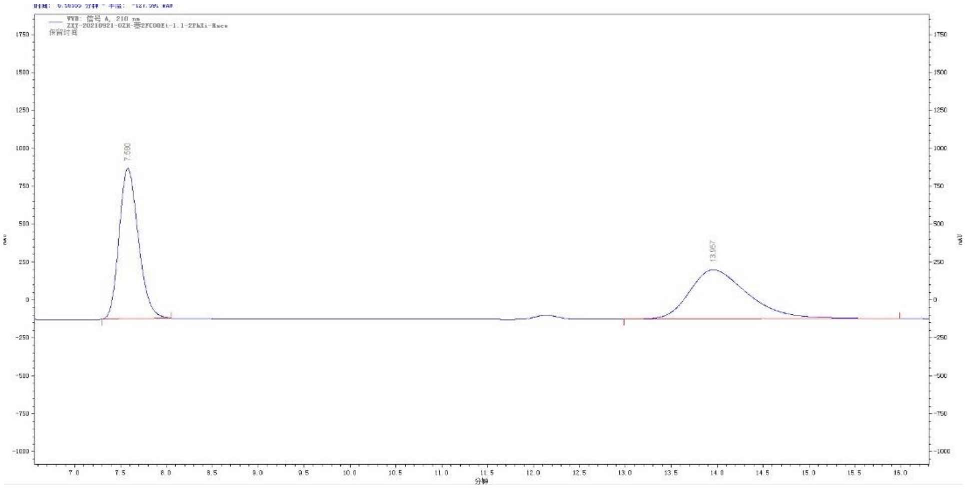

Enantiomer:

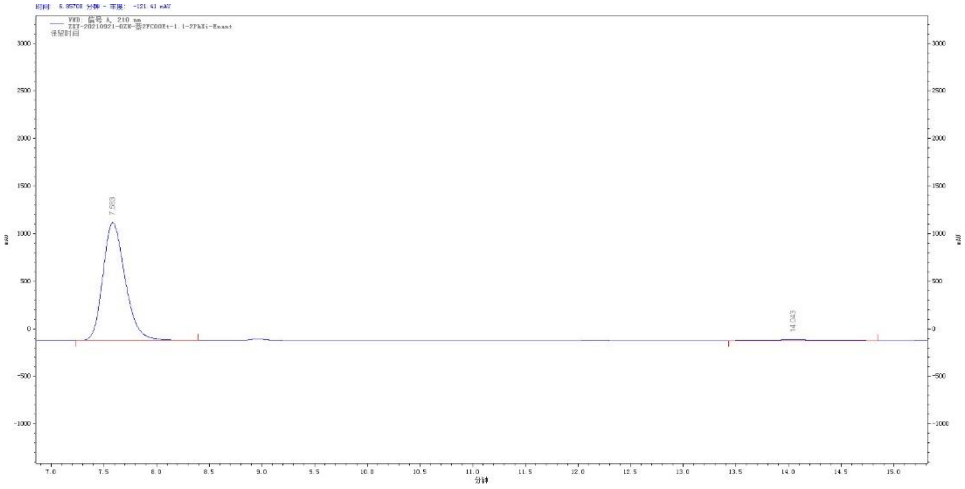

Figure S35. HPLC traces of compound 37

RetTime	Area	Area%	Hight	Hight%
7.583	303232865	98.43	20746562	99.43
14.043	4851364	1.57	118475	0.57

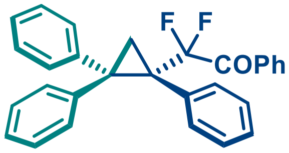

Chiralcel AD-H, 2 % *i*Pr-OH in hexane, 1 mL/min λ= 210 nm, tR= 6.430 min, major; tR= 7.640 min, minor.

Racemate:

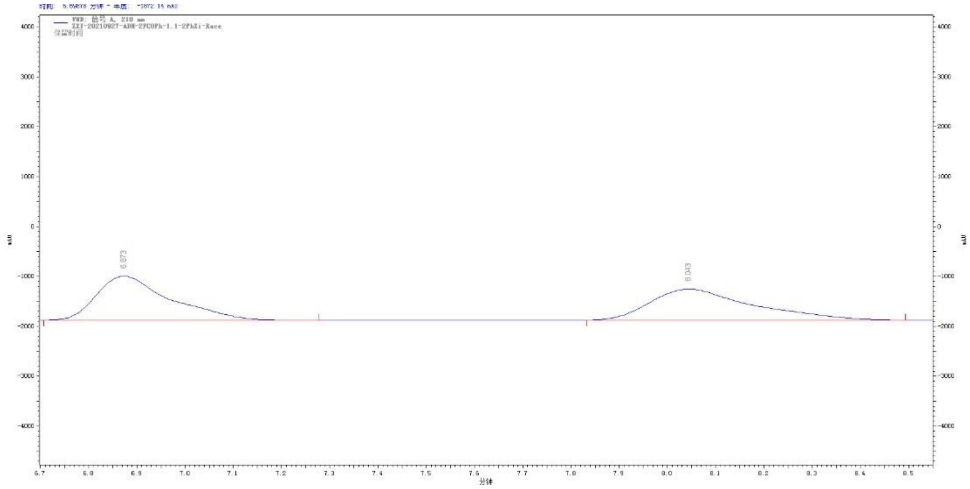

Enantiomer:

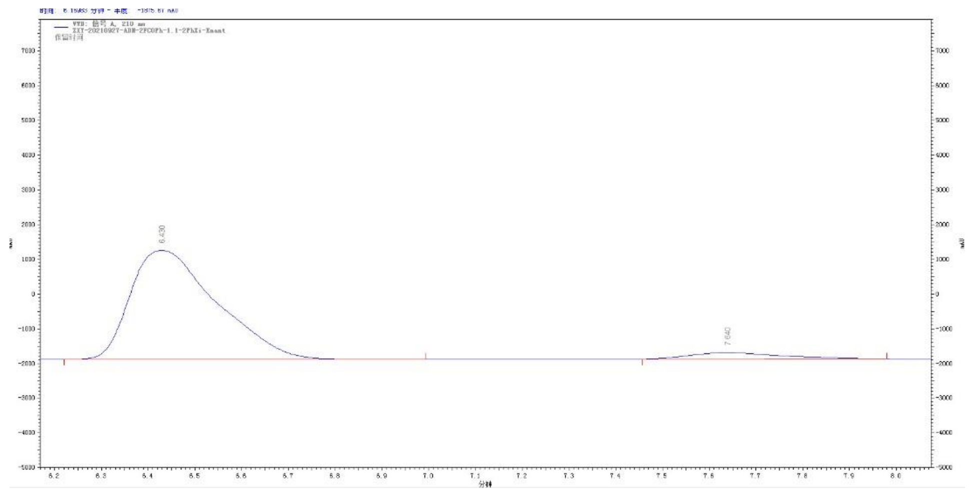

Figure S36. HPLC traces of compound 38

RetTime	Area	Area%	Hight	Hight%
6.430	668585089	94.09	52492981	94.50
7.640	41969473	5.91	3057643	5.50

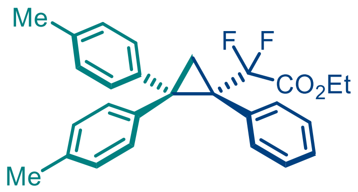

Chiralcel OD-H, 1 % *i*Pr-OH in hexane, 0.5 mL/min λ= 210 nm, tR= 9.980 min, major; tR= 11.507 min, minor.

Racemate:

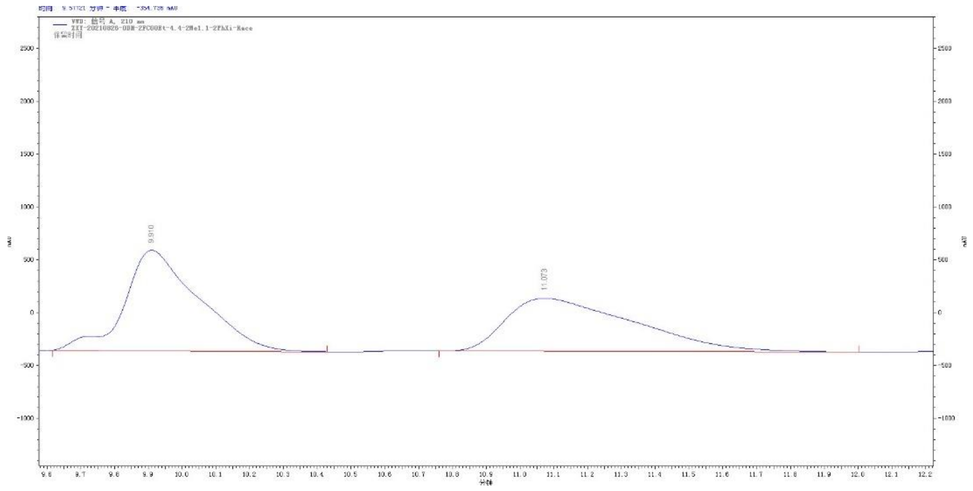

Enantiomer:

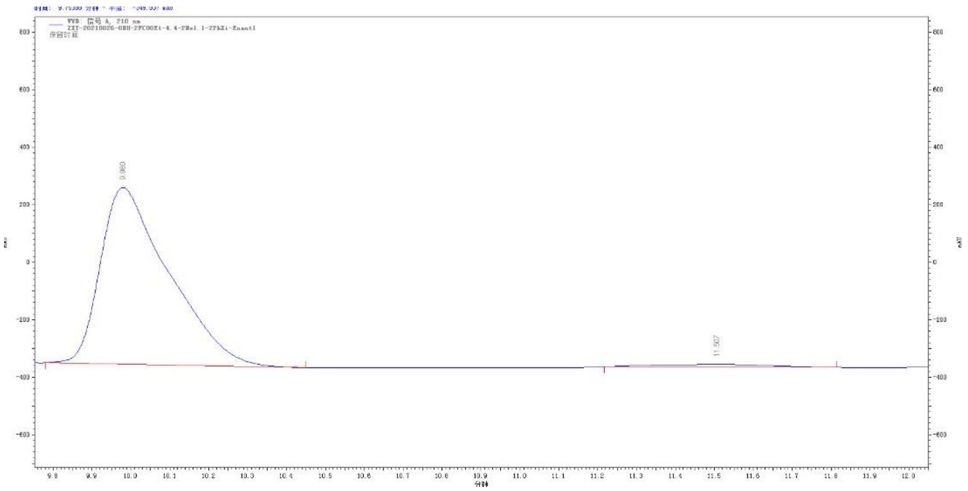

Figure S37. HPLC traces of compound 40

RetTime	Area	Area%	Hight	Hight%
9.980	125939504	97.55	10312572	98.56
11.507	3163831	2.45	150921	1.44

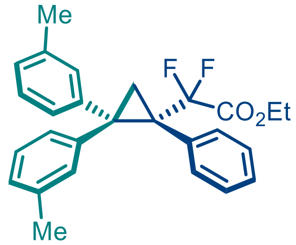

Chiralcel OD-H, 2 % *i*Pr-OH in hexane, 1 mL/min λ= 210 nm, tR= 6.117 min, major; tR= 6.894 min, minor.

Racemate:

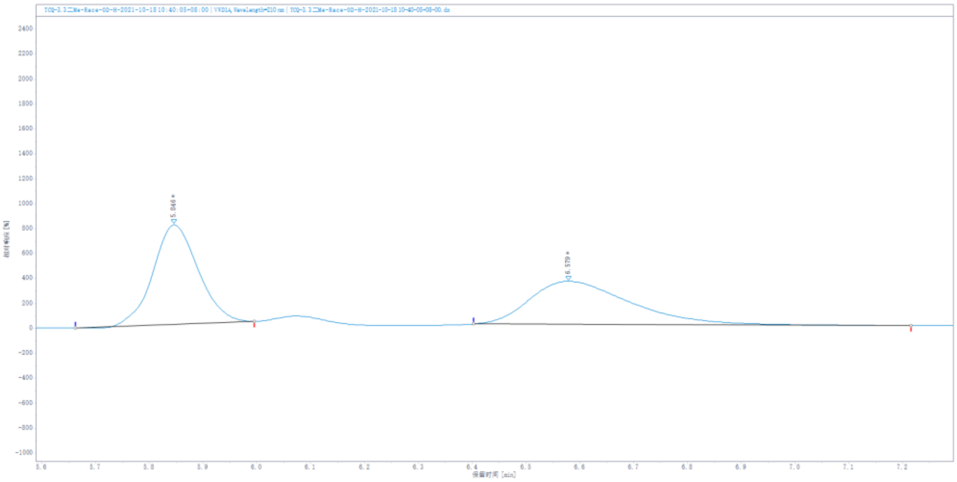

Enantiomer:

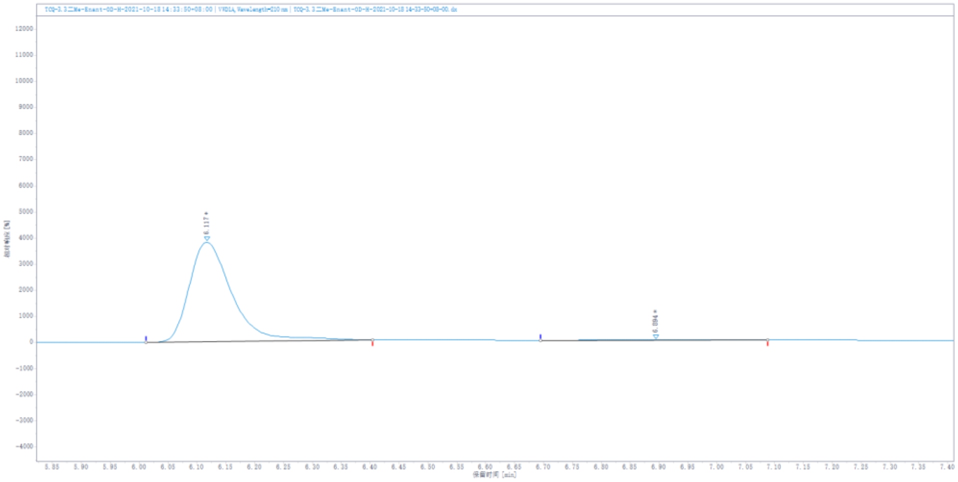

Figure S38. HPLC traces of compound 41

RetTime	Area	Area%	Hight	Hight%
6.117	11933.14	99.56	2327.63	99.80
6.894	52.96	0.44	4.64	0.20

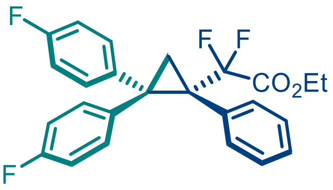

Chiralcel AD-H, 2 % *i*Pr-OH in hexane, 1 mL/min λ= 210 nm, tR= 7.643 min, major; tR= 9.223 min, minor.

Racemate:

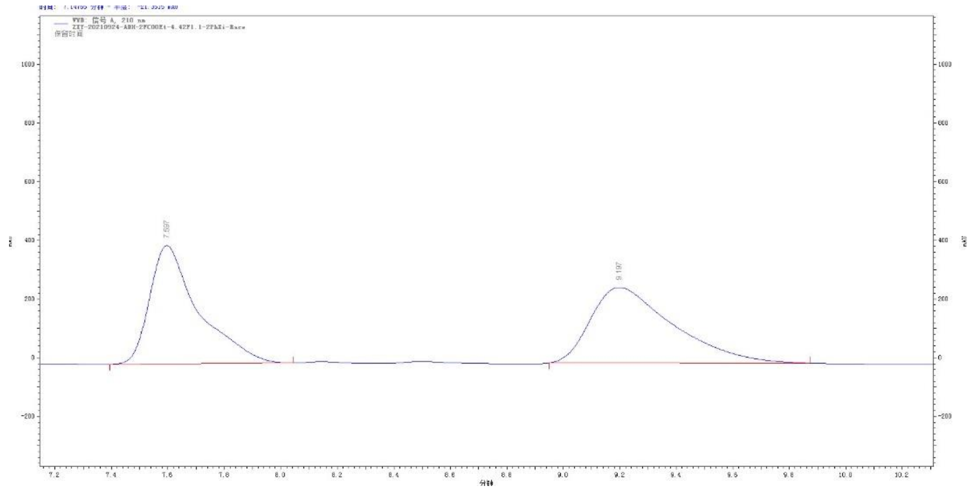

Enantiomer:

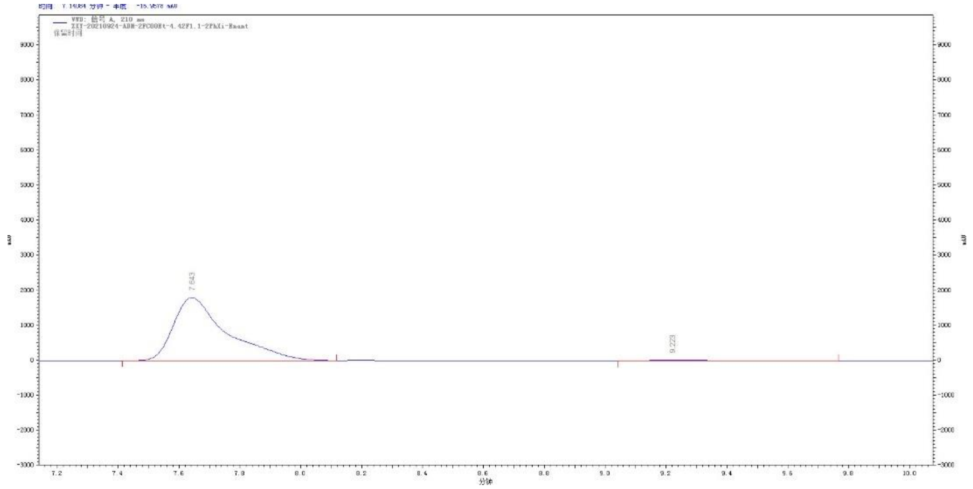

Figure S39. HPLC traces of compound 42

RetTime	Area	Area%	Hight	Hight%
7.643	365955544	99.16	30090892	99.41
9.223	3089508	0.84	178718	0.59

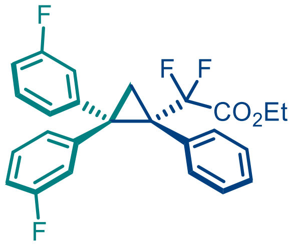

Chiralcel AD-H, 2 % *i*Pr-OH in hexane, 1 mL/min λ= 210 nm, tR= 8.165 min, major; tR= 9.228 min, minor.

Racemate:

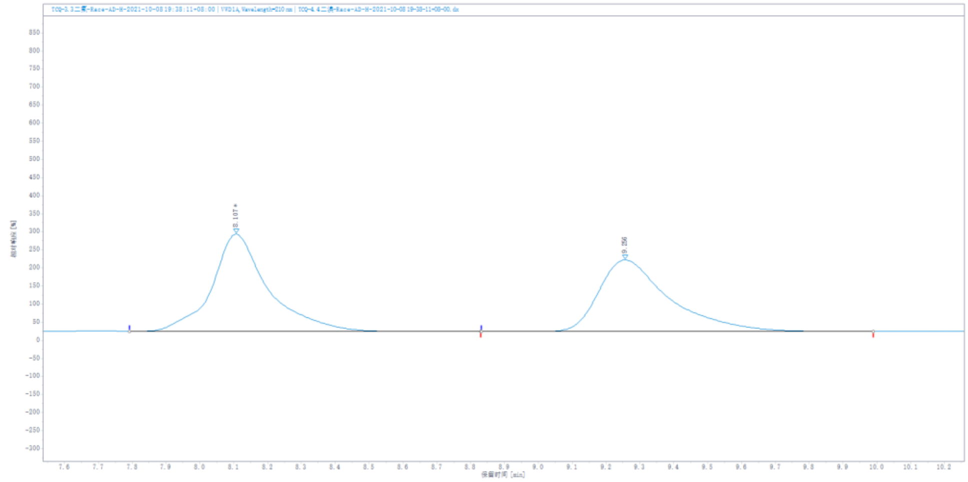

Enantiomer:

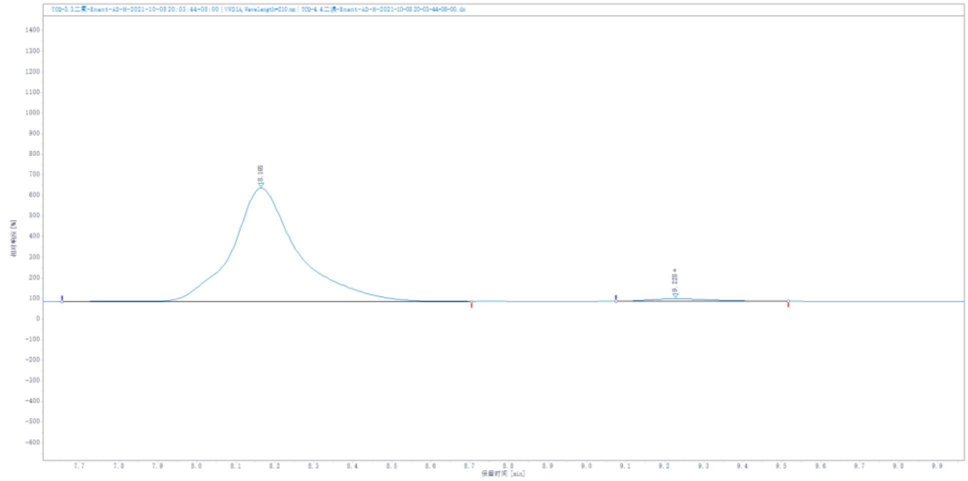

Figure S40. HPLC traces of compound 43

RetTime	Area	Area%	Hight	Hight%
8.165	3348.75	97.81	285.53	97.65
9.228	75.14	2.19	6.87	2.35

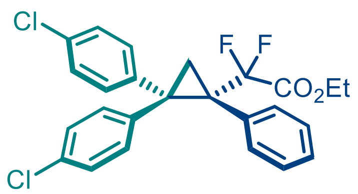

Chiralcel AD-H, 2 % *i*Pr-OH in hexane, 1 mL/min λ= 210 nm, tR= 8.557 min, major; tR= 10.787 min, minor.

Racemate:

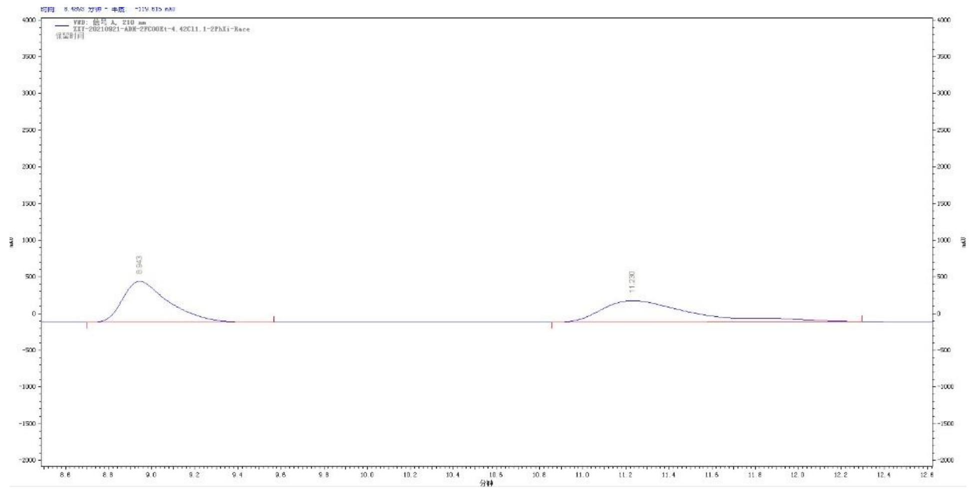

Enantiomer:

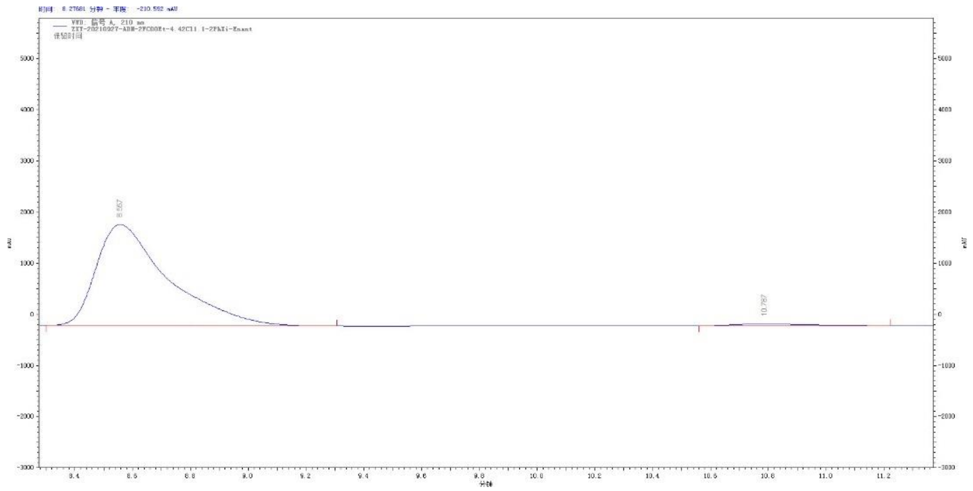

Figure S41. HPLC traces of compound 44

RetTime	Area	Area%	Hight	Hight%
8.557	567104188	98.12	33026510	98.25
10.787	10846608	1.88	589158	1.75

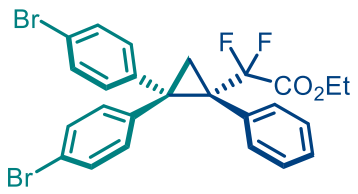

Chiralcel AD-H, 2 % *i*Pr-OH in hexane, 1 mL/min λ= 210 nm, tR= 13.167 min, major; tR= 17.774 min, minor.

Racemate:

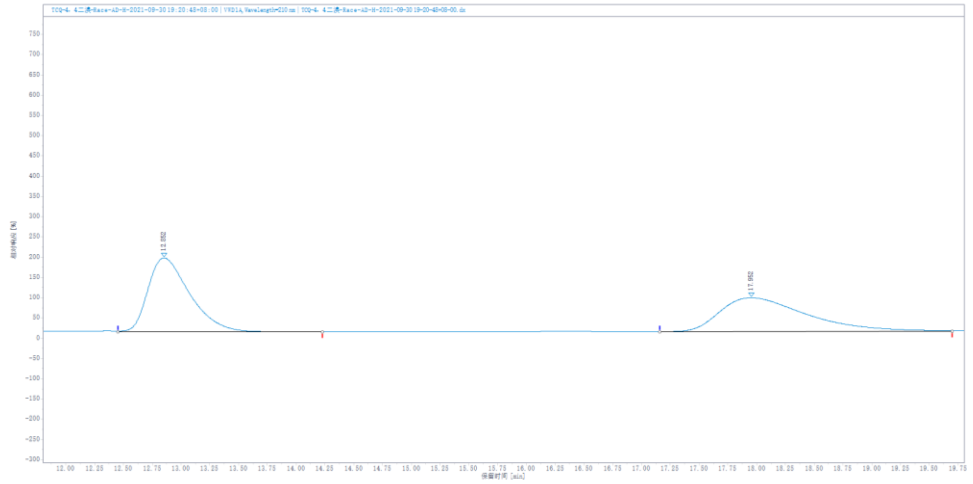

Enantiomer:

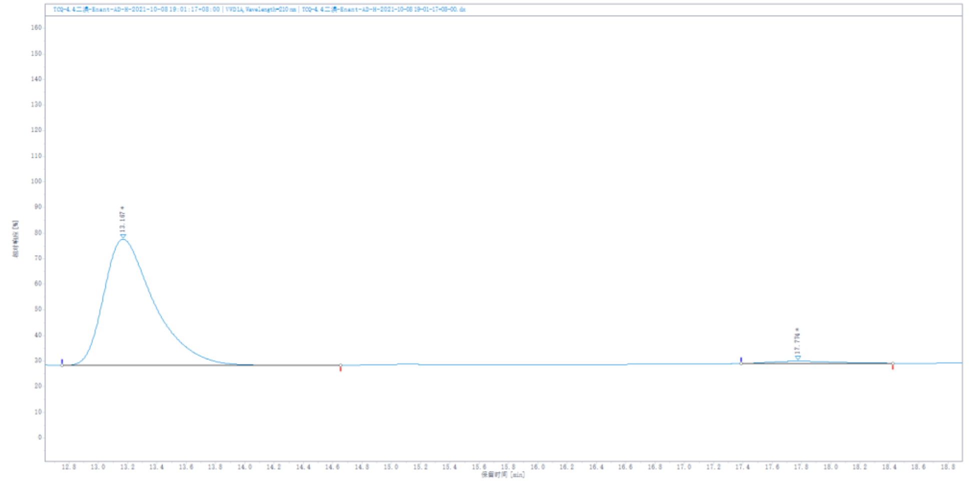

Figure S42. HPLC traces of compound 45

RetTime	Area	Area%	Hight	Hight%
13.167	1471.26	97.79	61.83	98.24
17.774	33.18	2.21	1.11	1.76

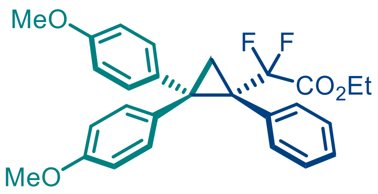

Chiralcel OD-H, 10 % *i*Pr-OH in hexane, 1 mL/min λ= 210 nm, tR= 7.413 min, major; tR= 9.793 min, minor.

Racemate:

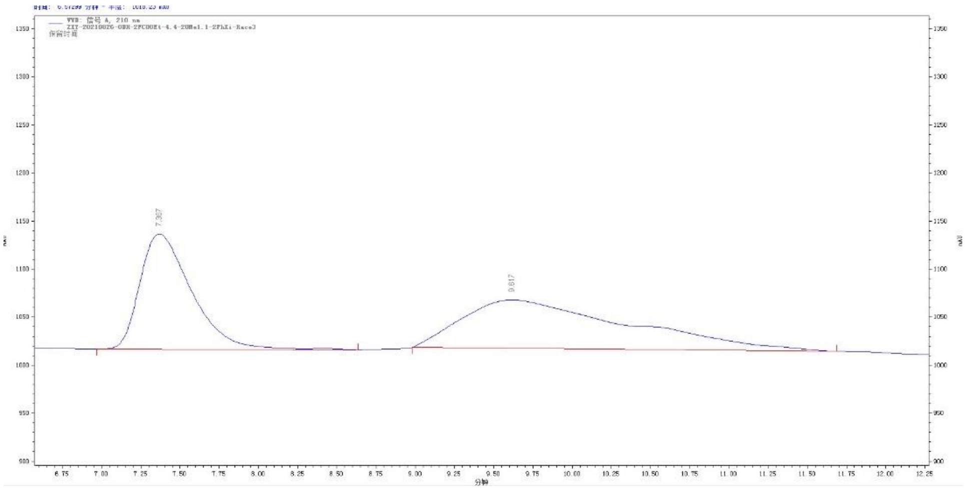

Enantiomer:

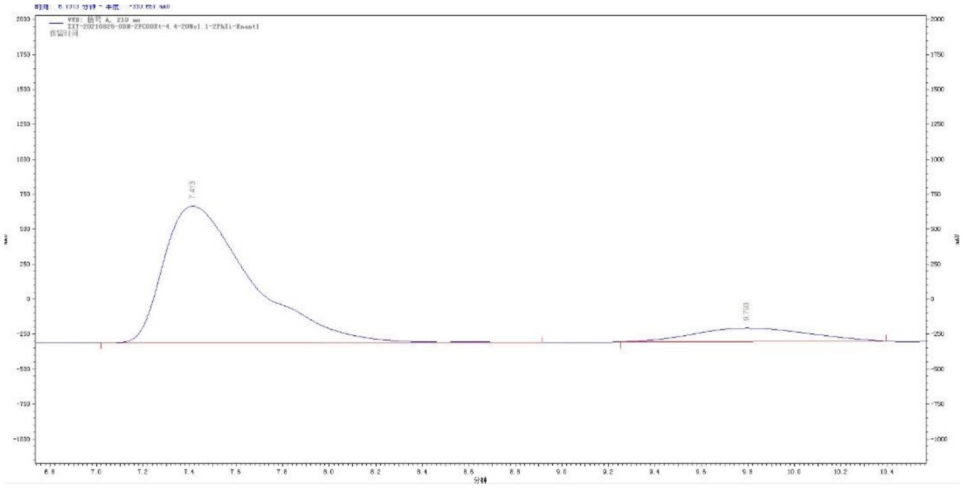

Figure S43. HPLC traces of compound 46

RetTime	Area	Area%	Hight	Hight%
7.413	426657681	88.67	16388379	91.07
9.793	54532347	11.33	1606340	8.93

Figure S44-S231, ^{1}H-NMR, ^{13}C-NMR and ^{19}F-NMR spectra copies of 1d related to scheme 2 and 3 related to scheme 2 and 3.

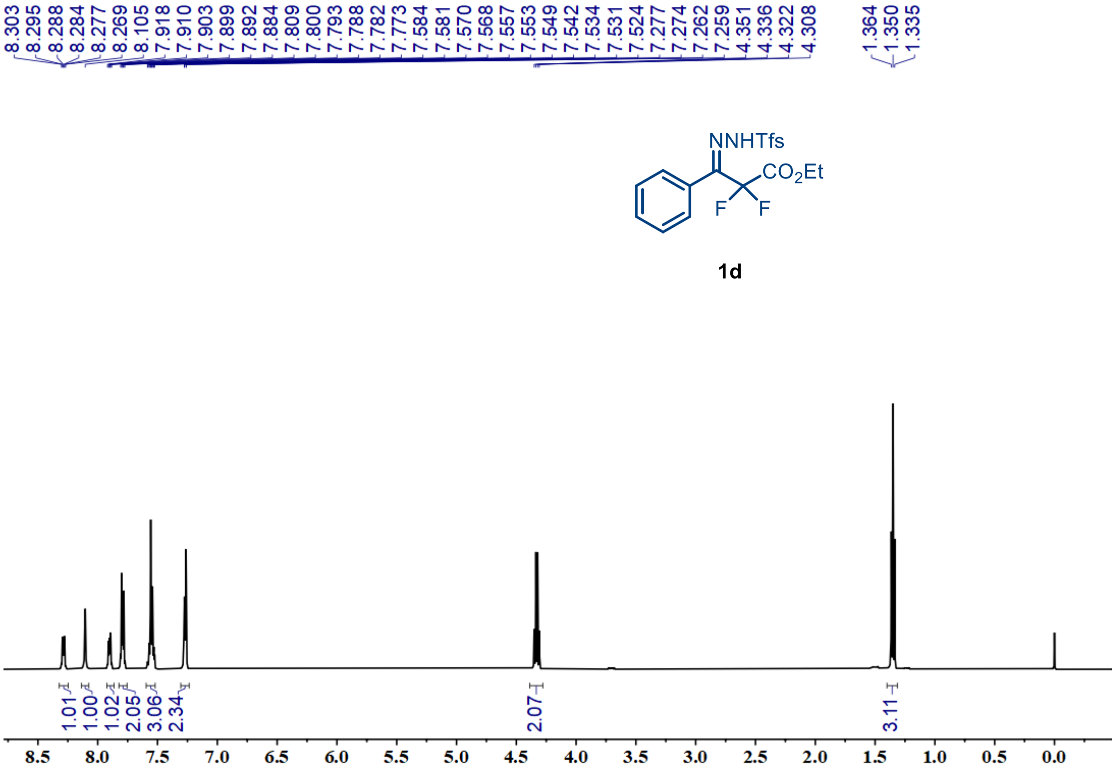

Figure S44, ^{1}H-NMR spectra copies of 1d related to scheme 2 and 3.

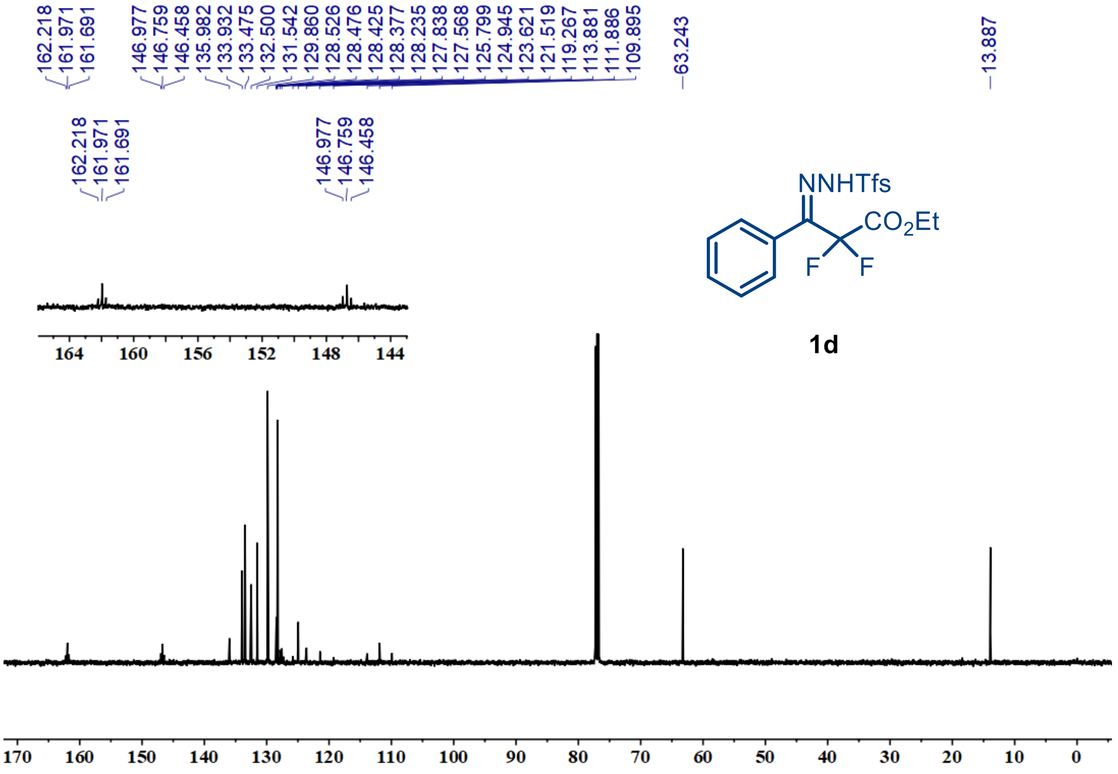

Figure S45, ^{13}C-NMR spectra copies of 1d related to scheme 2 and 3.

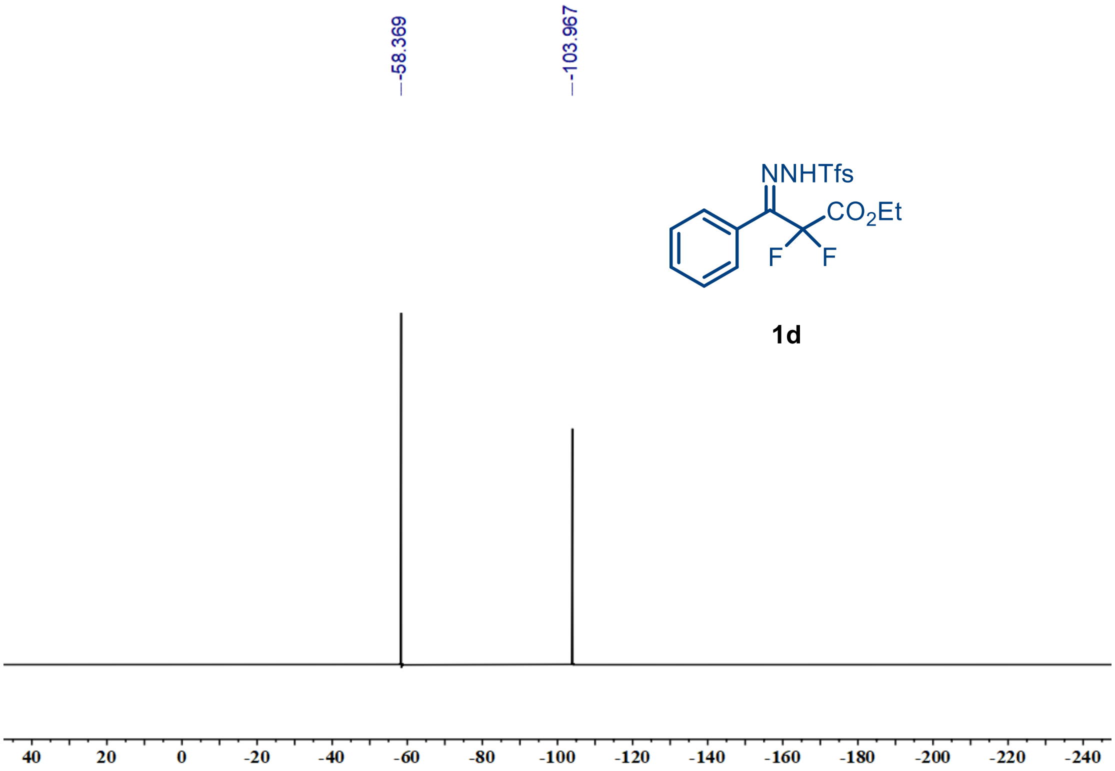

Figure S45, ^{19}F-NMR spectra copies of 1d related to scheme 2 and 3.

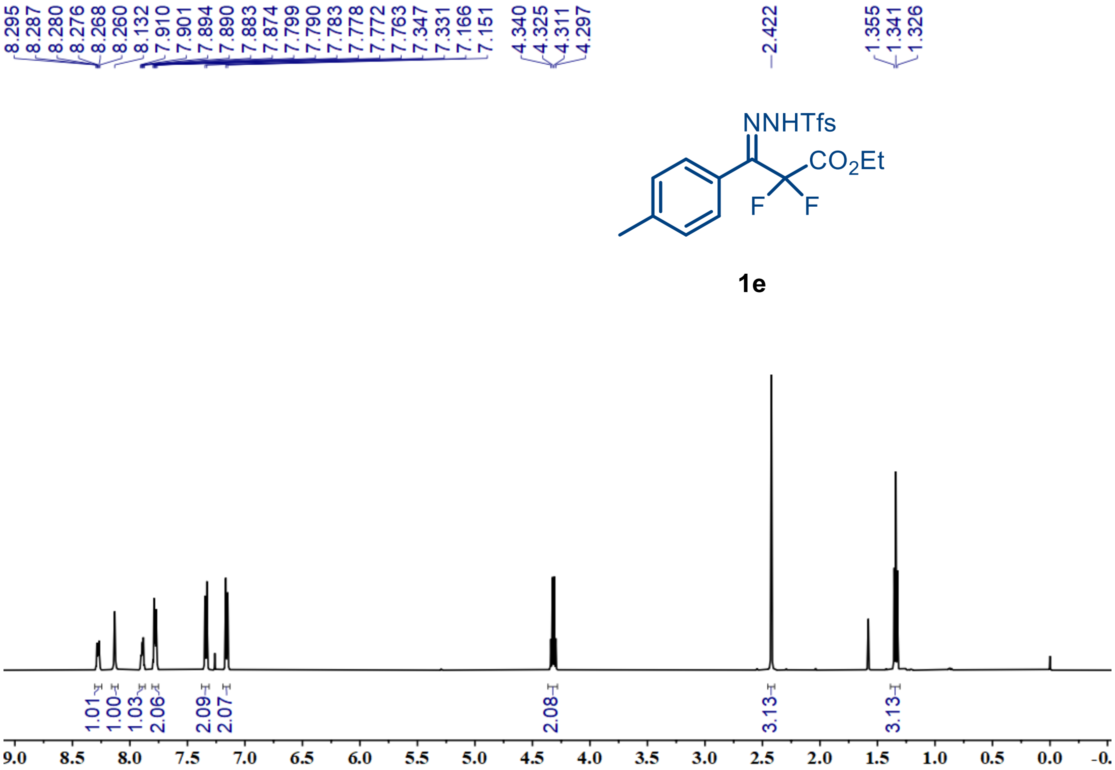

Figure S46, ^{1}H-NMR spectra copies of 1e related to scheme 2 and 3.

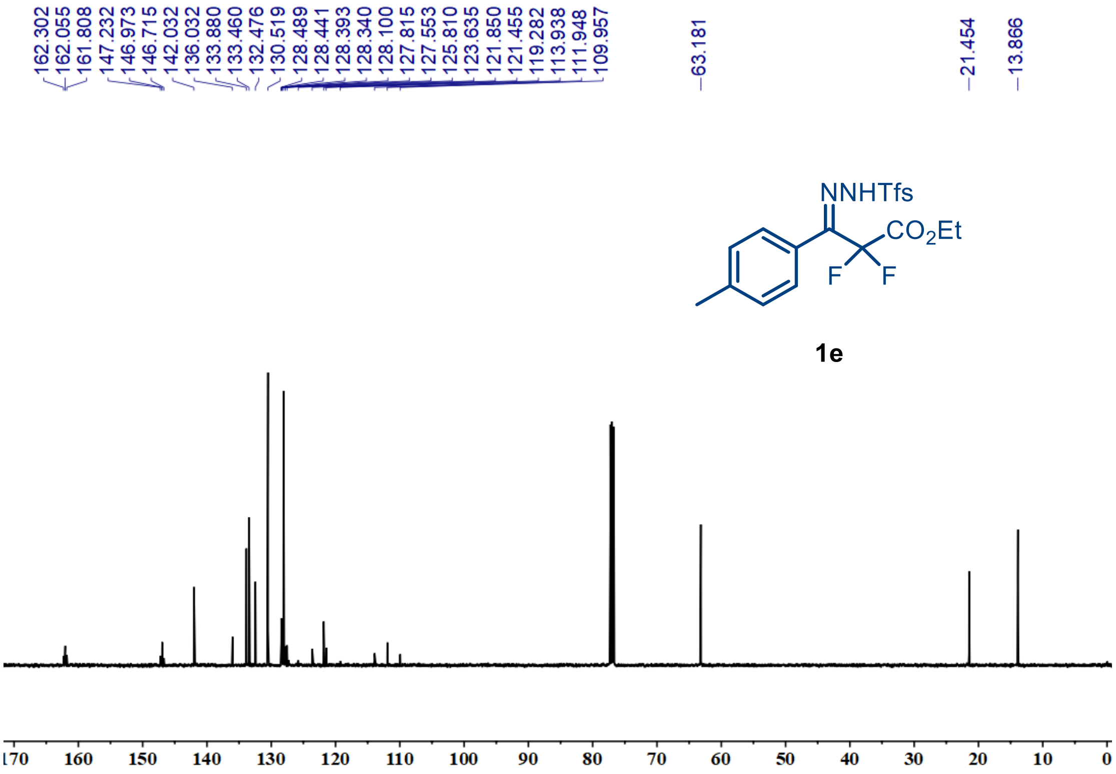

Figure S47, ^{13}C-NMR spectra copies of 1e related to scheme 2 and 3.

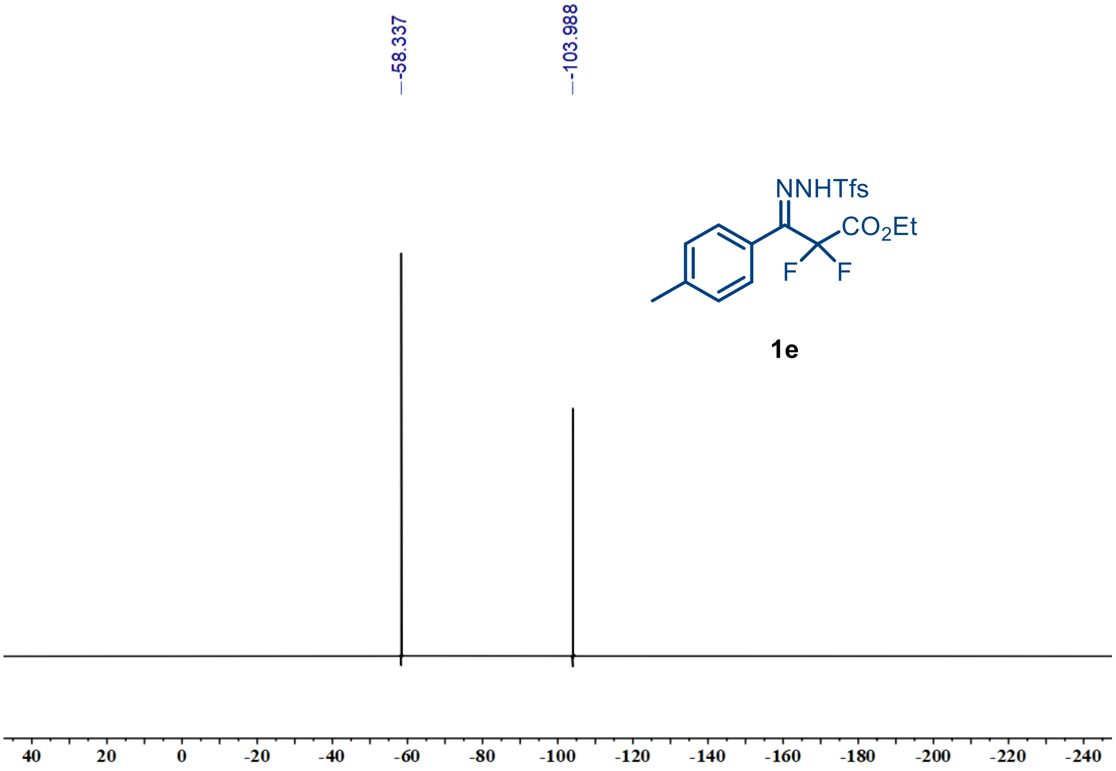

Figure S48, ^{19}F-NMR spectra copies of 1e related to scheme 2 and 3.

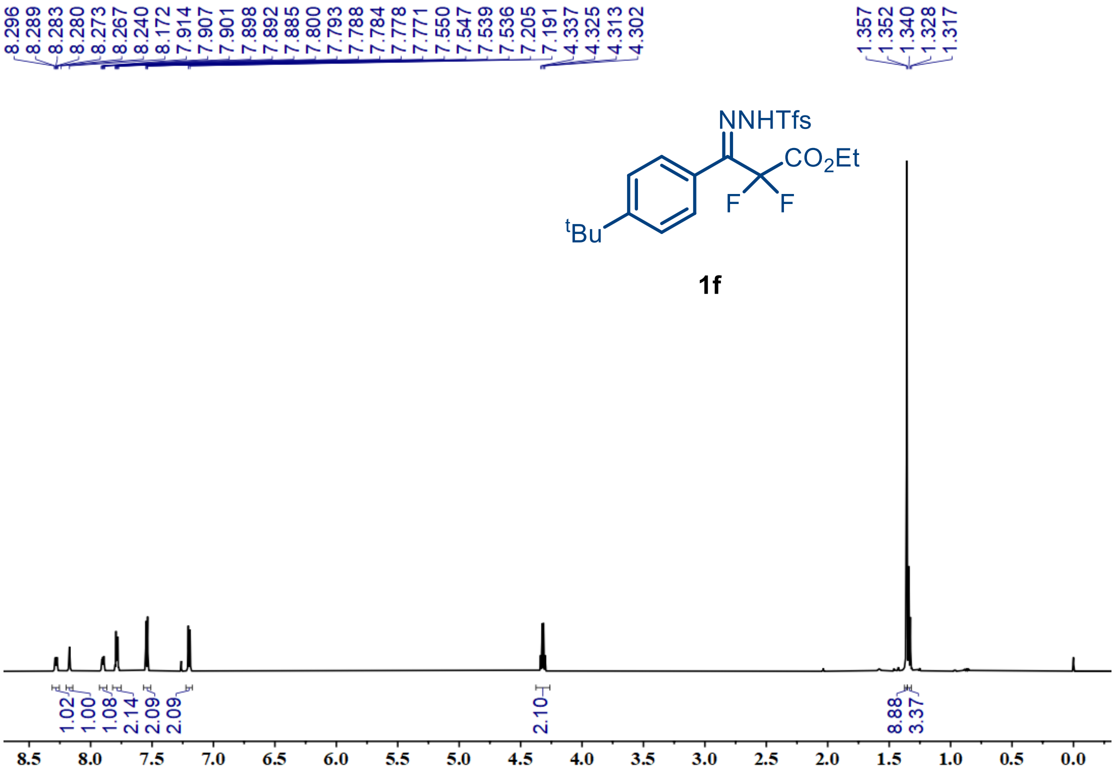

Figure S49, ^{1}H-NMR spectra copies of 1f related to scheme 2 and 3.

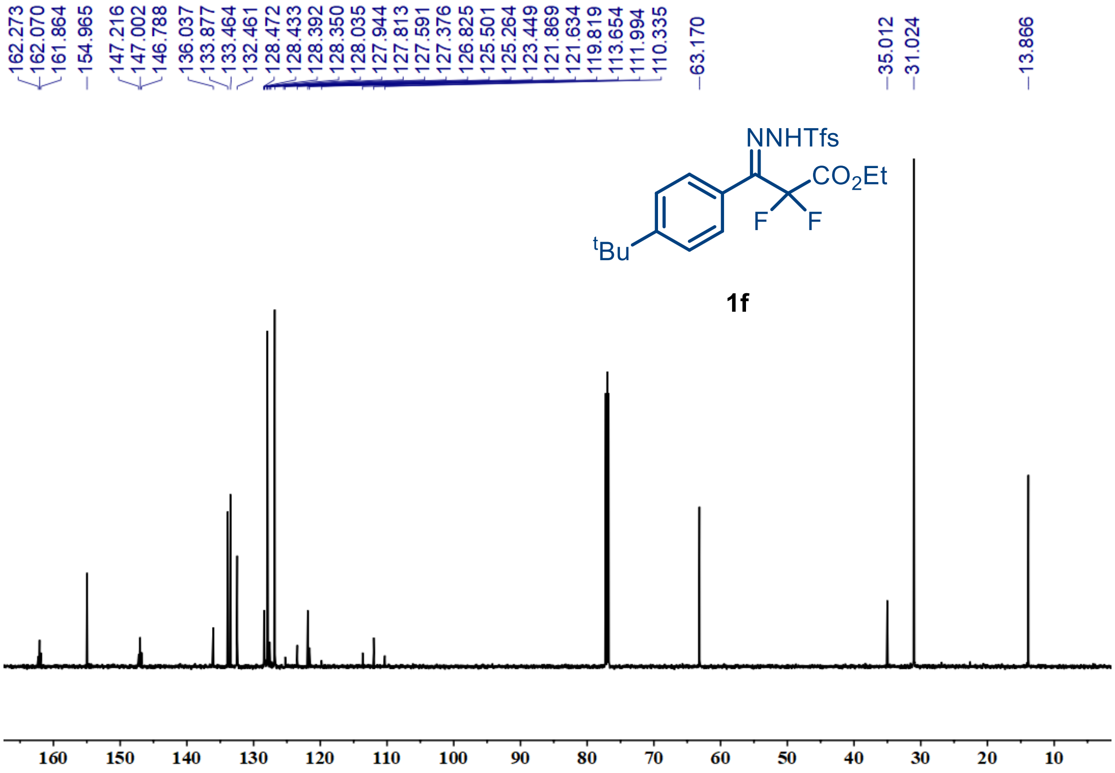

Figure S50, ^{13}C-NMR spectra copies of 1f related to scheme 2 and 3.

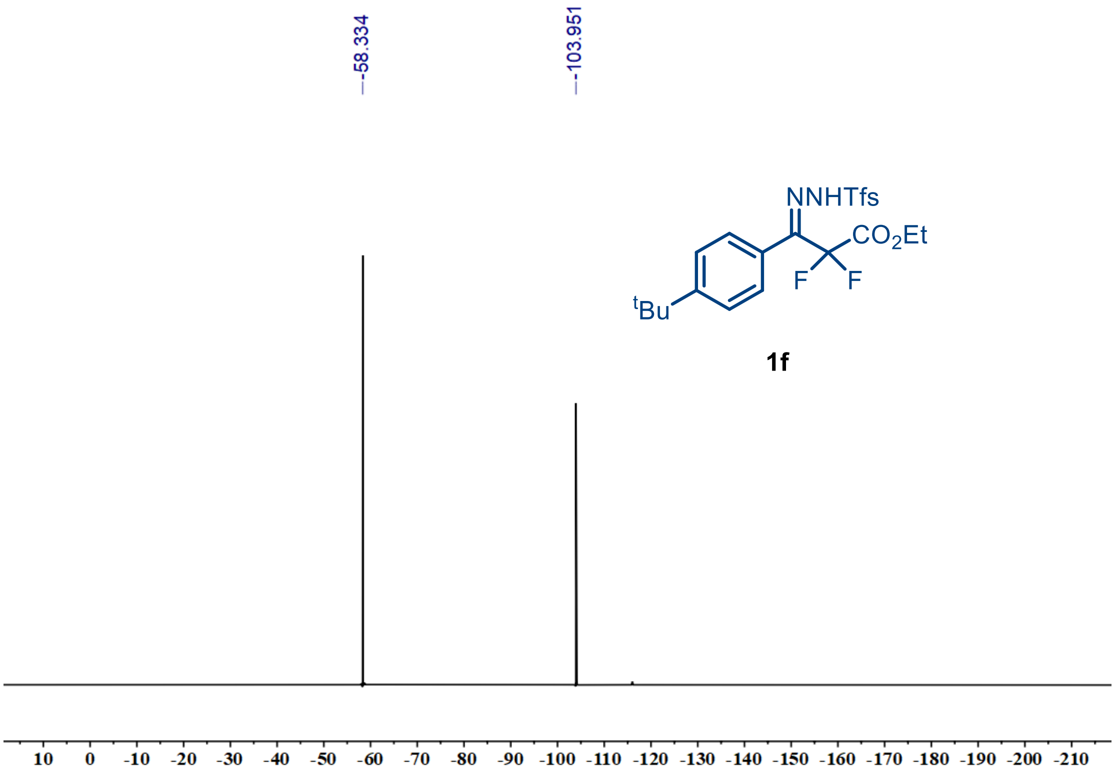

Figure S51, ^{19}F-NMR spectra copies of 1f related to scheme 2 and 3.

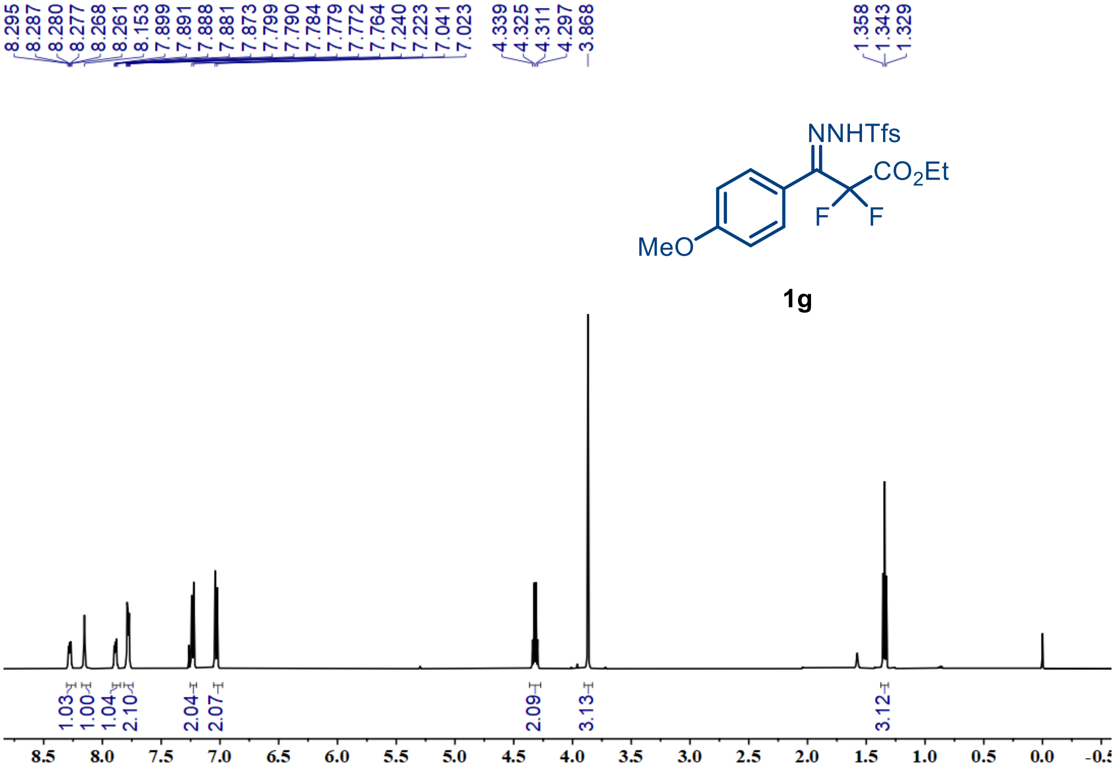

Figure S52, ^{1}H-NMR spectra copies of 1g related to scheme 2 and 3.

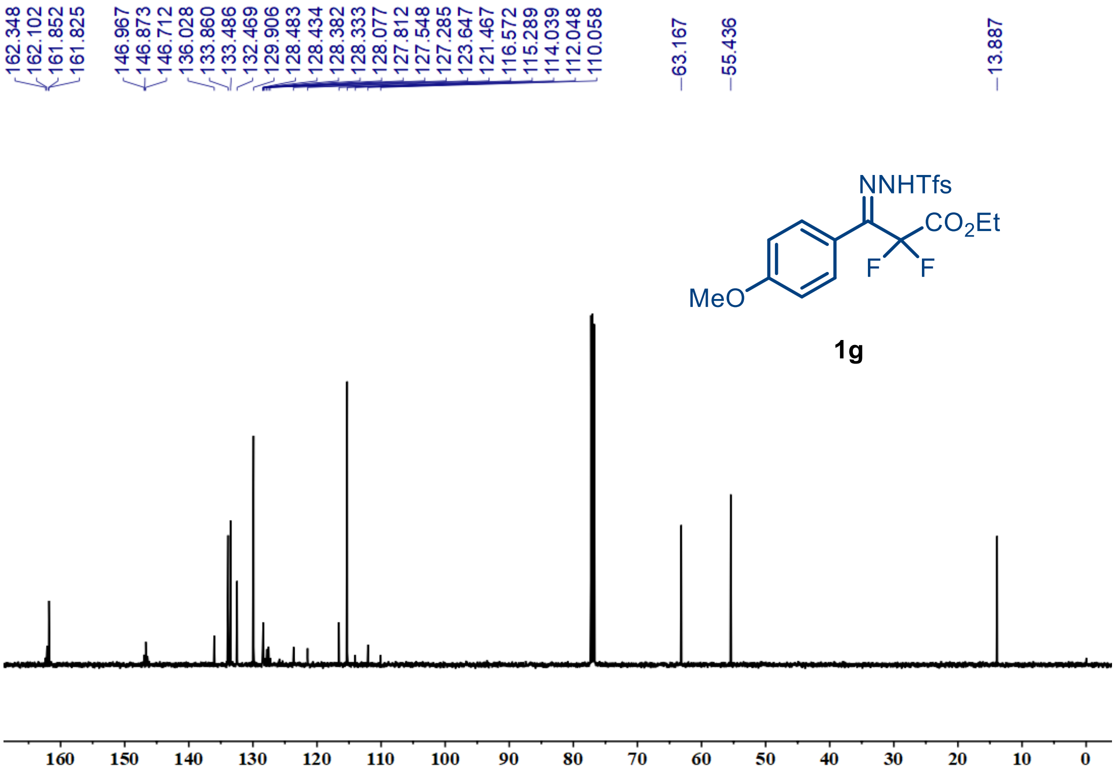

Figure S53, ^{13}C-NMR spectra copies of 1g related to scheme 2 and 3.

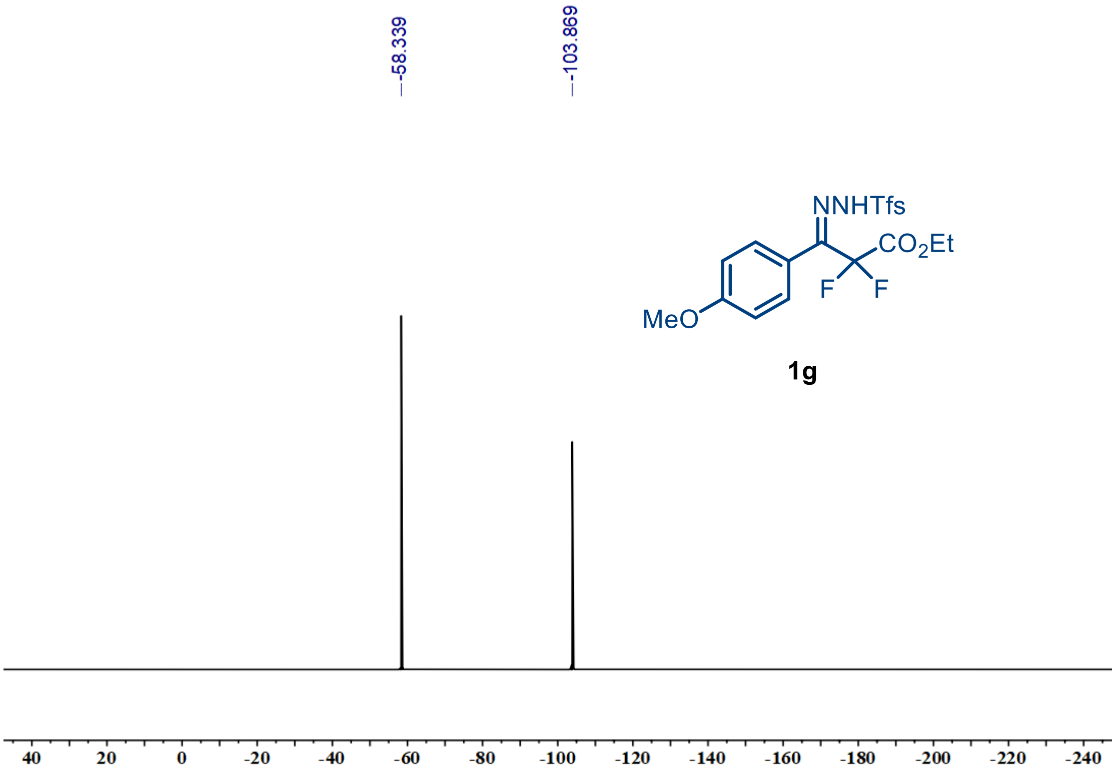

Figure S54, ^{19}F-NMR spectra copies of 1g related to scheme 2 and 3.

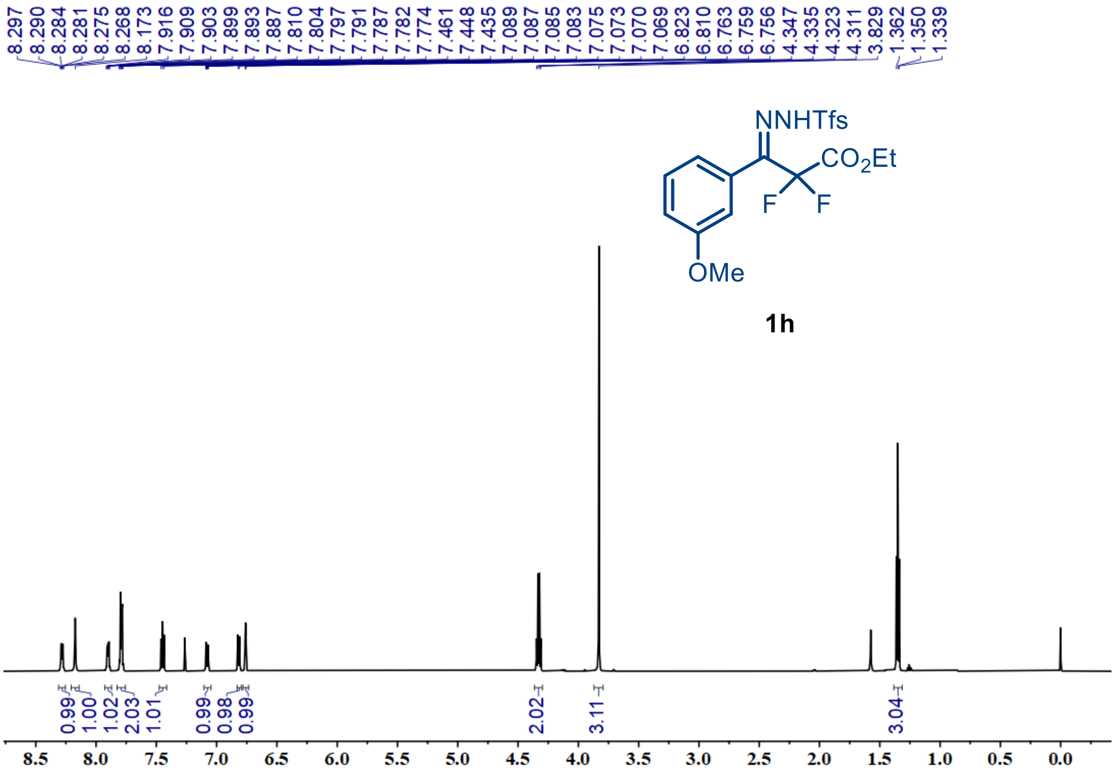

Figure S55, ^{1}H-NMR spectra copies of 1h related to scheme 2 and 3.

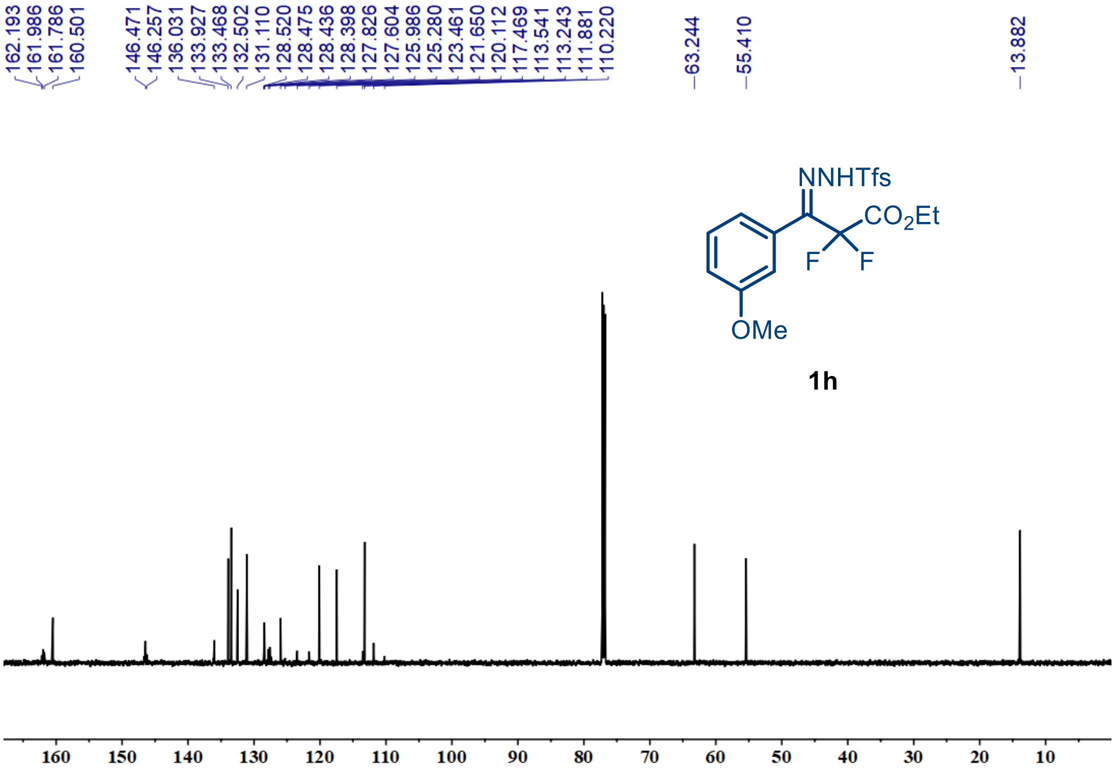

Figure S56, ^{13}C-NMR spectra copies of 1h related to scheme 2 and 3.

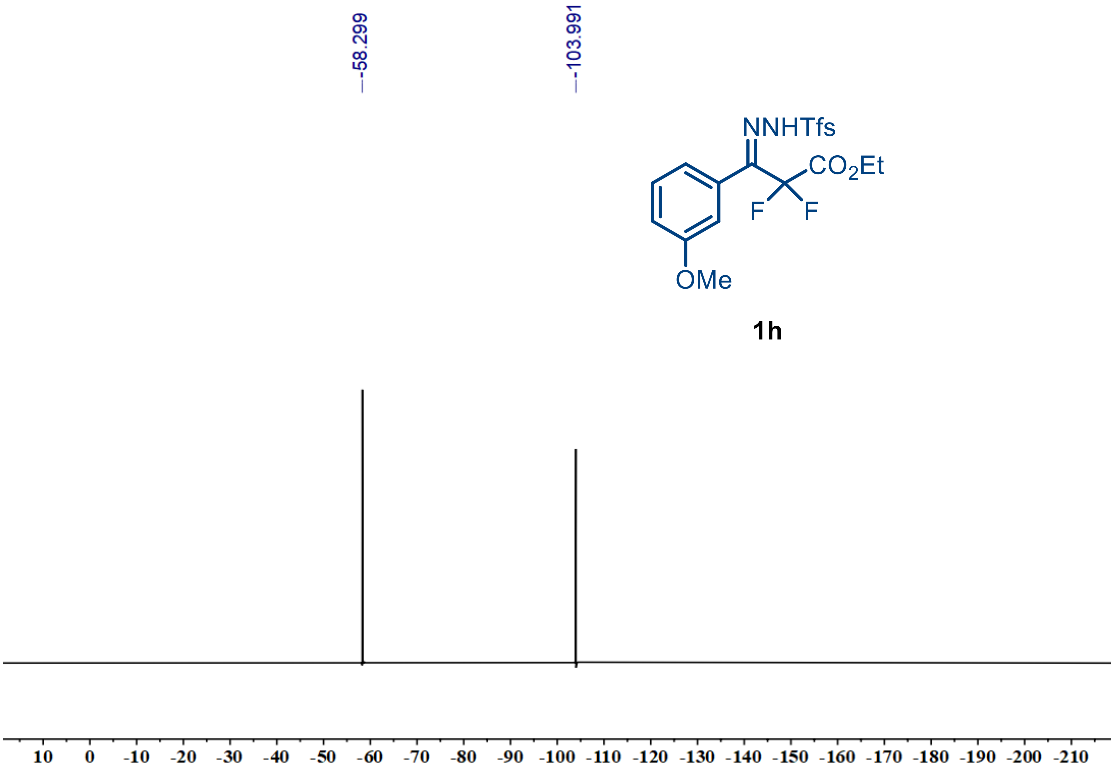

Figure S57, ^{19}F-NMR spectra copies of 1h related to scheme 2 and 3.

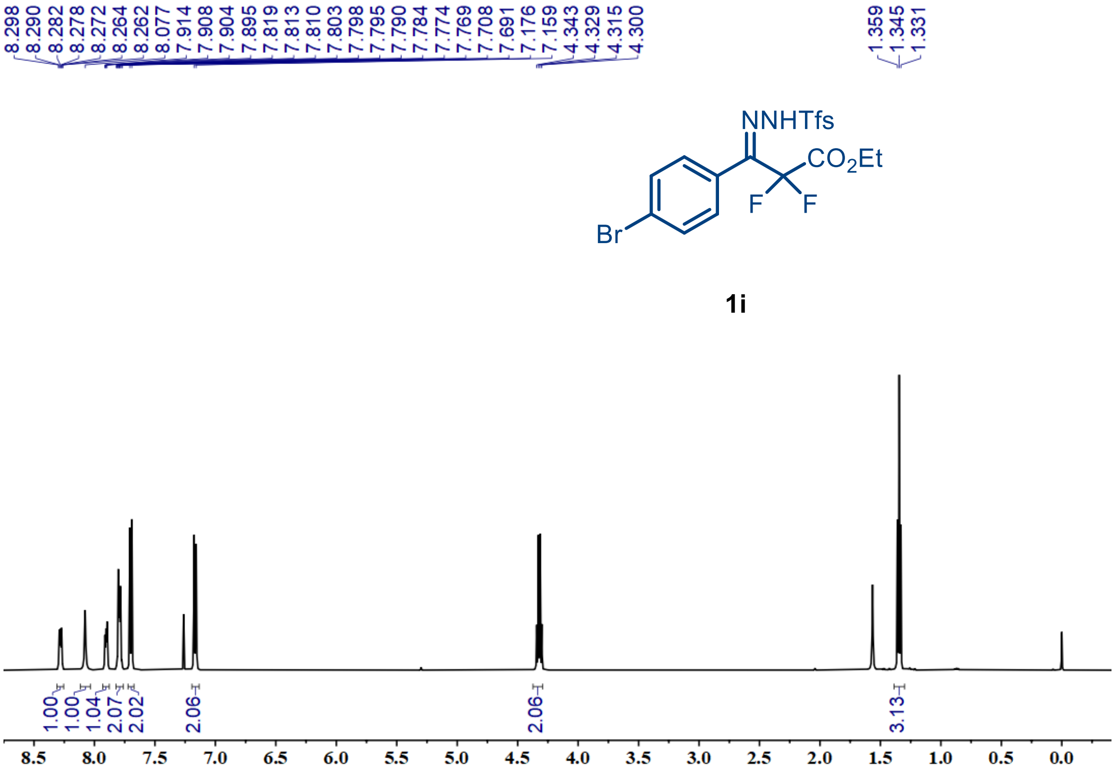

Figure S58, ^{1}H-NMR spectra copies of 1l related to scheme 2 and 3.

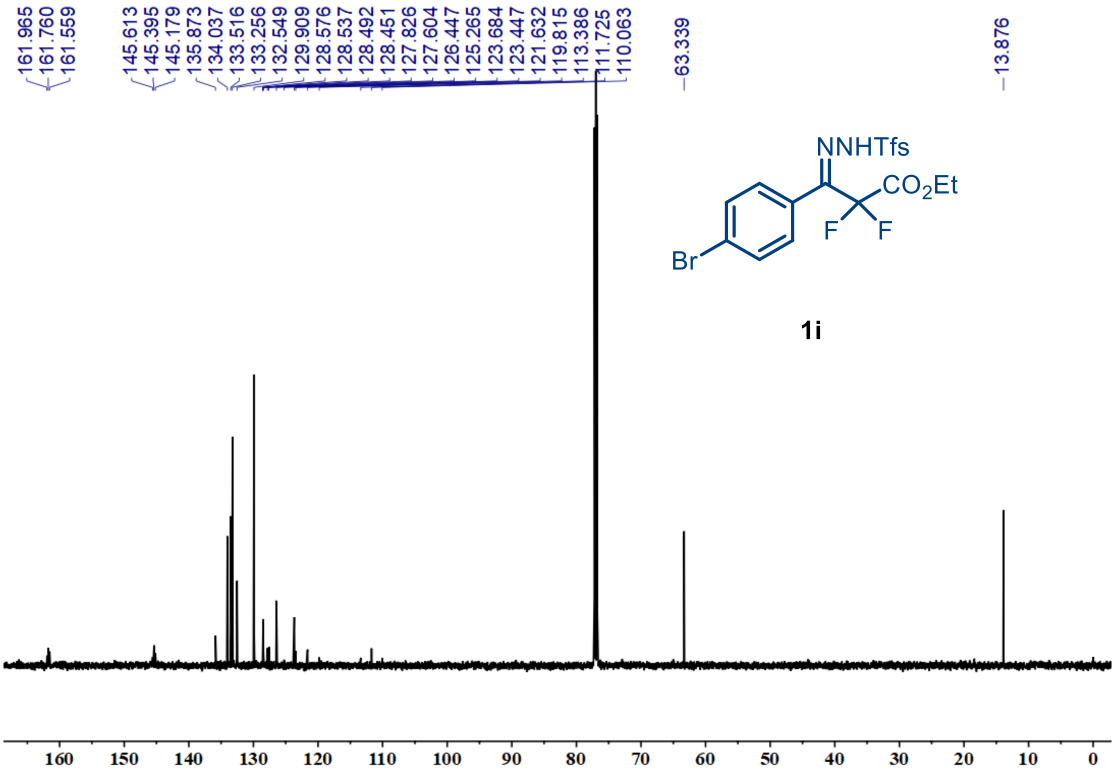

Figure S59, ^{13}C-NMR spectra copies of 1l related to scheme 2 and 3.

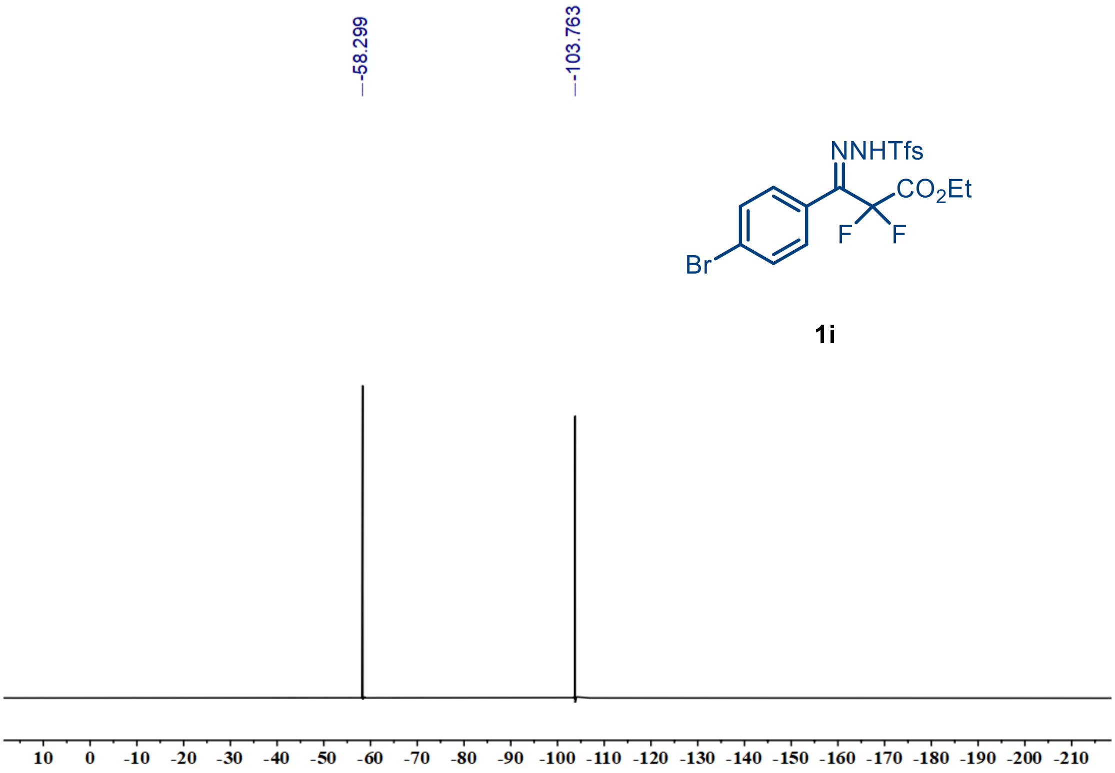

Figure S60, ^{19}F-NMR spectra copies of 1l related to scheme 2 and 3.

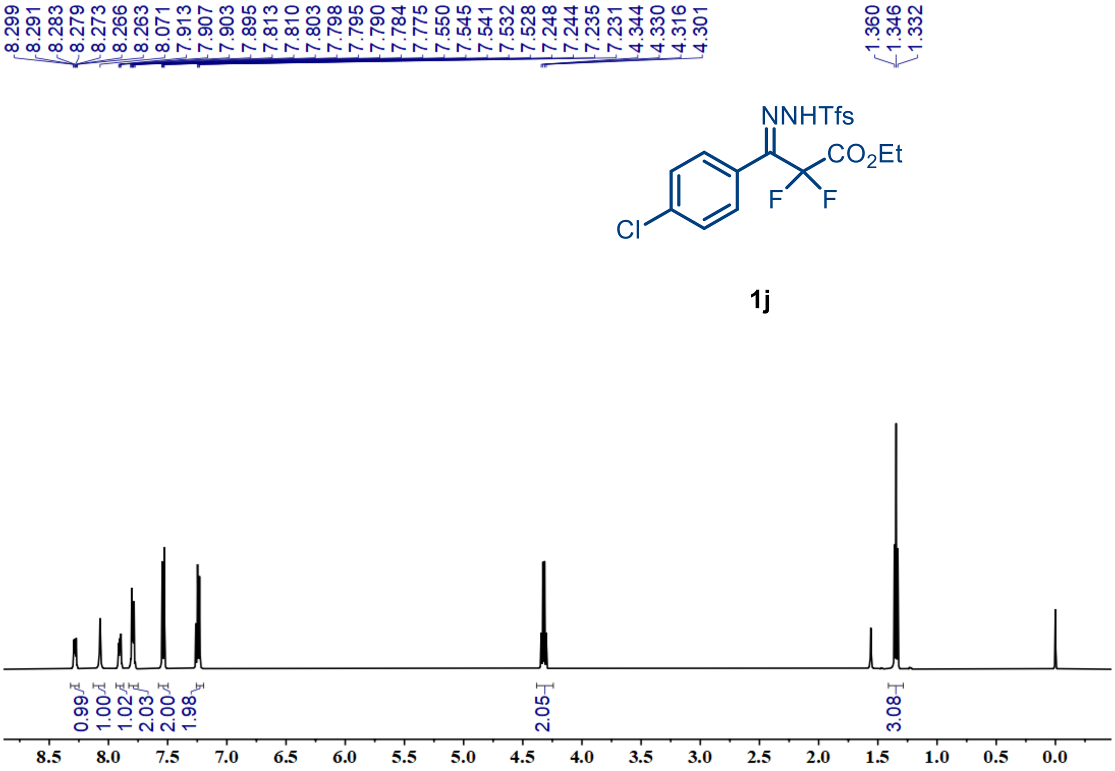

Figure S61, ^{1}H-NMR spectra copies of 1j related to scheme 2 and 3.

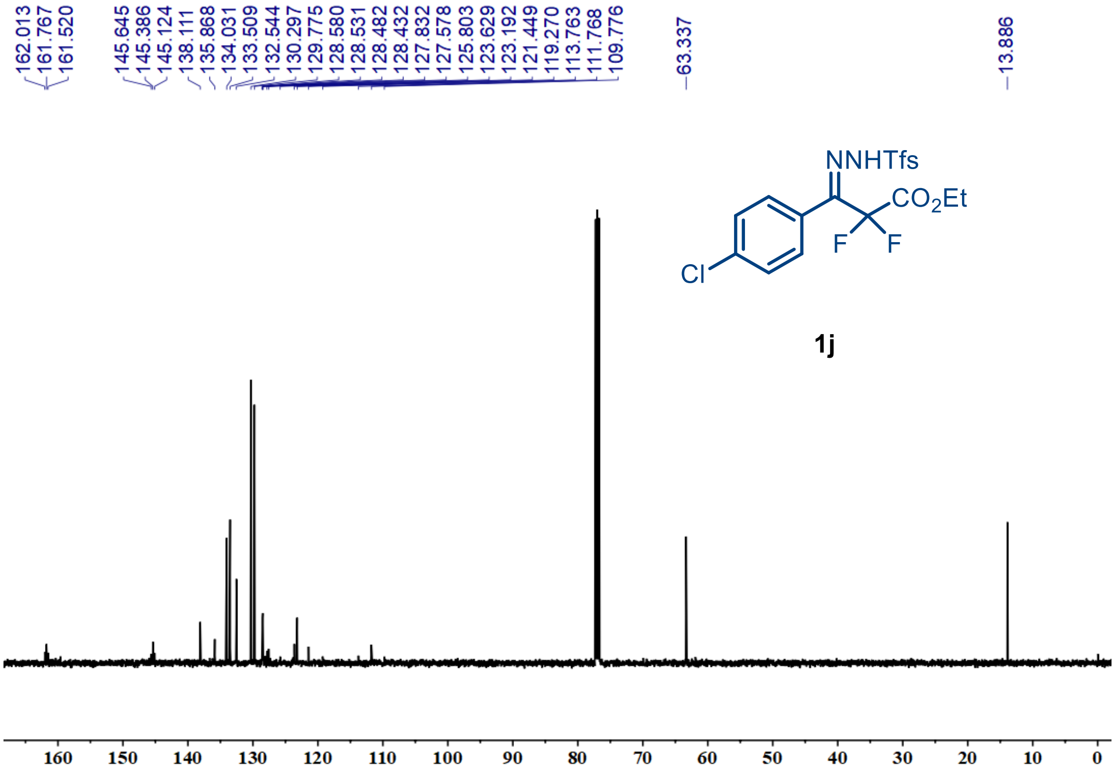

Figure S62, ^{13}C-NMR spectra copies of 1j related to scheme 2 and 3.

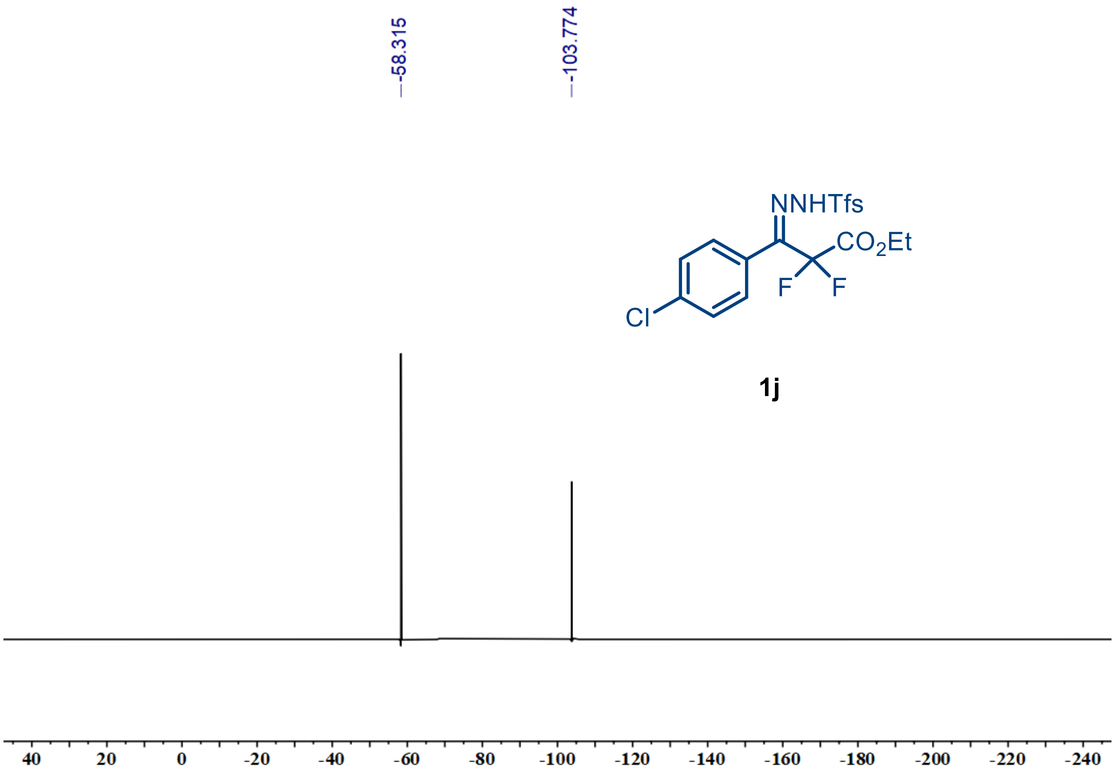

Figure S63, ^{19}F-NMR spectra copies of 1j related to scheme 2 and 3.

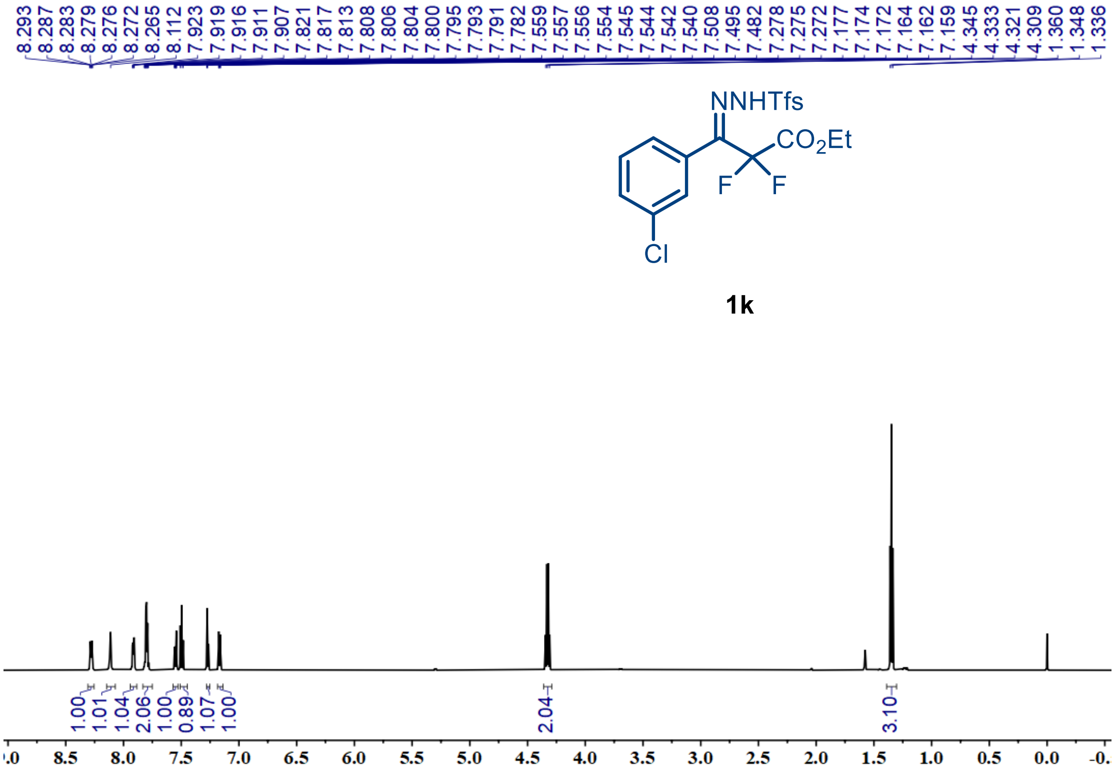

Figure S64, ^{1}H-NMR spectra copies of 1k related to scheme 2 and 3.

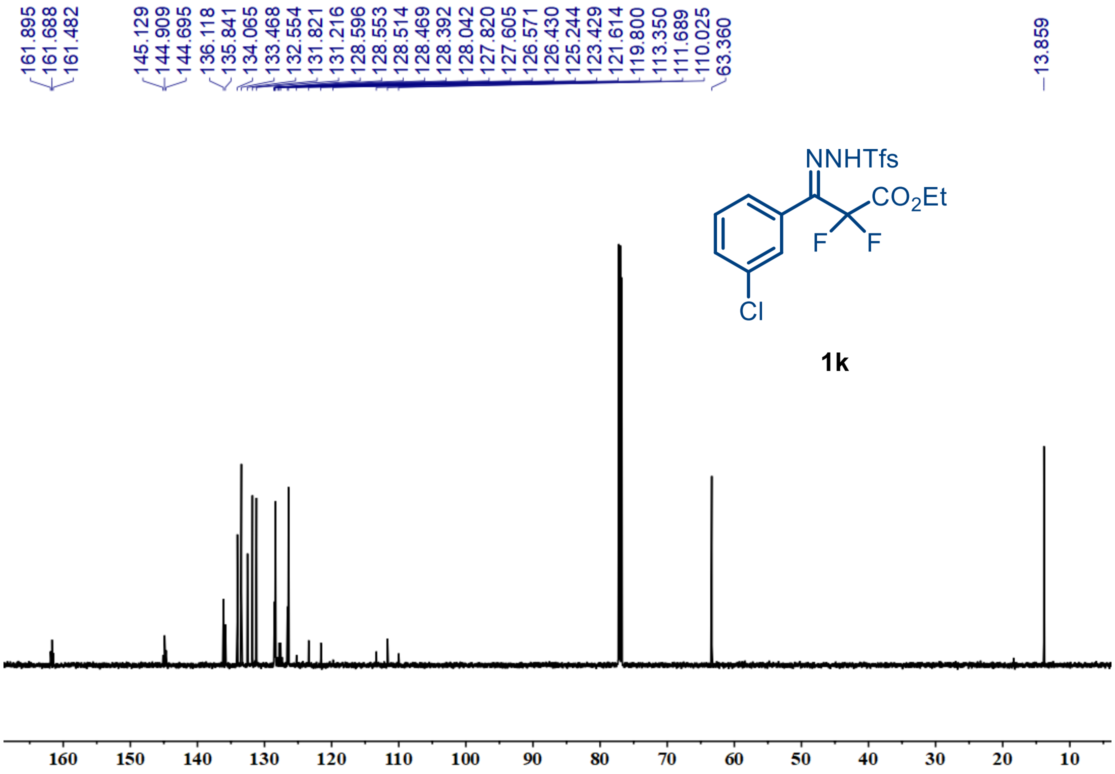

Figure S65, ^{13}C-NMR spectra copies of 1k related to scheme 2 and 3.

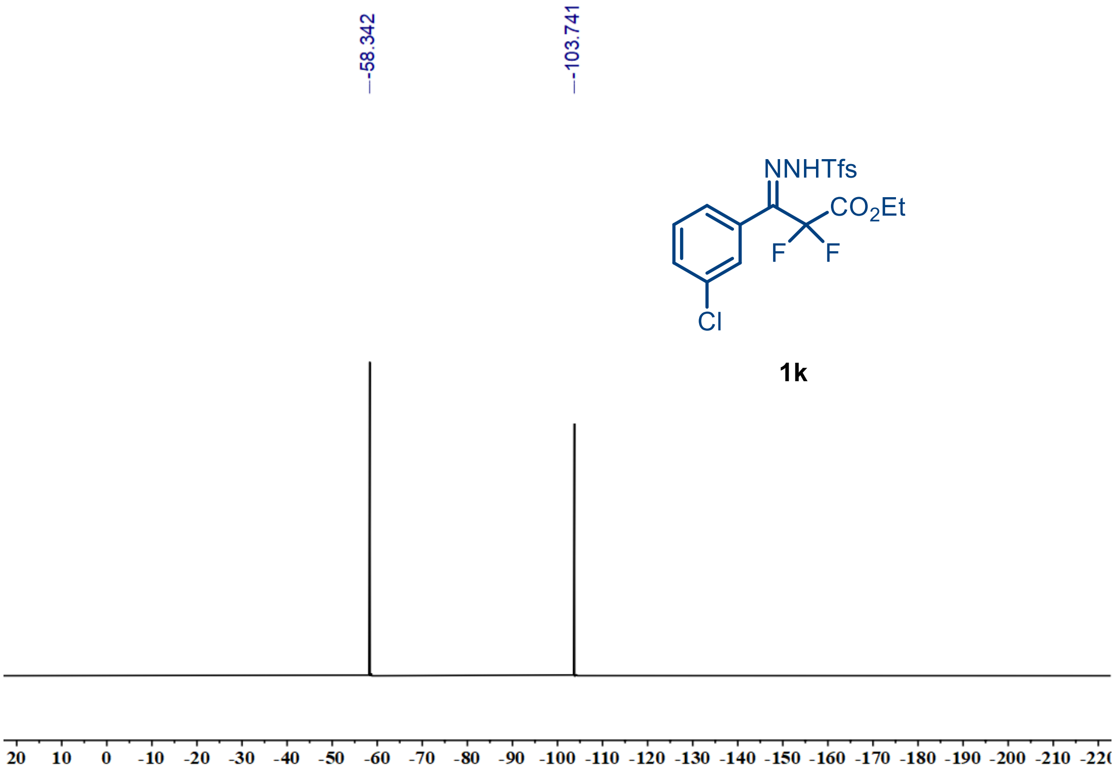

Figure S66, ^{19}F-NMR spectra copies of 1k related to scheme 2 and 3.

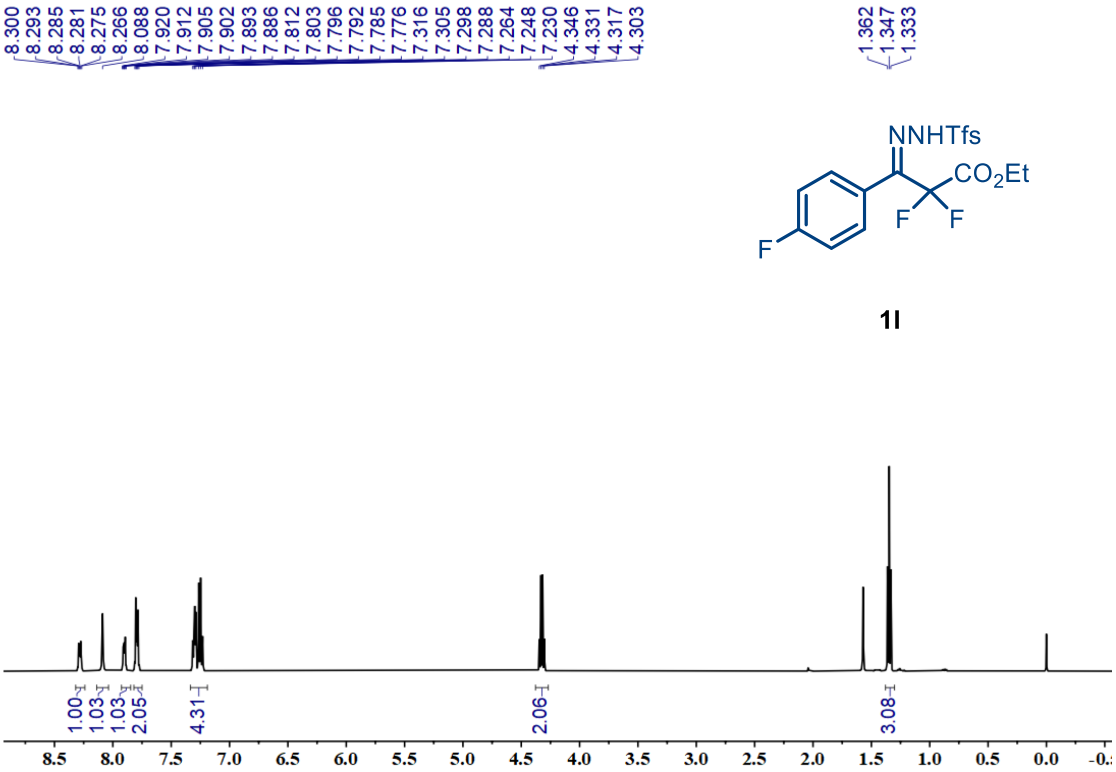

Figure S67, ^{1}H-NMR spectra copies of 1f related to scheme 2 and 3.

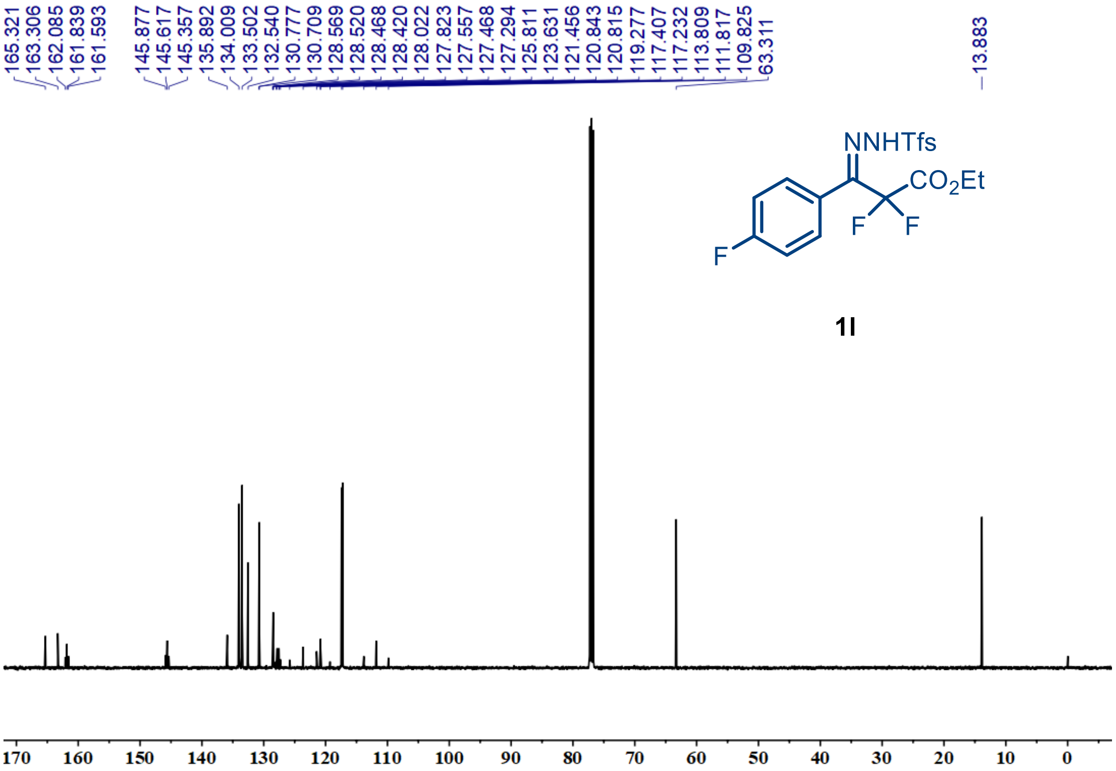

Figure S68, ^{13}C-NMR spectra copies of 1f related to scheme 2 and 3.

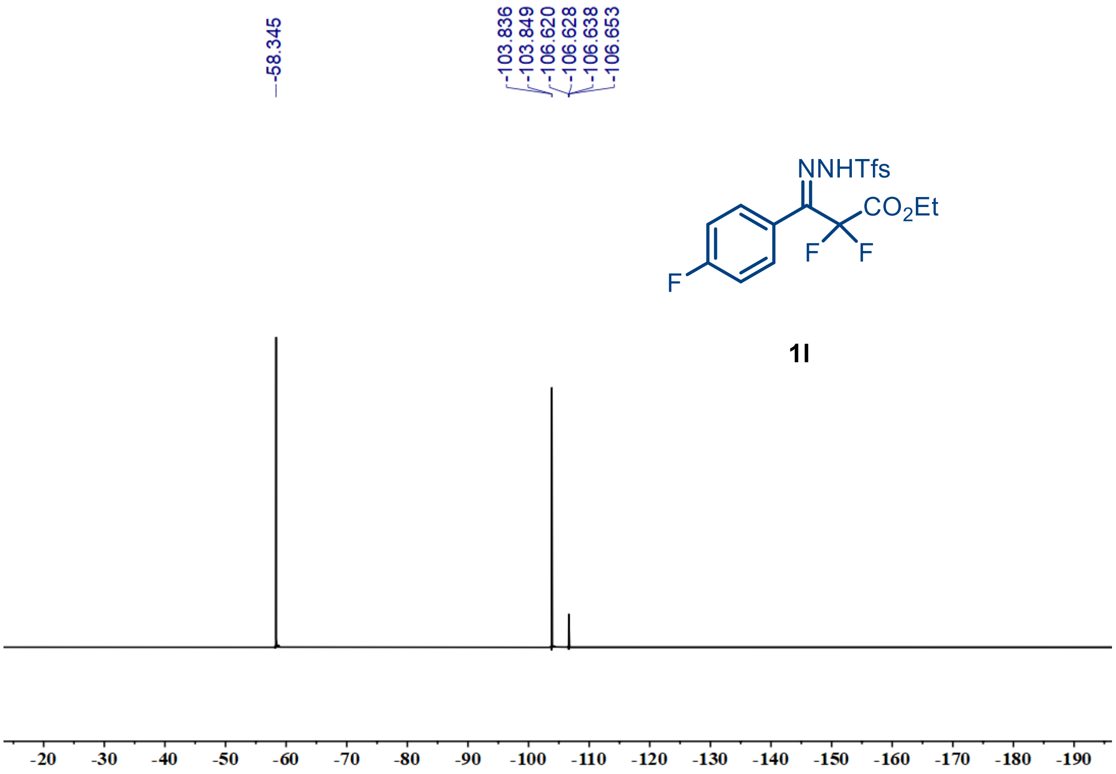

Figure S69, ^{19}F-NMR spectra copies of 1f related to scheme 2 and 3.

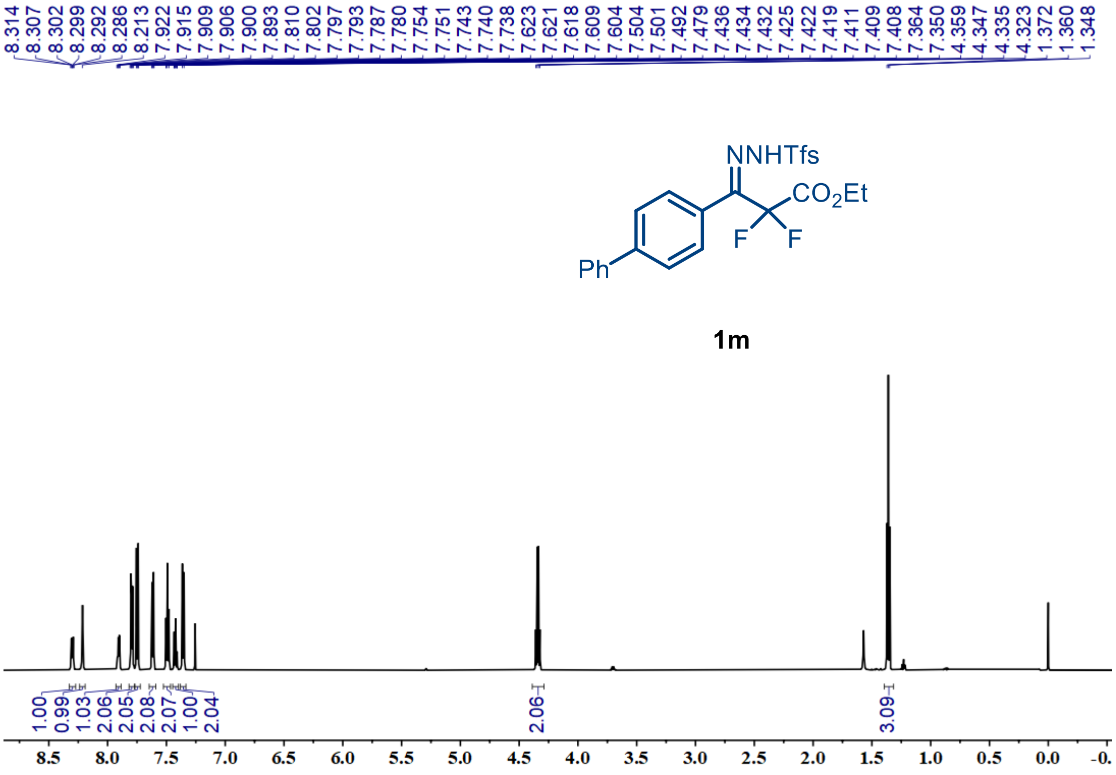

Figure S70, ^{1}H-NMR spectra copies of 1m related to scheme 2 and 3.

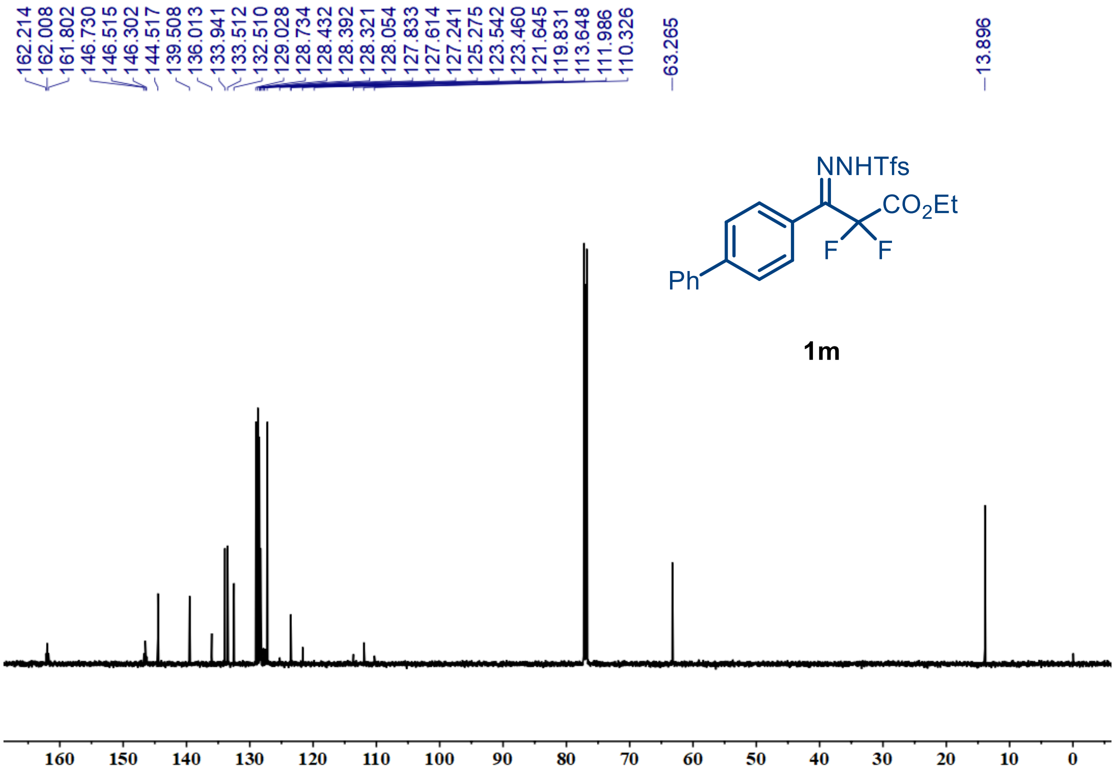

Figure S71, ^{13}C-NMR spectra copies of 1m related to scheme 2 and 3.

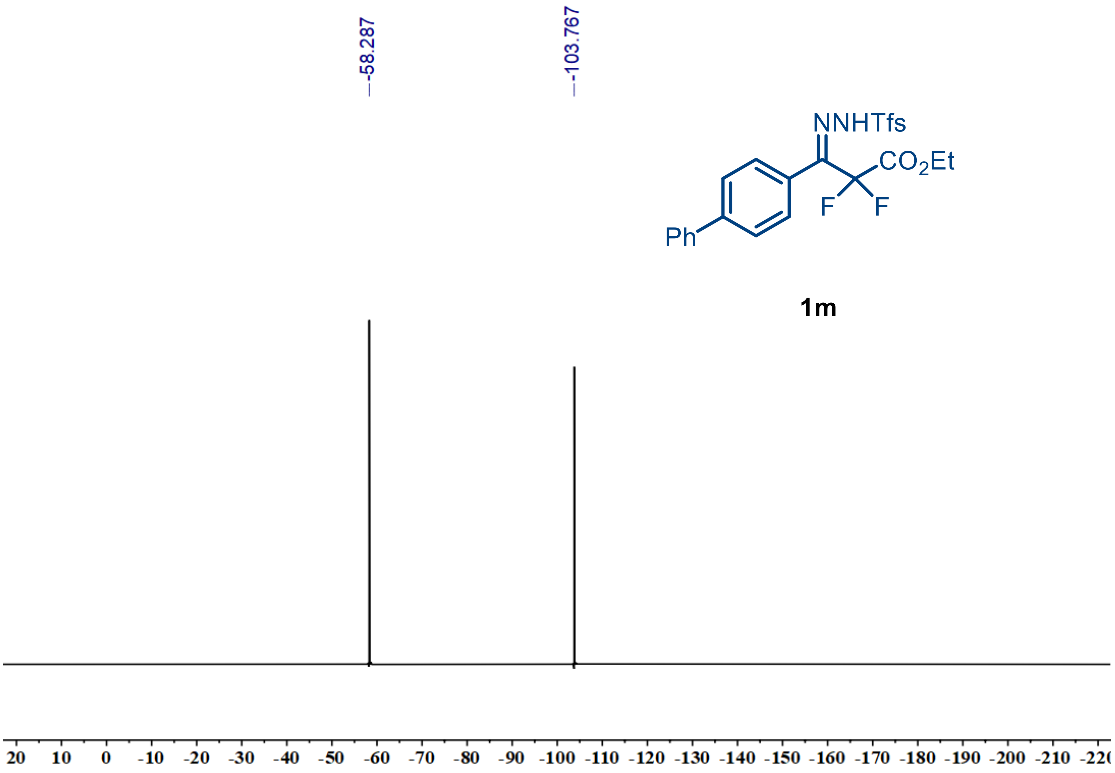

Figure S72, ^{19}F-NMR spectra copies of 1m related to scheme 2 and 3.

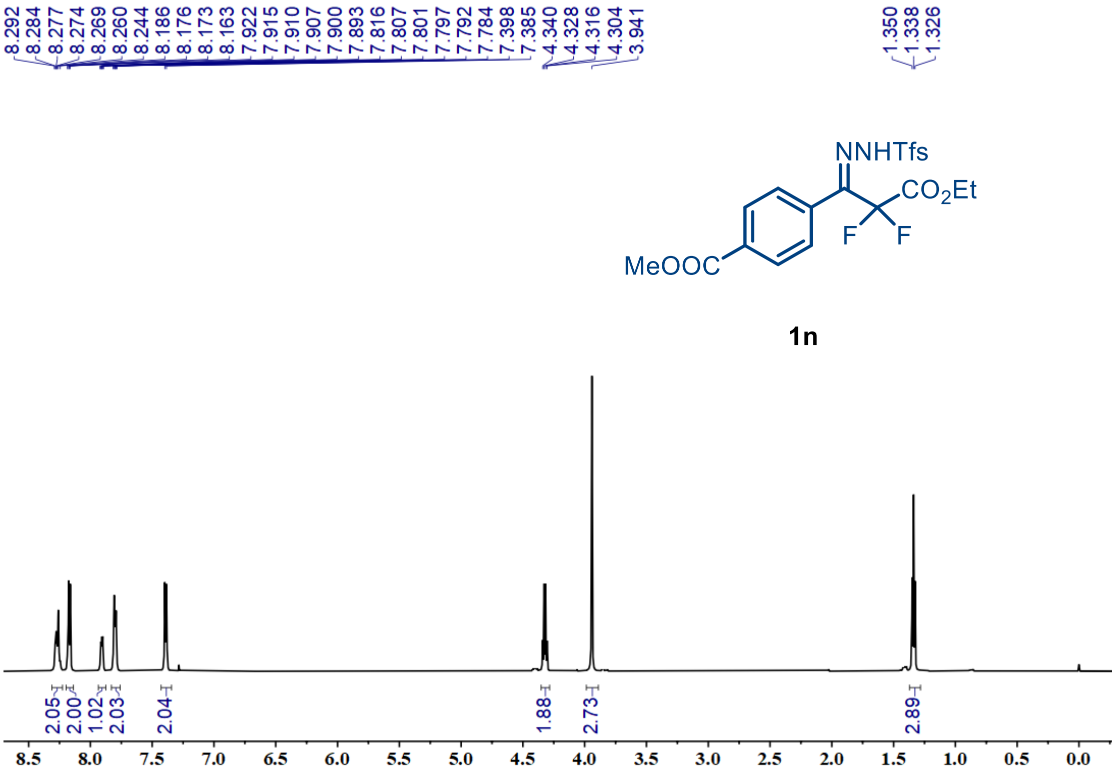

Figure S73, ^{1}H-NMR spectra copies of 1n related to scheme 2 and 3.

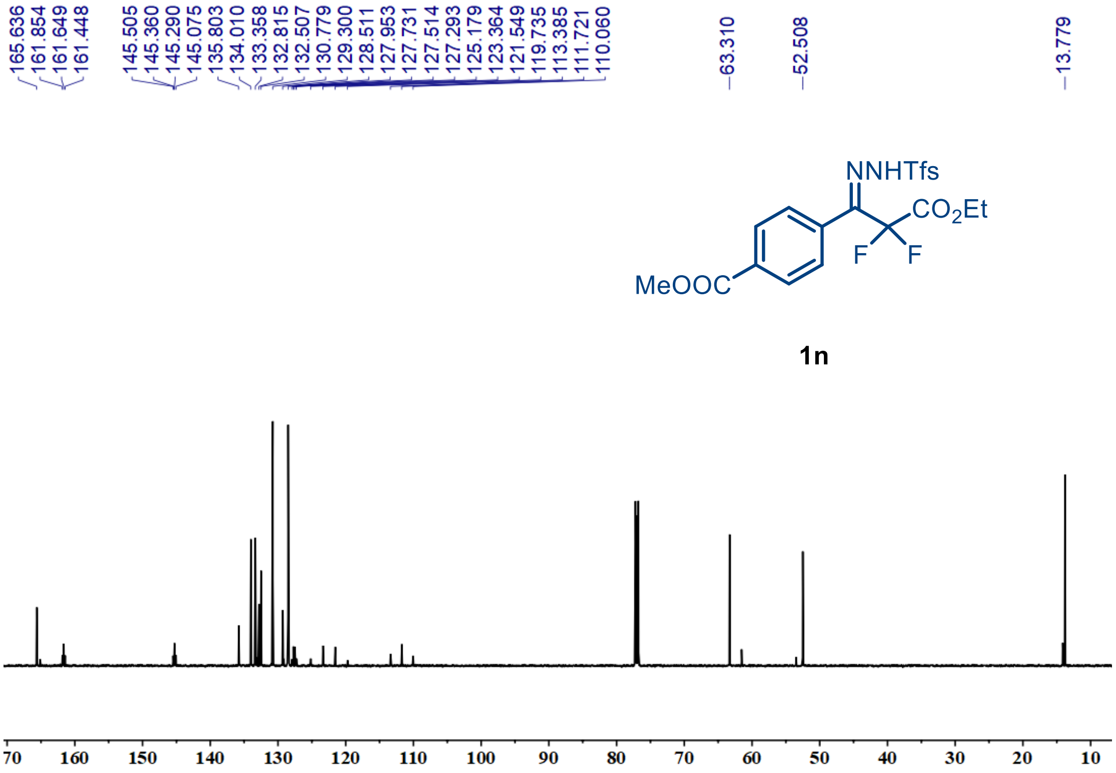

Figure S74, ^{13}C-NMR spectra copies of 1n related to scheme 2 and 3.

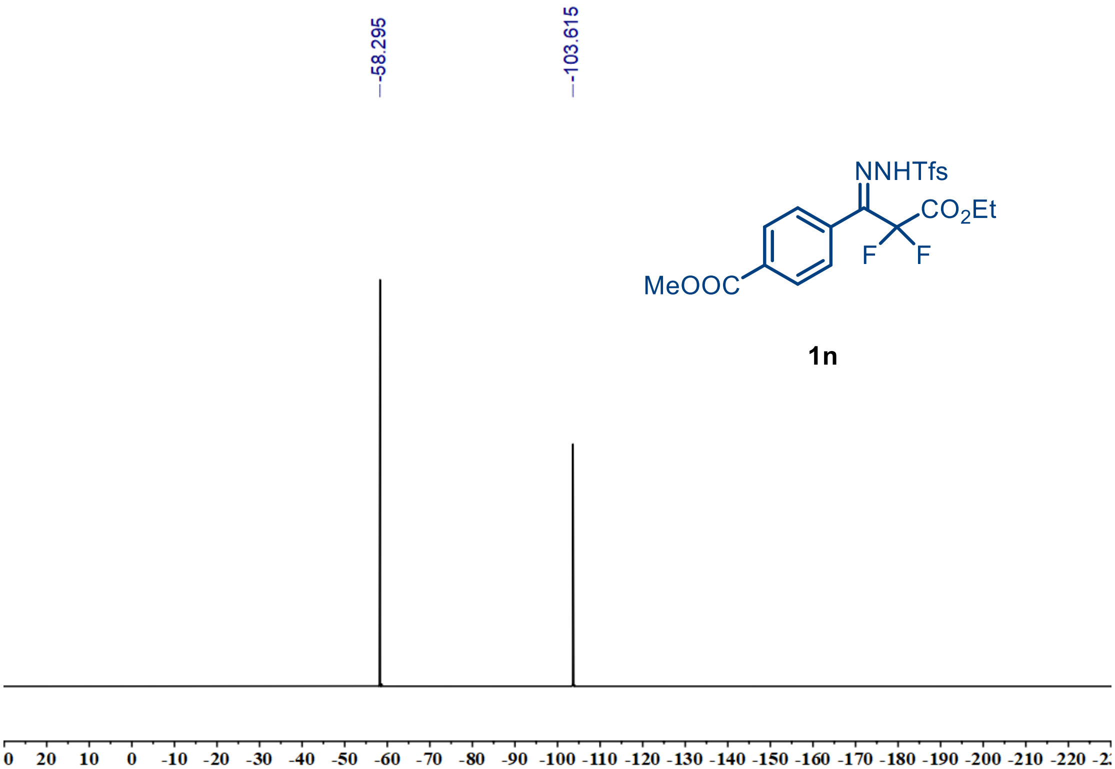

1n

Figure S75, ^{19}F-NMR spectra copies of 1n related to scheme 2 and 3.

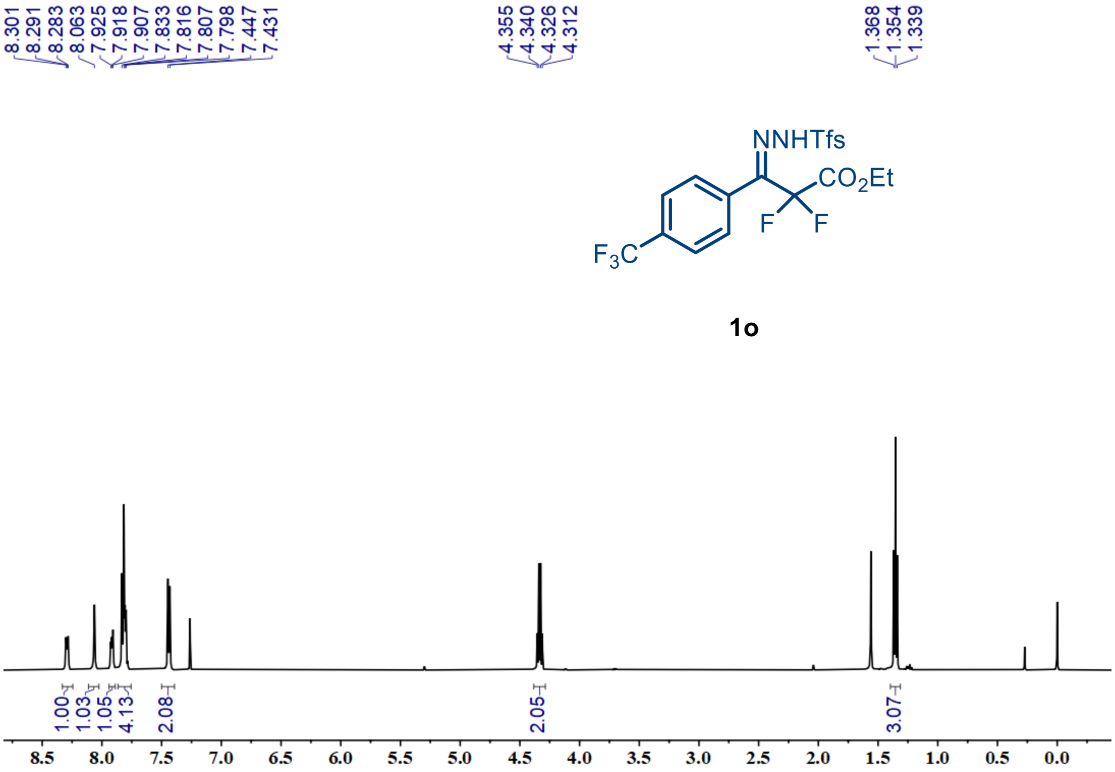

1o

Figure S76, ^{1}H-NMR spectra copies of 1o related to scheme 2 and 3.

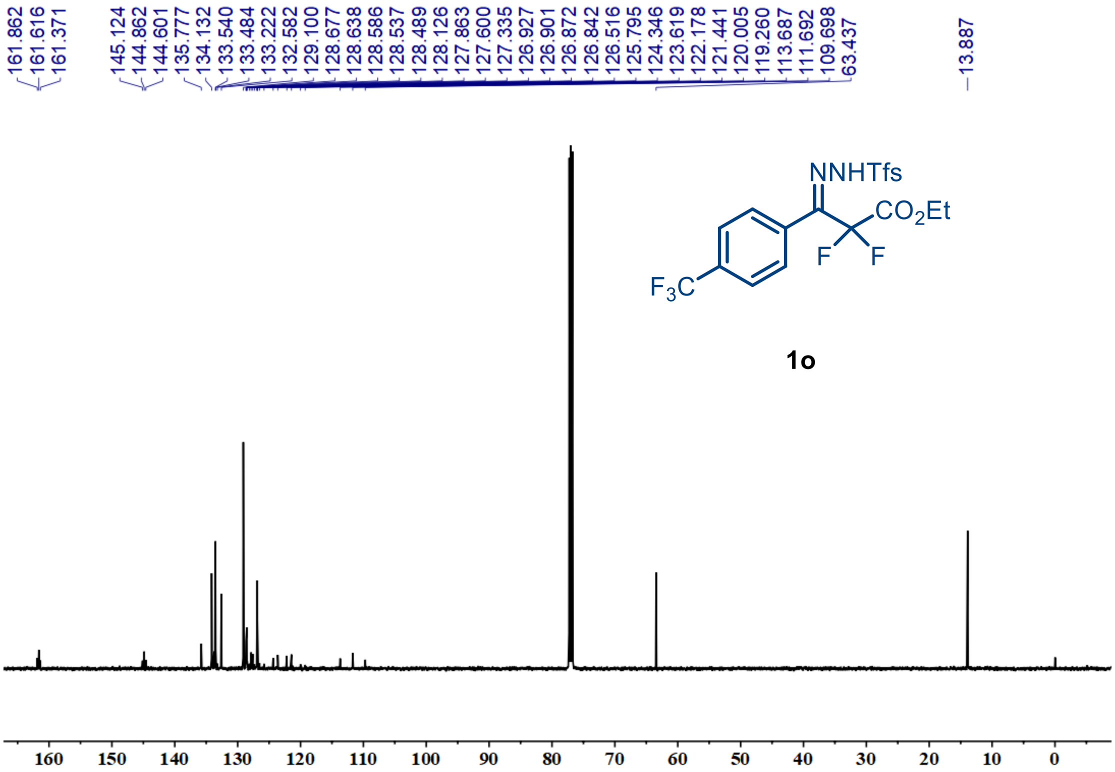

Figure S77, ^{13}C-NMR spectra copies of 1o related to scheme 2 and 3.

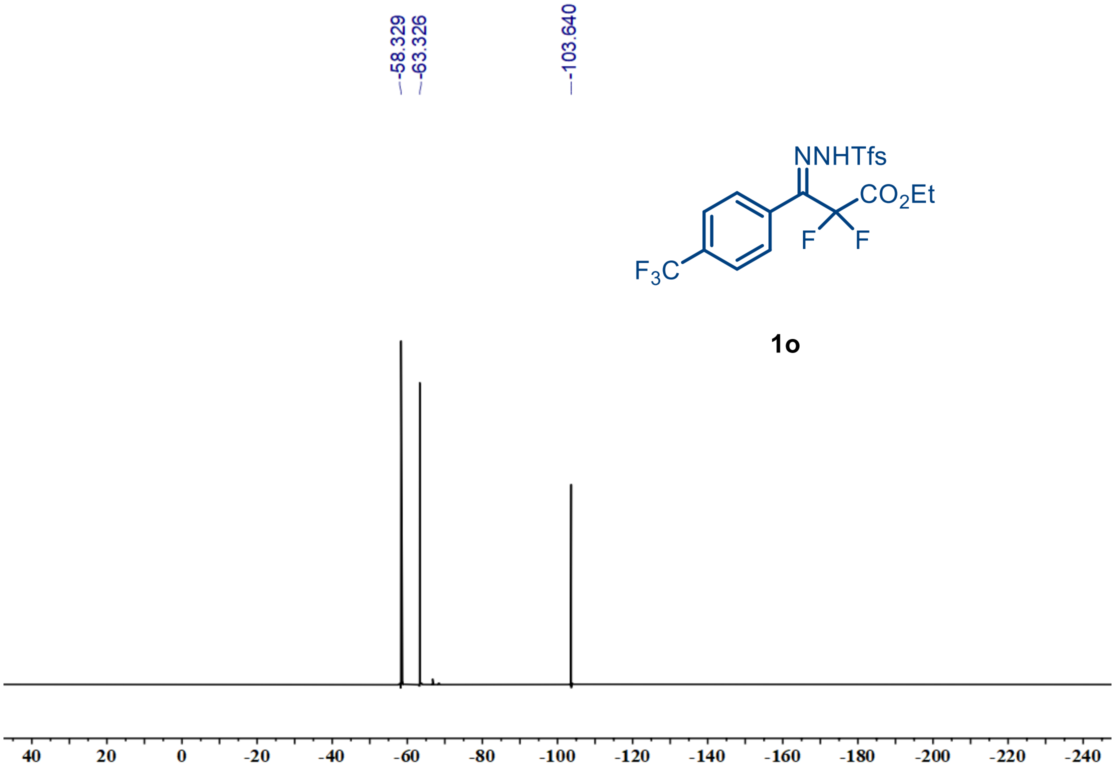

Figure S78, ^{19}F-NMR spectra copies of 1o related to scheme 2 and 3.

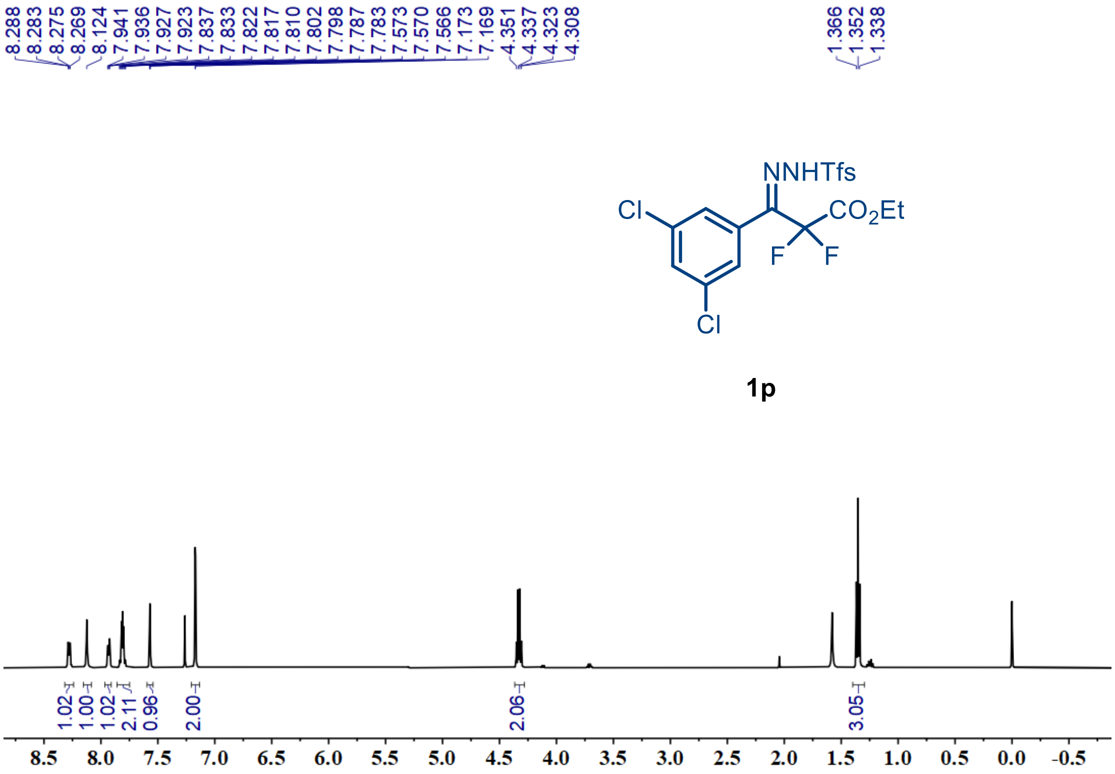

Figure S79, ^{1}H-NMR spectra copies of 1p related to scheme 2 and 3.

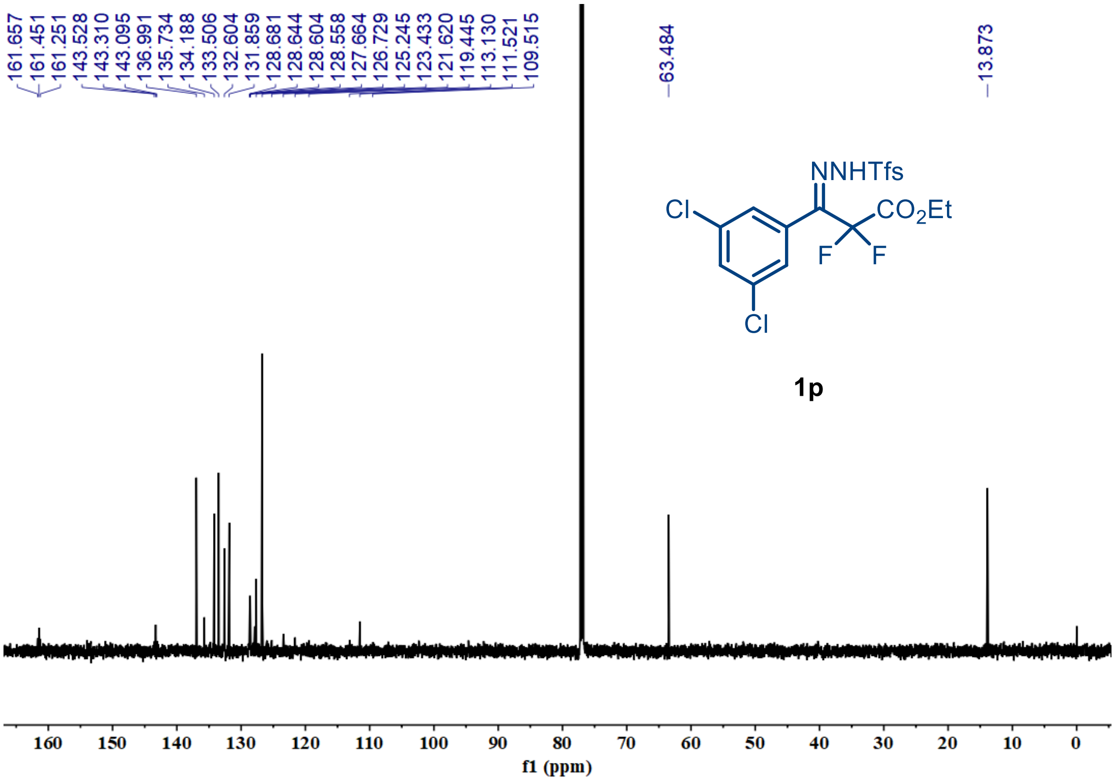

Figure S80, ^{13}C-NMR spectra copies of 1p related to scheme 2 and 3.

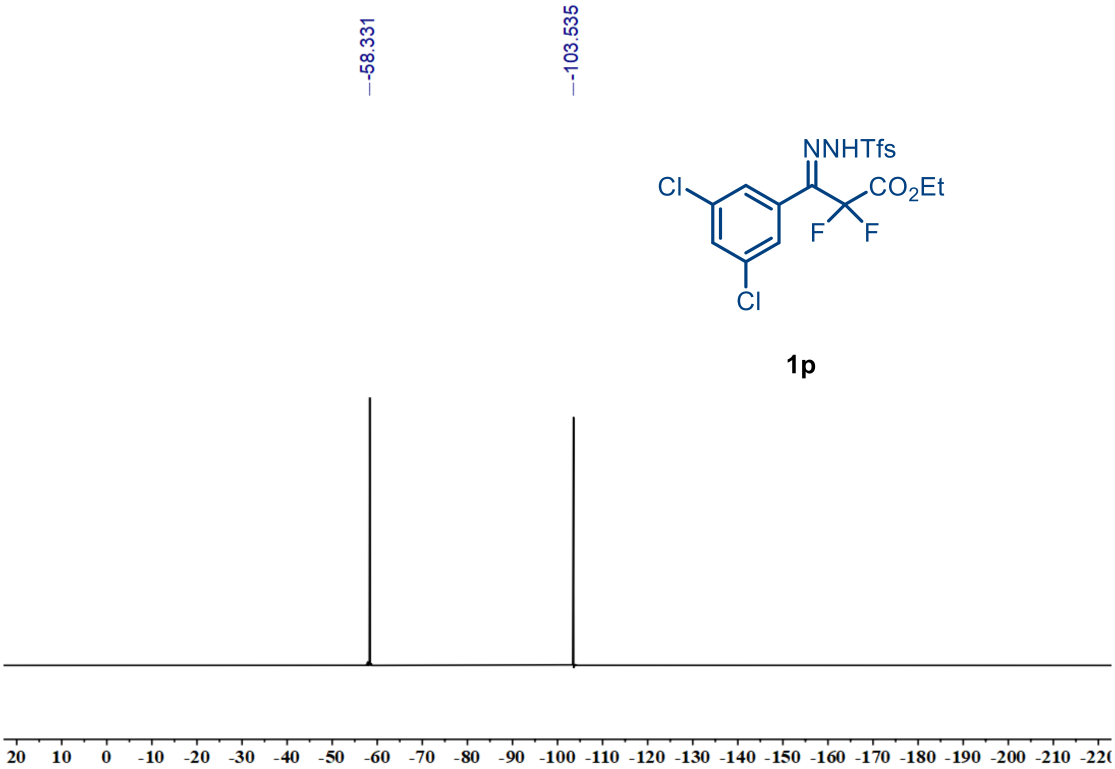

Figure S81, ^{19}F-NMR spectra copies of 1p related to scheme 2 and 3.

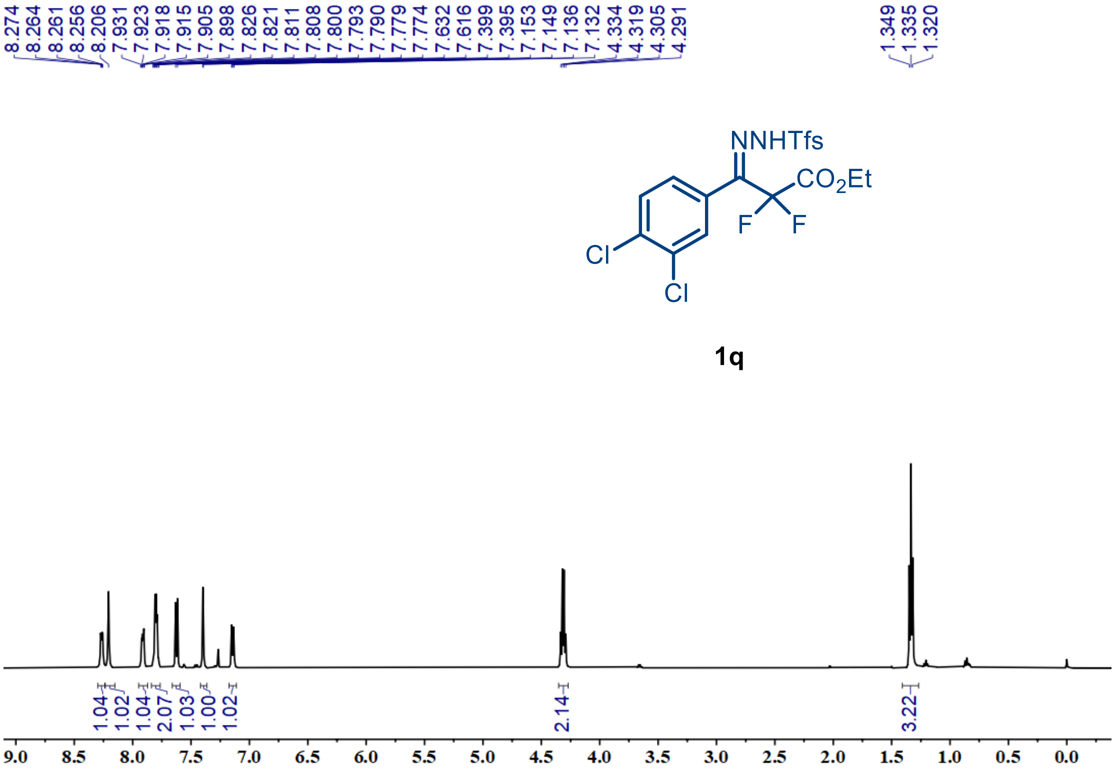

Figure S82, ^{1}H-NMR spectra copies of 1q related to scheme 2 and 3.

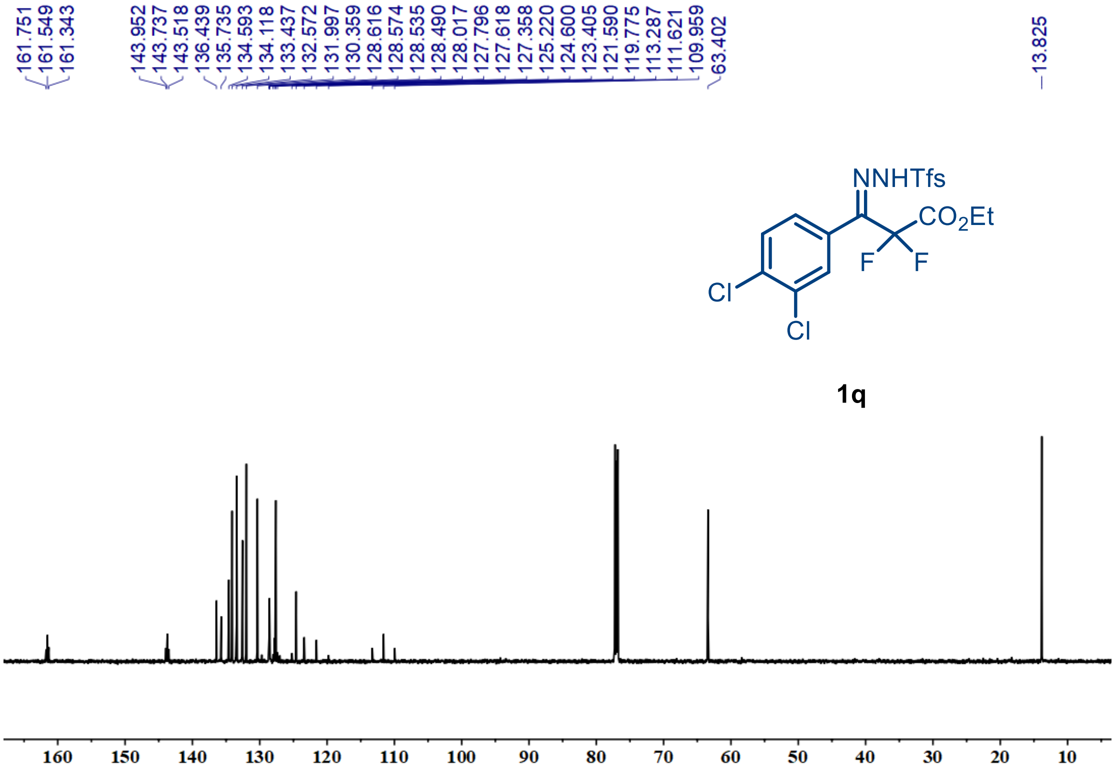

Figure S83, ^{13}C-NMR spectra copies of 1q related to scheme 2 and 3.

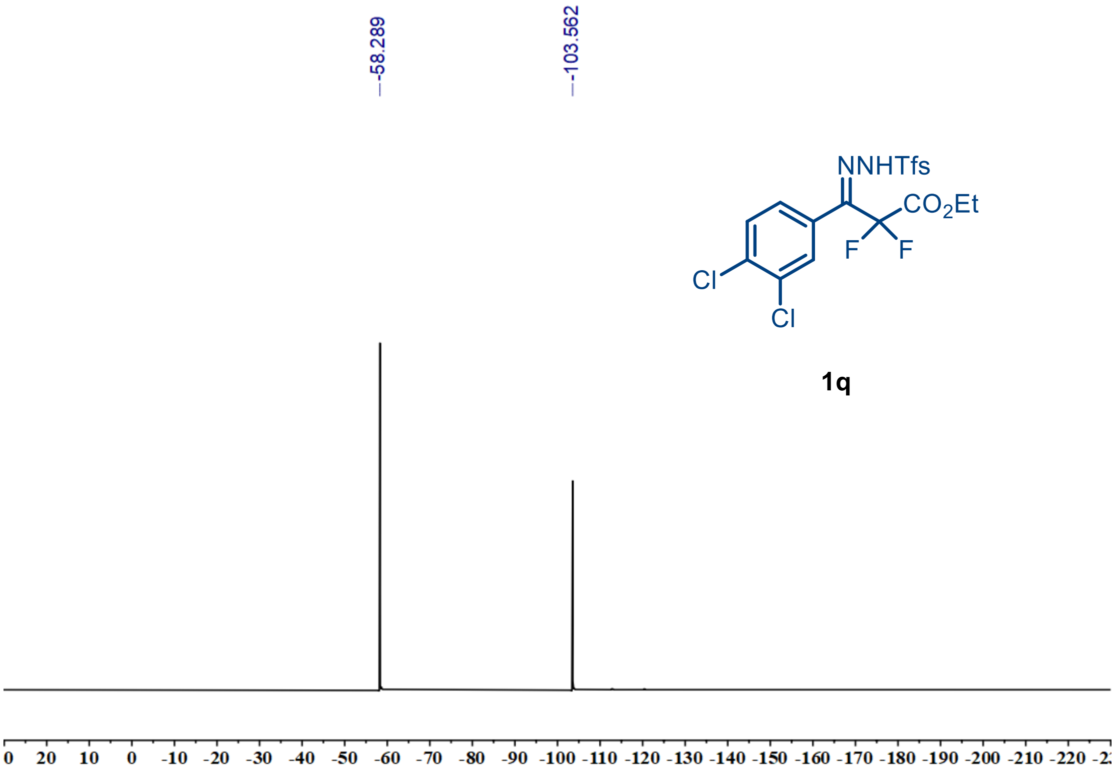

Figure S84, ^{19}F-NMR spectra copies of 1q related to scheme 2 and 3.

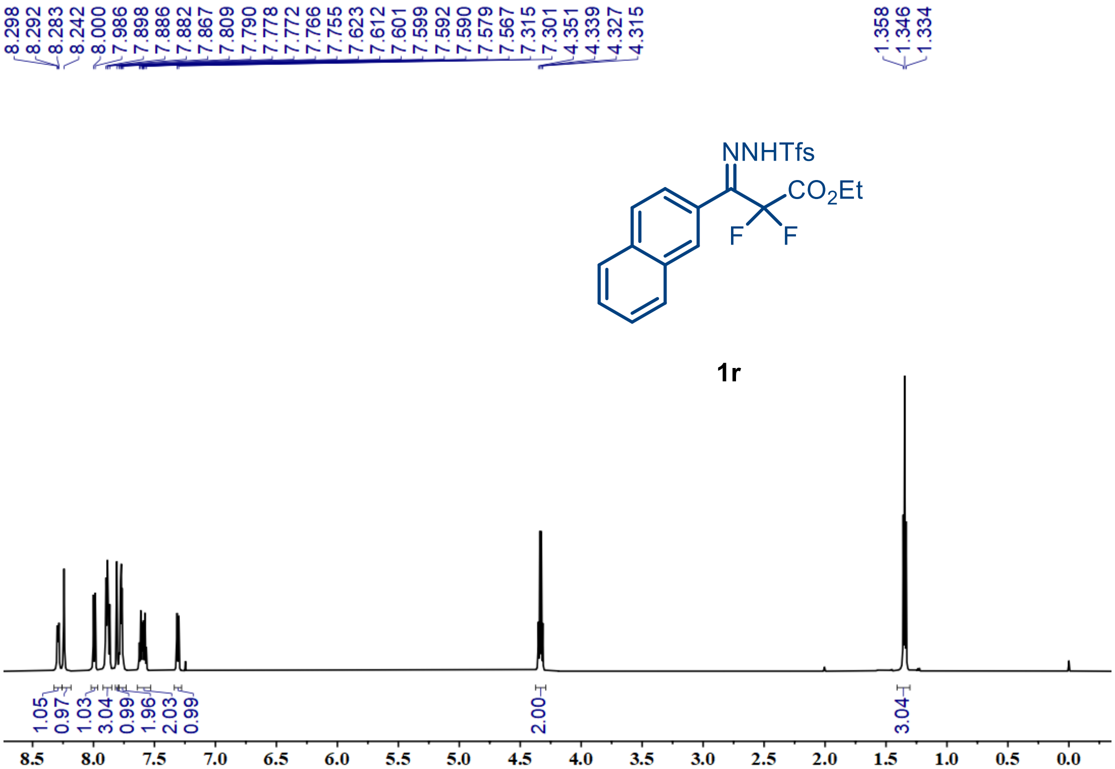

Figure S85, ^{1}H-NMR spectra copies of 1r related to scheme 2 and 3.

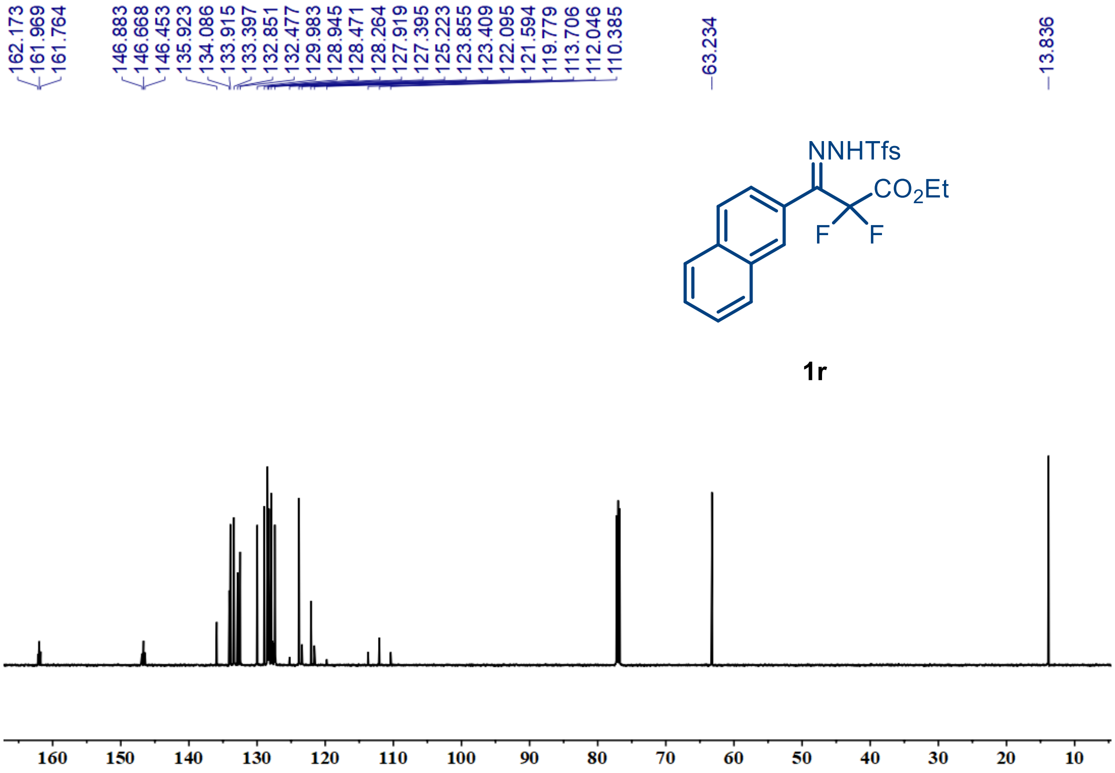

Figure S86, ^{13}C-NMR spectra copies of 1f related to scheme 2 and 3.

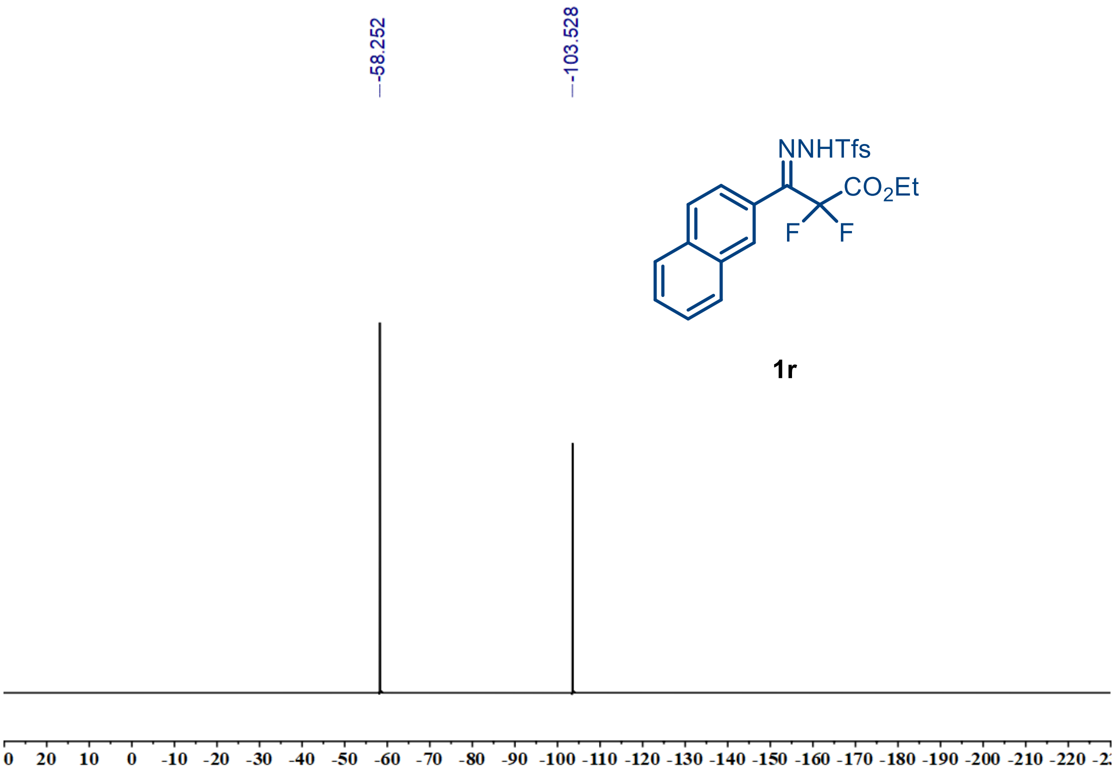

1r

Figure S87, ^{19}F-NMR spectra copies of 1r related to scheme 2 and 3.

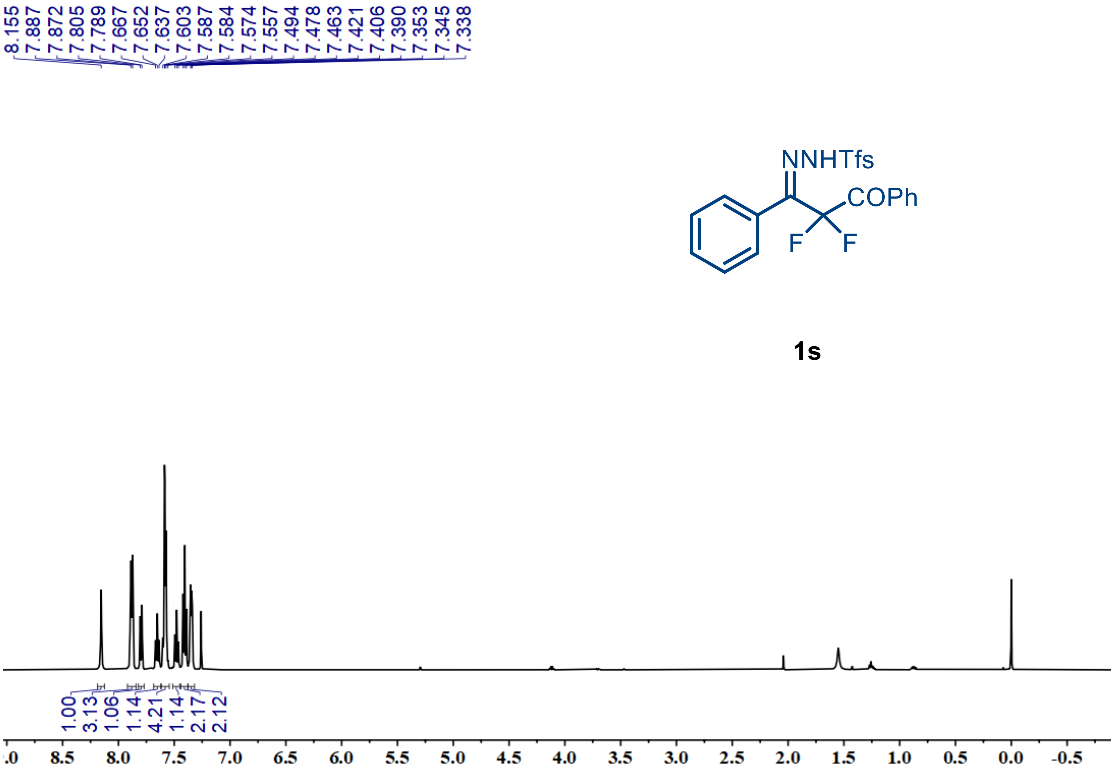

1s

Figure S88, ^{1}H-NMR spectra copies of 1s related to scheme 2 and 3.

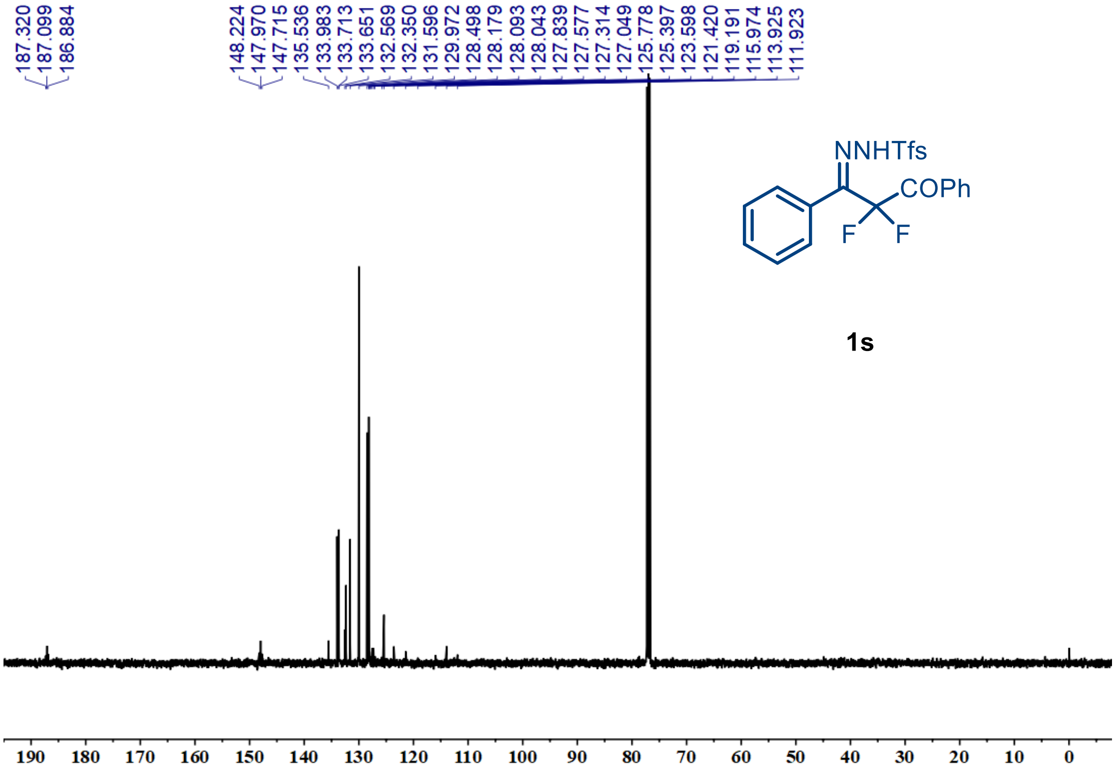

Figure S89, ^{13}C-NMR spectra copies of 1s related to scheme 2 and 3.

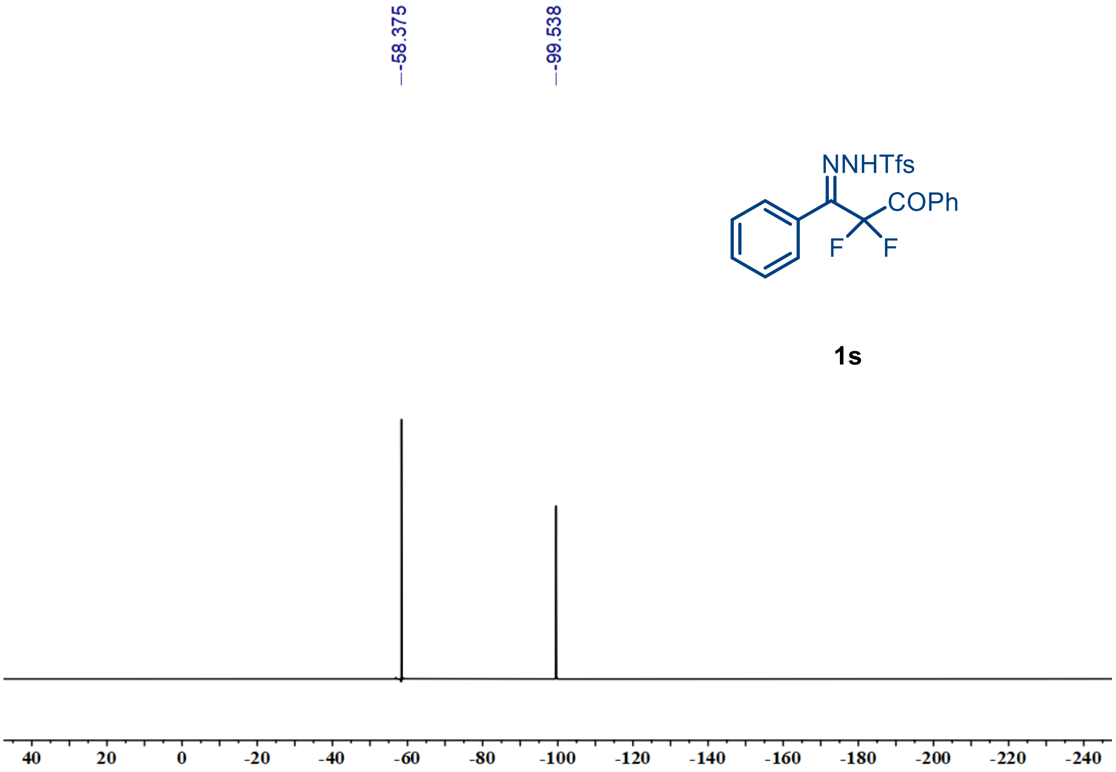

Figure S90, ^{19}F-NMR spectra copies of 1s related to scheme 2 and 3.

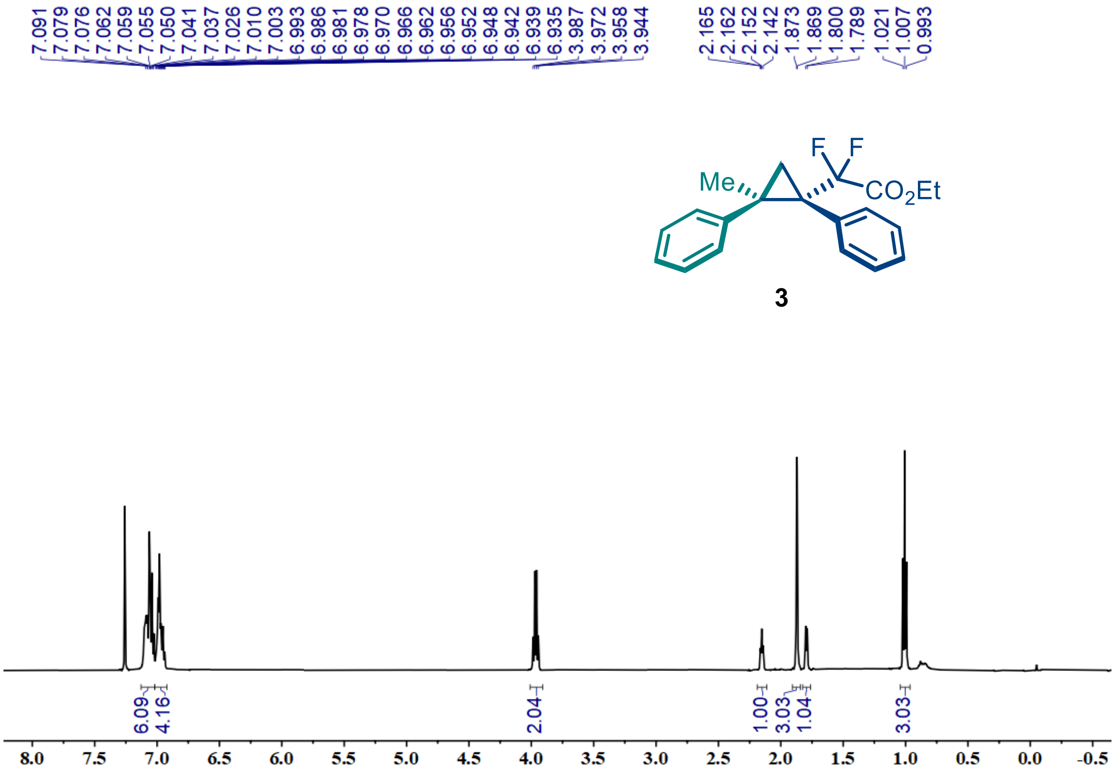

Figure S91, ^{1}H-NMR spectra copies of 3 related to scheme 2 and 3.

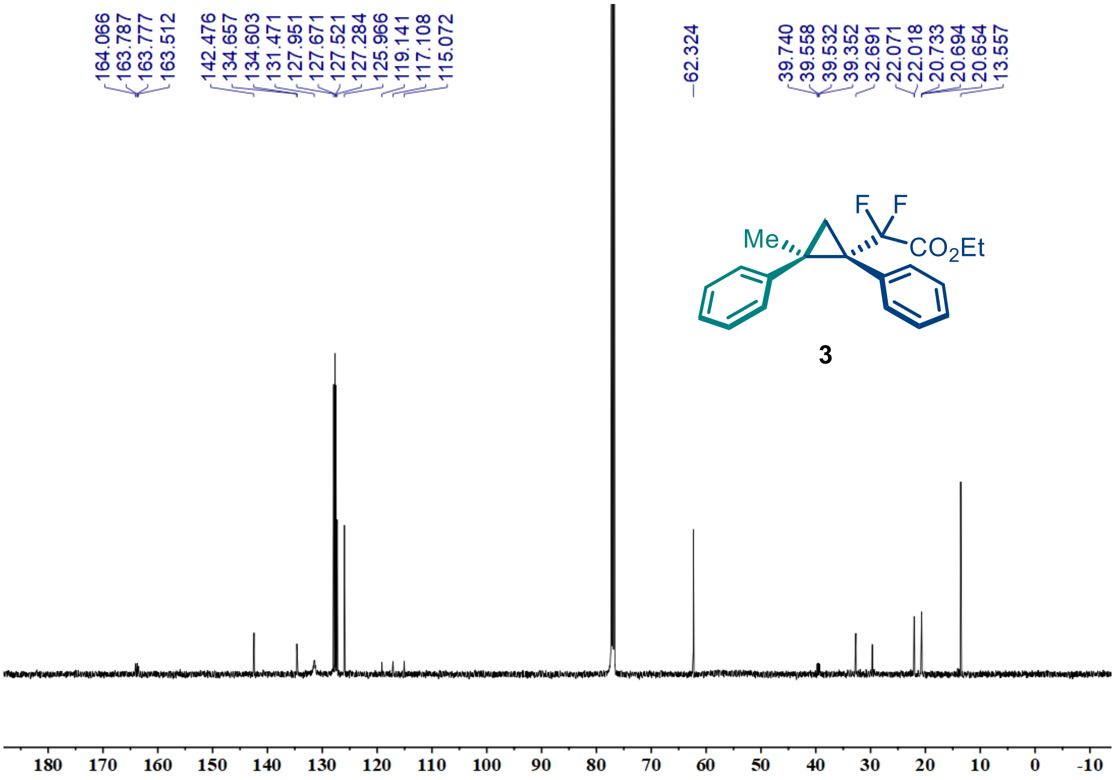

Figure S92, ^{13}C-NMR spectra copies of 3 related to scheme 2 and 3.

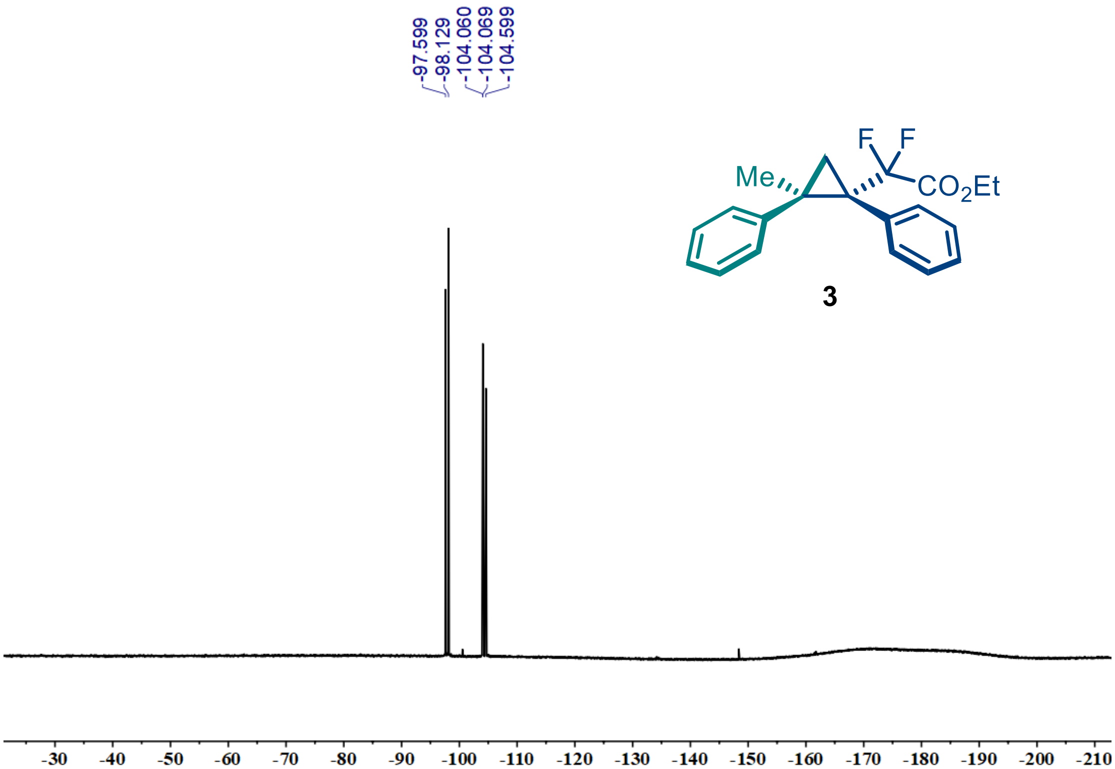

Figure S93, ^{19}F-NMR spectra copies of 3 related to scheme 2 and 3.

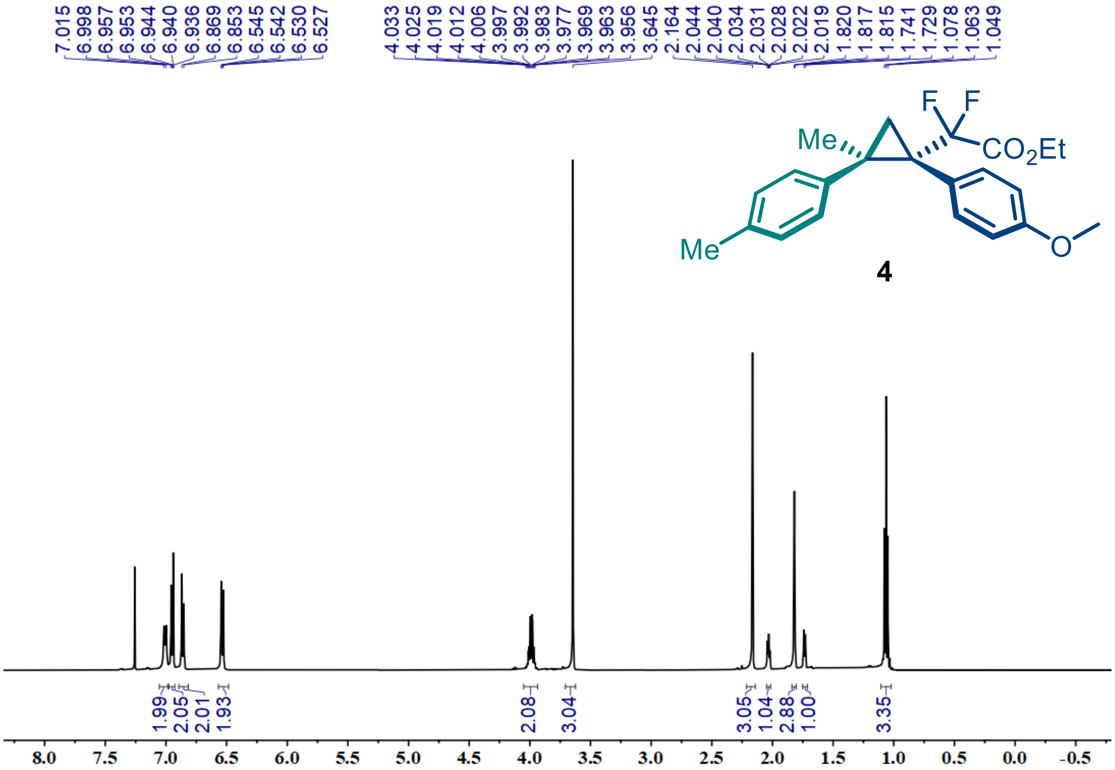

Figure S94, ^{1}H-NMR spectra copies of 4 related to scheme 2 and 3.

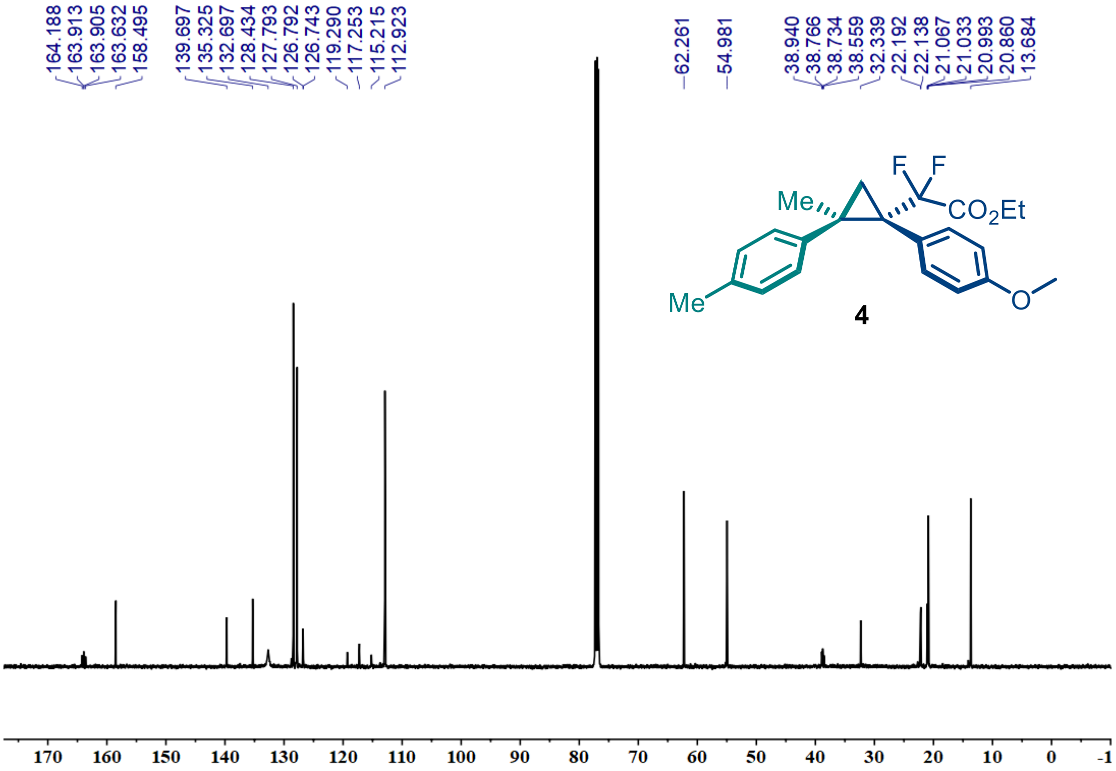

Figure S95, ^{13}C-NMR spectra copies of 4 related to scheme 2 and 3.

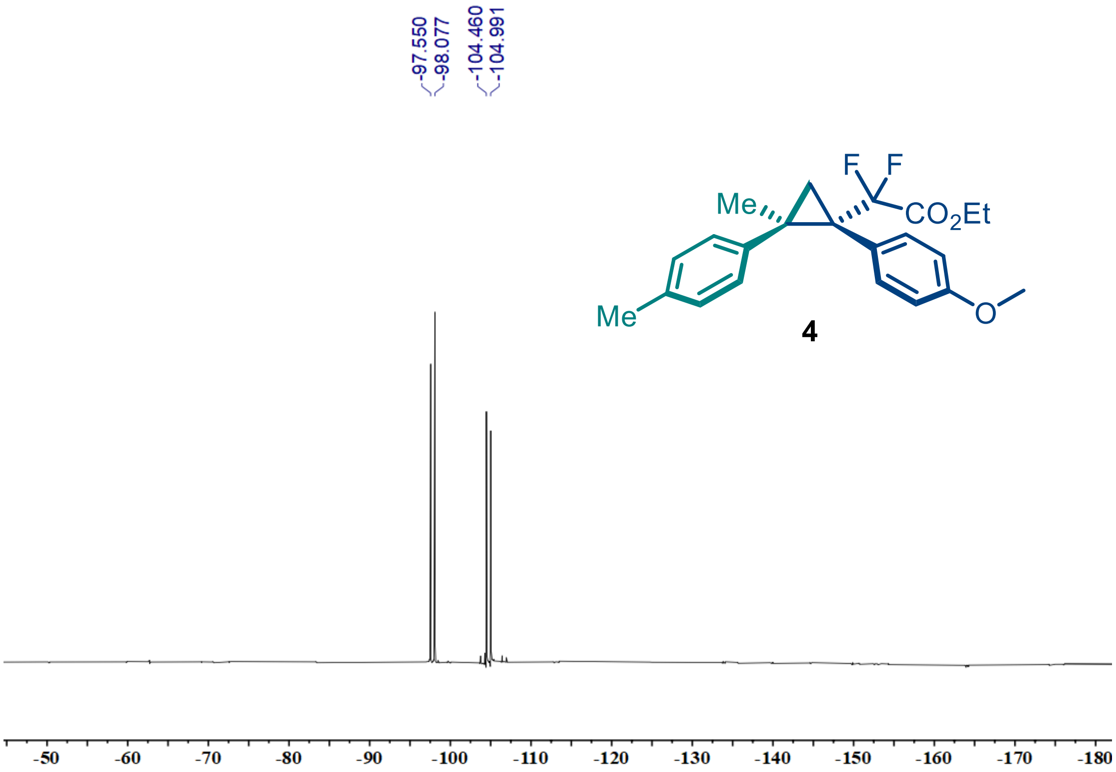

Figure S96, ^{19}F-NMR spectra copies of 4 related to scheme 2 and 3.

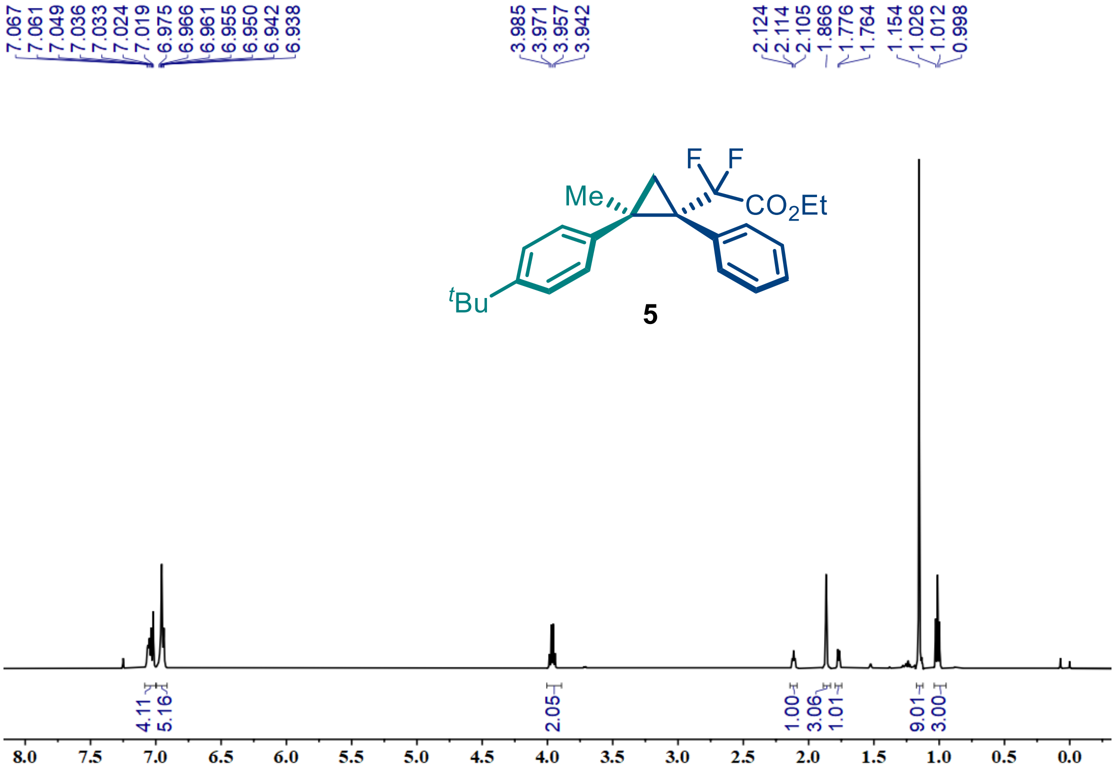

Figure S97, ^{1}H-NMR spectra copies of 5 related to scheme 2 and 3.

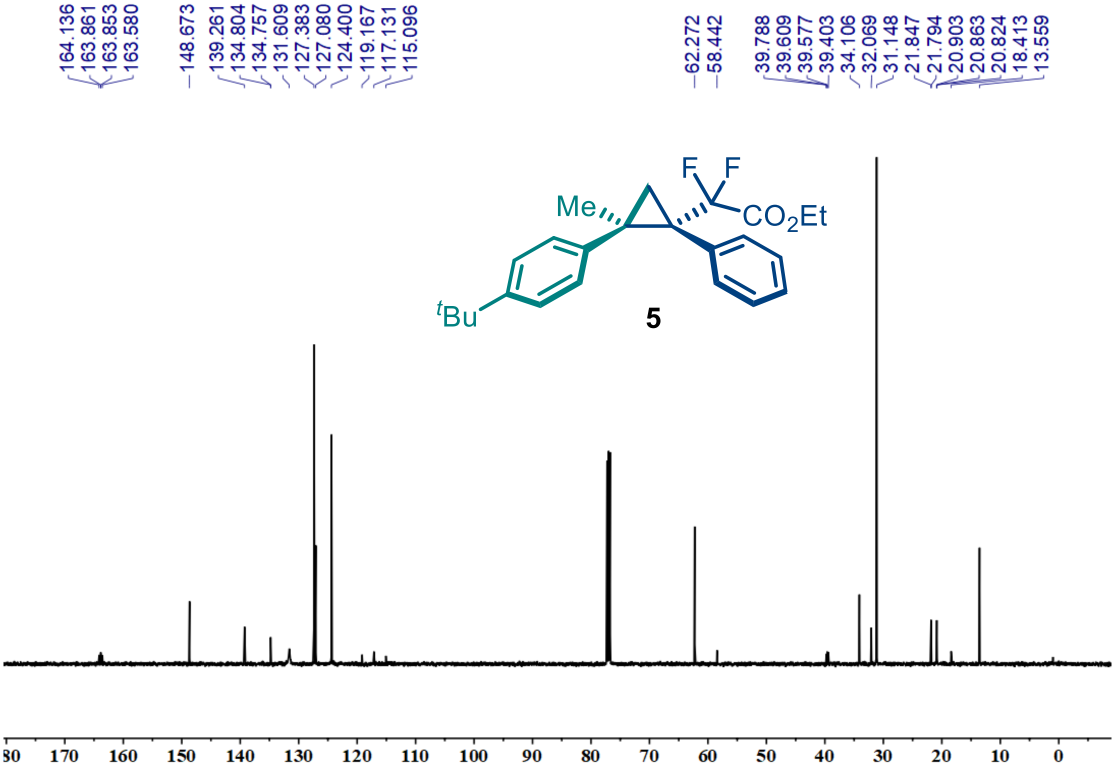

Figure S98, ^{13}C-NMR spectra copies of 5 related to scheme 2 and 3.

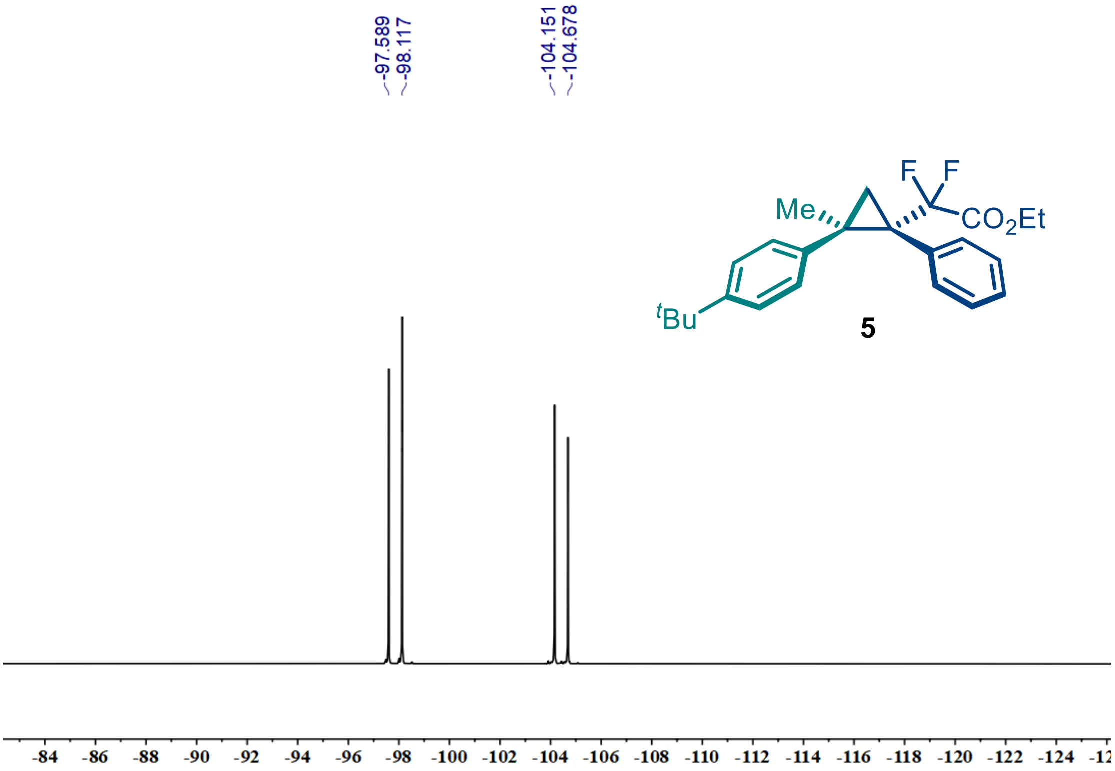

Figure S99, ^{19}F-NMR spectra copies of 5 related to scheme 2 and 3.

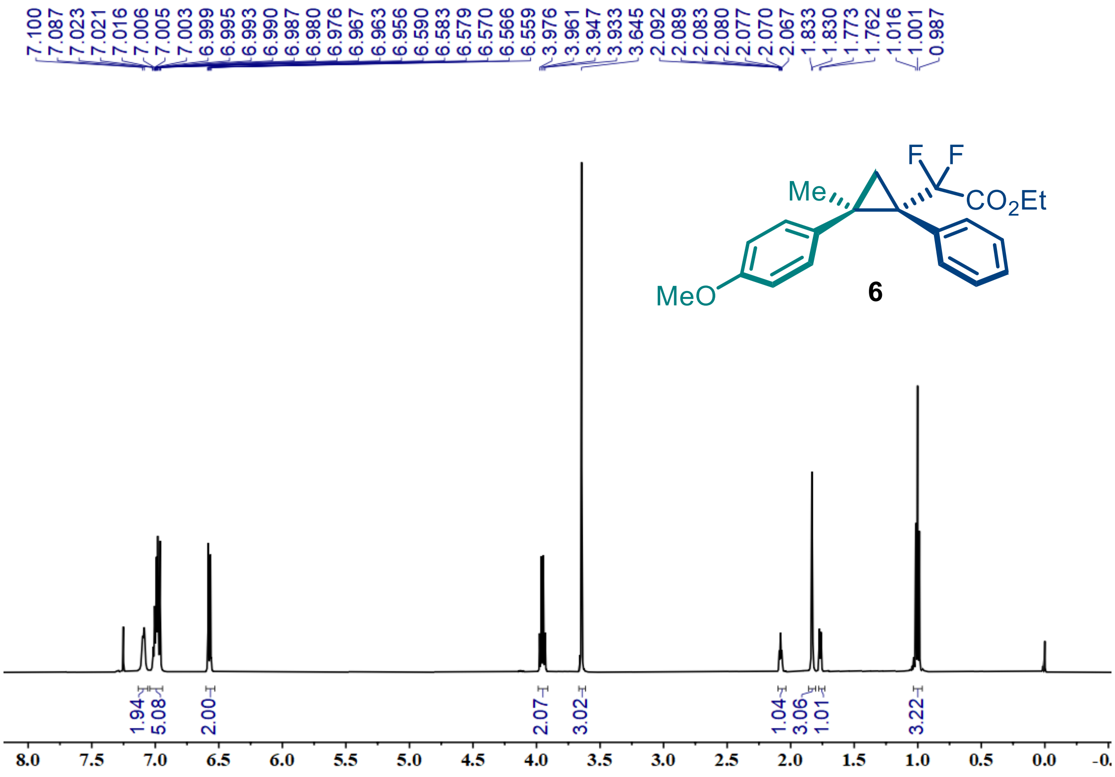

Figure S100, ^{1}H-NMR spectra copies of 6 related to scheme 2 and 3.

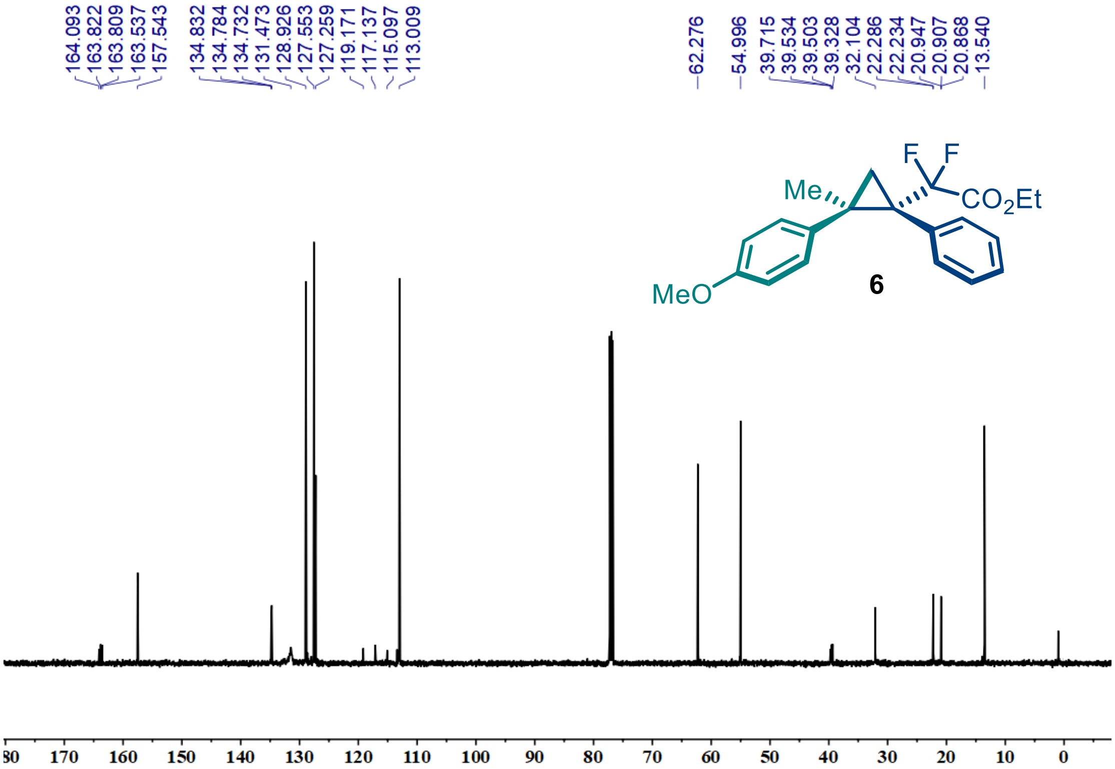

Figure S101, ^{13}C-NMR spectra copies of 6 related to scheme 2 and 3.

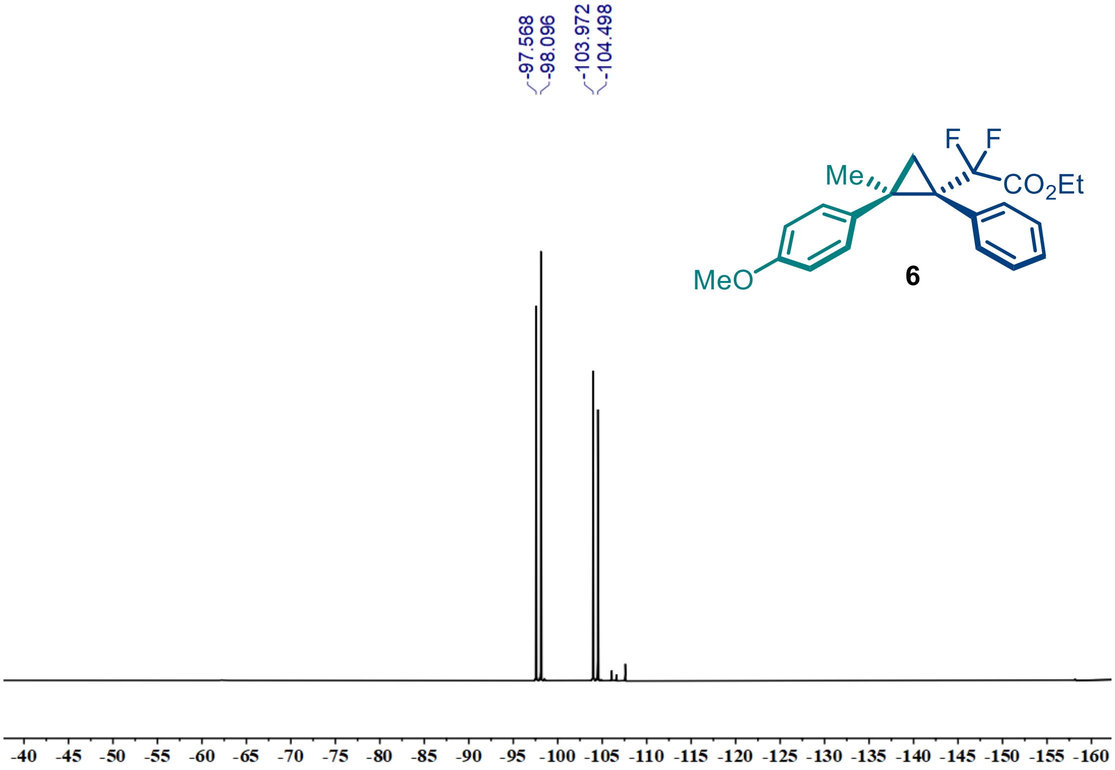

Figure S102, ^{19}F-NMR spectra copies of 6 related to scheme 2 and 3.

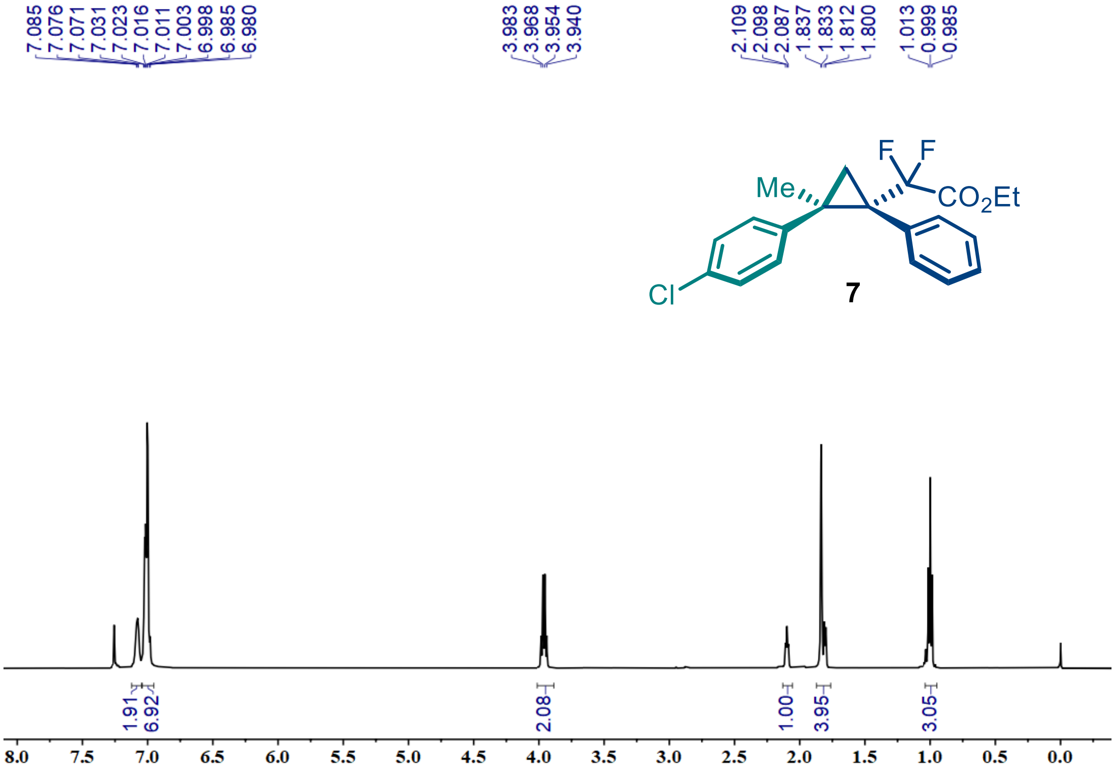

Figure S103, ^{1}H-NMR spectra copies of 7 related to scheme 2 and 3.

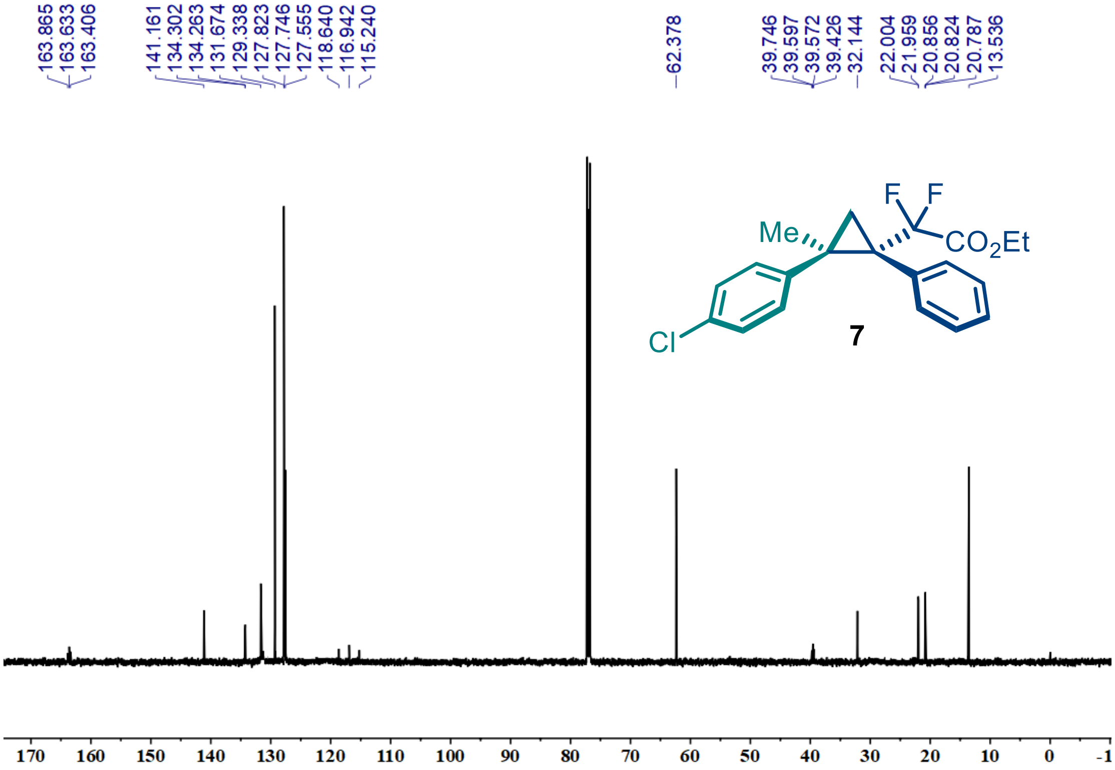

Figure S104, ^{13}C-NMR spectra copies of 7 related to scheme 2 and 3.

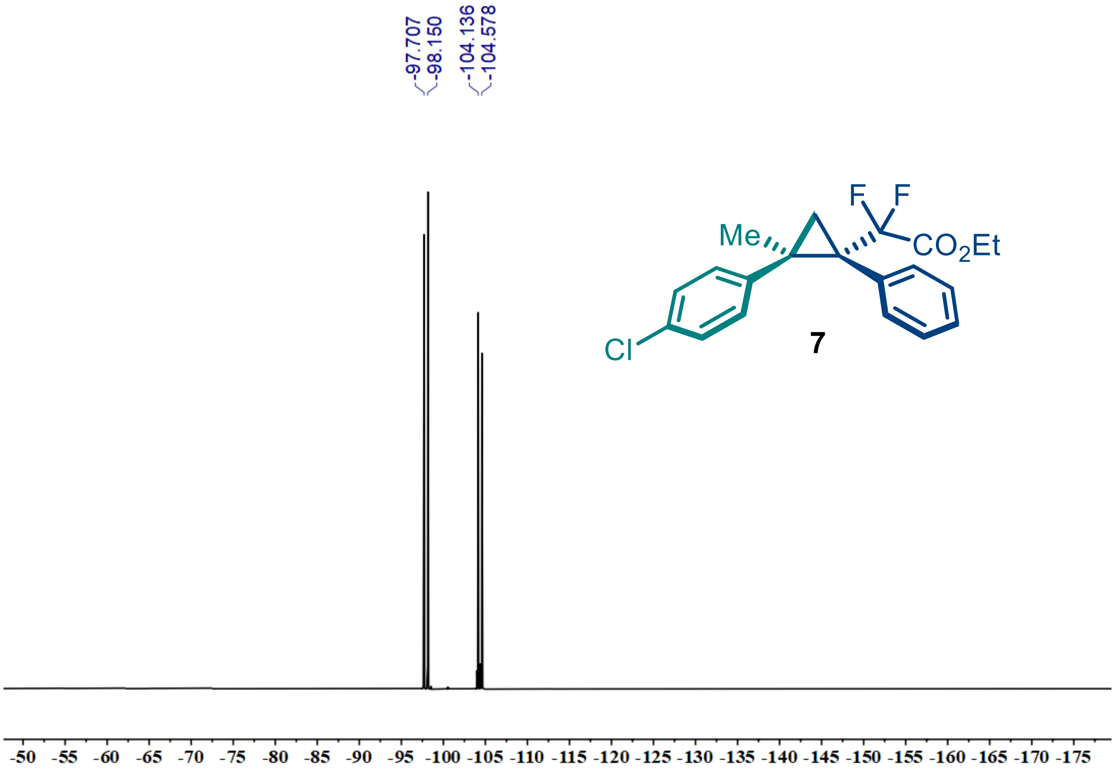

Figure S105, ^{19}F-NMR spectra copies of 7 related to scheme 2 and 3.

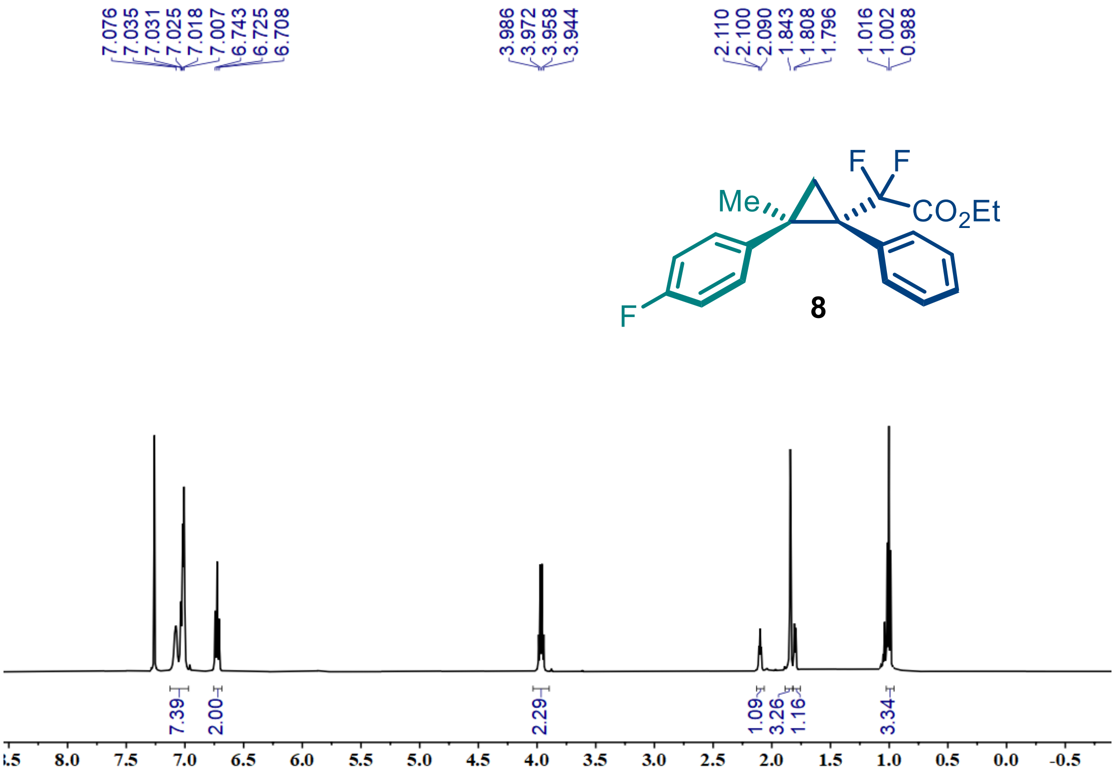

Figure S106, ^{1}H-NMR spectra copies of 8 related to scheme 2 and 3.

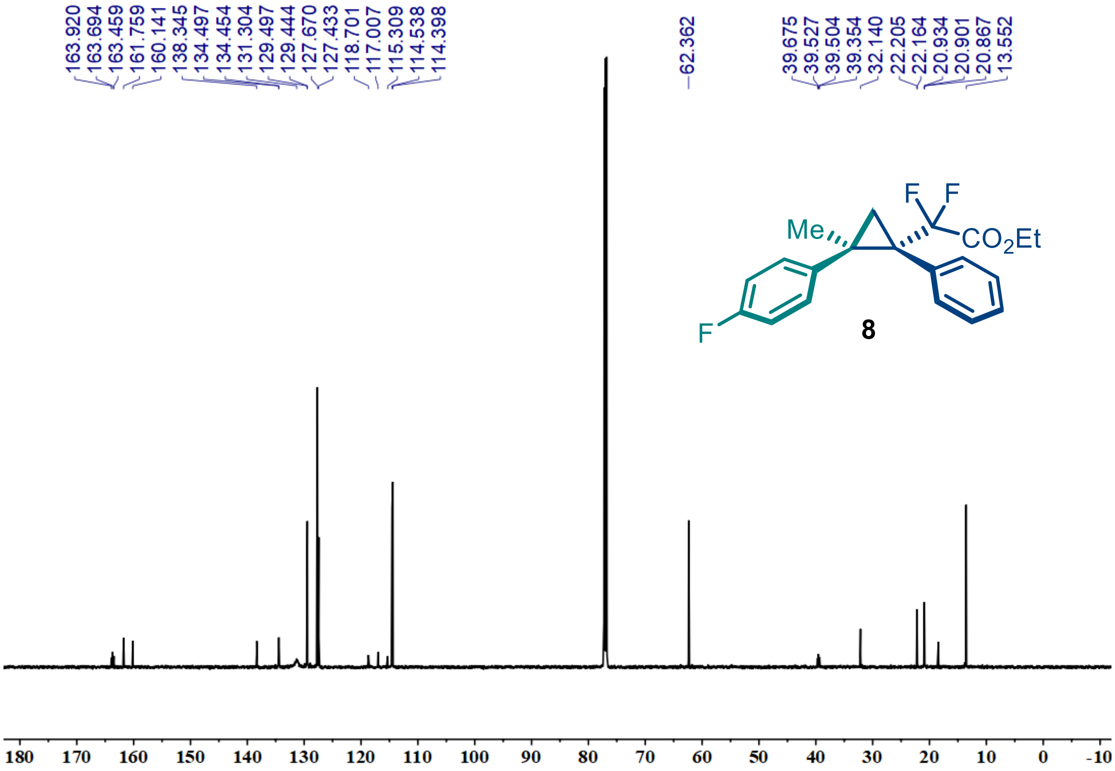

Figure S107, ^{13}C-NMR spectra copies of 8 related to scheme 2 and 3.

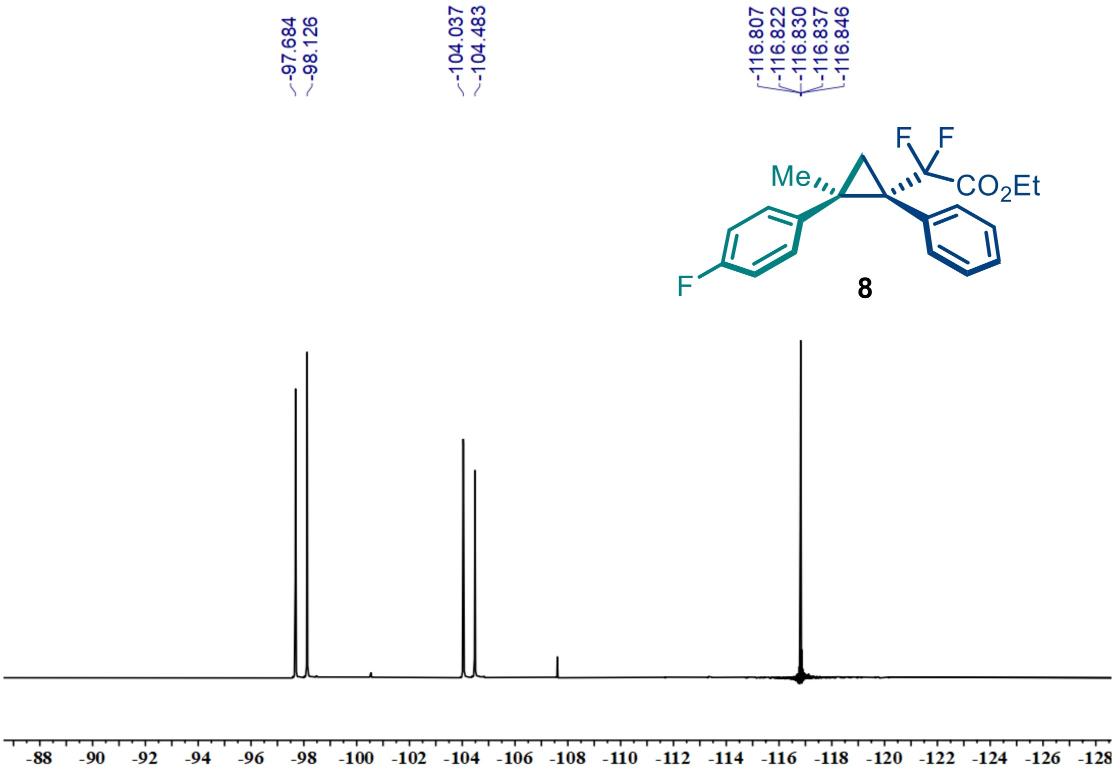

Figure S108, ^{19}F-NMR spectra copies of 8 related to scheme 2 and 3.

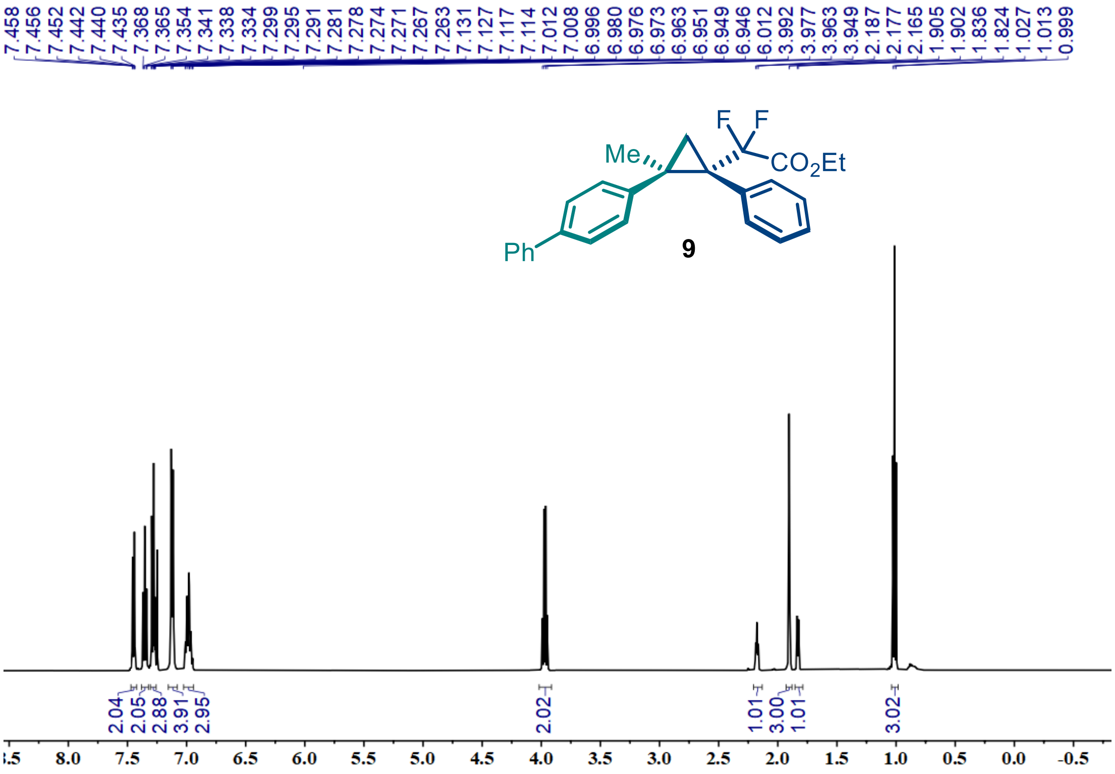

Figure S109, ^{1}H-NMR spectra copies of 9 related to scheme 2 and 3.

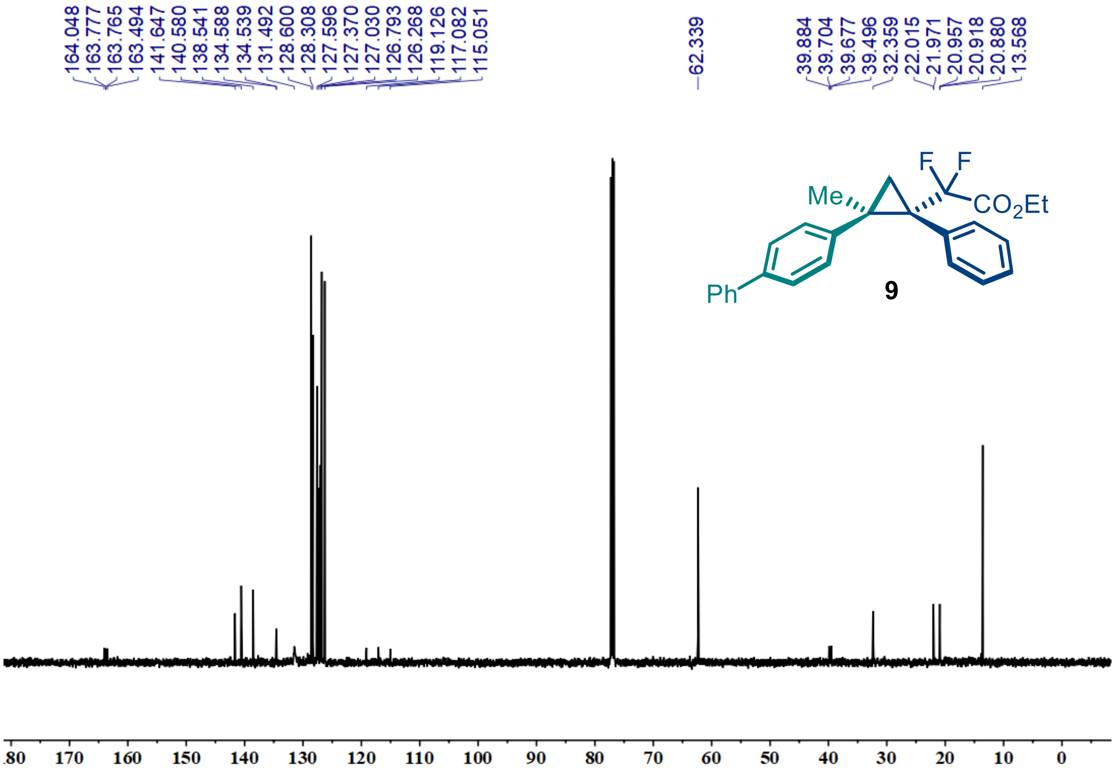

Figure S110, ^{13}C-NMR spectra copies of 9 related to scheme 2 and 3.

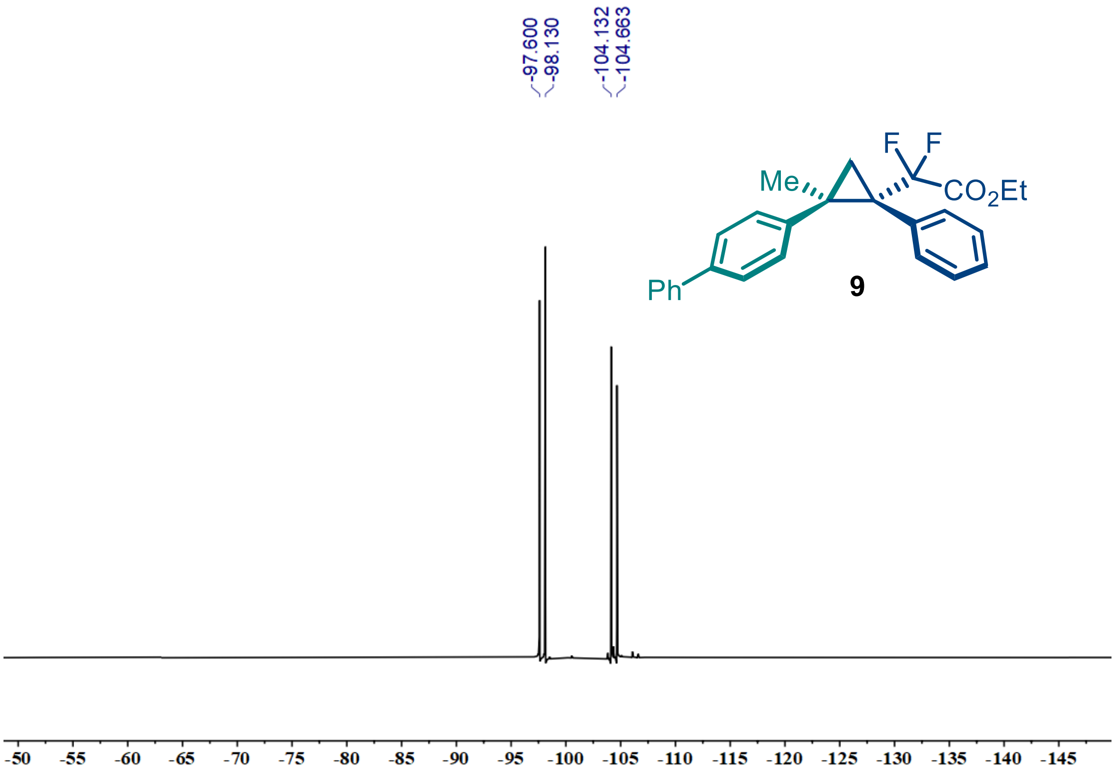

Figure S111, ^{19}F-NMR spectra copies of 9 related to scheme 2 and 3.

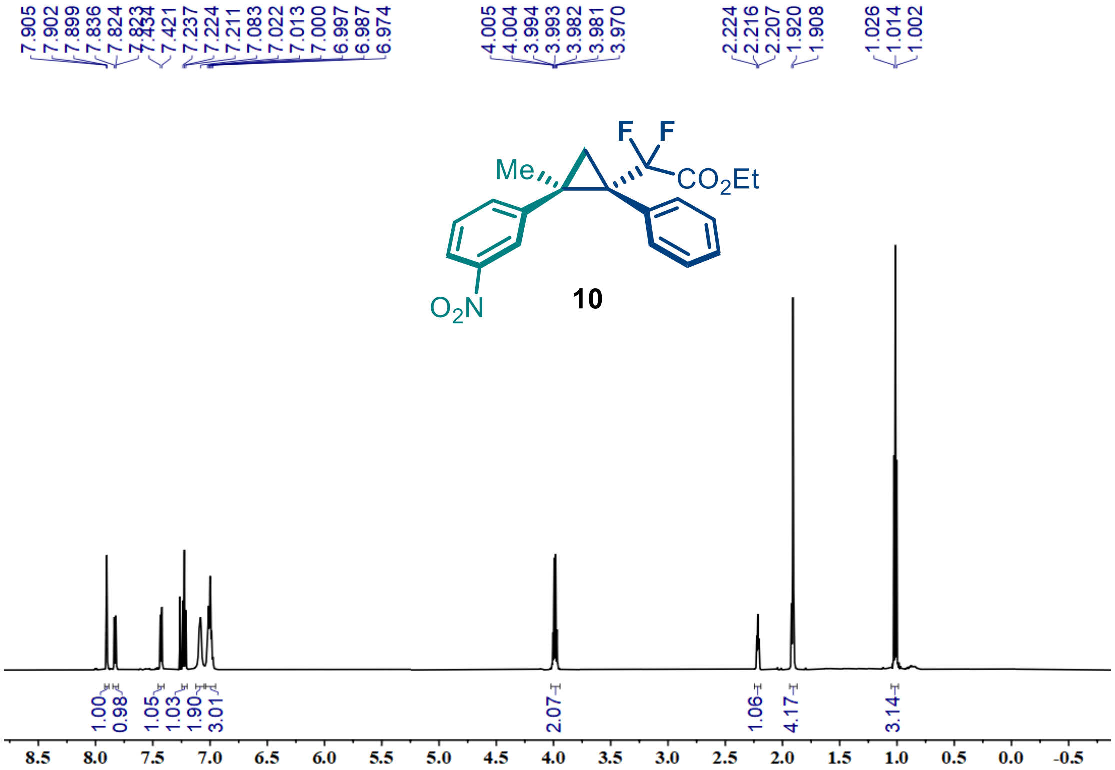

Figure S112, ^{1}H-NMR spectra copies of 10 related to scheme 2 and 3.

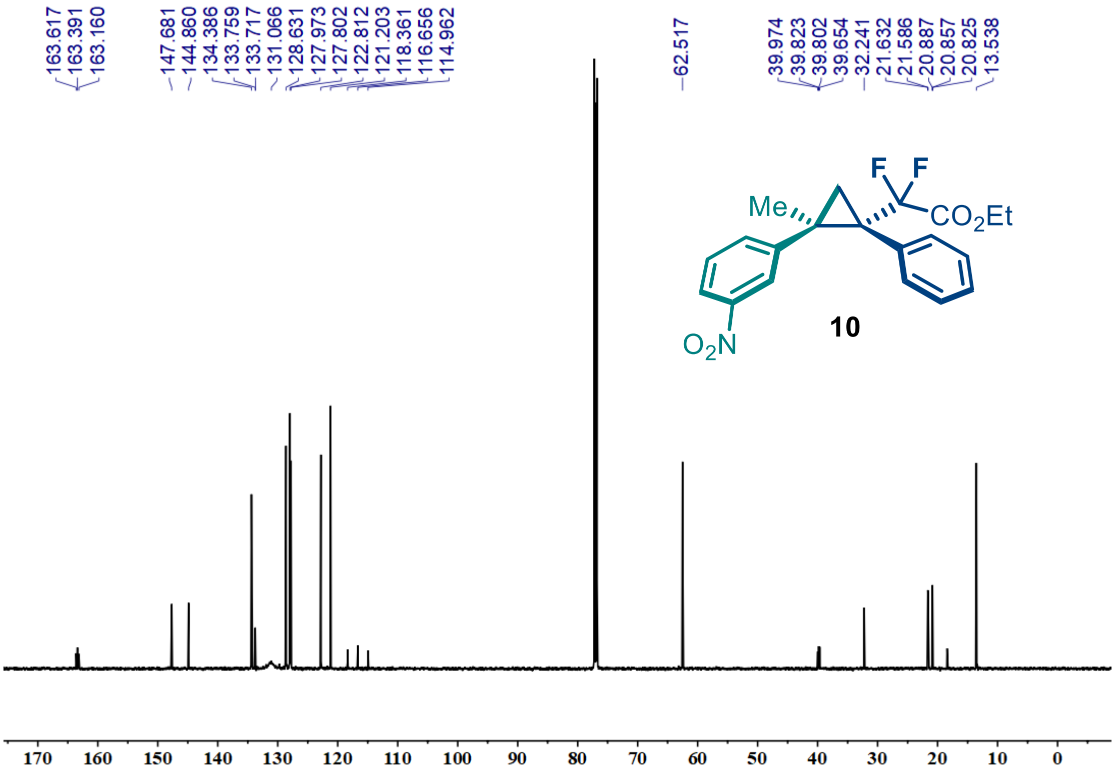

Figure S113, ^{13}C-NMR spectra copies of 10 related to scheme 2 and 3.

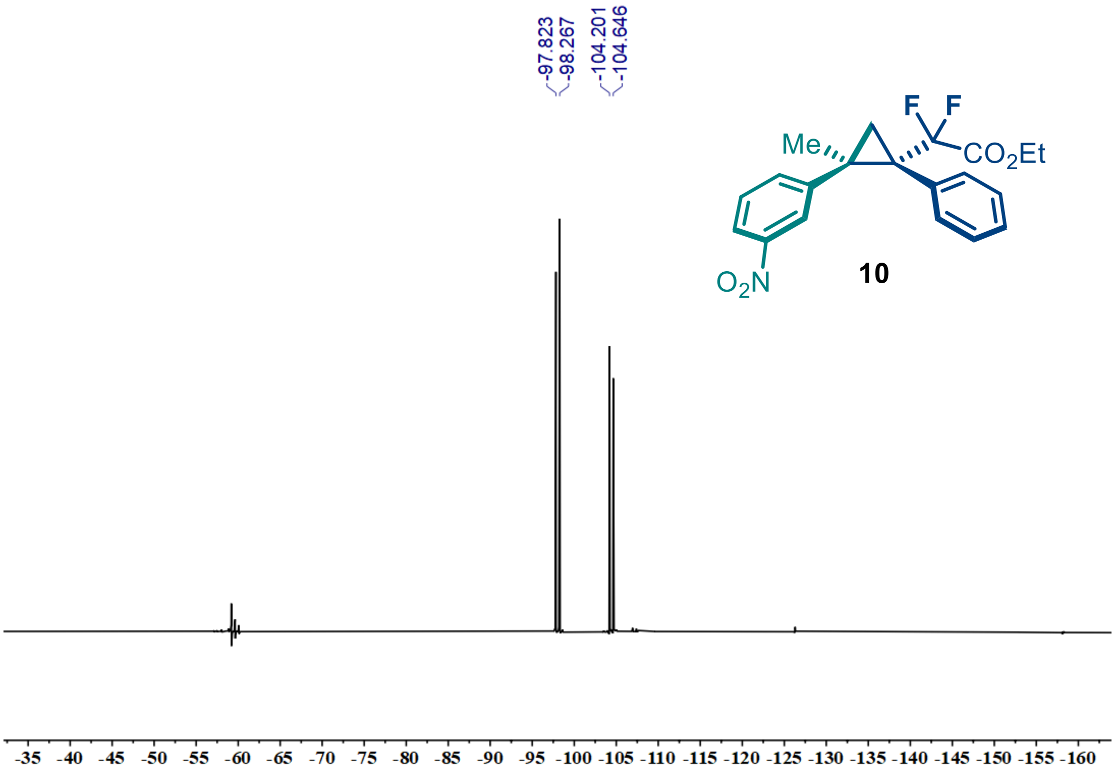

Figure S114, ^{19}F-NMR spectra copies of 10 related to scheme 2 and 3.

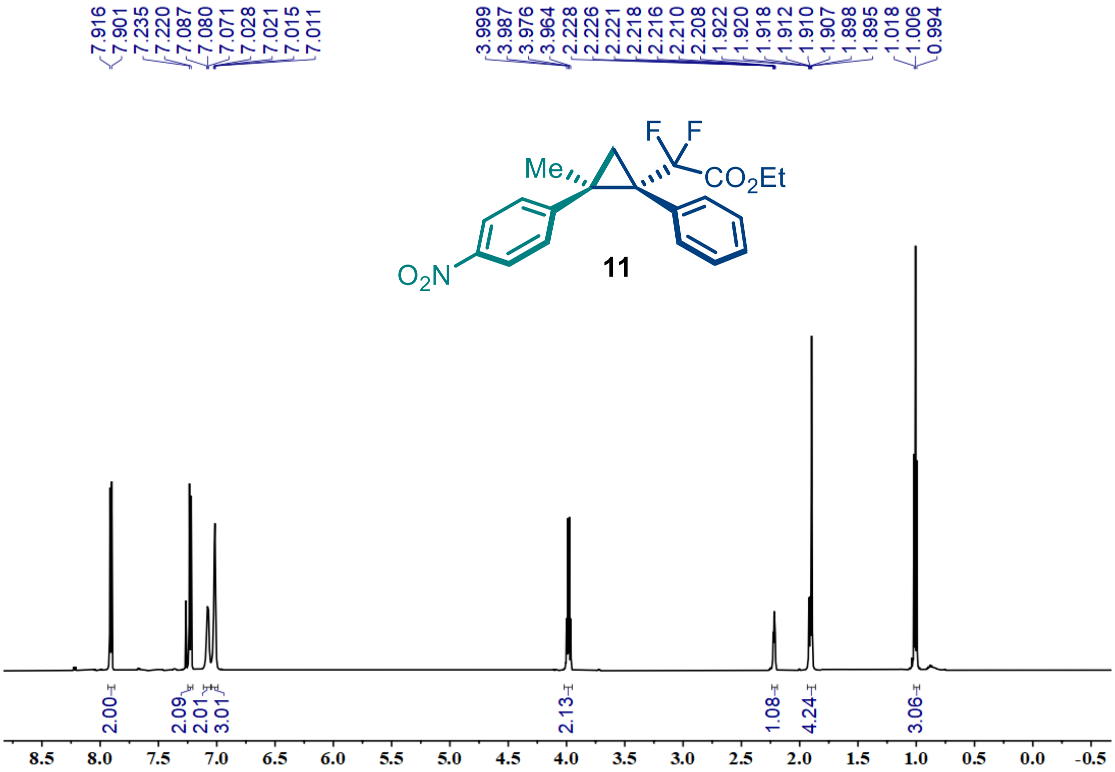

Figure S115, ^{1}H-NMR spectra copies of 11 related to scheme 2 and 3.

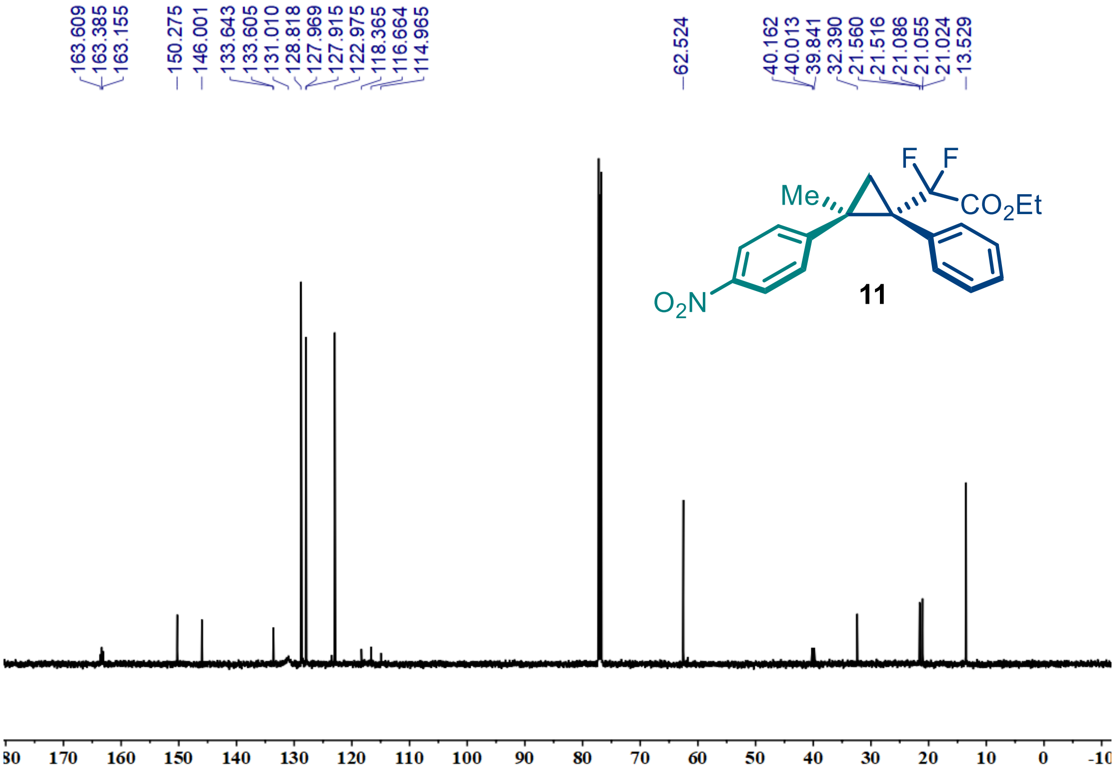

Figure S116, ^{13}C-NMR spectra copies of 11 related to scheme 2 and 3.

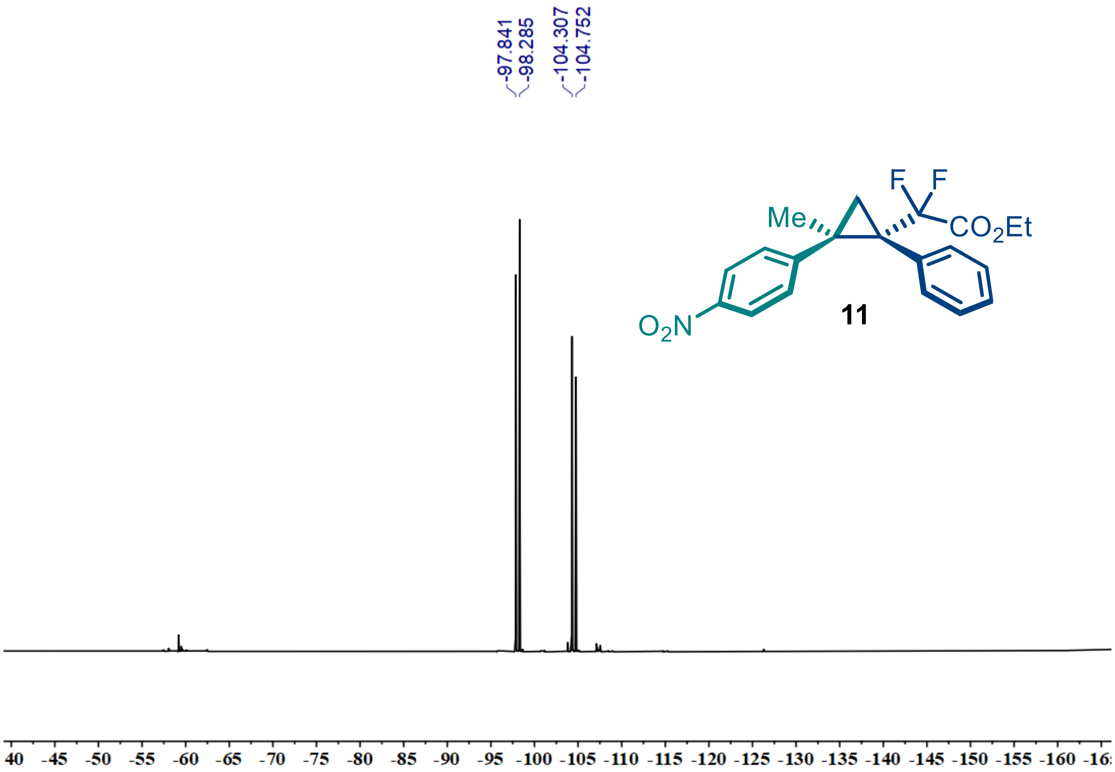

Figure S117, ^{19}F-NMR spectra copies of 11 related to scheme 2 and 3.

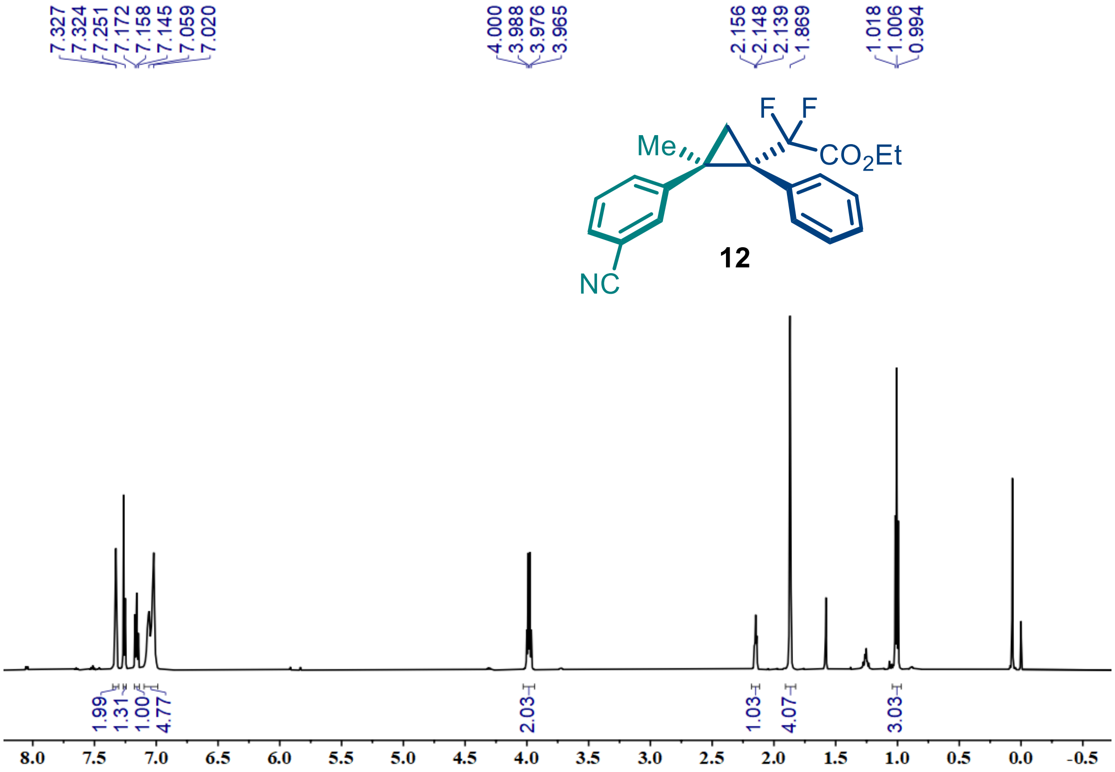

Figure S118, ^{1}H-NMR spectra copies of 12 related to scheme 2 and 3.

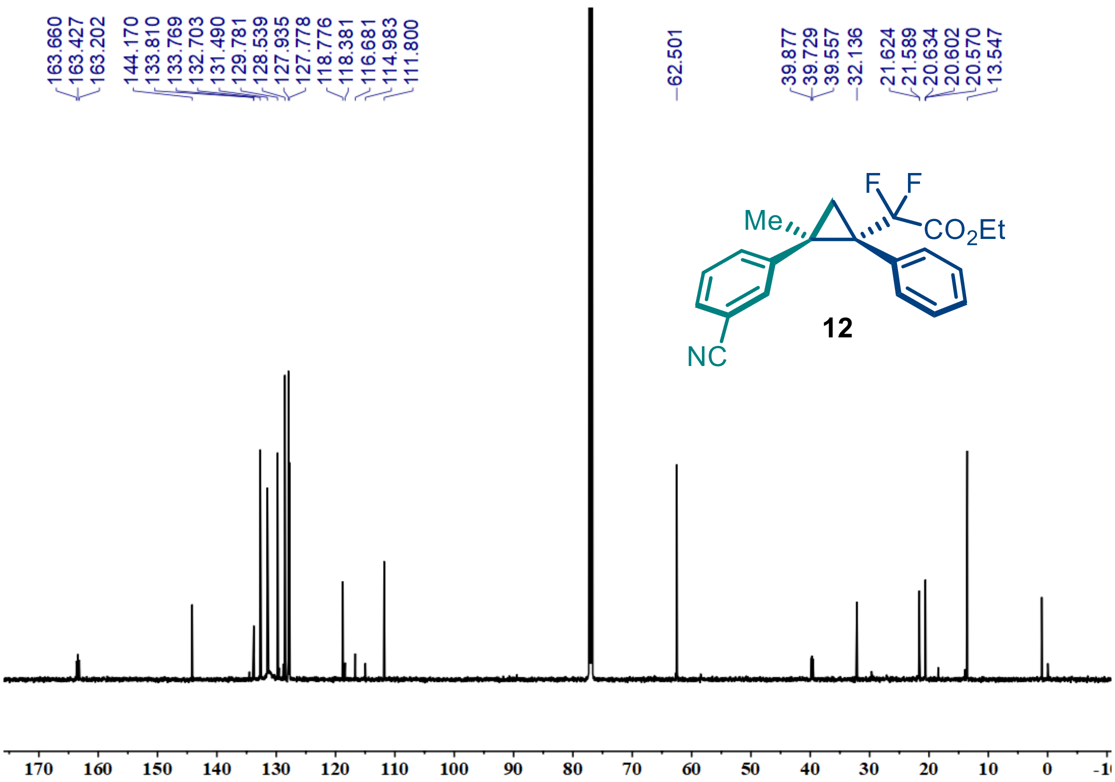

Figure S119, ^{13}C-NMR spectra copies of 12 related to scheme 2 and 3.

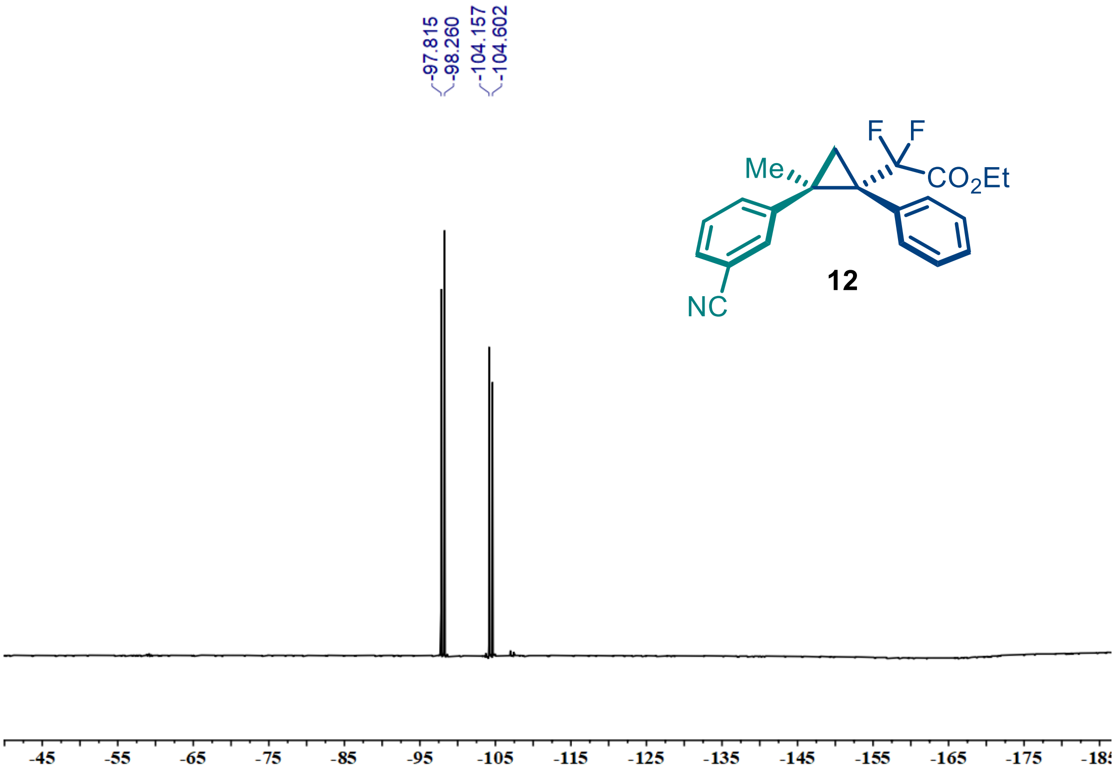

Figure S120, ^{19}F-NMR spectra copies of 12 related to scheme 2 and 3.

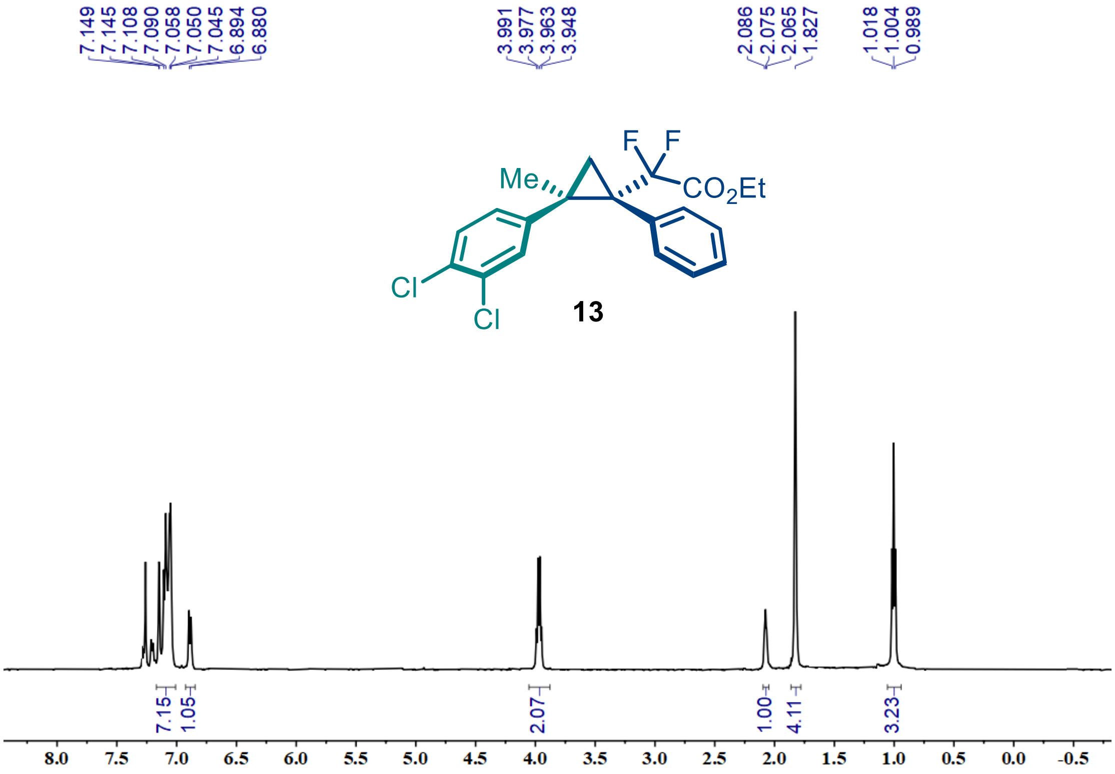

Figure S121, ^{1}H-NMR spectra copies of 13 related to scheme 2 and 3.

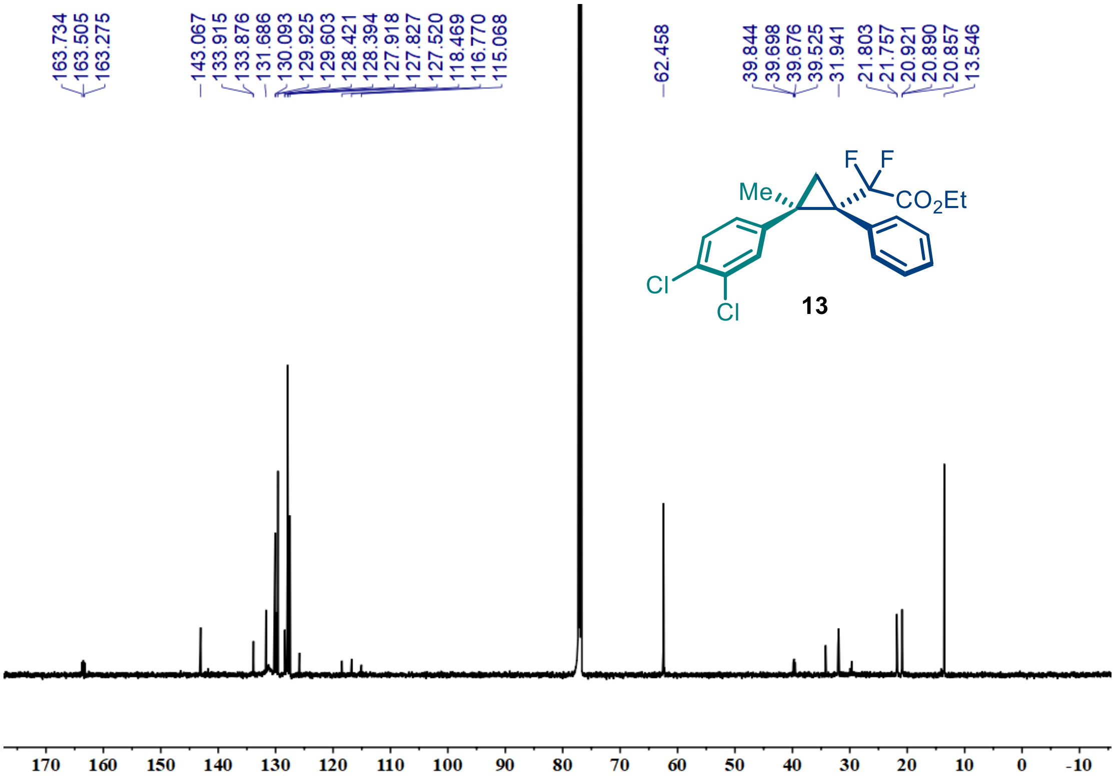

Figure S122, ^{13}C-NMR spectra copies of 13 related to scheme 2 and 3.

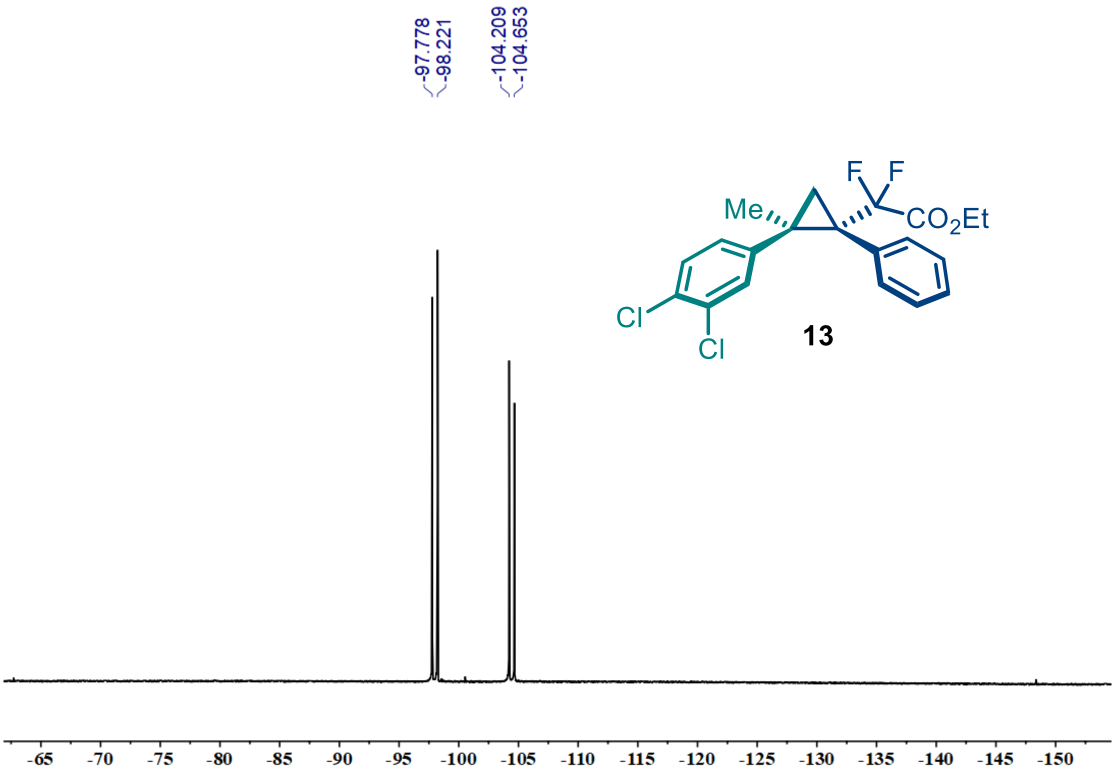

Figure S123, ^{19}F-NMR spectra copies of 13 related to scheme 2 and 3.

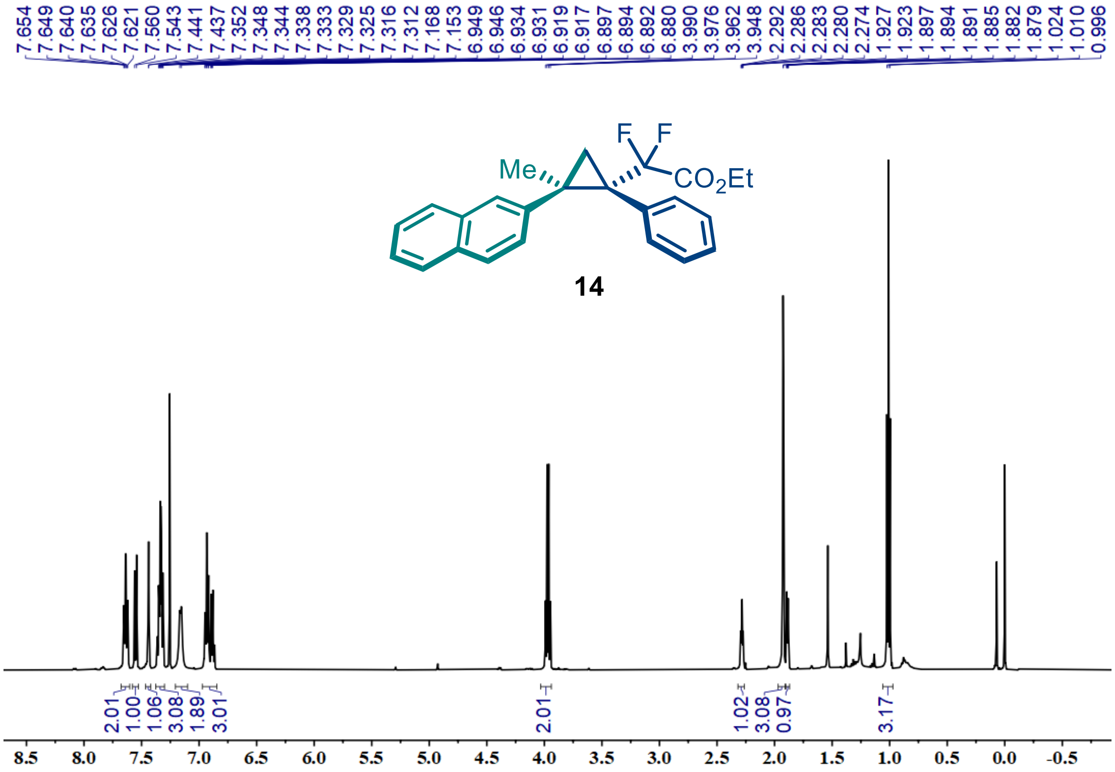

Figure S124, ^{1}H-NMR spectra copies of 14 related to scheme 2 and 3.

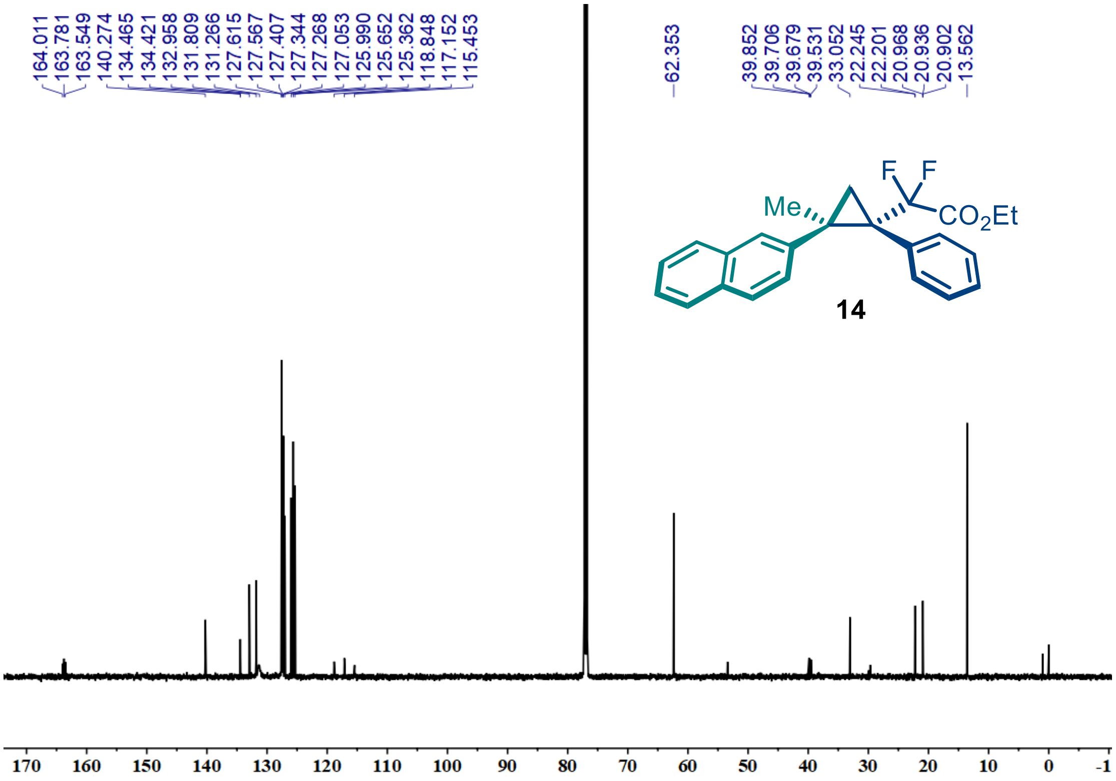

Figure S125, ^{13}C-NMR spectra copies of 14 related to scheme 2 and 3.

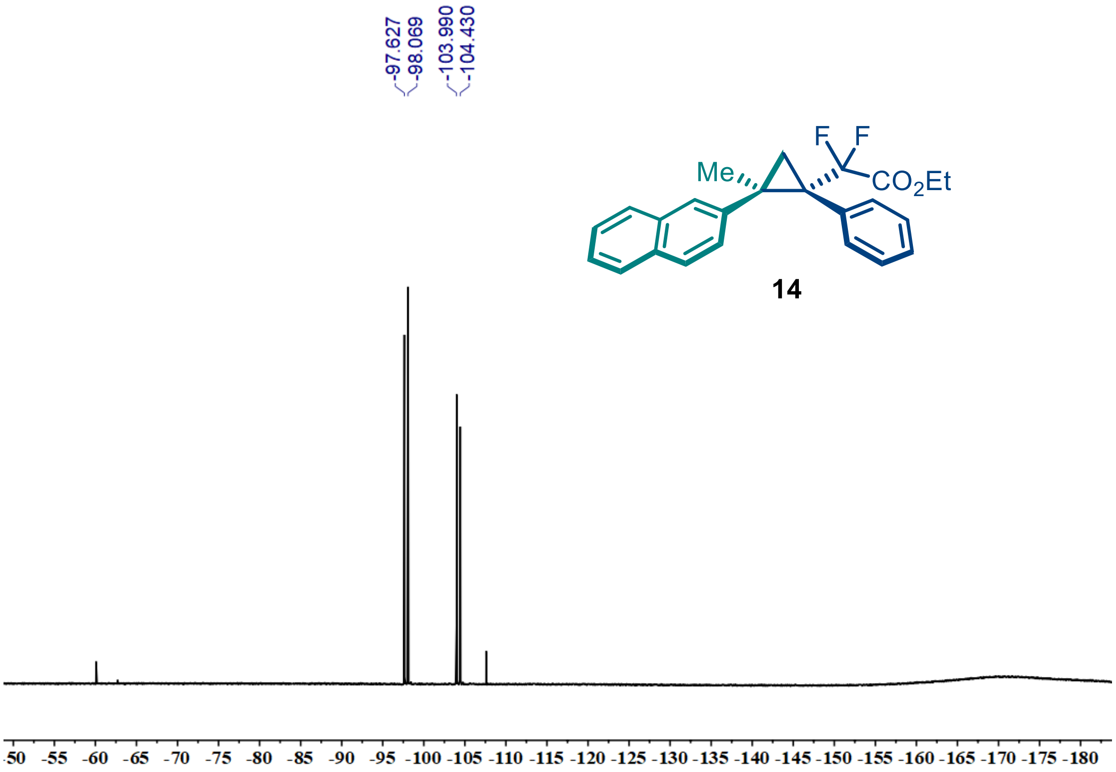

Figure S126, ^{19}F-NMR spectra copies of 14 related to scheme 2 and 3.

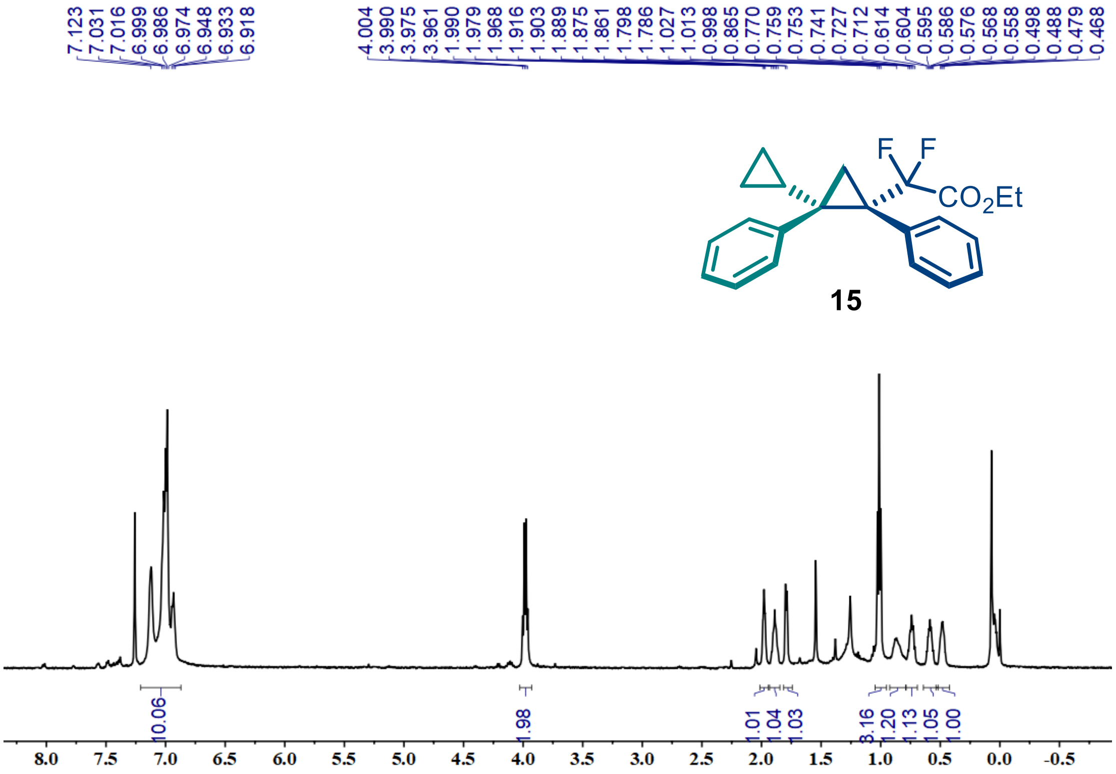

15

Figure S127, ^{1}H-NMR spectra copies of 15 related to scheme 2 and 3.

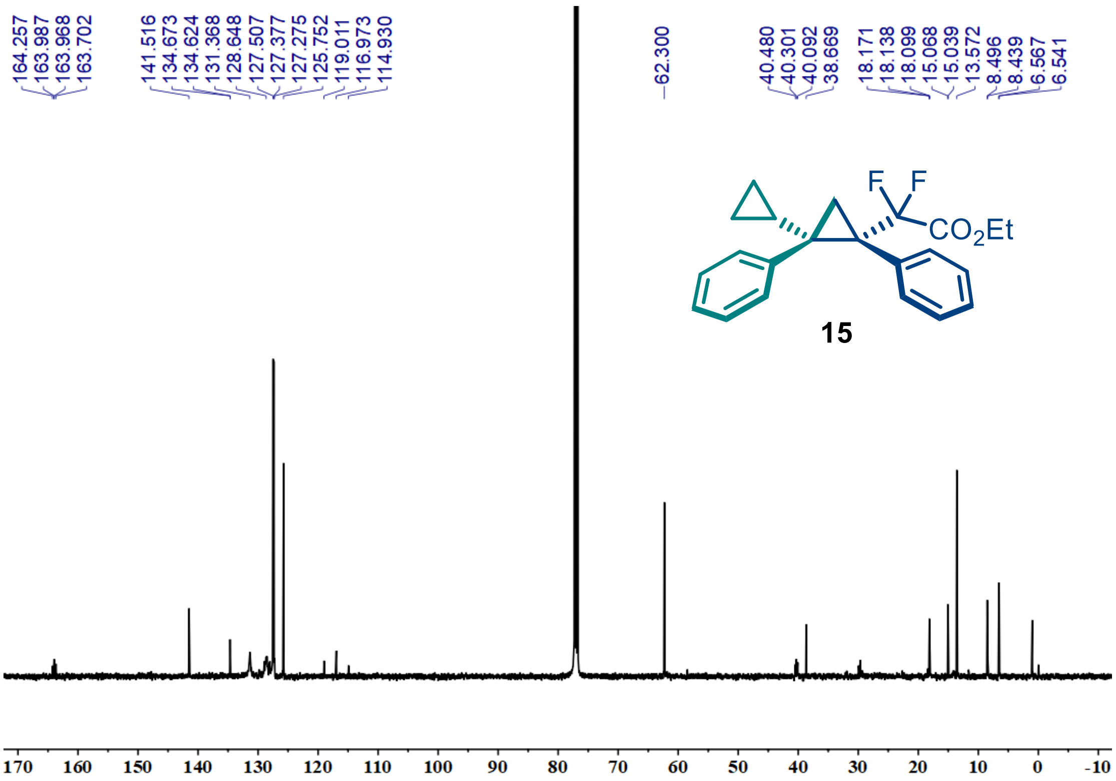

15

Figure S128, ^{13}C-NMR spectra copies of 15 related to scheme 2 and 3.

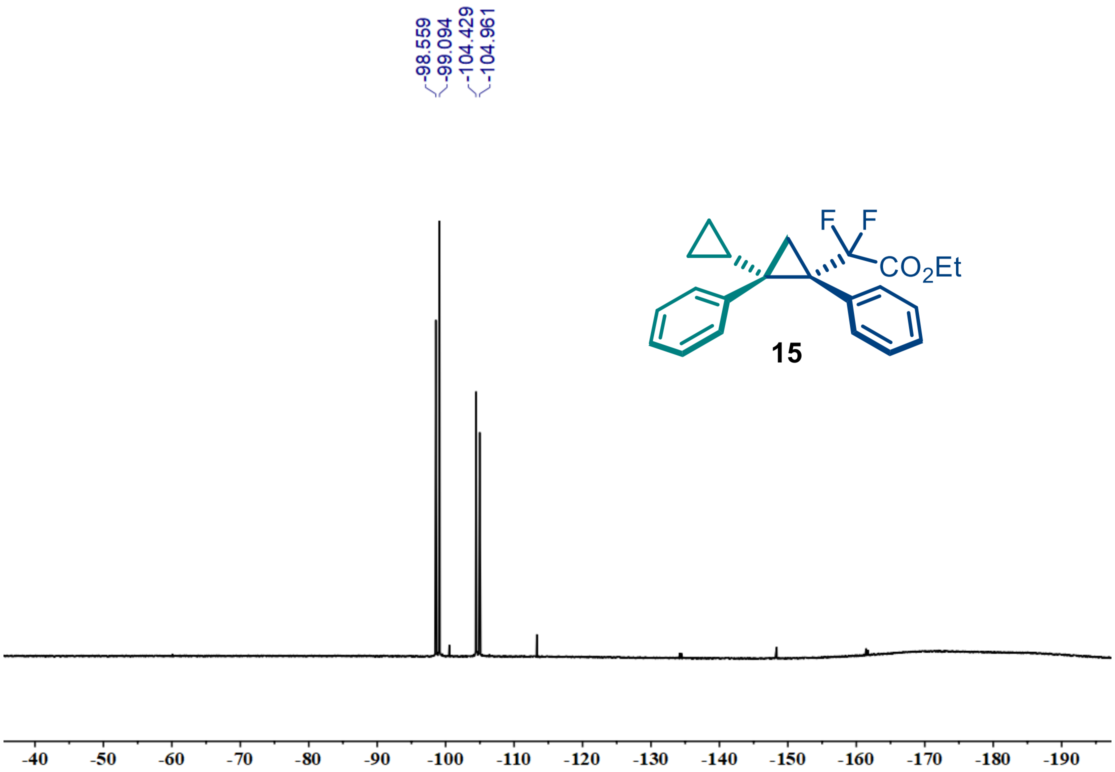

Figure S129, ^{19}F-NMR spectra copies of 15 related to scheme 2 and 3.

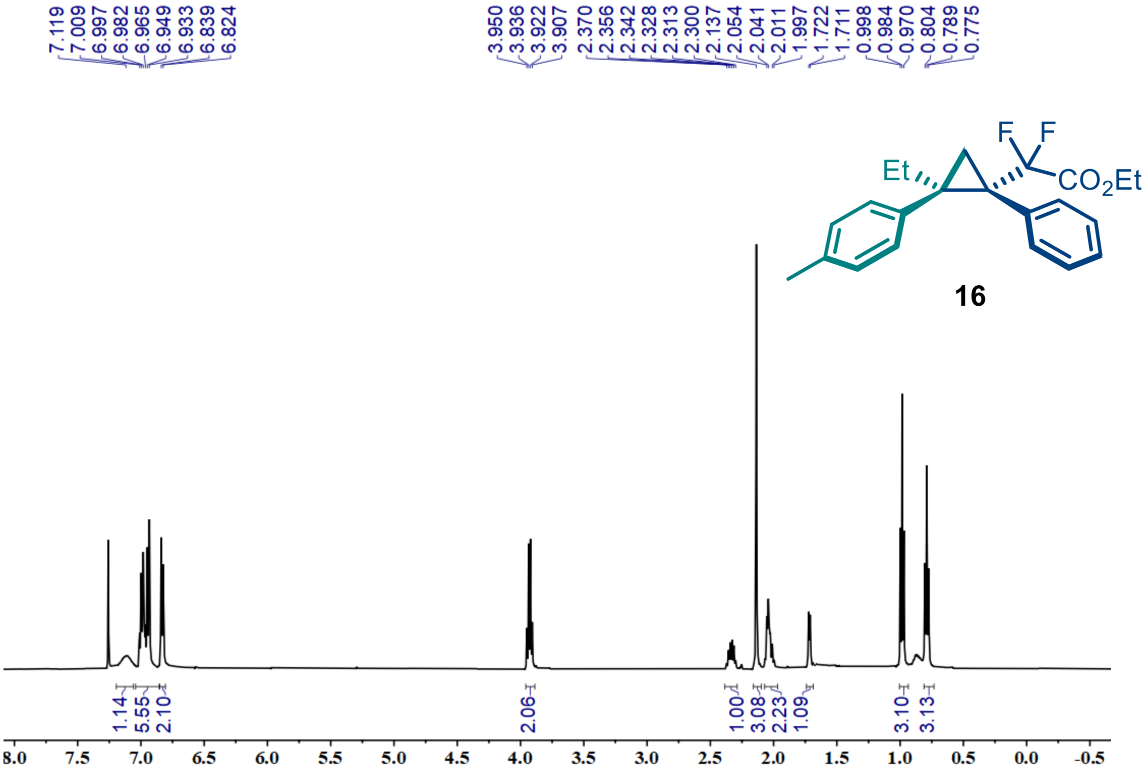

Figure S130, ^{1}H-NMR spectra copies of 16 related to scheme 2 and 3.

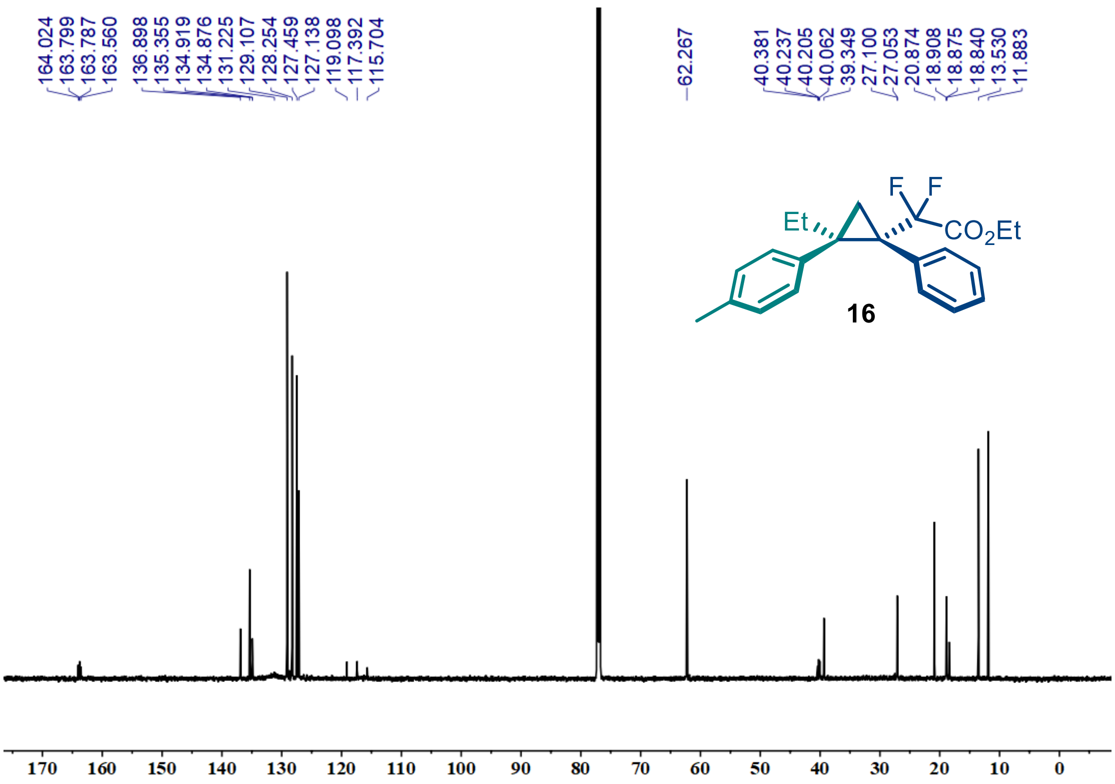

Figure S131, ^{13}C-NMR spectra copies of 16 related to scheme 2 and 3.

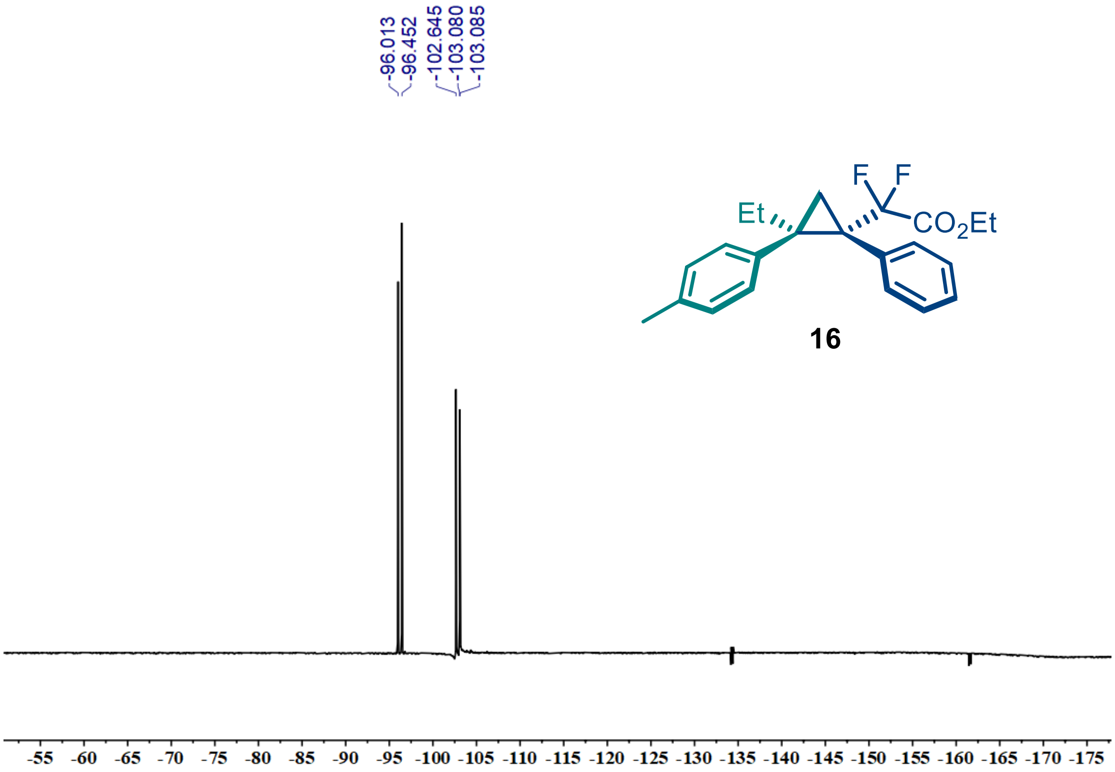

Figure S132, ^{19}F-NMR spectra copies of 16 related to scheme 2 and 3.

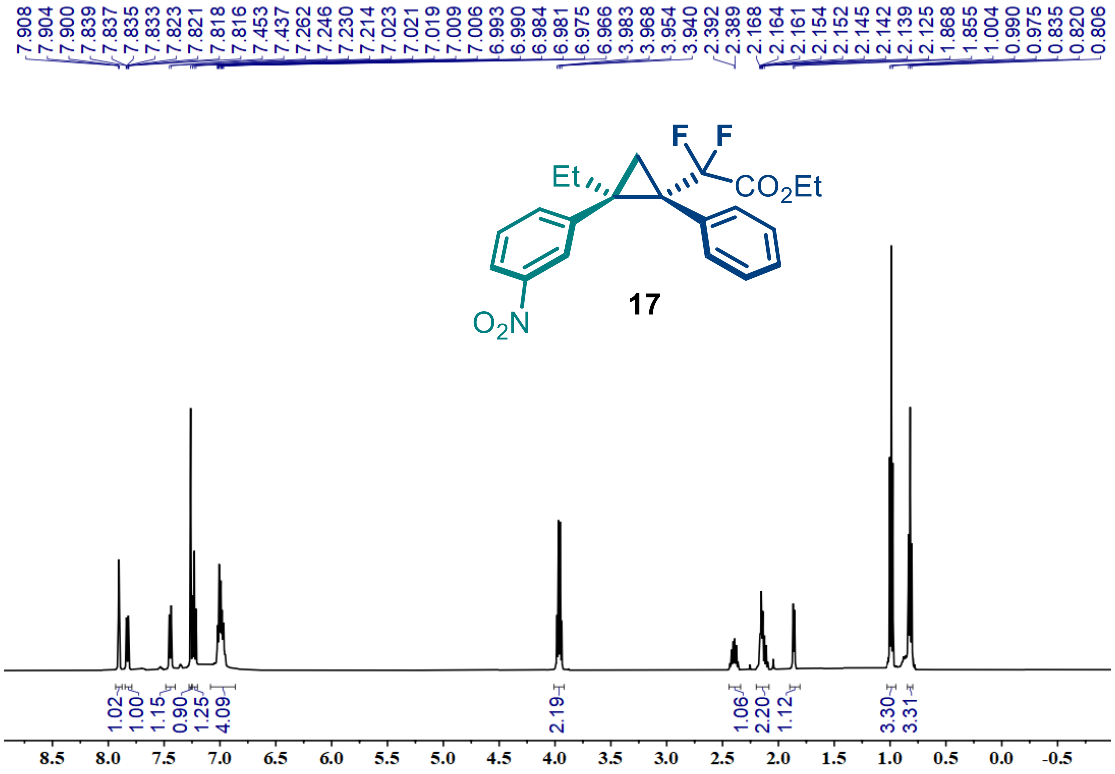

Figure S133, ^{1}H-NMR spectra copies of 17 related to scheme 2 and 3.

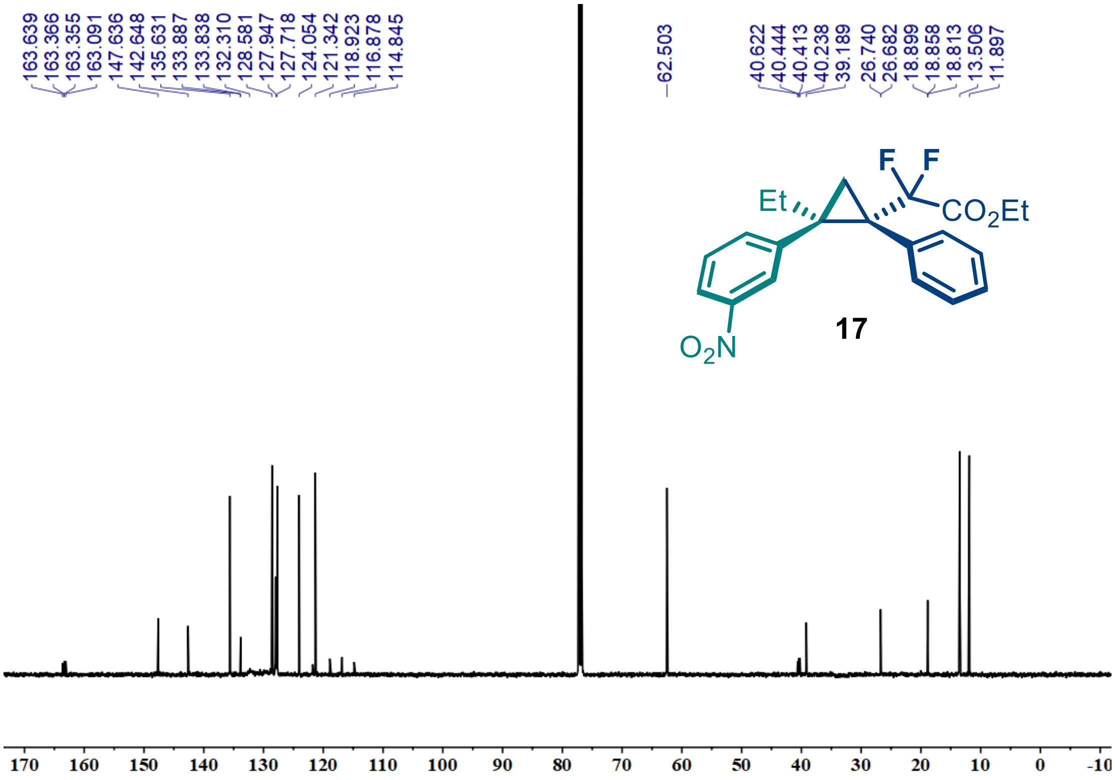

Figure S134, ^{13}C-NMR spectra copies of 17 related to scheme 2 and 3.

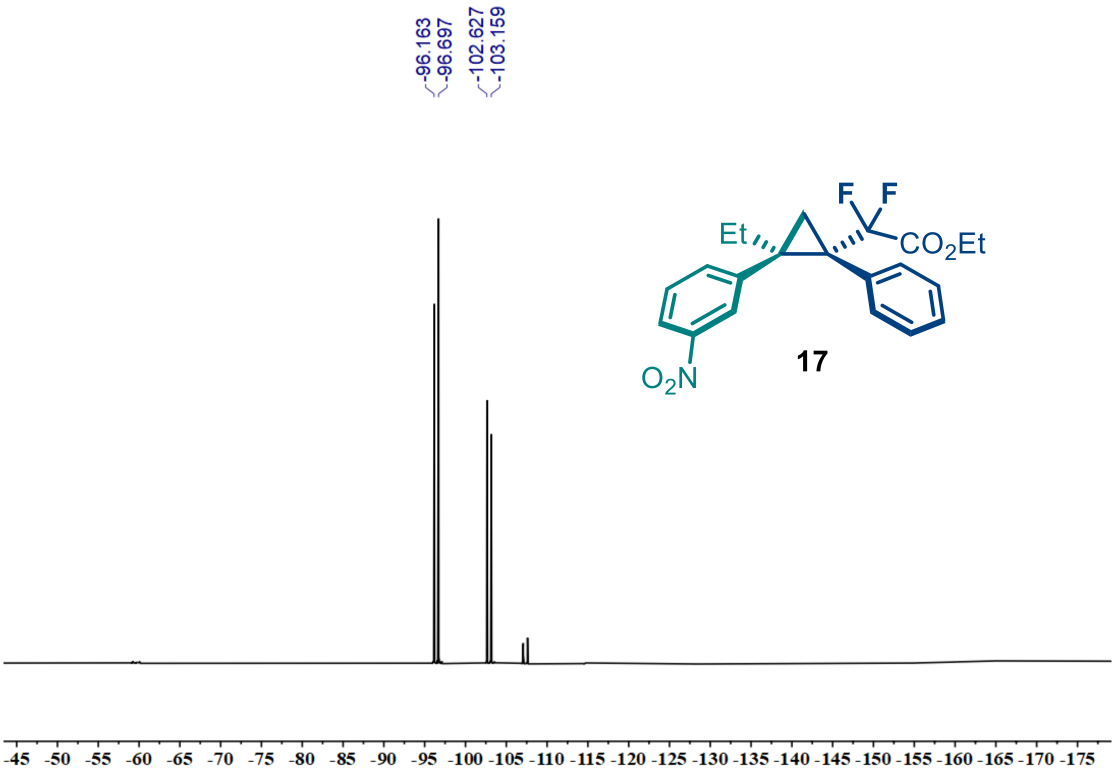

Figure S135, ^{19}F-NMR spectra copies of 17 related to scheme 2 and 3.

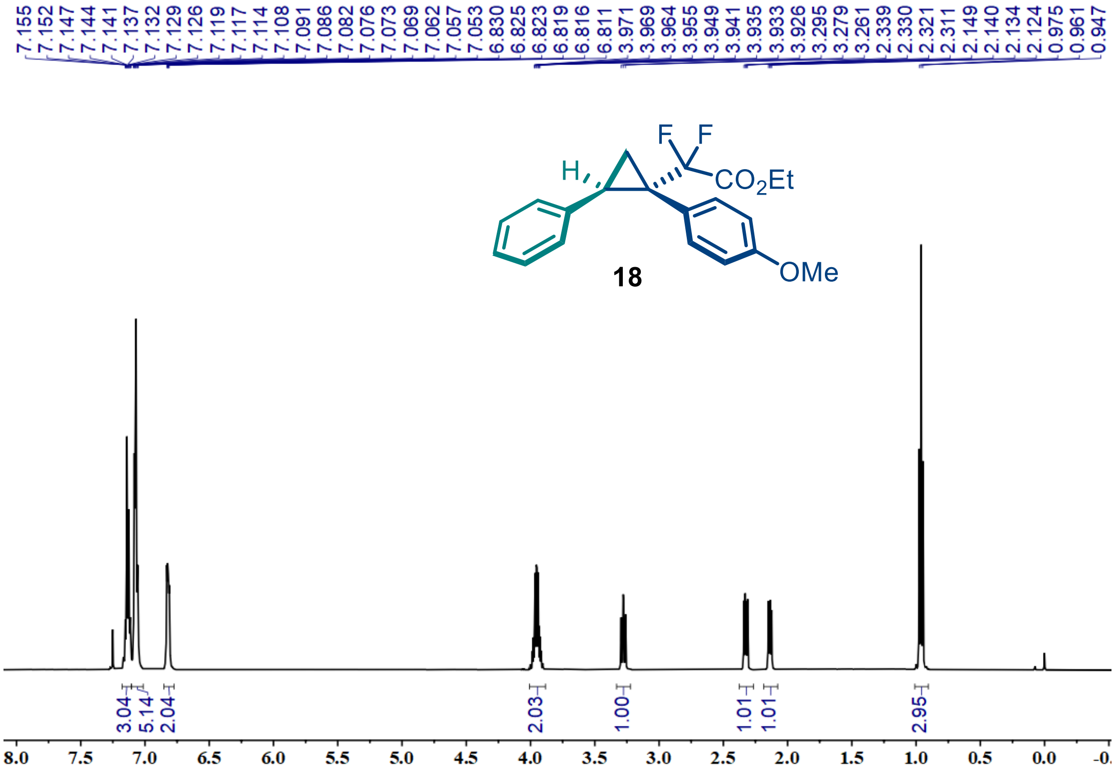

Figure S136, ^{1}H-NMR spectra copies of 18 related to scheme 2 and 3.

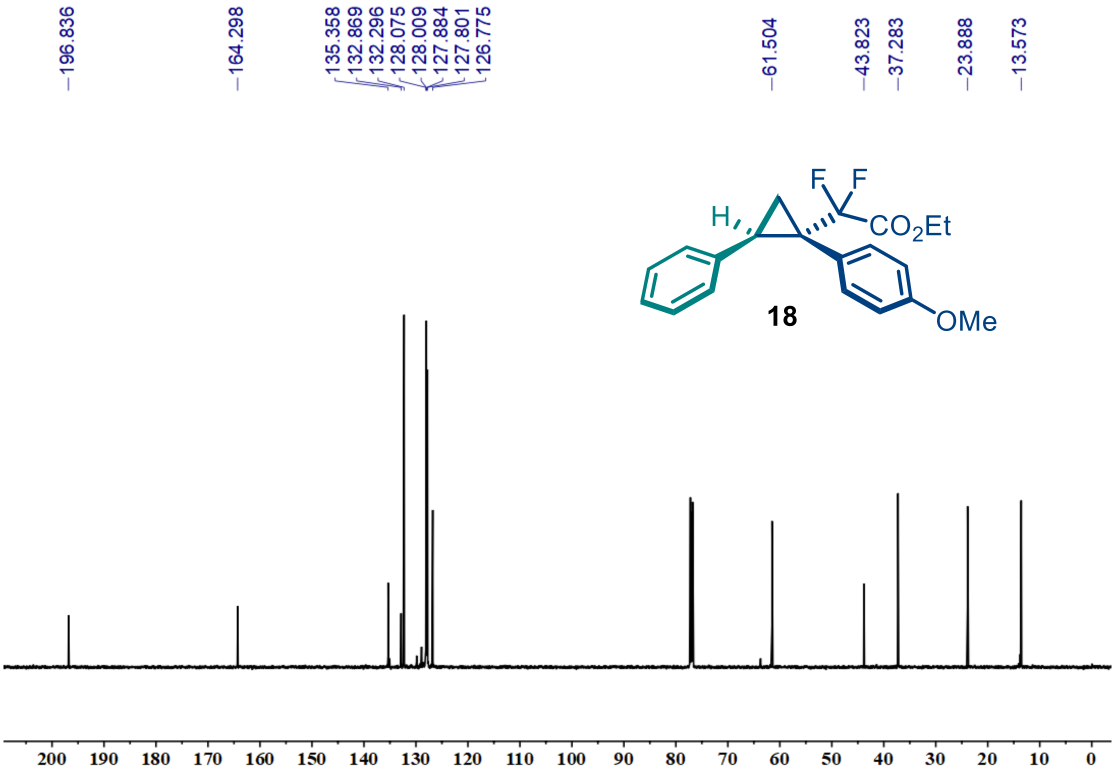

Figure S137, ^{13}C-NMR spectra copies of 18 related to scheme 2 and 3.

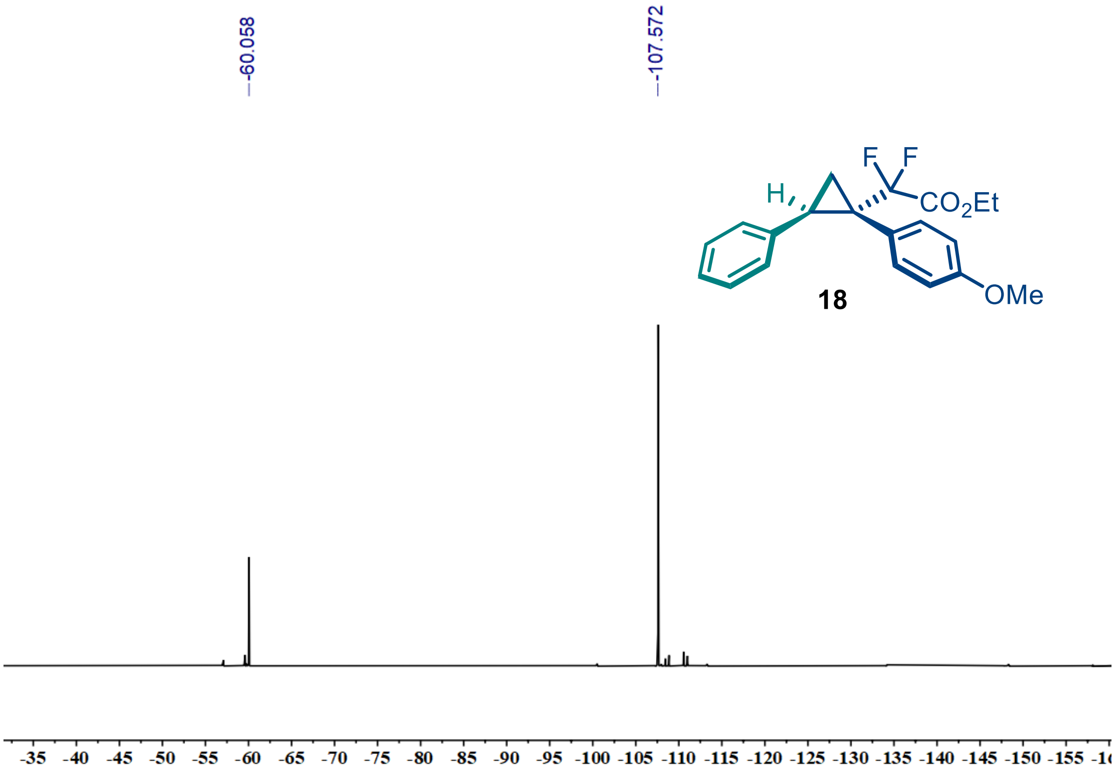

Figure S138, ^{19}F-NMR spectra copies of 18 related to scheme 2 and 3.

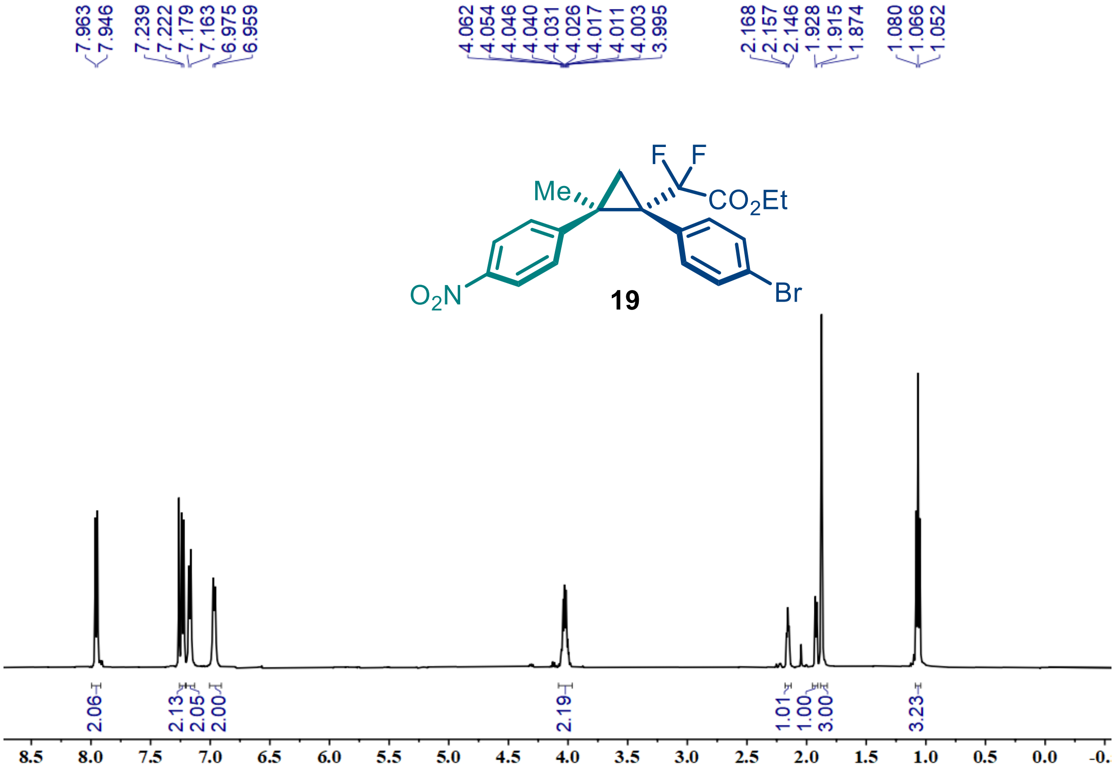

Figure S139, ^{1}H-NMR spectra copies of 19 related to scheme 2 and 3.

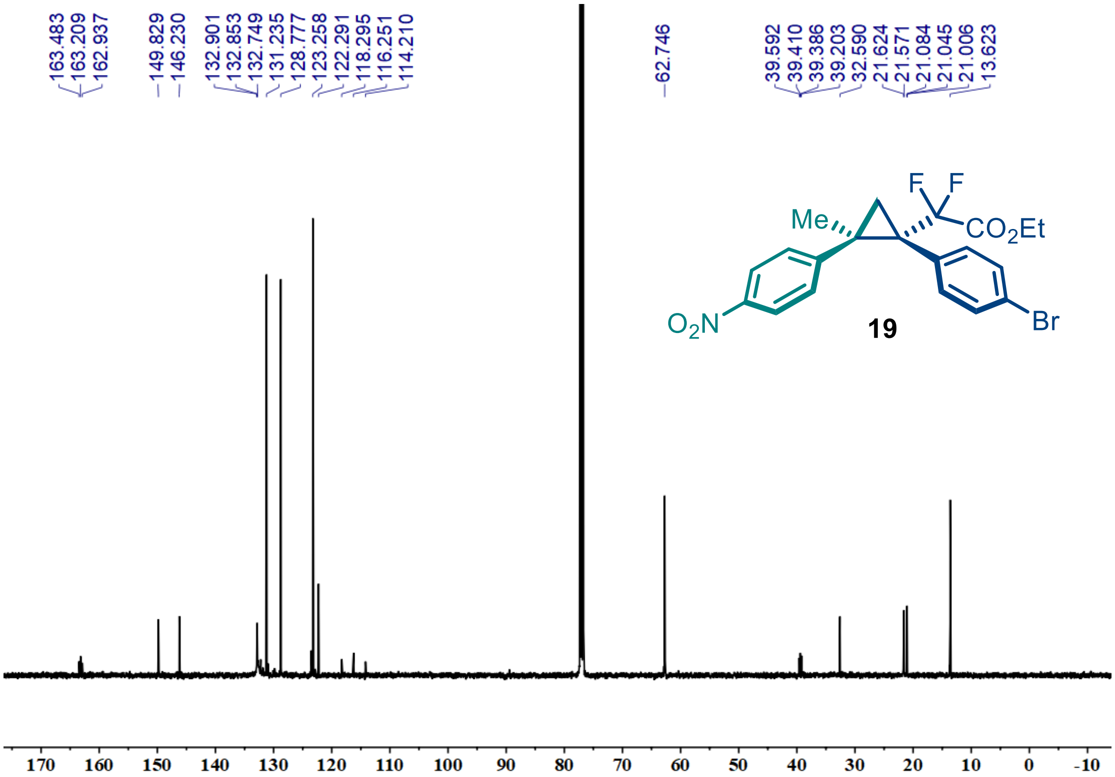

Figure S140, ^{13}C-NMR spectra copies of 19 related to scheme 2 and 3.

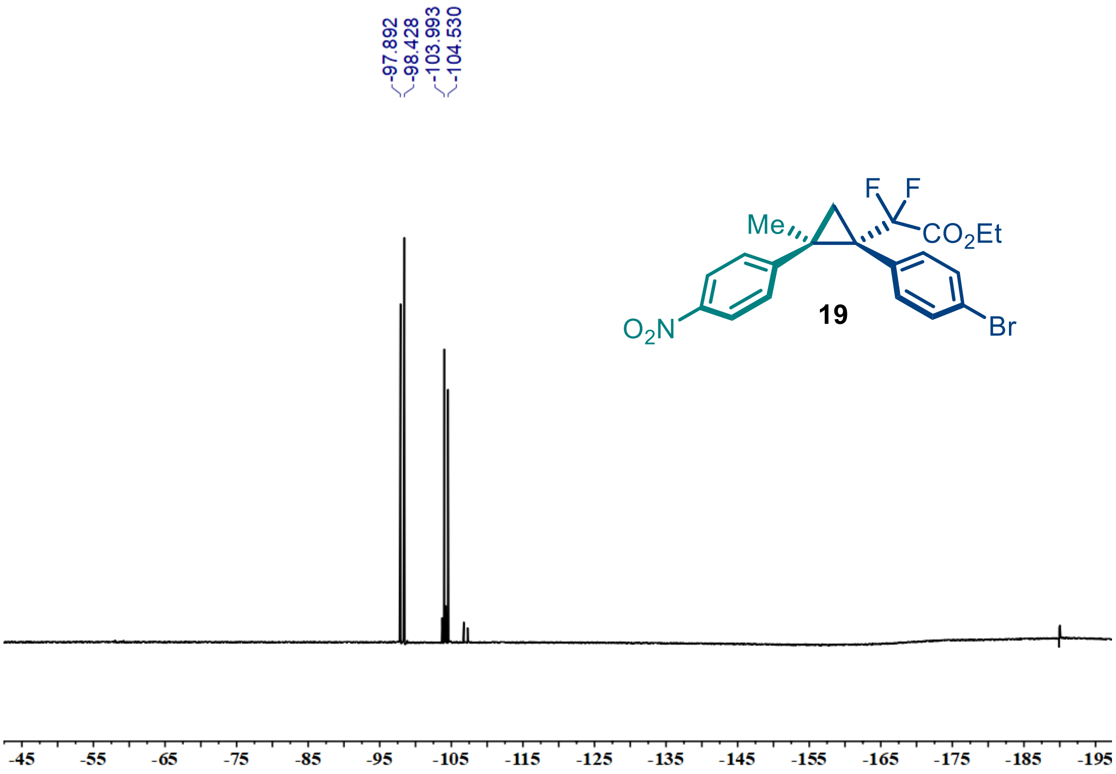

Figure S141, ^{19}F-NMR spectra copies of 19 related to scheme 2 and 3.

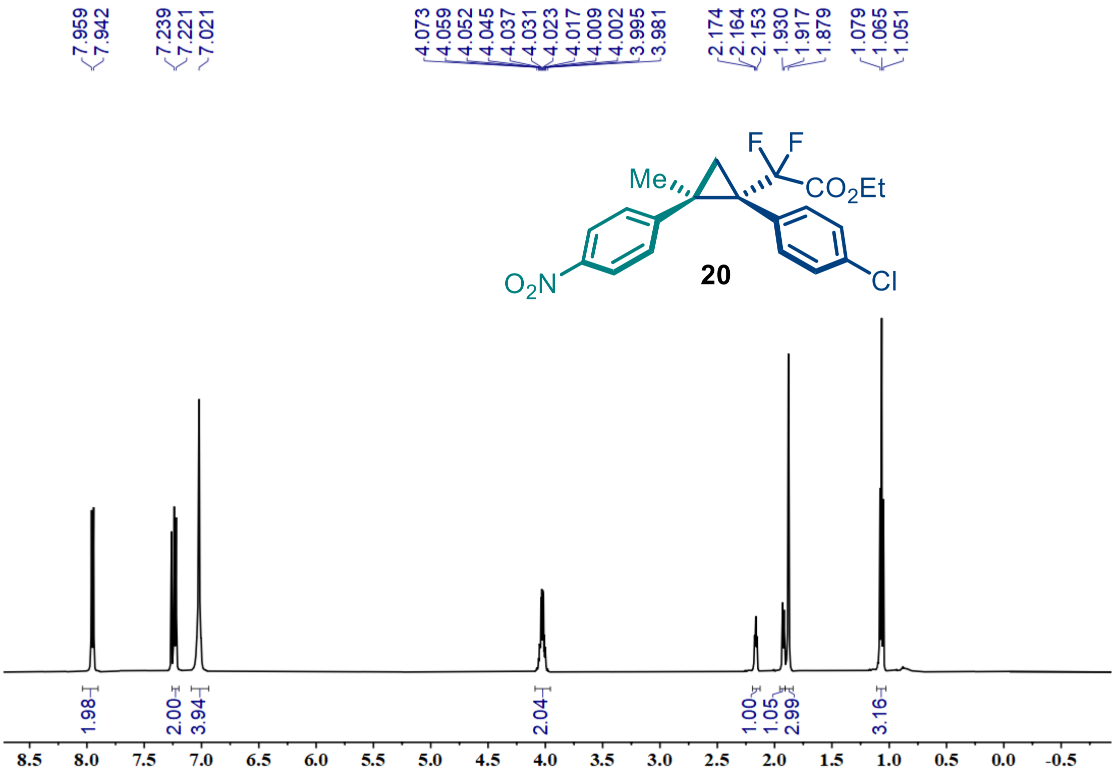

Figure S142, ^{1}H-NMR spectra copies of 20related to scheme 2 and 3.

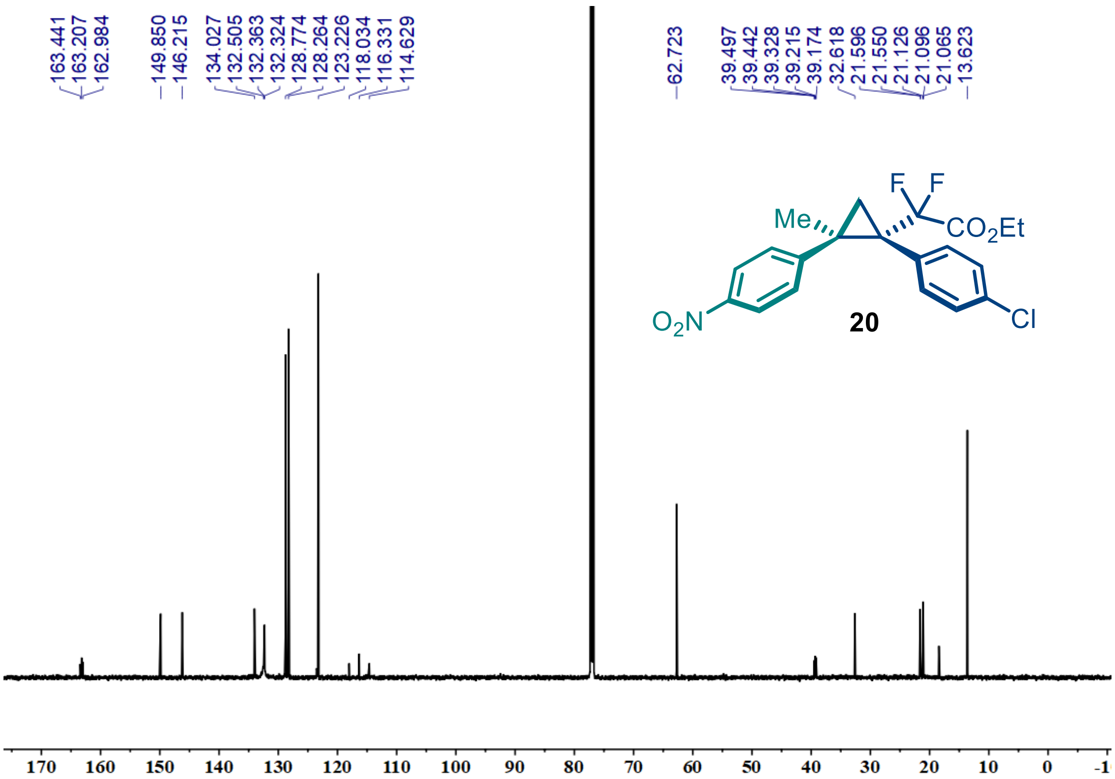

Figure S143, ^{13}C-NMR spectra copies of 20 related to scheme 2 and 3.

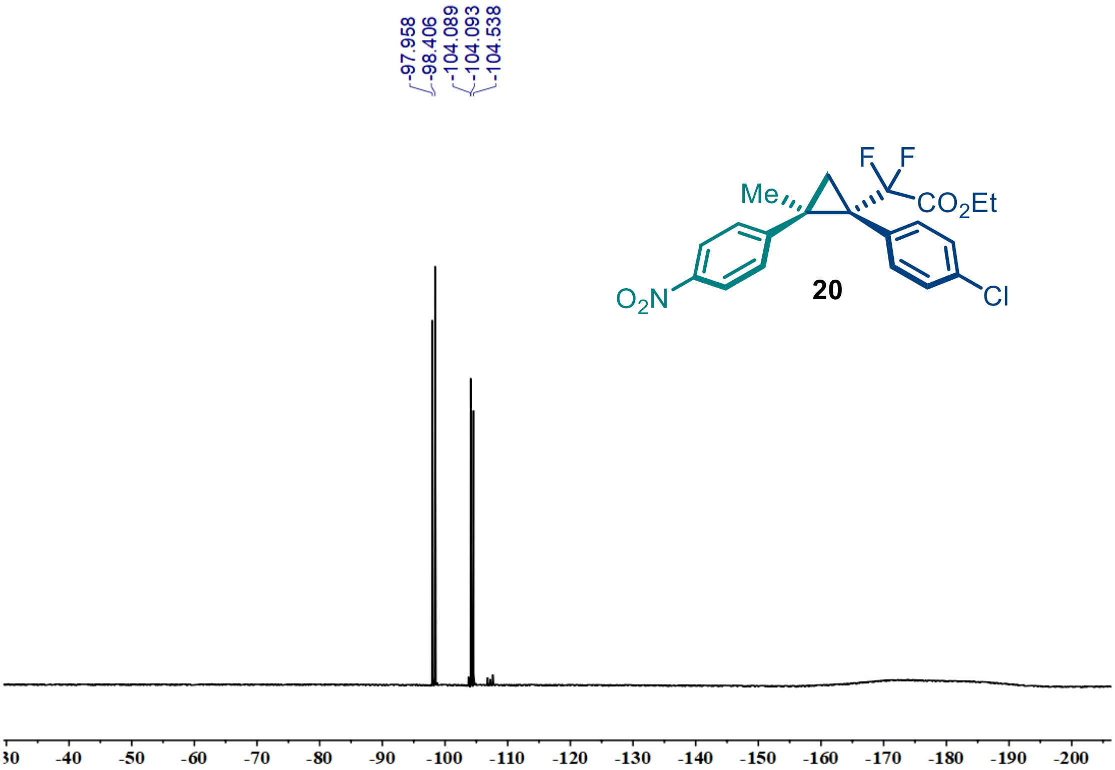

Figure S144, ^{19}F-NMR spectra copies of 20 related to scheme 2 and 3.

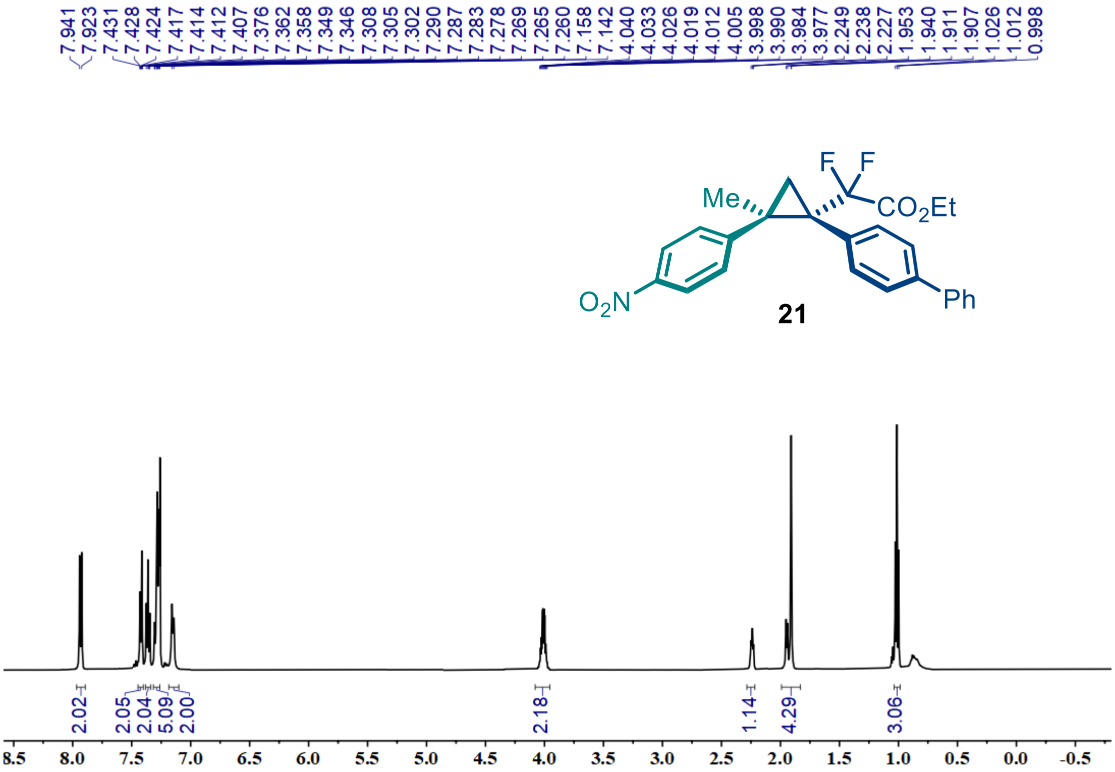

Figure S145, ^{1}H-NMR spectra copies of 21 related to scheme 2 and 3.

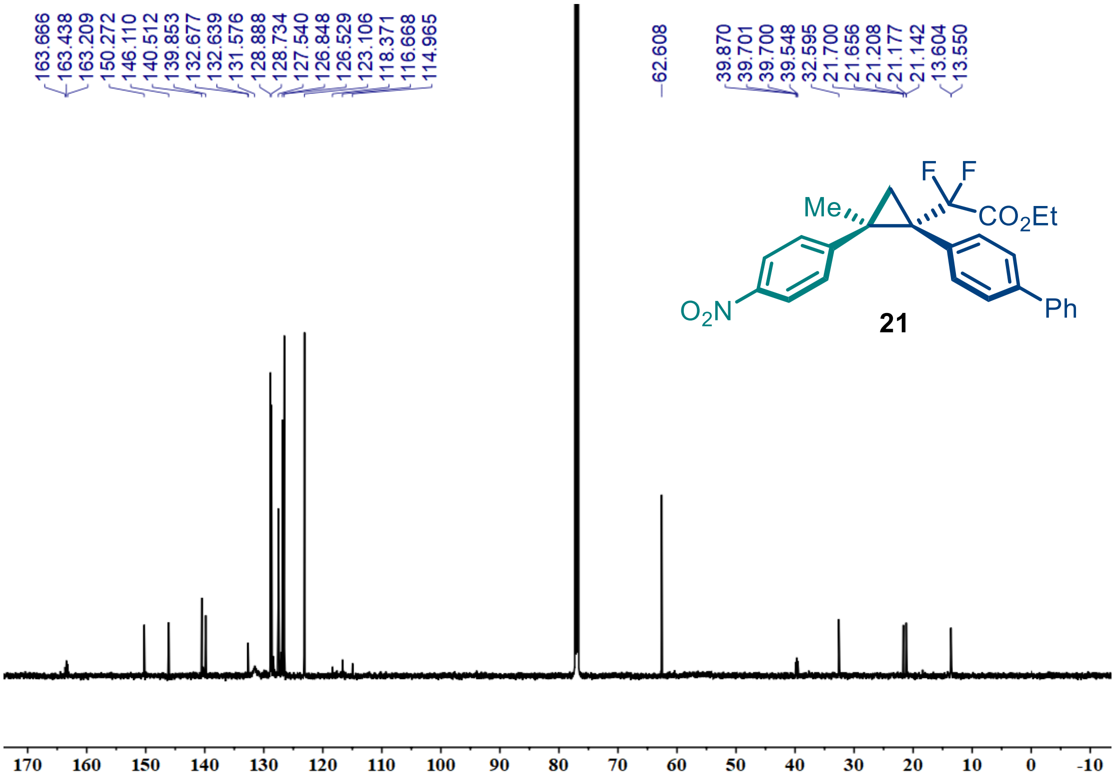

Figure S146, ^{13}C-NMR spectra copies of 21 related to scheme 2 and 3.

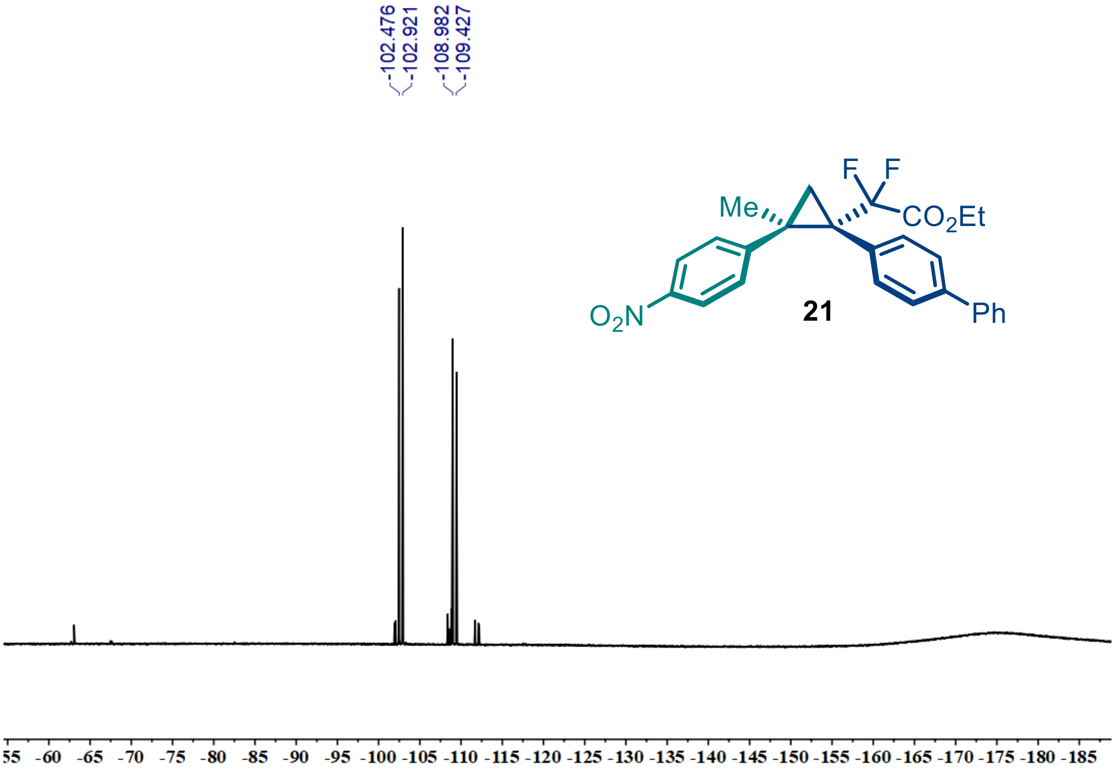

Figure S147, ^{19}F-NMR spectra copies of 21 related to scheme 2 and 3.

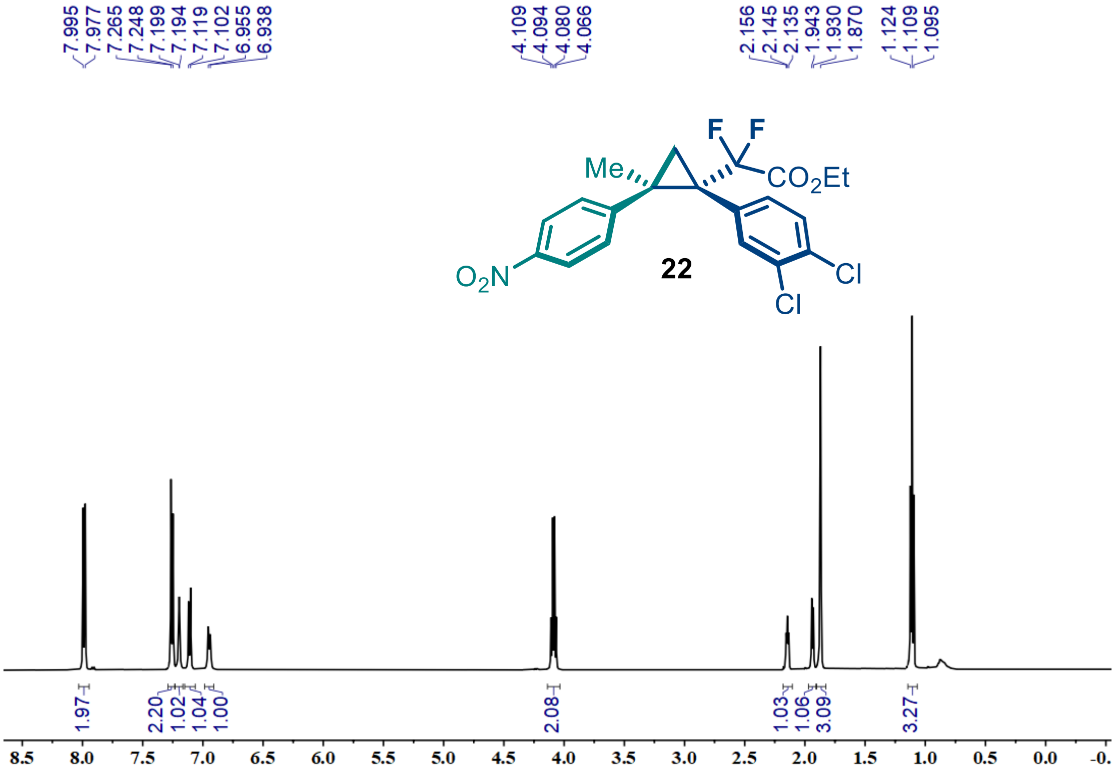

Figure S148, ^{1}H-NMR spectra copies of 22 related to scheme 2 and 3.

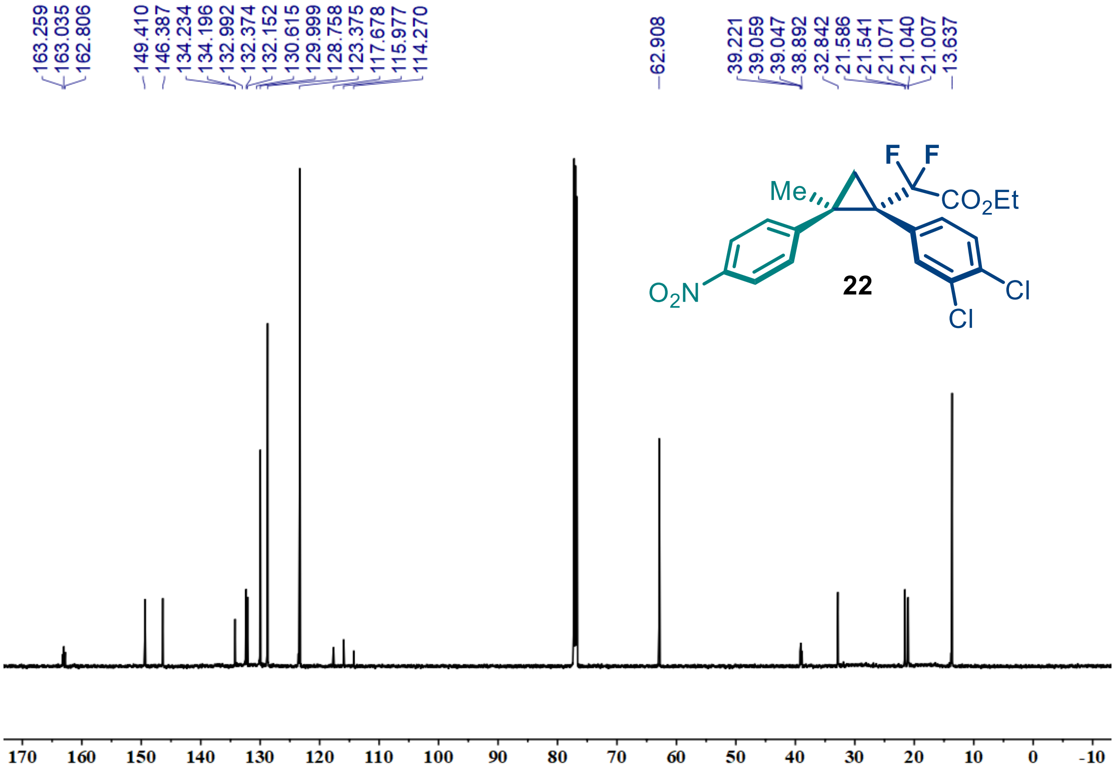

Figure S149, ^{13}C-NMR spectra copies of 22 related to scheme 2 and 3.

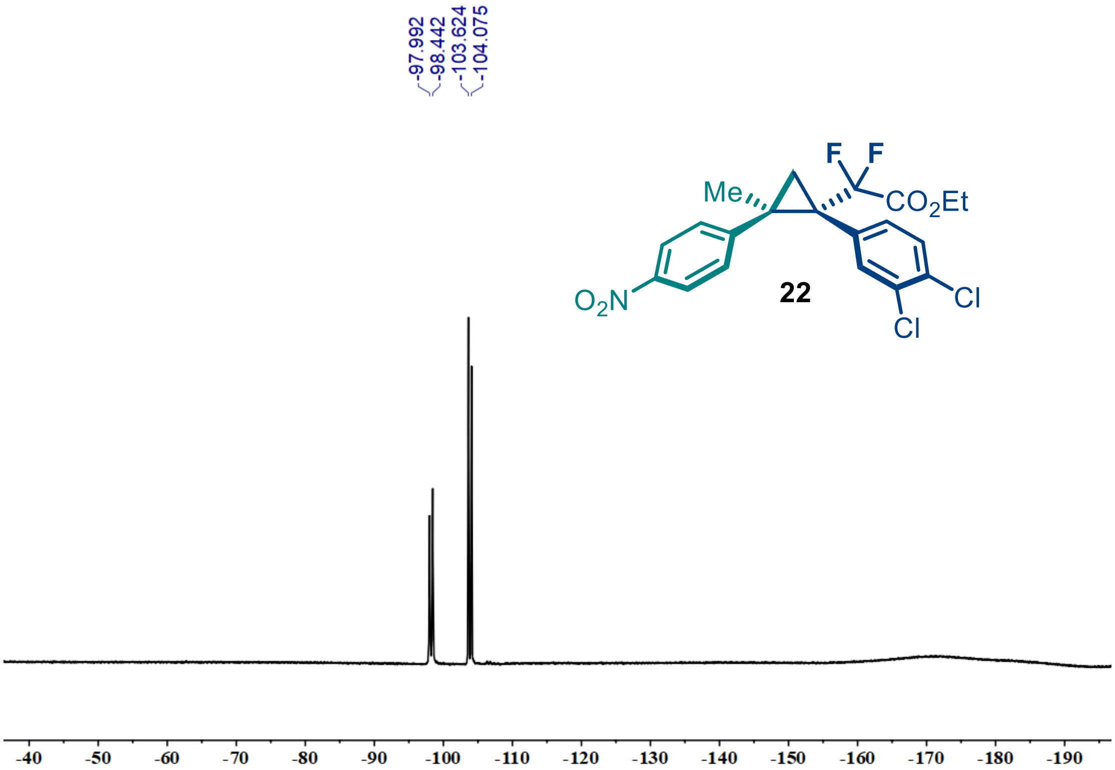

Figure S150, ^{19}F-NMR spectra copies of 22 related to scheme 2 and 3.

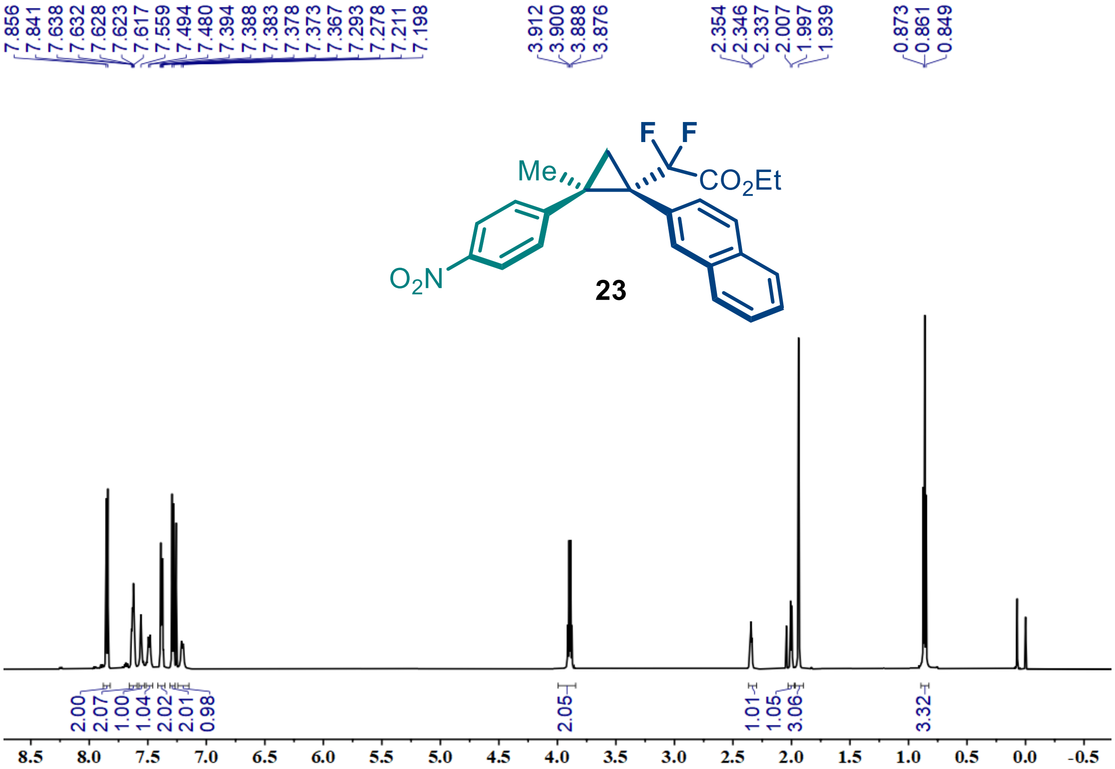

Figure S151, ^{1}H-NMR spectra copies of 23 related to scheme 2 and 3.

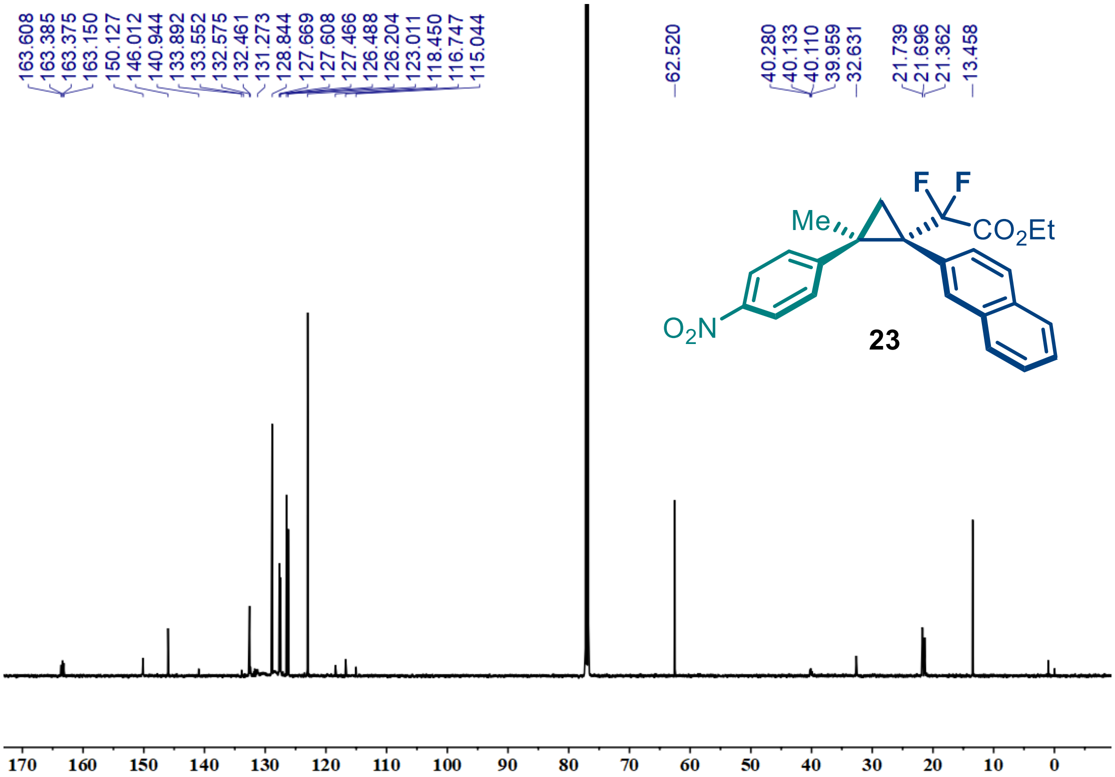

Figure S152, ^{13}C-NMR spectra copies of 23 related to scheme 2 and 3.

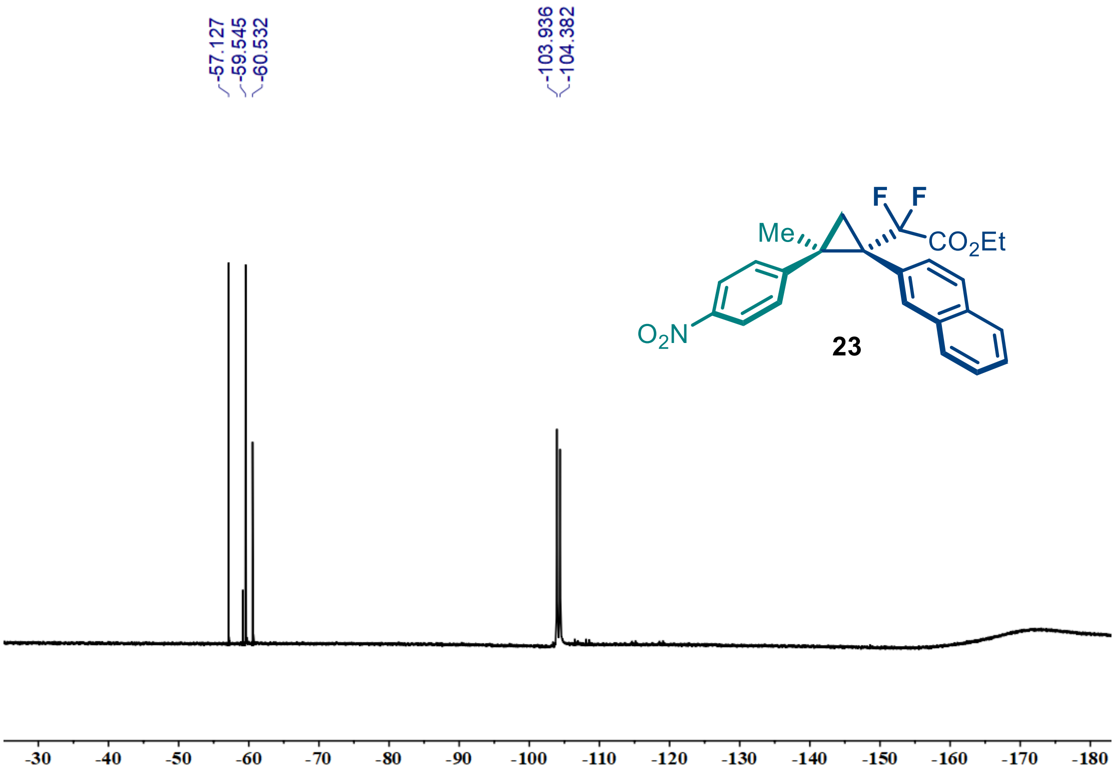

Figure S153, ^{19}F-NMR spectra copies of 23 related to scheme 2 and 3.

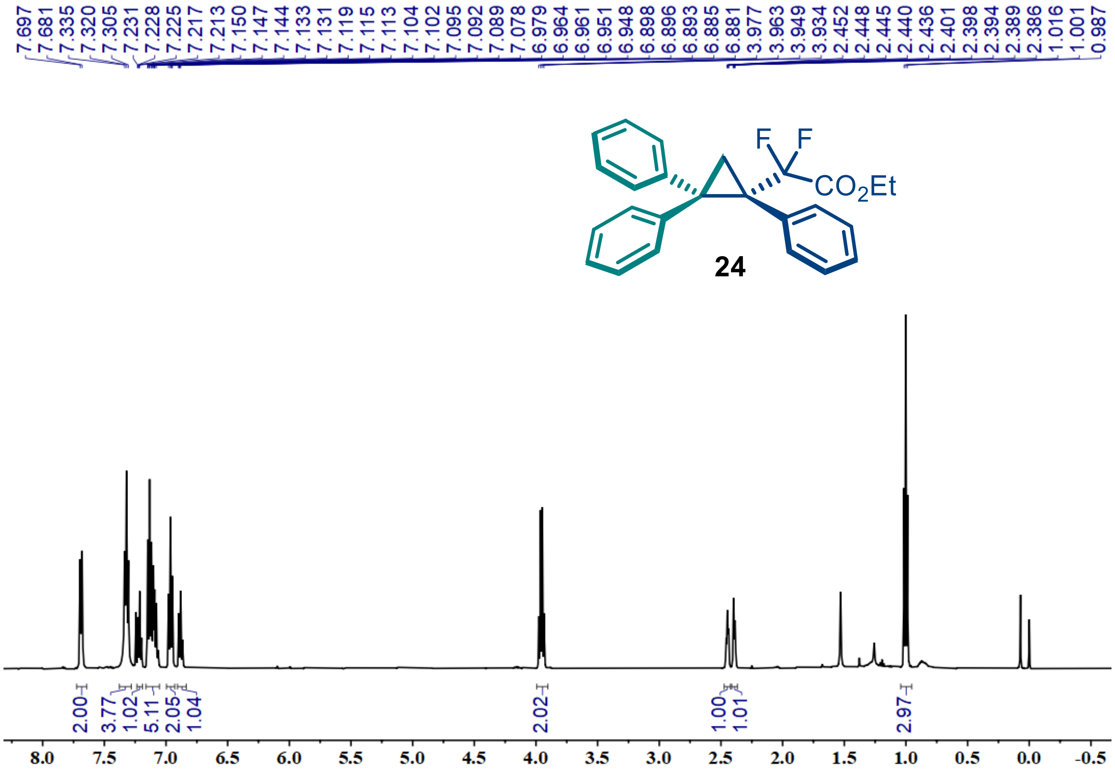

Figure S154, ^{1}H-NMR spectra copies of 24 related to scheme 2 and 3.

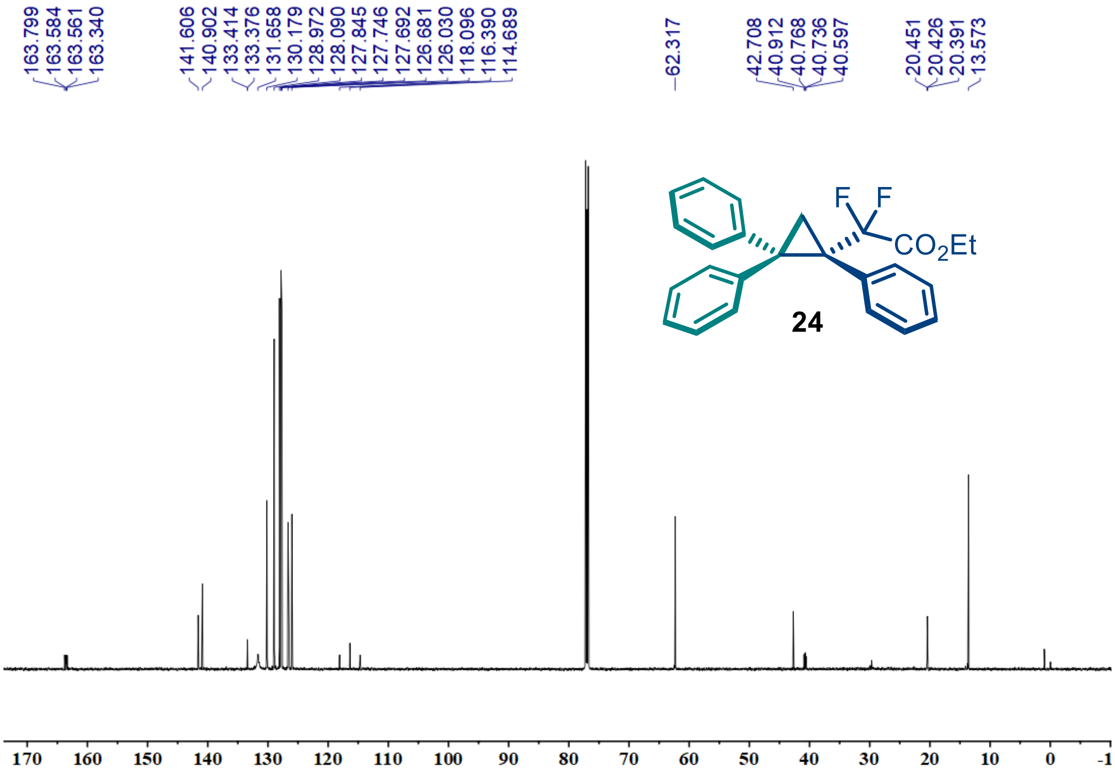

Figure S155, ^{13}C-NMR spectra copies of 24 related to scheme 2 and 3.

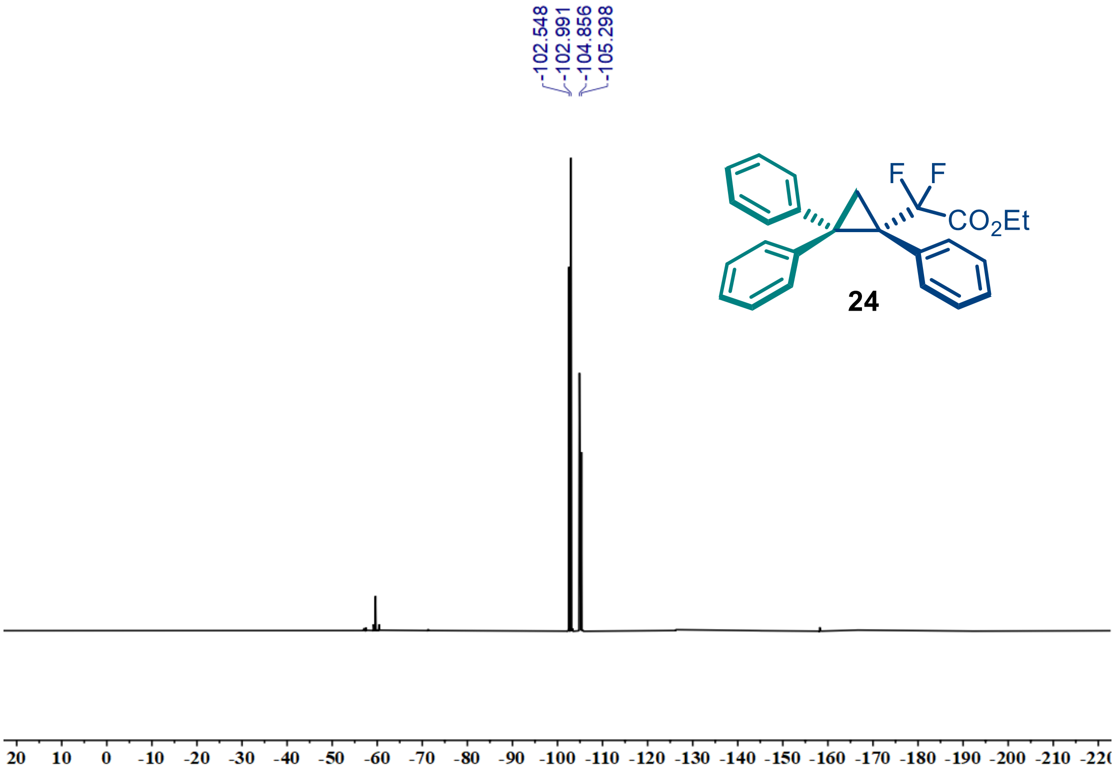

Figure S156, ^{19}F-NMR spectra copies of 24 related to scheme 2 and 3.

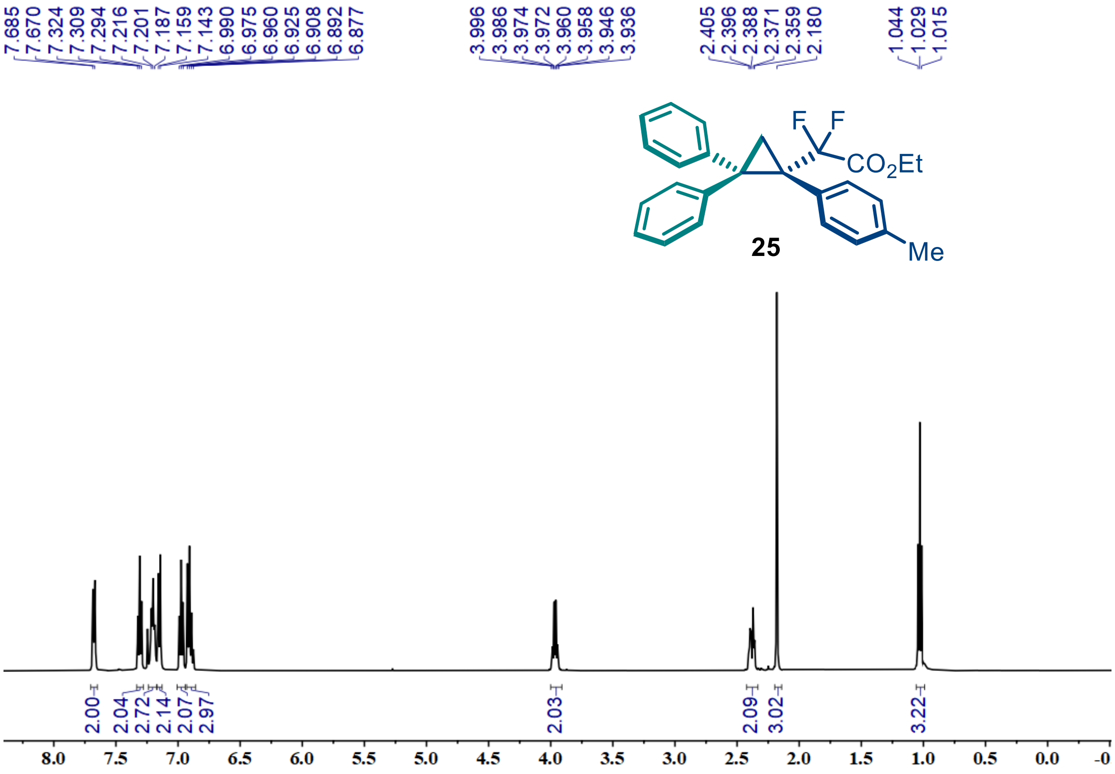

Figure S157, ^{1}H-NMR spectra copies of 25 related to scheme 2 and 3.

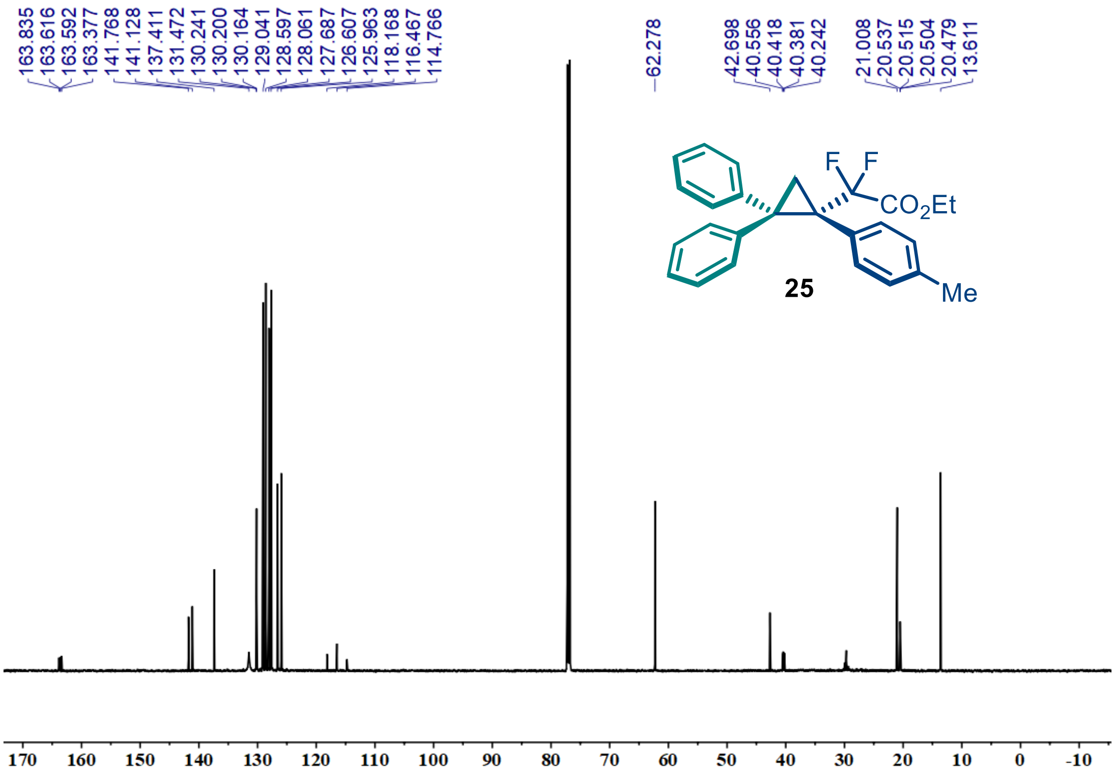

Figure S158, ^{13}C-NMR spectra copies of 25 related to scheme 2 and 3.

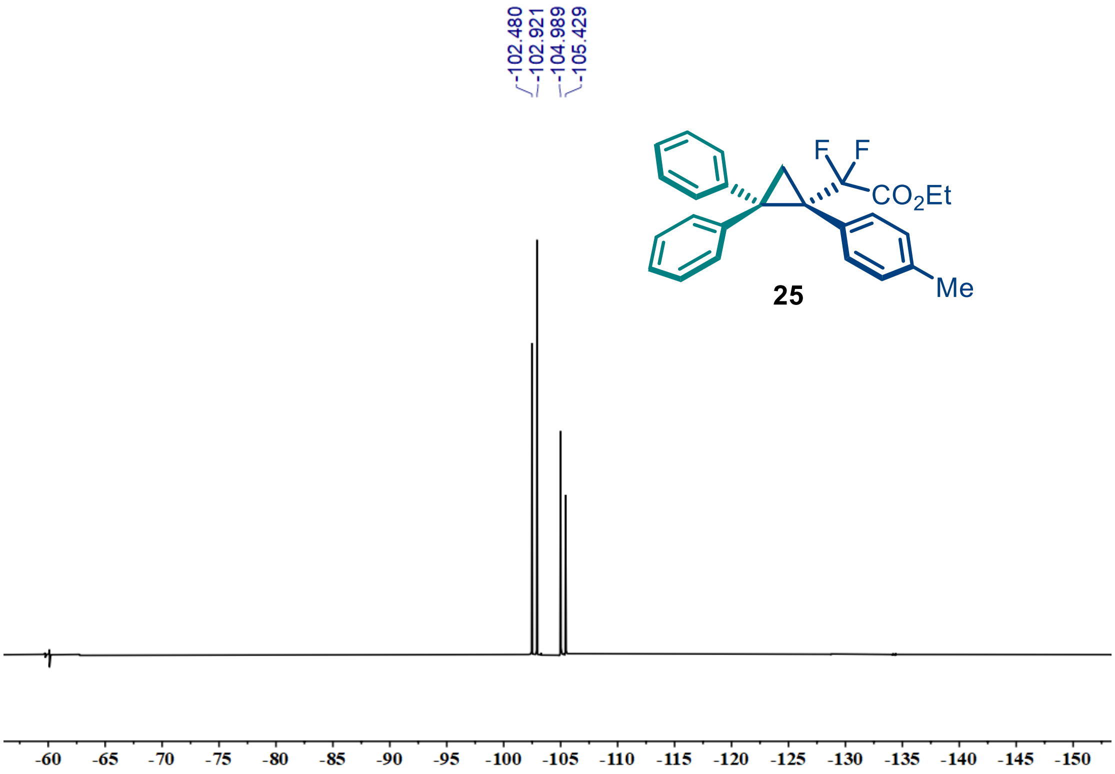

Figure S159, ^{19}F-NMR spectra copies of 25 related to scheme 2 and 3.

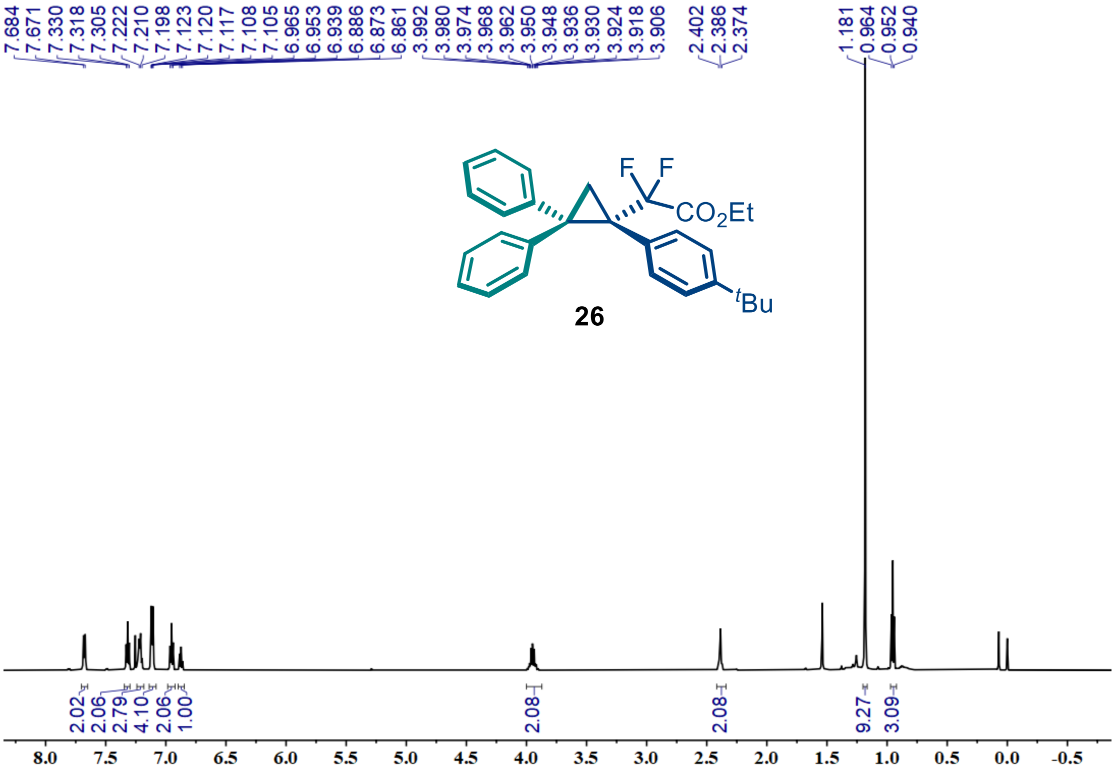

Figure S160, ^{1}H-NMR spectra copies of 26 related to scheme 2 and 3.

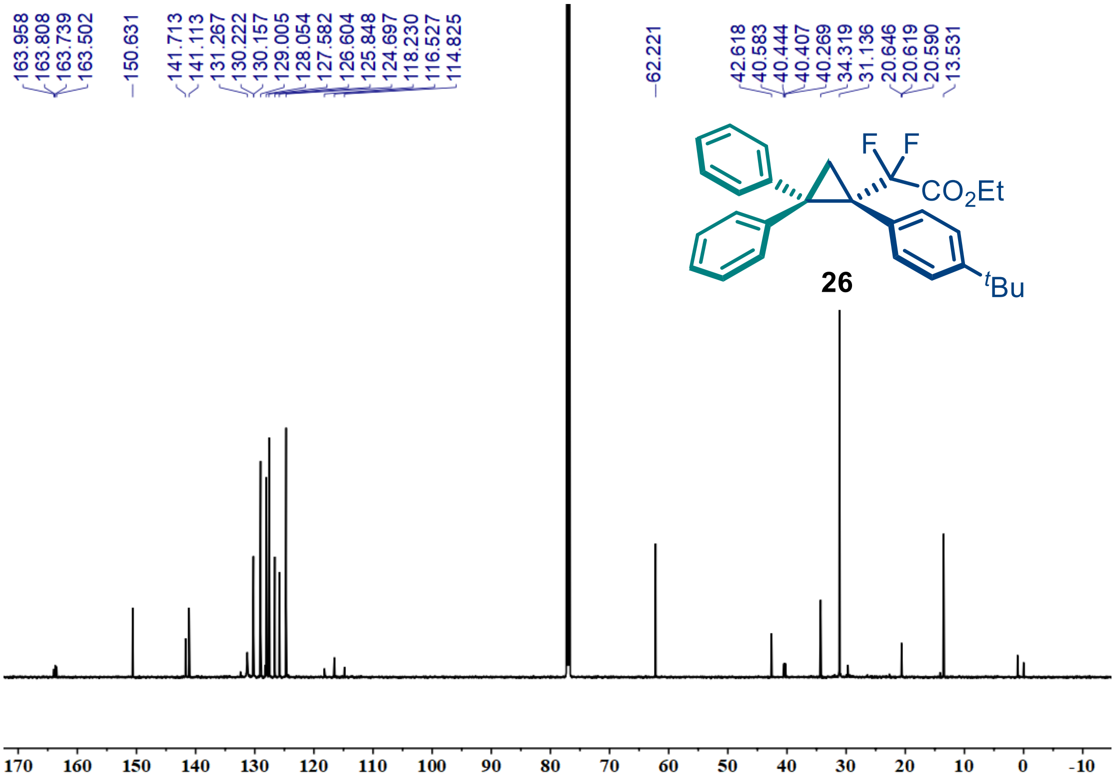

Figure S161, ^{13}C-NMR spectra copies of 26 related to scheme 2 and 3.

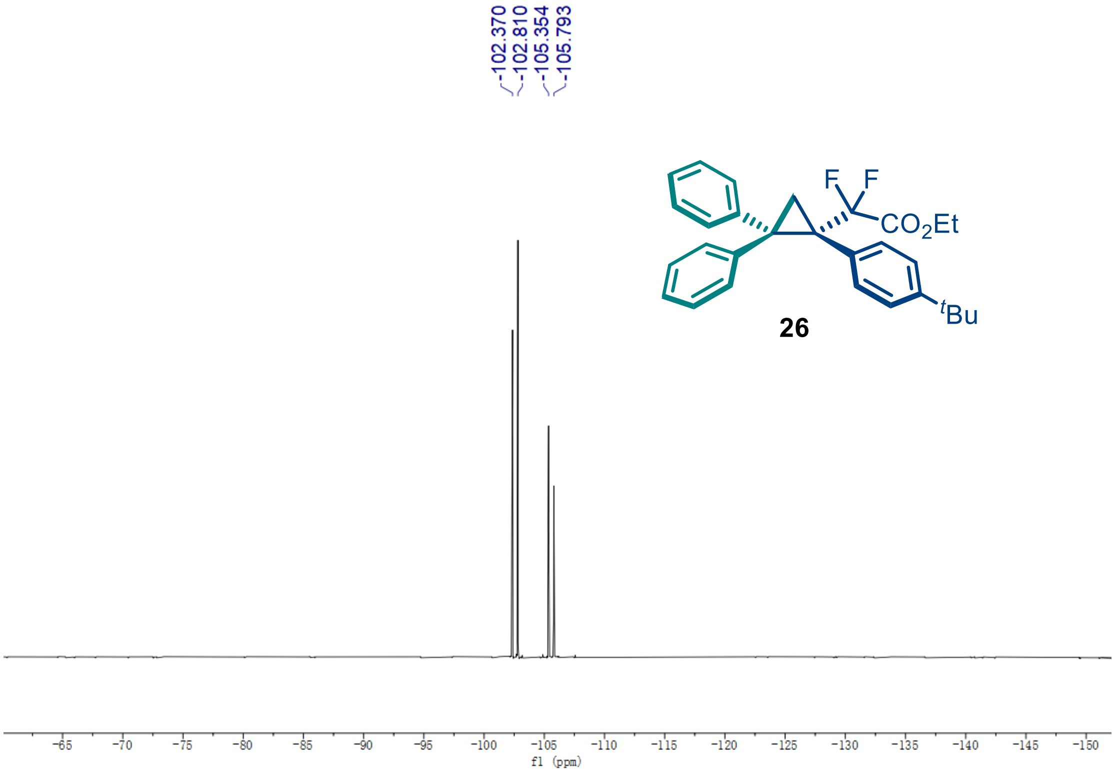

Figure S162, ^{19}F-NMR spectra copies of 26 related to scheme 2 and 3.

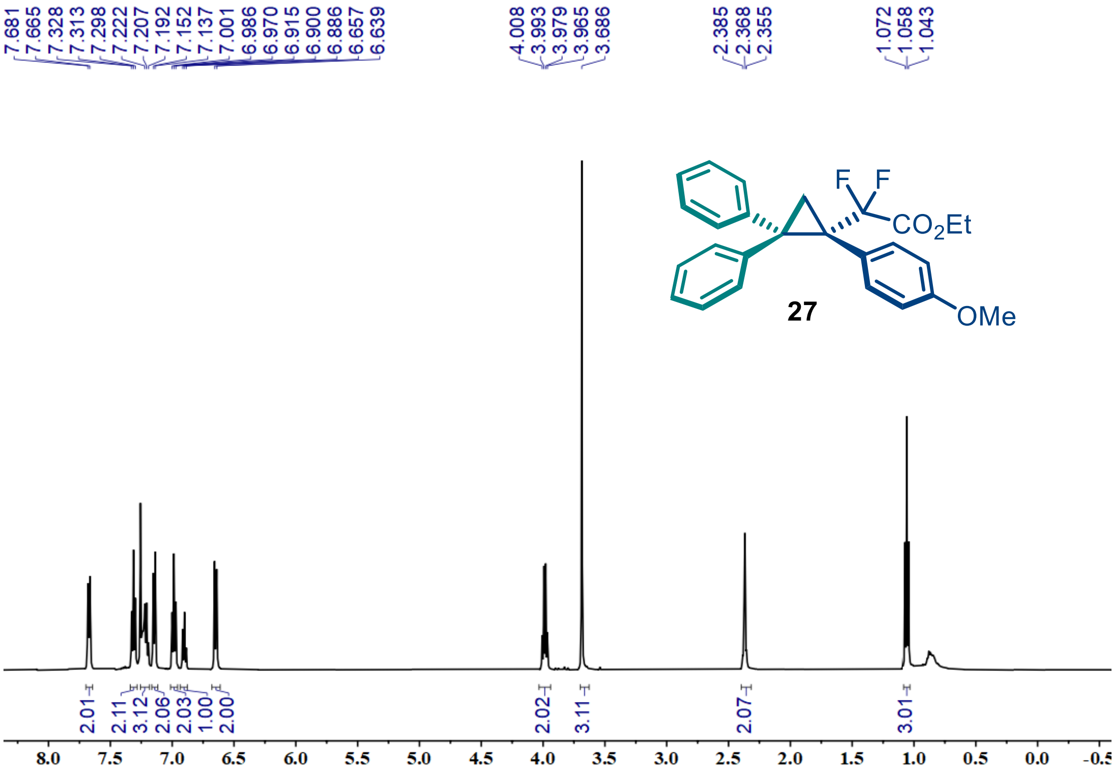

Figure S163, ¹H-NMR spectra copies of 27 related to scheme 2 and 3.

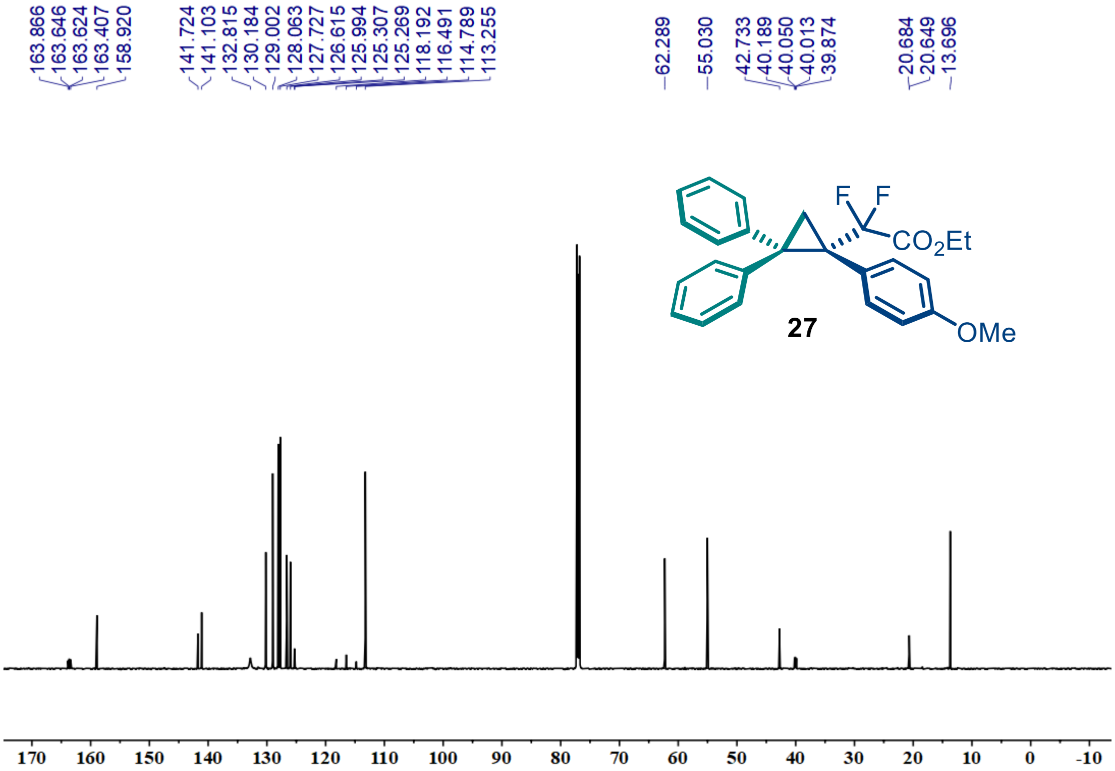

Figure S164, ¹³C-NMR spectra copies of 27 related to scheme 2 and 3.

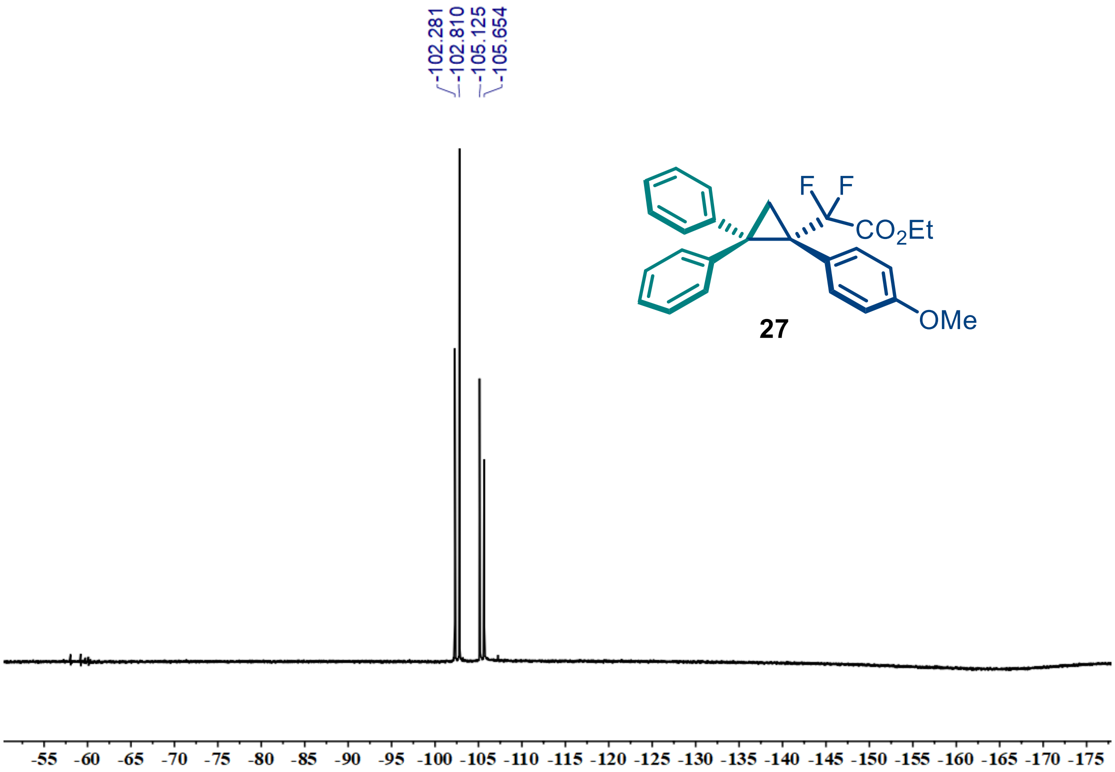

Figure S165, ^{19}F-NMR spectra copies of 27 related to scheme 2 and 3.

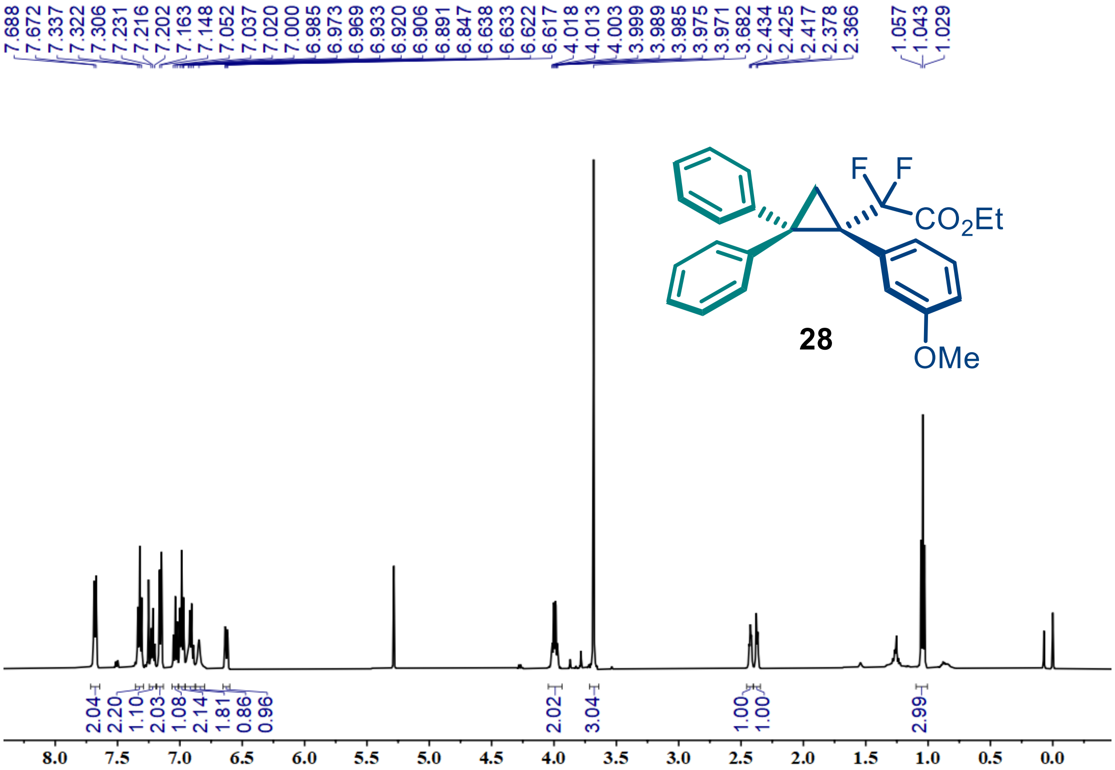

Figure S166, ^{1}H-NMR spectra copies of 28 related to scheme 2 and 3.

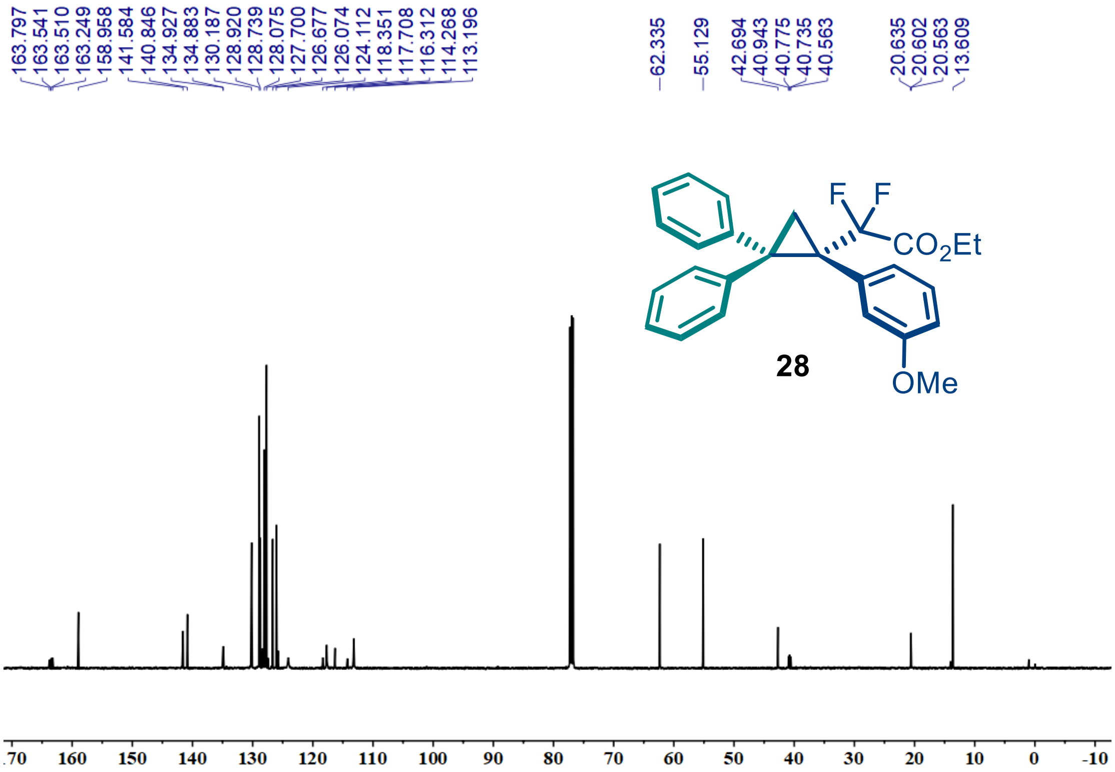

Figure S167, ^{13}C-NMR spectra copies of 28 related to scheme 2 and 3.

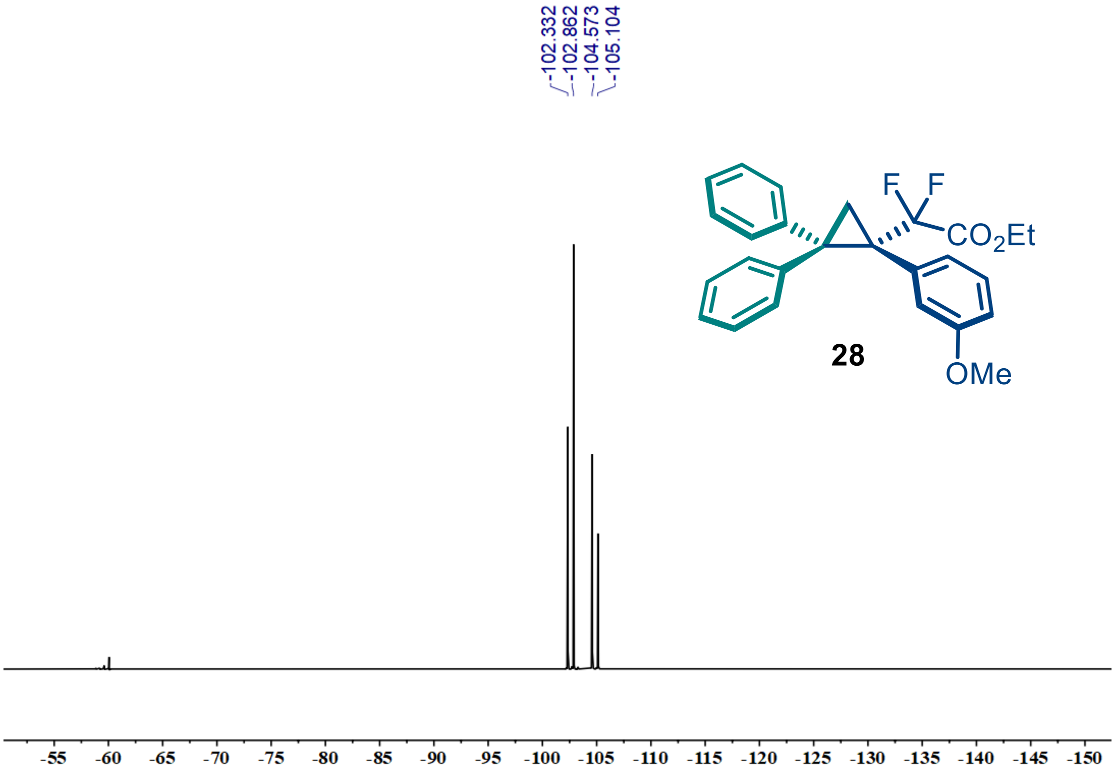

Figure S168, ^{19}F-NMR spectra copies of 28 related to scheme 2 and 3.

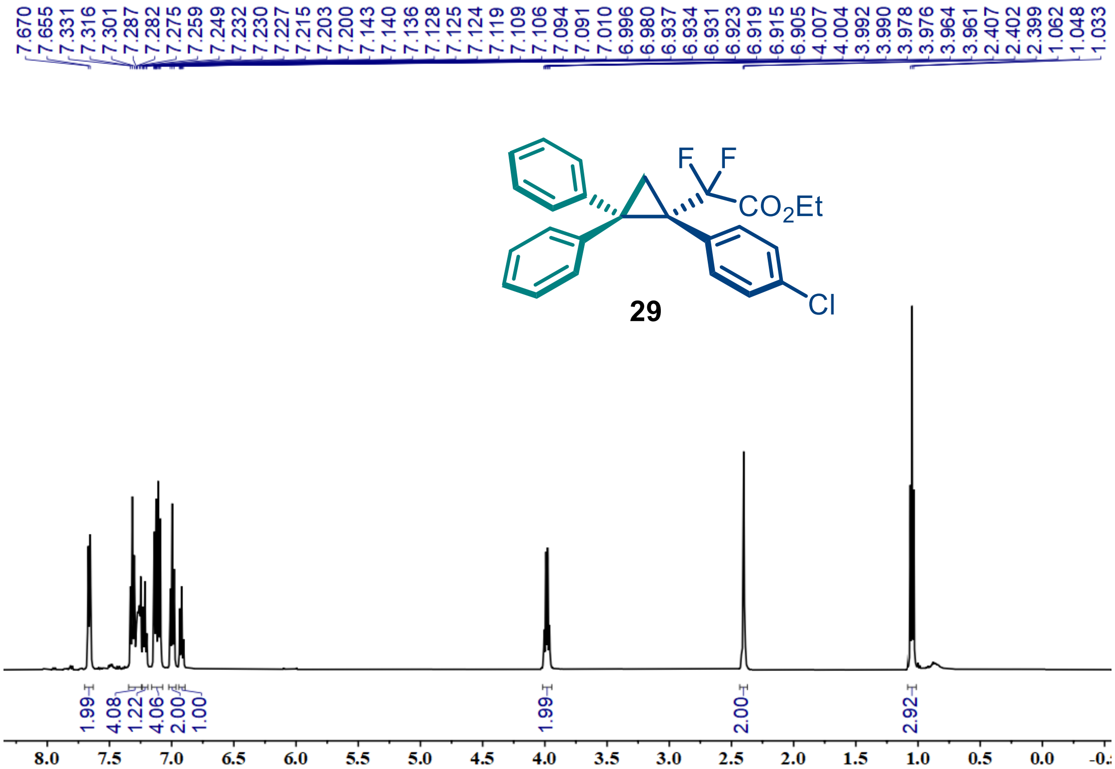

Figure S169, ^{1}H-NMR spectra copies of 29 related to scheme 2 and 3.

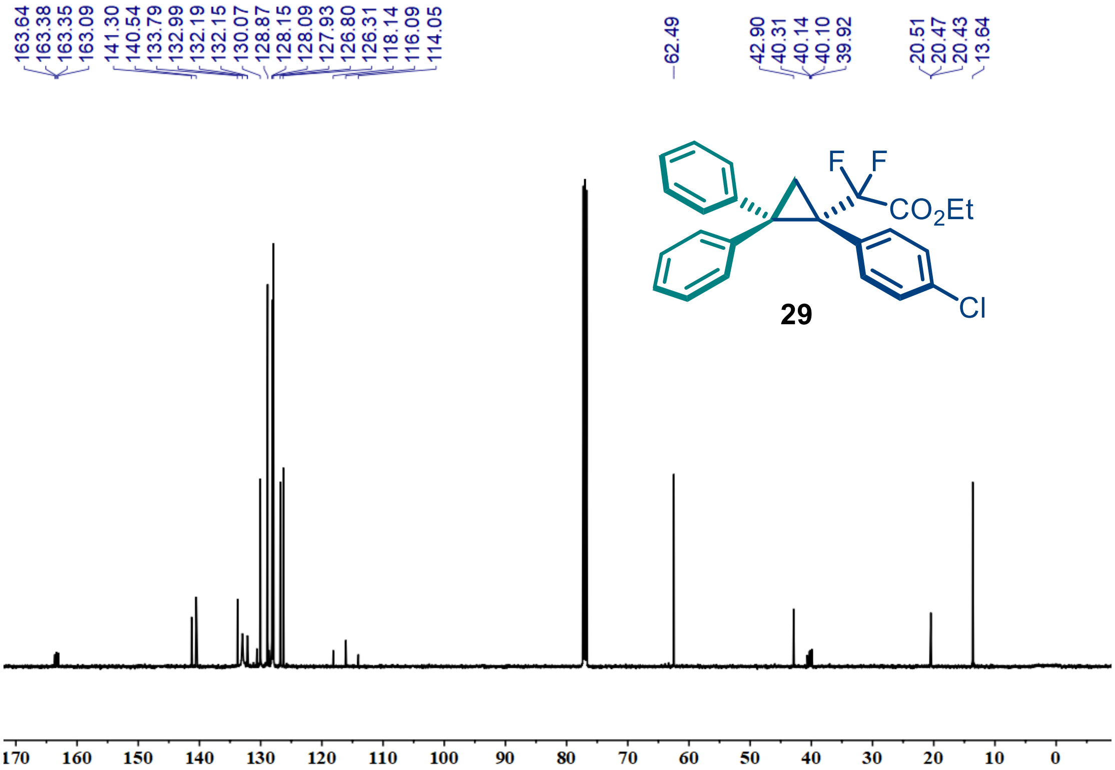

Figure S170, ^{13}C-NMR spectra copies of 29 related to scheme 2 and 3.

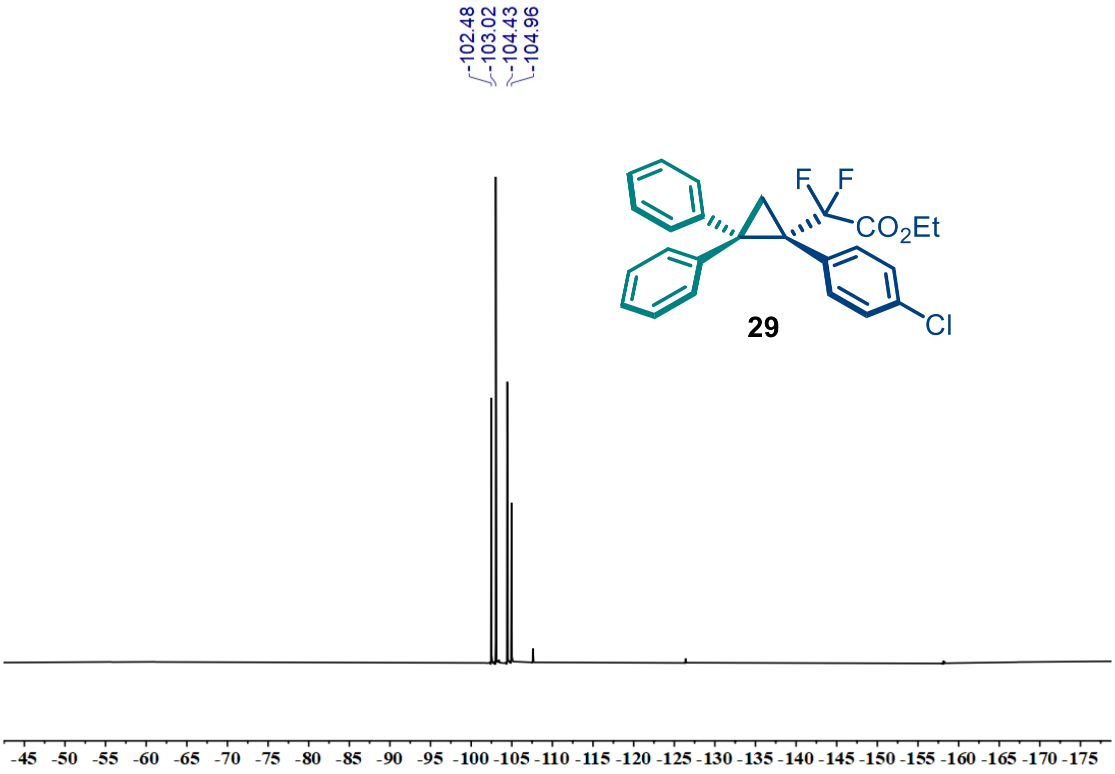

Figure S171, ^{19}F-NMR spectra copies of 29 related to scheme 2 and 3.

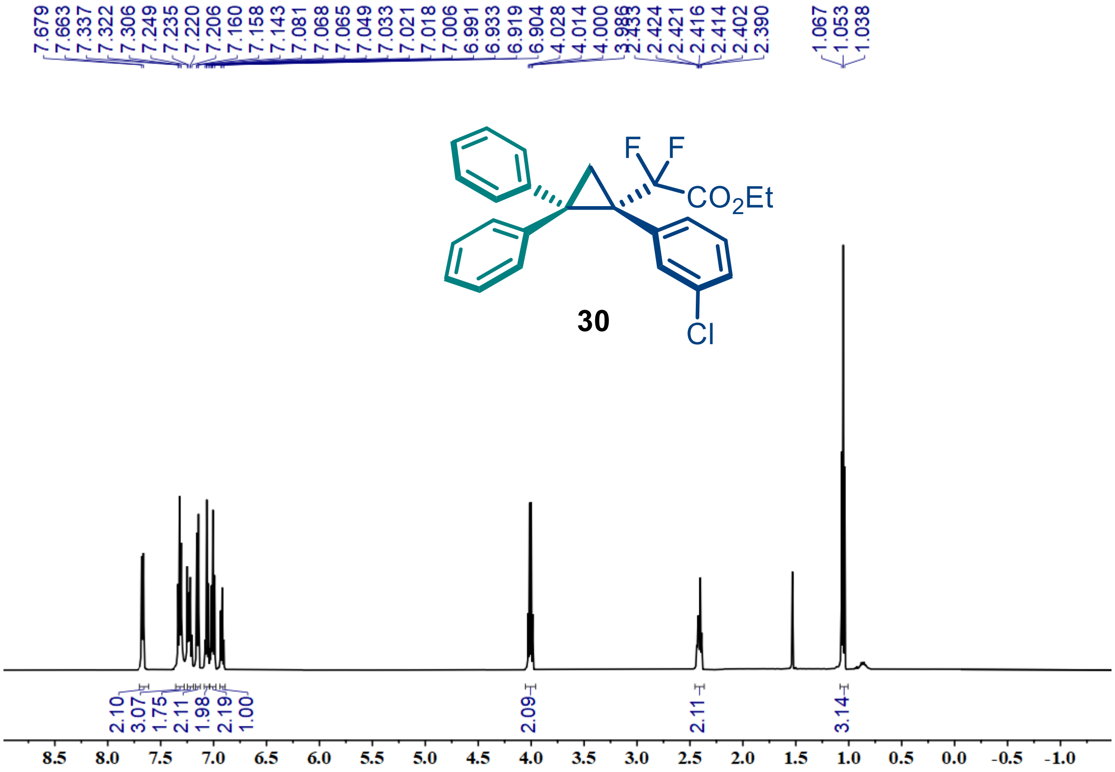

Figure S172, ^{1}H-NMR spectra copies of 30 related to scheme 2 and 3.

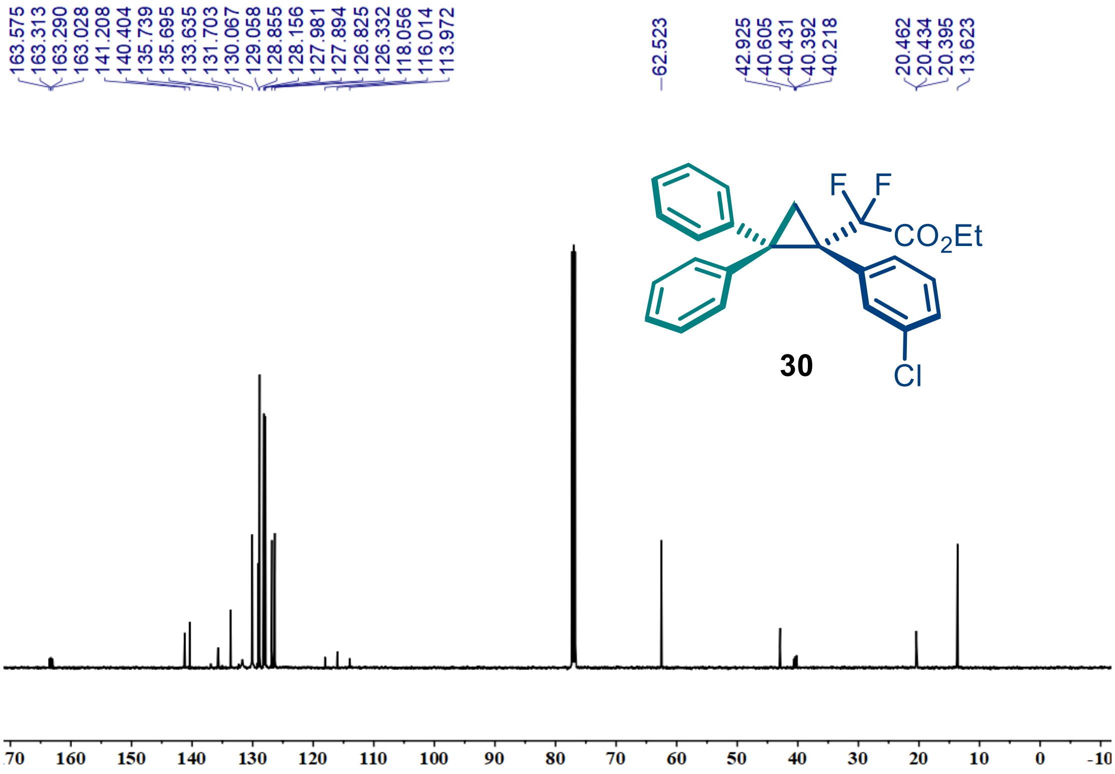

Figure S173, ^{13}C-NMR spectra copies of 30 related to scheme 2 and 3.

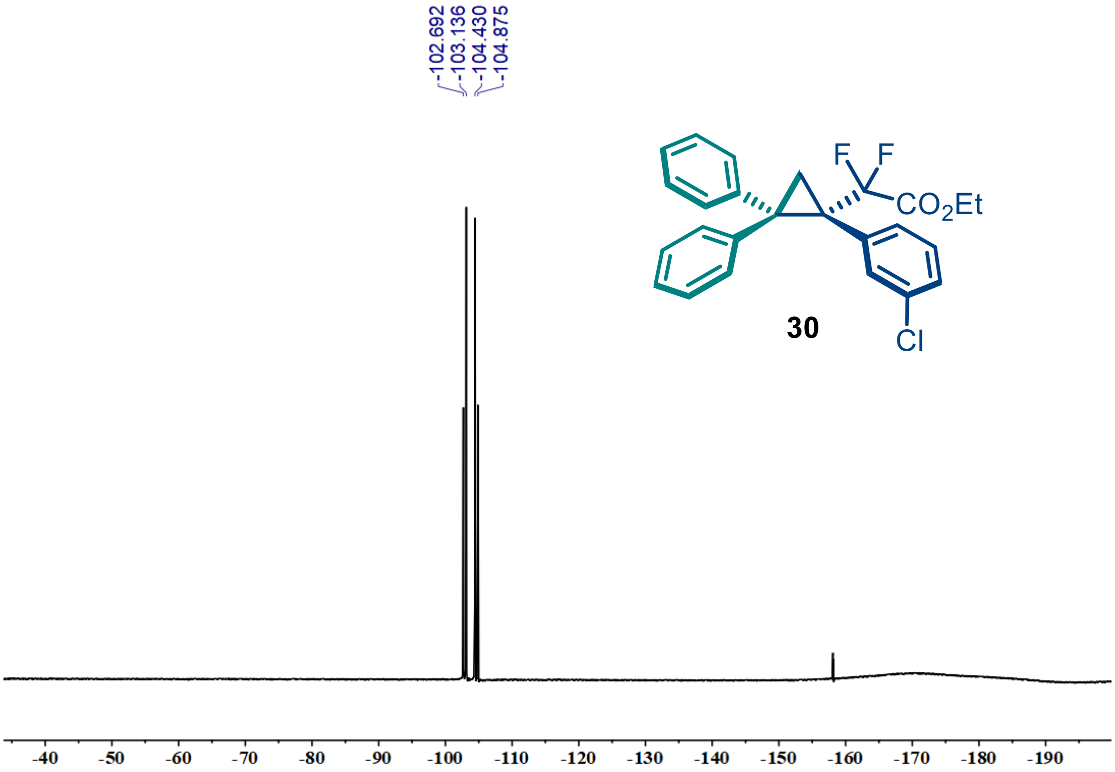

Figure S174, ^{19}F-NMR spectra copies of 30 related to scheme 2 and 3.

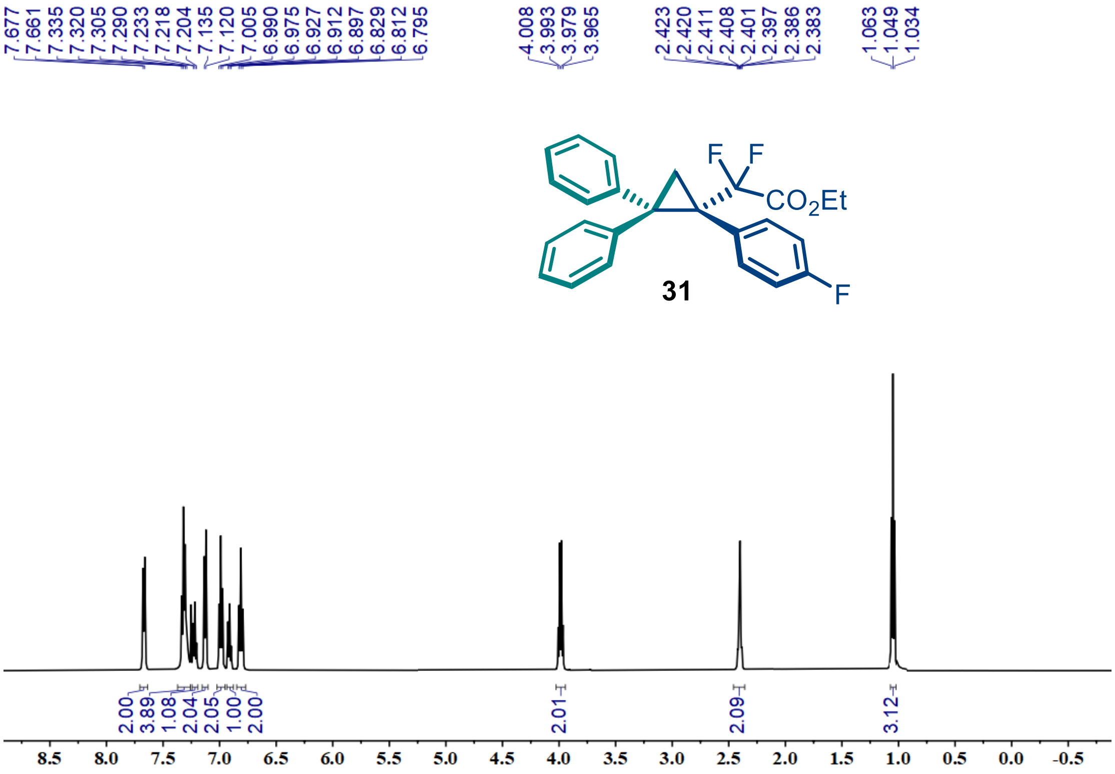

Figure S175, ^{1}H-NMR spectra copies of 31 related to scheme 2 and 3.

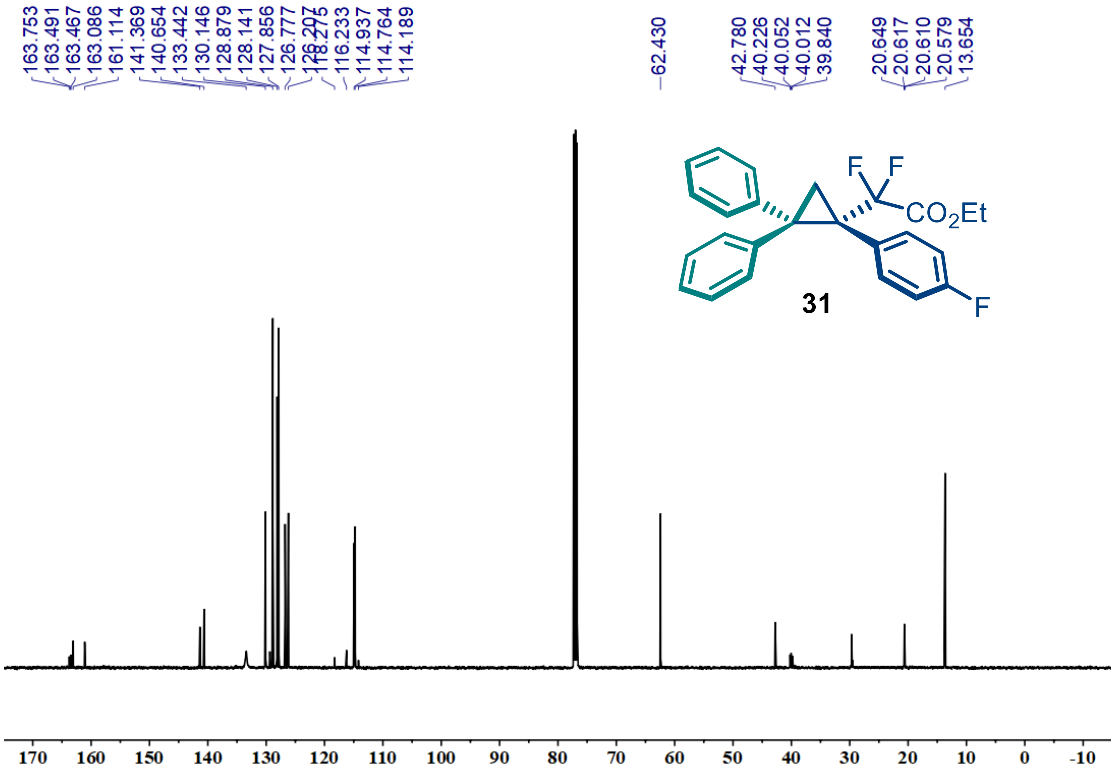

Figure S176, ^{13}C-NMR spectra copies of 31 related to scheme 2 and 3.

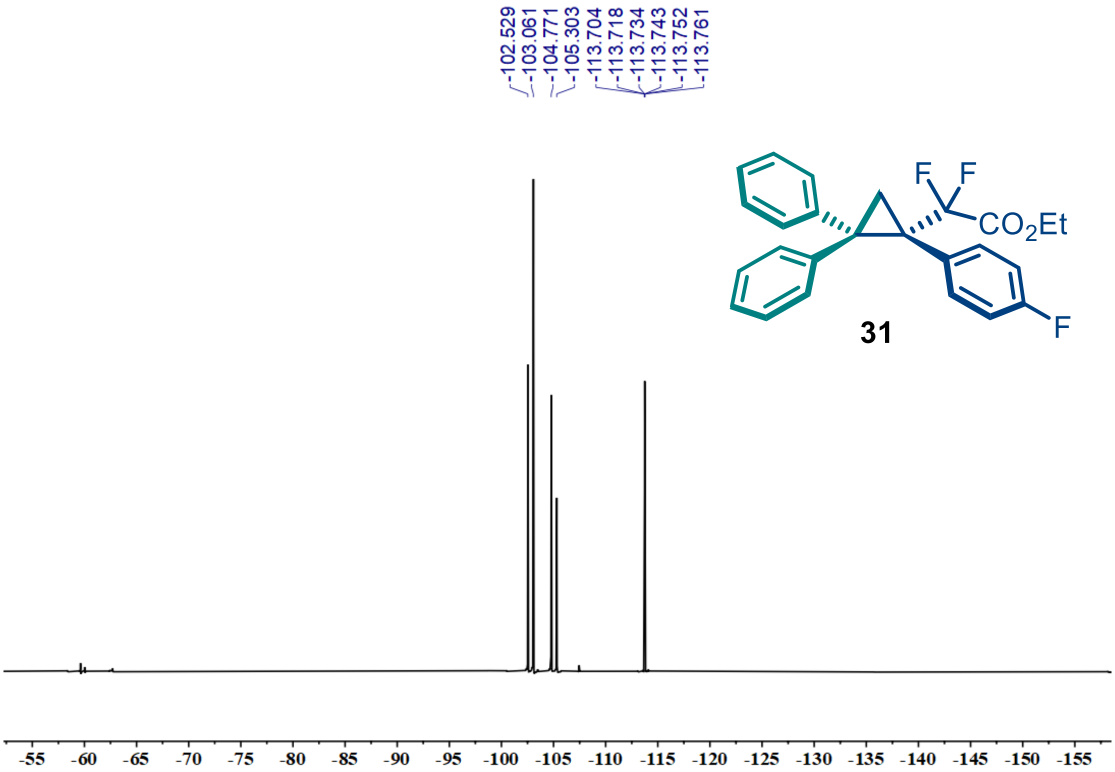

Figure S177, ^{19}F-NMR spectra copies of 31 related to scheme 2 and 3.

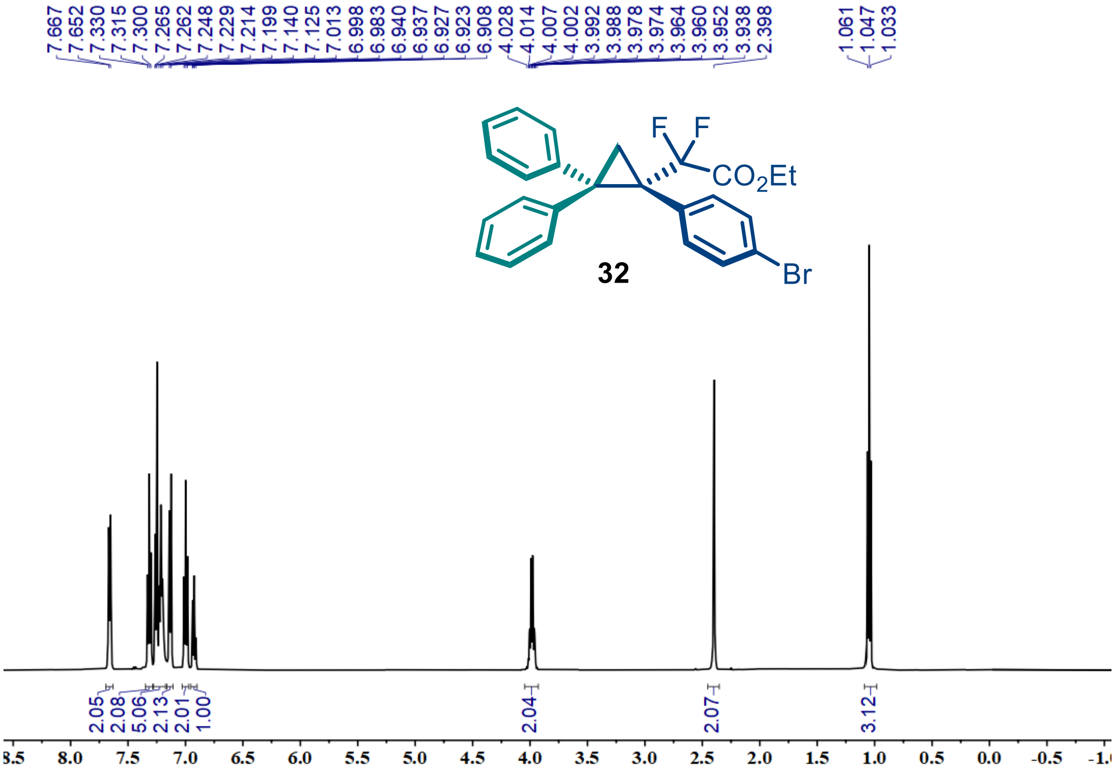

Figure S178, ^{1}H-NMR spectra copies of 32 related to scheme 2 and 3.

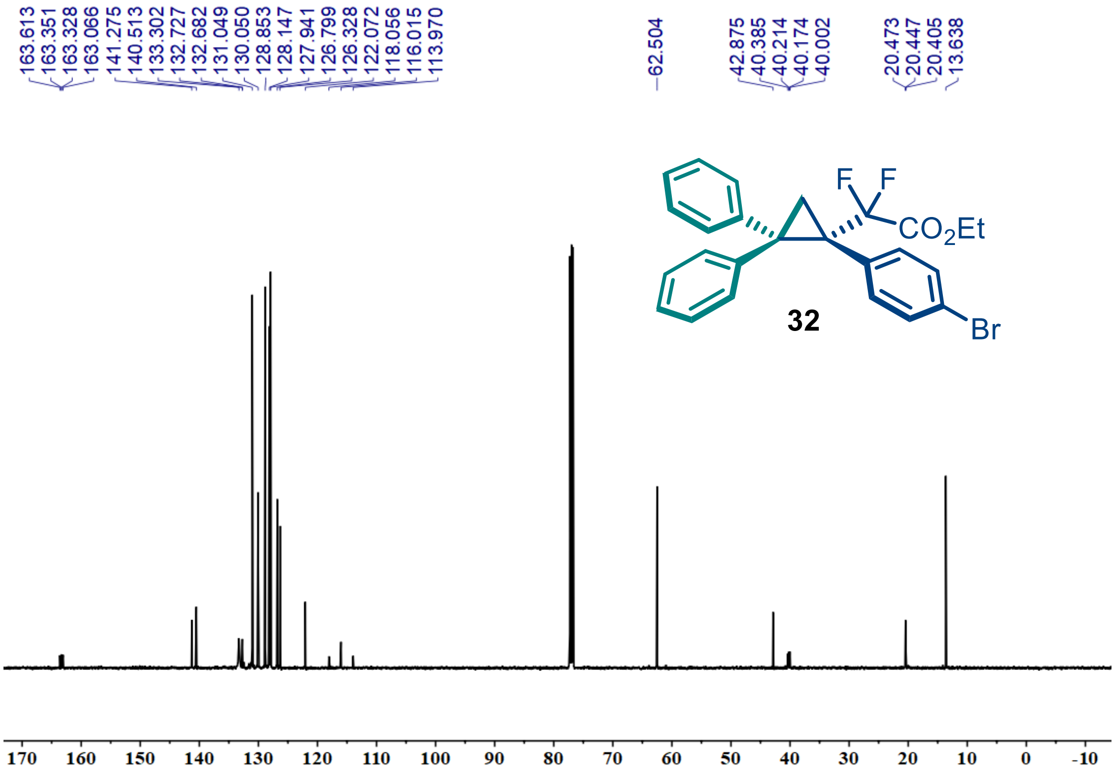

Figure S179, ^{13}C-NMR spectra copies of 32 related to scheme 2 and 3.

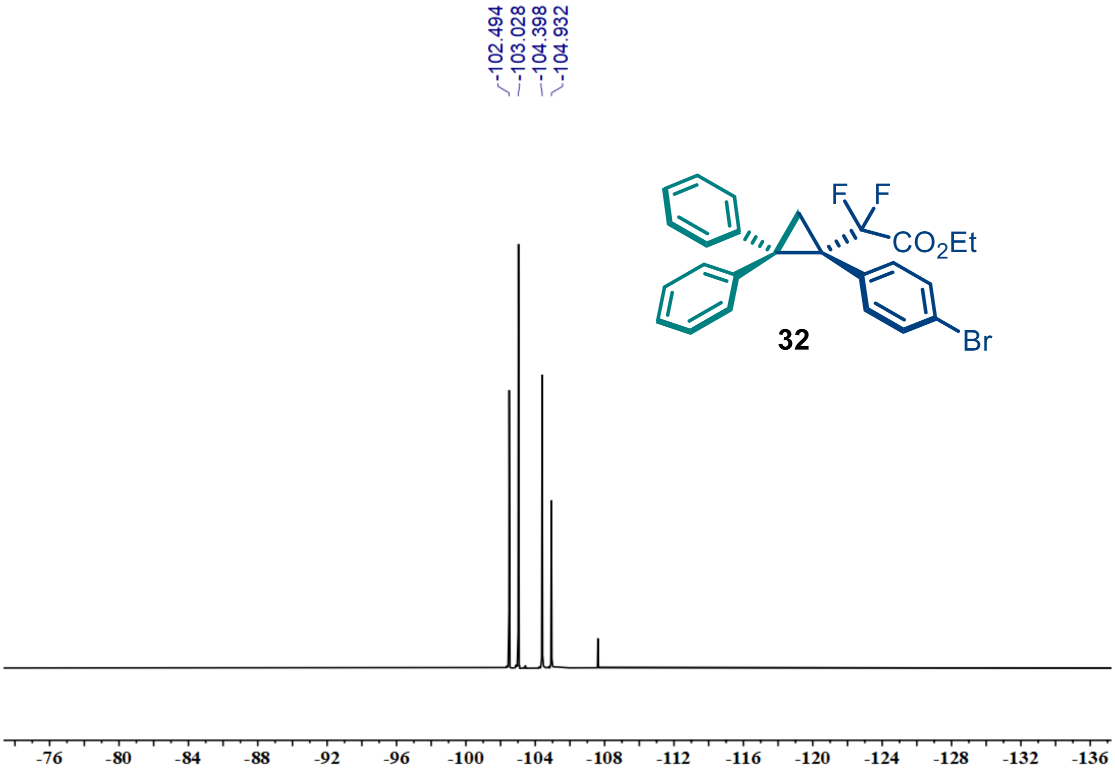

Figure S180, ^{19}F-NMR spectra copies of 32 related to scheme 2 and 3.

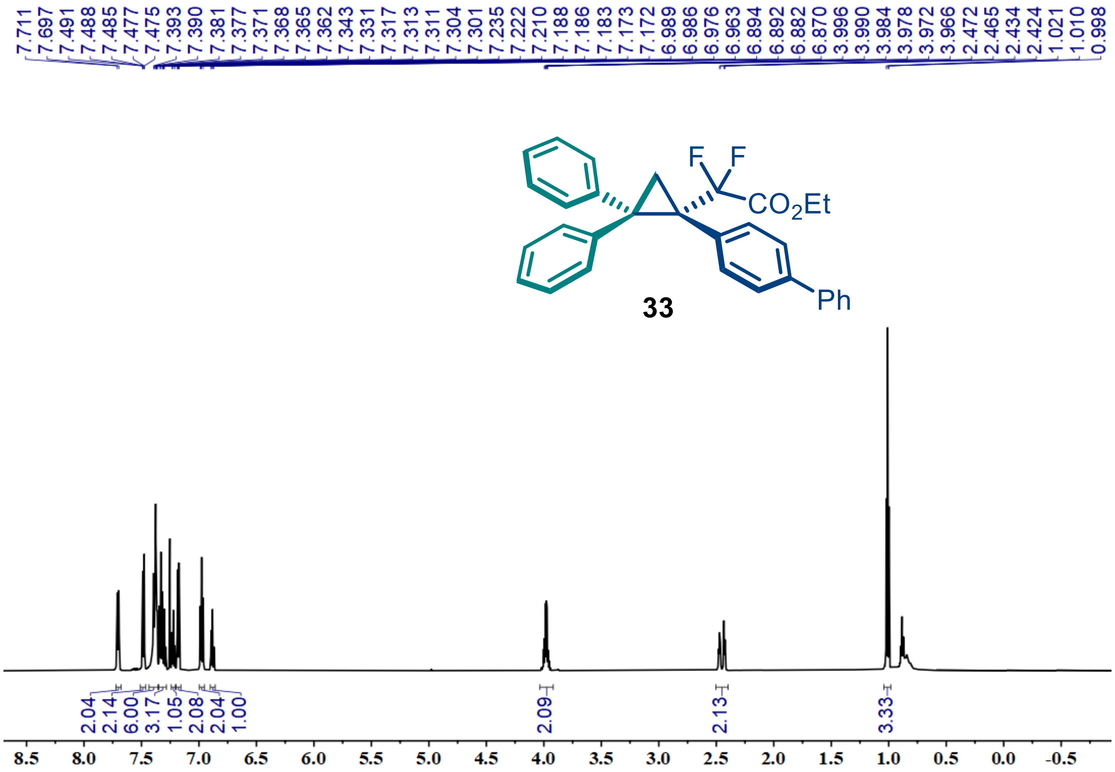

Figure S181, ^{1}H-NMR spectra copies of 33 related to scheme 2 and 3.

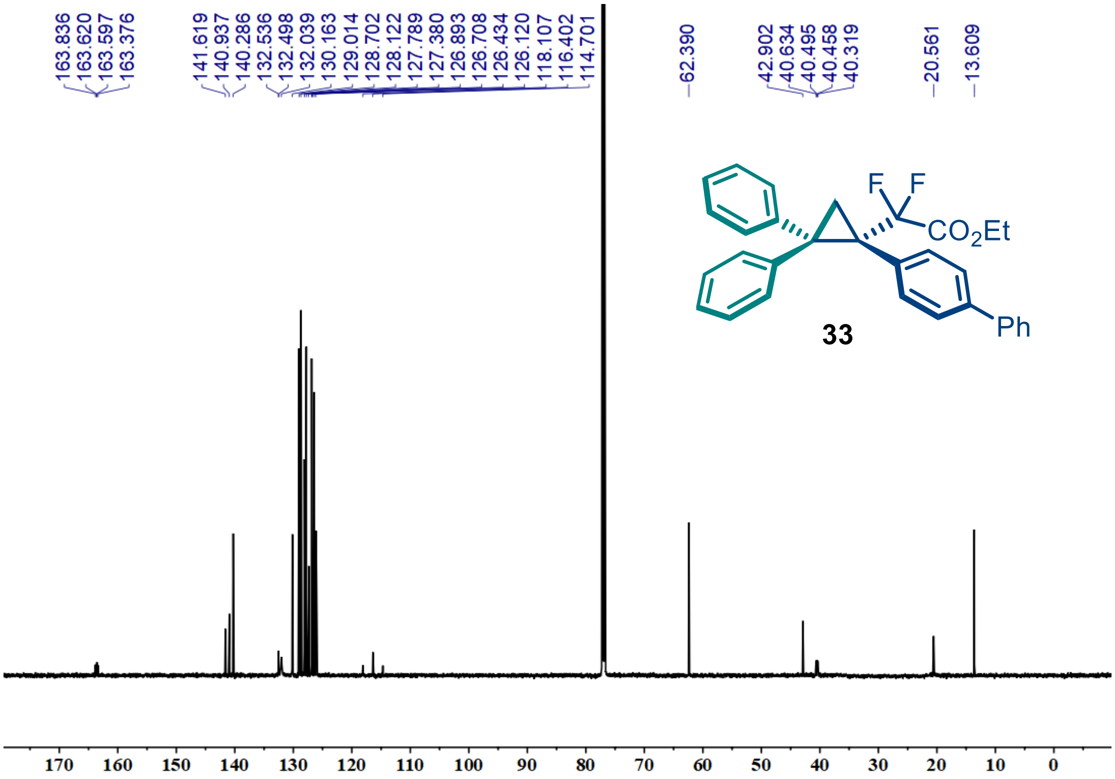

Figure S182, ^{13}C-NMR spectra copies of 33 related to scheme 2 and 3.

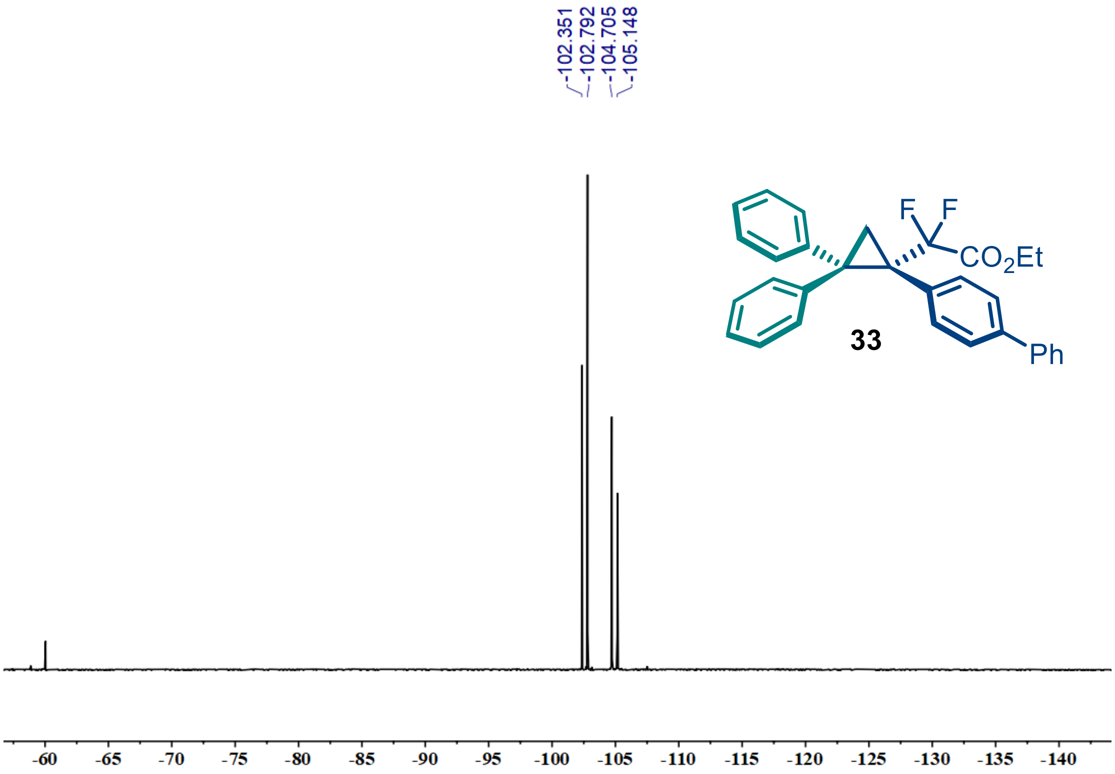

Figure S183, ^{19}F-NMR spectra copies of 33 related to scheme 2 and 3.

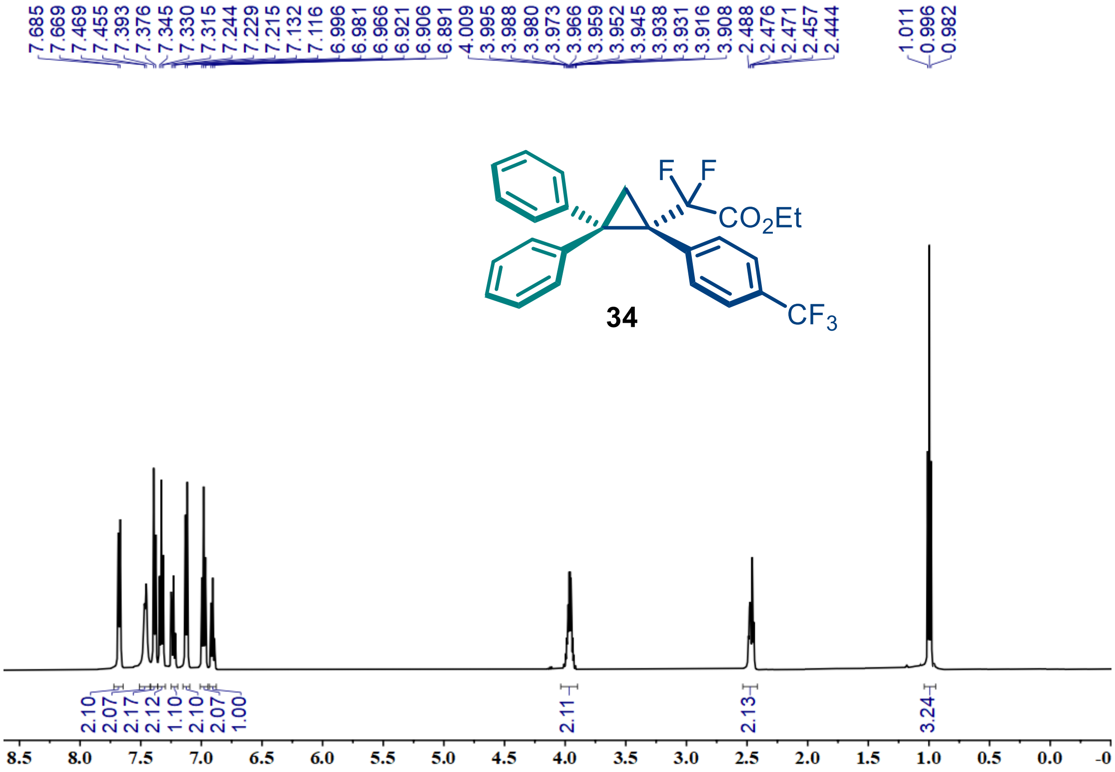

Figure S184, ^{1}H-NMR spectra copies of 34 related to scheme 2 and 3.

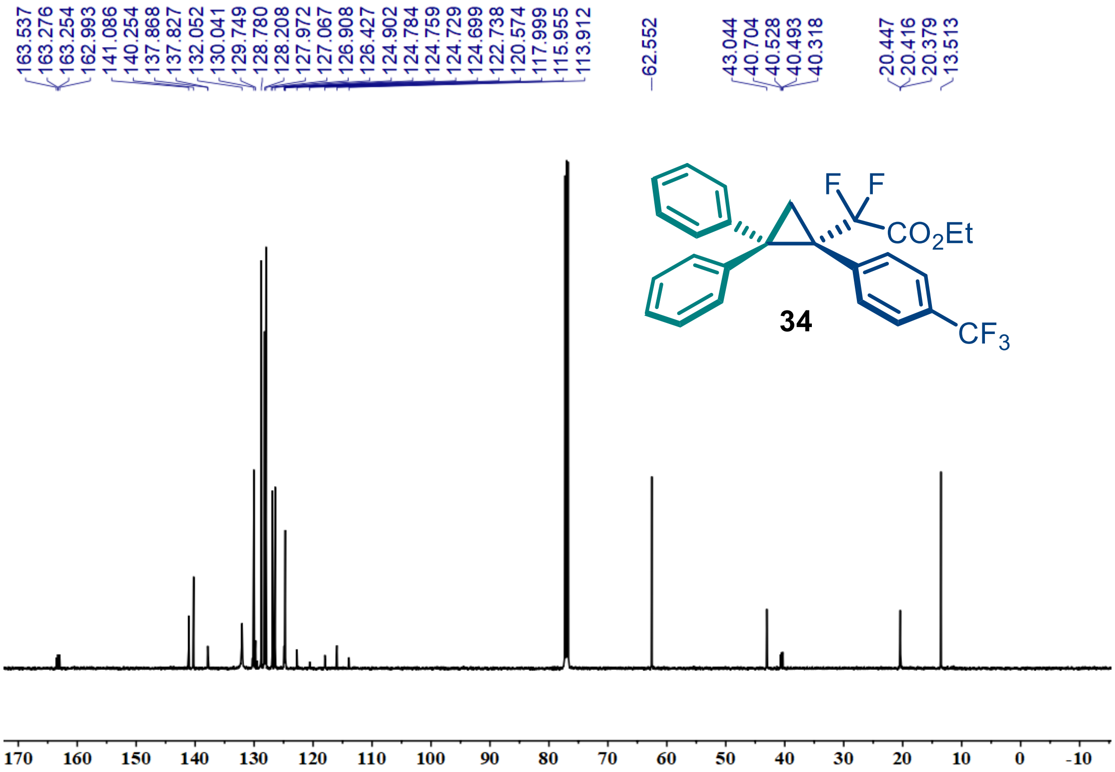

Figure S185, ^{13}C-NMR spectra copies of 34 related to scheme 2 and 3.

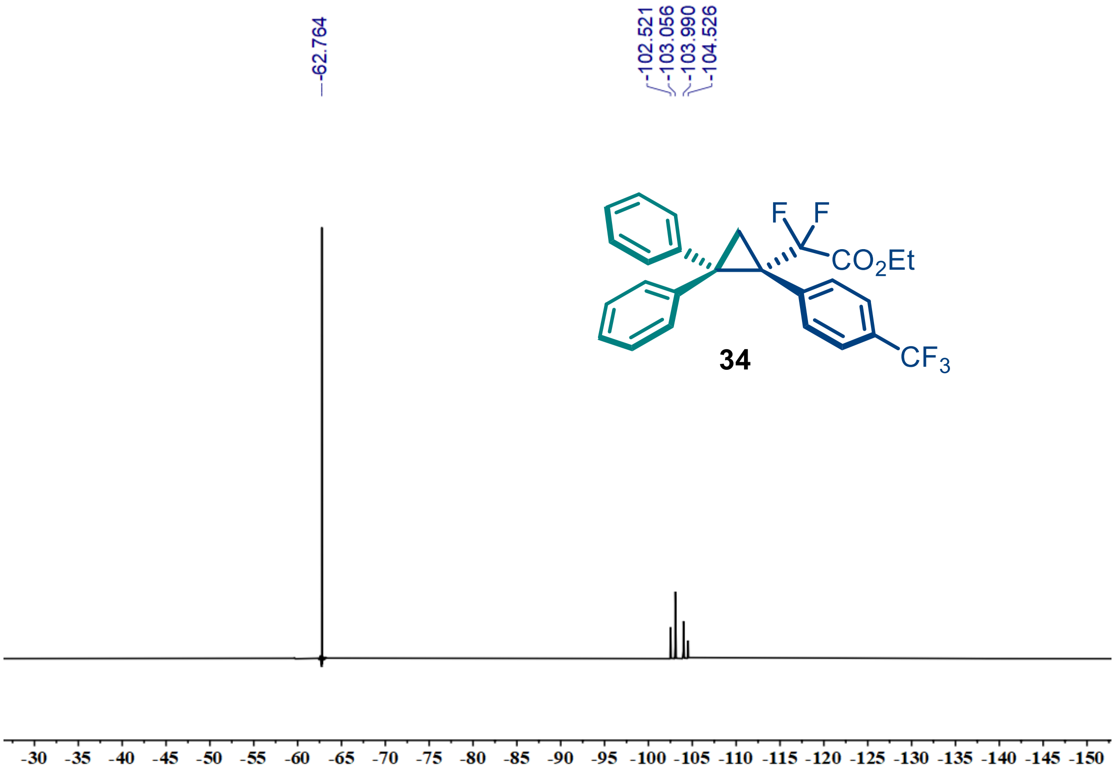

Figure S186, ^{19}F-NMR spectra copies of 34 related to scheme 2 and 3

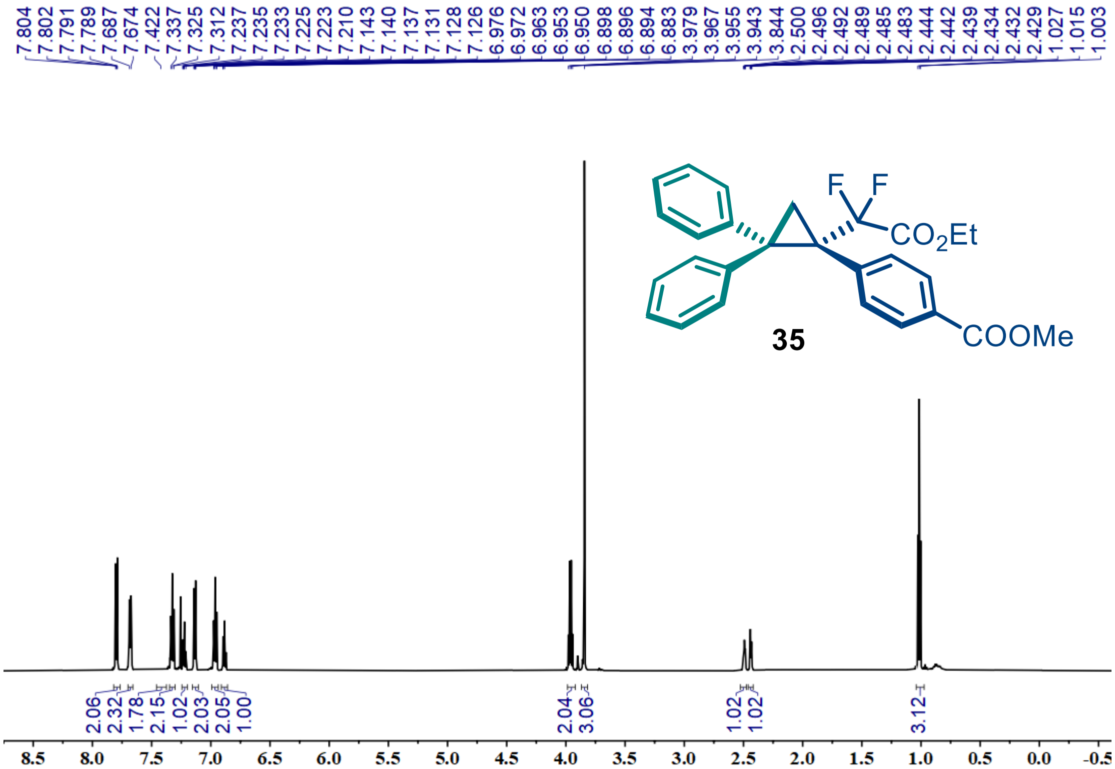

Figure S187, ^{1}H-NMR spectra copies of 35 related to scheme 2 and 3.

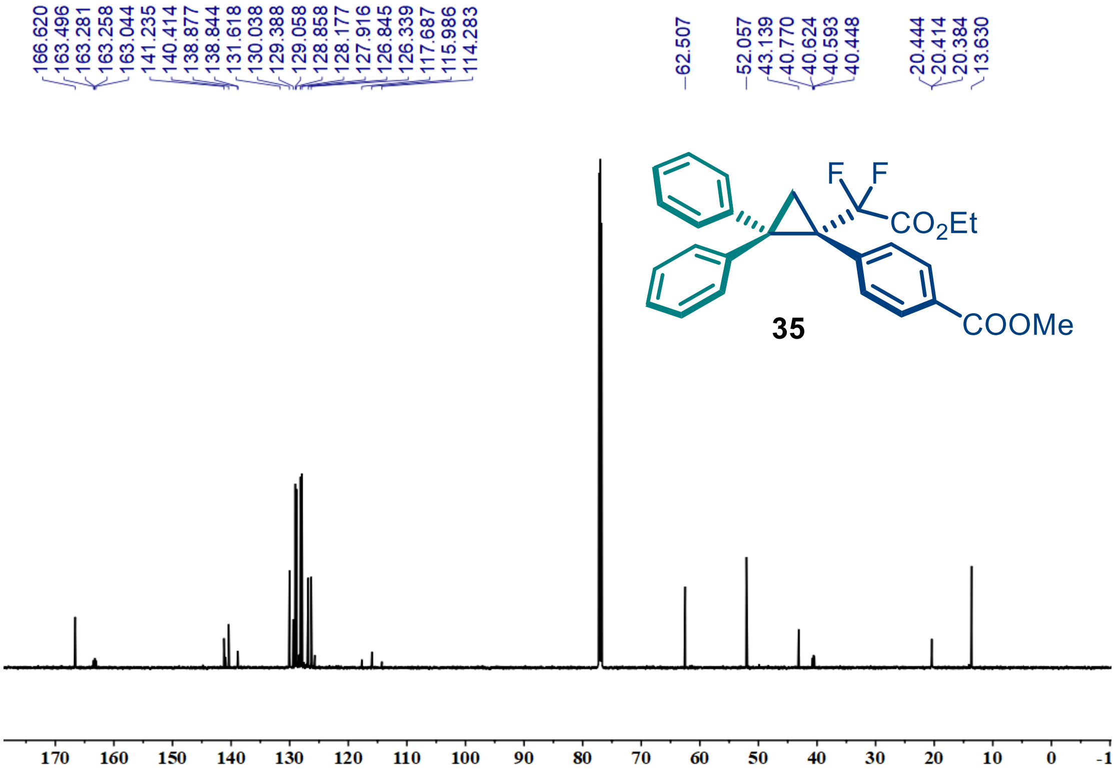

Figure S188, ^{13}C-NMR spectra copies of 35 related to scheme 2 and 3.

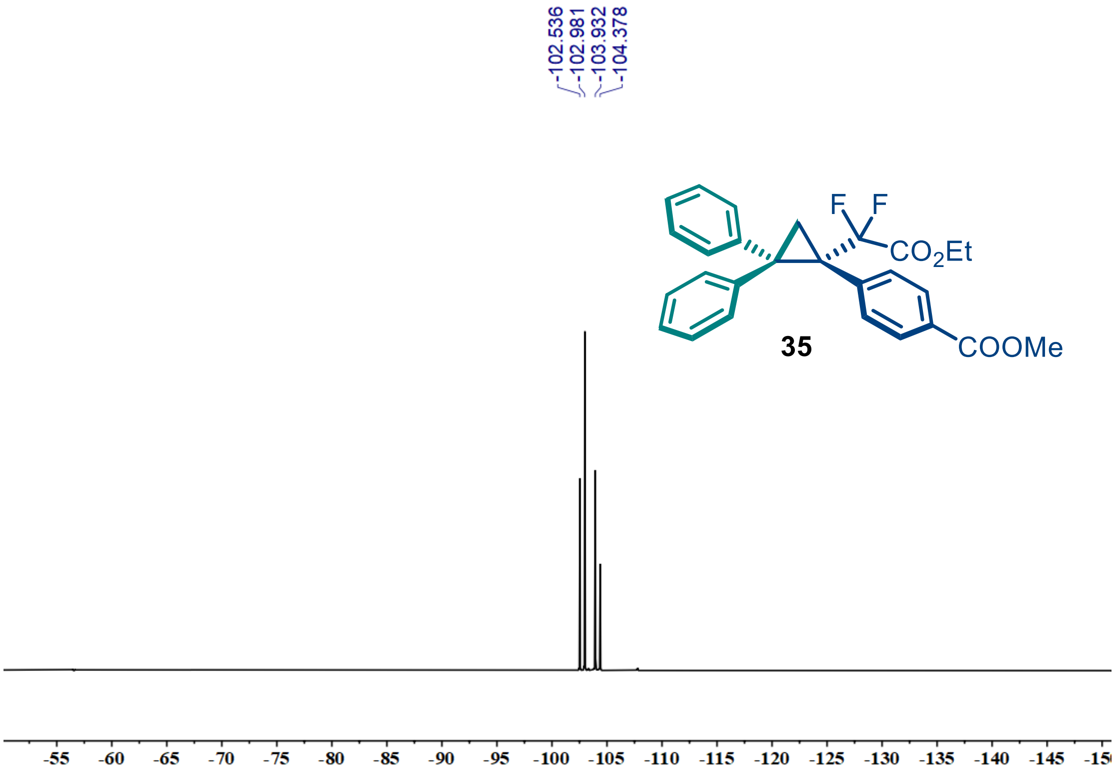

Figure S189, ^{19}F-NMR spectra copies of 35 related to scheme 2 and 3

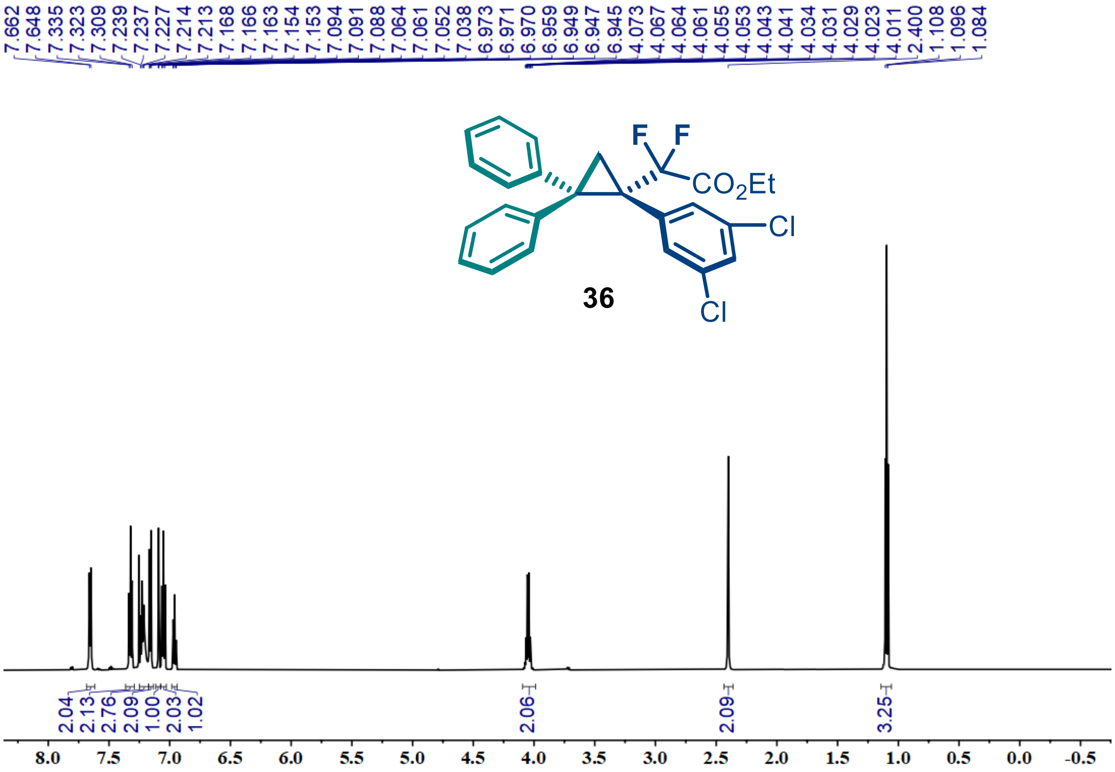

Figure S190, ^{1}H-NMR spectra copies of 36 related to scheme 2 and 3.

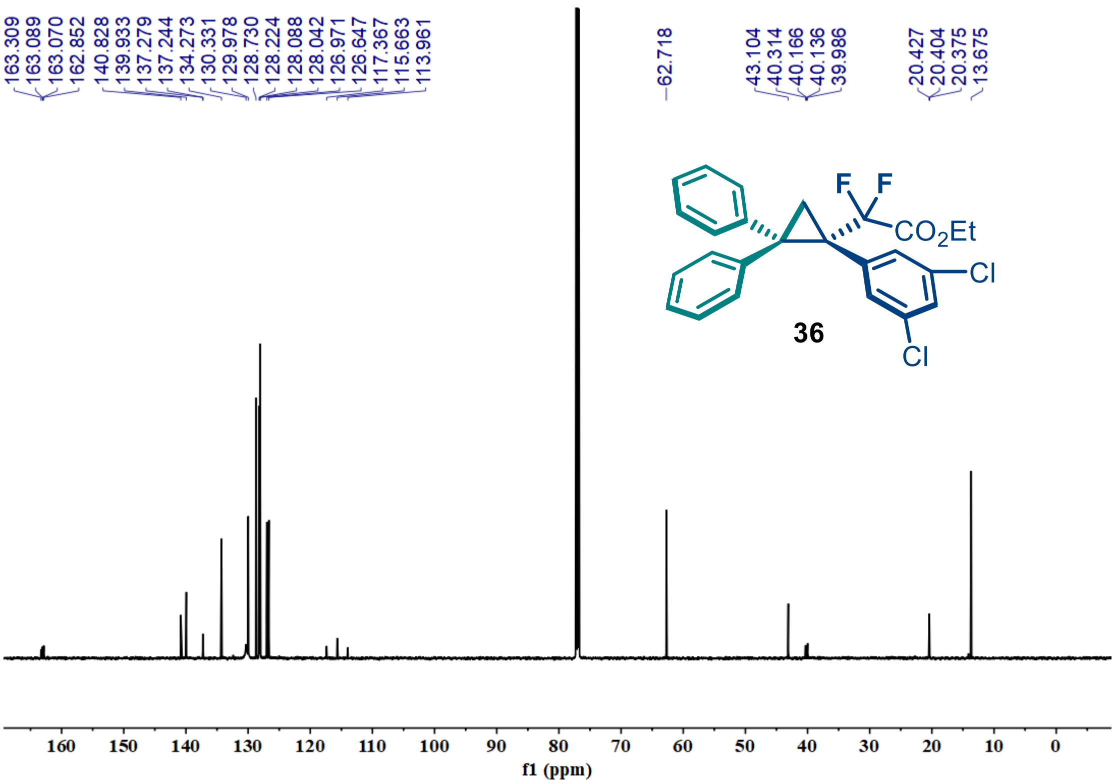

Figure S191, ^{13}C-NMR spectra copies of 36 related to scheme 2 and 3.

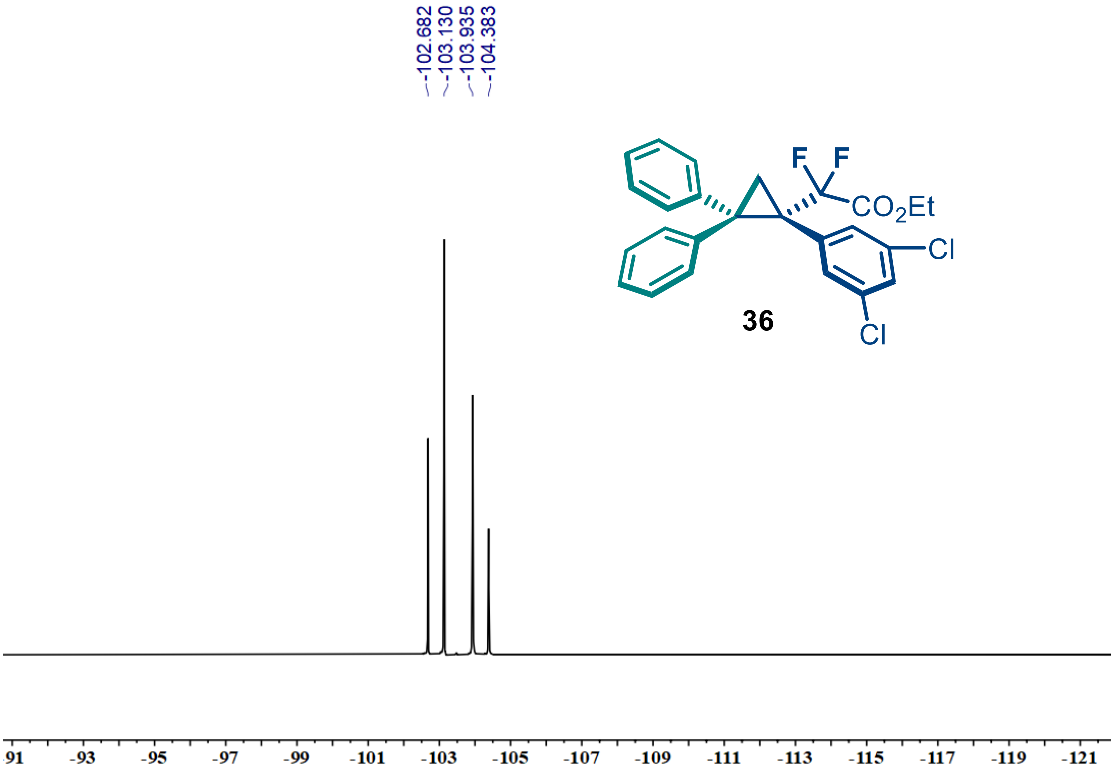

Figure S192, ^{19}F-NMR spectra copies of 36 related to scheme 2 and 3

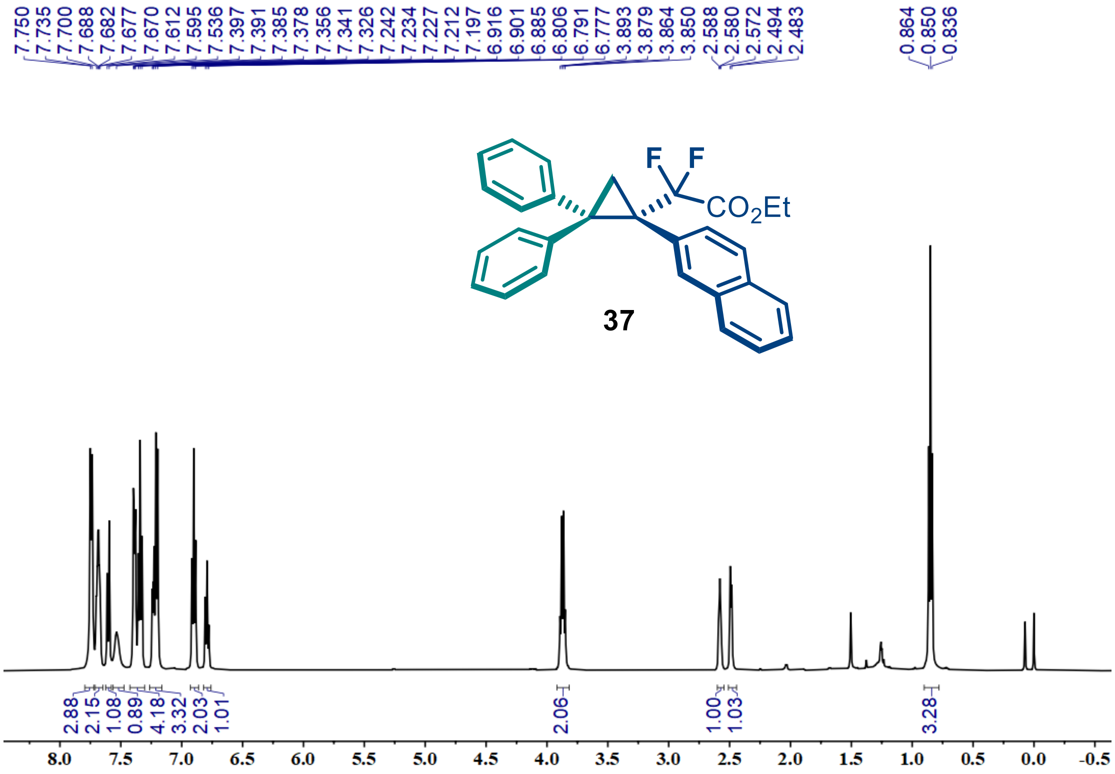

Figure S193, ^{1}H-NMR spectra copies of 37 related to scheme 2 and 3.

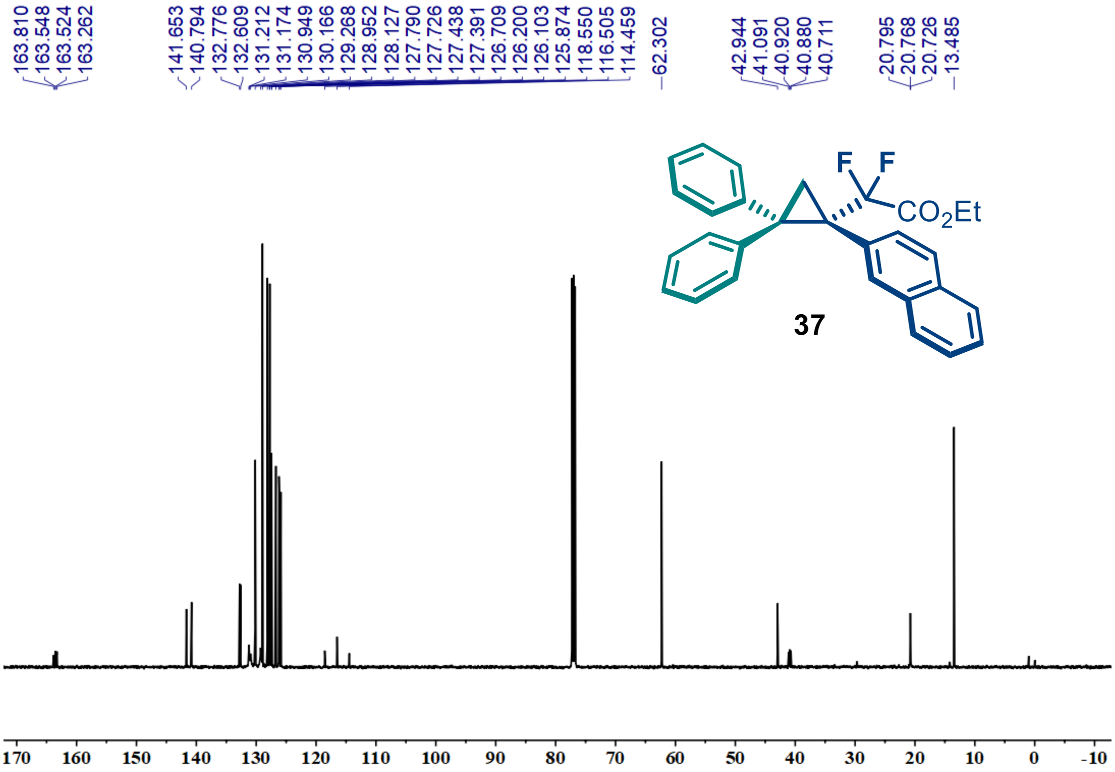

Figure S194, ^{13}C-NMR spectra copies of 37 related to scheme 2 and 3.

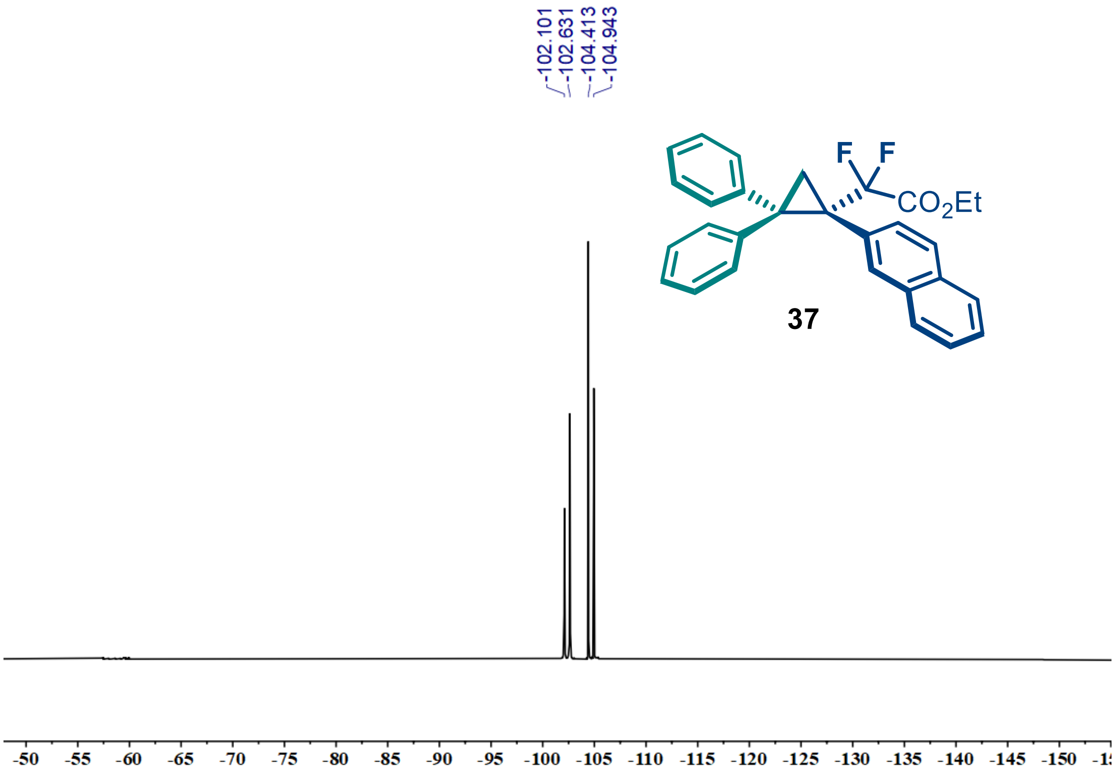

Figure S195, ^{19}F-NMR spectra copies of 37 related to scheme 2 and 3

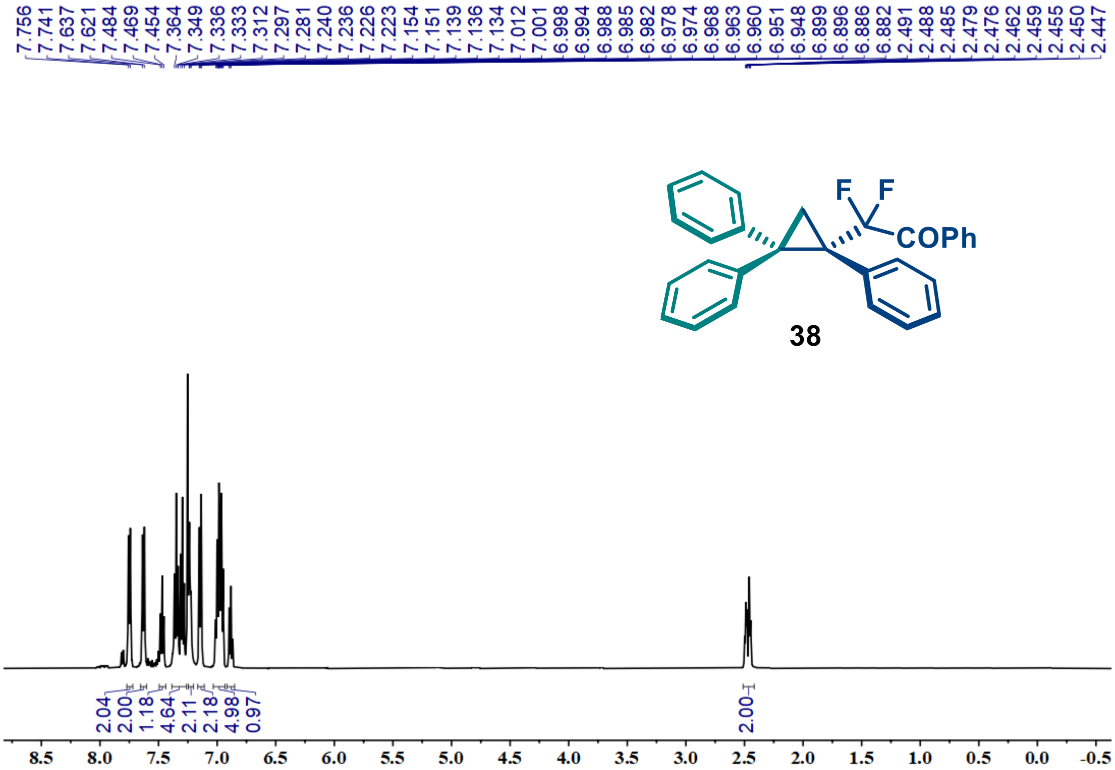

Figure S196, ^{1}H-NMR spectra copies of 38 related to scheme 2 and 3.

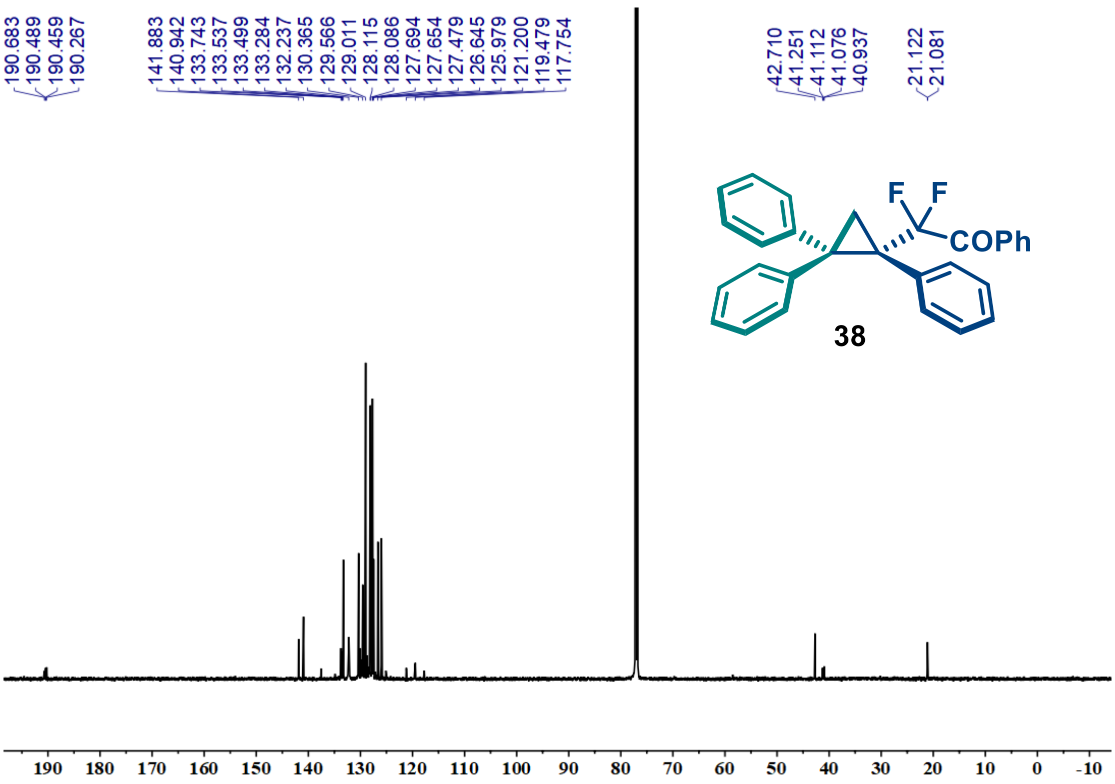

Figure S197, ^{13}C-NMR spectra copies of 38 related to scheme 2 and 3.

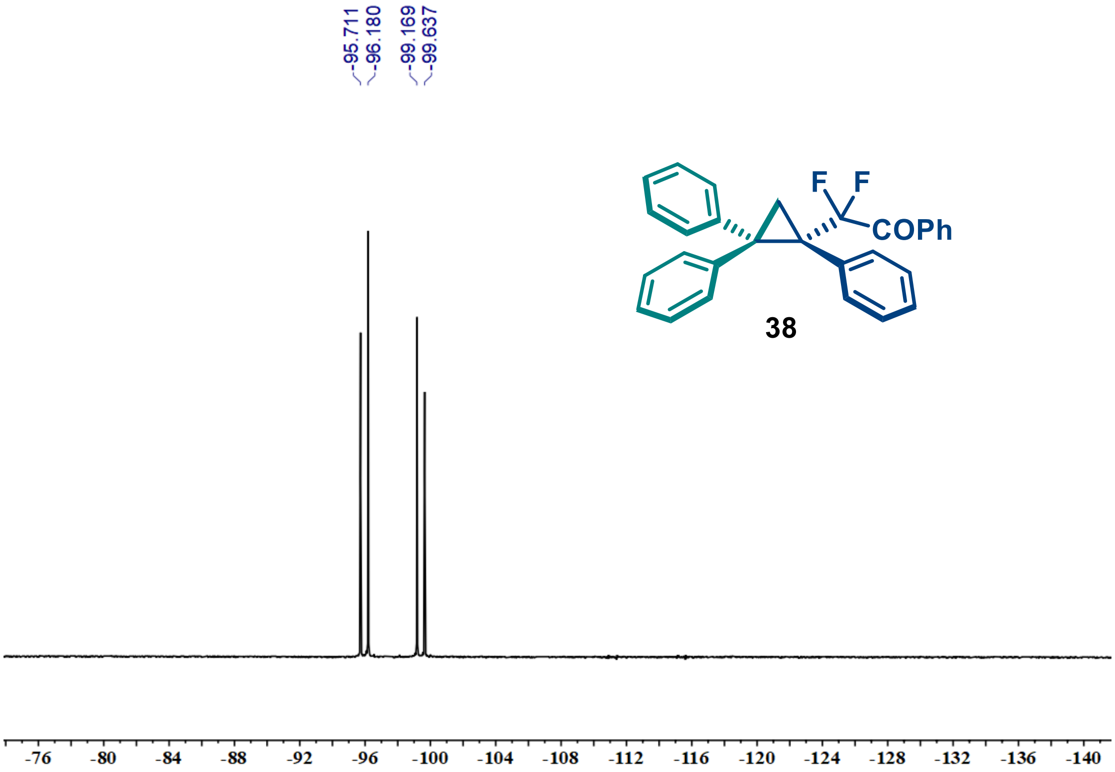

Figure S198, ^{19}F-NMR spectra copies of 38 related to scheme 2 and 3

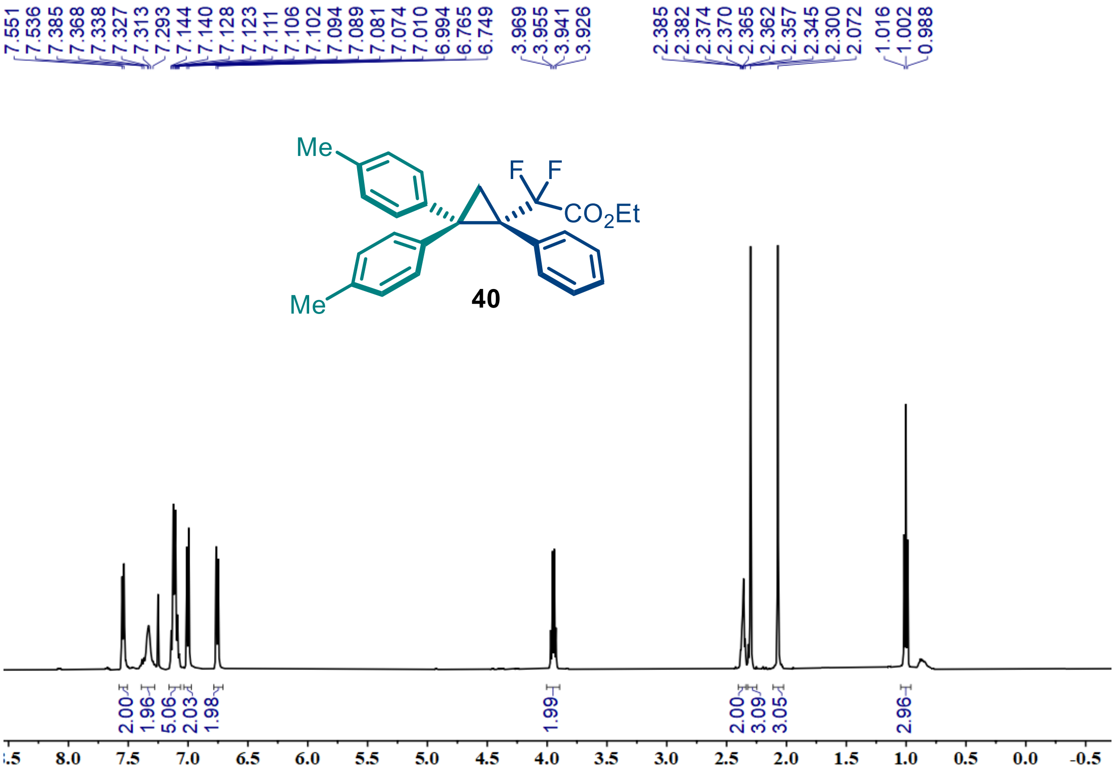

Figure S199, ^{1}H-NMR spectra copies of 40 related to scheme 2 and 3.

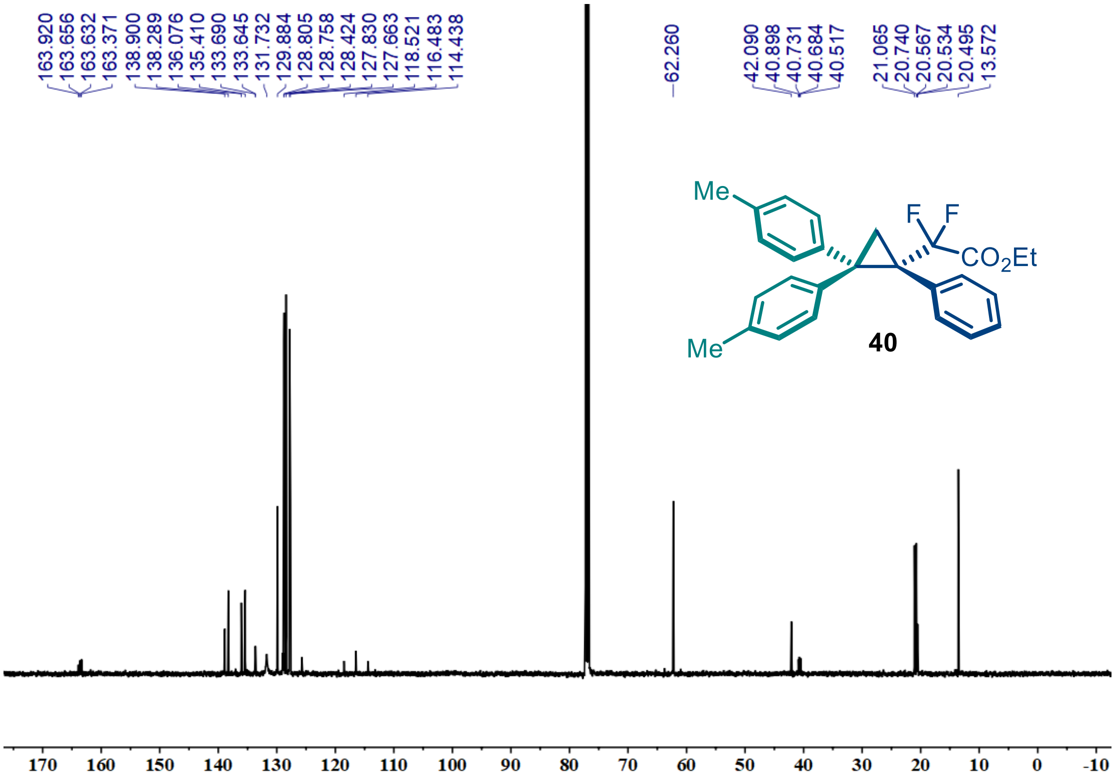

Figure S200, ^{13}C-NMR spectra copies of 40 related to scheme 2 and 3.

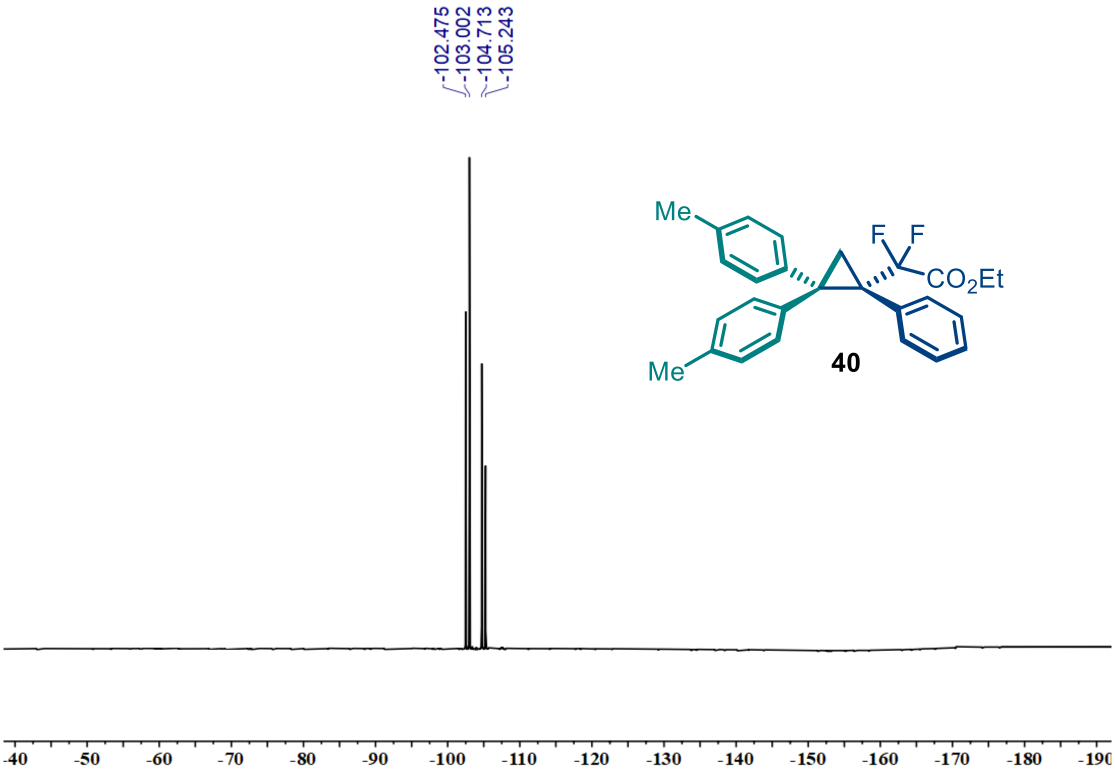

Figure S201, ^{19}F-NMR spectra copies of 40 related to scheme 2 and 3

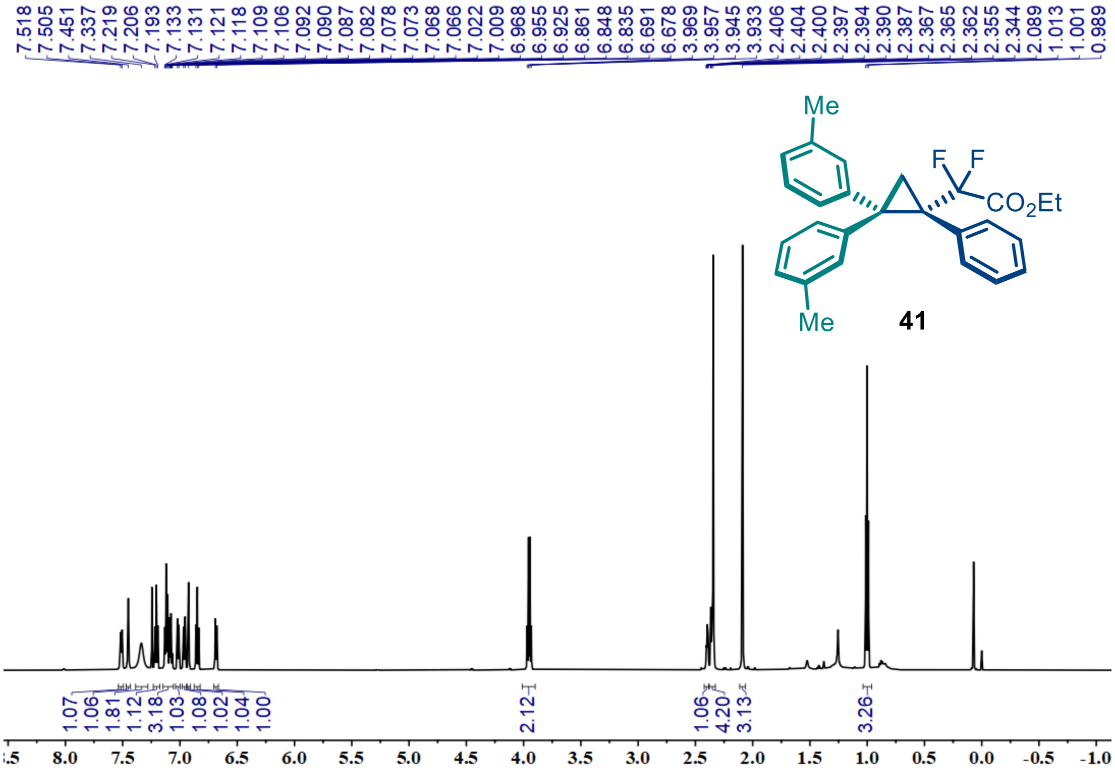

Figure S202, ^{1}H-NMR spectra copies of 41 related to scheme 2 and 3.

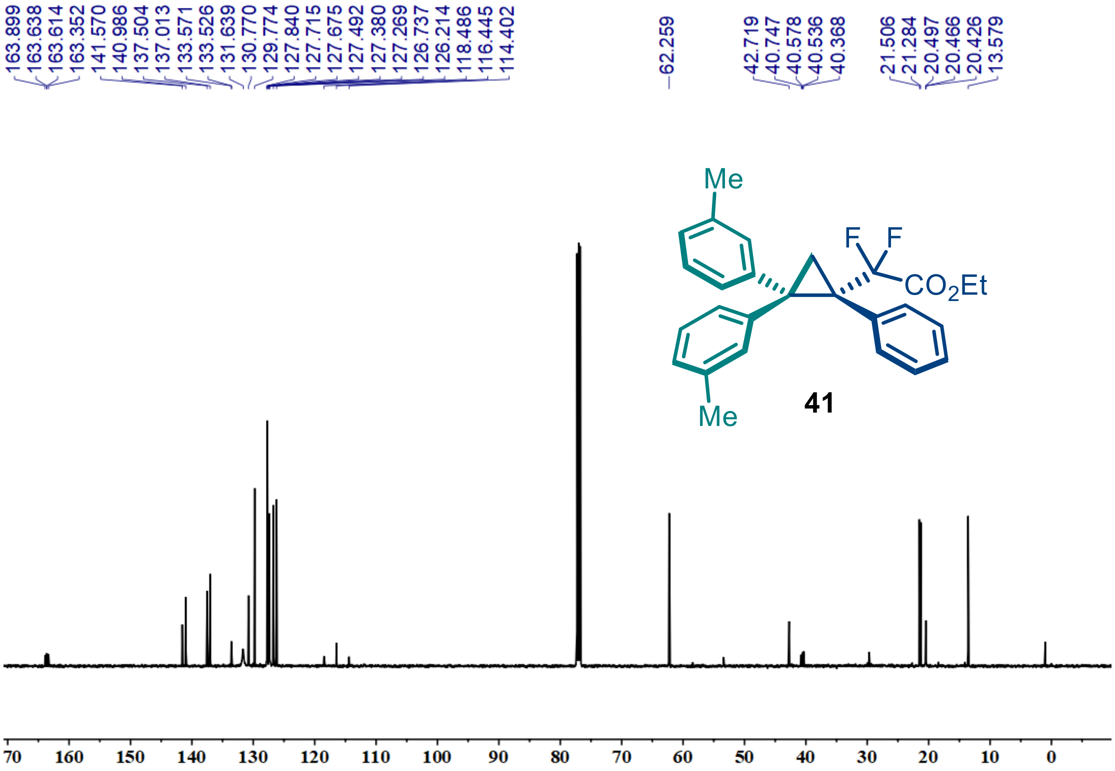

Figure S203, ^{13}C-NMR spectra copies of 41 related to scheme 2 and 3.

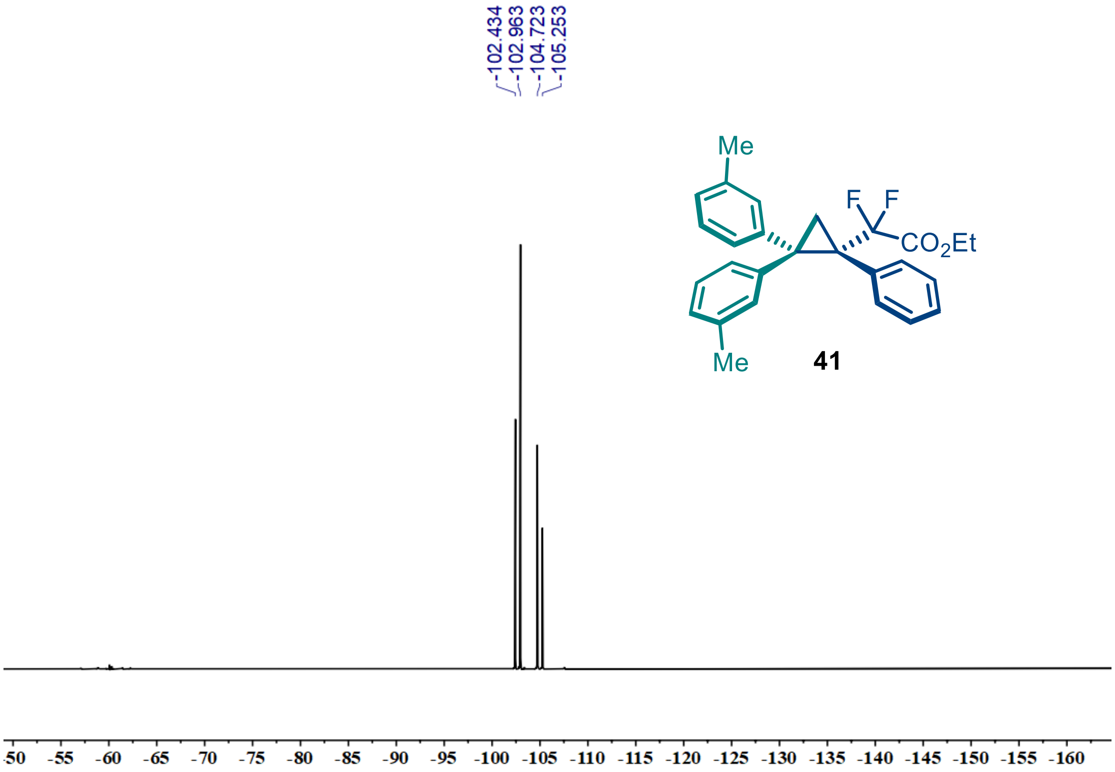

Figure S204, ^{19}F-NMR spectra copies of 41 related to scheme 2 and 3

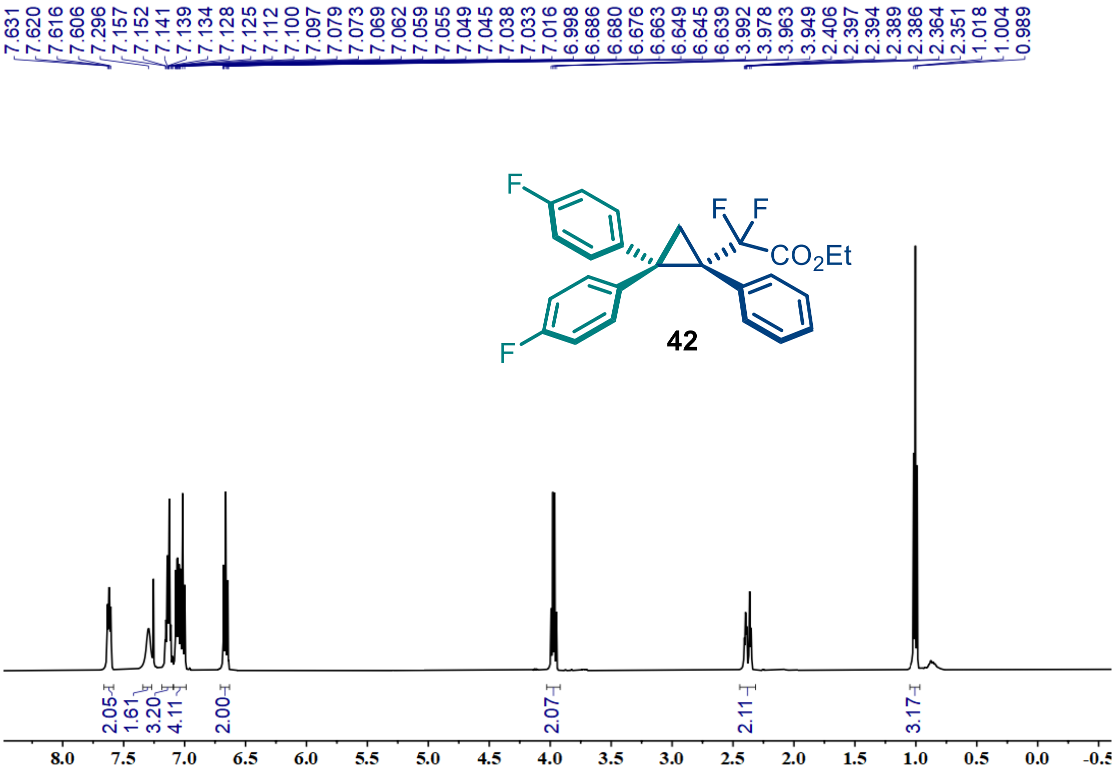

Figure S205, ^{1}H-NMR spectra copies of 42 related to scheme 2 and 3.

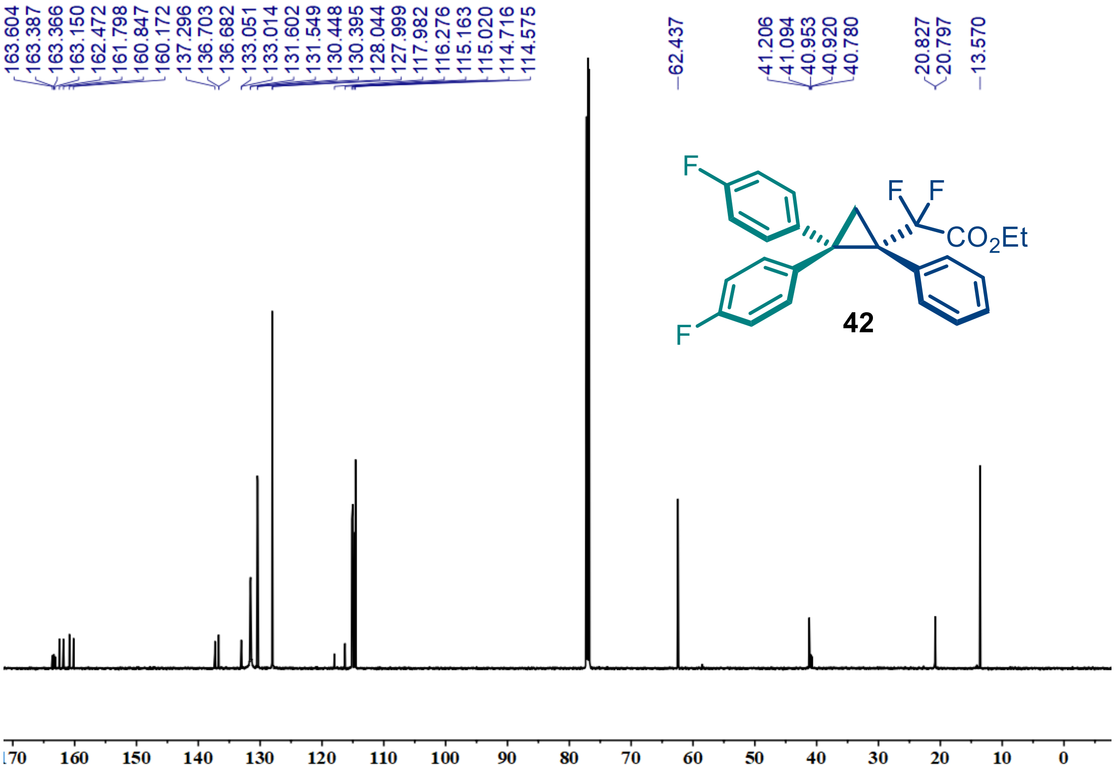

Figure S206, ^{13}C-NMR spectra copies of 42 related to scheme 2 and 3.

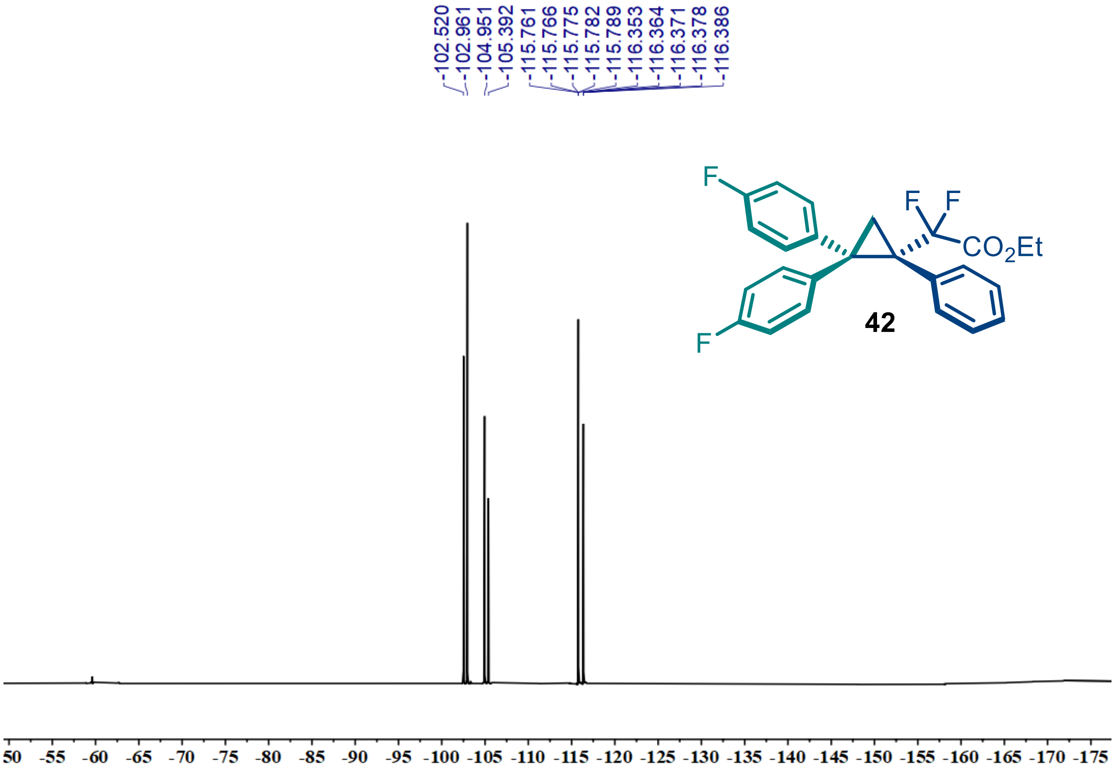

Figure S207, ^{19}F-NMR spectra copies of 42 related to scheme 2 and 3

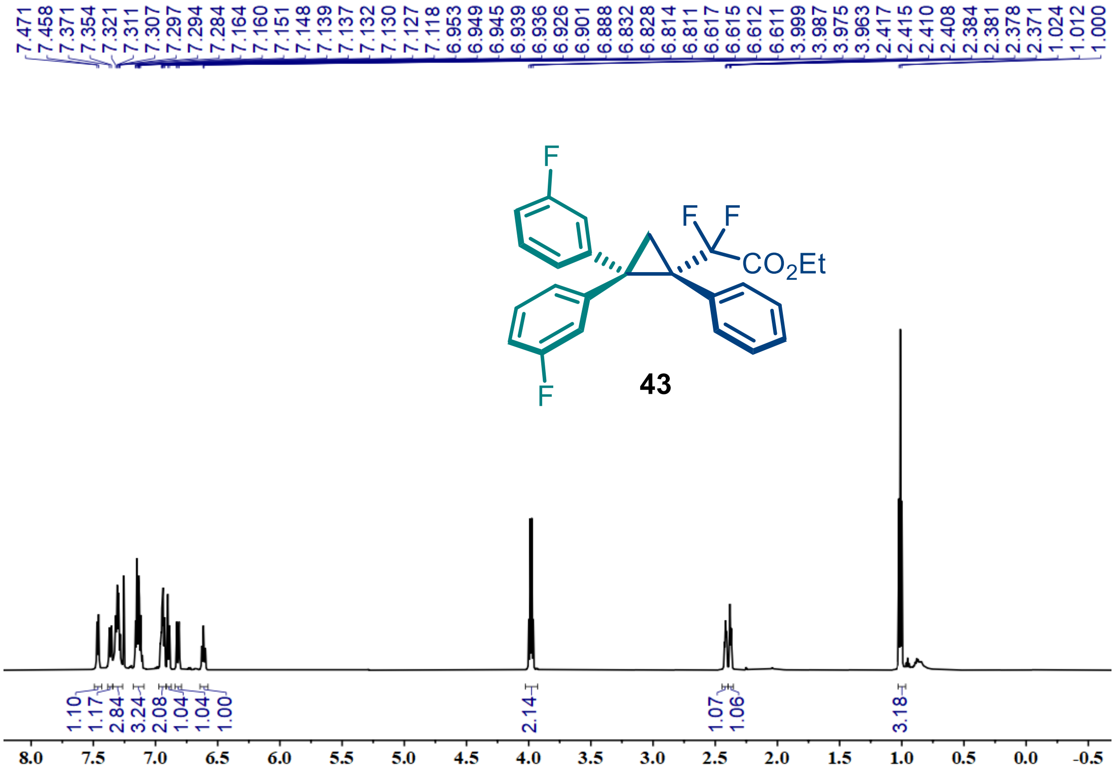

Figure S208, ^{1}H-NMR spectra copies of 43 related to scheme 2 and 3.

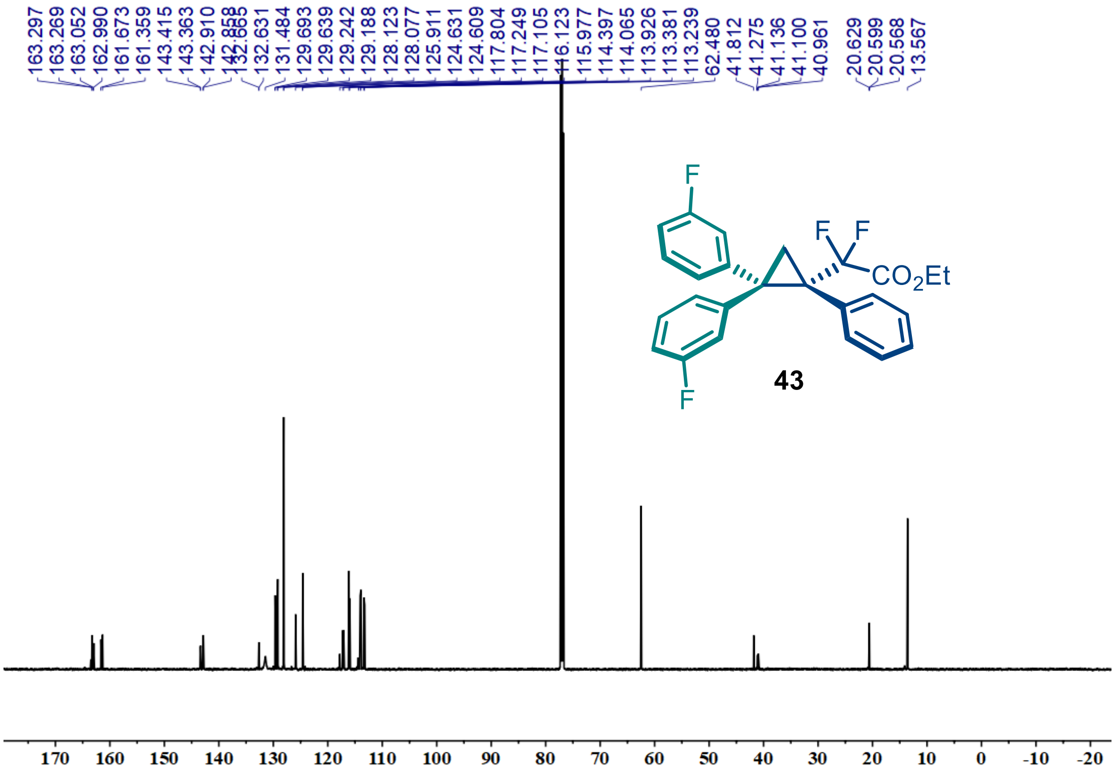

Figure S209, ^{13}C-NMR spectra copies of 43 related to scheme 2 and 3.

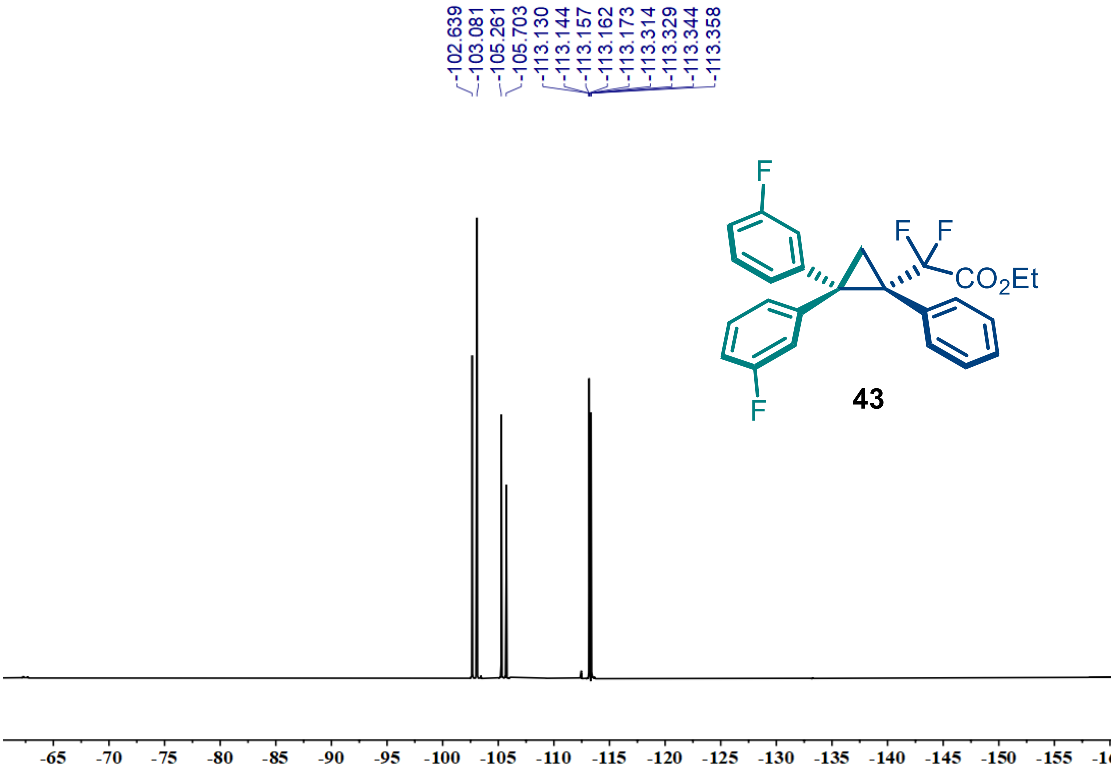

Figure S210, ^{19}F-NMR spectra copies of 43 related to scheme 2 and 3

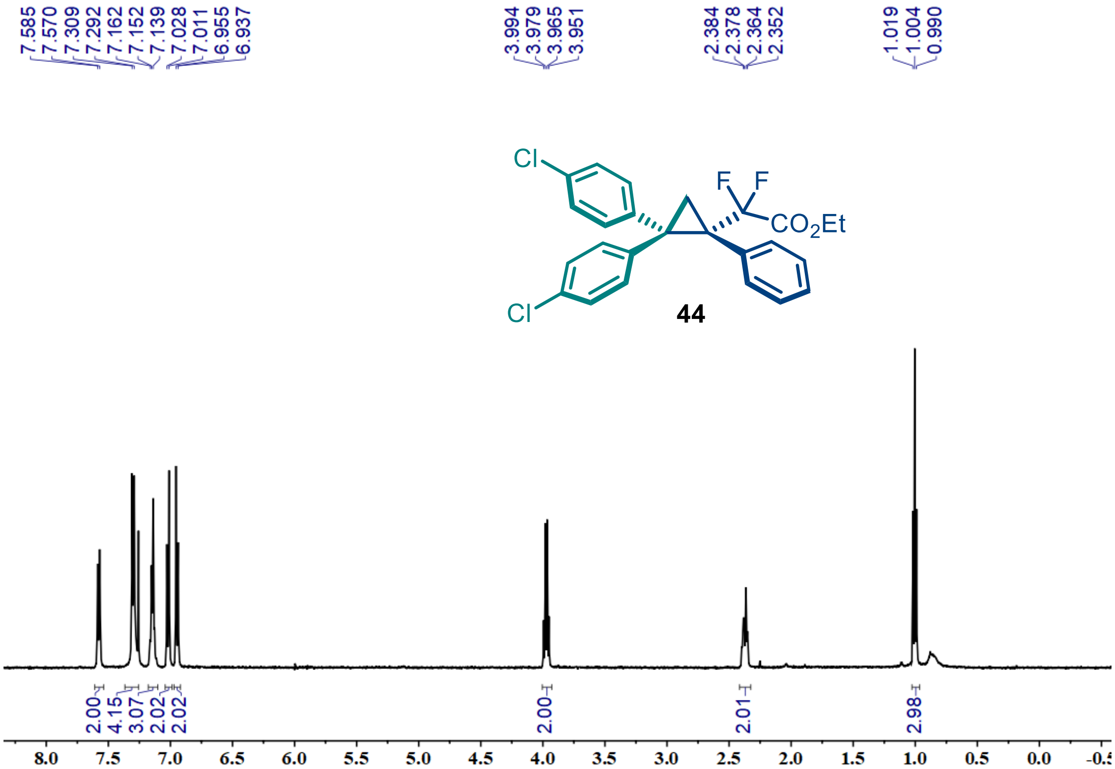

Figure S211, ^{1}H-NMR spectra copies of 44 related to scheme 2 and 3.

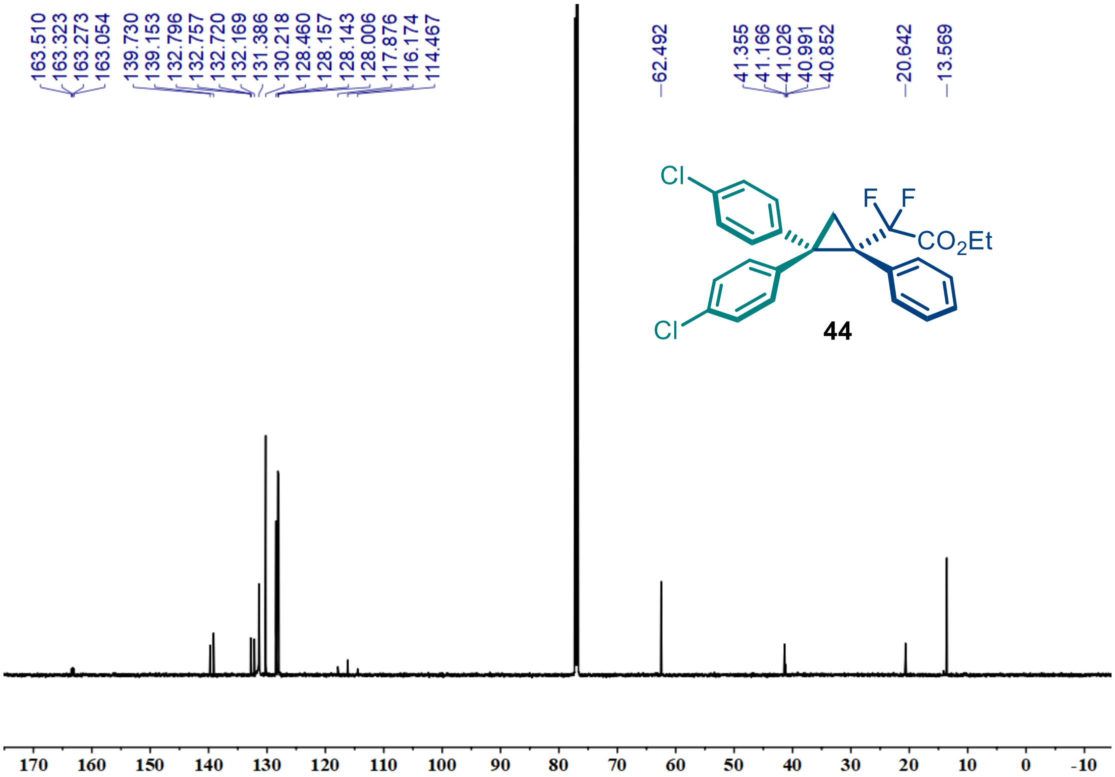

Figure S212, ^{13}C-NMR spectra copies of 44 related to scheme 2 and 3.

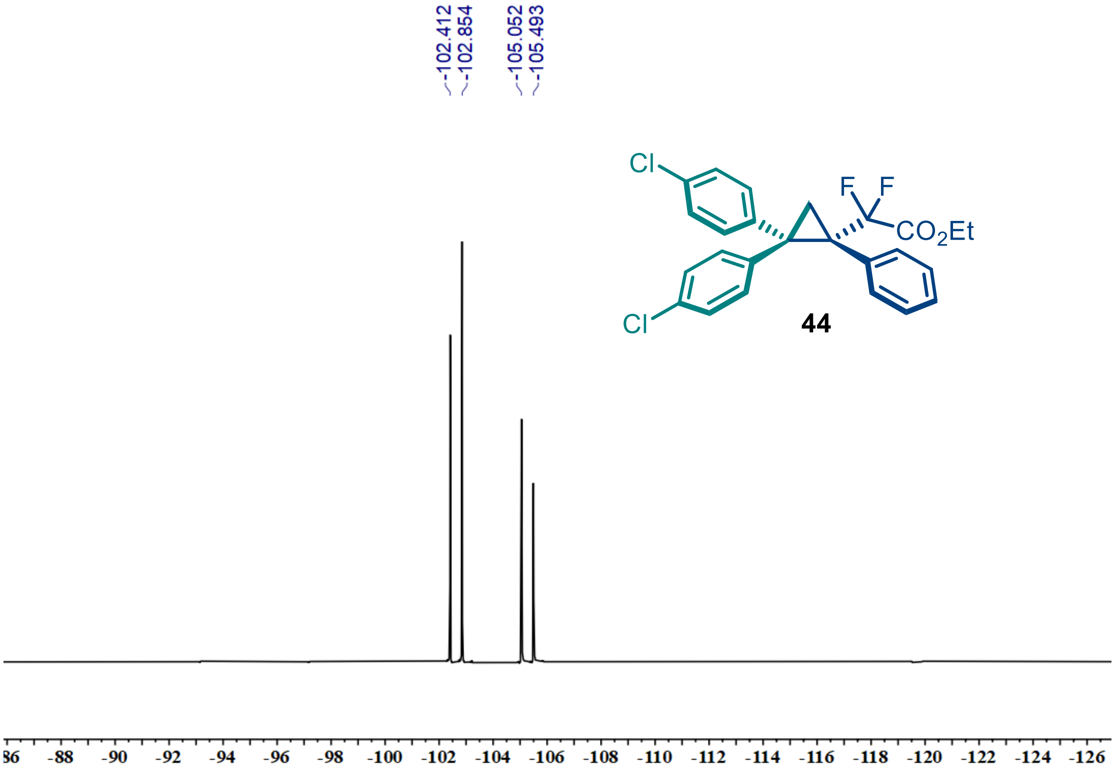

Figure S213, ^{19}F-NMR spectra copies of 44 related to scheme 2 and 3

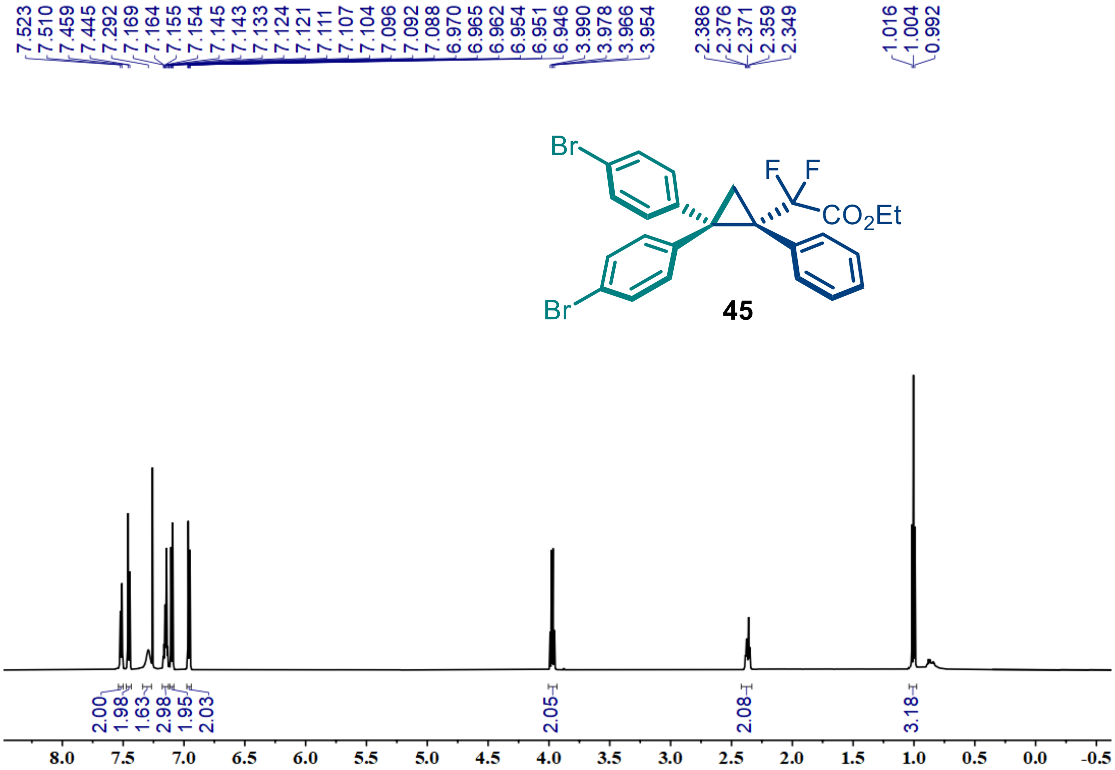

Figure S214, ^{1}H-NMR spectra copies of 45 related to scheme 2 and 3.

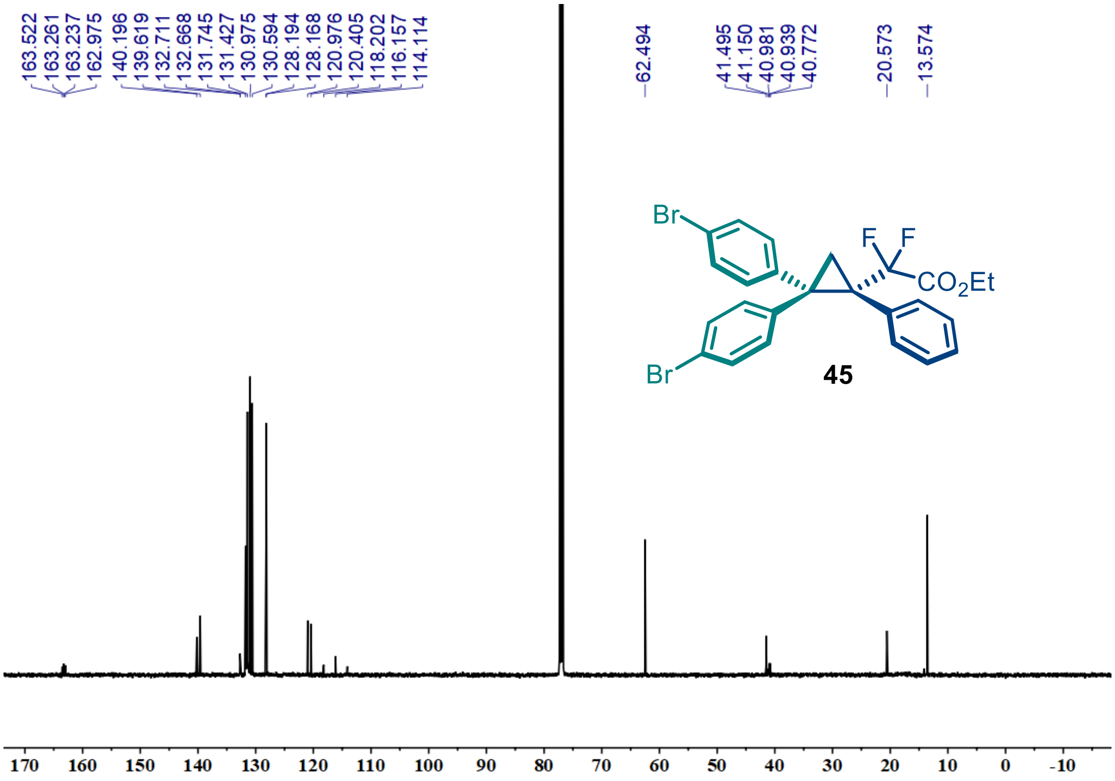

Figure S215, ^{13}C-NMR spectra copies of 45 related to scheme 2 and 3.

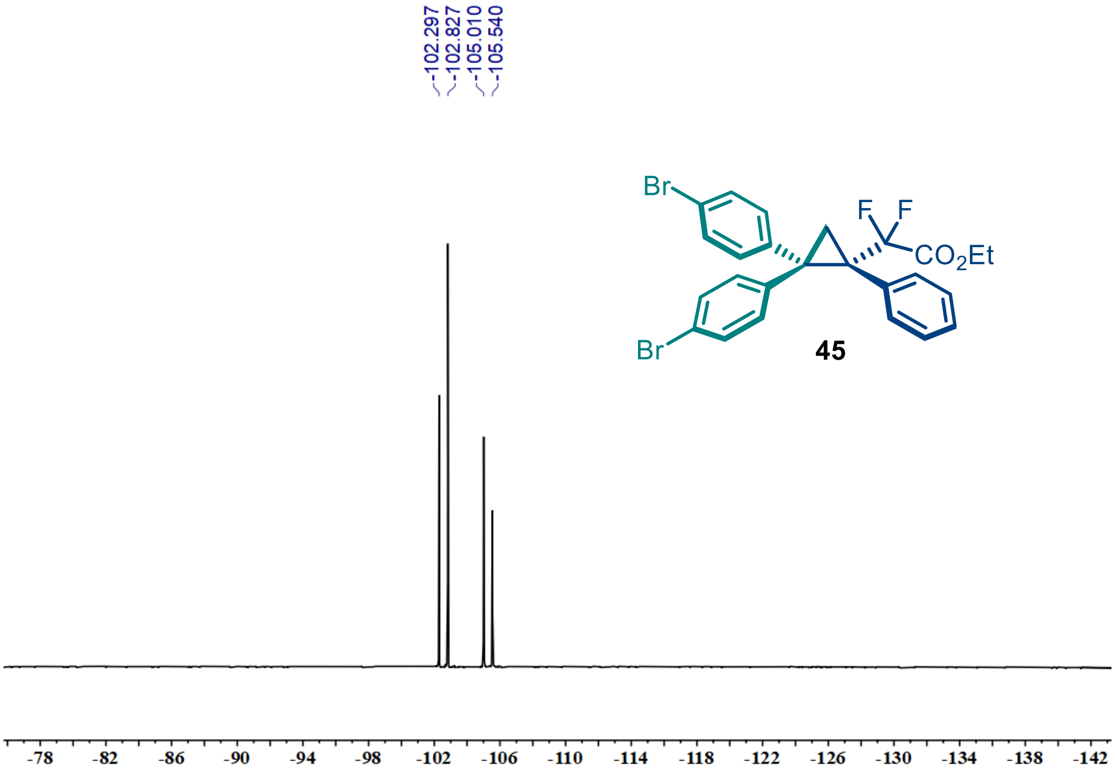

Figure S216, ^{19}F-NMR spectra copies of 45 related to scheme 2 and 3

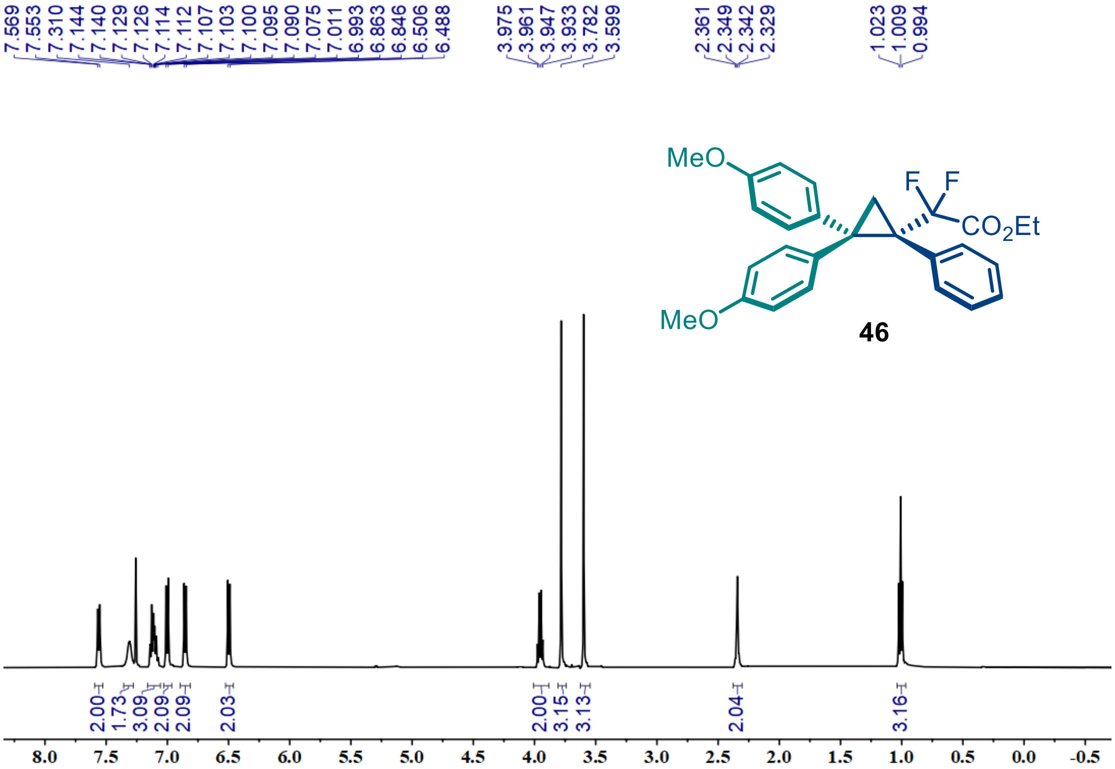

Figure S217, ^{1}H-NMR spectra copies of 46 related to scheme 2 and 3.

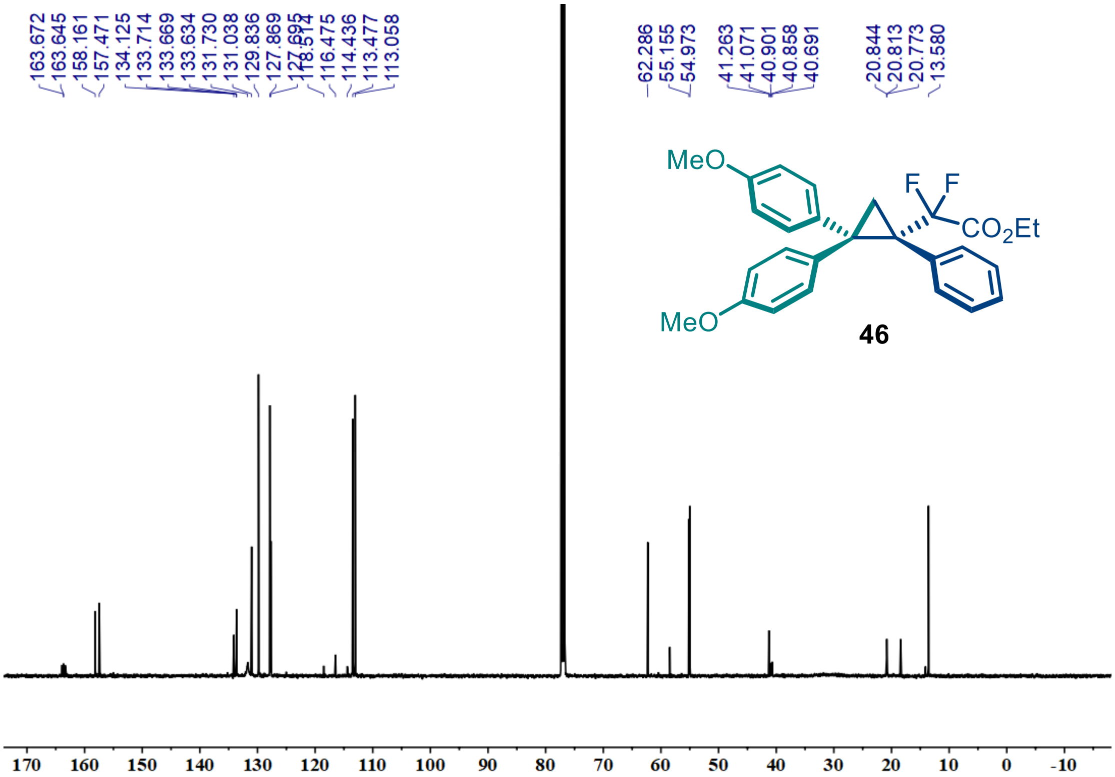

Figure S218, ^{13}C-NMR spectra copies of 46 related to scheme 2 and 3.

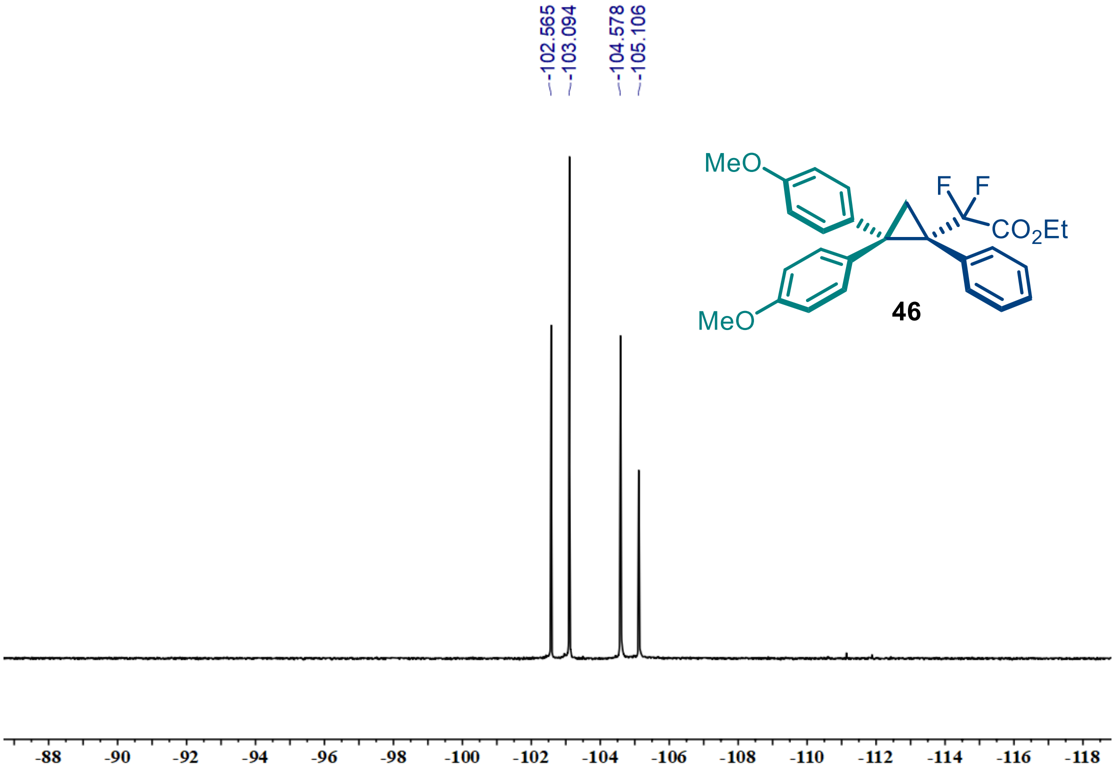

Figure S219, ^{19}F-NMR spectra copies of 46 related to scheme 2 and 3

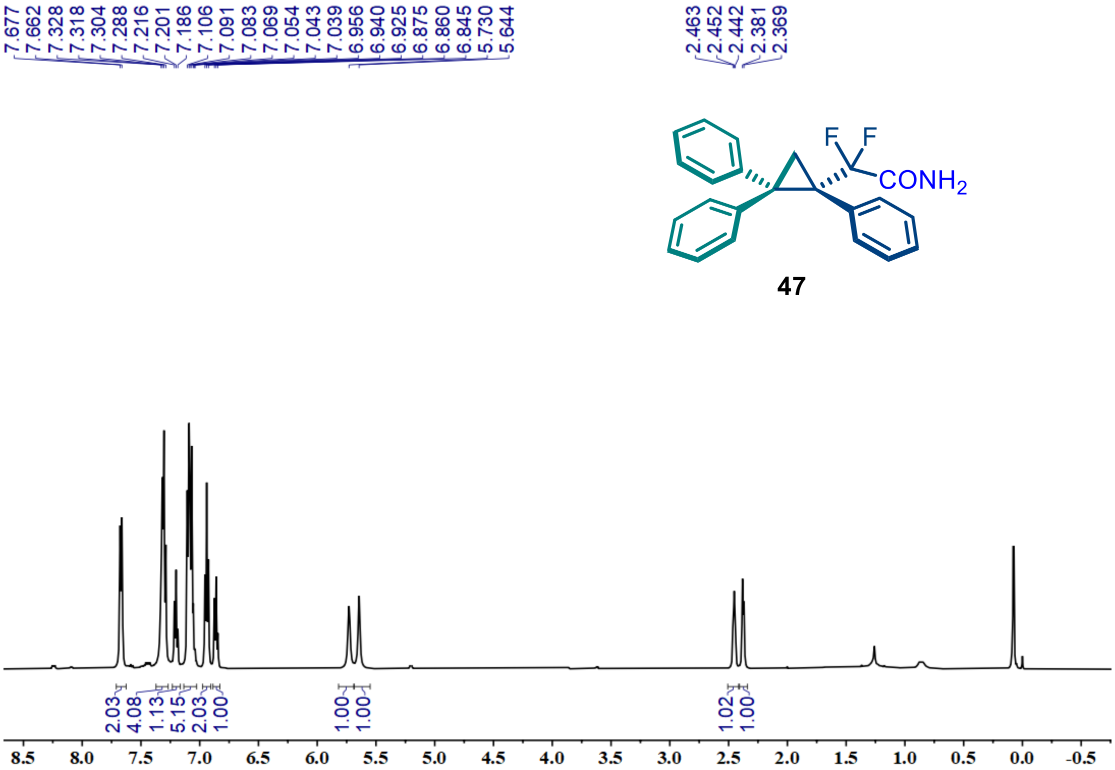

Figure S220, ^{1}H-NMR spectra copies of 47 related to scheme 2 and 3.

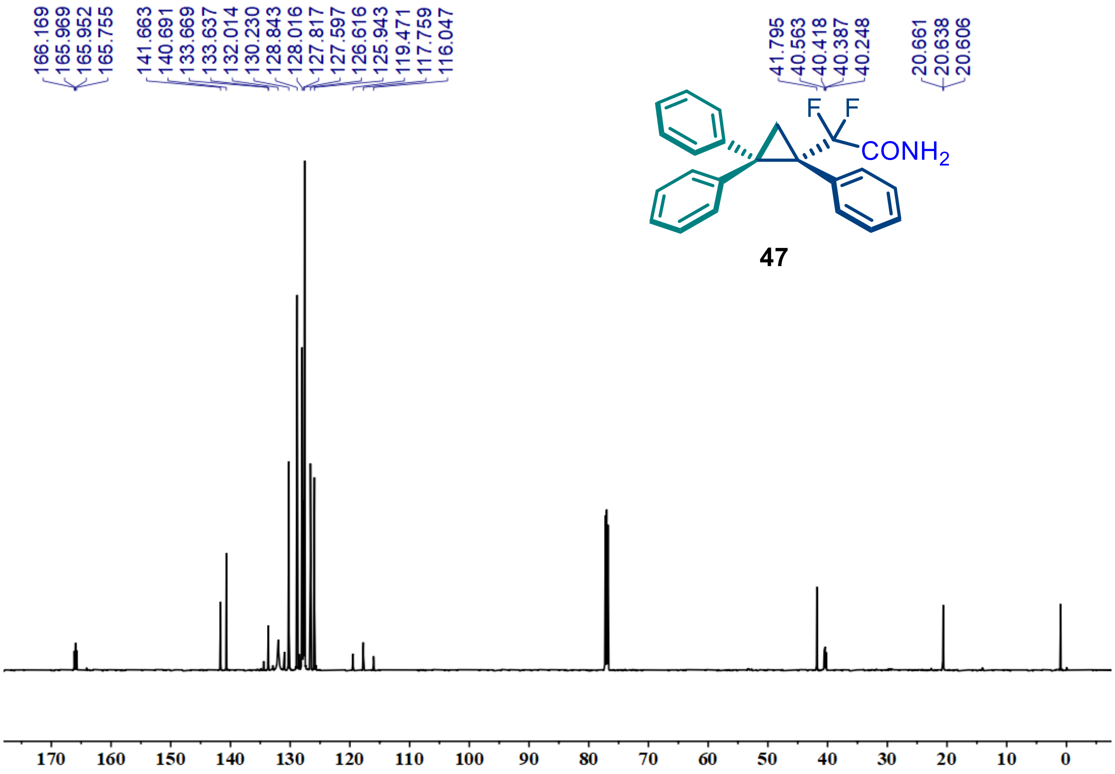

Figure S221, ^{13}C-NMR spectra copies of 47 related to scheme 2 and 3.

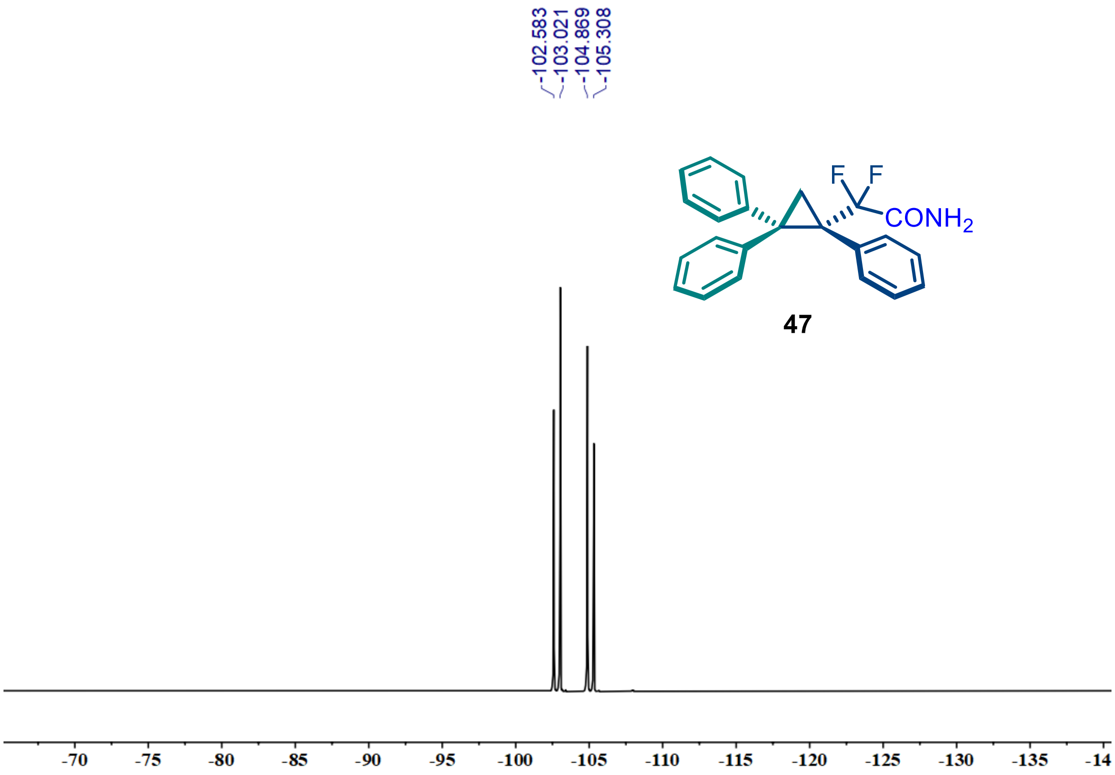

Figure S222, ^{19}F-NMR spectra copies of 47 related to scheme 2 and 3

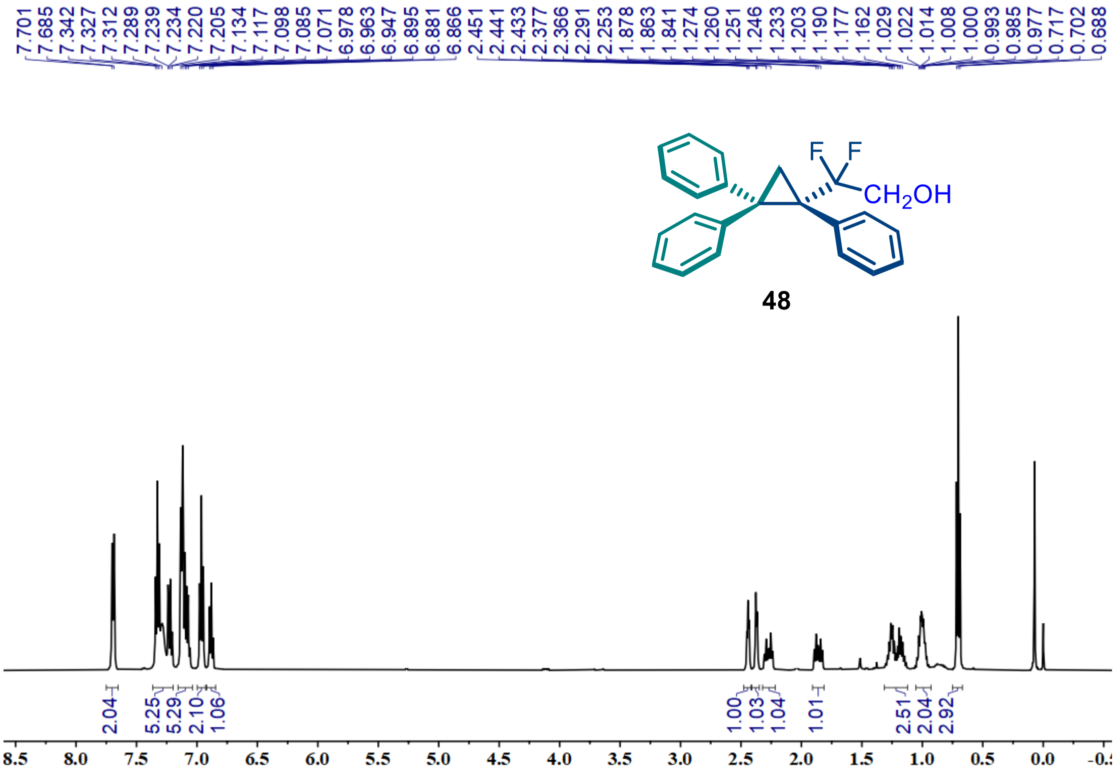

Figure S223, ^{1}H-NMR spectra copies of 48 related to scheme 2 and 3.

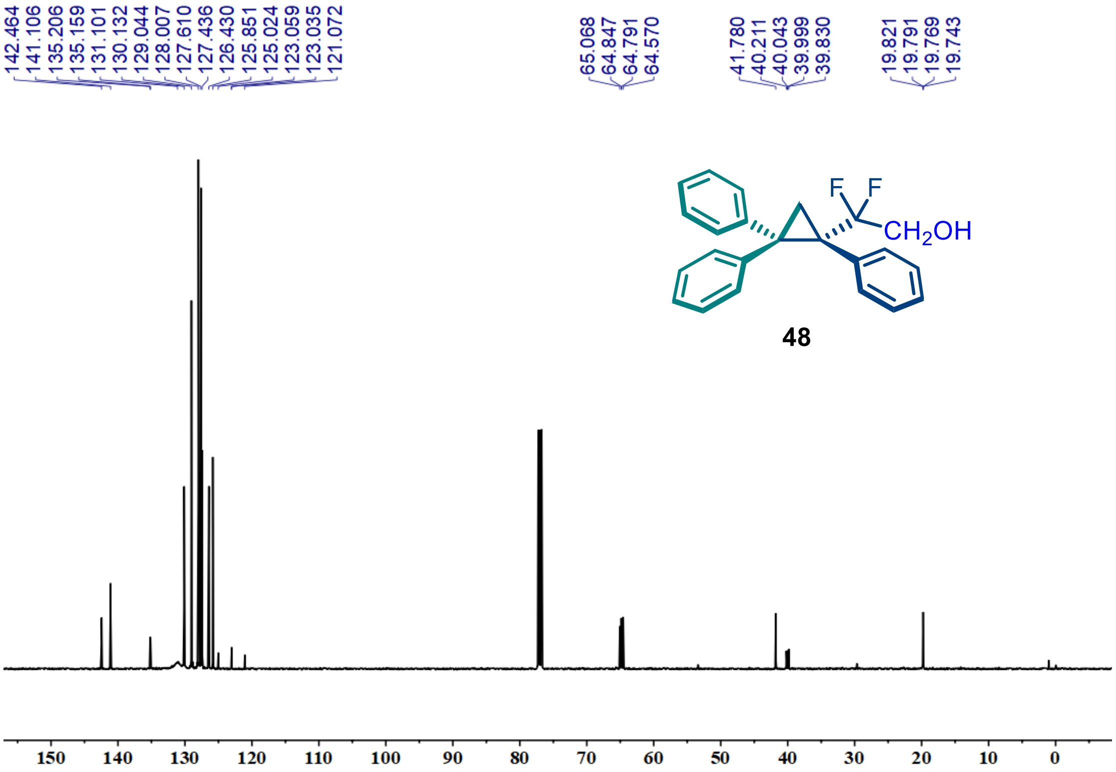

Figure S224, ^{13}C-NMR spectra copies of 48 related to scheme 2 and 3.

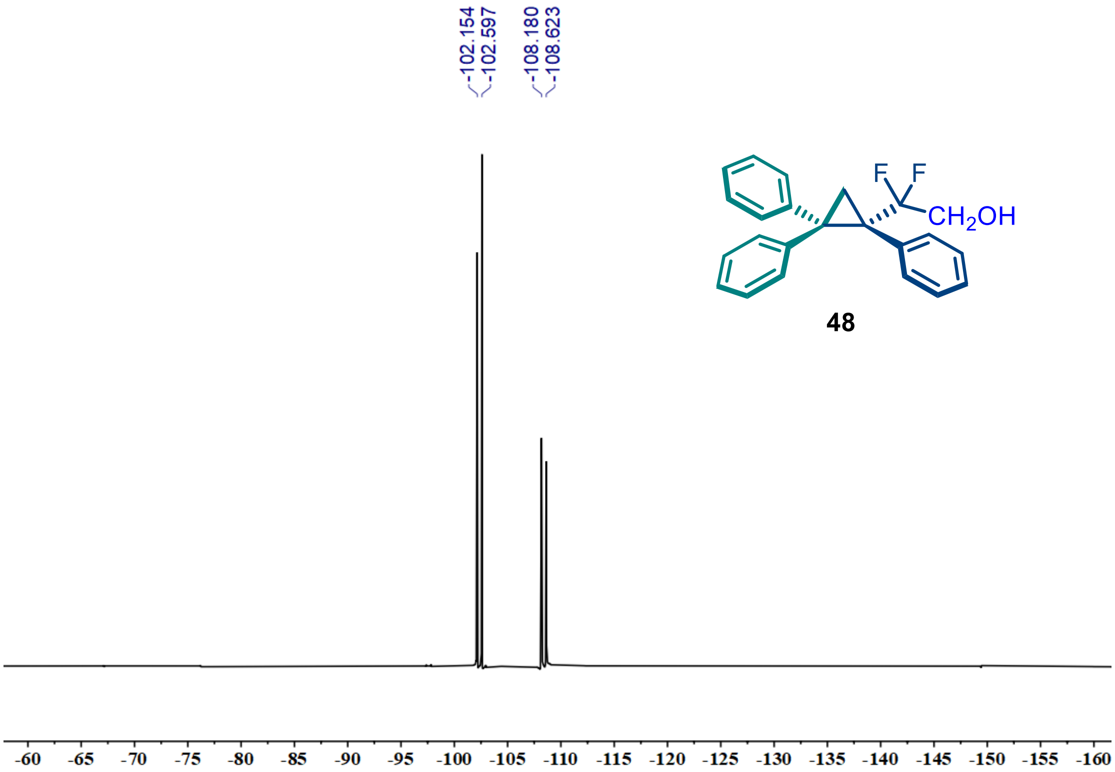

Figure S225, ^{19}F-NMR spectra copies of 48 related to scheme 2 and 3

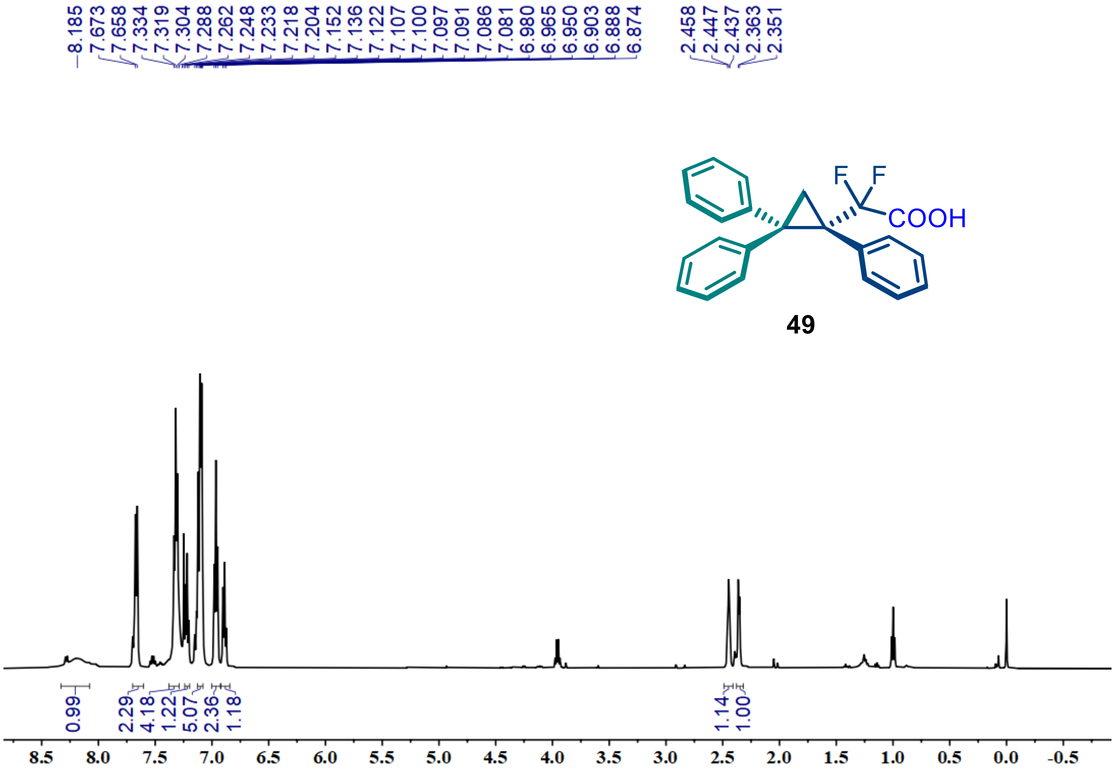

Figure S226, ^{1}H-NMR spectra copies of 49 related to scheme 2 and 3.

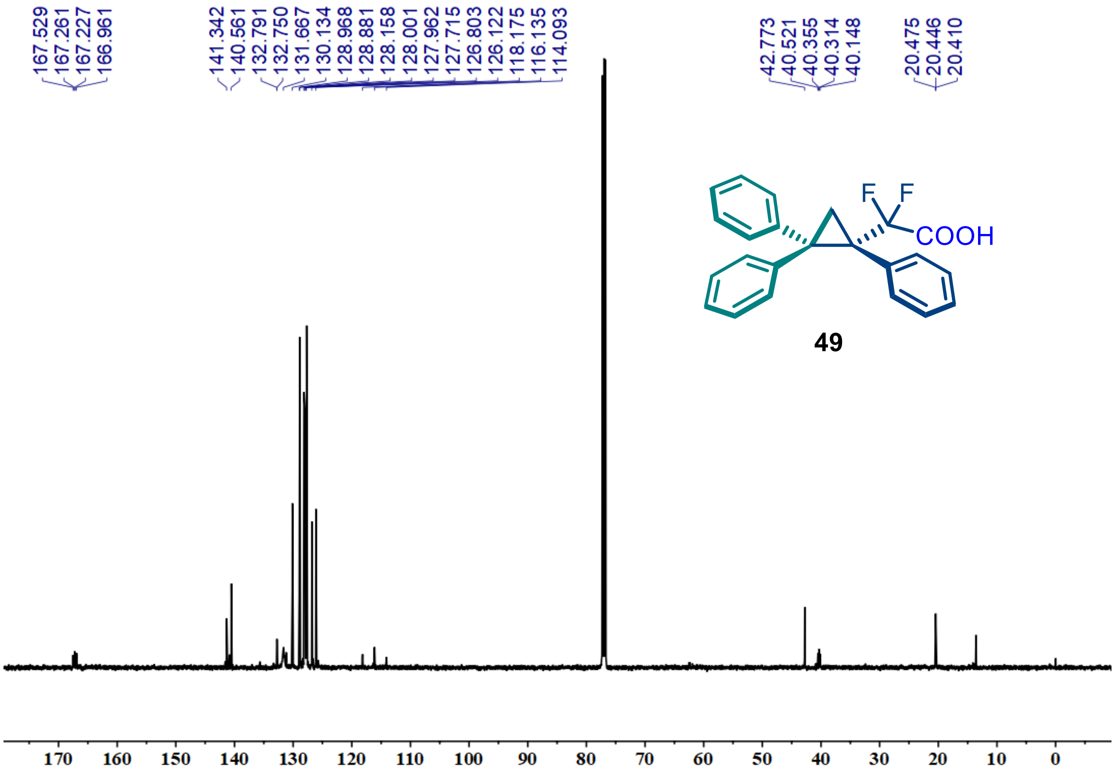

Figure S227, ^{13}C-NMR spectra copies of 49 related to scheme 2 and 3.

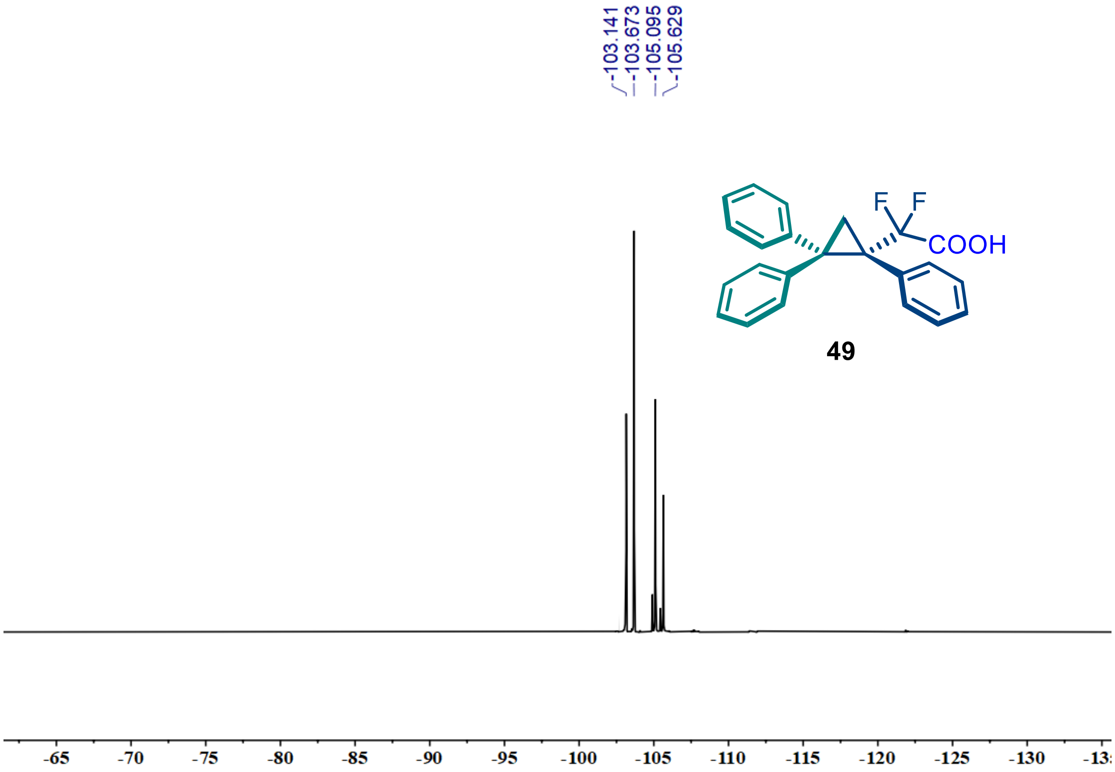

Figure S228, ^{19}F-NMR spectra copies of 49 related to scheme 2 and 3

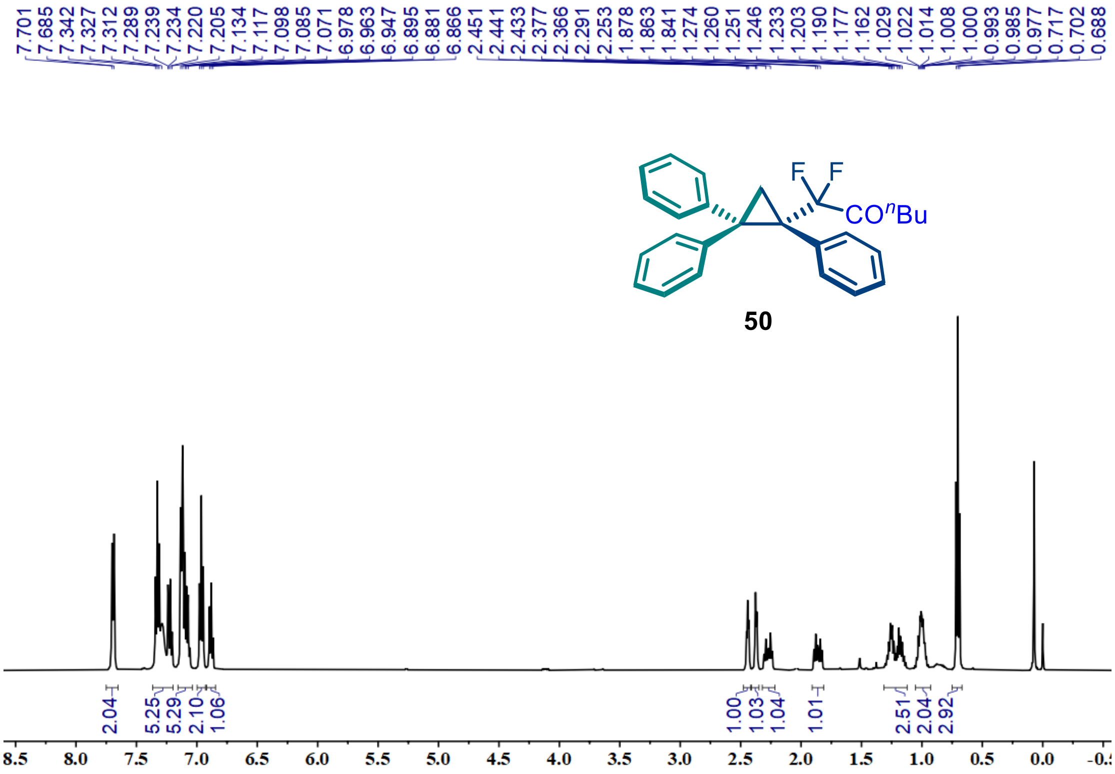

Figure S229, ^{1}H-NMR spectra copies of 50 related to scheme 2 and 3.

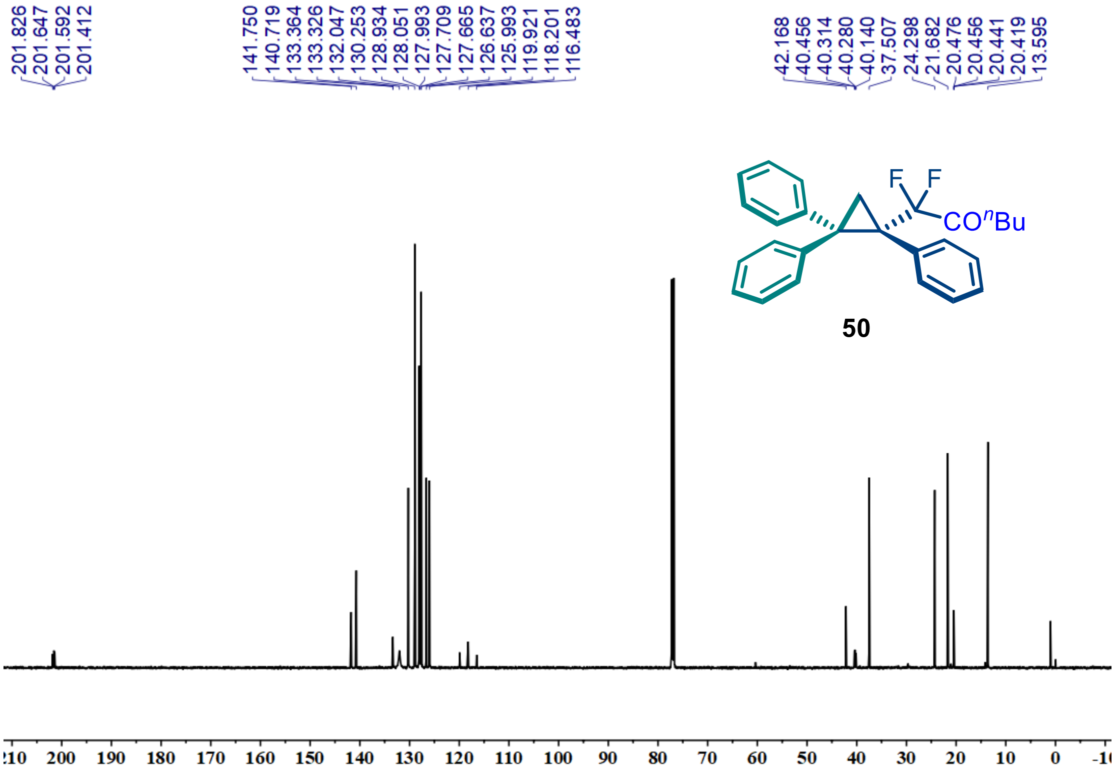

Figure S230, ^{13}C-NMR spectra copies of 50 related to scheme 2 and 3.

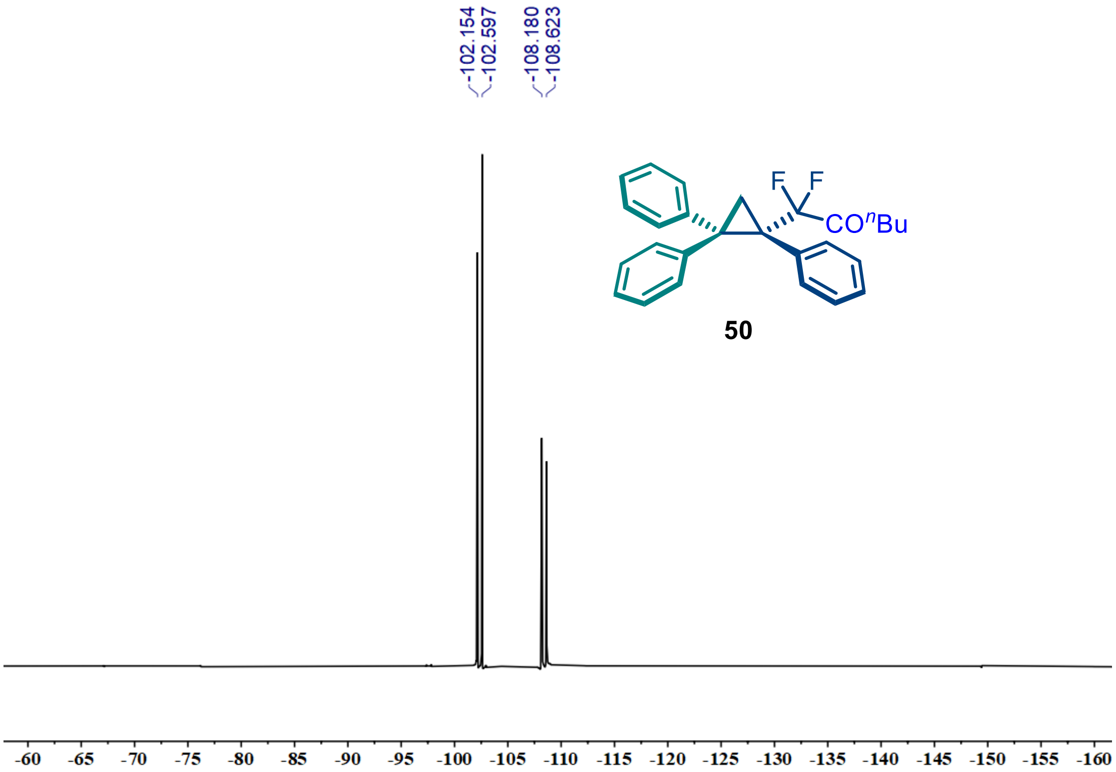

50

Figure S231, ^{19}F-NMR spectra copies of 50 related to scheme 2 and 3

Data S1, Computational studies related to scheme 4.

***Si*-face attack:**

Si-TS1-int

Zero-point correction=	1.888592 (Hartree/Particle)
Thermal correction to Energy=	2.000151
Thermal correction to Enthalpy=	2.001095
Thermal correction to Gibbs Free Energy=	1.735920
Sum of electronic and zero-point Energies=	-6973.667147
Sum of electronic and thermal Energies=	-6973.555587
Sum of electronic and thermal Enthalpies=	-6973.554643
Sum of electronic and thermal Free Energies=	-6973.819819

Rh	-0.28286300	-0.02040100	-0.26997600
Rh	-1.60220600	0.38014200	1.77325800
O	-1.78603000	-1.36239700	-0.78558400
O	-3.05202900	-0.89290000	1.03658700
O	-3.85182900	-3.73992700	-3.25520500
O	-1.93553500	-3.91579400	0.90834600
O	0.66019000	-1.56096000	0.77792600

O	-0.62430200	-1.24596200	2.61981900
O	1.91521200	-5.43991600	0.96647700
O	2.91290200	-1.29549600	2.67727800
O	1.02411200	1.42841700	0.47540800
O	-0.14575200	1.71498700	2.39210000
O	4.13390500	4.22390900	0.61514800
O	-0.43282700	4.04965500	0.12369600
O	-1.40803500	1.49346700	-1.11321400
O	-2.57929800	1.91608100	0.78627300
O	-2.30854800	4.16768800	-3.86025800
O	-4.12781100	0.47894600	-1.82906100
N	-3.19696400	-3.64891400	-1.02220400
N	2.12389000	-3.37722300	2.02671200
N	1.81399600	4.00959100	0.69672500
N	-3.34336500	2.54258400	-2.55307500
C	-2.82879000	-1.47410700	-0.05699100
C	-3.88098400	-2.41072400	-0.65609700
H	-4.15137200	-1.95010400	-1.61819400
C	-3.20109400	-4.17524100	-2.32633500
C	-2.25676400	-5.32117900	-2.29360800
C	-1.69271700	-5.38612700	-1.01932100
C	-2.24443600	-4.27557200	-0.21449100
C	0.22093300	-1.87915900	1.93300300
C	0.73766100	-3.22627800	2.45061700
H	0.19017200	-3.96779500	1.84531200
C	2.57284800	-4.45697200	1.25967100
C	3.97249700	-4.13607600	0.89630000
C	4.28013200	-2.87225600	1.39966600
C	3.08543300	-2.36029200	2.11643600
C	0.76734300	2.01762400	1.57871400
C	1.71862300	3.18250400	1.89854400
H	2.72355200	2.73885900	1.98964200
C	3.04138400	4.38731500	0.11209500
C	2.68794700	4.96705800	-1.20583100
C	1.30498200	4.88874700	-1.36704700
C	0.73091000	4.28736200	-0.14633100
C	-2.30155300	2.08935600	-0.43092500
C	-3.05983200	3.13851300	-1.24885300
H	-2.31112800	3.91612800	-1.46367800
C	-2.85729800	3.08722000	-3.75253800
C	-3.13830700	2.07336700	-4.79694400

C	-3.68127400	0.94829800	-4.17777300
C	-3.77508200	1.22149000	-2.72785400
C	-5.18971500	-2.63651200	0.12783200
C	-5.93934500	-1.29698400	0.27513400
C	-6.08623300	-3.58085400	-0.70013100
C	-4.97921400	-3.27157600	1.51283700
H	-6.09811900	-0.85896700	-0.72226600
H	-5.32992500	-0.58042100	0.83680600
C	-7.28076200	-1.51052100	0.97951300
H	-5.58377200	-4.55185300	-0.83180900
H	-6.23204100	-3.16272000	-1.70886000
C	-7.43477200	-3.79616600	-0.00824500
H	-4.31685000	-2.64452900	2.12275300
H	-4.47746600	-4.24482900	1.40170100
C	-6.32896800	-3.47133400	2.20990400
H	-7.78286200	-0.53738800	1.08742600
C	-8.15290400	-2.45386700	0.14879500
C	-7.03690600	-2.12185700	2.36266600
H	-8.04602200	-4.47634200	-0.61964300
C	-7.20220700	-4.41131600	1.37452600
H	-6.15625900	-3.91236100	3.20245800
H	-8.35215800	-2.01228500	-0.83955600
H	-9.12879900	-2.60272100	0.63597100
H	-6.42432700	-1.44124100	2.97289700
H	-7.99284400	-2.25509400	2.89216900
H	-8.16607400	-4.58380700	1.87753400
H	-6.71341700	-5.39290000	1.27537600
C	-4.26929400	3.83982300	-0.60168500
C	-3.79471400	4.65717600	0.61742100
C	-4.85470700	4.83310800	-1.62669500
C	-5.38874400	2.87474000	-0.17645600
H	-3.00655400	5.35763800	0.29760900
H	-3.34088500	3.99650200	1.36423300
C	-4.96624100	5.42561400	1.23260400
H	-5.20209900	4.28682200	-2.51716900
H	-4.06463000	5.52100200	-1.96801900
C	-6.02259900	5.61492300	-1.01998000
H	-4.99931700	2.13561700	0.53563700
H	-5.74559400	2.31098200	-1.05136200
C	-6.55150100	3.65704800	0.44230900
H	-4.60572000	5.98308900	2.10957000

C	-5.53849200	6.40015600	0.20105200
C	-6.05442000	4.43705600	1.66278700
H	-6.42008500	6.30899800	-1.77523000
C	-7.11845900	4.63566900	-0.59025300
H	-7.33476100	2.94807200	0.74977500
H	-4.76969600	7.12935700	-0.09696100
H	-6.37190300	6.97297400	0.63616100
H	-5.65546500	3.74236600	2.41728700
H	-6.89079100	4.97652800	2.13349300
H	-7.97112200	5.18687100	-0.16471500
H	-7.49824800	4.08448100	-1.46437900
C	1.46844400	3.97758300	3.19545600
C	1.64587700	3.04309000	4.40910800
C	2.54890900	5.07437400	3.30122200
C	0.08838000	4.65419700	3.24937300
H	2.65211300	2.59162200	4.37019500
H	0.92092800	2.22204100	4.37055400
C	1.48190800	3.82500900	5.71461000
H	2.46575000	5.76189700	2.44594200
H	3.54891500	4.61615600	3.23850200
C	2.39779200	5.86162000	4.60481500
H	-0.70868000	3.90712400	3.14527300
H	-0.01592600	5.34456000	2.39854700
C	-0.06348400	5.42605300	4.56436900
H	1.59083500	3.13215800	6.56155200
C	2.55259500	4.91476300	5.79648600
C	0.09074900	4.46558500	5.74742000
H	3.17508400	6.63870400	4.64393400
C	1.01238200	6.51219500	4.64869300
H	-1.06052200	5.88958100	4.59300200
H	3.55515600	4.46024000	5.79220100
H	2.45690000	5.47396800	6.73984800
H	-0.68478300	3.68586100	5.70434700
H	-0.05219000	5.00783400	6.69470500
H	0.89664600	7.09167700	5.57756800
H	0.89964200	7.22082600	3.81378400
C	0.48255800	-3.59304200	3.93108700
C	-1.03211900	-3.78444500	4.15882800
C	1.16299600	-4.94735700	4.22123800
C	1.03177200	-2.55827400	4.92663400
H	-1.40856100	-4.54335800	3.45520200

H	-1.57012200	-2.85686300	3.93528600
C	-1.30649900	-4.21917100	5.60006100
H	2.24979500	-4.86010400	4.06861300
H	0.80271300	-5.70310700	3.50490600
C	0.89019100	-5.39775000	5.65884400
H	0.58536000	-1.57451800	4.73232500
H	2.11617800	-2.44571600	4.78558500
C	0.74288700	-3.00382900	6.36375700
H	-2.39225400	-4.32713300	5.73838400
C	-0.61655300	-5.55688300	5.87227700
C	-0.76733800	-3.15792000	6.56358200
H	1.39515000	-6.35935400	5.83374000
C	1.43079800	-4.34527900	6.63078000
H	1.13226600	-2.24498800	7.05795200
H	-1.01559000	-6.33147700	5.19952300
H	-0.81908900	-5.89089300	6.90154700
H	-1.27159300	-2.19616800	6.38560300
H	-0.98348000	-3.44597700	7.60395600
H	1.25313600	-4.66472000	7.66916100
H	2.52018100	-4.24166200	6.51030500
C	3.50500000	5.45343700	-2.21223100
C	2.88799400	5.86189800	-3.39773800
C	1.50215600	5.77506000	-3.55963100
C	0.68245400	5.28489100	-2.53954300
C	-2.88663200	2.09614100	-6.15778800
C	-3.19958100	0.94600900	-6.88835500
C	-3.73437000	-0.18389300	-6.26384400
C	-3.98231700	-0.20124300	-4.88810800
C	-1.89874100	-6.22117800	-3.28284600
C	-0.95833400	-7.20194400	-2.95090600
C	-0.39938300	-7.26663000	-1.67169300
C	-0.75820500	-6.34931800	-0.67861200
C	4.89622000	-4.87785100	0.18019900
C	6.15748300	-4.31299100	-0.02532700
C	6.46948800	-3.05117500	0.48666600
C	5.53240000	-2.30802000	1.21115500
H	4.63973800	-5.86348800	-0.20461900
H	6.91192200	-4.86443500	-0.58348400
H	7.46598200	-2.64243200	0.32501300
H	5.77316100	-1.32566300	1.61450900
H	-0.30381800	-6.36888100	0.30998800

H	0.33397700	-8.03862100	-1.44673900
H	-0.65399400	-7.92820900	-3.70215200
H	-2.33564400	-6.16193800	-4.27763400
H	-2.45406400	2.97492700	-6.63181900
H	-3.01695600	0.92538700	-7.96116200
H	-3.95553800	-1.06785900	-6.85871000
H	-4.37633600	-1.08600600	-4.39058700
H	-0.39422000	5.18737500	-2.67337900
H	1.05364400	6.08512900	-4.50126600
H	3.49646700	6.24363600	-4.21541300
H	4.58463600	5.49587400	-2.08220800
F	-0.75048900	0.36984300	-3.49627800
F	0.70836800	1.84852500	-2.73424900
O	2.30723200	1.06180300	-4.72187100
O	1.04780400	-0.80273600	-4.96130700
C	1.41295500	0.32838300	-4.36248900
C	5.63470000	0.56544700	-2.42754400
C	6.56711100	-0.42130300	-2.07326800
H	6.71108100	-0.65805000	-1.02088700
C	6.16393000	0.13704100	-4.76658300
H	6.01844400	0.35665900	-5.82144100
C	0.55778200	0.55312600	-3.10095100
C	7.07119100	-0.84698600	-4.37738400
C	1.52432200	-1.58721500	-2.10388300
C	5.46118800	0.83719900	-3.79531000
H	4.75693000	1.60509900	-4.10815600
C	0.74050100	-0.39239900	-1.91989900
C	4.78808800	1.19958200	-1.39696700
C	3.56793600	1.68543900	-1.69844200
H	3.16998600	1.67886300	-2.71001400
H	2.91690300	2.09127900	-0.92861600
C	1.01212500	-2.84367400	-1.70576000
H	0.01921000	-2.88426600	-1.26332000
C	7.28320800	-1.12907700	-3.03163800
H	7.99208600	-1.90120300	-2.74056400
C	1.73408000	-4.00501600	-1.94234600
H	1.30789700	-4.96691300	-1.66599200
C	2.81822000	-1.54802900	-2.67900300
H	3.25436600	-0.59309400	-2.96172300
C	3.01429000	-3.94017000	-2.49259400
H	3.58641300	-4.85363600	-2.64784700

C	3.56536500	-2.70387600	-2.83565600
H	4.57436900	-2.63736800	-3.24236800
C	1.91874200	-1.24869300	-6.02716500
H	1.77831900	-0.58262900	-6.88681100
H	2.95662800	-1.13777200	-5.68519000
C	1.55493700	-2.67671100	-6.32105800
H	0.50996000	-2.76120500	-6.63647600
H	1.69580100	-3.29957000	-5.43058400
H	2.18972400	-3.07031400	-7.12171800
Cl	7.95273400	-1.73470600	-5.59347700
C	5.26011100	1.25616800	0.00263100
C	4.38465400	0.96038600	1.05320900
C	6.55845900	1.68182300	0.31835400
C	4.76685500	1.12978600	2.38004700
H	3.38705100	0.58034400	0.82737300
C	6.95363200	1.86822700	1.63716200
H	7.25915000	1.90864900	-0.48482000
C	6.04329000	1.60552200	2.65880200
H	4.08482100	0.86820300	3.18424100
H	7.95021600	2.23060400	1.87569000
Cl	6.51586900	1.86342400	4.31949300

Si-TS1

Zero-point correction= 1.889222 (Hartree/Particle)
Thermal correction to Energy= 1.999224
Thermal correction to Enthalpy= 2.000168
Thermal correction to Gibbs Free Energy= 1.741147
Sum of electronic and zero-point Energies= -6973.649470
Sum of electronic and thermal Energies= -6973.539469
Sum of electronic and thermal Enthalpies= -6973.538524
Sum of electronic and thermal Free Energies= -6973.797546

Rh	0.01703100	0.07926200	0.04167900
Rh	1.72439500	0.58840600	-1.67752400
O	1.17996200	-1.61245300	0.41136200
O	2.86050200	-0.98327700	-0.97464700
O	2.53980100	-4.35950900	2.94836700
O	1.41999300	-4.00657100	-1.48600300
O	-0.97828100	-1.03455000	-1.44169100
O	0.72697400	-0.75438400	-2.91166200

O	-2.25179000	-4.77314200	-2.08141700
O	-2.90900200	-0.53307100	-3.71133100
O	-0.83970400	1.88505200	-0.50659000
O	0.57584800	2.23691700	-2.23102200
O	-3.69511700	4.66441300	-0.68514200
O	0.64563200	4.15117000	0.72976000
O	1.23883600	1.18465900	1.29249500
O	2.70453200	1.81317800	-0.32014600
O	1.86149700	3.26806600	4.54395700
O	3.67458400	-0.27045100	2.25965800
N	2.32372300	-4.01800600	0.65321800
N	-2.26729800	-2.66007100	-3.04988200
N	-1.40943600	4.42857300	-0.31667900
N	2.97033800	1.74224600	3.17936600
C	2.34586400	-1.71816400	-0.08735800
C	3.17718900	-2.84046600	0.53916900
H	3.33064100	-2.51404500	1.57986300
C	2.05262500	-4.65609000	1.87497600
C	1.08005300	-5.73007400	1.55300200
C	0.78079000	-5.65639900	0.19293600
C	1.50837000	-4.49927700	-0.37453000
C	-0.32503000	-1.31626100	-2.50291400
C	-0.83192000	-2.54093900	-3.27602600
H	-0.41204000	-3.39217800	-2.71346000
C	-2.82342900	-3.72886200	-2.33698500
C	-4.19922800	-3.30462200	-1.98034800
C	-4.40295000	-2.01894600	-2.47990600
C	-3.15992700	-1.58828600	-3.16250300
C	-0.35690800	2.58413400	-1.45986100
C	-1.05405500	3.94115000	-1.64840000
H	-2.03518900	3.71669200	-2.10221900
C	-2.75115000	4.59163000	0.07655700
C	-2.73607100	4.62280100	1.55753600
C	-1.41783100	4.46624600	1.98710000
C	-0.55543000	4.33384100	0.79370500
C	2.27396800	1.78315900	0.86694600
C	3.00037700	2.56359100	1.96979700
H	2.32470800	3.40049400	2.20446900
C	2.30794300	2.15337000	4.34654700
C	2.25541900	0.96002500	5.22387100
C	2.81503000	-0.11352200	4.53412700

C	3.22897500	0.36594200	3.19698900
C	4.57235400	-3.14439700	-0.05103300
C	5.48804000	-1.91829000	0.14258300
C	5.19156900	-4.31107500	0.74741900
C	4.54635500	-3.54394200	-1.53635900
H	5.51456200	-1.65350100	1.20998700
H	5.07567200	-1.05095100	-0.38402000
C	6.90049000	-2.21685300	-0.36233000
H	4.56771900	-5.21212100	0.63828800
H	5.20059700	-4.06112600	1.82050300
C	6.60954200	-4.61549300	0.25633800
H	4.08259000	-2.74893300	-2.13424400
H	3.92489100	-4.44157000	-1.67038600
C	5.96828700	-3.83205900	-2.02941300
H	7.52172000	-1.31839000	-0.22837300
C	7.49446600	-3.37977900	0.43470300
C	6.84257900	-2.58822100	-1.84678400
H	7.01813400	-5.45361600	0.84023400
C	6.56169700	-4.99321400	-1.22674100
H	5.92829700	-4.10244500	-3.09480400
H	7.56185300	-3.11199700	1.50021200
H	8.51840300	-3.59665900	0.09317200
H	6.43183900	-1.74980900	-2.42920200
H	7.85662100	-2.77973900	-2.23054500
H	7.57437300	-5.22879900	-1.58861600
H	5.95294200	-5.89979800	-1.36778300
C	4.37930000	3.17316400	1.64947400
C	4.20540800	4.27063800	0.57876500
C	4.92020800	3.85007600	2.92550500
C	5.42113300	2.14735600	1.17259000
H	3.46671300	5.00789500	0.93284000
H	3.80103100	3.83655100	-0.34239100
C	5.54241400	4.95733400	0.29325500
H	5.05468000	3.09636100	3.71647500
H	4.18032400	4.57408000	3.30280000
C	6.25664000	4.54344700	2.64784000
H	5.05414700	1.62553300	0.27948900
H	5.56889300	1.37957700	1.94610800
C	6.75255800	2.84651200	0.87962600
H	5.39242900	5.72245600	-0.48287100
C	6.06637100	5.61472300	1.57156800

C	6.55084000	3.91512000	-0.19889300
H	6.61858400	5.00934700	3.57636600
C	7.27378600	3.50863700	2.15813300
H	7.47856700	2.09836000	0.52774800
H	5.35757200	6.38121100	1.92045700
H	7.02104900	6.12666900	1.37505300
H	6.18857000	3.44982600	-1.12803400
H	7.51106700	4.39920600	-0.43534500
H	8.24352800	3.99411600	1.96830100
H	7.44397400	2.74750000	2.93516300
C	-0.38118100	4.98086000	-2.56599800
C	-0.41226300	4.46310400	-4.01847700
C	-1.21517200	6.27698600	-2.51718900
C	1.06488000	5.32036500	-2.16920800
H	-1.45609500	4.23895900	-4.29887900
H	0.15127300	3.52671700	-4.09924900
C	0.17064300	5.50796700	-4.97277600
H	-1.22326300	6.67251800	-1.49040400
H	-2.26271300	6.05354500	-2.77688500
C	-0.63775700	7.32764000	-3.46893600
H	1.67975700	4.41071200	-2.16424800
H	1.08535600	5.71600200	-1.14417100
C	1.64330900	6.35787500	-3.13640200
H	0.14959900	5.10769800	-5.99678600
C	-0.66108800	6.79013700	-4.90148800
C	1.61630800	5.80927800	-4.56570100
H	-1.24650100	8.24147900	-3.40472500
C	0.80722100	7.63908100	-3.06799600
H	2.68095700	6.57678800	-2.84354300
H	-1.69678800	6.58701000	-5.21382400
H	-0.25901700	7.54436400	-5.59519000
H	2.22518600	4.89495700	-4.63115800
H	2.05916900	6.53998400	-5.25978500
H	1.22716300	8.40470500	-3.73834000
H	0.83686100	8.05493400	-2.04924100
C	-0.37151000	-2.73770200	-4.74031300
C	1.12422100	-3.12212200	-4.74521000
C	-1.15238800	-3.92595100	-5.33776600
C	-0.58998400	-1.51364500	-5.64440100
H	1.26897100	-4.01076800	-4.11169100
H	1.72179300	-2.31952100	-4.29780300

C	1.60198100	-3.40904600	-6.16997800
H	-2.22829100	-3.69413700	-5.35264600
H	-1.02788600	-4.81110300	-4.69271700
C	-0.67850600	-4.22807300	-6.76186100
H	-0.05645000	-0.64623700	-5.23410900
H	-1.65545300	-1.24839300	-5.66324200
C	-0.10267300	-1.81391700	-7.06575200
H	2.67219200	-3.66156600	-6.14275300
C	0.80966100	-4.58360700	-6.74755900
C	1.38655900	-2.16790100	-7.04037800
H	-1.25947200	-5.07307600	-7.16056000
C	-0.89514800	-2.99198200	-7.63937300
H	-0.26047300	-0.92387000	-7.69229800
H	0.97837400	-5.48720400	-6.14185500
H	1.15272800	-4.81305500	-7.76824300
H	1.96869600	-1.32291400	-6.64279000
H	1.74892900	-2.35432000	-8.06327100
H	-0.57353300	-3.20148200	-8.67123300
H	-1.96620500	-2.74111400	-7.68284900
C	-3.78577600	4.68639400	2.45853600
C	-3.47044800	4.58777200	3.81720300
C	-2.14840800	4.42941300	4.24421300
C	-1.09208200	4.36869400	3.33005100
C	1.69397500	0.79654200	6.47934100
C	1.71378100	-0.48860100	7.02890300
C	2.27018900	-1.56480200	6.33095000
C	2.82963300	-1.39349100	5.06177400
C	0.49761600	-6.68625000	2.36711900
C	-0.38987100	-7.58633600	1.76898100
C	-0.67395500	-7.52182900	0.40209600
C	-0.09208300	-6.54512500	-0.41202000
C	-5.17701800	-3.96063800	-1.25174400
C	-6.37973500	-3.28428100	-1.03174100
C	-6.58857600	-2.00028500	-1.54434500
C	-5.59675500	-1.34469700	-2.27978500
H	-5.00160100	-4.95961300	-0.85760600
H	-7.16887700	-3.76345200	-0.45463300
H	-7.53986400	-1.50057000	-1.36391900
H	-5.74624800	-0.34027300	-2.67296300
H	-0.33608900	-6.45743600	-1.46822200
H	-1.36779400	-8.23842400	-0.03306500

H	-0.86600100	-8.35408300	2.37608900
H	0.72746900	-6.73028600	3.43006600
H	1.24435100	1.63674200	7.00466900
H	1.28006600	-0.65801900	8.01300800
H	2.25631900	-2.55637700	6.78025800
H	3.22634300	-2.23307400	4.49344700
H	-0.06238100	4.21780800	3.65356500
H	-1.93833700	4.34058700	5.30816300
H	-4.26826800	4.62431700	4.55729700
H	-4.81317600	4.78977500	2.11266600
F	0.55449600	-0.82393500	2.93731700
F	-0.45048200	1.12149100	3.21603600
O	-2.20069900	-0.08803100	4.86354300
O	-0.99954300	-1.97615900	4.49799600
C	-1.38173500	-0.73007400	4.23847900
C	-5.13474500	0.50439700	2.23567900
C	-6.17785400	-0.30001000	1.74098200
H	-6.21370600	-0.52020800	0.67500800
C	-6.02136900	0.18905800	4.47911600
H	-5.97649500	0.37918400	5.54806600
C	-0.66511900	-0.20437500	2.98106900
C	-7.02805300	-0.62263100	3.95658300
C	-2.06012100	-1.75374600	1.59348200
C	-5.08592500	0.74747200	3.62060100
H	-4.29960800	1.37545200	4.03402100
C	-1.31351300	-0.46497900	1.60301300
C	-4.09292700	0.98525100	1.32486400
C	-2.77199600	1.11286400	1.76257000
H	-2.64554900	1.10515800	2.84310300
H	-2.12013300	1.78485700	1.21278400
C	-1.61526000	-2.85411200	0.83949100
H	-0.75536200	-2.74274500	0.18627100
C	-7.11554000	-0.87057700	2.58771700
H	-7.90030200	-1.51498500	2.19837000
C	-2.23802000	-4.09546200	0.95676700
H	-1.86976500	-4.92735300	0.36505300
C	-3.17531100	-1.96224200	2.43098800
H	-3.54020100	-1.14578200	3.04361500
C	-3.33700400	-4.27435200	1.78882600
H	-3.81682200	-5.25019900	1.86111300
C	-3.81466900	-3.18997800	2.52109700

H	-4.68030300	-3.29438100	3.17580500
C	-1.73015300	-2.66910600	5.52888100
H	-1.45367900	-2.23685700	6.49904300
H	-2.80168800	-2.48379600	5.37409000
C	-1.36381500	-4.12233500	5.40326900
H	-0.28274900	-4.26261900	5.51442000
H	-1.65398200	-4.50419300	4.41725500
H	-1.87327000	-4.71425500	6.17105000
Cl	-8.19342700	-1.33855200	5.03092200
C	-4.49138300	1.39933200	-0.01346000
C	-3.62691200	1.26828300	-1.11373800
C	-5.72672100	2.05262800	-0.20994600
C	-3.93922500	1.84010000	-2.33864700
H	-2.71509300	0.68533600	-1.01238300
C	-6.04940500	2.62712300	-1.42763300
H	-6.41189500	2.16501100	0.62885400
C	-5.13470800	2.54038500	-2.47655400
H	-3.27153100	1.72201200	-3.18648500
H	-6.97856300	3.17496400	-1.55894200
Cl	-5.48835400	3.32881200	-3.98330400

Si-int1

Zero-point correction=	1.892062 (Hartree/Particle)
Thermal correction to Energy=	2.002270
Thermal correction to Enthalpy=	2.003215
Thermal correction to Gibbs Free Energy=	1.744801
Sum of electronic and zero-point Energies=	-6973.656264
Sum of electronic and thermal Energies=	-6973.546056
Sum of electronic and thermal Enthalpies=	-6973.545112
Sum of electronic and thermal Free Energies=	-6973.803525

Rh	0.20145300	0.20436600	0.47203600
Rh	0.37879800	0.30438200	-2.01327800
O	-0.70813700	-1.65894900	0.25327100
O	-0.51211500	-1.59116400	-2.00890300
O	-1.31689400	-5.61986300	1.20755100
O	-3.61771700	-2.06242000	-0.56041700
O	-1.81994300	0.96271900	0.02909200
O	-1.48675000	1.13155600	-2.19366300
O	-5.87297100	0.39754400	0.48148100

O	-3.47855300	3.93063100	-1.24610300
O	1.27031800	1.98684600	0.47430900
O	1.32198000	2.15439800	-1.79069100
O	3.41221400	4.88440900	2.15089000
O	4.18433300	1.43751700	-0.78076700
O	2.02544700	-0.76972900	0.27678900
O	2.20390900	-0.63818200	-1.96957900
O	5.63997600	-2.16485900	1.66009800
O	1.82646600	-3.65005700	-0.41761900
N	-2.26347300	-3.85539500	0.01902800
N	-4.46989000	1.93671200	-0.57023800
N	3.64238000	3.36035500	0.40167600
N	3.83447300	-2.74867300	0.30876000
C	-0.78527500	-2.16254000	-0.91569600
C	-1.17379400	-3.64792000	-0.92879000
H	-0.31279400	-4.14664300	-0.45874700
C	-2.22469000	-4.82935700	1.02552300
C	-3.48717900	-4.66963900	1.78758200
C	-4.18842100	-3.59299400	1.24776800
C	-3.38950700	-3.03671800	0.13243800
C	-2.22594400	1.07015400	-1.17087800
C	-3.74412400	0.97401800	-1.39052000
H	-3.99716100	0.01314900	-0.91967400
C	-5.51259600	1.54192300	0.29276000
C	-6.03494900	2.79466900	0.88682200
C	-5.29486300	3.86046000	0.37855500
C	-4.30262500	3.32330200	-0.58498400
C	1.61996800	2.52937200	-0.62456500
C	2.48793200	3.77852500	-0.39912600
H	1.90906400	4.40867800	0.29310300
C	3.93125400	3.89337700	1.67087500
C	4.94394700	2.99608600	2.26810400
C	5.20385500	1.96579100	1.36655500
C	4.33846100	2.15958200	0.18679700
C	2.61243900	-0.98131500	-0.82703800
C	3.96873100	-1.68975800	-0.68670900
H	4.61796800	-0.94331800	-0.20363700
C	4.65453600	-2.84364300	1.44931200
C	4.05395600	-3.90966100	2.28590100
C	2.89984800	-4.36483700	1.65017200
C	2.73432400	-3.60181400	0.39536700

C	-1.39854200	-4.35104300	-2.28853300
C	-0.07818800	-4.36733100	-3.08734500
C	-1.78833200	-5.81954200	-2.01761900
C	-2.50225200	-3.70926900	-3.14187100
H	0.70568900	-4.84737000	-2.48171300
H	0.25909900	-3.34296600	-3.28098500
C	-0.26098800	-5.12104700	-4.40710900
H	-2.73147700	-5.85863200	-1.45023900
H	-1.02133500	-6.29651300	-1.38840200
C	-1.96367300	-6.58970900	-3.32847300
H	-2.24269600	-2.66466800	-3.34908600
H	-3.45280900	-3.69893000	-2.58546300
C	-2.67798900	-4.47774900	-4.45501200
H	0.68797900	-5.09688300	-4.96341600
C	-0.65327900	-6.57133100	-4.11836600
C	-1.36104700	-4.44965900	-5.23589400
H	-2.24460800	-7.62792800	-3.09749100
C	-3.06634200	-5.92827600	-4.15905000
H	-3.47233100	-3.99699900	-5.04633800
H	0.14240100	-7.07047400	-3.54474500
H	-0.77106100	-7.13182400	-5.05858900
H	-1.08202300	-3.40917300	-5.46390500
H	-1.47967400	-4.96896600	-6.19937600
H	-3.21642200	-6.48172700	-5.09872900
H	-4.02230200	-5.95966200	-3.61358700
C	4.68123100	-2.14491900	-1.98097400
C	5.07687500	-0.91015000	-2.81556700
C	5.98573900	-2.87219800	-1.59181900
C	3.83806800	-3.10584700	-2.83687100
H	5.70053200	-0.24519300	-2.19756700
H	4.18729300	-0.33301700	-3.09008400
C	5.84258400	-1.33413600	-4.07056700
H	5.75199600	-3.76244700	-0.98820200
H	6.60135600	-2.21489500	-0.95685700
C	6.76629700	-3.29588800	-2.83926200
H	2.88748000	-2.63196000	-3.11195400
H	3.58588900	-4.00151300	-2.24995700
C	4.61518000	-3.51498200	-4.09180100
H	6.09224500	-0.43703600	-4.65580300
C	7.12681700	-2.06032500	-3.66650600
C	4.96718400	-2.26937800	-4.91008900

H	7.68408200	-3.81546500	-2.52630800
C	5.90225800	-4.23635200	-3.68408000
H	3.98865000	-4.18905300	-4.69492600
H	7.77305100	-1.38771400	-3.08193100
H	7.69774000	-2.35721600	-4.55989700
H	4.04800700	-1.74893600	-5.21873500
H	5.49694500	-2.55922600	-5.83075600
H	6.45880600	-4.55871700	-4.57763300
H	5.66084200	-5.14569600	-3.11232400
C	2.83408700	4.66300600	-1.60626900
C	1.52455900	5.14992700	-2.25782000
C	3.58618000	5.90547300	-1.08606200
C	3.71252000	3.97156900	-2.66021900
H	0.90882300	5.65939400	-1.49682500
H	0.94163700	4.29656300	-2.62411300
C	1.82654400	6.11032400	-3.40994100
H	4.52431700	5.59440400	-0.60097500
H	2.98283500	6.40440500	-0.30949600
C	3.89070900	6.87359900	-2.23143600
H	3.21871600	3.06412400	-3.02916700
H	4.65788600	3.64973700	-2.19764100
C	4.00342200	4.93923100	-3.81231600
H	0.87871400	6.42924900	-3.86715900
C	2.58022200	7.33058600	-2.87749500
C	2.68668700	5.38973200	-4.45275600
H	4.43176600	7.74395800	-1.83191000
C	4.75283300	6.16348300	-3.27886200
H	4.62183100	4.42598600	-4.56297100
H	1.96083500	7.86605600	-2.14159300
H	2.78878000	8.03678400	-3.69585300
H	2.14440700	4.51993400	-4.85293800
H	2.88992600	6.05985700	-5.30218200
H	4.98962300	6.85361300	-4.10325400
H	5.71189000	5.85546400	-2.83467800
C	-4.26412500	0.87192800	-2.84921000
C	-3.66144200	-0.38948900	-3.49966500
C	-5.79247500	0.65691800	-2.79317200
C	-3.98464000	2.09566400	-3.73632100
H	-3.89448700	-1.25711500	-2.86709100
H	-2.56954000	-0.31386800	-3.54522600
C	-4.21899500	-0.60080600	-4.90743800

H	-6.27951800	1.53480700	-2.33819900
H	-6.01940600	-0.20396100	-2.14473300
C	-6.36724400	0.43835900	-4.19569300
H	-2.90641300	2.29211600	-3.77840700
H	-4.45190400	2.99010700	-3.30186900
C	-4.54694600	1.86535400	-5.14441000
H	-3.74396400	-1.49296000	-5.34451100
C	-5.73226000	-0.80220300	-4.82909800
C	-3.90249500	0.62476200	-5.76816500
H	-7.45583400	0.30026700	-4.11662500
C	-6.06239800	1.66213200	-5.06401800
H	-4.32569900	2.74733200	-5.76316200
H	-5.96089900	-1.69747700	-4.22951700
H	-6.15258400	-0.97080100	-5.83257900
H	-2.81306200	0.76182900	-5.84214400
H	-4.27801800	0.47767500	-6.79266800
H	-6.48279100	1.52407200	-6.07212800
H	-6.54147400	2.55742500	-4.63814100
C	5.52812400	3.01997300	3.52347100
C	6.37643000	1.95997500	3.85709000
C	6.62928000	0.92467800	2.95379900
C	6.05149000	0.91937200	1.68194600
C	4.41575200	-4.37145300	3.53868100
C	3.58348200	-5.32318600	4.13625500
C	2.42938100	-5.78114300	3.49482500
C	2.06454500	-5.30282200	2.23268100
C	-3.97948400	-5.38163600	2.86768400
C	-5.20795500	-4.97738400	3.39827300
C	-5.91132100	-3.89881400	2.85450800
C	-5.41065400	-3.18757400	1.75963800
C	-7.04785700	2.99059900	1.81071300
C	-7.29101000	4.30186200	2.22877200
C	-6.53932600	5.36972400	1.73057900
C	-5.52703000	5.16265900	0.78920300
H	-7.61912900	2.14975900	2.19910700
H	-8.07729000	4.49722400	2.95543100
H	-6.75155600	6.37897200	2.07784700
H	-4.94387600	5.98980700	0.38861900
H	-5.94310200	-2.33864200	1.33108000
H	-6.86279500	-3.60545600	3.29352400
H	-5.62592700	-5.50925700	4.25084500

H	-3.42196300	-6.21741600	3.28731200
H	5.29925400	-3.98466000	4.04211700
H	3.83112500	-5.70725000	5.12427700
H	1.79606100	-6.51245800	3.99387900
H	1.14872400	-5.62648200	1.73810400
H	6.22664300	0.09646100	0.99289900
H	7.26599700	0.09383100	3.24955800
H	6.83637400	1.93033200	4.84298100
H	5.31428300	3.82426800	4.22487900
C	-0.82690200	1.98017800	2.60168200
C	0.33968300	1.31885500	3.28048000
C	0.58259700	-0.12384700	2.85798600
H	1.23442600	1.87583200	2.98899800
C	-2.20085900	1.50996700	2.78604100
C	-3.10864800	2.40875900	3.38985300
C	-2.66606000	0.20863000	2.50864900
C	-4.37853000	2.01030500	3.78702700
H	-2.78625500	3.42473100	3.60914100
C	-3.93532300	-0.19263600	2.88023100
H	-2.03729500	-0.48922600	1.96390600
C	-4.77596700	0.70073600	3.53899000
H	-5.04946200	2.70742800	4.28256600
H	-4.27466900	-1.20024500	2.66097400
H	0.24452100	1.45581200	4.37636200
C	-0.23230000	-1.18020900	3.54176800
C	-0.21482700	-2.51902100	3.10720500
C	-1.01281900	-0.89057000	4.67705900
C	-0.93250200	-3.50559900	3.77776300
H	0.35248500	-2.78104500	2.21837300
C	-1.74490400	-1.86973200	5.33596300
H	-1.05792800	0.12944800	5.05526800
C	-1.70988700	-3.18863600	4.88933300
H	-0.88731400	-4.53260900	3.41718900
H	-2.34610000	-1.59827100	6.20330200
H	-2.28253500	-3.96119900	5.40095800
C	2.06005000	-0.43279900	3.04808200
C	2.64733700	-0.47317700	4.48960600
O	1.78357300	-0.05141300	5.41986100
O	3.76518200	-0.87840900	4.70367400
C	2.18034000	-0.30510700	6.78894500
C	1.94450700	-1.75498700	7.13381500

H	1.55081400	0.36947300	7.37602500
H	3.23072100	-0.01861200	6.90702800
H	2.17342900	-1.93707500	8.18972600
H	0.90021900	-2.03181100	6.95096900
H	2.58557100	-2.40490500	6.52850200
F	2.86826000	0.46505500	2.39290300
F	2.37351800	-1.68521900	2.59396700
C	-0.66118500	3.33755100	2.14272100
C	-1.57606200	3.86567100	1.20511300
C	0.38752900	4.17953100	2.57742600
C	-1.45511600	5.14812400	0.70506600
H	-2.34755900	3.20552600	0.82788500
C	0.48133000	5.49092600	2.14138900
H	1.12746900	3.82536500	3.29002400
C	-0.43136800	5.96069300	1.19623500
H	-2.13082100	5.50078600	-0.06925200
H	1.28881900	6.12712200	2.48869700
Cl	-0.26619500	7.57965900	0.59225100
Cl	-6.35945000	0.17158200	4.03564800

Si-TS2

Zero-point correction=	1.891380 (Hartree/Particle)
Thermal correction to Energy=	2.001039
Thermal correction to Enthalpy=	2.001983
Thermal correction to Gibbs Free Energy=	1.744433
Sum of electronic and zero-point Energies=	-6973.651255
Sum of electronic and thermal Energies=	-6973.541596
Sum of electronic and thermal Enthalpies=	-6973.540652
Sum of electronic and thermal Free Energies=	-6973.798202

Rh	-0.18210000	0.21846400	-0.42145100
Rh	-0.45856600	0.40524600	2.00539200
O	0.92855300	-1.52235500	-0.08014900
O	0.68902400	-1.31308200	2.16554600
O	2.27523400	-5.49706500	-0.54105800
O	3.84719800	-1.39102400	0.79176400
O	1.65780400	1.27993900	-0.06772300
O	1.27837300	1.48545600	2.14712000
O	5.84287100	1.41936800	-0.36893000
O	2.54914600	4.42963800	0.75119400

O	-1.45829200	1.86894600	-0.54359400
O	-1.64594000	2.07617700	1.70474200
O	-4.24000200	4.64982300	-2.14567200
O	-4.22374100	0.84281200	0.41959800
O	-1.90666900	-0.94406200	-0.29273400
O	-2.12895000	-0.78578200	1.95179300
O	-5.11204600	-3.02722100	-1.70697400
O	-1.29185100	-3.75606600	0.73006600
N	2.82687200	-3.43822400	0.39458800
N	4.06074200	2.69767700	0.42075500
N	-4.01918200	2.90054600	-0.62220600
N	-3.35448900	-3.23144700	-0.19063900
C	1.06645900	-1.91989400	1.12180300
C	1.68143800	-3.31502500	1.28693200
H	0.92749200	-3.99211900	0.85676800
C	3.02346700	-4.54124100	-0.44698500
C	4.29614000	-4.27834100	-1.16046200
C	4.77867700	-3.03699600	-0.74888900
C	3.82163500	-2.47084800	0.22992100
C	2.02462600	1.51411400	1.12651400
C	3.52896300	1.71547700	1.35455500
H	3.96677200	0.76856200	1.00396400
C	5.19816900	2.44867700	-0.37135600
C	5.40306100	3.68176700	-1.16794300
C	4.38937900	4.58340000	-0.84672400
C	3.53176300	3.96847300	0.19626600
C	-1.94649900	2.37702300	0.51425100
C	-2.99647300	3.47739600	0.25462200
H	-2.49768200	4.22656000	-0.38149100
C	-4.46735200	3.50288400	-1.81036300
C	-5.23499900	2.45855400	-2.52693500
C	-5.22975700	1.29924800	-1.75281900
C	-4.46278100	1.57542200	-0.52211800
C	-2.46886100	-1.21056100	0.81083500
C	-3.70254200	-2.11597600	0.68413000
H	-4.41914700	-1.52343200	0.09792100
C	-4.06886800	-3.54559000	-1.36165700
C	-3.28058000	-4.60443000	-2.03761900
C	-2.12847200	-4.83550000	-1.28766400
C	-2.14627700	-3.92453100	-0.12362300
C	1.97292300	-3.82160200	2.72300500

C	0.65094800	-3.96411300	3.50645400
C	2.59995100	-5.22951700	2.63022600
C	2.94355700	-2.92179200	3.50439700
H	-0.02740400	-4.63142400	2.95227500
H	0.14672100	-2.99497400	3.57973400
C	0.91543600	-4.52395400	4.90664000
H	3.55390500	-5.18075700	2.08191900
H	1.93840000	-5.89349600	2.05371300
C	2.85997600	-5.80854300	4.02329100
H	2.53287700	-1.90826500	3.58084600
H	3.89738200	-2.83869900	2.96054000
C	3.19702600	-3.49598300	4.90153200
H	-0.04039200	-4.58999900	5.44739000
C	1.54261800	-5.91455400	4.79343600
C	1.87001500	-3.59122100	5.65962500
H	3.30940000	-6.80677300	3.91499400
C	3.81899300	-4.88911400	4.78298400
H	3.88645300	-2.82920000	5.44095000
H	0.85363800	-6.59829800	4.27465300
H	1.72236600	-6.33799500	5.79363900
H	1.42211300	-2.59016100	5.75888600
H	2.04218900	-3.96840900	6.67946300
H	4.02616500	-5.30083700	5.78268500
H	4.78390600	-4.82858600	4.25608100
C	-4.42673600	-2.54880800	1.97759000
C	-4.99761100	-1.30600200	2.69029500
C	-5.62357400	-3.44257200	1.58827000
C	-3.53524000	-3.34486400	2.94625900
H	-5.65461200	-0.76274300	1.99315700
H	-4.19116600	-0.61478100	2.95844200
C	-5.78073800	-1.71699800	3.93885700
H	-5.26326600	-4.34463700	1.07009200
H	-6.26893200	-2.90652600	0.87409700
C	-6.42288800	-3.85459700	2.82771800
H	-2.65579300	-2.75176700	3.22499500
H	-3.15897800	-4.24951600	2.44529100
C	-4.33230300	-3.74233200	4.19246300
H	-6.15528300	-0.81120300	4.43806700
C	-6.95706700	-2.60798000	3.53597700
C	-4.85810400	-2.48478300	4.89040000
H	-7.26198600	-4.49381300	2.51536900

C	-5.51206700	-4.62871200	3.78471100
H	-3.67225100	-4.29644300	4.87637000
H	-7.63867200	-2.05600900	2.87085600
H	-7.54048700	-2.89855400	4.42331800
H	-4.01723200	-1.84484700	5.19790500
H	-5.40347200	-2.76046600	5.80626900
H	-6.07947400	-4.94225300	4.67456100
H	-5.14513800	-5.54651500	3.29976900
C	-3.55933000	4.23313300	1.47264600
C	-2.40017800	4.94065200	2.20267700
C	-4.52447100	5.32455000	0.96459300
C	-4.32971600	3.33761900	2.45752100
H	-1.86829400	5.59376100	1.48968100
H	-1.67378900	4.20612700	2.56766800
C	-2.93365800	5.76941600	3.37306600
H	-5.36478400	4.85628400	0.42988200
H	-4.00961300	5.96583700	0.23307700
C	-5.05968400	6.16288500	2.12766900
H	-3.68669600	2.52675700	2.81963000
H	-5.17459400	2.86104200	1.93653700
C	-4.85072300	4.17706400	3.62900800
H	-2.08739800	6.24734100	3.88758300
C	-3.89210000	6.84019100	2.84890900
C	-3.67453700	4.84689400	4.34640500
H	-5.74556100	6.92626100	1.73188800
C	-5.80567600	5.25405900	3.10793600
H	-5.38348700	3.51828600	4.33041900
H	-3.36193200	7.51524300	2.15908700
H	-4.26647000	7.45831800	3.67940100
H	-2.98647600	4.08281500	4.73868800
H	-4.03893600	5.42423900	5.21004500
H	-6.20461500	5.84697400	3.94545500
H	-6.66765600	4.78538000	2.60868600
C	4.02213500	1.90160900	2.81233800
C	3.69460700	0.62667200	3.61737800
C	5.56042700	2.03295300	2.79191400
C	3.44984700	3.13605600	3.52841600
H	4.13850100	-0.24101000	3.10573300
H	2.61242600	0.45793500	3.64476300
C	4.23634400	0.73035100	5.04384000
H	5.85243900	2.93343200	2.22830400

H	5.99765300	1.17275100	2.26211700
C	6.12148600	2.13069400	4.21351100
H	2.35433800	3.09280300	3.53986500
H	3.72365900	4.04678800	2.97625400
C	3.99945000	3.21988500	4.95687700
H	3.95869400	-0.18129600	5.59512900
C	5.75827700	0.87023100	5.00198400
C	3.62419100	1.95376300	5.73202300
H	7.21552600	2.23193600	4.15774500
C	5.52368600	3.35454800	4.91265200
H	3.56539000	4.09942100	5.45452500
H	6.20495900	-0.01763000	4.52833200
H	6.16834000	0.93136500	6.02189300
H	2.52940200	1.85101900	5.77907000
H	3.98464200	2.02498700	6.76991000
H	5.92933800	3.44278500	5.93235100
H	5.80601800	4.27331700	4.37565600
C	-5.83733400	2.48895300	-3.77370300
C	-6.42077800	1.30424700	-4.23375500
C	-6.39875100	0.13982500	-3.46154000
C	-5.80691100	0.12205900	-2.19574500
C	-3.50309800	-5.26156700	-3.23464000
C	-2.53188200	-6.17651100	-3.65449300
C	-1.38076600	-6.41046800	-2.89765200
C	-1.15821600	-5.73565900	-1.69357400
C	4.97859100	-5.04474300	-2.08940200
C	6.17048000	-4.52482200	-2.60239800
C	6.65640200	-3.28279200	-2.18483800
C	5.96614800	-2.51807700	-1.23980500
C	6.36815000	3.98372900	-2.11389200
C	6.27767500	5.22457900	-2.75071500
C	5.25215200	6.12217500	-2.44123500
C	4.29044300	5.81366600	-1.47489100
H	7.15365400	3.27045400	-2.35629200
H	7.01554700	5.49745900	-3.50281600
H	5.20680100	7.07891300	-2.95781800
H	3.49370200	6.51068300	-1.22173200
H	6.33423800	-1.54771100	-0.90669400
H	7.58700200	-2.90446500	-2.60297600
H	6.73310900	-5.09618800	-3.33818300
H	4.59223800	-6.01211500	-2.40637100

H	-4.39190600	-5.05506300	-3.82761600
H	-2.66960500	-6.71169700	-4.59250400
H	-0.64031700	-7.12222500	-3.25833500
H	-0.25103100	-5.88802600	-1.10818000
H	-5.76736500	-0.79463800	-1.60963900
H	-6.83329700	-0.77648100	-3.85572200
H	-6.89046800	1.28376800	-5.21542500
H	-5.83951100	3.39818500	-4.37207100
C	0.62681200	1.60968900	-3.06209800
C	-0.10946700	0.71993100	-4.01059000
C	-0.24616100	-0.59598200	-3.28032600
H	-1.10289400	1.12178600	-4.21540500
C	2.09679000	1.41736200	-3.02082500
C	2.85667600	2.33775900	-3.76470400
C	2.76022400	0.32428300	-2.45098700
C	4.21213000	2.13595100	-4.00415500
H	2.36696000	3.20885400	-4.19894300
C	4.11450300	0.12214600	-2.66556400
H	2.20933100	-0.37579800	-1.82722700
C	4.82560700	1.01238300	-3.46302100
H	4.78287200	2.84445400	-4.59942600
H	4.61797900	-0.72642300	-2.21435100
H	0.38917400	0.65177300	-4.99011600
C	0.71423700	-1.66859300	-3.56993200
C	0.68745800	-2.91358300	-2.89852600
C	1.67960400	-1.52524800	-4.59425700
C	1.51381600	-3.96017100	-3.29014100
H	0.02419800	-3.05193600	-2.05061500
C	2.51773400	-2.56449900	-4.96624200
H	1.79053400	-0.57550700	-5.11095700
C	2.42935000	-3.79969100	-4.32824800
H	1.45584000	-4.90917200	-2.76080100
H	3.24446300	-2.40607000	-5.76178700
H	3.08103400	-4.62121400	-4.62180400
C	-1.69224600	-0.99914600	-3.25063100
C	-2.31369700	-1.39090500	-4.62742700
O	-1.44177900	-1.24336500	-5.63140500
O	-3.43735900	-1.82267600	-4.72030500
C	-1.83608700	-1.83117600	-6.89387300
C	-1.53853500	-3.30968100	-6.87627500
H	-1.23843900	-1.29511900	-7.63598600

H	-2.89825000	-1.62418400	-7.06105800
H	-1.74440600	-3.75366100	-7.85636300
H	-0.48659600	-3.48856600	-6.62644800
H	-2.16117000	-3.81861800	-6.13261200
F	-2.49000500	0.00627900	-2.76671000
F	-1.93079300	-2.12131400	-2.51312600
C	0.09840500	2.88716800	-2.67933100
C	0.81474300	3.68353700	-1.75526200
C	-1.07861400	3.44228400	-3.23430000
C	0.39823700	4.95063000	-1.39723900
H	1.69207200	3.25216400	-1.28825500
C	-1.46796000	4.73712600	-2.94334900
H	-1.69365200	2.87064000	-3.92546000
C	-0.73967400	5.47740100	-2.01065900
H	0.94024800	5.51127300	-0.64014900
H	-2.35783400	5.16245400	-3.39283800
Cl	-1.28972500	7.06584000	-1.57724200
Cl	6.52097600	0.72335800	-3.76757700

Si-pro

Zero-point correction= 0.389499 (Hartree/Particle)

Thermal correction to Energy= 0.417144

Thermal correction to Enthalpy= 0.418088

Thermal correction to Gibbs Free Energy= 0.328646

Sum of electronic and zero-point Energies= -2234.641479

Sum of electronic and thermal Energies= -2234.613834

Sum of electronic and thermal Enthalpies= -2234.612890

Sum of electronic and thermal Free Energies= -2234.702332

C	-0.56231600	0.06236200	-0.64667700
C	0.27487600	-0.47992000	-1.76020400
C	0.77647300	-0.65143900	-0.35364700
H	-0.06303700	-1.38379700	-2.25862800
C	-0.58730300	1.55316200	-0.45533700
C	-0.86902300	2.39363400	-1.53376100
C	-0.37180800	2.12798100	0.80073600
C	-0.92944500	3.77463400	-1.37584500
H	-1.04628500	1.95886600	-2.51790800
C	-0.42378600	3.50481800	0.97953700
H	-0.12709200	1.48902300	1.64897000
C	-0.70281600	4.31787900	-0.11558300
H	-1.14826500	4.42559900	-2.21826500

H	-0.24247500	3.94866100	1.95478300
H	0.79251400	0.21912000	-2.41143500
C	1.94753000	0.14448900	0.14818900
C	2.40776300	-0.02247600	1.46362800
C	2.59788200	1.08835300	-0.65588500
C	3.48066800	0.72121400	1.94704600
H	1.92733900	-0.74176200	2.12153800
C	3.66247100	1.83871500	-0.16928600
H	2.26846300	1.25569700	-1.67901000
C	4.11306300	1.65725200	1.13543800
H	3.81755600	0.56601000	2.97047300
H	4.14336600	2.56863100	-0.81810800
H	4.94756400	2.24308700	1.51594200
C	0.71695000	-2.06387400	0.19967200
C	2.08805600	-2.76521100	0.20836800
O	2.60648300	-2.71674000	-1.02414700
O	2.59031000	-3.26973100	1.18202100
C	3.95803000	-3.22963600	-1.14305700
C	4.95339400	-2.18915700	-0.69538100
H	4.05577000	-3.47308000	-2.20429100
H	4.03174400	-4.14828600	-0.55198000
H	5.97495800	-2.55513800	-0.84588100
H	4.83220600	-1.26003200	-1.26258600
H	4.82573200	-1.96153300	0.36805600
F	-0.13634000	-2.85236900	-0.52557300
F	0.24306400	-2.06741300	1.48015400
C	-1.88291900	-0.58699800	-0.33687500
C	-2.36879600	-0.64256600	0.97273600
C	-2.69373200	-1.06854400	-1.36644900
C	-3.61946900	-1.17712600	1.25422800
H	-1.75272700	-0.27428100	1.79112600
C	-3.95030700	-1.60503100	-1.10691400
H	-2.33706200	-1.02330200	-2.39523200
C	-4.40215500	-1.65571100	0.20713400
H	-3.98754000	-1.22703100	2.27554200
H	-4.57547300	-1.97971600	-1.91312200
Cl	-5.97496100	-2.32893200	0.54952200
Cl	-0.76338100	6.04906500	0.09547400

***Re*-face attack:**

Re-TS1-int

Zero-point correction=	1.889039 (Hartree/Particle)
Thermal correction to Energy=	2.000014
Thermal correction to Enthalpy=	2.000958
Thermal correction to Gibbs Free Energy=	1.741257
Sum of electronic and zero-point Energies=	-6973.680399
Sum of electronic and thermal Energies=	-6973.569424
Sum of electronic and thermal Enthalpies=	-6973.568480
Sum of electronic and thermal Free Energies=	-6973.828181

Rh	-0.43089000	0.06944500	-0.45129200
Rh	-0.87620500	0.28433600	1.96606900
O	1.07768800	1.46791000	-0.22421000
O	0.65770000	1.68867200	1.99535000
O	3.42681500	4.69147100	-1.29857200
O	-0.61131300	4.14740400	0.84086200
O	-1.79527000	1.63104800	-0.62327800
O	-2.30402200	1.73623900	1.58490800
O	-3.95748500	4.20963600	-2.65997400
O	-4.49914900	0.28687400	-0.33532100
O	-1.97765500	-1.30018200	-0.43067800
O	-2.37864200	-1.14825200	1.79707800
O	-4.48555000	-3.66071500	-2.21560200
O	-1.00800300	-3.92627100	0.75491900
O	0.90637700	-1.47878200	0.06841900
O	0.47286400	-1.21466900	2.27620000
O	3.15969600	-5.11390500	0.05288800
O	3.69882300	-0.76508700	1.43968300
N	1.57999500	4.23868200	0.05112900
N	-4.25038700	2.43855500	-1.17760100
N	-2.99251400	-3.73773600	-0.43005700
N	3.13865000	-2.97114600	0.96463100
C	1.22527000	2.01602900	0.92138000
C	2.19914300	3.20016100	0.88473600
H	3.05983400	2.86017300	0.28631500
C	2.25834100	4.85881400	-1.01576200
C	1.24914000	5.70576400	-1.69641400
C	0.02376200	5.52778400	-1.05675200
C	0.21901700	4.58056300	0.06466200

C	-2.46876500	2.04750600	0.37961900
C	-3.56624100	3.03485400	-0.03039400
H	-3.02986500	3.89610300	-0.45959200
C	-4.28445900	3.05876700	-2.43749000
C	-4.74693100	2.01402100	-3.38384600
C	-4.84389600	0.80726100	-2.69047300
C	-4.53093300	1.06537500	-1.26998800
C	-2.56798300	-1.62906100	0.64744200
C	-3.62450200	-2.71315700	0.40322000
H	-4.35701000	-2.23643000	-0.26706300
C	-3.44038900	-4.04529200	-1.72694800
C	-2.37217400	-4.87733900	-2.33372100
C	-1.31461900	-4.96026600	-1.42942100
C	-1.68137700	-4.18626400	-0.22576700
C	1.01695400	-1.79576500	1.29630000
C	1.83076400	-3.05708200	1.59685800
H	1.31056800	-3.85738700	1.04914400
C	3.72548700	-4.06945600	0.31176600
C	5.12308600	-3.66514800	0.03555900
C	5.27994500	-2.33643700	0.42995400
C	3.99412700	-1.86681300	1.00706800
C	2.75831800	3.71740200	2.22091300
C	3.44935600	2.54953600	2.95129000
C	3.82887700	4.78372800	1.90875700
C	1.70448200	4.35320700	3.14120900
H	4.20416500	2.10061900	2.28539600
H	2.72481700	1.75754000	3.17861000
C	4.10611800	3.04522200	4.24115900
H	3.36316000	5.63340900	1.38533000
H	4.57992000	4.36694200	1.21967300
C	4.49396200	5.27858000	3.19404200
H	0.90764800	3.63358200	3.36255200
H	1.23043100	5.20348500	2.62591700
C	2.36883000	4.83929000	4.43462800
H	4.57917100	2.19340600	4.75117100
C	5.16194100	4.10179300	3.90863300
C	3.03293100	3.65720000	5.14751800
H	5.25037800	6.03474100	2.93769000
C	3.43043000	5.89322500	4.10835900
H	1.60006600	5.27837400	5.08716700
H	5.94585500	3.66855900	3.26751800

H	5.65759900	4.44895600	4.82818700
H	2.27890300	2.89765700	5.40344600
H	3.48399600	3.99342200	6.09376200
H	3.89530200	6.26367600	5.03499300
H	2.96383200	6.76060300	3.61626400
C	1.91387300	-3.50291200	3.08424000
C	0.49990500	-3.84775000	3.59833400
C	2.75058500	-4.79875200	3.17374100
C	2.57800900	-2.46275300	4.00483800
H	0.06310100	-4.62259800	2.95068300
H	-0.15918900	-2.97634100	3.52798000
C	0.55689200	-4.34409300	5.04405400
H	3.77929500	-4.60774500	2.82870100
H	2.32923500	-5.56266300	2.50333600
C	2.80653800	-5.32099300	4.61303000
H	2.04449500	-1.50716000	3.95383000
H	3.60663000	-2.27062300	3.66174800
C	2.61475500	-2.97747300	5.44789600
H	-0.46837600	-4.55023600	5.38674100
C	1.39265800	-5.62274600	5.11237300
C	1.18986900	-3.26678900	5.92932800
H	3.41210800	-6.23920400	4.63204900
C	3.44496300	-4.26122200	5.51367800
H	3.07156800	-2.20835100	6.08790400
H	0.93105300	-6.40881100	4.49563200
H	1.43043300	-6.00471000	6.14427300
H	0.58599700	-2.34735000	5.89354200
H	1.20665400	-3.59930700	6.97876400
H	3.50002000	-4.62905900	6.54988000
H	4.47803500	-4.06001100	5.19029100
C	-4.40702800	-3.26640300	1.60652800
C	-5.21745500	-2.12314400	2.25043500
C	-5.41112000	-4.31445200	1.08391400
C	-3.52352100	-3.93674700	2.67180100
H	-5.85694300	-1.65521300	1.48442900
H	-4.54207300	-1.34103100	2.61672300
C	-6.07542000	-2.65928900	3.39873100
H	-4.86610500	-5.14535000	0.61013700
H	-6.04132800	-3.86439800	0.30026200
C	-6.27765800	-4.85239000	2.22505900
H	-2.77875200	-3.22371200	3.04767900

H	-2.96258100	-4.76817200	2.21960400
C	-4.39184300	-4.46329400	3.81910700
H	-6.62991300	-1.82381600	3.85071500
C	-7.05926800	-3.70153500	2.86282500
C	-5.16860700	-3.30467200	4.45113400
H	-6.97784500	-5.59918600	1.82234500
C	-5.37918900	-5.50240700	3.28058400
H	-3.74208000	-4.92839500	4.57498800
H	-7.73043800	-3.24207300	2.12114300
H	-7.69447200	-4.08191400	3.67755300
H	-4.46891400	-2.55753800	4.85514600
H	-5.77146800	-3.67024600	5.29668700
H	-5.99198800	-5.90658300	4.10099000
H	-4.83262500	-6.35138700	2.84186200
C	-4.51396400	3.58310700	1.05153700
C	-3.70774700	4.40363500	2.07672700
C	-5.51134200	4.53968000	0.36600500
C	-5.31789100	2.49320200	1.78065000
H	-3.14236400	5.18930200	1.54974000
H	-2.96515100	3.76736500	2.57194900
C	-4.64465400	5.03280400	3.11066900
H	-6.10131500	3.98784900	-0.38141700
H	-4.95865400	5.31889700	-0.18442000
C	-6.45010100	5.17522600	1.39340100
H	-4.63641600	1.77752100	2.25766400
H	-5.91366800	1.92275700	1.05301000
C	-6.24517400	3.13198000	2.81972900
H	-4.04461900	5.60034900	3.83677000
C	-5.63060700	5.97127800	2.41103600
C	-5.41698500	3.92563000	3.83411100
H	-7.15104200	5.84480900	0.87331500
C	-7.22823600	4.07371500	2.11884500
H	-6.80117100	2.33690000	3.33769000
H	-5.08562600	6.78417700	1.90660200
H	-6.29782100	6.44313200	3.14859700
H	-4.71646900	3.25652100	4.35584400
H	-6.07527800	4.36227700	4.60090800
H	-7.91757300	4.51975700	2.85212300
H	-7.84504900	3.51055900	1.40169200
C	-2.28646600	-5.44543000	-3.59346900
C	-1.09889200	-6.10569200	-3.92124700

C	-0.03412000	-6.17295600	-3.01762200
C	-0.12462100	-5.59337400	-1.74873400
C	6.17030800	-4.39544100	-0.50034600
C	7.40056600	-3.74805500	-0.63748900
C	7.56015400	-2.41562500	-0.24829900
C	6.49778400	-1.68896200	0.29640900
C	1.37160000	6.52029800	-2.81019600
C	0.21280100	7.15045500	-3.27552300
C	-1.01880200	6.95928100	-2.64183300
C	-1.13055300	6.14322600	-1.51252300
C	-4.99637800	2.08252800	-4.74370100
C	-5.33194200	0.89287300	-5.39698500
C	-5.39866800	-0.31959200	-4.70400900
C	-5.15690400	-0.38228700	-3.32824600
H	-4.91451500	3.02689100	-5.27935100
H	-5.53400400	0.90639800	-6.46678300
H	-5.64321000	-1.23164300	-5.24485800
H	-5.19119000	-1.32615800	-2.78473600
H	-2.09151000	5.98026800	-1.02716200
H	-1.90698100	7.44676600	-3.03818400
H	0.26664500	7.79381800	-4.15165700
H	2.33228800	6.64973100	-3.30465900
H	6.02801900	-5.43014000	-0.80437400
H	8.25006100	-4.28871300	-1.05085900
H	8.53098600	-1.93767800	-0.36453300
H	6.62443000	-0.65432600	0.60828400
H	0.71232300	-5.61819000	-1.05070200
H	0.88453400	-6.67965500	-3.30913800
H	-0.99263100	-6.56515800	-4.90307600
H	-3.10992700	-5.36415400	-4.30034500
F	-0.23223400	-2.40841000	-2.19454500
F	-2.03294000	-1.71491300	-3.24055400
O	0.94180400	-0.94639800	-4.78245100
O	-0.34814900	-2.80843700	-4.91270900
C	0.07035900	-1.67116900	-4.35847600
C	4.98310000	1.81188900	-1.84484900
C	5.28602400	1.47597300	-0.51833900
H	4.75854000	0.65133800	-0.03645700
C	6.56906300	3.64806000	-1.70487800
H	7.07285700	4.49838300	-2.15687600
C	-0.69769900	-1.43640700	-3.03933800

C	6.83186700	3.30587100	-0.38294800
C	-0.80016600	1.04489700	-3.30472100
C	5.64460000	2.89942100	-2.42436700
H	5.41855600	3.16926700	-3.45515500
C	-0.51785900	-0.06451900	-2.43968200
C	3.94333600	1.05618100	-2.57591600
C	2.94773700	1.68735600	-3.22061800
C	-1.50483000	0.90538500	-4.53074500
H	-1.91636200	-0.06019500	-4.81467000
C	6.19885500	2.22119900	0.21938200
H	6.40773000	1.97450000	1.25935500
C	-1.72364000	1.99271300	-5.35716000
H	-2.27917000	1.86127200	-6.28395400
C	-0.34002600	2.34081500	-2.95814600
H	0.21814800	2.45819200	-2.03130900
C	-1.24186300	3.25249300	-4.99912700
H	-1.41326200	4.10902800	-5.64946200
C	-0.54454700	3.41847600	-3.80297600
H	-0.16298900	4.40029500	-3.53528800
C	0.36340100	-3.22060800	-6.10817900
H	-0.33229200	-3.91136000	-6.59350700
H	0.50278800	-2.33992100	-6.74473700
C	1.67450900	-3.87460800	-5.75924500
H	1.52523600	-4.70104200	-5.05527600
H	2.36305200	-3.15523900	-5.30426400
H	2.14856900	-4.27454800	-6.66242600
H	2.86232700	2.77395800	-3.19734800
H	2.19675100	1.13213700	-3.78201800
C	4.03295700	-0.42127300	-2.60638600
C	5.23843500	-1.05088600	-2.95096200
C	2.90688900	-1.22113900	-2.38356700
C	5.29746600	-2.42193500	-3.16517400
H	6.13547600	-0.44781300	-3.09352100
C	2.95423100	-2.60171100	-2.55767600
H	1.97947200	-0.75024200	-2.06021800
C	4.14405700	-3.18248100	-2.98424600
H	6.22465200	-2.90216900	-3.46799400
H	2.07429000	-3.21655200	-2.37296500
Cl	7.96006400	4.26070200	0.54804700
Cl	4.20178200	-4.90558500	-3.28154800

Re-TS1

Zero-point correction= 1.889277 (Hartree/Particle)
Thermal correction to Energy= 1.999071
Thermal correction to Enthalpy= 2.000015
Thermal correction to Gibbs Free Energy= 1.743447
Sum of electronic and zero-point Energies= -6973.642374
Sum of electronic and thermal Energies= -6973.532579
Sum of electronic and thermal Enthalpies= -6973.531635
Sum of electronic and thermal Free Energies= -6973.788203

Rh	0.27640100	-0.02693800	-0.38068400
Rh	0.26828100	1.39301700	1.64241500
O	0.22499300	-1.70043800	0.89348600
O	0.14990600	-0.35321800	2.71877100
O	0.68125200	-5.58327100	2.61395200
O	2.98070600	-1.60703200	2.38651200
O	2.35143600	0.08726600	-0.16524200
O	2.33034900	1.34948400	1.72076000
O	6.33820500	-0.49321200	-1.12381000
O	3.17171200	2.84258200	-1.04091300
O	0.27091000	1.80875000	-1.33675500
O	0.35472100	3.11965600	0.50980100
O	0.70279000	4.11307500	-4.56413900
O	-2.46256100	2.96264700	-1.43940600
O	-1.83550100	0.02491600	-0.34474700
O	-1.76281200	1.42156000	1.43993600
O	-6.27350500	-0.29854300	-0.69411200
O	-2.78606300	-1.54056500	2.04422200
N	1.54997400	-3.42599300	2.64702400
N	4.75663800	1.16796700	-0.71905900
N	-0.72332300	3.78362800	-2.75267900
N	-4.42301100	-0.56478800	0.70046500
C	0.22219300	-1.47279900	2.14728800
C	0.32507700	-2.72339800	3.03196000
H	-0.48877800	-3.39530300	2.71307400
C	1.60428800	-4.81187400	2.43922300
C	2.98559500	-5.09566800	1.98198600
C	3.68908900	-3.89276300	1.94083300
C	2.76938400	-2.80333500	2.33878000
C	2.90060900	0.75957600	0.76480500

C	4.43011900	0.79565200	0.65723900
H	4.75044200	-0.25593200	0.73491000
C	5.65026900	0.43107300	-1.51242900
C	5.54074900	1.00800800	-2.87556200
C	4.55179400	1.99028900	-2.85793400
C	4.03704700	2.10526500	-1.47526100
C	0.28261900	2.92232300	-0.73460200
C	0.23579300	4.11202100	-1.69880600
H	1.21073800	4.09363700	-2.21216600
C	-0.36421500	3.74706400	-4.10994500
C	-1.52999700	3.16485800	-4.81911500
C	-2.48221800	2.80899500	-3.86771200
C	-1.95970400	3.16520500	-2.52919200
C	-2.36442900	0.73737300	0.56933200
C	-3.90138100	0.79820500	0.60894700
H	-4.22069200	1.15921600	-0.38225400
C	-5.60377200	-0.97814600	0.05808600
C	-5.81903500	-2.38267600	0.48252000
C	-4.74684100	-2.76498200	1.28459100
C	-3.83518400	-1.60493200	1.43113700
C	0.19476600	-2.52987700	4.56135100
C	-1.18642900	-1.92942500	4.89049200
C	0.26882900	-3.91414900	5.24093700
C	1.30781700	-1.65507500	5.16732800
H	-1.97197800	-2.57991800	4.47280100
H	-1.30400700	-0.95281000	4.40847800
C	-1.36488700	-1.79510100	6.40432800
H	1.24353800	-4.38125800	5.03376100
H	-0.49314500	-4.58205100	4.81051200
C	0.08210400	-3.79419800	6.75555600
H	1.32173700	-0.66858800	4.69124700
H	2.28748000	-2.11694900	4.96758700
C	1.10830000	-1.51989200	6.68053600
H	-2.34763200	-1.34461600	6.60777700
C	-1.28361700	-3.17567100	7.05824400
C	-0.26041800	-0.89471600	6.96599300
H	0.14399900	-4.79762500	7.20219700
C	1.18217800	-2.90126600	7.33475300
H	1.90057700	-0.87474000	7.08743500
H	-2.08715100	-3.82344100	6.67487400
H	-1.42978600	-3.09409400	8.14635800

H	-0.32155400	0.10468800	6.50947700
H	-0.39566900	-0.76138600	8.05041000
H	1.06372900	-2.81500700	8.42589500
H	2.17025400	-3.35235800	7.15504000
C	-4.51893900	1.74651100	1.67674700
C	-4.09384500	3.19843500	1.37288700
C	-6.05995200	1.69999900	1.58080900
C	-4.14730900	1.36533000	3.12360800
H	-4.41044100	3.45707400	0.35028400
H	-3.00437100	3.29419600	1.39319100
C	-4.72168800	4.16530400	2.37753200
H	-6.42021400	0.68434500	1.80480700
H	-6.38001400	1.92623900	0.55360200
C	-6.70732300	2.67533900	2.56897000
H	-3.06116100	1.34201800	3.25318400
H	-4.51593000	0.34844500	3.33918100
C	-4.77517000	2.35593100	4.11030200
H	-4.37254900	5.18444500	2.15180800
C	-6.24562500	4.10208300	2.26801000
C	-4.28952300	3.77488400	3.79392200
H	-7.80036000	2.60788100	2.46461900
C	-6.29942200	2.30240600	3.99539000
H	-4.47001200	2.08119400	5.13079900
H	-6.56398000	4.39957200	1.25725000
H	-6.71304000	4.80731400	2.97251000
H	-3.19329300	3.82602700	3.87943400
H	-4.70250100	4.48556800	4.52632200
H	-6.76351300	2.99397700	4.71543500
H	-6.66167900	1.29239100	4.24248400
C	0.05944600	5.52442800	-1.10690700
C	1.30969100	5.88043000	-0.27699200
C	-0.03241600	6.53385900	-2.26945800
C	-1.19899400	5.68064600	-0.23526900
H	2.20552800	5.77692300	-0.91078600
H	1.42680500	5.17134500	0.55058000
C	1.20501200	7.30930800	0.26099700
H	-0.91557300	6.30910200	-2.88709500
H	0.84621100	6.42472900	-2.92491100
C	-0.13348200	7.96633100	-1.73992200
H	-1.17317000	4.96110500	0.59251100
H	-2.09686600	5.44768200	-0.82762700

C	-1.29023100	7.11164300	0.30447600
H	2.09977800	7.53251500	0.86031900
C	1.10683100	8.29411300	-0.90617700
C	-0.04522700	7.42898900	1.13795600
H	-0.20321900	8.65928900	-2.59153200
C	-1.38310500	8.09627400	-0.86409400
H	-2.18812700	7.19930200	0.93421000
H	2.01097300	8.23150900	-1.53093300
H	1.04784300	9.32735200	-0.53058400
H	0.02479500	6.73638700	1.99021600
H	-0.11907700	8.44523700	1.55513200
H	-1.47463400	9.12693900	-0.48796600
H	-2.28615700	7.89184700	-1.45978400
C	5.21142000	1.60380100	1.71335100
C	4.97416200	0.99909100	3.11143100
C	6.71890300	1.48144500	1.40748400
C	4.84847900	3.09890300	1.72103400
H	5.25975400	-0.06539000	3.09723400
H	3.90917400	1.03238700	3.36557200
C	5.79413700	1.74982500	4.16334200
H	6.93277300	1.89899500	0.41178000
H	7.00402100	0.41844300	1.36753100
C	7.55111200	2.22208900	2.45713300
H	3.77535400	3.22728900	1.90698900
H	5.05107900	3.53188600	0.72941400
C	5.67131100	3.83515100	2.78281500
H	5.59110900	1.31351200	5.15241500
C	7.28421000	1.62473100	3.84020600
C	5.39242400	3.22806000	4.16091900
H	8.61728700	2.11854000	2.20636500
C	7.16201900	3.70286500	2.46112100
H	5.38611900	4.89740100	2.78310500
H	7.58795600	0.56670600	3.86126000
H	7.88654900	2.14600000	4.60027000
H	4.32486000	3.32871600	4.40803000
H	5.95427700	3.77357700	4.93488500
H	7.76306000	4.24922800	3.20439500
H	7.37847600	4.15414900	1.48053100
C	-1.72690700	2.90816500	-6.16486800
C	-2.91252800	2.26489800	-6.53183200
C	-3.86029500	1.89370900	-5.57355500

C	-3.65708800	2.16825000	-4.21825200
C	-6.84769000	-3.26025100	0.18338300
C	-6.76344900	-4.55232000	0.70975000
C	-5.68161400	-4.94025300	1.50588400
C	-4.65446700	-4.04287900	1.81149000
C	3.56986600	-6.28925800	1.59068900
C	4.89948600	-6.24004400	1.15914800
C	5.60552100	-5.03381700	1.12488100
C	5.00492300	-3.83327600	1.51410600
C	6.19792300	0.66898200	-4.04653600
C	5.82240200	1.34072500	-5.21328700
C	4.81911400	2.31410800	-5.19527100
C	4.16617100	2.65846100	-4.00845500
H	6.97032900	-0.09844200	-4.05132200
H	6.31295000	1.09845900	-6.15416500
H	4.53987600	2.80973700	-6.12286400
H	3.36869200	3.39977600	-3.98979400
H	5.53729700	-2.88458100	1.45590900
H	6.63671700	-5.02915500	0.77693100
H	5.39472200	-7.15561400	0.84146900
H	3.00841200	-7.22158000	1.61653900
H	-7.67951100	-2.95109200	-0.44777900
H	-7.55192400	-5.27175700	0.49669900
H	-5.64577000	-5.95390800	1.90037100
H	-3.81183000	-4.32701900	2.44209200
H	-4.38760800	1.89226600	-3.45866700
H	-4.77076400	1.38750900	-5.89057300
H	-3.10292000	2.04339300	-7.58114700
H	-0.97463000	3.18533600	-6.90144700
F	-0.78623700	0.51131000	-3.29738400
F	1.37474600	0.93199300	-3.50210000
O	1.15194300	-1.66047700	-5.17915000
O	-0.34640100	-0.03244900	-5.65533900
C	0.47442100	-0.71377800	-4.84987200
C	-1.08080100	-4.37713300	-1.40314400
C	-1.69012600	-4.76439300	-0.19400400
H	-2.46716200	-4.12890700	0.22849400
C	0.37162600	-6.32492900	-1.22699000
H	1.17171700	-6.94423800	-1.62501800
C	0.44185900	-0.08238900	-3.43077300
C	-0.28626600	-6.70134000	-0.05903000

C	1.95756100	-1.72262100	-2.20965600
C	-0.02758500	-5.17283000	-1.88935900
H	0.46464500	-4.90992400	-2.82348200
C	0.69099400	-0.97411300	-2.22245900
C	-1.55076800	-3.20253800	-2.13179100
C	-0.67849300	-2.44504000	-2.91526100
C	3.07986900	-1.40261400	-3.00039900
H	3.04922600	-0.55661500	-3.67981100
C	-1.30418000	-5.91200200	0.47782200
H	-1.76229600	-6.19120200	1.42254600
C	4.24021300	-2.16191500	-2.92997000
H	5.09819200	-1.88335700	-3.53947500
C	2.06912500	-2.82398800	-1.33681500
H	1.23026900	-3.06115200	-0.68567400
C	4.31065400	-3.27656100	-2.09954400
H	5.21813000	-3.87750200	-2.06182100
C	3.21099800	-3.61098000	-1.31208600
H	3.24671600	-4.49183600	-0.67251500
C	-0.49226000	-0.55988300	-6.99365400
H	-0.78783500	0.30877400	-7.58936400
H	0.48039900	-0.92544900	-7.33822800
C	-1.54208600	-1.64208100	-7.00137800
H	-2.47895900	-1.27308700	-6.56714600
H	-1.20845700	-2.50950100	-6.42121300
H	-1.74281800	-1.97659300	-8.02494000
H	0.21174700	-2.93941900	-3.28808200
H	-1.17845400	-1.81804400	-3.64813900
C	-2.98345200	-2.92760900	-2.22854200
C	-3.90554500	-3.99267600	-2.18525500
C	-3.46480300	-1.65618600	-2.61245200
C	-5.22992500	-3.82053800	-2.56103700
H	-3.56472200	-4.98945800	-1.91397300
C	-4.78669700	-1.47431900	-2.97920300
H	-2.79063100	-0.80060400	-2.60282700
C	-5.66175900	-2.56199900	-2.96509400
H	-5.92322000	-4.65777000	-2.54889300
H	-5.16073500	-0.49089600	-3.24830400
Cl	0.15843500	-8.18525900	0.72590600
Cl	-7.32004400	-2.33511800	-3.43505900

Re-int1

Zero-point correction= 1.890301 (Hartree/Particle)
Thermal correction to Energy= 1.999631
Thermal correction to Enthalpy= 2.000575
Thermal correction to Gibbs Free Energy= 1.745242
Sum of electronic and zero-point Energies= -6973.671966
Sum of electronic and thermal Energies= -6973.562636
Sum of electronic and thermal Enthalpies= -6973.561692
Sum of electronic and thermal Free Energies= -6973.817024

Rh	-0.25325900	0.07284900	0.33755100
Rh	-0.89985500	0.04501900	-2.04697900
O	1.76826700	0.16004600	-0.25507700
O	1.11691400	0.20808100	-2.42462700
O	6.12870900	-0.02677400	-0.75388700
O	2.36751300	-2.56240500	-1.52710400
O	-0.25258800	-2.03747100	0.19942900
O	-0.71550800	-2.00872500	-2.02685600
O	0.17700200	-5.94280000	1.27884300
O	-3.12893000	-3.11875500	-0.20547900
O	-2.30035400	-0.07369600	0.65412800
O	-2.89547400	-0.09209000	-1.53635100
O	-5.61545300	-0.41215300	2.87119600
O	-3.45757200	2.67806400	0.25019100
O	-0.32399700	2.11834600	0.18152700
O	-1.08283300	2.10555000	-1.95806600
O	-0.28212700	5.97237400	1.48168500
O	1.92322100	3.22805600	-1.47187500
N	4.10046500	-1.01737800	-1.33787600
N	-1.32617800	-4.54060400	0.18919900
N	-4.67610900	0.96591700	1.24887300
N	0.52310800	4.61829600	-0.23369700
C	1.98314300	0.17050000	-1.51279500
C	3.45859100	0.15130800	-1.94455400
H	3.93581400	1.01754900	-1.45732500
C	5.41013300	-1.00654600	-0.83741600
C	5.69939300	-2.40132300	-0.42983400
C	4.56745700	-3.17446400	-0.67999800
C	3.51493800	-2.28900000	-1.23079700
C	-0.48230600	-2.58037500	-0.92832600
C	-0.44637600	-4.11211300	-0.89518000

H	0.56615400	-4.37630900	-0.55008800
C	-0.91956600	-5.41941500	1.20195200
C	-2.07697100	-5.53174100	2.12174600
C	-3.07458900	-4.66559800	1.67583400
C	-2.58917800	-3.98823800	0.45169600
C	-3.13861800	-0.06401900	-0.29782200
C	-4.59194200	-0.03554800	0.18716100
H	-4.74330100	-0.99227200	0.71271900
C	-5.13660600	0.65544500	2.53892700
C	-4.88332800	1.86602400	3.35697900
C	-4.21610600	2.79711000	2.56316500
C	-4.04055200	2.21459100	1.21304300
C	-0.72442000	2.67096300	-0.88941000
C	-0.76609300	4.19642700	-0.77999100
H	-1.48801200	4.40500400	0.02546700
C	0.64317500	5.45677100	0.88677500
C	2.10198400	5.56072800	1.14908800
C	2.76644900	4.72551100	0.25180200
C	1.75522400	4.07102200	-0.60962100
C	3.73794800	0.25116600	-3.46845000
C	3.19781200	1.59427400	-4.00185900
C	5.26330000	0.24726000	-3.71059100
C	3.14577600	-0.92199800	-4.27381500
H	3.66496400	2.41761300	-3.43856100
H	2.12045400	1.67428500	-3.82811600
C	3.50166100	1.74079400	-5.49393000
H	5.69845700	-0.70100700	-3.35891100
H	5.73842200	1.04694100	-3.12278400
C	5.58679700	0.41102000	-5.19872000
H	2.06520700	-0.99529300	-4.11182800
H	3.58510000	-1.86934700	-3.92031900
C	3.45229600	-0.74861500	-5.76504700
H	3.07885300	2.69188500	-5.84934100
C	5.01490500	1.73329600	-5.71240700
C	2.86493500	0.57688000	-6.25957200
H	6.67953000	0.40316100	-5.32563600
C	4.96652400	-0.74722200	-5.98337700
H	2.99849900	-1.58317700	-6.31947100
H	5.47726300	2.57941200	-5.18129500
H	5.25129000	1.85745400	-6.78045800
H	1.77411400	0.58334800	-6.11465200

H	3.04639600	0.68959900	-7.33953800
H	5.19905900	-0.64604100	-7.05464700
H	5.39735400	-1.70464500	-5.65147100
C	-1.20308500	5.00796400	-2.01535000
C	-2.65456300	4.63718500	-2.38197900
C	-1.18412200	6.50669700	-1.65025900
C	-0.28972200	4.80440700	-3.23620300
H	-3.30205000	4.80039300	-1.50616300
H	-2.72249000	3.57124300	-2.62579700
C	-3.13849000	5.48057400	-3.56309200
H	-0.16086800	6.81198100	-1.38071300
H	-1.80746300	6.67945800	-0.75904900
C	-1.67397700	7.35974400	-2.82302500
H	-0.25383700	3.74229100	-3.50799600
H	0.73971600	5.10140200	-2.98184700
C	-0.78983500	5.64727600	-4.41412100
H	-4.16727400	5.18299300	-3.81504200
C	-3.10702000	6.96263100	-3.18504700
C	-2.22336900	5.23935100	-4.76740200
H	-1.64401700	8.42026200	-2.53193500
C	-0.76352700	7.12957300	-4.03228200
H	-0.13342300	5.47721100	-5.28026100
H	-3.77892200	7.14982400	-2.33351500
H	-3.46996100	7.57971100	-4.02156700
H	-2.25312600	4.17788200	-5.05604800
H	-2.57734800	5.81824800	-5.63455100
H	-1.09786500	7.74766100	-4.87984900
H	0.26594100	7.44080600	-3.79621500
C	-5.70850300	0.08329900	-0.86837400
C	-5.69848000	-1.17526400	-1.75930100
C	-7.06872400	0.12460000	-0.14182500
C	-5.59657000	1.34435700	-1.74277600
H	-5.80291300	-2.06773500	-1.12058800
H	-4.73539800	-1.27004900	-2.27358500
C	-6.84134200	-1.11943800	-2.77516000
H	-7.11502000	1.00980000	0.51096700
H	-7.16534600	-0.75502000	0.51441600
C	-8.22180500	0.17326100	-1.14766900
H	-4.62643400	1.36492700	-2.25436700
H	-5.63476500	2.24103900	-1.10572800
C	-6.74244100	1.38263700	-2.75877900

H	-6.79994400	-2.01645300	-3.41039500
C	-8.18114300	-1.07068600	-2.03798900
C	-6.68826000	0.13347400	-3.64333800
H	-9.17490800	0.20693000	-0.59912700
C	-8.08256300	1.42419500	-2.01976700
H	-6.63627700	2.28213000	-3.38328600
H	-8.31015600	-1.97813200	-1.42813400
H	-9.01393600	-1.04740800	-2.75787400
H	-5.73310700	0.09921400	-4.18867700
H	-7.48859000	0.17003200	-4.39867500
H	-8.91422200	1.47675800	-2.73942700
H	-8.14179200	2.32942500	-1.39596900
C	-0.68972200	-4.88505300	-2.20981300
C	0.39946000	-4.51719000	-3.23731800
C	-0.55546700	-6.39451600	-1.91662500
C	-2.08330600	-4.64319400	-2.81571200
H	1.39080500	-4.71574200	-2.79950800
H	0.36597800	-3.44502100	-3.45860200
C	0.22005400	-5.32884800	-4.52202400
H	-1.31620900	-6.70232200	-1.18247600
H	0.42444400	-6.59721100	-1.45522000
C	-0.72582900	-7.21762300	-3.19590200
H	-2.23600900	-3.57369100	-3.00505900
H	-2.85660000	-4.94875000	-2.09449200
C	-2.24443100	-5.45371000	-4.10595300
H	0.99676300	-5.03149200	-5.24215400
C	0.34914600	-6.82086800	-4.21006200
C	-1.16447700	-5.04503500	-5.11170600
H	-0.62990900	-8.28562100	-2.95004500
C	-2.10921700	-6.94628800	-3.79339900
H	-3.23897100	-5.25405600	-4.53075400
H	1.35022900	-7.03727500	-3.80597900
H	0.23939700	-7.41632400	-5.12957200
H	-1.25897900	-3.97561600	-5.35284400
H	-1.29617900	-5.59978800	-6.05356700
H	-2.24828800	-7.54233200	-4.70853400
H	-2.89465700	-7.25696300	-3.08720100
C	-5.13301700	2.10809100	4.69659600
C	-4.68928500	3.32410800	5.22421800
C	-4.00612400	4.24947100	4.43035400
C	-3.75556500	3.99610300	3.07849900

C	2.79894400	6.34200500	2.05607400
C	4.19529600	6.27838300	2.02060600
C	4.85888400	5.44493600	1.11585200
C	4.14806200	4.64070200	0.22157600
C	6.84940400	-2.95427400	0.10849800
C	6.83791800	-4.32618700	0.37597900
C	5.70649100	-5.10325200	0.11330800
C	4.54481200	-4.53310000	-0.41524100
C	-2.22844500	-6.27229100	3.28119400
C	-3.42551300	-6.12204600	3.98779900
C	-4.42380500	-5.25060900	3.54214500
C	-4.26018300	-4.50174700	2.37244800
H	-1.43673800	-6.93314200	3.62878000
H	-3.58288900	-6.68644300	4.90500300
H	-5.33920500	-5.14738500	4.12113600
H	-5.02167400	-3.80255800	2.03080000
H	3.64960300	-5.12784400	-0.59787100
H	5.72574300	-6.16755000	0.33763700
H	7.72392200	-4.80070200	0.79323100
H	7.72595100	-2.33956500	0.30513400
H	2.27273000	6.99096200	2.75342000
H	4.78117000	6.88718900	2.70787700
H	5.94703100	5.41902300	1.11585600
H	4.65491200	3.97882300	-0.47990200
H	-3.19836300	4.69763800	2.45905000
H	-3.65866700	5.18027100	4.87456900
H	-4.86787500	3.55241300	6.27385600
H	-5.64335700	1.36890900	5.31132900
F	-0.46177200	2.59457200	2.72748700
F	-2.01719800	1.05705100	2.93925200
O	0.49761300	1.85001300	5.23407700
O	-1.63845400	1.11810200	5.38984400
C	-0.54117300	1.46297000	4.72713100
C	3.21722100	-1.12771000	2.47633900
C	2.30163800	-2.03196300	1.89285300
H	1.45596400	-1.65206300	1.33472600
C	4.49200400	-3.04525900	3.28717100
H	5.32657600	-3.45802400	3.84797000
C	-0.71418900	1.32905500	3.19541700
C	3.54537600	-3.90019700	2.71569800
C	4.33341900	-1.67721700	3.15737100

H	5.04122300	-1.01710200	3.65362800
C	0.21565900	0.26582700	2.58567900
C	2.94448200	0.28477500	2.51442700
C	1.59936900	0.89164900	2.59174600
C	2.46418900	-3.40147500	1.98981800
H	1.74206400	-4.07967700	1.53731900
C	-1.55865400	1.20005900	6.83386700
H	-1.02680700	2.12069100	7.09783000
H	-2.60426200	1.28509100	7.14305300
C	-0.89050900	-0.02244000	7.40683400
H	0.16332600	-0.06357900	7.11454000
H	-1.37949900	-0.93548700	7.05047800
H	-0.94482900	-0.00178700	8.50115700
H	1.65320900	1.40201300	3.57749000
H	1.58285800	1.75476900	1.90520500
C	4.04861200	1.22316500	2.67244600
C	3.93156700	2.42068500	3.41627200
C	5.29633800	0.95827700	2.07228600
C	5.00573500	3.27893300	3.57045400
H	3.00488600	2.66728900	3.93058400
C	6.36981300	1.82619700	2.18855700
H	5.41593200	0.06546300	1.46984400
C	6.22073600	2.97898600	2.95462600
H	4.90952200	4.17837300	4.17248200
H	7.30699000	1.61099200	1.68230200
C	-0.49754600	-3.21978900	5.04434500
C	0.64765200	-2.45355200	5.23745800
H	-2.32621700	-3.41775700	3.92933300
C	-1.40779600	-2.84837000	4.05596800
C	0.87756300	-1.33866900	4.43894000
C	0.00045800	-0.98119400	3.39953800
C	-1.15989600	-1.75514300	3.23251000
H	1.75599700	-0.72642200	4.63920000
H	-1.86918200	-1.48126700	2.45130500
Cl	7.56276800	4.06922400	3.14701400
Cl	3.71407300	-5.61273100	2.93079900
H	1.36062300	-2.71208900	6.02003300
H	-0.69076400	-4.09269900	5.66696800

Re-TS2

Zero-point correction= 1.891485 (Hartree/Particle)
Thermal correction to Energy= 2.000861
Thermal correction to Enthalpy= 2.001805
Thermal correction to Gibbs Free Energy= 1.745713
Sum of electronic and zero-point Energies= -6973.639018
Sum of electronic and thermal Energies= -6973.529642
Sum of electronic and thermal Enthalpies= -6973.528698
Sum of electronic and thermal Free Energies= -6973.784789

Rh	0.06650700	-0.04153600	-0.26896500
Rh	-0.52903200	-0.04293100	2.08308300
O	1.04890600	-1.84162900	0.07022300
O	0.30311300	-1.92688600	2.20880500
O	3.29167600	-5.26700600	-0.52634800
O	3.42105900	-1.49612900	2.09362000
O	1.81391500	0.95786000	0.27206800
O	1.29595100	0.84973300	2.47767400
O	5.48030700	2.99335900	0.11277300
O	1.04517600	3.73913200	1.04140600
O	-0.96217300	1.75735800	-0.27979300
O	-1.38453200	1.83567800	1.93682300
O	-1.42837100	5.52109800	-1.71833400
O	-3.87715400	1.80002600	-0.60050600
O	-1.79985000	-0.95023300	-0.62996300
O	-2.34862800	-0.82556000	1.56698400
O	-5.48637000	-1.35700100	-2.56008800
O	-2.96573900	-3.95075200	0.27970700
N	3.05940400	-3.51214100	0.98738100
N	3.28462100	3.15953400	0.87018300
N	-2.60604600	3.73159900	-0.79933100
N	-4.23376200	-2.38275000	-0.88393900
C	0.93996500	-2.37902700	1.21687200
C	1.65814700	-3.72629500	1.33841000
H	1.26362600	-4.34312300	0.51590100
C	3.76240400	-4.32244300	0.07981700
C	5.13348800	-3.76367500	0.02728100
C	5.17112600	-2.62154100	0.82631500
C	3.82787800	-2.41645200	1.40928200
C	2.01547800	1.20750100	1.50572000
C	3.28550300	2.02319800	1.79023100
H	4.12989800	1.39827200	1.45350900

C	4.38989600	3.53309700	0.09087300
C	3.91395700	4.64572500	-0.76846600
C	2.54542000	4.80752000	-0.55642900
C	2.13574200	3.88336000	0.52194200
C	-1.41316900	2.31122600	0.76050700
C	-1.96601500	3.72168100	0.51419900
H	-1.06211100	4.33316800	0.36997600
C	-2.21297100	4.59721500	-1.83078700
C	-2.93756400	4.14164700	-3.03955200
C	-3.67605900	3.00833700	-2.70559500
C	-3.44936500	2.71933900	-1.27311100
C	-2.61779400	-1.01031600	0.34206300
C	-4.08931800	-1.22524900	-0.01529400
H	-4.33370100	-0.36880100	-0.66113700
C	-5.02769500	-2.35630300	-2.03982200
C	-5.18085900	-3.77288600	-2.44715900
C	-4.46107500	-4.56627400	-1.55221400
C	-3.77423100	-3.67299400	-0.58520200
C	1.46384000	-4.54167500	2.63219700
C	-0.03388900	-4.86814800	2.79582900
C	2.21625200	-5.88066600	2.48098400
C	1.99249000	-3.84133900	3.89466000
H	-0.39128300	-5.38957100	1.89198900
H	-0.62088900	-3.94648700	2.87978400
C	-0.25598000	-5.74534200	4.02964500
H	3.29427400	-5.69156100	2.35895700
H	1.88242400	-6.39296500	1.56465000
C	1.99508200	-6.77175400	3.70544400
H	1.50658100	-2.86678000	4.02167800
H	3.07076700	-3.64642300	3.78328600
C	1.75586900	-4.72771900	5.12232500
H	-1.33192900	-5.94482500	4.13576400
C	0.50167700	-7.06439100	3.86526300
C	0.25763200	-5.01012800	5.27157200
H	2.54647900	-7.71328100	3.56651300
C	2.50773700	-6.05073600	4.95465800
H	2.12368200	-4.20425900	6.01680900
H	0.12542200	-7.60948800	2.98615800
H	0.33559300	-7.71257600	4.73937300
H	-0.29290900	-4.06678900	5.40528100
H	0.07667800	-5.61729300	6.17182100

H	2.36460800	-6.68555700	5.84251600
H	3.58904900	-5.86284800	4.86730500
C	-5.12902900	-1.22281700	1.14332300
C	-5.11972000	0.15148500	1.84507100
C	-6.53868000	-1.40617200	0.53805200
C	-4.91982700	-2.34014200	2.18066900
H	-5.30592200	0.93665800	1.09948000
H	-4.13188500	0.35401000	2.27243800
C	-6.18253000	0.21064800	2.94325500
H	-6.60852200	-2.38330400	0.03311600
H	-6.71631800	-0.63816700	-0.23057700
C	-7.61788600	-1.33829100	1.62321200
H	-3.91502800	-2.27426000	2.61414000
H	-4.98909900	-3.32247900	1.69150000
C	-5.98800400	-2.25826600	3.27693600
H	-6.12551000	1.19387100	3.43523300
C	-7.56710600	0.02144700	2.32267000
C	-5.92072500	-0.89458800	3.96912000
H	-8.60261500	-1.47897400	1.15331000
C	-7.37322500	-2.44432300	2.65275300
H	-5.80415600	-3.05572000	4.01188400
H	-7.77038000	0.82800300	1.60182000
H	-8.34833300	0.07709600	3.09648900
H	-4.93139000	-0.75375900	4.43044000
H	-6.66334800	-0.84523500	4.78055700
H	-8.15099200	-2.41434100	3.43138700
H	-7.44048100	-3.43241500	2.17141900
C	-2.78883800	4.40575400	1.62559300
C	-1.88119300	4.66213000	2.84697100
C	-3.26258100	5.77894000	1.10624300
C	-4.02445300	3.60405600	2.06275200
H	-1.00623300	5.25188600	2.52851000
H	-1.49461000	3.71386300	3.23658700
C	-2.65158100	5.40810300	3.93922000
H	-3.91409300	5.64051500	0.22978200
H	-2.39450300	6.36389800	0.76363500
C	-4.02869000	6.53789700	2.19232000
H	-3.71877600	2.61368900	2.42518800
H	-4.68392600	3.43275400	1.19872700
C	-4.78533900	4.35986100	3.15674400
H	-1.98846600	5.56036800	4.80350700

C	-3.12049100	6.76224700	3.40324200
C	-3.86694600	4.57711900	4.36254200
H	-4.35649200	7.50726800	1.78851600
C	-5.24825700	5.71609700	2.61808900
H	-5.66064300	3.76653300	3.46040200
H	-2.25286400	7.37655900	3.11777000
H	-3.66252700	7.31671300	4.18482300
H	-3.53953500	3.60652200	4.76554600
H	-4.41448900	5.09069500	5.16788200
H	-5.81826900	6.25721800	3.38894500
H	-5.92536800	5.57156100	1.76215900
C	3.56604800	2.43105000	3.25429600
C	3.77991800	1.16990500	4.11509100
C	4.87895400	3.24194600	3.29392300
C	2.45540400	3.30484900	3.86474700
H	4.59601100	0.57076500	3.68005900
H	2.88796400	0.53528100	4.09717600
C	4.12407600	1.55746300	5.55499000
H	4.77255800	4.15586900	2.68962000
H	5.69147500	2.65677400	2.83538700
C	5.24029700	3.62869000	4.73054500
H	1.49112700	2.78361600	3.82405000
H	2.34058900	4.22294600	3.26857500
C	2.81033700	3.67419800	5.30854200
H	4.24846500	0.64095200	6.15007500
C	5.42274100	2.36557200	5.57387800
C	2.98608000	2.39987500	6.13929800
H	6.17557300	4.20765800	4.71932600
C	4.11375800	4.47679300	5.32681500
H	1.99621000	4.28122800	5.73105100
H	6.25085000	1.75939600	5.17534800
H	5.69201700	2.63580400	6.60656800
H	2.05118800	1.81940900	6.14201100
H	3.20609300	2.65913000	7.18639500
H	4.36728600	4.77053500	6.35704100
H	3.99288600	5.40649900	4.74972700
C	-2.91595000	4.63302000	-4.33396700
C	-3.65646000	3.94136400	-5.29615300
C	-4.38786700	2.79895000	-4.95983000
C	-4.41024400	2.31158100	-3.64978000
C	-5.90388500	-4.32888400	-3.48811800

C	-5.90518400	-5.72341100	-3.59867900
C	-5.20492200	-6.51780200	-2.68889100
C	-4.46512800	-5.94573400	-1.64897300
C	6.24710800	-4.18916700	-0.67585500
C	7.41705700	-3.43639000	-0.54236200
C	7.45406300	-2.29112900	0.25684400
C	6.32119800	-1.86147500	0.95345200
C	4.58480400	5.38565600	-1.72771300
C	3.83089200	6.27670100	-2.49647300
C	2.45277100	6.40742500	-2.30483600
C	1.78319200	5.67635300	-1.31926500
H	5.65433200	5.25806500	-1.88639200
H	4.32210000	6.86965600	-3.26589700
H	1.88831600	7.09415300	-2.93313000
H	0.70714200	5.76167600	-1.17615800
H	6.33370800	-0.96155200	1.56673900
H	8.37759700	-1.72021100	0.32690600
H	8.31107300	-3.73483900	-1.08552600
H	6.20395300	-5.07012300	-1.31327200
H	-6.45567000	-3.69881200	-4.18295100
H	-6.46636600	-6.19905800	-4.40060700
H	-5.22801100	-7.60047500	-2.79726300
H	-3.90311000	-6.55570500	-0.94531000
H	-4.96028000	1.41150200	-3.37949900
H	-4.94226800	2.27547700	-5.73605100
H	-3.65499200	4.28800200	-6.32794100
H	-2.33197500	5.51693100	-4.58488900
F	-1.24403700	1.35209300	-2.83794800
F	0.37871400	2.72472600	-2.19426900
O	-0.63408400	2.06665900	-5.47545300
O	0.42573200	3.78903300	-4.42605300
C	-0.14221100	2.58108500	-4.49489300
C	2.39499300	-1.73396400	-2.94855500
C	3.23095400	-1.09013700	-2.03306700
H	2.79047000	-0.48297800	-1.24304500
C	4.36196800	-2.59901500	-4.09083800
H	4.80921800	-3.18578600	-4.88913500
C	-0.00108000	1.83122800	-3.15042600
C	5.16538200	-1.85920600	-3.22885400
C	2.98231600	-2.52948700	-3.94350500
H	2.34483000	-3.06871200	-4.64477600

C	0.98067000	0.72232000	-3.36117300
C	0.91393200	-1.55511100	-3.03360300
C	0.42696100	-0.51561800	-4.00002000
C	4.61503800	-1.13172300	-2.17969800
H	5.25703500	-0.56543400	-1.50795100
C	0.59728700	4.48077800	-5.68287300
H	-0.23287900	4.21678200	-6.34745800
H	0.52875300	5.54075600	-5.41928300
C	1.93841200	4.12282400	-6.27118700
H	1.97849200	3.05724100	-6.52329100
H	2.74222500	4.33225400	-5.55576000
H	2.12389700	4.69834200	-7.18501100
H	0.72769900	-0.70871500	-5.04651700
H	-0.66360800	-0.46967200	-4.00062400
C	0.11145100	-2.69595200	-2.69540900
C	-1.18345600	-2.92024000	-3.22612600
C	0.73892600	-3.78924400	-2.04391900
C	-1.72681700	-4.19479900	-3.26175000
H	-1.74634400	-2.10070000	-3.66777900
C	0.19276600	-5.05609600	-2.04232100
H	1.71656600	-3.64286900	-1.59923400
C	-1.01742600	-5.26240100	-2.70360200
H	-2.68065700	-4.37771500	-3.75107500
H	0.72935800	-5.88335300	-1.58317800
C	5.01573900	1.82645400	-4.32368200
C	4.28061400	1.00849400	-5.17798600
H	5.00240200	2.91350100	-2.47326600
C	4.41543300	2.31528600	-3.16465800
C	2.97742300	0.66214400	-4.85608600
C	2.34745700	1.14124800	-3.68623700
C	3.09800200	2.00190600	-2.85627200
H	2.43858200	-0.00467500	-5.52462000
H	2.65623800	2.34899600	-1.92564800
Cl	-1.60338400	-6.88800400	-2.88722500
Cl	6.88903700	-1.80957800	-3.51128300
H	4.73426700	0.61221100	-6.08557400
H	6.05536700	2.06144300	-4.54451200

Re-pro

Zero-point correction= 0.389336 (Hartree/Particle)

Thermal correction to Energy= 0.417021

Thermal correction to Enthalpy= 0.417965

Thermal correction to Gibbs Free Energy= 0.328028

Sum of electronic and zero-point Energies= -2234.641811

Sum of electronic and thermal Energies= -2234.614127

Sum of electronic and thermal Enthalpies= -2234.613182

Sum of electronic and thermal Free Energies= -2234.703119

F	0.09737700	-2.81154500	0.53745500
F	0.81140900	-2.00910900	-1.37597600
O	2.70952600	-2.60998800	1.51804400
O	3.17505100	-2.59812500	-0.70736700
C	2.39616900	-2.43972400	0.36189100
C	-0.82887400	1.49598500	0.45424700
C	-1.02791700	1.98686700	-0.84078900
H	-1.02006900	1.29679700	-1.68374300
C	-1.07912800	3.76177400	1.29953100
H	-1.10934600	4.45998400	2.13188000
C	0.99161300	-1.94444600	-0.02024400
C	-1.25823300	4.21907900	-0.00036300
C	-0.86613200	2.40297000	1.51440000
H	-0.73756400	2.04532200	2.53510000
C	0.78900400	-0.52961000	0.48168700
C	-0.63134400	0.02157000	0.68818500
C	0.21120200	-0.39581800	1.85753000
C	-1.23718300	3.33857000	-1.07892700
H	-1.38320300	3.71177600	-2.08912700
C	4.55226600	-2.95231700	-0.41996800
H	4.55777300	-3.68987500	0.38930900
H	4.89794800	-3.42407300	-1.34347900
C	5.35201000	-1.72402300	-0.06825500
H	5.00067600	-1.28702200	0.87182900
H	5.26848000	-0.96459500	-0.85328000
H	6.40950500	-1.98511500	0.04978400
H	0.62975100	0.37354900	2.50177500
H	-0.05466400	-1.31795800	2.36720900
C	-1.85296300	-0.78708200	0.34917000
C	-2.78154300	-1.07135800	1.35315000
C	-2.14007100	-1.19288000	-0.95679800
C	-3.96369500	-1.74804700	1.07490500
H	-2.57406900	-0.75451800	2.37534600
C	-3.31591100	-1.87175800	-1.25589100

H	-1.42119600	-1.00393500	-1.75163800
C	-4.22028400	-2.14305900	-0.23399800
H	-4.68058500	-1.96900300	1.86136000
H	-3.52921800	-2.19597000	-2.27105000
C	3.98657400	2.05579700	-0.79686000
C	3.86113800	1.71290300	0.54618200
H	3.16576600	1.81820300	-2.77659700
C	3.07274600	1.55761900	-1.72379200
C	2.82295400	0.88230900	0.96037600
C	1.89219000	0.38896100	0.04245400
C	2.03614200	0.72941200	-1.30806800
H	2.74189000	0.59567700	2.00854400
H	1.32859100	0.33648200	-2.03598600
Cl	-5.69762100	-2.99748800	-0.59926000
Cl	-1.51895000	5.92020000	-0.28653500
H	4.57646400	2.08819500	1.27608700
H	4.79468900	2.70858900	-1.12206700
